# Fragmented mitochondrial genomes in two suborders of parasitic lice of eutherian mammals (Anoplura and Rhynchophthirina, Insecta)

**DOI:** 10.1038/srep17389

**Published:** 2015-11-30

**Authors:** Renfu Shao, Stephen C Barker, Hu Li, Simon Song, Shreekanta Poudel, Yuan Su

**Affiliations:** 1 GeneCology Research Centre, Faculty of Science, Health, Education and Engineering, University of the Sunshine Coast, Maroochydore, Queensland 4556, Australia; 2 Parasitology Section, School of Chemistry and Molecular Biosciences, The University of Queensland, Queensland 4072, Australia; 3 Department of Entomology, China. Agricultural University, Beijing 100193, China; 4 Manitoba Health, Winnipeg, Manitoba, R2W 3C7, Canada

## Abstract

Parasitic lice (order Phthiraptera) infest birds and mammals. The typical animal mitochondrial (mt) genome organization, which consists of a single chromosome with 37 genes, was found in chewing lice in the suborders Amblycera and Ischnocera. The sucking lice (suborder Anoplura) known, however, have fragmented mt genomes with 9–20 minichromosomes. We sequenced the mt genome of the elephant louse, *Haematomyzus elephantis* – the first species of chewing lice investigated from the suborder Rhynchophthirina. We identified 33 mt genes in the elephant louse, which were on 10 minichromosomes. Each minichromosome is 3.5–4.2 kb in size and has 2–6 genes. Phylogenetic analyses of mt genome sequences confirm that the elephant louse is more closely related to sucking lice than to the chewing lice in the Amblycera and Ischnocera. Our results indicate that mt genome fragmentation is shared by the suborders Anoplura and Rhynchophthirina. Nine of the 10 mt minichromosomes of the elephant louse differ from those of the sucking lice (Anoplura) known in gene content and gene arrangement, indicating that distinct mt karyotypes have evolved in Anoplura and Rhynchophthirina since they diverged ~92 million years ago.

The parasitic lice (order Phthiraptera) have ~5,000 described species in four suborders. The sucking lice, which feed exclusively on the blood of eutherian mammals, are in the suborder Anoplura^1^. The chewing lice, which infest both birds and mammals and feed mainly on feathers, dead skin, blood or secretions, are in three other suborders: Amblycera, Ischnocera and Rhynchophthirina^2^. The mitochondrial (mt) genomes of the wallaby louse, *Heterodoxus macropus* (Amblycera), and four species in the suborder Ischnocera, *Campanulotes bidentatus* (small pigeon louse), *Bothriometopus macrocnemis* (screamer louse), *Coloceras* sp. (pigeon feather louse) and *Ibidoecus bisignatus* (ibis head louse), have been sequenced^3^,^4^,^5^,^6^. Like most other bilateral animals, these five species of chewing lice all have the typical mt chromosomes with 37 genes, 14,670 to 15,564 bp in size^6^,^7^. In contrast, all of the sucking lice that have been sequenced (10 species/subspecies in total) have fragmented mt genomes and there is no evidence for the presence of the typical mt chromosome in these lice. The human body louse, *Pediculus humanus*, and the human head louse, *Pe. h. capitis*, have their 37 mt genes on 20 minichromosomes^8^,^9^. The 37 mt genes of the rat lice, *Polyplax asiatica* and *Po*. *spinulosa*, are on 11 minichromosomes^10^, whereas those of the pig lice, *Haematopinus suis* and *Haematopinus apri*, and the horse louse, *Haematopinus asini*, are on nine minichromosomes^11^,^12^. The 34 mt genes identified in the human pubic louse, *Pthirus pubis*, are on 14 minichromosomes^9^; the 34 mt genes identified in the rat louse, *Hoplopleura kitti*, are on 11 minichromosomes; and the 28 mt genes identified in the mouse louse, *Ho. akanezumi*, are on 10 minichromosomes^13^.

Despite being classified as chewing lice based on mouth parts^14^, the suborder Rhynchophthirina is phylogenetically more closely related to the sucking lice (Anoplura) than to the other two suborders of the chewing lice^15^,^16^,^17^,^18^,^19^,^20^. Rhynchophthirina has one family, Haematomyzidae, one genus, *Haematomyzus*, and three species: *Haematomyzus elephantis* (elephant louse), *Haematomyzus hopkinsi* (African wart-hog louse) and *Haematomyzus porci* (African red-river hog louse)^2^. We sequenced the mt genome of the elephant louse, *H. elephantis*, and identified 33 mt genes in this louse; these genes were on 10 minichromosomes. Our results indicate that mt genome fragmentation is shared by parasitic lice in the suborders Anoplura and Rhynchophthirina. Further, nine of the 10 mt minichromosomes of the elephant louse differ from those of the sucking lice known in gene content and gene arrangement, indicating that distinct mt karyotypes have evolved between Anoplura and Rhynchophthirina since they diverged ~92 million years ago^20^,^21^.

## Results

### The mitochondrial genome of the elephant louse, *Haematomyzus elephantis*

**Figure 1:**
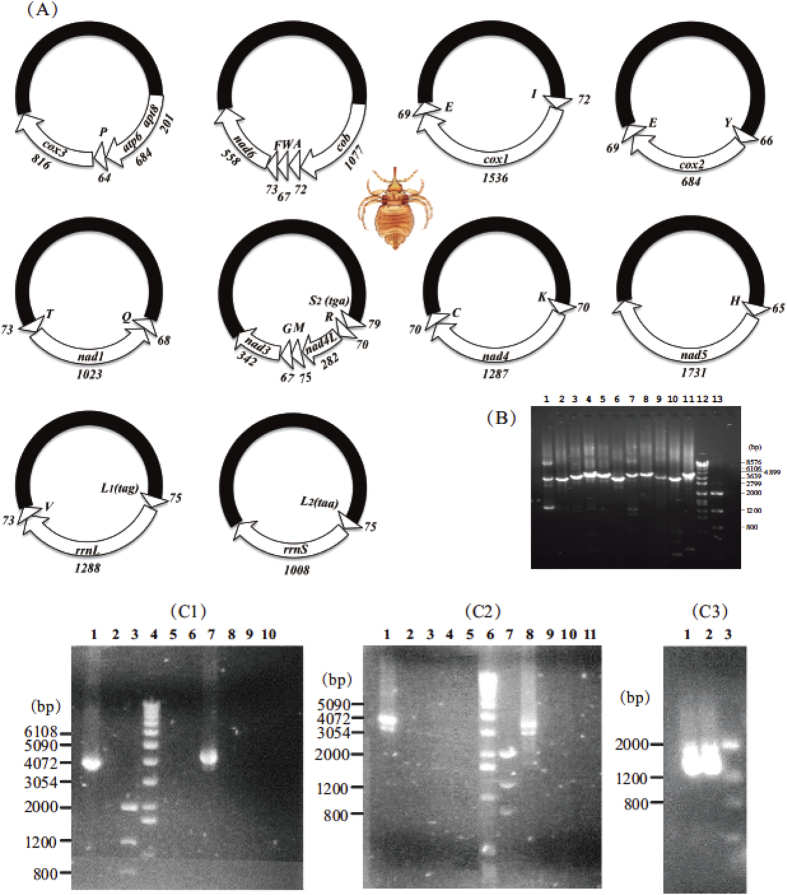
(**A**) Mitochondrial minichromosomes of the elephant louse, *Haematomyzus elephantis*. Arrows indicate protein-coding and rRNA genes: *cox1-3* for cytochrome c oxidase subunits 1–3, *cob* for cytochrome b, *nad1-5* and *nad4L* for NADH dehydrogenase subunits 1–5 and 4L, *rrnS* and *rrnL* for small and large ribosome RNA subunits. Triangles indicate tRNA genes (labeled with single-letter abbreviations of their corresponding amino acids). Numbers near each gene indicate gene length in bp. Non-coding regions are in black. Drawing of elephant louse was by Hu Li. (**B**) Verification of mitochondrial minichromosomes of *H. elephantis* by PCR. Lanes 1–11: amplicons from minichromosomes *T-**nad1**-Q*, *S*_*2*_*(tga)-R-nad4L-M-G-**nad3***, *K-**nad4**-C*, *H-**nad5***, *I-**cox1**-E*, *Y-**cox2**-E*, *atp8-atp6-P-**cox3***, ***cob**-A-W-F-nad6*, *L*_*1*_*(tag)-**rrnL**-V*, *L*_*2*_*(taa)-**rrnS***, and ***H**-nad5* (genes from which PCR primers were designed are in bold). Lanes 12-13: DNA Molecular Weight Marker VII (Roche) and Low DNA Mass Ladder (Life Technologies). (**C**) PCR amplicons with elephant-louse-specific primers (see also Table S5). (C1) Lane 1: primer pair EL16SF-EL16SR, which amplified *trnL*_*1*_*(tag)-rrnL-trnV* minichromosome in near full length. Lanes 2, 5 and 6: negative controls for EL16SF-LX16SR that had no forward primer, no reverse primer and no DNA template respectively. Lanes 3 and 4: Low Mass Ladder (LML) and DNA Molecular Weigh Marker X (DMWMX). Lane 7: primer pair ELC1F-ELC1R, which amplified *trnI-cox1-trnE* minichromosome in near full length. Lanes 8, 9 and 10: negative controls for ELC1F-ELC1R that had no forward primer, no reverse primer and no DNA template respectively. (C2) Lane 1: primer pair ELC2F-ELC2R1, which amplified *trnY-cox2-trnE* minichromosome in near full length. Lanes 2, 3 and 4: negative controls for ELC2F-ELC2R1 that had no forward primer, no reverse primer and no DNA template respectively. Lanes 6 and 7: DMWMX and LML. Lane 8: primer pair EL12SF1-EL12SR, which amplified *trnL*_*2*_*(taa)-rrnS* minichromosome in near full length. Lanes 9, 10 and 11: negative controls for EL12SF1-EL12SR that had no forward primer, no reverse primer and no DNA template respectively. (C3) Lanes 1 and 2: amplicons produced by the primer pair USFB1567-ELR from the full-length coding regions of all mitochondrial minichromosomes. Lane 3: LML.

We obtained 14,760 and 1,137,360 sequence-reads from the amplicons of the mt genome of the elephant louse, *H. elephantis*, by Roche 454 sequencing and Illumina Hiseq sequencing, respectively (Table 1, Table 2). Roche sequence-reads are 100–611 bp in size (mean 344 bp, standard deviation 108); Illumina sequence-reads are all 90 bp in size. We assembled these sequence-reads into contigs and identified 33 of the 37 mt genes typical of bilateral animals. These genes are on 10 minichromosomes; each minichromosome is 3.5–4.2 kb in size and consists of a coding region and a non-coding region (NCR) in a circular organization (Fig. 1A,B). The coding regions have 2–6 genes each and vary in size from 857 bp to 1,879 bp (Table 1; Fig. 1A). All of the mt genes of the elephant louse have the same orientation of transcription relative to the NCRs, except for *trnT*, *nad1* and *trnQ*, which form a cluster and have an opposite orientation of transcription to that of other genes (Fig. 1A; see Fig. 1 legend for the full name of each mt gene). With the exception of *trnE*, all of the mt genes we identified in the elephant louse were found in only one type of minichromosome. *trnE* gene was found in two types of minichromosomes in the elephant louse: one in the *trnI*-*cox1*-*trnE* minichromosome and the other in the *trnY*-*cox2*-*trnE* minichromosome (Note: minichromosomes are named after the genes they contain hereafter). Further, the two copies of *trnE* gene have identical sequences to each other. We did not find *trnD*, *trnN*, *trnS*_*1*_*(tct)* and *nad2* genes in the sequence-reads of the elephant louse generated by Roche sequencing, nor Illumina sequencing. A possible explanation is that the primer pair USFB1567–ELR, which we used to amplify the coding regions of the mt minichromosomes of the elephant louse, are not conserved in the minichromosome(s) that contain *trnD*, *trnN*, *trnS*_*1*_*(tct)* and *nad2* genes; these minichromosome(s) were thus not amplified by the PCR with USFB1567–ELR.

**Figure 2:**
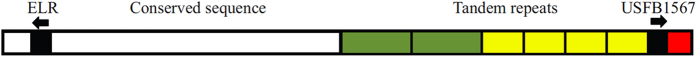
Schematic illustration of the non-coding regions of the mitochondrial minichromosomes of the elephant louse, *Haematomyzus elephantis*. USFB1567 and ELR are the primers used to amplify the coding regions of all mitochondrial minichromosomes. A 346-bp unit (in green) repeated twice and a 225-bp unit (in yellow) repeated 4 times; the sequence similarity between/among the repeat copies of each unit is ~85%. A 99-bp sequence (in red) is present only in *T-nad1-Q* minichromosome.

We sequenced the NCRs of four mt minichromosomes of the elephant louse in full length. The NCRs are 2,324, 2,457, 2,348 and 2,359 bp respectively for *trnI*-*cox1*-*trnE*, *trnT-nad1-trnQ*, *trnK-nad4-trnC* and *trnH-nad5* minichromosomes, and account for 58%, 68%, 61% and 57% of the total size of each minichromosome (Fig. S1; Alignment S1). The NCR of *T-nad1-Q* minichromosome has a 99-bp sequence at the 3′ end, which is not seen in the NCRs of other minichromosomes. Otherwise, the NCRs of the four minichromosomes have high sequence similarity (>97%) to each other. Contrary to those of most animals, the NCRs of the elephant louse have higher GC content (thus lower AT content) than the coding regions: 40% vs 38% for *trnI*-*cox1*-*trnE* minichromosome, 39% vs 34% for *trnT-nad1-trnQ* minichromosome, 40% vs 34% for *trnK-nad4-trnC* minichromosome, and 41% vs 35% for *trnH-nad5* minichromosome. Whether or not the high GC content in the NCRs has any functional significance to the fragmented mt genome of the elephant louse remains to be investigated. There are tandem-repeat units in the NCRs of the elephant louse, 225 and 346 bp in length, respectively. The 225-bp unit repeated four times whereas the 346-bp unit repeated twice; the different copies of each unit have ~85% identity to each other (Fig. 2). We also sequenced the NCRs of the other six minichromosomes of the elephant louse except for the GC-rich parts and the tandem-repeat parts using a Sanger sequencing platform (Fig. S2; Alignment S2). The exact length of the NCRs of these six minichromosomes was unknown. The full-length NCR sequences shown in Fig. S1 were the consensus sequences obtained from the assembly of the Illumina sequence-reads. The high coverage of the Illumina sequence-reads also revealed numerous heteroplasmic sites in these NCRs of the elephant louse (Table S2–S5), which is consistent with that observed in human lice and pig lice^8^,^9^,^11^,^22^.

### Concerted evolution between the two mitochondrial tRNA genes for leucine in the elephant louse and the sucking lice

**Figure 3:**
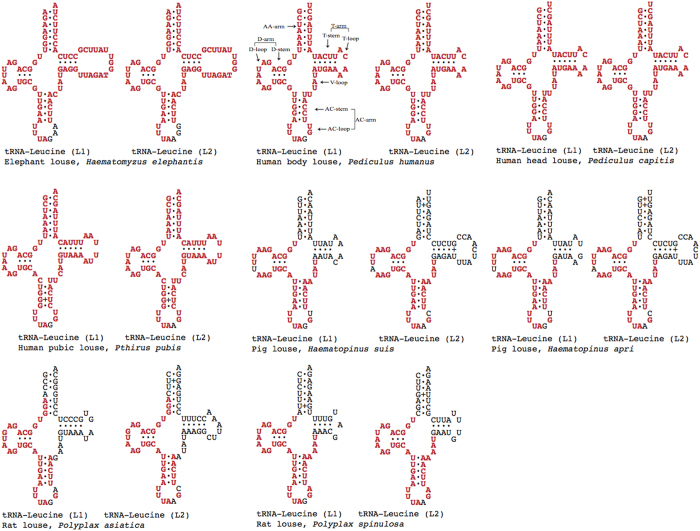
Inferred secondary structures of the mitochondrial tRNAs for leucine of the elephant louse, three human lice, two pig lice and two rat lice. Shared identical sequences between the two tRNA genes of each species are in red.

It is common for animals to have two tRNA genes for leucine, *trnL*_*1*_*(tag)* and *trnL*_*2*_*(taa)*, in their mt genomes^23^. The two tRNA genes for leucine of the elephant louse have identical sequences except for three nucleotides starting from the third anti-codon position (Fig. 3). Near identical sequences between the two *trnL* genes were also observed in the human body louse, *Pe. h. humanus*, the head louse, *Pe. h. capitis*, and the pubic louse *Pthirus pubis*^8^,^9^. In the pig lice, *Haemapinus suis* and *Haemapinus apri*, and the rat lice, *Polyplax asiatica* and *Po. spinulosa*, the two *trnL* genes have identical or near identical sequences at the D-arm, the anti-codon arm and the V-loop but differ at the amino-acid arm and the T-arm (Fig. 3)^10^,^11^.

**Figure 4:**
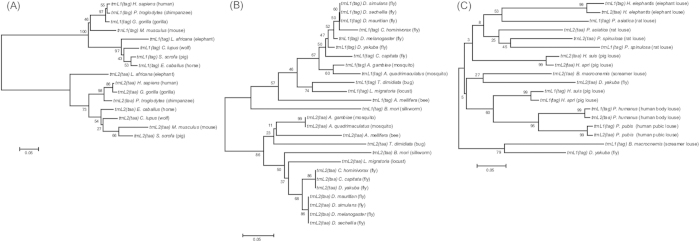
Independent evolution of the two mitochondrial (mt) tRNA genes for leucine in mammals (A) and insects (B), and a mixture of both independent and concerted evolution of these two genes in parasitic lice (C). Trees were constructed using neighbor-joining method with MEGA 5.2.2 (Tamura *et al.* 2011). Bootstrap support values (%) are indicated on branches. All of the mammals and insects in (**A**) and (**B**) and the fly and the screamer louse in (**C**) have the typical, single-chromosome mt genomes of bilateral animals. The elephant louse and the sucking lice (Anoplura) in (**C**) have fragmented mt genomes with multiple minichromosomes. The human head louse, *Pe. h. capitis*, was not included in (**C**) because it has identical sequences with the human body louse, *Pe. h. humanus*, for *trnL*_*1*_ and *trnL*_*2*_ genes.

We used distance-based neighbor-joining (NJ) method to analyze the sequences of *trnL*_*1*_*(tag)* and *trnL*_*2*_*(taa)* genes. The NJ tree showed that these two genes evolved independently in a wide range of species of mammals and insects that have the typical single-chromosome mt genome organization. As would be expected for any pair of homologous genes, *trnL*_*1*_*(tag)* of a given species was more closely related to *trnL*_*1*_*(tag)* of other species than it is to *trnL*_*2*_*(taa)* of its own, and *vice versa* (Fig. 4A,B; Alignment S3 and S4). In the elephant lice and the sucking lice that have fragmented mt genomes, however, both independent evolution and concerted evolution were observed between the two tRNA genes for leucine (Fig. 4C; Alignment S5). In the two species of pig lice, *Haematopinus suis* and *Haematopinus apri*, both *trnL* genes evolved independently: *trnL*_*1*_*(tag)* genes of the two species are most closely related to each other whereas *trnL*_*2*_*(tag)* genes of the two species are most closely related. In the elephant louse, the human lice and the rat louse, *Po. spinulosa*, however, the two *trnL* genes evolved in concert: *trnL*_*1*_*(tag)* of each species is more closely related to *trnL*_*2*_*(taa)* of the same species than to *trnL*_*1*_*(tag)* of other species, and *vice versa*.

The complexity in the evolution of the two *trnL* genes in the elephant louse and the sucking lice has apparently been driven by recombination events between these genes^8^,^9^,^24^. Depending on the frequency of recombination between *trnL*_*1*_*(tag)* and *trnL*_*2*_*(taa),* different patterns of evolution can be observed. Concerted evolution of these two genes would be expected over long term or between distantly related species whereas independent evolution would be expected over short term (between two recombination events) or between closely related species, such as the two species of pig lice. Also, if recombination occurs only between parts of the genes, then concerted evolution would be expected only for the recombinant parts whereas the other parts of the genes will remain to evolve independently from each other.

### Phylogenetic relationship of the elephant louse to other parasitic lice, barklice and booklice inferred with mitochondrial genome sequences and gene rearrangements

**Figure 5:**
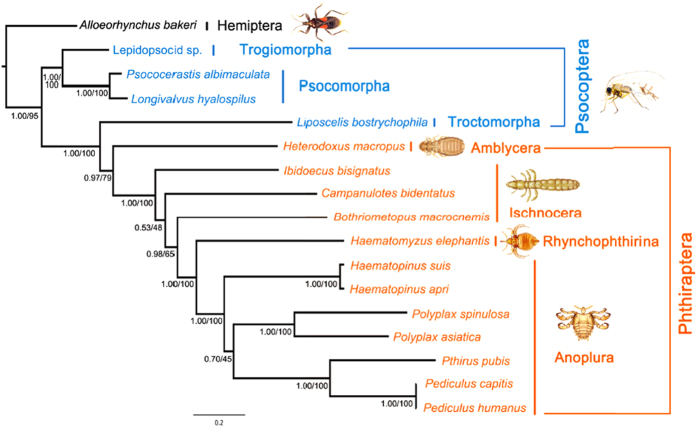
Phylogenetic relationship of the elephant louse to other parasitic lice (Phthiraptera), booklice and barklice (Psocoptera). The tree was constructed using Bayesian and maximum likelihood methods with concatenated sequences of the first and second codon positions of 11 mitochondrial (mt) protein-coding genes and two mt rRNA genes (6,850 nucleotides). Posterior probability and bootstrap support value (%) for each grouping were indicated near the branch nodes. Trees were rooted with the true bug, *Alloeorhynchus bakeri*. Drawing of insects was by Hu Li.

The elephant louse, *Haematomyzus elephantis*, together with the African wart-hog louse, *Haematomyzus hopkinsi*, and the African red-river hog louse, *Haematomyzus porci*, differ markedly from other parasitic lice in morphology and thus are in a suborder of their own, Rhyncophthirina^14^. We used maximum likelihood (ML) and Bayesian inference (BI) methods and generated eight phylogenetic trees in total with concatenated sequences of: 1) 11 mt protein-coding genes (Alignment S6); 2) the first and second codon positions of the 11 mt protein-coding genes (Alignment S7); 3) the 11 mt protein-coding genes and the two mt rRNA genes (Alignment S8); and 4) the first and second codon positions of the 11 mt protein-coding genes and the two mt rRNA genes (Alignment S9; also see Method below). In all of the eight trees, the elephant louse (suborder Rhyncophthirina) was placed as the sister group to the seven species of sucking lice (suborder Anoplura) (Fig. 5; Fig. S3). The monophyly of Anoplura and the sister-group relationship between Anoplura and Rhyncophthirina (represented by the elephant louse in the current study) were strongly supported in all of the trees with bootstrap support values (BSV) 97–100% and posterior probabilities (PP) 1. The suborder Ischnocera was paraphyletic: one of its species, *Bothriometopus macrocnemis* (screamer louse), was more closely related to Anoplura + Rhyncophthirina than to the other two ischnoceran species (BSV 62–88%, PP 0.88–1). The monophyly of the parasitic lice (order Phthiraptera) was supported in six of the eight trees (PCGRNA_ML, PCGRNA_BA, PCG12_ML, PCG12_BA, PCG12RNA_ML, PCG12RNA_BA; BSV 72–79%, PP 0.97–1). The monophyly of the barklice and booklice (order Psocoptera) was strongly rejected in all of the eight trees as the booklouse *Liposcelis bostrychophila* was more closely related to the parasitic lice than to the barklice (BSV 100%, PP 1). The sister-group relationship between Anoplura and Rhyncophthirina was also supported by four shared characters of mt gene arrangement: *L*_*1*_*-rrnL*, *L*_*2*_*-rrnS*, *T-nad1-Q* and *rrnL-V* (Table 3). These characters are derived for insects and present only in the elephant louse and the sucking lice, not in other parasitic lice, nor in other insects.

## Discussion

**Figure 6:**
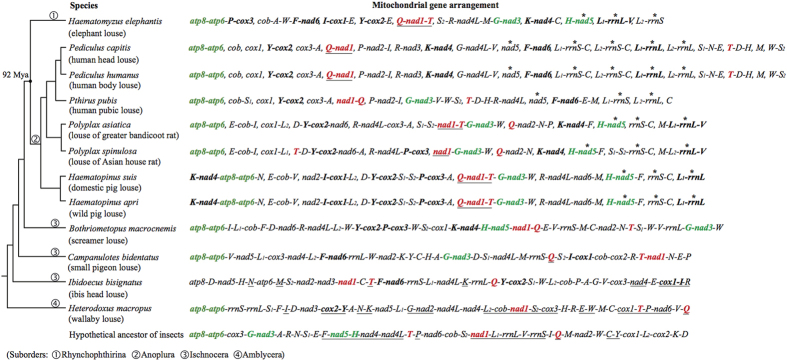
Comparison of mitochondrial gene arrangement between the elephant louse, *Haematomyzus elephantis*, and other parasitic lice. The phylogenetic tree is consistent with that shown in Fig. 5. The black dot on the tree indicates the most recent common ancestor (MRCA) shared by species of the two suborders, Rhynchophthirina and Anoplura. *nad5*, *rrnS* and *rrnL* genes of the elephant louse and sucking lice are highlighted with asterisks; each of these genes has its own minichromosomes, not shared with other protein-coding genes or rRNA genes. *trnT*, *nad1* and *trnQ* genes are highlighted in bold red. The ancestral gene-arrangements of insects retained in the elephant louse are in bold green; the derived gene-arrangements of insects present in the elephant louse and shared by other parasitic lice are in bold black. Hyphens link neighboring genes on the same minichromosome; commas separate minichromosomes.

The sister-group relationship between the suborder Anoplura (sucking lice) and the suborder Rhynchophthirina (elephant louse, wart-hog louse and red-river hog louse) indicated above by mt genome sequence analyses and derived gene-arrangement characters is consistent with the phylogeny inferred previously by morphological and molecular analyses^15^,^16^,^17^,^18^,^19^,^20^. Furthermore, Light *et al.*^20^ and Smith *et al.*^21^ dated the most recent common ancestor (MRCA) of Anoplura and Rhynchophthirina to be ~92 million years old. The species from Anoplura and Rhynchophthirina investigated to date all have fragmented minichromosomes. Thus, the most plausible explanation is that fragmented mt genomes were already present in the MRCA of Anoplura and Rhynchophthirina, and all species of these two suborders retained this novel mt genome organization (Fig. 6). Cameron *et al.* reported three minicircles with six mt genes in a *Damalinia* species (Trichodectidae, Ischnocera)^6^, indicating that fragmented mt genomes may be present in parasitic lice outside Anoplura and Rhynchophthirina. The exact origin of fragmented mt genomes in parasitic lice remains to be determined with more and wider sampling of species from the suborders Ischnocera and Amblycera.

What did the fragmented mitochondrial genome of the MRCA of Anoplura and Rhynchophthirina look like? How different was it from those of the elephant louse and the sucking lice we observed today? For the convenience and accuracy of communication, we find it helpful to introduce the expression (and concept) of “mitochondrial karyotype” for the elephant louse and the sucking lice whose mt genomes contain multiple minichromosomes. Analogous to “nuclear karyotype”, by “mitochondrial karyotype” we mean: 1) the number of mt minichromosomes, 2) the topology of minichromosomes (i.e. linear or circular), and 3) the gene content and gene arrangement of each minichromosome. With the data available now, we cannot re-construct yet the ancestral mt karyotype of Anoplura and Rhyncophthirina. However, we can infer that several common features that the elephant louse shares with the sucking lice were likely present in the ancestral mt karyotype of Anoplura and Rhyncophthirina (Fig. 6). These features include: 1) the two rRNA genes, *rrnS* and *rrnL*, and *nad5* gene were on their own minichromosomes, likely with tRNA genes downstream and/or upstream but not with any other protein-coding or rRNA genes; 2) three genes, *trnT*, *nad1* and *trnQ*, were in a cluster and had the opposite orientation of transcription relative to that of other mt genes, with subsequent rearrangement of these genes in *Pediculus* species, *Pthirus pubis* and *Polyplax* species; 3) three ancestral gene-arrangement characters of insects were retained: *atp8-atp6*, *trnG-nad3* and *trnH-nad5*; and 4) six derived gene-arrangement characters to insects were present: *trnP-cox3*, *trnF-nad6*, *trnI-cox1*, *trnY-cox2*, *trnK-nad4* and *trnL*_*1*_-*rrnL-trnV*.

The number of mt minichromosomes known varies from 9 in the pig lice, *Haemapinus suis* and *Haemapinus apri*, and the horse louse, *Haemapinus asini*, to 20 in the human head louse, *Pe. h. capitis*, and the human body louse, *Pe. h. humanus*^8^,^9^,^11^,^12^. The elephant louse, *Haematomyzus elephantis*, has 10 minichromosomes (with 4 mt genes not identified yet). The rat lice, *Po. asiatica* and *Po. spinulosa*, have 11 minichromosomes^10^ and the human pubic louse, *Pt. pubis*, has 14 minichromosomes^9^ (3 genes not identified; Fig. 6). The substantial variation in the number of mt minichromosomes among the elephant louse and the sucking lice indicates that the process of mt genome fragmentation is likely a continuous process. One possibility is that the MRCA of Anoplura and Rhyncophthirina had less minichromosomes than what we have seen today in the species investigated from these two suborders. Fragmentation since the MRCA may have occurred at different rates in different lineages of the Anoplura and Rhyncophthirina and thus generated the mt minichromosome diversity we see today. We speculate that lineage-specific factors may have a role in generating the variation in the extent and the rate of mt genome fragmentation. Dong *et al.* noticed a casual, inverse link between the length of life cycle of the sucking lice and the extent of mitochondrial genome fragmentation^10^. Among the sucking lice investigated to date, the pig lice have the longest life cycle (29–48 days depending on weather) and the least fragmented mt genome. In contrast, the human head lice and the body lice have the shortest life cycle (13–20 days) and the most fragmented mt genomes. The rat lice, *Po. spinulosa*, have both the length of life cycle (25–28 days) and the extent of mt genome fragmentation in between the pig lice and the human head and body lice. Furthermore, the human pubic lice have both the length of life cycle (16–25 days) and the extent of mt genome fragmentation in between the rat lice and the human head and body lice. This link can be tested further with data from more sucking lice and other eukaryotes that have fragmented mt genomes^10^. In theory, if mt genome fragmentation was a continuous process and each life cycle contributed equally to the fragmentation, then species with shorter life cycles would be expected to have more fragmented mt genomes than species with longer life cycles.

Despite the common gene-arrangement features above, nine of the 10 mt minichromosomes of the elephant louse differ from those of the sucking lice known in gene content and gene arrangement; only *trnH-nad5* minichromosome is also seen in *Po. asiatica*, the louse of greater bandicoot rat (Fig. 6). Apparently, distinct mt karyotypes have evolved between Anoplura and Rhynchophthirina since they diverged. Distinct mt karyotypes have evolved within the sucking lice too. The human lice, pig lice, the horse louse and rat lice all have their unique mt karyotypes not seen in other lice^8^,^9^,^10^,^11^,^12^. Animal mt genomes are usually extremely stable in terms of gene content, gene arrangement and genome organization^23^,^25^. The mt genomes of the elephant louse and the sucking lice apparently evolved at a much higher rate than in other insects and animals. What caused the fragmentation of mt genomes in the sucking lice and the elephant louse? Why are the mt karyotypes of these lice so variable? We cannot address these questions yet with the data available now. Parasitic lice do display features not usually seen in other insects or animals. For instance, a recent study showed that DNA substitution rate in the parasitic lice of humans and chimpanzees was on average 14 times higher than in their hosts^26^. We expect further studies on parasitic lice and related insects will be able to reveal the evolutionary forces and/or the cellular or biochemical mechanisms that drove the fragmentation of mt genomes in these insects.

## Methods

### Sample collection, DNA extraction and amplification of mitochondrial genome

Specimens of the elephant louse, *Haematomyzus elephantis*, were collected from elephants in Chitwan National Park, Nepal. Specimens were stored in 100% ethanol at −20 °C. Total DNA was extracted from individual specimens with DNeasy Blood & Tissue kit (QIAGEN).

Fragments of mt *cox1*, *cox2*, *rrnS* and *rrnL* genes were amplified by PCR with primer pairs mtd6–mtd11 (659 bp), mtd16–mtd18 (299 bp), 12SA–12SB (487 bp) and mtd32m–mtd34 (544 bp), respectively (Table S6). These four pairs of primers target conserved sequence motifs in the mt genome of the elephant louse. The amplicons of these fragments were sequenced with AB3730*xl* 96-capillary sequencers at the AGRF (Australian Genome Research Facilities). Four pairs of elephant-louse-specific primers, ELC1F–ELC1R, ELC2F–ELC2R1, EL12SF1–EL12SR and EL16SF–LX16SR, were designed from *cox1*, *cox2*, *rrnS* and *rrnL* genes; the forward and reverse primer in each pair are close to each other with 78, 7, 2 and 8 bp in between respectively (Table S6). PCRs with these four pairs of elephant-louse-specific primers amplified in near full-length the four minichromosomes that contain *cox1*, *cox2*, *rrnS* and *rrnL* genes respectively (Fig. 1C). The amplicons from these four minichromosomes were cloned into JM109 Competent Cells with pGEM-T Easy Vector System (Promega) and sequenced with AB3730*xl* 96-capillary sequencers at the AGRF using a primer-walking strategy (Table S6). Sequences of the non-coding regions of these four minichromosomes near the 5′ end and the 3′ end of the coding regions were obtained and aligned with ClustalX^27^; a forward primer USFB1567 and a reverse primer ELR were designed from the conserved sequence motives (Table S6). The PCR with USFB1567–ELR produced a mixture of amplicons, 1.2–2.0 kb in size (Fig. 1C), expected from the coding regions of all mt minichromosomes of the elephant louse; these amplicons were sequenced with next-generation platforms (see below). The PCR amplification strategy used in this study was developed from our observations in previous studies that each mt minichromosome of a sucking louse species has a distinct coding region and a well-conserved non-coding region^13^.

Elongase Enzyme (Life Technologies) was used in short PCRs to amplify fragments of *cox1*, *cox2*, *rrnS* and *rrnL* genes; TaKaRa Ex and LA *Taq* were used subsequently in long PCR amplifications, following the manufacturers’ instructions. PCR cycling conditions were: 94 °C for 1 min, 35 cycles of 98 °C for 10 sec, 50–65 °C (depending on primers) for 30 sec, and 72 °C for 1–4 min (depending on target size, ~1 min/kb), followed by 72 °C for 2–8 min. Negative controls were executed with each PCR experiment to detect DNA contamination and false positive amplicons. PCR amplicons were checked with agarose-gel electrophoresis. The sizes of PCR amplicons were estimated by comparison with molecular markers. PCR amplicons used for cloning and sequencing were purified with Wizard SV Gel/PCR Clean-up System (Promega).

### Next-generation sequencing, assembly and verification of mitochondrial minichromosomes

Purified PCR amplicons generated above with the primer pair USFB1567–ELR from the coding regions of mt minichromosomes of the elephant louse were sequenced with Roche GS FLX (454) platform at the AGRF. We chose this platform because of its long reads relative to other platforms, which was an advantage for *de novo* assembly. Sequence-reads were assembled *de novo* into contigs with Geneious 6.1.2^28^. The parameters for sequence assembly were: 1) minimum overlap 100 bp; 2) minimum overlap identity 90%; 3) maximum gaps per read 10%; and 4) maximum gap size 10 bp. tRNA genes were identified using tRNAscan-SE and ARWEN based on their secondary structures (Fig. S4)^29^,^30^. Protein-coding genes and rRNA genes were identified by BLAST searches of NCBI database^31^. Identical sequences shared between mt genes were identified with Wordmatch^32^. BLAST searches did not find significant matches to *nad6* gene; this gene was identified by comparison of the hydrophilicity profile of its putative protein NAD6 and the conserved amino acid sequences of NAD6 with those of other animals (Figs S5, 6; Alignment S10).

Outbound primers were designed from the coding regions of each mt minichromosome to verify the size and the circular organization of the minichromosomes revealed above by sequence assembly (Table S1). The two primers in each pair are either immediately next to each other or are 2–35 bp apart; these primers amplify each minichromosome in full or near-full length if that minichromosome has a circular organization. PCR set-up and cycling conditions were the same as described above.

To obtain full-length non-coding region sequences, we sequenced, in a separate batch, the amplicons from four full-length mt minichromosomes of the elephant louse with Illumina Hiseq 2000 platform at the BGI (Beijing Genomics Institute). We chose this platform because of its high coverage and accuracy relative to other platforms. Illumina sequence-reads were assembled *de novo* with Geneious 7.0.5^28^; the assembly parameters were: 1) minimum overlap 70 bp; 2) minimum overlap identity 90%; 3) maximum gaps per read 5%; and 4) maximum gap size 5 bp. We also sequenced the non-coding regions of the other six minichromosomes except for the GC-rich parts and the tandem-repeat parts using a Sanger sequencing platform. The Sanger sequence-reads were assembled using the full-length NCR sequences generated above as references with the parameters: 1) minimum overlap 200 bp; 2) minimum overlap identity 95%; 3) maximum gaps per read 5%; and 4) maximum gap size 5 bp. Heteroplasmic sites in NCRs were called with the “Find Variations/ SNPs” function in Geneious 7.0.5 with the parameters “Minimum Coverage” 50, and “Minimum Variant Frequency” 10%.

### Phylogenetic analyses of mitochondrial genome sequences and tRNA-leucine gene sequences

We inferred the phylogenetic relationship of the elephant louse to 15 other parasitic lice (Phthiraptera), barklice and booklice (Psocoptera) with concatenated sequences of: 1) 11 mt protein-coding genes (*nad2* and *nad4* excluded; 8,040 nucleotides, PCG hereafter); 2) the first and second codon positions of the 11 mt protein-coding genes (5,360 nucleotides, PCG12 hereafter); 3) the 11 mt protein-coding genes and the two mt rRNA genes (9,530 nucleotides, PCGRNA hereafter); and 4) the first and second codon positions of the 11 mt protein-coding genes and the two mt rRNA genes (6,850 nucleotides, PCG12RNA hereafter) (Table S7)^38^,^39^,^40^,^41^. Sequences of mt protein-coding and rRNA genes were aligned individually with MUSCLE (gap open: -2.9; gap extend; 0; hydrophobicity multiplier: 1.2; clustering methods: UPGMB; diagonals min length: 24)^33^. *nad2* and *nad4* were excluded because *nad2* was not identified in the elephant louse whereas *nad4* was not identified in the human pubic louse^9^. Segments of identical sequences (26–127 bp long) shared between different mt genes in the sucking lice^8^,^9^,^11^ were also excluded to ensure only homologous regions of the mt genes were aligned and used in subsequent analyses. Putative amino acid sequences were used to guide nucleotide sequence alignments for the protein-coding genes. Alignments of individual genes were concatenated after removing poorly aligned sites using Gblocks 0.91 (minimum number of sequences for a conserved position: 11; minimum number of sequences for a flanking position: 11; maximum number of contiguous nonconserved positions: 8; minimum length of an initial block: 5; minimum length of a block: 5; allowed gap positions: with half)^34^. The concatenated alignment was used in maximum likelihood (ML) and Bayesian analyses with RAxML 7.0.3 and MrBayes 3.2.1^35^,^36^. Bootstrap values for node support in ML trees were obtained by heuristic searches of 1,000 resampled datasets. For Bayesian analyses, two simultaneous runs of 10 million generations were conducted; trees were sampled every 1,000 generations with the first 25% discarded as burn-in. Stationarity was considered to be reached when the average standard deviation of split frequencies was below 0.01.

We analyzed the sequences of the two mt tRNA genes for leucine, *trnL*_*1*_*(tag)* and *trnL*_*2*_*(taa)*, with distanced-based neighbor-joining (NJ) method to compare the evolution patterns of these two genes in the elephant louse, the sucking lice and other insects and mammals. The sequences of *trnL*_*1*_(tag) and *trnL*_*2*_(taa) were retrieved from GenBank for sucking lice and other insects and mammals, and were aligned according to their secondary structures. NJ trees were constructed with MEGA 5.2.2^37^. P-distance model was used and 5,000 replicates of bootstrap resampling were run.

## Additional Information

**Accession codes:** The nucleotide sequence of the mt genome of the elephant louse reported in this manuscript has been deposited in GenBank under accession numbers KF933032-41. 

**How to cite this article**: Shao, R. *et al.* Fragmented mitochondrial genomes in two suborders of parasitic lice of eutherian mammals (Anoplura and Rhynchophthirina, Insecta). *Sci. Rep.* **5**, 17389; doi: 10.1038/srep17389 (2015).

## Supplementary Material

Supplementary Information

Supplementary Dataset 1

Supplementary Dataset 2

Supplementary Dataset 3

Supplementary Dataset 4

Supplementary Dataset 5

Supplementary Dataset 6

Supplementary Dataset 7

Supplementary Dataset 8

Supplementary Dataset 9

## Figures and Tables

**Table 1:** The mitochondrial minichromosomes of the elephant louse, *Haematomyzus elephantis*, identified by Roche GS FLX (454) sequencing.

Gene content and gene arrangement in each minichromosome	Size of coding region (bp)	Number of Roche sequence reads	Coverage (fold)	
			Range	Mean
atp8-atp6-trnP-cox3*	1754	410	14–109	74
*cob-trnA-trnW-trnF-nad6*	1879	215	6–70	38
*trnI-cox1-trnE*	1707	1157	77–340	208
*trnY-cox2-trnE*	857	678	23–299	127
*trnT-nad1-trnQ*	1143	4041	308–1701	983
*trnS*_*2*_*(tga)-trnR-nad4L-trnM-trnG-nad3*	968	1384	102–558	336
*trnK-nad4-trnC*	1528	38	1–20	8
*trnH-nad5*	1795	455	28–197	71
*trnL*_*2*_*(taa)-rrnS*	961	5125	352–2165	1310
*trnL*_*1*_*(tag)-rrnL-trnV*	1436	1257	50–497	263

^*^See Fig. 1 legend for the full names of mt genes.

**Table 2:** Illumina sequencing of PCR amplicons of four near full-length mitochondrial minichromosomes of the elephant louse, *Haematomyzus elephantis.*

Target minichromosome	Primer pair used^*#*^	Size of amplicon (bp)	Number of Illumina sequence reads	Coverage (fold)	
				Range	Mean
trnI-cox1-trnE*	YSC1F(2)-YSC1R(2)	4026	114037	807–8319	2558
*trnT-nad1-trnQ*	YSN1F-YSN1R	3600	176234	828–16628	4388
*trnK-nad4-trnC*	YSN4F-YSN4R	3875	278107	1068–19823	6462
*trnH-nad5*	YSN5F(2)-YSN5R(2)	4153	158711	1258–9427	3453
*trnH-nad5*	YSHF-YSHR	4150	410271	2487–25650	8921

^#^See Table S1 for primer sequences.

^*^See Fig. 1 legend for the full names of mt genes.

**Table 3:** The four derived characters of mitochondrial gene arrangement shared exclusively by the elephant louse and the sucking lice.

Species of insects	Order/suborder	*L*_*1*_*-rrnL*	*L*_*2*_*-rrnS*	*T-nad1-Q*	*rrnL-V*
*Haematomyzus elephantis (elephant louse)*	Phthiraptera/Rhynchophthirina	+	+	+	+
			
*Pediculus humanus capitis (human head louse)*	Phthiraptera/Anoplura	+	+	—	—
			
*Pediculus humanus humanus (human body louse)*	Phthiraptera/Anoplura	+	+	—	—
			
*Pthirus pubis (human pubic louse)*	Phthiraptera/Anoplura	—	+	—	—
			
*Polyplax asiatica (rat louse)*	Phthiraptera/Anoplura	+	—	—	+
			
*Polyplax spinulosa* (rat louse)	Phthiraptera/Anoplura	—	—	—	+
*Haematopinus suis (domestic pig louse)*	Phthiraptera/Anoplura	+	—	+	—
			
*Haemapinus apri (wild pig louse)*	Phthiraptera/Anoplura	+	—	+	—
			

Note: “+” is for “presence”; “-” is for “absence”.
