# Supplementary material for: Fragmented mitochondrial genomes in two suborders of parasitic lice of eutherian mammals (Anoplura and Rhynchophthirina, Insecta)
Source: Sci Rep. 2015 Nov 30;5:17389. doi: 10.1038/srep17389 (PMC4663631; doi:10.1038/srep17389)
Supplement: Supplementary Information [file srep17389-s1.pdf]

Supplementary information: Table S1-S7 and Figure S1-S6

Fragmented mitochondrial genomes in two suborders of parasitic lice of eutherian mammals (Anoplura and Rhynchophthirina, Insecta)

Renfu Shao, Stephen C Barker, Hu Li, Simon Song, Shreekanta Poudel, Yuan Su

Table S1. PCR primers used to verify the mitochondrial minichromosomes of the elephant louse, *Haematomyzus elephantis*

Primer	Target gene or region	Sequence (5'-3')	Use
YSC3F	*cox3*	TACTTGCACTTGCGCA GCTTTGTGGTGACG	Verifying *atp8-atp6-trnP-cox3* minichromosome with YSC3R
YSC3R	*cox3*	GCGATCCACCTAGTTA CAGCCCCTATGAAG	Verifying *atp8-atp6-trnP-cox3* minichromosome with YSC3F
YSCBF	*cob*	GTAGAAGAAATCCAT TAGGGTTGTCGAGGG	Verifying *cob-trnA-trnW-trnF-nad6* minichromosome with YSCBR
YSCBR	*cob*	GGGCCGCAATGTGCG TAATAGCCAAGCTTA	Verifying *cob-trnA-trnW-trnF-nad6* minichromosome with YSCBF
YSC1F(2)	*cox1*	GTTATTGCTATTCCTA CTGGAGTAAAAGTG	Verifying *trnI-cox1-trnE* minichromosome with YSC1R(2)
YSC1R(2)	*cox1*	GTAGCACTTGTAAAGT AAGCACGACTGTCA	Verifying *trnI-cox1-trnE* minichromosome with YSC1F(2)
YSC2F(2)	*cox2*	TGAGATGTTCGCCTTT TAAGAGTTGATCAG	Verifying *trnY-cox2-trnE* minichromosome with YSC2R(2)
YSC2R(2)	*cox2*	CCCTTATTATGTAAGA ATCGAAGGAAACTC	Verifying *trnY-cox2-trnE* minichromosome with YSC2F(2)
YSN1F	*nad1*	GGGAAAGTGAACTAG TAGCAGGTTACACAG	Verifying *trnT-nad1-trnQ* minichromosome with YSN1R
YSN1R	*nad1*	CTTCTGAAAGGTCAA AAGGTCTCCGACCCC	Verifying *trnT-nad1-trnQ* minichromosome with YSN1F
YSN3F	*nad3*	GGTCAAATGAGCCTTT TGAATGTGGAATGG	Verifying *$trnS_2$(tga)-trnR-nad4L-trnM-trnG-nad3* minichromosome with YSN3R
YSN3R	*nad3*	GCTTTAATTCGGACCC TGAGAATAAACCTC	Verifying *$trnS_2$(tga)-trnR-nad4L-trnM-trnG-nad3* minichromosome with YSN3F
YSN4F	*nad4*	CCCATTAGAGGGGAG TATCCTCTTAGCCGC	Verifying *trnK-nad4-trnC* minichromosome with YSN4R
YSN4R	*nad4*	GCTTCAACATGTGCTT TAGGGAGCCACGAA	Verifying *trnK-nad4-trnC* minichromosome with YSN4F
YSN5F(2)	*nad5*	ATTTGACCTTGCCTTG TTTCATATAATGGC	Verifying *trnH-nad5* minichromosome with YSN5R(2)
YSN5R(2)	*nad5*	TTGAAGGGCAACAGA GTTAATCATTACTCC	Verifying *trnH-nad5* minichromosome with YSN5F(2)
YSHF	*trnH*	TGGAGTCATAAATGG GCTCTCCCCCCTTCC	Verifying *trnH-nad5* minichromosome with YSHR
YSHR	*trnH*	GTCACAGTTTTACAAT TAAAACTACCCTTC	Verifying *trnH-nad5* minichromosome with YSHF
YSrSF	*rrnS*	CAGGTCAAGGTGCAG	Verifying *$trnL_2$(taa)-rrnS*

		GTTATATTTATGGGG	minichromosome with YSrSR
YSrSR	*rrnS*	CAGCCACGTATTCCAC<br>ATATGAGTGCTGAT	Verifying *trnL$_2$(taa)-rrnS*<br>minichromosome with YSrSF
YSrLF	*rrnL*	GTTGAACTATTAGACC<br>CTCCACAGCGAGGG	Verifying *trnL$_1$(tag)-rrnL-trnV*<br>minichromosome with YSrLR
YSrLR	*rrnL*	CCGCCCCAGACAAAA<br>TTACTTTTCATCTCT	Verifying *trnL$_1$(tag)-rrnL-trnV*<br>minichromosome with YSrLF

Table S2. Polymorphic sites in the non-coding region of *I-cox1-E* minichromosome

Name	Type	Minimum	Maximum	Length	Coverage	Polymorphism Type	Variant Frequency
A	Polymorphism	103	103	1	2,563	SNP	43.10%
T	Polymorphism	103	103	1	2,563	SNP	56.90%
A	Polymorphism	148	148	1	1,452	SNP	63.00%
C	Polymorphism	148	148	1	1,452	SNP	36.80%
C	Polymorphism	152	152	1	1,387	SNP	62.90%
T	Polymorphism	152	152	1	1,387	SNP	37.10%
C	Polymorphism	186	186	1	1,012	SNP	40.60%
T	Polymorphism	186	186	1	1,012	SNP	59.40%
A	Polymorphism	304	304	1	1,515	SNP	42.40%
T	Polymorphism	304	304	1	1,515	SNP	52.70%
A	Polymorphism	305	305	1	1,531	SNP	57.90%
G	Polymorphism	305	305	1	1,531	SNP	40.80%
A	Polymorphism	307	307	1	1,540	SNP	40.50%
G	Polymorphism	307	307	1	1,540	SNP	59.50%
A	Polymorphism	308	308	1	1,555	SNP	58.80%
G	Polymorphism	308	308	1	1,555	SNP	41.20%
A	Polymorphism	311	311	1	1,586	SNP	61.90%
G	Polymorphism	311	311	1	1,586	SNP	34.20%
A	Polymorphism	431	431	1	1,968	SNP	39.30%
T	Polymorphism	431	431	1	1,968	SNP	60.50%
A	Polymorphism	447	447	1	1,931	SNP	80.30%
G	Polymorphism	447	447	1	1,931	SNP	18.50%
A	Polymorphism	448	448	1	1,926	SNP	18.20%
G	Polymorphism	448	448	1	1,926	SNP	81.80%
A	Polymorphism	452	452	1	1,914	SNP	18.20%
G	Polymorphism	452	452	1	1,914	SNP	81.80%
C	Polymorphism	457	457	1	1,882	Indel	78.60%
	Polymorphism	457	457	1	1,882	Indel	19.60%
C	Polymorphism	460	460	1	1,876	SNP	78.10%
T	Polymorphism	460	460	1	1,876	SNP	21.90%
A	Polymorphism	469	469	1	1,822	Indel	85.90%
	Polymorphism	469	469	1	1,822	Indel	12.00%
T	Polymorphism	470	470	1	1,816	Indel	86.30%
	Polymorphism	470	470	1	1,816	Indel	12.00%
T	Polymorphism	471	471	1	1,791	Indel	87.50%
	Polymorphism	471	471	1	1,791	Indel	12.20%
A	Polymorphism	473	473	1	1,803	SNP	12.30%
C	Polymorphism	473	473	1	1,803	SNP	87.00%
C	Polymorphism	503	503	1	1,704	SNP	45.60%
T	Polymorphism	503	503	1	1,704	SNP	54.40%
A	Polymorphism	505	505	1	1,703	SNP	39.00%
T	Polymorphism	505	505	1	1,703	SNP	61.00%
C	Polymorphism	507	507	1	1,711	SNP	60.60%
T	Polymorphism	507	507	1	1,711	SNP	39.30%

A	Polymorphism	572	572	1	1,402	SNP	46.40%
T	Polymorphism	572	572	1	1,402	SNP	53.40%
C	Polymorphism	632	632	1	2,054	SNP	43.30%
T	Polymorphism	632	632	1	2,054	SNP	56.70%
C	Polymorphism	838	838	1	3,379	SNP	19.70%
T	Polymorphism	838	838	1	3,379	SNP	80.30%
C	Polymorphism	842	842	1	3,347	SNP	20.10%
T	Polymorphism	842	842	1	3,347	SNP	79.80%
C	Polymorphism	849	849	1	3,348	SNP	15.30%
T	Polymorphism	849	849	1	3,348	SNP	84.60%
G	Polymorphism	853	853	1	3,341	SNP	16.10%
T	Polymorphism	853	853	1	3,341	SNP	83.70%
C	Polymorphism	1,650	1,650	1	3,058	SNP	71.50%
T	Polymorphism	1,650	1,650	1	3,058	SNP	27.80%
C	Polymorphism	1,652	1,652	1	3,074	SNP	29.00%
T	Polymorphism	1,652	1,652	1	3,074	SNP	70.10%
A	Polymorphism	1,653	1,653	1	3,073	SNP	28.90%
C	Polymorphism	1,653	1,653	1	3,073	SNP	27.50%
T	Polymorphism	1,653	1,653	1	3,073	SNP	43.50%
C	Polymorphism	1,654	1,654	1	3,083	SNP	88.00%
T	Polymorphism	1,654	1,654	1	3,083	SNP	12.00%
C	Polymorphism	1,655	1,655	1	3,108	SNP	72.40%
T	Polymorphism	1,655	1,655	1	3,108	SNP	27.60%
A	Polymorphism	1,656	1,656	1	3,109	SNP	83.70%
T	Polymorphism	1,656	1,656	1	3,109	SNP	15.90%
C	Polymorphism	1,660	1,660	1	3,128	SNP	36.10%
T	Polymorphism	1,660	1,660	1	3,128	SNP	63.90%
C	Polymorphism	1,661	1,661	1	3,132	SNP	83.60%
T	Polymorphism	1,661	1,661	1	3,132	SNP	16.30%
G	Polymorphism	1,665	1,665	1	3,151	SNP	83.70%
T	Polymorphism	1,665	1,665	1	3,151	SNP	16.10%
C	Polymorphism	1,721	1,721	1	3,149	SNP	55.70%
T	Polymorphism	1,721	1,721	1	3,149	SNP	44.30%
A	Polymorphism	1,736	1,736	1	3,048	SNP	80.90%
G	Polymorphism	1,736	1,736	1	3,048	SNP	19.10%
A	Polymorphism	1,856	1,856	1	3,278	SNP	45.60%
C	Polymorphism	1,856	1,856	1	3,278	SNP	54.30%
C	Polymorphism	1,877	1,877	1	3,154	SNP	14.50%
T	Polymorphism	1,877	1,877	1	3,154	SNP	85.50%
C	Polymorphism	2,100	2,100	1	2,919	SNP	71.20%
T	Polymorphism	2,100	2,100	1	2,919	SNP	28.70%
C	Polymorphism	2,105	2,105	1	2,960	SNP	28.80%
T	Polymorphism	2,105	2,105	1	2,960	SNP	71.10%
C	Polymorphism	2,110	2,110	1	2,973	SNP	37.80%
T	Polymorphism	2,110	2,110	1	2,973	SNP	62.00%
A	Polymorphism	2,187	2,187	1	2,901	SNP	70.10%

G	Polymorphism	2,187	2,187	1	2,901	SNP	29.80%

Table S3. Heteroplasmic sites in the non-coding region of *T-nad1-Q* minichromosome

Name	Type	Minimum	Maximum	Length	Coverage	Polymorphism Type	Variant Frequency
A	Polymorphism	264	264	1	1,300	Indel	88.10%
	Polymorphism	264	264	1	1,300	Indel	10.20%
C	Polymorphism	265	265	1	1,257	Indel	89.20%
	Polymorphism	265	265	1	1,257	Indel	10.60%
G	Polymorphism	269	269	1	1,121	SNP	19.60%
T	Polymorphism	269	269	1	1,121	SNP	80.20%
C	Polymorphism	275	275	1	946	Indel	72.30%
	Polymorphism	275	275	1	946	Indel	25.80%
C	Polymorphism	279	279	1	893	SNP	60.20%
T	Polymorphism	279	279	1	893	SNP	39.80%
C	Polymorphism	280	280	1	880	SNP	57.70%
G	Polymorphism	280	280	1	880	SNP	41.70%
A	Polymorphism	281	281	1	862	SNP	12.30%
C	Polymorphism	281	281	1	862	SNP	43.40%
T	Polymorphism	281	281	1	862	SNP	43.70%
A	Polymorphism	282	282	1	855	SNP	46.40%
C	Polymorphism	282	282	1	855	SNP	14.20%
G	Polymorphism	282	282	1	855	SNP	38.70%
C	Polymorphism	283	283	1	850	SNP	64.60%
G	Polymorphism	283	283	1	850	SNP	34.80%
C	Polymorphism	284	284	1	859	Indel	49.70%
G	Polymorphism	284	284	1	859	Indel	39.10%
	Polymorphism	284	284	1	859	Indel	11.20%
A	Polymorphism	285	285	1	877	Indel	16.10%
C	Polymorphism	285	285	1	877	Indel	72.90%
	Polymorphism	285	285	1	877	Indel	10.90%
C	Polymorphism	286	286	1	867	Indel	76.10%
G	Polymorphism	286	286	1	867	Indel	12.80%
	Polymorphism	286	286	1	867	Indel	11.10%
A	Polymorphism	287	287	1	895	Indel	76.20%
	Polymorphism	287	287	1	895	Indel	10.70%
G	Polymorphism	287	287	1	895	Indel	10.50%
A	Polymorphism	289	289	1	916	SNP	64.30%
G	Polymorphism	289	289	1	916	SNP	11.40%
T	Polymorphism	289	289	1	916	SNP	24.30%
G	Polymorphism	293	293	1	1,046	Indel	78.70%
	Polymorphism	293	293	1	1,046	Indel	21.30%
A	Polymorphism	345	345	1	2,481	SNP	33.50%
G	Polymorphism	345	345	1	2,481	SNP	66.40%
A	Polymorphism	349	349	1	2,537	SNP	67.30%
G	Polymorphism	349	349	1	2,537	SNP	32.60%
G	Polymorphism	364	364	1	2,701	Indel	83.20%

	Polymorphism	364	364	1	2,701	Indel	16.20%
G	Polymorphism	376	376	1	2,491	SNP	13.70%
T	Polymorphism	376	376	1	2,491	SNP	85.90%
C	Polymorphism	379	379	1	2,375	SNP	22.90%
T	Polymorphism	379	379	1	2,375	SNP	77.00%
A	Polymorphism	380	380	1	2,342	SNP	77.80%
T	Polymorphism	380	380	1	2,342	SNP	22.00%
G	Polymorphism	386	386	1	2,093	SNP	87.80%
T	Polymorphism	386	386	1	2,093	SNP	12.10%
A	Polymorphism	389	389	1	1,948	SNP	10.50%
C	Polymorphism	389	389	1	1,948	SNP	89.50%
A	Polymorphism	419	419	1	1,747	SNP	84.30%
G	Polymorphism	419	419	1	1,747	SNP	15.70%
G	Polymorphism	424	424	1	1,872	Indel	84.30%
	Polymorphism	424	424	1	1,872	Indel	15.70%
T	Polymorphism	425	425	1	1,894	Indel	78.70%
	Polymorphism	425	425	1	1,894	Indel	15.10%
A	Polymorphism	468	468	1	2,372	SNP	39.10%
T	Polymorphism	468	468	1	2,372	SNP	60.60%
A	Polymorphism	484	484	1	2,583	SNP	74.50%
G	Polymorphism	484	484	1	2,583	SNP	25.10%
A	Polymorphism	485	485	1	2,600	SNP	24.50%
G	Polymorphism	485	485	1	2,600	SNP	75.50%
A	Polymorphism	489	489	1	2,640	SNP	24.10%
G	Polymorphism	489	489	1	2,640	SNP	75.90%
C	Polymorphism	494	494	1	2,734	Indel	69.20%
	Polymorphism	494	494	1	2,734	Indel	29.00%
C	Polymorphism	497	497	1	2,752	SNP	68.10%
T	Polymorphism	497	497	1	2,752	SNP	31.90%
A	Polymorphism	506	506	1	2,891	Indel	83.80%
	Polymorphism	506	506	1	2,891	Indel	14.40%
T	Polymorphism	507	507	1	2,905	Indel	84.40%
	Polymorphism	507	507	1	2,905	Indel	14.30%
T	Polymorphism	508	508	1	2,934	Indel	85.50%
	Polymorphism	508	508	1	2,934	Indel	14.10%
A	Polymorphism	510	510	1	2,961	SNP	12.30%
C	Polymorphism	510	510	1	2,961	SNP	87.00%
C	Polymorphism	540	540	1	3,160	SNP	69.60%
T	Polymorphism	540	540	1	3,160	SNP	30.30%
A	Polymorphism	542	542	1	3,165	SNP	60.40%
T	Polymorphism	542	542	1	3,165	SNP	39.50%
C	Polymorphism	544	544	1	3,182	SNP	39.60%
T	Polymorphism	544	544	1	3,182	SNP	60.30%
A	Polymorphism	609	609	1	2,408	SNP	60.80%
T	Polymorphism	609	609	1	2,408	SNP	39.00%
C	Polymorphism	669	669	1	2,795	SNP	59.80%

T	Polymorphism	669	669	1	2,795	SNP	40.20%
C	Polymorphism	875	875	1	3,655	SNP	21.00%
T	Polymorphism	875	875	1	3,655	SNP	79.00%
C	Polymorphism	877	877	1	3,689	SNP	24.50%
T	Polymorphism	877	877	1	3,689	SNP	75.40%
A	Polymorphism	878	878	1	3,705	SNP	24.10%
C	Polymorphism	878	878	1	3,705	SNP	73.50%
C	Polymorphism	879	879	1	3,707	SNP	42.50%
T	Polymorphism	879	879	1	3,707	SNP	57.40%
C	Polymorphism	880	880	1	3,748	SNP	27.50%
T	Polymorphism	880	880	1	3,748	SNP	72.30%
A	Polymorphism	881	881	1	3,772	SNP	74.90%
T	Polymorphism	881	881	1	3,772	SNP	24.50%
C	Polymorphism	885	885	1	3,911	SNP	73.40%
T	Polymorphism	885	885	1	3,911	SNP	26.60%
C	Polymorphism	886	886	1	3,956	SNP	29.40%
T	Polymorphism	886	886	1	3,956	SNP	70.50%
G	Polymorphism	890	890	1	4,039	SNP	34.60%
T	Polymorphism	890	890	1	4,039	SNP	65.30%
A	Polymorphism	919	919	1	4,883	SNP	10.40%
C	Polymorphism	919	919	1	4,883	SNP	88.60%
C	Polymorphism	1,337	1,337	1	2,509	SNP	31.70%
T	Polymorphism	1,337	1,337	1	2,509	SNP	67.30%
A	Polymorphism	1,338	1,338	1	2,550	Indel	25.30%
C	Polymorphism	1,338	1,338	1	2,550	Indel	57.70%
	Polymorphism	1,338	1,338	1	2,550	Indel	10.20%
C	Polymorphism	1,341	1,341	1	2,616	SNP	21.00%
T	Polymorphism	1,341	1,341	1	2,616	SNP	69.00%
	Polymorphism	1,341	1,340	0	2,565	Indel	89.40%
A	Polymorphism	1,341	1,340	0	2,565	Indel	10.60%
A	Polymorphism	1,342	1,342	1	2,642	SNP	78.40%
T	Polymorphism	1,342	1,342	1	2,642	SNP	18.10%
A	Polymorphism	1,344	1,344	1	2,727	Indel	70.30%
	Polymorphism	1,344	1,344	1	2,727	Indel	20.00%
G	Polymorphism	1,352	1,352	1	2,765	SNP	55.40%
T	Polymorphism	1,352	1,352	1	2,765	SNP	44.40%
C	Polymorphism	1,404	1,404	1	4,374	SNP	22.50%
T	Polymorphism	1,404	1,404	1	4,374	SNP	77.40%
C	Polymorphism	1,687	1,687	1	5,402	SNP	79.70%
T	Polymorphism	1,687	1,687	1	5,402	SNP	19.00%
C	Polymorphism	1,689	1,689	1	5,381	SNP	19.60%
T	Polymorphism	1,689	1,689	1	5,381	SNP	79.10%
A	Polymorphism	1,690	1,690	1	5,367	SNP	19.40%
C	Polymorphism	1,690	1,690	1	5,367	SNP	15.10%
T	Polymorphism	1,690	1,690	1	5,367	SNP	65.40%
C	Polymorphism	1,692	1,692	1	5,350	SNP	84.90%

T	Polymorphism	1,692	1,692	1	5,350	SNP	15.00%
A	Polymorphism	1,693	1,693	1	5,345	SNP	80.80%
T	Polymorphism	1,693	1,693	1	5,345	SNP	18.70%
C	Polymorphism	1,697	1,697	1	5,233	SNP	39.40%
T	Polymorphism	1,697	1,697	1	5,233	SNP	60.50%
C	Polymorphism	1,698	1,698	1	5,222	SNP	83.60%
T	Polymorphism	1,698	1,698	1	5,222	SNP	16.20%
G	Polymorphism	1,702	1,702	1	5,150	SNP	84.60%
T	Polymorphism	1,702	1,702	1	5,150	SNP	15.30%
T	Polymorphism	1,725	1,725	1	4,783	Indel	80.70%
	Polymorphism	1,725	1,725	1	4,783	Indel	16.30%
C	Polymorphism	1,727	1,727	1	4,791	SNP	82.20%
T	Polymorphism	1,727	1,727	1	4,791	SNP	17.80%
A	Polymorphism	1,732	1,732	1	4,787	SNP	77.70%
C	Polymorphism	1,732	1,732	1	4,787	SNP	18.90%
C	Polymorphism	1,758	1,758	1	4,613	SNP	40.70%
T	Polymorphism	1,758	1,758	1	4,613	SNP	59.20%
A	Polymorphism	1,773	1,773	1	4,541	SNP	68.80%
G	Polymorphism	1,773	1,773	1	4,541	SNP	31.20%
C	Polymorphism	1,914	1,914	1	4,274	SNP	42.00%
T	Polymorphism	1,914	1,914	1	4,274	SNP	58.00%
C	Polymorphism	2,137	2,137	1	4,048	SNP	44.00%
T	Polymorphism	2,137	2,137	1	4,048	SNP	55.90%
C	Polymorphism	2,142	2,142	1	4,045	SNP	56.30%
T	Polymorphism	2,142	2,142	1	4,045	SNP	43.70%
C	Polymorphism	2,147	2,147	1	4,077	SNP	63.80%
T	Polymorphism	2,147	2,147	1	4,077	SNP	36.20%
A	Polymorphism	2,224	2,224	1	4,411	SNP	78.20%
G	Polymorphism	2,224	2,224	1	4,411	SNP	21.70%
	Polymorphism	2,362	2,361	0	3,305	Indel	89.60%
G	Polymorphism	2,362	2,361	0	3,305	Indel	10.40%
	Polymorphism	2,362	2,361	0	3,305	Indel	89.60%
A	Polymorphism	2,362	2,361	0	3,305	Indel	10.40%

Table S4. Heteroplasmic sites in the non-coding region of *K-nad4-C* minichromosome

Name	Type	Minimum	Maximum	Length	Coverage	Polymorphism Type	Variant Frequency
A	Polymorphism	158	158	1	4,637	SNP	13.80%
C	Polymorphism	158	158	1	4,637	SNP	86.20%
C	Polymorphism	162	162	1	4,467	SNP	13.60%
T	Polymorphism	162	162	1	4,467	SNP	86.40%
C	Polymorphism	196	196	1	3,571	SNP	18.30%
T	Polymorphism	196	196	1	3,571	SNP	81.70%
A	Polymorphism	200	200	1	3,476	SNP	84.10%
G	Polymorphism	200	200	1	3,476	SNP	15.90%
A	Polymorphism	202	202	1	3,458	SNP	27.40%
T	Polymorphism	202	202	1	3,458	SNP	72.40%

A	Polymorphism	203	203	1	3,447	SNP	27.30%
T	Polymorphism	203	203	1	3,447	SNP	72.60%
C	Polymorphism	246	246	1	3,221	SNP	84.80%
T	Polymorphism	246	246	1	3,221	SNP	15.20%
A	Polymorphism	331	331	1	3,479	SNP	82.70%
G	Polymorphism	331	331	1	3,479	SNP	17.10%
A	Polymorphism	335	335	1	3,597	SNP	25.50%
G	Polymorphism	335	335	1	3,597	SNP	74.50%
G	Polymorphism	350	350	1	3,676	Indel	85.90%
	Polymorphism	350	350	1	3,676	Indel	13.20%
C	Polymorphism	365	365	1	3,344	SNP	31.30%
T	Polymorphism	365	365	1	3,344	SNP	68.60%
A	Polymorphism	366	366	1	3,325	SNP	68.80%
T	Polymorphism	366	366	1	3,325	SNP	31.00%
A	Polymorphism	454	454	1	4,281	SNP	75.40%
T	Polymorphism	454	454	1	4,281	SNP	24.40%
A	Polymorphism	470	470	1	4,429	SNP	74.10%
G	Polymorphism	470	470	1	4,429	SNP	25.40%
A	Polymorphism	471	471	1	4,433	SNP	25.30%
G	Polymorphism	471	471	1	4,433	SNP	74.70%
A	Polymorphism	475	475	1	4,456	SNP	24.80%
G	Polymorphism	475	475	1	4,456	SNP	75.20%
C	Polymorphism	480	480	1	4,473	Indel	63.60%
	Polymorphism	480	480	1	4,473	Indel	33.50%
C	Polymorphism	483	483	1	4,415	SNP	61.70%
T	Polymorphism	483	483	1	4,415	SNP	38.20%
A	Polymorphism	492	492	1	4,442	Indel	80.30%
	Polymorphism	492	492	1	4,442	Indel	17.90%
T	Polymorphism	493	493	1	4,453	Indel	80.70%
	Polymorphism	493	493	1	4,453	Indel	17.90%
T	Polymorphism	494	494	1	4,482	Indel	81.90%
	Polymorphism	494	494	1	4,482	Indel	17.70%
C	Polymorphism	526	526	1	4,318	SNP	64.30%
T	Polymorphism	526	526	1	4,318	SNP	35.70%
A	Polymorphism	528	528	1	4,330	SNP	55.90%
T	Polymorphism	528	528	1	4,330	SNP	44.00%
C	Polymorphism	530	530	1	4,332	SNP	44.00%
T	Polymorphism	530	530	1	4,332	SNP	55.90%
A	Polymorphism	595	595	1	3,509	SNP	39.30%
T	Polymorphism	595	595	1	3,509	SNP	60.60%
C	Polymorphism	655	655	1	5,113	SNP	80.20%
T	Polymorphism	655	655	1	5,113	SNP	19.80%
C	Polymorphism	861	861	1	7,796	SNP	18.20%
T	Polymorphism	861	861	1	7,796	SNP	81.70%
C	Polymorphism	863	863	1	7,800	SNP	13.30%
T	Polymorphism	863	863	1	7,800	SNP	86.70%

A	Polymorphism	864	864	1	7,771	SNP	12.60%
C	Polymorphism	864	864	1	7,771	SNP	81.10%
C	Polymorphism	865	865	1	7,764	SNP	27.10%
T	Polymorphism	865	865	1	7,764	SNP	72.90%
C	Polymorphism	866	866	1	7,785	SNP	19.30%
T	Polymorphism	866	866	1	7,785	SNP	80.60%
A	Polymorphism	867	867	1	7,809	SNP	86.80%
T	Polymorphism	867	867	1	7,809	SNP	12.80%
C	Polymorphism	871	871	1	8,086	SNP	84.30%
T	Polymorphism	871	871	1	8,086	SNP	15.70%
C	Polymorphism	872	872	1	8,172	SNP	23.50%
T	Polymorphism	872	872	1	8,172	SNP	76.40%
G	Polymorphism	876	876	1	8,382	SNP	26.50%
T	Polymorphism	876	876	1	8,382	SNP	73.20%
C	Polymorphism	900	900	1	9,311	SNP	10.70%
T	Polymorphism	900	900	1	9,311	SNP	88.90%
A	Polymorphism	946	946	1	10,071	SNP	88.30%
G	Polymorphism	946	946	1	10,071	SNP	11.60%
G	Polymorphism	1,338	1,338	1	7,364	SNP	67.60%
T	Polymorphism	1,338	1,338	1	7,364	SNP	32.20%
C	Polymorphism	1,390	1,390	1	8,283	SNP	34.40%
T	Polymorphism	1,390	1,390	1	8,283	SNP	65.50%
C	Polymorphism	1,673	1,673	1	9,348	SNP	77.90%
T	Polymorphism	1,673	1,673	1	9,348	SNP	21.00%
C	Polymorphism	1,675	1,675	1	9,365	SNP	21.60%
T	Polymorphism	1,675	1,675	1	9,365	SNP	77.10%
A	Polymorphism	1,676	1,676	1	9,428	SNP	21.50%
T	Polymorphism	1,676	1,676	1	9,428	SNP	68.50%
A	Polymorphism	1,679	1,679	1	9,567	SNP	80.70%
T	Polymorphism	1,679	1,679	1	9,567	SNP	18.20%
C	Polymorphism	1,683	1,683	1	9,522	SNP	33.00%
T	Polymorphism	1,683	1,683	1	9,522	SNP	66.90%
C	Polymorphism	1,684	1,684	1	9,536	SNP	89.30%
T	Polymorphism	1,684	1,684	1	9,536	SNP	10.20%
G	Polymorphism	1,688	1,688	1	9,425	SNP	85.70%
T	Polymorphism	1,688	1,688	1	9,425	SNP	14.20%
T	Polymorphism	1,711	1,711	1	8,915	Indel	64.80%
	Polymorphism	1,711	1,711	1	8,915	Indel	31.10%
C	Polymorphism	1,713	1,713	1	8,890	SNP	66.40%
T	Polymorphism	1,713	1,713	1	8,890	SNP	33.50%
A	Polymorphism	1,718	1,718	1	8,714	SNP	53.90%
C	Polymorphism	1,718	1,718	1	8,714	SNP	38.10%
C	Polymorphism	1,726	1,726	1	8,733	SNP	10.30%
G	Polymorphism	1,726	1,726	1	8,733	SNP	89.70%
C	Polymorphism	1,744	1,744	1	9,260	SNP	59.20%
T	Polymorphism	1,744	1,744	1	9,260	SNP	40.80%

A	Polymorphism	1,759	1,759	1	9,504	SNP	66.10%
G	Polymorphism	1,759	1,759	1	9,504	SNP	33.90%
A	Polymorphism	1,879	1,879	1	9,025	SNP	18.40%
C	Polymorphism	1,879	1,879	1	9,025	SNP	81.60%
C	Polymorphism	1,900	1,900	1	8,321	SNP	10.80%
T	Polymorphism	1,900	1,900	1	8,321	SNP	89.20%
C	Polymorphism	2,123	2,123	1	8,082	SNP	29.30%
T	Polymorphism	2,123	2,123	1	8,082	SNP	70.70%
C	Polymorphism	2,128	2,128	1	8,095	SNP	70.70%
T	Polymorphism	2,128	2,128	1	8,095	SNP	29.20%
C	Polymorphism	2,133	2,133	1	8,143	SNP	77.80%
T	Polymorphism	2,133	2,133	1	8,143	SNP	22.10%
A	Polymorphism	2,210	2,210	1	7,940	SNP	36.00%
G	Polymorphism	2,210	2,210	1	7,940	SNP	64.00%

Table S5. Heteroplasmic sites in the non-coding region of *H-nad5* minichromosome

Name	Type	Minimum	Maximum	Length	Coverage	Polymorphism Type	Variant Frequency
A	Polymorphism	23	23	1	3,279	SNP	42.30%
G	Polymorphism	23	23	1	3,279	SNP	57.60%
G	Polymorphism	315	315	1	2,555	SNP	31.80%
T	Polymorphism	315	315	1	2,555	SNP	67.80%
G	Polymorphism	363	363	1	2,738	SNP	61.70%
T	Polymorphism	363	363	1	2,738	SNP	36.40%
	Polymorphism	364	363	0	2,703	Indel	65.80%
A	Polymorphism	364	363	0	2,703	Indel	34.10%
G	Polymorphism	370	370	1	2,633	SNP	14.60%
T	Polymorphism	370	370	1	2,633	SNP	85.20%
G	Polymorphism	410	410	1	2,668	Indel	86.50%
	Polymorphism	410	410	1	2,668	Indel	12.60%
A	Polymorphism	411	411	1	2,692	SNP	19.50%
T	Polymorphism	411	411	1	2,692	SNP	78.00%
A	Polymorphism	413	413	1	2,728	SNP	48.60%
G	Polymorphism	413	413	1	2,728	SNP	47.60%
A	Polymorphism	418	418	1	2,774	Indel	70.30%
	Polymorphism	418	418	1	2,774	Indel	28.60%
A	Polymorphism	425	425	1	2,760	SNP	20.80%
T	Polymorphism	425	425	1	2,760	SNP	78.40%
A	Polymorphism	468	468	1	2,737	SNP	51.00%
T	Polymorphism	468	468	1	2,737	SNP	48.80%
A	Polymorphism	484	484	1	2,721	SNP	69.90%
G	Polymorphism	484	484	1	2,721	SNP	29.10%
A	Polymorphism	485	485	1	2,717	SNP	28.80%
G	Polymorphism	485	485	1	2,717	SNP	71.20%
A	Polymorphism	489	489	1	2,703	SNP	28.80%
G	Polymorphism	489	489	1	2,703	SNP	70.80%
	Polymorphism	494	494	1	2,749	Indel	58.10%

C	Polymorphism	494	494	1	2,749	Indel	41.80%
C	Polymorphism	497	497	1	2,782	SNP	41.70%
T	Polymorphism	497	497	1	2,782	SNP	57.40%
	Polymorphism	506	506	1	2,749	Indel	64.90%
A	Polymorphism	506	506	1	2,749	Indel	35.00%
	Polymorphism	507	507	1	2,749	Indel	64.90%
T	Polymorphism	507	507	1	2,749	Indel	35.00%
	Polymorphism	508	508	1	2,748	Indel	64.90%
T	Polymorphism	508	508	1	2,748	Indel	35.00%
A	Polymorphism	510	510	1	2,775	SNP	33.10%
C	Polymorphism	510	510	1	2,775	SNP	63.20%
C	Polymorphism	538	538	1	2,412	SNP	89.10%
T	Polymorphism	538	538	1	2,412	SNP	10.90%
C	Polymorphism	540	540	1	2,428	SNP	73.10%
T	Polymorphism	540	540	1	2,428	SNP	26.90%
A	Polymorphism	542	542	1	2,425	SNP	61.70%
T	Polymorphism	542	542	1	2,425	SNP	38.10%
C	Polymorphism	544	544	1	2,436	SNP	38.60%
T	Polymorphism	544	544	1	2,436	SNP	61.30%
A	Polymorphism	609	609	1	1,896	SNP	52.80%
T	Polymorphism	609	609	1	1,896	SNP	47.00%
C	Polymorphism	669	669	1	2,496	SNP	79.90%
T	Polymorphism	669	669	1	2,496	SNP	20.10%
C	Polymorphism	875	875	1	3,688	SNP	18.50%
T	Polymorphism	875	875	1	3,688	SNP	81.50%
C	Polymorphism	877	877	1	3,663	SNP	19.20%
T	Polymorphism	877	877	1	3,663	SNP	80.80%
A	Polymorphism	878	878	1	3,627	SNP	18.80%
C	Polymorphism	878	878	1	3,627	SNP	80.50%
C	Polymorphism	879	879	1	3,615	SNP	35.60%
T	Polymorphism	879	879	1	3,615	SNP	64.30%
C	Polymorphism	880	880	1	3,638	SNP	20.10%
T	Polymorphism	880	880	1	3,638	SNP	79.80%
A	Polymorphism	881	881	1	3,729	SNP	79.50%
T	Polymorphism	881	881	1	3,729	SNP	19.90%
C	Polymorphism	885	885	1	3,794	SNP	77.40%
T	Polymorphism	885	885	1	3,794	SNP	22.60%
C	Polymorphism	886	886	1	3,830	SNP	24.70%
T	Polymorphism	886	886	1	3,830	SNP	75.10%
G	Polymorphism	890	890	1	3,881	SNP	30.50%
T	Polymorphism	890	890	1	3,881	SNP	69.30%
A	Polymorphism	919	919	1	4,412	SNP	10.90%
C	Polymorphism	919	919	1	4,412	SNP	88.80%
C	Polymorphism	1,404	1,404	1	3,895	SNP	17.20%
T	Polymorphism	1,404	1,404	1	3,895	SNP	82.80%
A	Polymorphism	1,419	1,419	1	3,908	SNP	14.30%

G	Polymorphism	1,419	1,419	1	3,908	SNP	85.60%
C	Polymorphism	1,464	1,464	1	4,065	SNP	69.80%
T	Polymorphism	1,464	1,464	1	4,065	SNP	30.10%
C	Polymorphism	1,687	1,687	1	4,725	SNP	73.20%
T	Polymorphism	1,687	1,687	1	4,725	SNP	25.80%
C	Polymorphism	1,689	1,689	1	4,753	SNP	26.60%
T	Polymorphism	1,689	1,689	1	4,753	SNP	72.20%
A	Polymorphism	1,690	1,690	1	4,758	SNP	26.70%
C	Polymorphism	1,690	1,690	1	4,758	SNP	18.80%
T	Polymorphism	1,690	1,690	1	4,758	SNP	54.30%
C	Polymorphism	1,692	1,692	1	4,805	SNP	81.30%
T	Polymorphism	1,692	1,692	1	4,805	SNP	18.60%
A	Polymorphism	1,693	1,693	1	4,811	SNP	74.40%
T	Polymorphism	1,693	1,693	1	4,811	SNP	25.00%
C	Polymorphism	1,697	1,697	1	4,748	SNP	34.10%
T	Polymorphism	1,697	1,697	1	4,748	SNP	65.90%
C	Polymorphism	1,698	1,698	1	4,738	SNP	82.10%
T	Polymorphism	1,698	1,698	1	4,738	SNP	17.60%
G	Polymorphism	1,702	1,702	1	4,688	SNP	88.80%
T	Polymorphism	1,702	1,702	1	4,688	SNP	11.10%
T	Polymorphism	1,725	1,725	1	4,489	Indel	83.50%
	Polymorphism	1,725	1,725	1	4,489	Indel	13.90%
C	Polymorphism	1,727	1,727	1	4,469	SNP	85.20%
T	Polymorphism	1,727	1,727	1	4,469	SNP	14.80%
A	Polymorphism	1,732	1,732	1	4,404	SNP	73.00%
C	Polymorphism	1,732	1,732	1	4,404	SNP	17.00%
C	Polymorphism	1,758	1,758	1	4,698	SNP	56.20%
T	Polymorphism	1,758	1,758	1	4,698	SNP	43.80%
A	Polymorphism	1,773	1,773	1	4,649	SNP	43.30%
G	Polymorphism	1,773	1,773	1	4,649	SNP	56.70%
A	Polymorphism	1,893	1,893	1	4,660	SNP	14.50%
C	Polymorphism	1,893	1,893	1	4,660	SNP	85.40%
C	Polymorphism	1,914	1,914	1	4,253	SNP	33.30%
T	Polymorphism	1,914	1,914	1	4,253	SNP	66.60%
C	Polymorphism	2,137	2,137	1	4,178	SNP	59.20%
T	Polymorphism	2,137	2,137	1	4,178	SNP	40.80%
C	Polymorphism	2,142	2,142	1	4,204	SNP	41.50%
T	Polymorphism	2,142	2,142	1	4,204	SNP	58.40%
C	Polymorphism	2,147	2,147	1	4,183	SNP	47.90%
T	Polymorphism	2,147	2,147	1	4,183	SNP	52.00%
A	Polymorphism	2,224	2,224	1	3,690	SNP	60.70%
G	Polymorphism	2,224	2,224	1	3,690	SNP	39.30%

Table S6. PCR primers used to amplify and sequence the mitochondrial genes and genomes of the elephant louse, *Haematomyzus elephantis*

Primer	Target gene or region	Sequence (5'-3')	Use
mtd6	*cox1*	GGAGGATTTGGAAATTGATTAGTTCC	Amplifying *cox1* fragment with mtd11
mtd11	*cox1*	ACTGTAAATATATGATGAGCTCA	Amplifying *cox1* fragment with mtd6
mtd16	*cox2*	ATTGGACATCAATGATATTGA	Amplifying *cox2* fragment with mtd18
mtd18	*cox2*	CCACAAATTTCTGAACATTGACCA	Amplifying *cox2* fragment with mtd16
12SA	*rrnS*	TACTATGTTACGACTTAT	Amplifying *rrnS* fragment with 12SB
12SB	*rrnS*	AAACTAGGATTAGATACCC	Amplifying *rrnS* fragment with 12SA
mtd32m	*rrnL*	ACGCCGGTCTGAACTCAAATCATGTAA	Amplifying *rrnL* fragment with mtd34
mtd34	*rrnL*	CGCCTGTTTAACAAAAACAT	Amplifying *rrnL* fragment with mtd34
ELC1F	*cox1*	TTTATTCTGGTTTTTGGTCATCCAGAAGT	Amplifying *trnI-cox1-trnE* minichromosome with ELC1R
ELC1R	*cox1*	AGCATAGTAATCGCCCCTGCAAGGACCGG	Amplifying *trnI-cox1-trnE* minichromosome with ELC1F
ELC2F	*cox2*	GAGTTGATCAGAGTGTGATTCTCCCGGTTC	Amplifying *trnY-cox2-trnE* minichromosome with ELC2R1
ELC2R1	*cox2*	GCGAACATCTCAGACATTTAAATCCCTTAT	Amplifying *trnY-cox2-trnE* minichromosome with ELC2F
EL12SF1	*rrnS*	AATTTGTGGTAAGATGTTCGTGTAATTTCG	Amplifying *trnL$_2$(taa)-rrnS* minichromosome with EL12SR
EL12SR	*rrnS*	ATTGTCCTTGCACACCGCCGTCGGAAGGG	Amplifying *trnL$_2$(taa)-rrnS* minichromosome with EL12SF1
EL16SF	*rrnL*	GCGGCCCTTCACATCAAGGGTGCAGCGGG	Amplifying *trnL$_1$(tag)-rrnL-trnV* minichromosome with LX16SR
LX16SR	*rrnL*	GACTGTGCTAAGGTAGCATAAT	Amplifying *trnL$_1$(tag)-rrnL-trnV* minichromosome with EL16SF
ELC1seqF	*cox1*	TTATTGGTGTAAATGTTACTTTCTTCCCTC	Sequencing by primer walking
ELC1seqR	*cox1*	TCAATTGGCGAAACCTCCAATTATAATTGG	Sequencing by primer walking
EL16SF1	*rrnL*	CAAAAGGTACAGGAACAACCCTTACTTGTC	Sequencing by primer walking
ELNCRf	NCR*	CATGAGTCACGTCATGGAGGCTGTGTGGAG	Sequencing by primer walking

ELNCRr	NCR	AATTAGGGTCAAATTT CGACCTCCCAAGGG	Sequencing by primer walking
ELNCRf1	NCR	CTTCTTATCCATCTCT ATGGATAATTACCC	Sequencing by primer walking
ELNCRf2	NCR	TTAGCCAGGTAAAAG GGTGATTTTGCCTTC	Sequencing by primer walking
ELNCRf3	NCR	GATTTCAATCTCTGAT GAGATGGCCTCTAC	Sequencing by primer walking
ELNCRf4	NCR	GGGAGAGTACCCCCA GAGAGGATACCCACT	Sequencing by primer walking
ELNCRf5	NCR	AATCCTCAGGTGATA GAGAGATGGAACCTC	Sequencing by primer walking
ELNCRf6	NCR	AAGTGAGATAGGACC ATCTTCATCGATCGA	Sequencing by primer walking
USFB1567	NCR	TGAGAATTGATTTCAA TCCTCAGGTGATAG	Amplifying coding regions of all minichromosomes with ELR
ELR	NCR	AATAACATATTTCTG TTGCAAAGTCACTC	Amplifying coding regions of all minichromosomes with USFB1567

*Note: NCR for non-coding region.

Table S7. Species of insects for phylogenetic analyses

Species	Common name	GenBank accession number	Reference
Haematomyzus elephantis	Elephant louse	KF933032-41	This manuscript
Pediculus humanus	Human body louse	FJ499473-90	Shao et al.[8]
Pediculus capitis	Human head louse	JX080388-407	Shao et al.[9]
Pthirus pubis	Human pubic louse	EU219988-95, HM241895-8	Shao et al.[9]
Haematopinus suis	Domestic pig louse	KC814602–10	Jiang et al.[11]
Haematopinus apri	Wild pig louse	KC814611–19	Jiang et al.[11]
Polyplax asiatica	Rat louse	KF647751-61	Dong et al.[10]
Polyplax spinulosa	Rat louse	KF647762-72	Dong et al.[10]
Bothriometopus macrocnemis	Screamer louse	NC_009983	Cameron et al.[5]
Campanulotes bidentatus compar	Small pigeon louse	NC_007884	Covacin et al.[4]
Ibidoecus bisignatus	Glossy ibis head louse	NC_015999	Cameron et al.[6]
Heterodoxus macropus	Wallaby louse	NC_002651	Shao et al.[3]
Liposcelis bostrychophila	Booklouse	JN645275-6	Wei et al.[38]
Psococerastis albimaculata	Barklouse	NC_021400	Li et al.[39]
Longivalvus hyalospilus	Barklouse	JQ910986	Li et al.[39]
Lepidopsocid sp.	Barklouse	NC_004816	Shao et al.[40]
Alloeorhynchus bakeri	True bug	NC_016432	Li et al.[41]

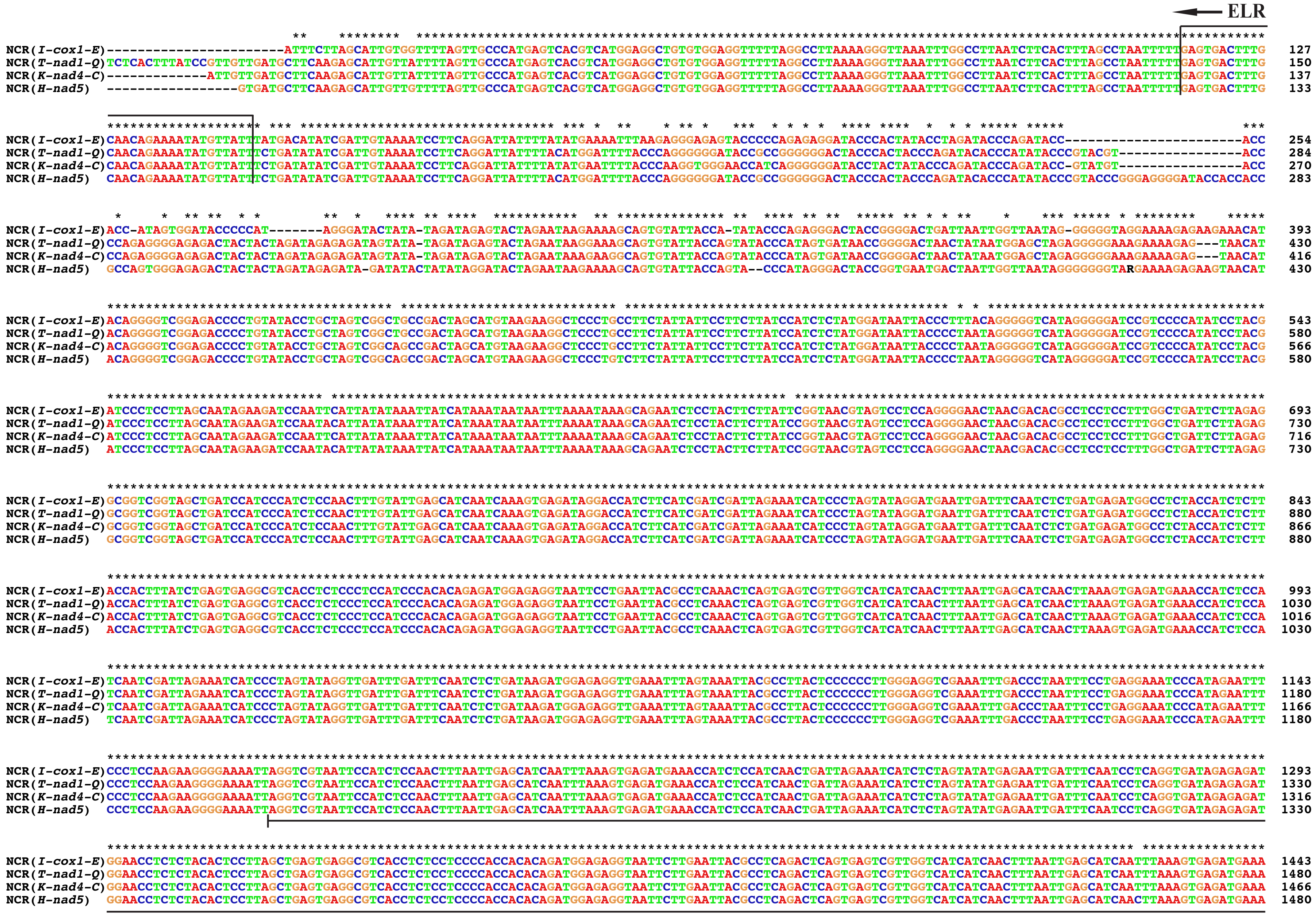

(Fig. S1)

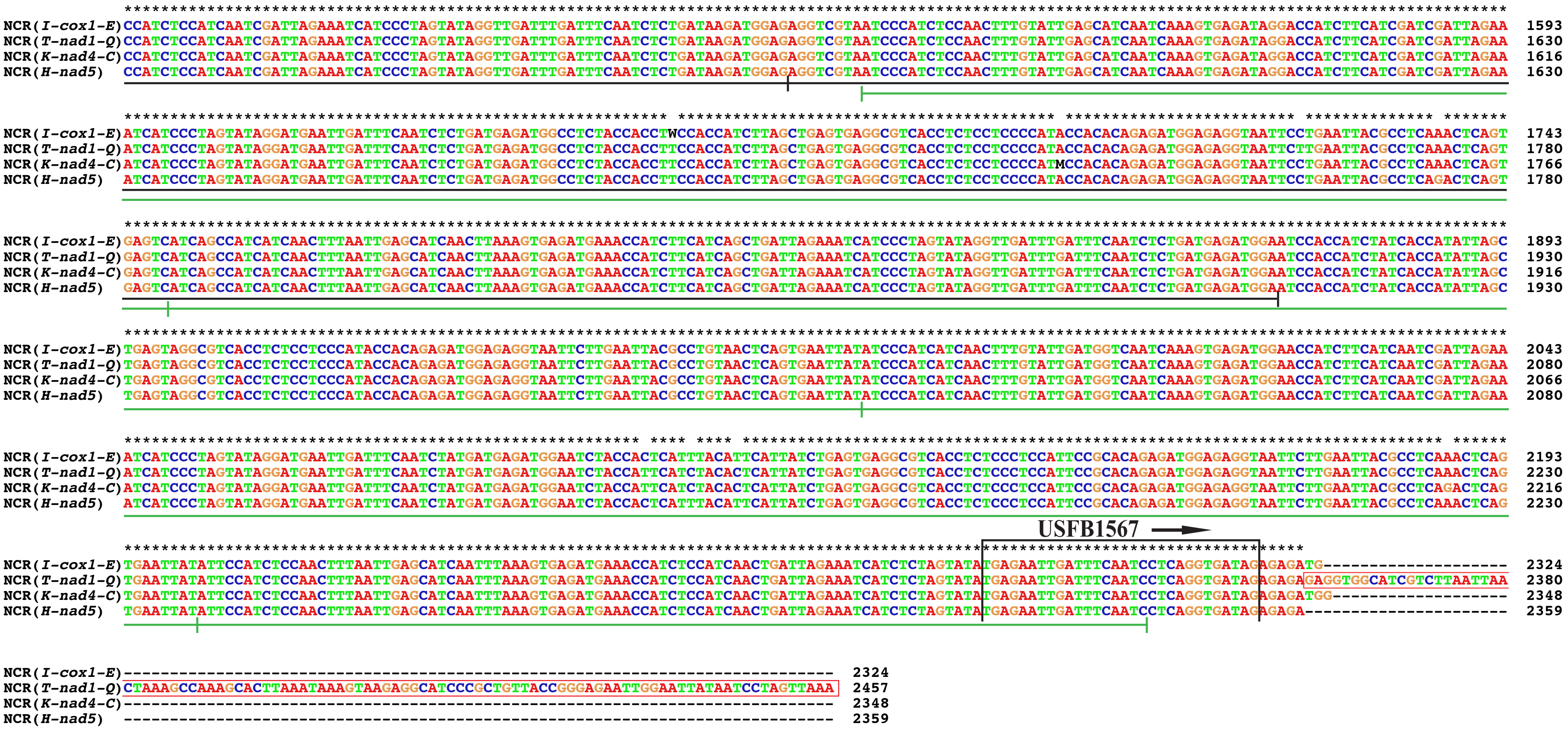

Fig. S1. Alignment of the full-length sequences of the non-coding regions of four mitochondrial minichromosomes of the elephant louse, *Haematomyzus elephantis*. USFB1567 and ELR are the primers used to amplify the coding regions of the 10 minichromosomes identified in the current study. The two 346-bp repeat units are underlined in black and the four 225-bp repeat units are underlined in green. Short vertical lines in black and green separate repeat units. The 99-bp sequence at the 3' end unique to *T-nad1-Q* minichromosome is in red-line box. Asteroisks "*" indicate identical nucleotides whereas hyphens "-" indicate absent nucleotides.

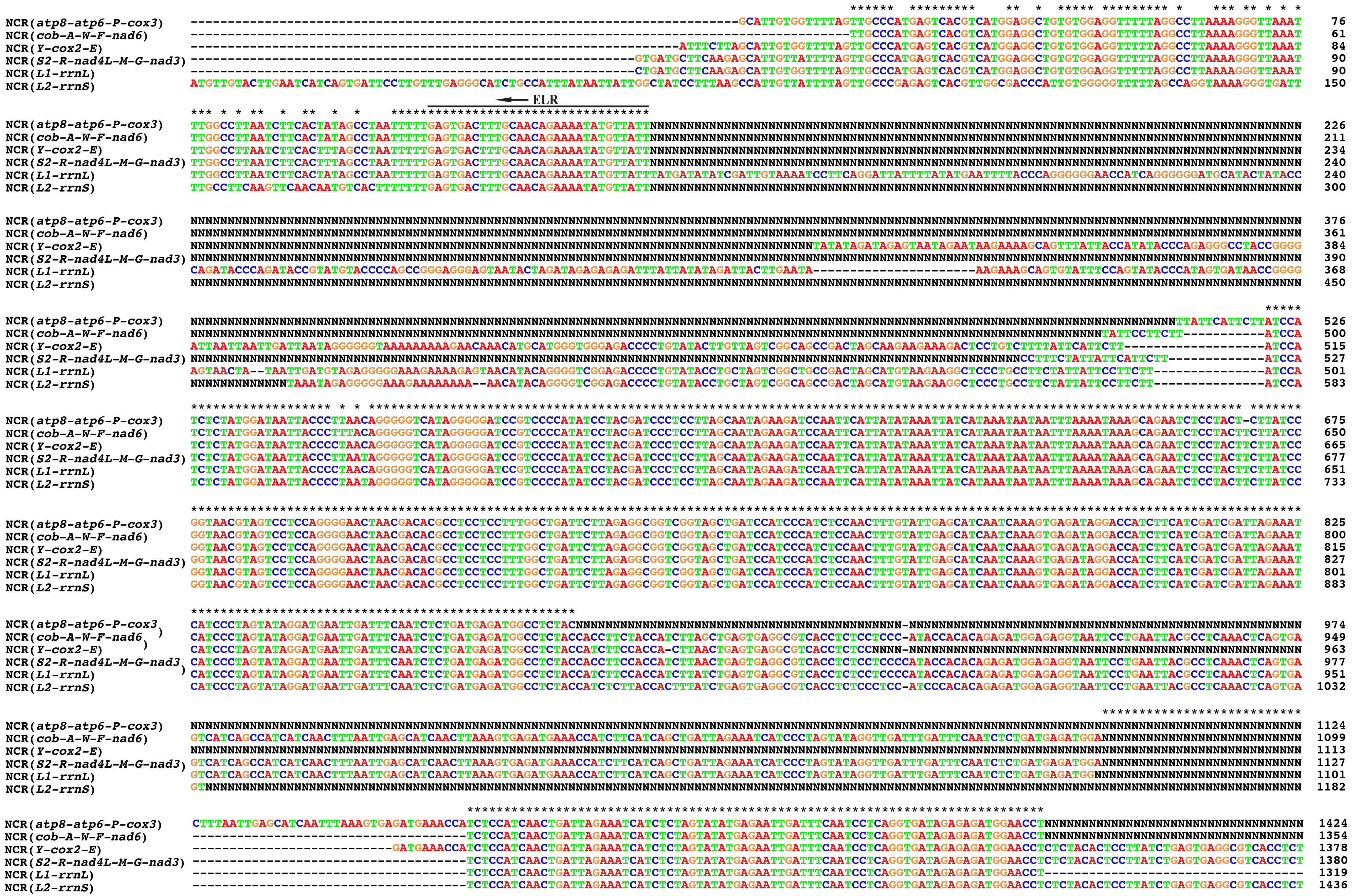
NCR(atp8-atp6-P-cox3)
NCR(cob-A-W-F-nad6)
NCR(Y-cox2-E)
NCR(S2-R-nad4L-M-G-nad3)
NCR(L1-rrnL)
NCR(L2-rrnS)
ELR

(Fig. S2)

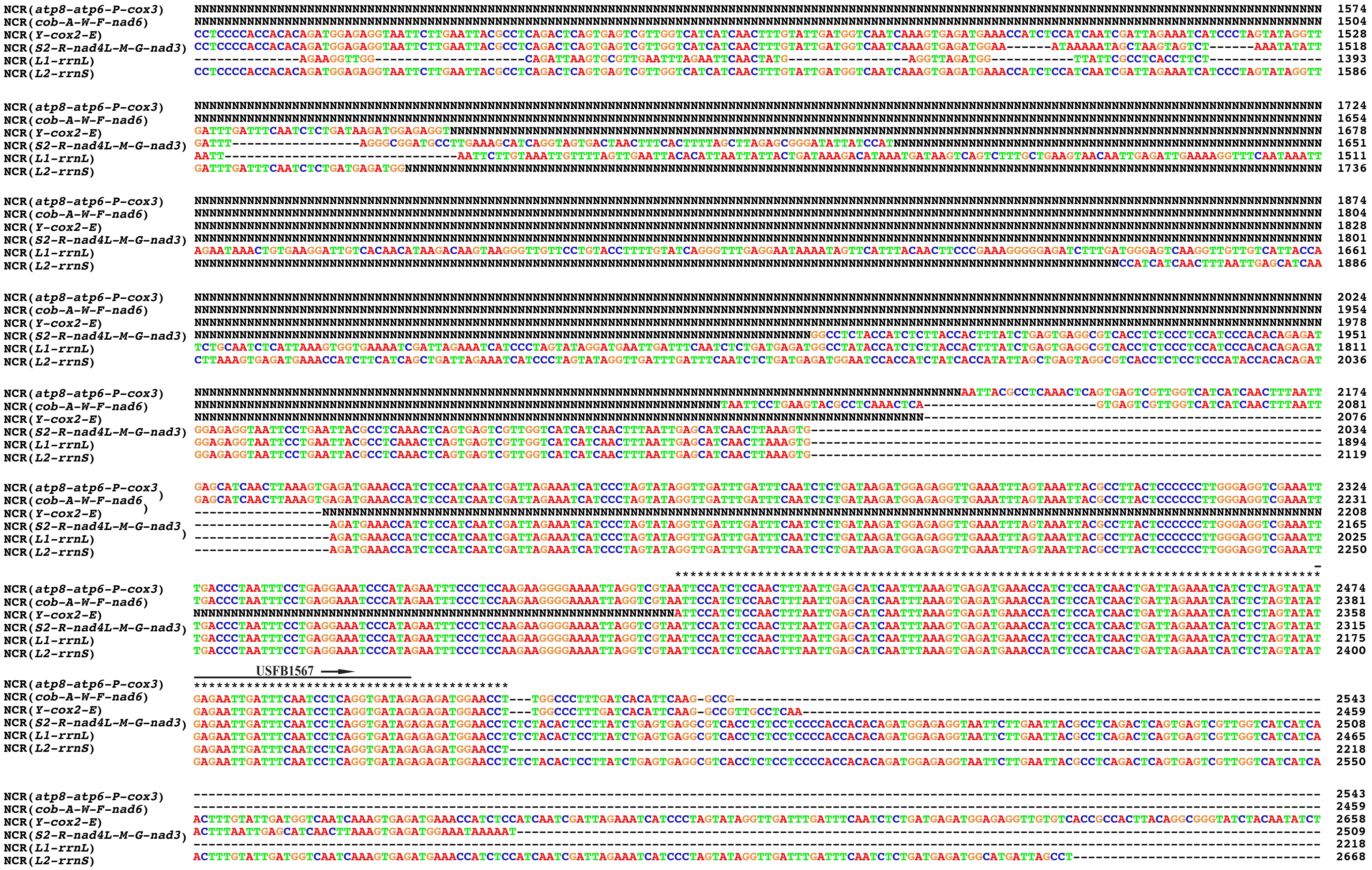

Fig. S2. Alignment of the sequences of the partially-sequenced non-coding regions of six mitochondrial minichromosomes of the elephant louse, *Haematomyzus elephantis*. The un-sequenced nucleotides were indicated with "N". ELR (downstream coding region) and USFB1567 (upstream coding region) are the primers used to amplify the coding regions of the 10 minichromosomes of the elephant louse identified in the current study. Asteroisks "*" indicate identical nucleotides whereas hyphens "-" indicate absent nucleotides.

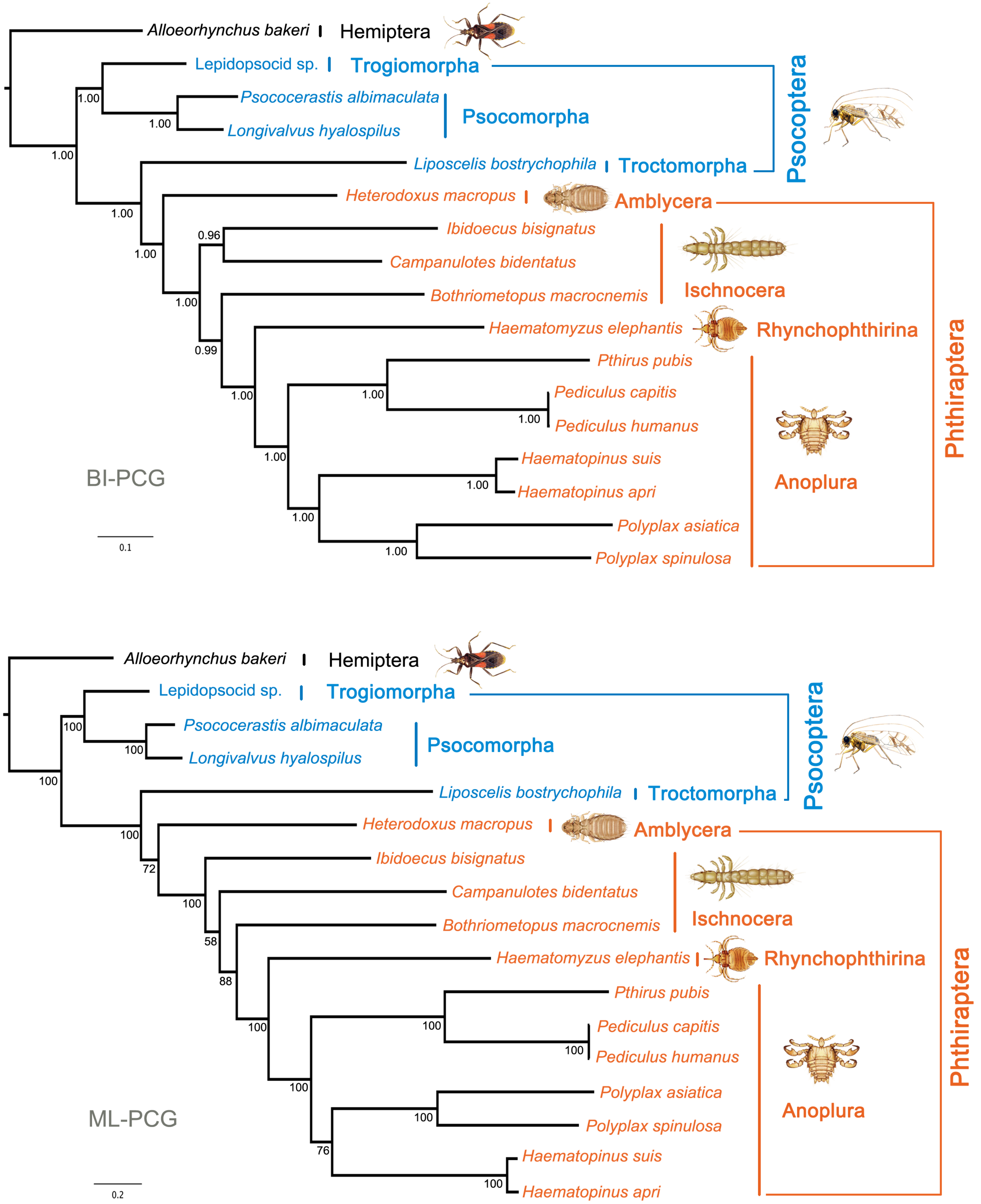

Fig. S3(A). Phylogenetic relationship of the elephant louse, *Haematomyzus elephantis*, with other parasitic lice (Phthiraptera), booklice and barklice (Psocoptera). The trees were constructed using Bayesian (upper) and maximum likelihood (lower) methods with concatenated sequences of 11 mitochondrial protein-coding genes (8,040 nucleotides). Posterior probability (BI-PCG tree) and bootstrap support value (%, ML-PCG tree) for each grouping were indicated near the branch nodes. Trees were rooted with the true bug, *Alloeorhynchus bakeri*.

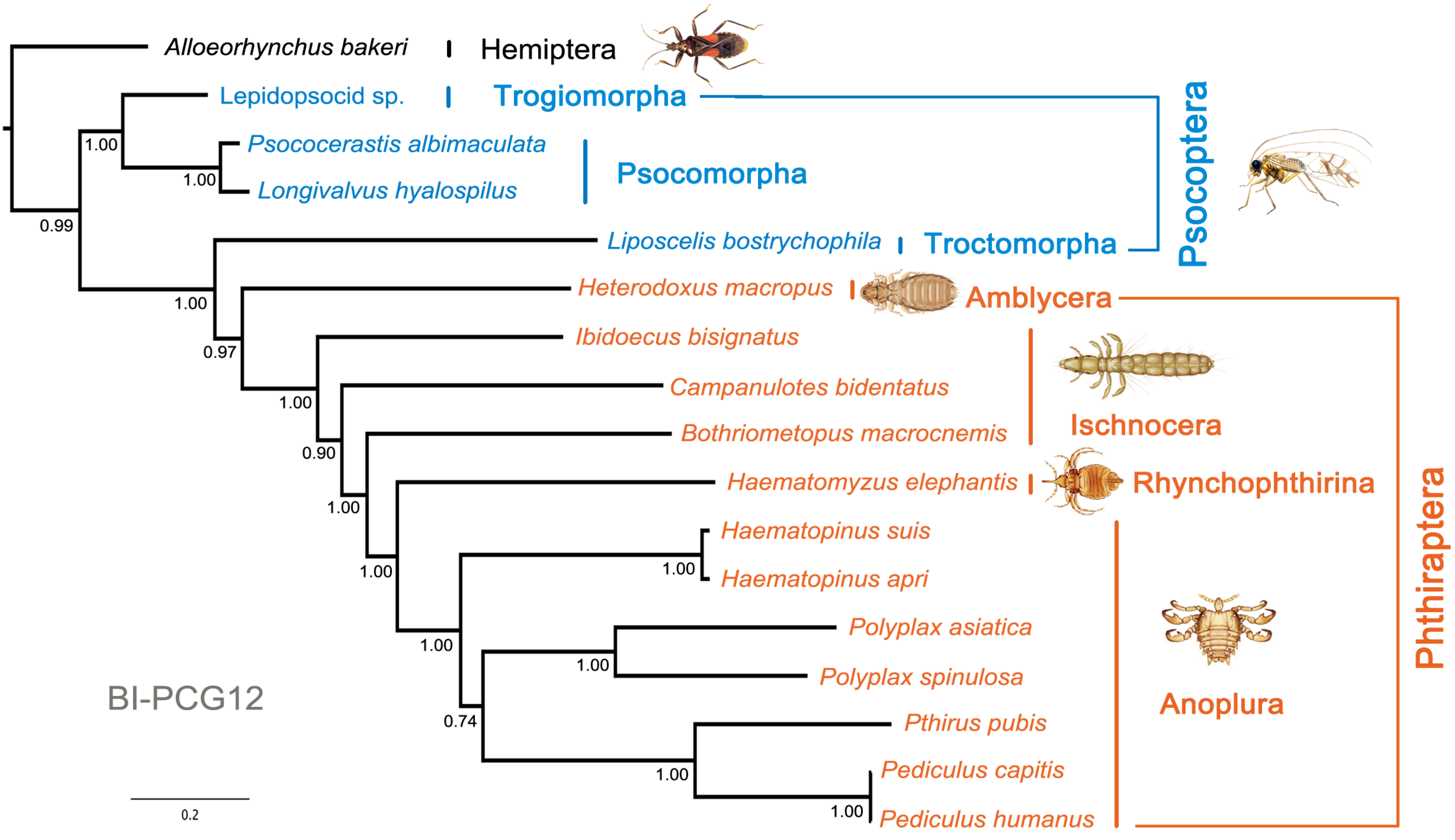

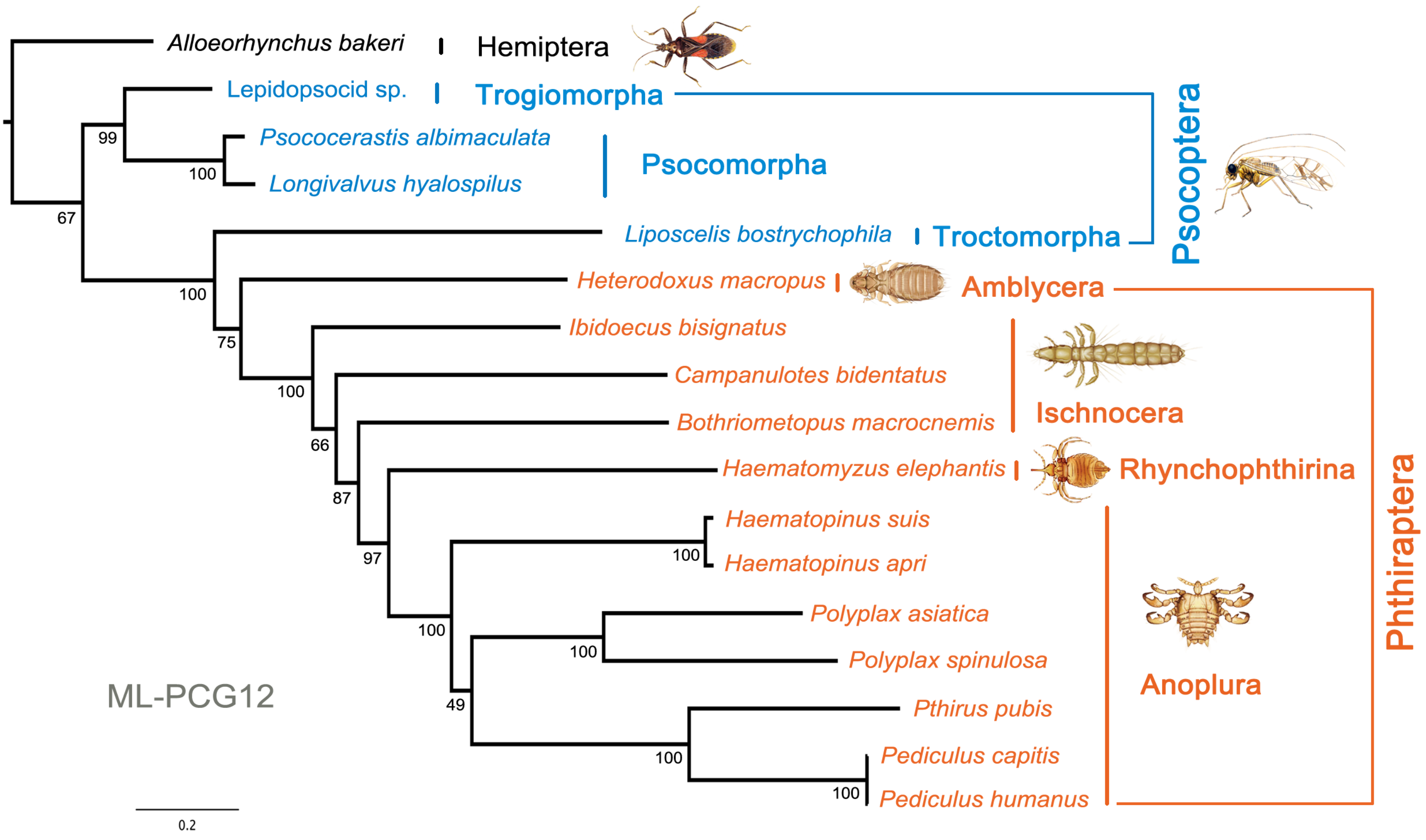

Fig. S3(B). Phylogenetic relationship of the elephant louse, *Haematomyzus elephantis*, with other parasitic lice (Phthiraptera), booklice and barklice (Psocoptera). The trees were constructed using Bayesian (upper) and maximum likelihood (lower) methods with concatenated sequences of 11 mitochondrial protein-coding genes at the first and second coding positions (5,360 nucleotides). Posterior probability (BI-PCG12 tree) and bootstrap support value (%, ML-PCG12 tree) for each grouping were indicated near the branch nodes. Trees were rooted with the true bug, *Alloeorhynchus bakeri.*

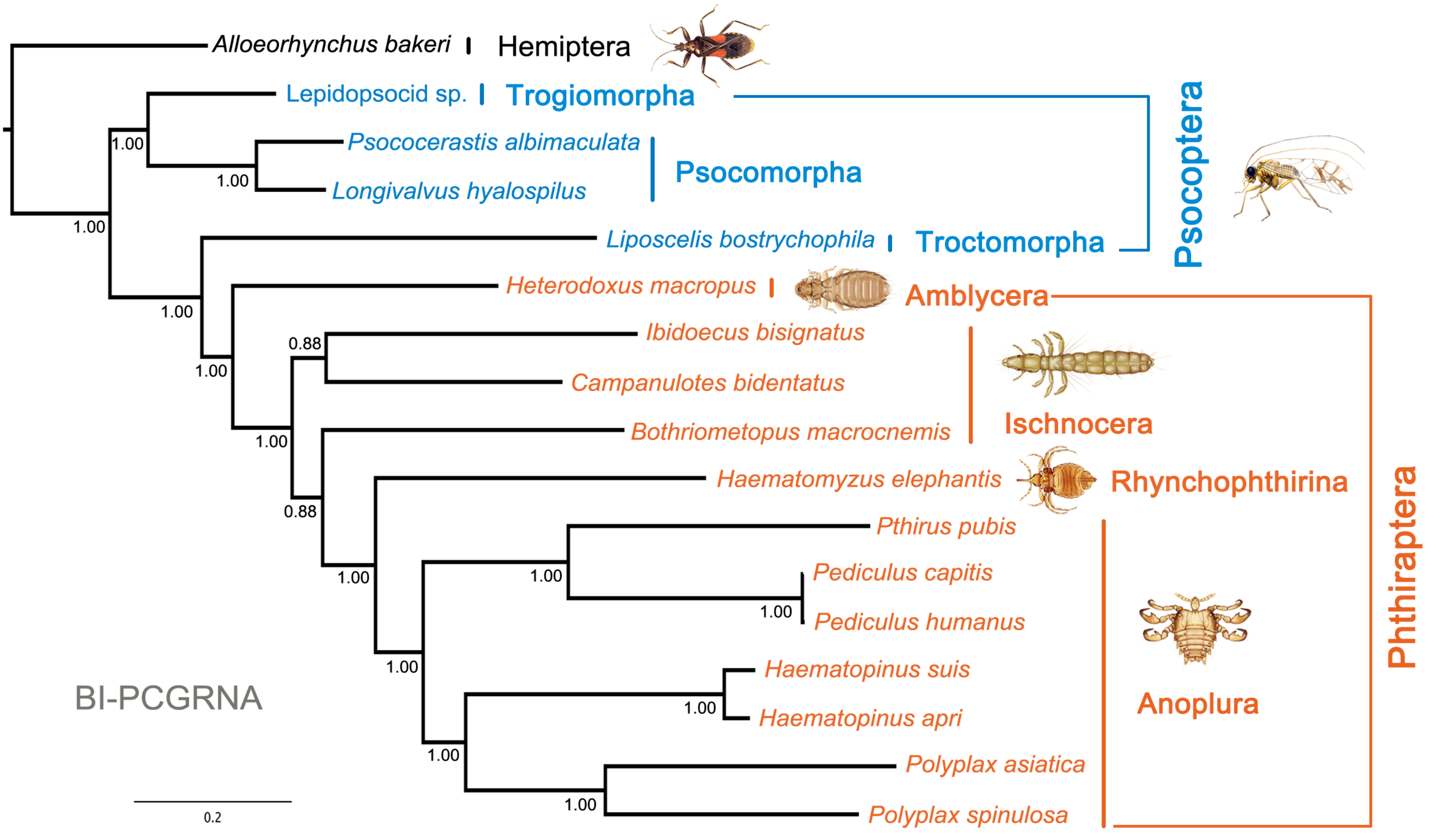

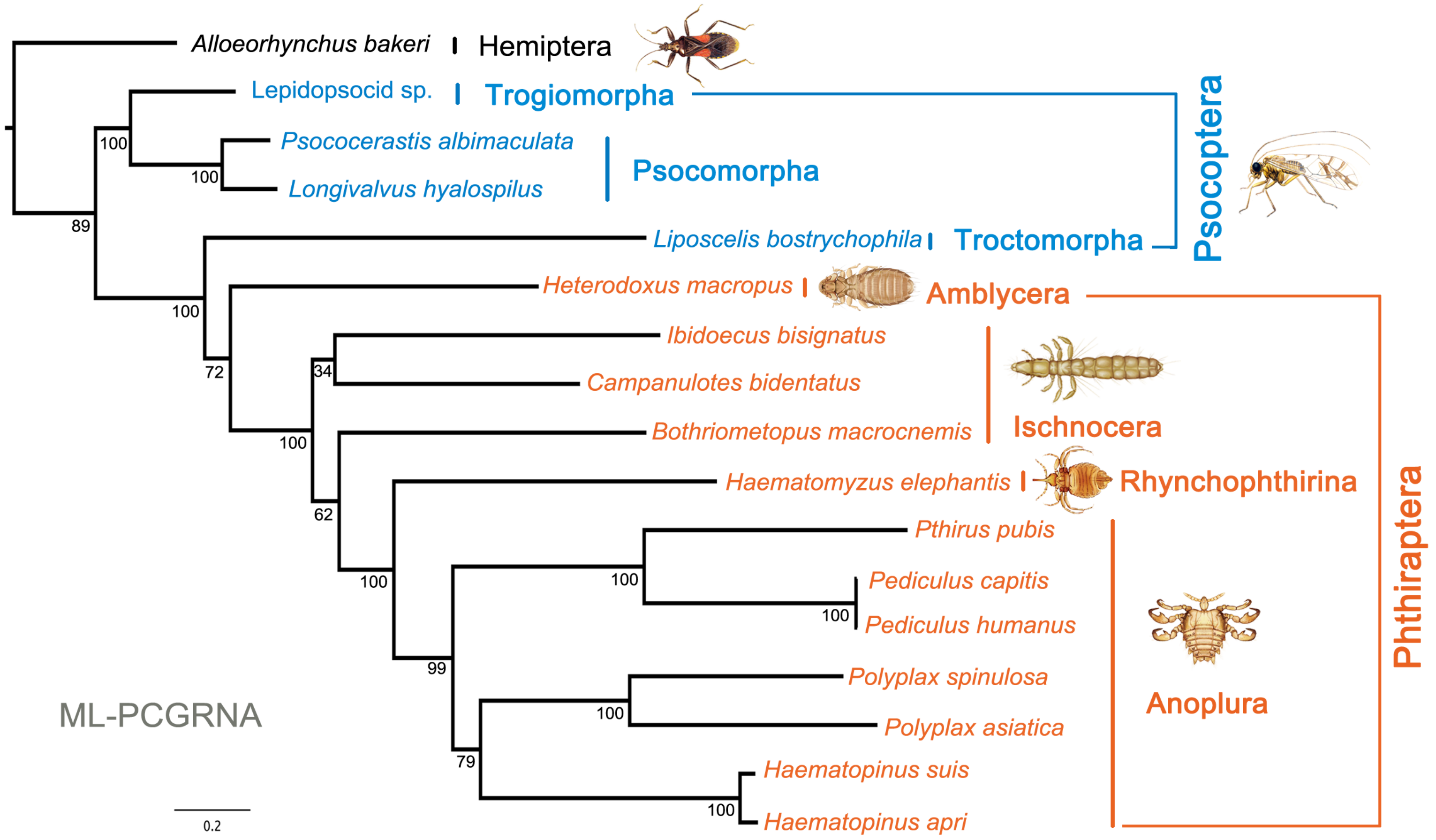

Fig. S3(C). Phylogenetic relationship of the elephant louse, *Haematomyzus elephantis*, with other parasitic lice (Phthiraptera), booklice and barklice (Psocoptera). The trees were constructed using Bayesian (upper) and maximum likelihood (lower) methods with concatenated sequences of 11 mitochondrial (mt) protein-coding genes and the mt rRNA genes (9,530 nucleotides). Posterior probability (BI-PCGRNA tree) and bootstrap support value (%, ML-PCGRNA tree) for each grouping were indicatednear the branch nodes. Trees were rooted with the true bug, *Alloeorhynchus bakeri.* Drawing of insects here and in Fig. S3(A) and (B) was by Hu Li.

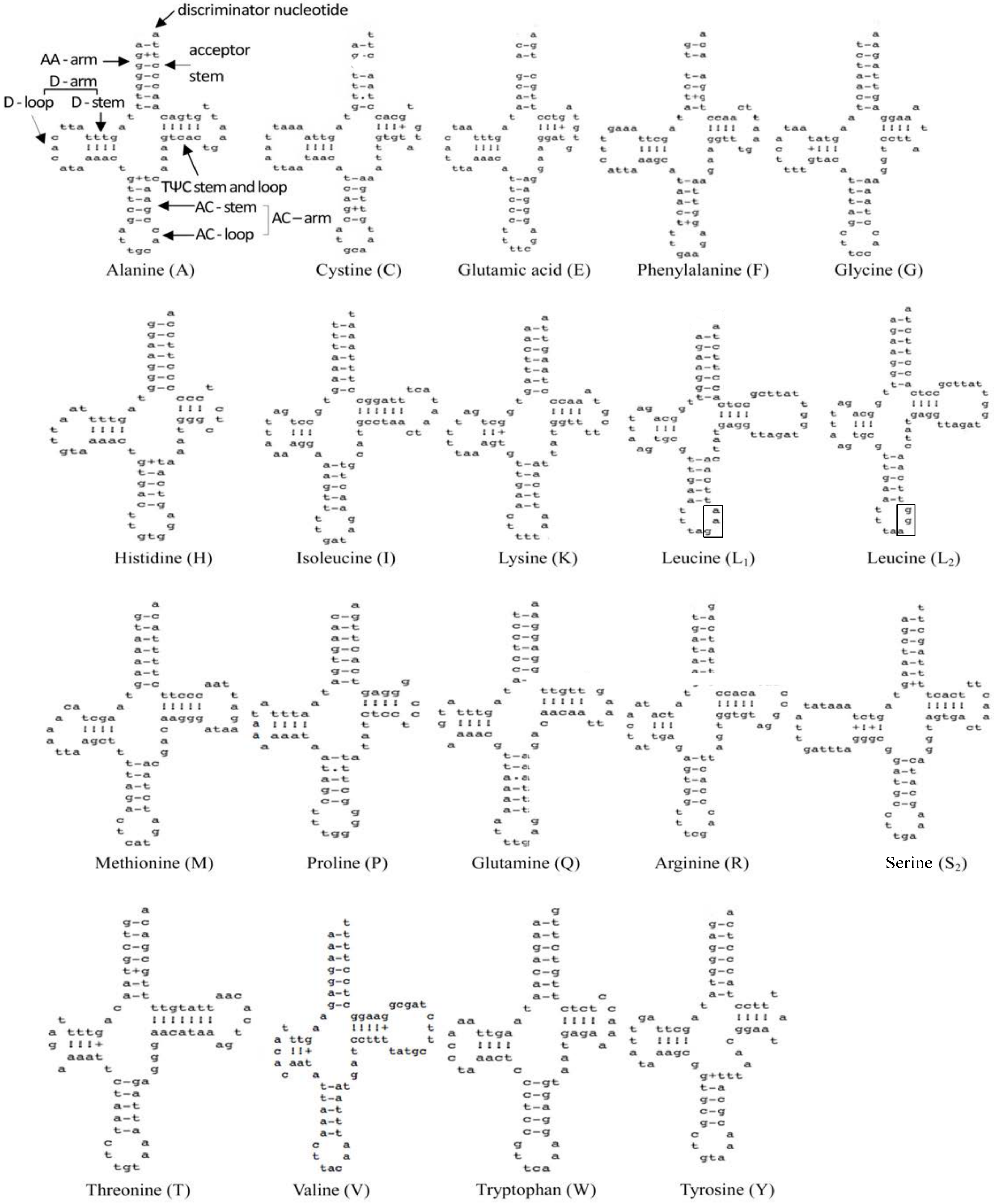

Fig. S4. Inferred secondary structures of the mitochondrial tRNAs of the elephant louse, *Haematomyus elephantis*. The three nucleotides that differ between L1 and L2 are boxed.

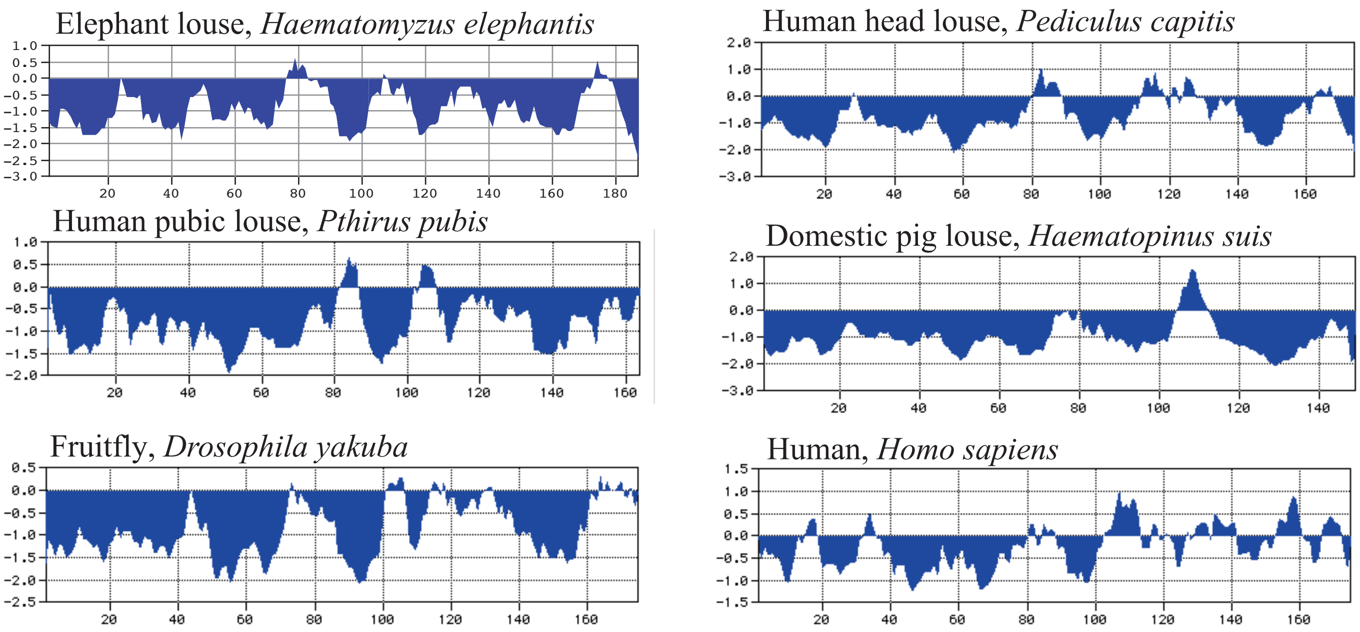

Fig. S5. Comparison of the Hopp/Woods hydrophilicity profiles of NAD6 proteins of the elepahnt louse, *Haematomyzus elephantis,* with the human lice, pig louse, fly and human. Amino acids are along the horizontal lines. Hydrophilicity values of amino acids are along the vertical lines.

```
           1        10        20        30        40        50        60
He_NAD6        MLMMLVLKILFWFTQILFWLSSSIVTELISLISFTIIVSFWSFMTWSSEFAPLMFTYATLGG
Ha_NAD6         MILWTCCLIAALCSLYLWMASSCSMVVLSLSVQIVMYSVMLAMSVTHYWIVILSIMGLLGG
Hs_NAD6         MILWTCCLIAALCSLYLWMASSCSMVVLSLSVQIVMYSVMLAMSVTHYWIVILSIMGLLGG
Pp_NAD6           MVKVLYLLSLVCLVYMSLTKSLVIMLLDMAFMGVIIKVLLSLYVTSNWPWLLLLLGVVGG
Ph_NAD6    MTLSIPLSLLLYVVAMFFTFLILVGSDLLLKLFSLAMTVFTVGLLILLNSYSSWSWLLLWLGILGG

              70        80        90       100       110       120       130
He_NAD6    LTILTTFMIMWSPR----WSKVGRPLHSLSKVVAWFVVVVLFLCLTDSIMLDLNTGVTLPWLWV-
Ha_NAD6    LIVFLSFILMMLPNP---DLGVFNKWNYVGLSMLMLLMLTSIYLPVVDKNEPELLPISLHLYAYG-
Hs_NAD6    LIVFLSFILMMLPNP---DLGVFNKWNYVGLSMLMLLMLTSIYLPVVDKNEPELLPISLHLYAYG-
Pp_NAD6    LIVVISVILVVMPGPSA-LHWSVDKGSTVTFVLVLILAVAISYLDELDSFFSLTYSTCRLNMLSN-
Ph_NAD6    LIVSLSMAFIVTPKINSSKDWSSSLSFMSAFWILTSVMLSAMMWKVEFKEWSDLYSTESVSETYNS

               140       150       160       170       180       190
He_NAD6    -NTSWMGVQDWSAGVSNLHWS-WIESHFVWPNVCIIGFMLMILLFSLLIVDSSTYGKGGSLYLALLF
Ha_NAD6    -LCFFLLLFVLLIMVGIMNYG-YKSIYL
Hs_NAD6    -LCFFLLLFVLLIMVGIMNYG-YKSIYL
Pp_NAD6    -SSFLYPEGVLVLLIGLLLMLPVMEVLLSFYGRSAIHYWE
Ph_NAD6    LLMDLKVYTAVVILLIYILMLPVMEVLTSPYSRSSAAGWGIY
```

Fig. S6. Alignment of the putative amino acid sequences of mitochondrial NAD6 proteins of the elephant louse and four sucking lice (Anoplura). Conserved amino acid sequences were highlighted in colors. Abbreviations of species names are: He – *Haematomyzus elephantis* (elephant louse), Ha – *Haematopinus apri* (wild pig louse), Hs – *Haematopinus suis* (domestic pig louse), Pp – *Pthirus pubis* (human pubic louse), and Ph – *Pediculus humanus* (human body louse). Alignment was with Geneious 7.0.4. BLOSUM cost matrix was used; gap open cost was 10 and gap extend cost was 5.
