# Supplementary material for: Fragmented mitochondrial genomes in two suborders of parasitic lice of eutherian mammals (Anoplura and Rhynchophthirina, Insecta)
Source: Sci Rep. 2015 Nov 30;5:17389. doi: 10.1038/srep17389 (PMC4663631; doi:10.1038/srep17389)
Supplement: Supplementary Dataset 1 [file srep17389-s2.doc]

4 2477

NCR_I-cox1 ---------- ---------- ---ATTTCTT AGCATTGTGG TTTTAGTTGC

NCR_T-nad1 TCTCACTTTA TCCGTTGTTG ATGCTTCAAG AGCATTGTTA TTTTAGTTGC

NCR_K-nad4 ---------- ---ATTGTTG ATGCTTCAAG AGCATTGTTA TTTTAGTTGC

NCR_H-nad5 ---------- -------GTG ATGCTTCAAG AGCATTGTTG TTTTAGTTGC

CCATGAGTCA CGTCATGGAG GCTGTGTGGA GGTTTTTAGG CCTTAAAAGG

CCATGAGTCA CGTCATGGAG GCTGTGTGGA GGTTTTTAGG CCTTAAAAGG

CCATGAGTCA CGTCATGGAG GCTGTGTGGA GGTTTTTAGG CCTTAAAAGG

CCATGAGTCA CGTCATGGAG GCTGTGTGGA GGTTTTTAGG CCTTAAAAGG

GTTAAATTTG GCCTTAATCT TCACTTTAGC CTAATTTTTG AGTGACTTTG

GTTAAATTTG GCCTTAATCT TCACTTTAGC CTAATTTTTG AGTGACTTTG

GTTAAATTTG GCCTTAATCT TCACTTTAGC CTAATTTTTG AGTGACTTTG

GTTAAATTTG GCCTTAATCT TCACTTTAGC CTAATTTTTG AGTGACTTTG

CAACAGAAAA TATGTTATTT ATGACATATC GATTGTAAAA TCCTTCAGGA

CAACAGAAAA TATGTTATTT CTGATATATC GATTGTAAAA TCCTTCAGGA

CAACAGAAAA TATGTTATTT CTGATATATC GATTGTAAAA TCCTTCAGGA

CAACAGAAAA TATGTTATTT CTGATATATC GATTGTAAAA TCCTTCAGGA

TTATTTTATA TGAAAATTTA AGAGGGAGAG TACCCCCAGA GAGGATACCC

TTATTTTACA TGGATTTTAC CCAGGGGGGA TACCGCCGGG GGGACTACCC

TTATTTTATA TGAATTTTAC CCAAGGTGGG AACCATCAGG GGGGATACCT

TTATTTTACA TGGATTTTAC CCAGGGGGGA TACCGCCGGG GGGACTACCC

ACTATACCTA GATACCCAGA TACC------ ---------- -------ACC

ACTACCCAGA TACACCCATA TACCCGTACG T--------- -------ACC

ACTATACCCA GATACCCAGA TACC-GTATG T--------- -------ACC

ACTACCCAGA TACACCCATA TACCCGTACC CGGGAGGGGA TACCACCACC

ACC-ATAGTG GATACCCCCA T-------AG GGATACTATA -TAGATAGAG

CCAGAGGGGA GAGACTACTA CTAGATAGAG AGATAGTATA -TAGATAGAG

CCAGAGGGGA GAGACTACTA CTAGATAGAG AGATAGTATA -TAGATAGAG

GCCAGTGGGA GAGACTACTA CTAGATAGAG ATA-GATATA CTATATAGGA

TACTAGAATA AGAAAAGCAG TGTATTACCA -TATACCCAG AGGGACTACC

TACTAGAATA AGGAAAGCAG TGTATTACCA GTATACCCAT AGTGATAACC

TACTAGAATA AAGAAGGCAG TGTATTACCA GTATACCCAT AGTGATAACC

TACTAGAATA AGAAAAGCAG TGTATTACCA GTA--CCCAT AGGGACTACC

GGGGACTGAT TAATTGGTTA ATAG-GGGGG TAGGAAAAGA GAAGAAACAT

GGGGACTAAC TATAATGGAG CTAGAGGGGG AAAGAAAAGA G---TAACAT

GGGGACTAAC TATAATGGAG CTAGAGGGGG AAAGAAAAGA G---TAACAT

GGTGAATGAC TAATTGGTTA ATAGGGGGGG TARGAAAAGA GAAGTAACAT

ACAGGGGTCG GAGACCCCTG TATACCTGCT AGTCGGCTGC CGACTAGCAT

ACAGGGGTCG GAGACCCCTG TATACCTGCT AGTCGGCTGC CGACTAGCAT

ACAGGGGTCG GAGACCCCTG TATACCTGCT AGTCGGCAGC CGACTAGCAT

ACAGGGGTCG GAGACCCCTG TATACCTGCT AGTCGGCAGC CGACTAGCAT

GTAAGAAGGC TCCCTGCCTT CTATTATTCC TTCTTATCCA TCTCTATGGA

GTAAGAAGGC TCCCTGCCTT CTATTATTCC TTCTTATCCA TCTCTATGGA

GTAAGAAGGC TCCCTGCCTT CTATTATTCC TTCTTATCCA TCTCTATGGA

GTAAGAAGGC TCCCTGTCTT CTATTATTCC TTCTTATCCA TCTCTATGGA

TAATTACCCT TTACAGGGGG TCATAGGGGG ATCCGTCCCC ATATCCTACG

TAATTACCCC TAATAGGGGG TCATAGGGGG ATCCGTCCCC ATATCCTACG

TAATTACCCC TAATAGGGGG TCATAGGGGG ATCCGTCCCC ATATCCTACG

TAATTACCCC TAATAGGGGG TCATAGGGGG ATCCGTCCCC ATATCCTACG

ATCCCTCCTT AGCAATAGAA GATCCAATTC ATTATATAAA TTATCATAAA

ATCCCTCCTT AGCAATAGAA GATCCAATAC ATTATATAAA TTATCATAAA

ATCCCTCCTT AGCAATAGAA GATCCAATTC ATTATATAAA TTATCATAAA

ATCCCTCCTT AGCAATAGAA GATCCAATAC ATTATATAAA TTATCATAAA

TAATAATTTA AAATAAAGCA GAATCTCCTA CTTCTTATTC GGTAACGTAG

TAATAATTTA AAATAAAGCA GAATCTCCTA CTTCTTATCC GGTAACGTAG

TAATAATTTA AAATAAAGCA GAATCTCCTA CTTCTTATCC GGTAACGTAG

TAATAATTTA AAATAAAGCA GAATCTCCTA CTTCTTATCC GGTAACGTAG

TCCTCCAGGG GAACTAACGA CACGCCTCCT CCTTTGGCTG ATTCTTAGAG

TCCTCCAGGG GAACTAACGA CACGCCTCCT CCTTTGGCTG ATTCTTAGAG

TCCTCCAGGG GAACTAACGA CACGCCTCCT CCTTTGGCTG ATTCTTAGAG

TCCTCCAGGG GAACTAACGA CACGCCTCCT CCTTTGGCTG ATTCTTAGAG

GCGGTCGGTA GCTGATCCAT CCCATCTCCA ACTTTGTATT GAGCATCAAT

GCGGTCGGTA GCTGATCCAT CCCATCTCCA ACTTTGTATT GAGCATCAAT

GCGGTCGGTA GCTGATCCAT CCCATCTCCA ACTTTGTATT GAGCATCAAT

GCGGTCGGTA GCTGATCCAT CCCATCTCCA ACTTTGTATT GAGCATCAAT

CAAAGTGAGA TAGGACCATC TTCATCGATC GATTAGAAAT CATCCCTAGT

CAAAGTGAGA TAGGACCATC TTCATCGATC GATTAGAAAT CATCCCTAGT

CAAAGTGAGA TAGGACCATC TTCATCGATC GATTAGAAAT CATCCCTAGT

CAAAGTGAGA TAGGACCATC TTCATCGATC GATTAGAAAT CATCCCTAGT

ATAGGATGAA TTGATTTCAA TCTCTGATGA GATGGCCTCT ACCATCTCTT

ATAGGATGAA TTGATTTCAA TCTCTGATGA GATGGCCTCT ACCATCTCTT

ATAGGATGAA TTGATTTCAA TCTCTGATGA GATGGCCTCT ACCATCTCTT

ATAGGATGAA TTGATTTCAA TCTCTGATGA GATGGCCTCT ACCATCTCTT

ACCACTTTAT CTGAGTGAGG CGTCACCTCT CCCTCCATCC CACACAGAGA

ACCACTTTAT CTGAGTGAGG CGTCACCTCT CCCTCCATCC CACACAGAGA

ACCACTTTAT CTGAGTGAGG CGTCACCTCT CCCTCCATCC CACACAGAGA

ACCACTTTAT CTGAGTGAGG CGTCACCTCT CCCTCCATCC CACACAGAGA

TGGAGAGGTA ATTCCTGAAT TACGCCTCAA ACTCAGTGAG TCGTTGGTCA

TGGAGAGGTA ATTCCTGAAT TACGCCTCAA ACTCAGTGAG TCGTTGGTCA

TGGAGAGGTA ATTCCTGAAT TACGCCTCAA ACTCAGTGAG TCGTTGGTCA

TGGAGAGGTA ATTCCTGAAT TACGCCTCAA ACTCAGTGAG TCGTTGGTCA

TCATCAACTT TAATTGAGCA TCAACTTAAA GTGAGATGAA ACCATCTCCA

TCATCAACTT TAATTGAGCA TCAACTTAAA GTGAGATGAA ACCATCTCCA

TCATCAACTT TAATTGAGCA TCAACTTAAA GTGAGATGAA ACCATCTCCA

TCATCAACTT TAATTGAGCA TCAACTTAAA GTGAGATGAA ACCATCTCCA

TCAATCGATT AGAAATCATC CCTAGTATAG GTTGATTTGA TTTCAATCTC

TCAATCGATT AGAAATCATC CCTAGTATAG GTTGATTTGA TTTCAATCTC

TCAATCGATT AGAAATCATC CCTAGTATAG GTTGATTTGA TTTCAATCTC

TCAATCGATT AGAAATCATC CCTAGTATAG GTTGATTTGA TTTCAATCTC

TGATAAGATG GAGAGGTTGA AATTTAGTAA ATTACGCCTT ACTCCCCCCT

TGATAAGATG GAGAGGTTGA AATTTAGTAA ATTACGCCTT ACTCCCCCCT

TGATAAGATG GAGAGGTTGA AATTTAGTAA ATTACGCCTT ACTCCCCCCT

TGATAAGATG GAGAGGTTGA AATTTAGTAA ATTACGCCTT ACTCCCCCCT

TGGGAGGTCG AAATTTGACC CTAATTTCCT GAGGAAATCC CATAGAATTT

TGGGAGGTCG AAATTTGACC CTAATTTCCT GAGGAAATCC CATAGAATTT

TGGGAGGTCG AAATTTGACC CTAATTTCCT GAGGAAATCC CATAGAATTT

TGGGAGGTCG AAATTTGACC CTAATTTCCT GAGGAAATCC CATAGAATTT

CCCTCCAAGA AGGGGAAAAT TAGGTCGTAA TTCCATCTCC AACTTTAATT

CCCTCCAAGA AGGGGAAAAT TAGGTCGTAA TTCCATCTCC AACTTTAATT

CCCTCCAAGA AGGGGAAAAT TAGGTCGTAA TTCCATCTCC AACTTTAATT

CCCTCCAAGA AGGGGAAAAT TAGGTCGTAA TTCCATCTCC AACTTTAATT

GAGCATCAAT TTAAAGTGAG ATGAAACCAT CTCCATCAAC TGATTAGAAA

GAGCATCAAT TTAAAGTGAG ATGAAACCAT CTCCATCAAC TGATTAGAAA

GAGCATCAAT TTAAAGTGAG ATGAAACCAT CTCCATCAAC TGATTAGAAA

GAGCATCAAT TTAAAGTGAG ATGAAACCAT CTCCATCAAC TGATTAGAAA

TCATCTCTAG TATATGAGAA TTGATTTCAA TCCTCAGGTG ATAGAGAGAT

TCATCTCTAG TATATGAGAA TTGATTTCAA TCCTCAGGTG ATAGAGAGAT

TCATCTCTAG TATATGAGAA TTGATTTCAA TCCTCAGGTG ATAGAGAGAT

TCATCTCTAG TATATGAGAA TTGATTTCAA TCCTCAGGTG ATAGAGAGAT

GGAACCTCTC TACACTCCTT AGCTGAGTGA GGCGTCACCT CTCCTCCCCA

GGAACCTCTC TACACTCCTT AGCTGAGTGA GGCGTCACCT CTCCTCCCCA

GGAACCTCTC TACACTCCTT AGCTGAGTGA GGCGTCACCT CTCCTCCCCA

GGAACCTCTC TACACTCCTT AGCTGAGTGA GGCGTCACCT CTCCTCCCCA

CCACACAGAT GGAGAGGTAA TTCTTGAATT ACGCCTCAGA CTCAGTGAGT

CCACACAGAT GGAGAGGTAA TTCTTGAATT ACGCCTCAGA CTCAGTGAGT

CCACACAGAT GGAGAGGTAA TTCTTGAATT ACGCCTCAGA CTCAGTGAGT

CCACACAGAT GGAGAGGTAA TTCTTGAATT ACGCCTCAGA CTCAGTGAGT

CGTTGGTCAT CATCAACTTT AATTGAGCAT CAATTTAAAG TGAGATGAAA

CGTTGGTCAT CATCAACTTT AATTGAGCAT CAATTTAAAG TGAGATGAAA

CGTTGGTCAT CATCAACTTT AATTGAGCAT CAATTTAAAG TGAGATGAAA

CGTTGGTCAT CATCAACTTT AATTGAGCAT CAACTTAAAG TGAGATGAAA

CCATCTCCAT CAATCGATTA GAAATCATCC CTAGTATAGG TTGATTTGAT

CCATCTCCAT CAATCGATTA GAAATCATCC CTAGTATAGG TTGATTTGAT

CCATCTCCAT CAATCGATTA GAAATCATCC CTAGTATAGG TTGATTTGAT

CCATCTCCAT CAATCGATTA GAAATCATCC CTAGTATAGG TTGATTTGAT

TTCAATCTCT GATAAGATGG AGAGGTCGTA ATCCCATCTC CAACTTTGTA

TTCAATCTCT GATAAGATGG AGAGGTCGTA ATCCCATCTC CAACTTTGTA

TTCAATCTCT GATAAGATGG AGAGGTCGTA ATCCCATCTC CAACTTTGTA

TTCAATCTCT GATAAGATGG AGAGGTCGTA ATCCCATCTC CAACTTTGTA

TTGAGCATCA ATCAAAGTGA GATAGGACCA TCTTCATCGA TCGATTAGAA

TTGAGCATCA ATCAAAGTGA GATAGGACCA TCTTCATCGA TCGATTAGAA

TTGAGCATCA ATCAAAGTGA GATAGGACCA TCTTCATCGA TCGATTAGAA

TTGAGCATCA ATCAAAGTGA GATAGGACCA TCTTCATCGA TCGATTAGAA

ATCATCCCTA GTATAGGATG AATTGATTTC AATCTCTGAT GAGATGGCCT

ATCATCCCTA GTATAGGATG AATTGATTTC AATCTCTGAT GAGATGGCCT

ATCATCCCTA GTATAGGATG AATTGATTTC AATCTCTGAT GAGATGGCCT

ATCATCCCTA GTATAGGATG AATTGATTTC AATCTCTGAT GAGATGGCCT

CTACCACCTW CCACCATCTT AGCTGAGTGA GGCGTCACCT CTCCTCCCCA

CTACCACCTT CCACCATCTT AGCTGAGTGA GGCGTCACCT CTCCTCCCCA

CTACCACCTT CCACCATCTT AGCTGAGTGA GGCGTCACCT CTCCTCCCCA

CTACCACCTT CCACCATCTT AGCTGAGTGA GGCGTCACCT CTCCTCCCCA

TACCACACAG AGATGGAGAG GTAATTCCTG AATTACGCCT CAAACTCAGT

TACCACACAG AGATGGAGAG GTAATTCTTG AATTACGCCT CAAACTCAGT

TMCCACACAG AGATGGAGAG GTAATTCCTG AATTACGCCT CAAACTCAGT

TACCACACAG AGATGGAGAG GTAATTCCTG AATTACGCCT CAGACTCAGT

GAGTCATCAG CCATCATCAA CTTTAATTGA GCATCAACTT AAAGTGAGAT

GAGTCATCAG CCATCATCAA CTTTAATTGA GCATCAACTT AAAGTGAGAT

GAGTCATCAG CCATCATCAA CTTTAATTGA GCATCAACTT AAAGTGAGAT

GAGTCATCAG CCATCATCAA CTTTAATTGA GCATCAACTT AAAGTGAGAT

GAAACCATCT TCATCAGCTG ATTAGAAATC ATCCCTAGTA TAGGTTGATT

GAAACCATCT TCATCAGCTG ATTAGAAATC ATCCCTAGTA TAGGTTGATT

GAAACCATCT TCATCAGCTG ATTAGAAATC ATCCCTAGTA TAGGTTGATT

GAAACCATCT TCATCAGCTG ATTAGAAATC ATCCCTAGTA TAGGTTGATT

TGATTTCAAT CTCTGATGAG ATGGAATCCA CCATCTATCA CCATATTAGC

TGATTTCAAT CTCTGATGAG ATGGAATCCA CCATCTATCA CCATATTAGC

TGATTTCAAT CTCTGATGAG ATGGAATCCA CCATCTATCA CCATATTAGC

TGATTTCAAT CTCTGATGAG ATGGAATCCA CCATCTATCA CCATATTAGC

TGAGTAGGCG TCACCTCTCC TCCCATACCA CAGAGATGGA GAGGTAATTC

TGAGTAGGCG TCACCTCTCC TCCCATACCA CAGAGATGGA GAGGTAATTC

TGAGTAGGCG TCACCTCTCC TCCCATACCA CAGAGATGGA GAGGTAATTC

TGAGTAGGCG TCACCTCTCC TCCCATACCA CAGAGATGGA GAGGTAATTC

TTGAATTACG CCTGTAACTC AGTGAATTAT ATCCCATCAT CAACTTTGTA

TTGAATTACG CCTGTAACTC AGTGAATTAT ATCCCATCAT CAACTTTGTA

TTGAATTACG CCTGTAACTC AGTGAATTAT ATCCCATCAT CAACTTTGTA

TTGAATTACG CCTGTAACTC AGTGAATTAT ATCCCATCAT CAACTTTGTA

TTGATGGTCA ATCAAAGTGA GATGGAACCA TCTTCATCAA TCGATTAGAA

TTGATGGTCA ATCAAAGTGA GATGGAACCA TCTTCATCAA TCGATTAGAA

TTGATGGTCA ATCAAAGTGA GATGGAACCA TCTTCATCAA TCGATTAGAA

TTGATGGTCA ATCAAAGTGA GATGGAACCA TCTTCATCAA TCGATTAGAA

ATCATCCCTA GTATAGGATG AATTGATTTC AATCTATGAT GAGATGGAAT

ATCATCCCTA GTATAGGATG AATTGATTTC AATCTATGAT GAGATGGAAT

ATCATCCCTA GTATAGGATG AATTGATTTC AATCTATGAT GAGATGGAAT

ATCATCCCTA GTATAGGATG AATTGATTTC AATCTATGAT GAGATGGAAT

CTACCACTCA TTTACATTCA TTATCTGAGT GAGGCGTCAC CTCTCCCTCC

CTACCATTCA TCTACACTCA TTATCTGAGT GAGGCGTCAC CTCTCCCTCC

CTACCATTCA TCTACACTCA TTATCTGAGT GAGGCGTCAC CTCTCCCTCC

CTACCACTCA TTTACATTCA TTATCTGAGT GAGGCGTCAC CTCTCCCTCC

ATTCCGCACA GAGATGGAGA GGTAATTCTT GAATTACGCC TCAAACTCAG

ATTCCGCACA GAGATGGAGA GGTAATTCTT GAATTACGCC TCAAACTCAG

ATTCCGCACA GAGATGGAGA GGTAATTCTT GAATTACGCC TCAGACTCAG

ATTCCGCACA GAGATGGAGA GGTAATTCTT GAATTACGCC TCAAACTCAG

TGAATTATAT TCCATCTCCA ACTTTAATTG AGCATCAATT TAAAGTGAGA

TGAATTATAT TCCATCTCCA ACTTTAATTG AGCATCAATT TAAAGTGAGA

TGAATTATAT TCCATCTCCA ACTTTAATTG AGCATCAATT TAAAGTGAGA

TGAATTATAT TCCATCTCCA ACTTTAATTG AGCATCAATT TAAAGTGAGA

TGAAACCATC TCCATCAACT GATTAGAAAT CATCTCTAGT ATATGAGAAT

TGAAACCATC TCCATCAACT GATTAGAAAT CATCTCTAGT ATATGAGAAT

TGAAACCATC TCCATCAACT GATTAGAAAT CATCTCTAGT ATATGAGAAT

TGAAACCATC TCCATCAACT GATTAGAAAT CATCTCTAGT ATATGAGAAT

TGATTTCAAT CCTCAGGTGA TAGAGAGATG ---------- ----------

TGATTTCAAT CCTCAGGTGA TAGAGAGAGA GGTGGCATCG TCTTAATTAA

TGATTTCAAT CCTCAGGTGA TAGAGAGATG G--------- ----------

TGATTTCAAT CCTCAGGTGA TAGAGAGA-- ---------- ----------

---------- ---------- ---------- ---------- ----------

CTAAAGCCAA AGCACTTAAA TAAAGTAAGA GGCATCCCGC TGTTACCGGG

---------- ---------- ---------- ---------- ----------

---------- ---------- ---------- ---------- ----------

---------- ---------- -------

AGAATTGGAA TTATAATCCT AGTTAAA

---------- ---------- -------

---------- ---------- -------
