# Supplementary material for: Fragmented mitochondrial genomes in two suborders of parasitic lice of eutherian mammals (Anoplura and Rhynchophthirina, Insecta)
Source: Sci Rep. 2015 Nov 30;5:17389. doi: 10.1038/srep17389 (PMC4663631; doi:10.1038/srep17389)
Supplement: Supplementary Dataset 2 [file srep17389-s3.doc]

6 2866

NCR(atp8-atp6-P-cox3) ----------------------------------------------------------------------------GCATTGTGGTTTTAGTTGCCCATGAGTCACGTCATGGAGGCTGTGTGGAGGTTTTTAGGCCT-TAAAAGGGTTAAATTTGGCCTTAATCTTCACTATAGCCTAATTTTTGAGTGACTTTGCAACAGAAAATATGTTATTNNNNNNNNNNNNNNNNNNNNNNNNNNNNNNNNNNNNNNNNNNNNNNNNNNNNNNNNNNNNNNNNNNNNNNNNNNNNNNNNNNNNNNNNNNNNNNNNNNNNNNNNNNNNNNNNNNNNNNNNNNNNNNNNNNNNNNNNNNNNNNNNNNNNNNNNNNNNNNNNNNNNNNNNNNNNNNNNNNNNNNNNNNNNNNNNNNNNNNNNNNNNNNNNNNNNNNNNNNNNNNNNNNNNNNNNNNNNNNNNNNNNNNNNNNNNNNNNNNNNNNNNNNNNNNNNNNNNNNNNNNNNNNNNNNNNNNNNNNNNNNNNNNNNNNNNNNNNNNNNNNNNNNNNNNNNNNNNNNNNNNNNNN-NNNNNNNNNNNNNNNNNNNNNNNNNTTA----------TTCATTCTTATCCATCTCTATGGATAATTACCCTTAACAGGGGGTCATAGGGGGATCCGTCCCCATATCCTACGATCCCTCCTTAGCAATAGAAGATCCAATTCATTATATAAATTATCATAAATAATAATTTAAAATAAAGCAGAATCTCCTACT-CTTATCCGGTAACGTAGTCCTCCAGGGGAACTAACGACACGCCTCCTCCTTTGGCTGATTCTTAGAGGCGGTCGGTAGCTGATCCATCCCATCTCCAACTTTGTATTGAGCATCAATCAAAGTGAGATAGGACCATCTTCATCGATCGATTAGAAATCATCCCTAGTATAGGATGAATTGATTTCAATCTCTGATGAGATGGCCTCTACNNNNNNNNNNNNNNNNNNNNNNNNNNNNNNNNNNNNNNNN-NNNNNNNNNNNNNNNNNNNNNNNNNNNNNNNNNNNNNNNNNNNNNNNNNNNNNNNNNNNNNNNNNNNNNNNNNNNNNNNNNNNNNNNNNNNNNNNNNNNNNNNNNNNNNNNNNNNNNNNNNNNNNNNNNNNNNNNNNNNNNNNNNNNNNNNNNNNNNNNNNNNNNNNNNNNNNNNNNNNNNNNNNNNNNNNNNNNNNNNNNNNNNNNNNNNNNNNNNNNNNNNNNNNNNNNNNNNNNNNNNNNNNNNNNNNNNNNNNNNNNNNNNNNNNNNNNNNNNNNNNNNNNNNNNNNNNNNNNNNNNNNNNNNNNNNNNNNNNNNNNNNNNNNNNNNNNNNNNNNNNNNNNNNNNATCTCCAACTTTAATTGAGCATCAATTTAAAGTGAGATGAAACCATCTCCATCAACTGATTAGAAATCATCTCTAGTATATGAGAATTGATTTCAATCCTCAGGTGATAGAGAGATGGAACCTNNNNNNNNNNNNNNNNNNNNNNNNNNNNNNNNNNNNNNNNNNNNNNNNNNNNNNNNNNNNNNNNNNNNNNNNNNNNNNNNNNNNNNNNNNNNNNNNNNNNNNNNNNNNNNNNNNNNNNNNNNNN-NNNNNNNNNNNNNNNNNNNNNNNNNNNNNNNNNNNNNNNNNNNNNNNNNNNNNNNNNNNNNNNNNNNNNNNNNNNNNNNNNNNNNNNNNNNNNNNNNNNNNNNNNNNNNNNNNNNNNNNNNNNNNNNNNNNNNNNNNNNNNNNNNNNNNNNNNNNNNNNNNNNNNNNNNNNNNNNNNNNNNNNNNNNNNNNNNNNNNNNNNNNNNNNNNNNNNNNNNNNNNNNNNNNNNNNNNNNNNNNNNNNNNNNNNNNNNNNNNNNNNNNNNNNNNNNNNNNNNNNNNNNNNNNNNNNNNNNNNNNNNNNNNNNNNNNNNNNNNNNNNNNNNNNNNNNNNNNNNNNNNNNNNNNNNNNNNNNNNNNNNNNNNNNNNNNNNNNNNNNNNNNNNNNNNNNNNNNNNNNNNNNNNNNNNNNNNNNNNNNNNNNNNNNNNNNNNNNNNNNNNNNNNNNNNNNNNNNNNNNNNNNNNNNNNNNNNNNNNNNNNNNNNNNNNNNNNNNNNNNNNNNNNNNNNNNNNNNNNNNNNNNNNNNNNNNNNNNNNNNNNNNNNNNNNNNNNNNNNNNNNNNNNNNNNNNNNNNNNNNNNNNNNNNNNNNNNNNNNNNNNNNNNNNNNNNNNAATTACGCCTCAAACTCAGTGAGTCGTTGGTCATCATCAACTTTAATTGAGCATCAACTTAAAGTGAGATGAAACCATCTCCATCAATCGATTAGAAATCATCCCTAGTATAGGTTGATTTGATTTCAATCTCTGATAAGATGGAGAGGTTGAAATTTAGTAAATTACGCCTTACTCCCCCCTTGGGAGGTCGAAATTTGACCCTAATTTCCTGAGGAAATCCCATAGAATTTCCCTCCAAGAAGGGGAAAATTAGGTCGTAATTCCATCTCCAACTTTAATTGAGCATCAATTTAAAGTGAGATGAAACCATCTCCATCAACTGATTAGAAATCATCTCTAGTATATGAGAATTGATTTCAATCCTCAGGTGATAGAGAGATGGAACCT---TG-GCCCTTTGATCACATTCAAG-GCCG------------------------------------------------------------------------------------------------------------------------------------------------------------------------------------------------------------------------------------

NCR(cob-A-W-F-nad6) -------------------------------------------------------------------------------------------TTGCCCATGAGTCACGTCATGGAGGCTGTGTGGAGGTTTTTAGGCCT-TAAAAGGGTTAAATTTGGCCTTAATCTTCACTATAGCCTAATTTTTGAGTGACTTTGCAACAGAAAATATGTTATTNNNNNNNNNNNNNNNNNNNNNNNNNNNNNNNNNNNNNNNNNNNNNNNNNNNNNNNNNNNNNNNNNNNNNNNNNNNNNNNNNNNNNNNNNNNNNNNNNNNNNNNNNNNNNNNNNNNNNNNNNNNNNNNNNNNNNNNNNNNNNNNNNNNNNNNNNNNNNNNNNNNNNNNNNNNNNNNNNNNNNNNNNNNNNNNNNNNNNNNNNNNNNNNNNNNNNNNNNNNNNNNNNNNNNNNNNNNNNNNNNNNNNNNNNNNNNNNNNNNNNNNNNNNNNNNNNNNNNNNNNNNNNNNNNNNNNNNNNNNNNNNNNNNNNNNNNNNNNNNNNNNNNNNNNNNNNNNNNNNNNNNNNN-NNNNNNNNNNNNNNNTATTCCTTCTT---------------------ATCCATCTCTATGGATAATTACCCTTTACAGGGGGTCATAGGGGGATCCGTCCCCATATCCTACGATCCCTCCTTAGCAATAGAAGATCCAATTCATTATATAAATTATCATAAATAATAATTTAAAATAAAGCAGAATCTCCTACTTCTTATCCGGTAACGTAGTCCTCCAGGGGAACTAACGACACGCCTCCTCCTTTGGCTGATTCTTAGAGGCGGTCGGTAGCTGATCCATCCCATCTCCAACTTTGTATTGAGCATCAATCAAAGTGAGATAGGACCATCTTCATCGATCGATTAGAAATCATCCCTAGTATAGGATGAATTGATTTCAATCTCTGATGAGATGGCCTCTACCACCTTCTACCATCTTAGCTGAGTGAGGCGTCACCTCTCC-TCCCATACCACACAGAGATGGAGAGGTAATTCCTGAATTACGCCTCAAACTCAGTGAGTCATCAGCCATCATCAACTTTAATTGAGCATCAACTTAAAGTGAGATGAAACCATCTTCATCAGCTGATTAGAAATCATCCCTAGTATAGGTTGATTTGATTTCAATCTCTGATGAGATGGANNNNNNNNNNNNNNNNNNNNNNNNNNNNNNNNNNNNNNNNNNNNNNNNNNNNNNNNNNNNNNNNNNNNNNNNNNNNNNNNNNNNNNNNNNNNNNNNNNNNNNNNNNNNNNNNNNNNNNNNNNNNCATCTCCACTTTAATTGAGCATCAATTTAAAGTGAGATGAAACCA---------------------------------------------TCTCCATCAACTGATTAGAAATCATCTCTAGTATATGAGAATTGATTTCAATCCTCAGGTGATAGAGAGATGGAACCTNNNNNNNNNNNNNNNNNNNNNNNNNNNNNNNNNNNNNNNNNNNNNNNNNNNNNNNNNNNNNNNNNNNNNNNNNNNNNNNNNNNNNNNNNNNNNNNNNNNNNNNNNNNNNNNNNNNNNNNNNNNN-NNNNNNNNNNNNNNNNNNNNNNNNNNNNNNNNNNNNNNNNNNNNNNNNNNNNNNNNNNNNNNNNNNNNNNNNNNNNNNNNNNNNNNNNNNNNNNNNNNNNNNNNNNNNNNNNNNNNNNNNNNNNNNNNNNNNNNNNNNNNNNNNNNNNNNNNNNNNNNNNNNNNNNNNNNNNNNNNNNNNNNNNNNNNNNNNNNNNNNNNNNNNNNNNNNNNNNNNNNNNNNNNNNNNNNNNNNNNNNNNNNNNNNNNNNNNNNNNNNNNNNNNNNNNNNNNNNNNNNNNNNNNNNNNNNNNNNNNNNNNNNNNNNNNNNNNNNNNNNNNNNNNNNNNNNNNNNNNNNNNNNNNNNNNNNNNNNNNNNNNNNNNNNNNNNNNNNNNNNNNNNNNNNNNNNNNNNNNNNNNNNNNNNNNNNNNNNNNNNNNNNNNNNNNNNNNNNNNNNNNNNNNNNNNNNNNNNNNNNNNNNNNNNNNNNNNNNNNNNNNNNNNNNNNNNNNNNNNNNNNNNNNNNNNNNNNNNNNNNNNNNNNNNNNNNNNNNNNNNNNNNNNNNNNNNNNNNNNNNNNNNNNNNNNNNNNNNNNNNNNNTAATTCCTGAAGTACGCCTCAAACTCA-----------------------GTGAGTCGTTGGTCATCATCAACTTTAATTGAGCATCAACTTAAAGTGAGATGAAACCATCTCCATCAATCGATTAGAAATCATCCCTAGTATAGGTTGATTTGATTTCAATCTCTGATAAGATGGAGAGGTTGAAATTTAGTAAATTACGCCTTACTCCCCCCTTGGGAGGTCGAAATTTGACCCTAATTTCCTGAGGAAATCCCATAGAATTTCCCTCCAAGAAGGGGAAAATTAGGTCGTAATTCCATCTCCAACTTTAATTGAGCATCAATTTAAAGTGAGATGAAACCATCTCCATCAACTGATTAGAAATCATCTCTAGTATATGAGAATTGATTTCAATCCTCAGGTGATAGAGAGATGGAACCT---TG-GCCCTTTGATCACATTCAAG-GCCGTTGCCTCAA---------------------------------------------------------------------------------------------------------------------------------------------------------------------------------------------------------------------------

NCR(Y-cox2-E) --------------------------------------------------------------------ATTTCTTAGCATTGTGGTTTTAGTTGCCCATGAGTCACGTCATGGAGGCTGTGTGGAGGTTTTTAGGCCT-TAAAAGGGTTAAATTTGGCCTTAATCTTCACTTTAGCCTAATTTTTGAGTGACTTTGCAACAGAAAATATGTTATTNNNNNNNNNNNNNNNNNNNNNNNNNNNNNNNNNNNNNNNNNNNNNNNNNNNNNNNNNNNNNNNNNNNNNNNNNNNNNNNNNNNNNNNNNNNNNNNNNNNNNNNNNNNNNNNNNNNNNNNNNNNNNNNNNNNNNNNNNNNNNNNNNNNNNNNNNNNNNNNNNNNNNNNNNNNNTATATAGATAGAGTAATAGAATAAGAAAAGCAGTTTATTACCATATACCCAGAGGGCCTACCGGGGATTAATTAATTGATTAATAGGGGGGTAAAAAAAAAGAACAAACATGCATGGGTGGGAGACCCCTGTATACTTGTTAGTCGGCAGCCGACTAGCAAGAAGAAAGACTCC-TGTCTTTTATTCAT--------TCTT---------------------ATCCATCTCTATGGATAATTACCCCTAACAGGGGGTCATAGGGGGATCCGTCCCCATATCCTACGATCCCTCCTTAGCAATAGAAGATCCAATTCATTATATAAATTATCATAAATAATAATTTAAAATAAAGCAGAATCTCCTACTTCTTATCCGGTAACGTAGTCCTCCAGGGGAACTAACGACACGCCTCCTCCTTTGGCTGATTCTTAGAGGCGGTCGGTAGCTGATCCATCCCATCTCCAACTTTGTATTGAGCATCAATCAAAGTGAGATAGGACCATCTTCATCGATCGATTAGAAATCATCCCTAGTATAGGATGAATTGATTTCAATCTCTGATGAGATGGCCTCTACCATCTTCCACCA-CTTAACTGAGTGAGGCGTCACCTCTCC-NNNNNNNNNNNNNNNNNNNNNNNNNNNNNNNNNNNNNNNNNNNNNNNNNNNNNNNNNNNNNNNNNNNNNNNNNNNNNNNNNNNNNNNNNNNNNNNNNNNNNNNNNNNNNNNNNNNNNNNNNNNNNNNNNNNNNNNNNNNNNNNNNNNNNNNNNNNNNNNNNNNNNNNNNNNNNNNNNNNNNNNNNNNNNNNNNNNNNNNNNNNNNNNNNNNNNNNNNNNNNNNNNNNNNNNNNNNNNNNNNNNNNNNNNNNNNNNNNNNNNNNNNNNNNNNNNNNNNNNNNNNNNNNNNNNNNNNNNNNNNNNNNNNNNNNN-TTCATCTCCACTTTAATTGAGCATCAATTTAAAGTGA--------------------------------------------------------GAT--GAAACC---------------------ATCTCCA---TCAACTGATT-AGAAATC--ATCTCTAGTATATGAGAATTGATTTCAATCCTCAGGTGATAGAGAGATGGAACCTCTCTACACTCCTTATCTGAGTGAGGCGTCACCTCTCCTCCCCACCACACAGATGGAGAGGTAATTCTTGAATT-ACGCCTCAGACTCAGTGAGTCGTTGGTCATCATCAACTTTGTATTGATGGTCAATCAAAGTGAGATGAAACCATCTCCATCAATCGATTAGAAATCATCCCTAGTATAGGTTGATTTGATTTCAATCTCTGATAAGATGGAGAGGTNNNNNNNNNNNNNNNNNNNNNNNNNNNNNNNNNNNNNNNNNNNNNNNNNNNNNNNNNNNNNNNNNNNNNNNNNNNNNNNNNNNNNNNNNNNNNNNNNNNNNNNNNNNNNNNNNNNNNNNNNNNNNNNNNNNNNNNNNNNNNNNNNNNNNNNNNNNNNNNNNNNNNNNNNNNNNNNNNNNNNNNNNNNNNNNNNNNNNNNNNNNNNNNNNNNNNNNNNNNNNNNNNNNNNNNNNNNNNNNNNNNNNNNNNNNNNNNNNNNNNNNNNNNNNNNNNNNNNNNNNNNNNNNNNNNNNNNNNNNNNNNNNNNNNNNNNNNNNNNNNNNNNNNNNNNNNNNNNNNNNNNNNNNNNNNNNNNNNNNNNNNNNNNNNNNNNNNNNNNNNNNNNNNNNNNNNNNNNNNNNNNNNNNNNNNNNNNNNNNNNNNNNNNNNNNNNNNNNNNNNNNNNNNNNNNNNNNNNNNNNNNNNN-------------------NNNNNNNNNNNNNNNNNNNNNNNNNNNNNNNNNNNNNNNNNNNNNNNNNNNNNNNNNNNNNNNNNNNNNNNNNNNNNNNNNNNNNNNNNNNNNNNNNNNNNNNNNNNNNNNNNNNNNNNNNNNNNNNNNNNNNNNNNNNNNNNNNNNNNNNNNNNNNNNNNNNNNNNNNNNNNNNNNNNNNNNNNNNNNNNNNNNNNNNNNNNNNNNNNNNNNNNNNNNNNNNNNNNNNNNNNNNNNNNNNNNNATTCCATCTCCAACTTTAATTGAGCATCAATTTAAAGTGAGATGAAACCATCTCCATCAACTGATTAGAAATCATCTCTAGTATATGAGAATTGATTTCAATCCTCAGGTGATAGAGAGATGGAACCTCTCTACACTCCTT-ATCTGAGTGAGGCGTCACCTCTCCTCCCCACCACACAGATGGAGAGGTAATTCTTGAATTACGCCTCAGACTCAGTGAGTCGTTGGTCATCATCAACTTTGTATTGATGGTCAATCAAAGTGAGATGAAACCATCTCCATCAATCGATTAGAAATCATCCCTAGTATAGGTTGATTTGATTTCAATCTCTGATGAGATGGAGAGGTTGTGTCACCGCCACTTACAGGCGGGTATCTACAATATCT

NCR(S2-R-nad4L-M-G-nad3) --------------------------------------------------------------GTGATGCTTCAAGAGCATTGTTATTTTAGTTGCCCATGAGTCACGTCATGGAGGCTGTGTGGAGGTTTTTAGGCCT-TAAAAGGGTTAAATTTGGCCTTAATCTTCACTTTAGCCTAATTTTTGAGTGACTTTGCAACAGAAAATATGTTATTNNNNNNNNNNNNNNNNNNNNNNNNNNNNNNNNNNNNNNNNNNNNNNNNNNNNNNNNNNNNNNNNNNNNNNNNNNNNNNNNNNNNNNNNNNNNNNNNNNNNNNNNNNNNNNNNNNNNNNNNNNNNNNNNNNNNNNNNNNNNNNNNNNNNNNNNNNNNNNNNNNNNNNNNNNNNNNNNNNN-NNNNNNNNNNNNNNNNNNNNNNNNNNN-NNNNNNNNNNNNNNNNNNNNNNNNNNNNNNNNNNNNNNNNNNNNN--NNNN--------------------NNNNNNNNNNNNNNNNNNNNNNNNNNNNNNNNNNNNNNNNNNNNNNNNNNNNNNNNNNNNNNNNNNNNNNNNNNNNNNNNNNNNNNNNNNNNNNNCCTTTCTATTATTCATTCTTATCCATCTCTATGGATAATTACCCTTAATAGGGGGTCATAGGGGGATCCGTCCCCATATCCTACGATCCCTCCTTAGCAATAGAAGATCCAATTCATTATATAAATTATCATAAATAATAATTTAAAATAAAGCAGAATCTCCTACTTCTTATCCGGTAACGTAGTCCTCCAGGGGAACTAACGACACGCCTCCTCCTTTGGCTGATTCTTAGAGGCGGTCGGTAGCTGATCCATCCCATCTCCAACTTTGTATTGAGCATCAATCAAAGTGAGATAGGACCATCTTCATCGATCGATTAGAAATCATCCCTAGTATAGGATGAATTGATTTCAATCTCTGATGAGATGGCCTCTACCACCTTCCACCATCTTAACTGAGTGAGGCGTCACCTCTCCTCCCCATACCACACAGAGATGGAGAGGTAATTCCTGAATTACGCCTCAAACTCAGTGAGTCATCAGCCATCATCAACTTTAATTGAGCATCAACTTAAAGTGAGATGAAACCATCTTCATCAGCTGATTAGAAATCATCCCTAGTATAGGTTGATTTGATTTCAATCTCTGATGAGATGGANNNNNNNNNNNNNNNNNNNNNNNNNNNNNNNNNNNNNNNNNNNNNNNNNNNNNNNNNNNNNNNNNNNNNNNNNNNNNNNNNNNNNNNNNNNNNNNNNNNNNNNNNNNNNNNNNNNNNNNT-CCATCTCCAAC------------TTTAATTGAGCATCAATTTAAAGTGA--------------------------------------------------------GAT--GAAACC---------------------ATCTCCA---TCAACTGATT-AGAAATC--ATCTCTAGTATATGAGAATTGATTTCAATCCTCAGGTGATAGAGAGATGGAACCTCTCTACACTCCTTATCTGAGTGAGGCGTCACCTCTCCTCCCCACCACACAGATGGAGAGGTAATTCTTGAATT-ACGCCTCAGACTCAGTGAGTCGTTGGTCATCATCAACTTTGTATTGATGGTCAATCAAAGTGAGATGGAAATAAAAATAGCTA---AGTA------GTCTAA-ATATA--TTGATTTAGGGCGGATGCC--------TTGAAAGCATCAGGTAGTGACTAACTTTCACTTTTAGCTTAGAGCGGGATATTATCCATNNNNNNNNNNNNNNNNNNNNNNNNNNNNNNNNNNNNNNNNNNNNNNNNNNNNNNNNNNNNNNNNNNNNNNNNNNNNNNNNNNNNNNNNNNNNNNNNNNNNNNNNNNNNNNNNNNNNNNNNNNNNNNNNNNNNNNNNNNNNNNNNNNNNNNNNNNNNNNNNNNNNNNNNNNNNNNNNNNNNNNNNNNNNNNNNNNNNNNNNNNNNNNNNNNNNNNNN-----NNN--NNNNNNNNN--NNNNN--NNN----------NNNNNNNNNNNNNNNNNNNNNNNNNNNNNNNNNNNNNNNNNNNNNNNNNNNNNGGCCTCTACCATCTCTTACCACTTTATCTGAGTGAGGCGTCACCTCTCCCTCCATCCCACACAGAGATGGAGAGGTAATTCCTGAATTACGCCTCAAACTCA-----------------------GTGAGTCGTTGGTCATCATCAACTTTAATTGAGCATCAACTTAAAGTGAGATGAAACCATCTCCATCAATCGATTAGAAATCATCCCTAGTATAGGTTGATTTGATTTCAATCTCTGATAAGATGGAGAGGTTGAAATTTAGTAAATTACGCCTTACTCCCCCCTTGGGAGGTCGAAATTTGACCCTAATTTCCTGAGGAAATCCCATAGAATTTCCCTCCAAGAAGGGGAAAATTAGGTCGTAATTCCATCTCCAACTTTAATTGAGCATCAATTTAAAGTGAGATGAAACCATCTCCATCAACTGATTAGAAATCATCTCTAGTATATGAGAATTGATTTCAATCCTCAGGTGATAGAGAGATGGAACCTCTCTACACTCCTT-ATCTGAGTGAGGCGTCACCTCTCCTCCCCACCACACAGATGGAGAGGTAATTCTTGAATTACGCCTCAGACTCAGTGAGTCGTTGGTCATCATCAACTTTAATTGAGCATCAACTTAAAGTGAGATGGAAATAAAAAT-----------------------------------------------------------------------------------------------------------

NCR(L2-rrnL) --------------------------------------------------------------CTGATGCTTCAAGAGCATTGTGGTTTTAGTTGCCCATGAGTCACGTCATGGAGGCTGTGTGGAGGTTTTTAGGCCT-TAAAAGGGTTAAATTTGGCCTTAATCTTCACTATAGCCTAATTTTTGAGTGACTTTGCAACAGAAAATATGTTATTTATGATATATCGATTGTAAAATCCTTCAGGATTATTTTATATGAATTTTACCCAGGGGGGAACCATCAGGGGGGATGCATACTATACCCAGATACCCAGATACCGTATGTACCCCAGCCGGGAGGGAGTAATACTAGATAGAGAGAGATTTATTATATAGATTACTTGAATAAAGAAAG-CAGTGTATTTCCAGTATACCCATAGTG-ATAACCGGGGAGTAACTATAATTGATGTAGAGGGGGAAAGAAAAG--AGTA--------------------ACATACAGGGGTCGGAGACCCCTGTATACCTGCTAGTCGGCTGCCGACTAGCATGTAAGAAGGCTCCCTGCCTTCTATTATTCCTTCTTATCCATC--------------------------TCTATGGATAATTACCCCTAACAGGGGGTCATAGGGGGATCCGTCCCCATATCCTACGATCCCTCCTTAGCAATAGAAGATCCAATTCATTATATAAATTATCATAAATAATAATTTAAAATAAAGCAGAATCTCCTACTTCTTATCCGGTAACGTAGTCCTCCAGGGGAACTAACGACACGCCTCCTCCTTTGGCTGATTCTTAGAGGCGGTCGGTAGCTGATCCATCCCATCTCCAACTTTGTATTGAGCATCAATCAAAGTGAGATAGGACCATCTTCATCGATCGATTAGAAATCATCCCTAGTATAGGATGAATTGATTTCAATCTCTGATGAGATGGCCTCTACCATCTTCCACCATCTTATCTGAGTGAGGCGTCACCTCTCCTCCCCATACCACACAGAGATGGAGAGGTAATTCCTGAATTACGCCTCAAACTCAGTGAGTCATCAGCCATCATCAACTTTAATTGAGCATCAACTTAAAGTGAGATGAAACCATCTTCATCAGCTGATTAGAAATCATCCCTAGTATAGGTTGATTTGATTTCAATCTCTGATGAGATGGNNNNNNNNNNNNNNNNNNNNNNNNNNNNNNNNNNNNNNNNNNNNNNNNNNNNNNNNNNNNNNNNNNNNNNNNNNNNNNNNNNNNNNNNNNNNNNNNNNNNNNNNNNNNNNNNNNNNNNNNN-NNNNNNNNNNN------------NNNNAT-GAGCATCAATTTAAAGTGA--------------------------------------------------------GAT--GAAACC---------------------ATCTCCA---TCAACTGATT-AGAAATC--ATCTCTAGTATATGAGAATTGATTTCAATCCTCAGGTGATAGAGAGATGGAACCT------A--------------GAAG-GTTG-----------------GCAGATTAAGTG-----CGTTGAATTTAGAATTCA-ACT--ATGAG--GTTAG--ATGGTTA--TTCGCCTC--------ACC----T----T--------------CTA---ATTA------ATTCTT-GTAAA--TTGTTTTAG------------------TTGA----ATTACACA----TTAATTATTACT--GA---TAAAG----ACATAA-----ATGATAAGTCAGTCTTTGCTGAAGTAACAATTGAGATTGAAAAGGTTTCAATAAATTAGAATAAACTGTGAAGGATTGTCACAACATAAGACAAGTAAGGGTTGTTCCTGTACCTTTTGTATCAGGGTTTGAGGAATAAAATAGTTCATTTACAACTTCCCGAAAGGGGGAGATCTTTGATGGGAGTCAAGGTTGTTGTCATTACCATCTGCAATC-----TCA--TTAAAGTGG--TGAAA--ATC----------GATTAGAAATCATCCCTAGTATAGGATGAATTGATTTCAATCTCTGATGAGATGGCCTATACCATCTCTTACCACTTTATCTGAGTGAGGCGTCACCTCTCCCTCCATCCCACACAGAGATGGAGAGGTAATTCCTGAATTACGCCTCAAACTCA-----------------------GTGAGTCGTTGGTCATCATCAACTTTAATTGAGCATCAACTTAAAGTGAGATGAAACCATCTCCATCAATCGATTAGAAATCATCCCTAGTATAGGTTGATTTGATTTCAATCTCTGATAAGATGGAGAGGTTGAAATTTAGTAAATTACGCCTTACTCCCCCCTTGGGAGGTCGAAATTTGACCCTAATTTCCTGAGGAAATCCCATAGAATTTCCCTCCAAGAAGGGGAAAATTAGGTCGTAATTCCATCTCCAACTTTAATTGAGCATCAATTTAAAGTGAGATGAAACCATCTCCATCAACTGATTAGAAATCATCTCTAGTATATGAGAATTGATTTCAATCCTCAGGTGATAGAGAGATGGAACCT-------------------------------------------------------------------------------------------------------------------------------------------------------------------------------------------------------------------------------------------------------------------

NCR(L2-rrnS) ATGTTGTACTTGAATCATCAGTGATTCCTTGTTTGAGGGCATCTGCCATTTATAATTATTGGCT-ATCCTTTAAGC-CATTGTTATTTTAGTTGCCCGAGAGTCACGTTGGCGACCCATTGTGGGGGTTTTTAGCCAGGTAAAAGGGT-GATTTTGCCTTCAAGTTCAACAATGTCACTTTTTTTGAGTGACTTTGCAACAGAAAATATGTTATTNNNNNNNNNNNNNNNNNNNNNNNNNNNNNNNNNNNNNNNNNNNNNNNNNNNNNNNNNNNNNNNNNNNNNNNNNNNNNNNNNNNNNNNNNNNNNNNNNNNNNNNNNNNNNNNNNNNNNNNNNNNNNNNNNNNNNNNNNNNNNNNNNNNNNNNNNNNNNNNNNNNNNNNNNNNNNNNNNNN-NNNNNNNNNNNNNNNNNNNNNNNNNNN-NNNNNNNNNNNNNNNNNNNNNNNNNNNNNNNNNNNNNNNNNNNNNTAAATAGAGGGGGAAAGAAAAAAAAAACATACAGGGGTCGGAGACCCCTGTATACCTGCTAGTCGGCAGCCGACTAGCATGTAAGAAGGCTCCCTGCCTTCTATTATTCCTTCTTATCCATC--------------------------TCTATGGATAATTACCCCTAATAGGGGGTCATAGGGGGATCCGTCCCCATATCCTACGATCCCTCCTTAGCAATAGAAGATCCAATTCATTATATAAATTATCATAAATAATAATTTAAAATAAAGCAGAATCTCCTACTTCTTATCCGGTAACGTAGTCCTCCAGGGGAACTAACGACACGCCTCCTCCTTTGGCTGATTCTTAGAGGCGGTCGGTAGCTGATCCATCCCATCTCCAACTTTGTATTGAGCATCAATCAAAGTGAGATAGGACCATCTTCATCGATCGATTAGAAATCATCCCTAGTATAGGATGAATTGATTTCAATCTCTGATGAGATGGCCTCTACCATCTCTTACCACTTTATCTGAGTGAGGCGTCACCTCTCC-CTCCATCCCACACAGAGATGGAGAGGTAATTCCTGAATTACGCCTCAAACTCAGTGAGTNNNNNNNNNNNNNNNNNNNNNNNNNNNNNNNNNNNNNNNNNNNNNNNNNNNNNNNNNNNNNNNNNNNNNNNNNNNNNNNNNNNNNNNNNNNNNNNNNNNNNNNNNNNNNNNNNNNNNNNNNNNNNNNNNNNNNNNNNNNNNNNNNNNNNNNNNNNNNNNNNNNNNNNNNNNNNNNNNNNNNNNNNNNNNNNNNNNNNNNNNNNNNNNNNNNNNNNNNNNNNNNNNNNNNNNNNNNNNNNATTCCATCTCCAAC------------TTTAATTGAGCATCAATTTAAAGTGA--------------------------------------------------------GAT--GAAACC---------------------ATCTCCA---TCAACTGATT-AGAAATC--ATCTCTAGTATATGAGAATTGATTTCAATCCTCAGGTGATAGAGAGATGGAACCTCTCTACACTCCTTATCTGAGTGAGGCGTCACCTCTCCTCCCCACCACACAGATGGAGAGGTAATTCTTGAATT-ACGCCTCAGACTCAGTGAGTCGTTGGTCATCATCAACTTTGTATTGATGGTCAATCAAAGTGAGATGAAACCATCTCCATCAATCGATTAGAAATCATCCCTAGTATAGGTTGATTTGATTTCAAT-CT--------CTGATGAGATGGNNNNNNNNNNNNNNNNNNNNNNNNNNNNNNNNNNNNNNNNNNNNNNNNNNNNNNNNNNNNNNNNNNNNNNNNNNNNNNNNNNNNNNNNNNNNNNNNNNNNNNNNNNNNNNNNNNNNNNNNNNNNNNNNNNNNNNNNNNNNNNNNNNNNNNNNNNNNNNNNNNNNNNNNNNNNNNNNNNNNNNNNNNNNNNNNNNNNNNNNNNNNNNNNNNNNNNNNNNNNNNNNNNNNNNNNNNNNNNNNNNNNNCCATCATCAACTTTAATTGAGCATCAACTTAAAGTGAGATGAAACCATCTTCATCAGCTGATTAGAAATCATCCCTAGTATAGGTTGATTTGATTTCAATCTCTGATGAGATGGAATCCACCATCTATCACCATATTAGCTGAGT-AGGCGTCACCTCTCCTCCCATACCACACAGA--TGGAGAGGTAATTCCTGAATTACGCCTCAAACTCA-----------------------GTGAGTCGTTGGTCATCATCAACTTTAATTGAGCATCAACTTAAAGTGAGATGAAACCATCTCCATCAATCGATTAGAAATCATCCCTAGTATAGGTTGATTTGATTTCAATCTCTGATAAGATGGAGAGGTTGAAATTTAGTAAATTACGCCTTACTCCCCCCTTGGGAGGTCGAAATTTGACCCTAATTTCCTGAGGAAATCCCATAGAATTTCCCTCCAAGAAGGGGAAAATTAGGTCGTAATTCCATCTCCAACTTTAATTGAGCATCAATTTAAAGTGAGATGAAACCATCTCCATCAACTGATTAGAAATCATCTCTAGTATATGAGAATTGATTTCAATCCTCAGGTGATAGAGAGATGGAACCTCTCTACACTCCTT-ATCTGAGTGAGGCGTCACCTCTCCTCCCCACCACACAGATGGAGAGGTAATTCTTGAATTACGCCTCAGACTCAGTGAGTCGTTGGTCATCATCAACTTTGTATTGATGGTCAATCAAAGTGAGATGAAACCATCTCCATCAATCGATTAGAAATCATCCCTAGTATAGGTTGATTTGATTTCAATCTCTGATGAGATGGCATGATTAGCCT---------------------------------
