# Supplementary material for: Fragmented mitochondrial genomes in two suborders of parasitic lice of eutherian mammals (Anoplura and Rhynchophthirina, Insecta)
Source: Sci Rep. 2015 Nov 30;5:17389. doi: 10.1038/srep17389 (PMC4663631; doi:10.1038/srep17389)
Supplement: Supplementary Dataset 3 [file srep17389-s4.doc]

16 75

L1Hsapiens

ACTTTTAAAG GATAACAGCT ---ATCCATT GGTCTTAGGC CCCAAAAAT- TTTGGTGCAA CTCCAAATAA AAGTA

L1Ptroglod

.......... ........T. ---....... .......... .........- .......... .......... .....

L1Ggorilla

.......... .......... ---....... .........A .........- .......... .......... .....

L1Mmusculu

.......T.. .....T..TA ---....... .........A A.......C- C......... A......... .....

L1Clupus(w

.......T.. ....GG..TA ---....G.. .........A A.......A- ..G.TGCA.C T-...G.... .....

L1Sscrofa

.......... .......... ---....G.. .........A A.......A- ..G.TGCA.C T-........ .....

L1Ecaballu

.......... ....GG.... ---....G.. .........A A.......A- ..G.TGCA.C T-........ .....

L1Lafrican

.......... .G...G.... ---.C..... .......... A.......A- C.G.TGCA.C T-...G.... .....

L2Hsapiens

GT.AAG.TG. C.G.G.CCGG TAATCG...A AAA....AAA .TTT.C.G.C AGA...T... T...TCT.CT T.AC.

L2Ptroglod

GT.AAG.TG. C.G.G.CCGG TAAT.G...A AAA....AAA .TTT.C...C AGA...T... T...TCT.CT TGAC.

L2Ggorilla

GT.AAG.TG. C.G.G.CCGG TAATCG...A AAA....AAA .TTT.T.G.C AGA...T... T...TCT.CT T.AC.

L2Mmusculu

.T.AGGGTG. C.G.G.CAGG AAAT.G.G.A A.A....AAA ..TTGTTCCC AGA...T... A...TCTCCC T.A..

L2Clupus(w

GT.AGGGTG. C.G.G.CCGG TAAC.G.G.A AAA....AA. .TTT.CT..C AGA...T... T...TCTCCC T.AC.

L2Sscrofa

.T.AGGGTG. C.G.GACCGG TAAT.G.G.A AAA....AA. .TTT.TT.CC AGA...T... ....TCTCCC T.A..

L2Ecaballu

GT.AGGGTG. C.G.G.CCGG AAAT.G...A AAA....AA. .TTT.C.C.C AGA...T... ....TCTCCC T.AC.

L2Lafrican

GT.AAG.T.. C.A..ACTGG TCAC.G...A AAA....A.. TTTT.CTCAC GGA...T... ....TCT.CT T.AC.
