# Supplementary material for: Fragmented mitochondrial genomes in two suborders of parasitic lice of eutherian mammals (Anoplura and Rhynchophthirina, Insecta)
Source: Sci Rep. 2015 Nov 30;5:17389. doi: 10.1038/srep17389 (PMC4663631; doi:10.1038/srep17389)
Supplement: Supplementary Dataset 4 [file srep17389-s5.doc]

26 75

L1Dyakuba

ACTATTTTGG CAGATTA--- GTGCAATAAA TTTAGAATTT ATATAT-GTA ATTTTT---- --ATTACAAA TAGTA

L1Dmauriti

.......... .......--- .......... .......... ..T...-..G ......---- --........ .....

L1Dsimulan

.......... .......--- .......... .......... ..T...-..G ......---- --........ .....

L1Dmelanog

.......... .......--- .......... .......... ......-..G ......---- --........ .....

L1Dsechell

.......... .......--- .......... .......... ..T...-..G ......---- --........ .....

L1Chominiv

.......... .......--- .......... .......... ..T...-..G ......---- --G.C..... .....

L1Ccapitat

.......... .......--- .......... ......C... ..T...-... ....AA---- --........ .....

L1Agambiae

.......... ....C..--- .......... .......... ......-A.. ......A--- --....T... .....

L1Aquadrim

.......... .......--- .......... .......... ...A..-A.. ...AA.A--- --....T... .....

L1Amellife

GT......A. ..T.A..--- .....T.... .....G.... .AT...-A.T .A..C.TTTA AAT.A.T... ..A..

L1Bmori

.......... ....G..A-- A..T.T.GGT .......G.C .AT.TATA.. TAAA..AATT --TA..T.G. .....

L1Tdimidia

.......... .......--- ....GG.... .......... .CT...-... GG..G.---- --TC....G. .....

L1Lmigrato

.......... .....A.--- .....G.C.. .........C ..T...-... GA....TT-- --TC...... .....

L2Dyakuba

T...A.A... .......--- .......GG. ....AGC.CC ......-AA. G.A...T--- --.C.TTT.T ...A.

L2Dmauriti

T...A.A... .......--- ........G. ....AGC.C. ......-AA. G.A...T--- --.C.TTT.T ...A.

L2Dsimulan

T...A.A... .......--- ........G. ....AGC.C. ......-AA. G.A...T--- --.C.TTT.T ...A.

L2Dmelanog

T...A.A... .......--- ........G. ....AGC.C. ......-AA. G.A...T--- --.C.TTT.T ...A.

L2Dsechell

T...A.A... .......--- ........G. ....AGC.C. ......-AA. G.A...T--- --.C.TTT.T ...A.

L2Chominiv

T...A.A... .......--- .......GG. ....AGC.CC ......-AA. G.A...T--- --.C.TTT.T ...A.

L2Ccapitat

T...A.A... .......--- .......GG. ....AGC.CC ......-AA. G.A...T--- --.C.TTT.T ...A.

L2Agambiae

T...A.A... .......--- .......G.. ....AGC..C ......-AA. GA....A--- --TC.TTTGT ...A.

L2Aquadrim

T...A.A... .......--- .......G.. ....AGC..C ......-AA. GA....A--- --TC.TTTGT ...A.

L2Amellife

TT..A.A... ....A..A-- .....T.G.. C...AG...C .A....-AA. G.A...TTAA --.C.TTTGT ..AA.

L2Bmori

T...A.A... ....C..T-- A..T...GG. ....A.CCCC ..T...-AA. GGAA.AT--- --CC.TTTTT ...A.

L2Tdimidia

CT..A.G... ....AATTAT A......G.. ....AGC..C ..T...-AA. GA.CC.---- --TC.TTCGT ..AG.

L2Lmigrato

T...A.A... .......--- A....T..G. ....AGC.C. .A.A..-AA. G....GA--- --CC.TTT.T ...A.
