# Supplementary material for: Fragmented mitochondrial genomes in two suborders of parasitic lice of eutherian mammals (Anoplura and Rhynchophthirina, Insecta)
Source: Sci Rep. 2015 Nov 30;5:17389. doi: 10.1038/srep17389 (PMC4663631; doi:10.1038/srep17389)
Supplement: Supplementary Dataset 5 [file srep17389-s6.doc]

18 81

Hs_L2_taa

ATTCTAGTGG CAGAATTAAA ----GTGCAT TGAATTTAAG CTTCAAATAT GAGATTTATC AACC----GT CTCCTAGAGT T

Hap_L2_taa

G.....T... .......... ----...... .......... .......... .......... ....----.. .......... .

Bm_L2_taa

TC.G...... ......C..- ----.....C .......... A....G.... ..A..A.C.- --------A. T.....T..A A

Bm_L1_tag

T.CA.CT... ....C.A.-- ----...... CA......GA A..TG.T..A .T.....T-- --------T. .A.AG.TG.A A

DyakuL1

.C.A.TT... ....T.A--- ----.....A .A......GA A..T.T.... .TA....T.- --------A. TA.AA.T... A

PhumaL1

GC.AA.T... ....--.... ----....G. ..G.....G. T.C..TT... AT..AAACA- --------T. .ATA.TT..C .

PhumaL2

GC.AA.T... ....--.... ----....G. ..G....... T.C..TT... AT..AAACA- --------T. .ATA.TT..C .

Pp_L1_tag

GC.AA.T... ....--.... ----.....C ..GG....G. TC...TTC.. .TA.AA.T.A .-------T. TA.A.TT..C A

Pp_L2_taa

GC.AA.T... ....--.... ----.....C ..GG...... TC...TTC.. .TA.AA.T.A .-------T. TA.A.TT..C A

Hs_L1_tag

G..AA.T... .......T.. ----...... ........G. T......... A.T.-CA.A- --------TA T.-A.TT.AC A

Hap_L1_tag

G..AA.T... .......T.. ----...... ........G. T......... ..T.-AGTA- --------TA T.-A.TT.AC A

DyakuL2

TC.AATA... ....T.A--- ----.....A ..G....... ..C..T.... A.AG.A.T.T --------AC T.TTATT..A A

He_L1_tag

.GAAGGT... ....T.A.-- ----....G. ........GA A.....C... ...G..AGAT GGTTATTC.C ...ACCTTC. A

He_L2_taa

.GAAGGT... ....T.A.-- ----....G. .......... G.....C... ...G..AGAT GGTTATTC.C ...ACCTTC. A

Pa_L1_tag

GCCAAG.... ....G.A.-- ----...... ........G. A.....GA.. AG.GC.AG.- --------.C .CT.CT.G.C A

Pa_L2_taa

C...AG.... ....G.A.-- ----...... .......... .......... A.AGGC.TAA .-------CC T.T.CT...G A

Ps_L2_taa

C..GAGC... ....TAA.-- ----...... .......... A......A.. ..ATG..--- --------TA T..GCTT.AG A

Ps_L1_tag

C.CT.TA... ....TAA.-- ----...... ........G. A......A.. A.ACGA---- --------TG T.TTG.AGAG A
