# Supplementary material for: Fragmented mitochondrial genomes in two suborders of parasitic lice of eutherian mammals (Anoplura and Rhynchophthirina, Insecta)
Source: Sci Rep. 2015 Nov 30;5:17389. doi: 10.1038/srep17389 (PMC4663631; doi:10.1038/srep17389)
Supplement: Supplementary Dataset 6 [file srep17389-s7.doc]

17 8040

Alloeorhynchus_bakeri TTTTCTACCTTTGACCCTAGAACAGGCCTATTC---------TTTTCATTA------AACTGAACTAGAACGTTT---------------------ATTATTATTTTACTATTTCCCAATCTATACTGACTTTTCCCTACCCGGAATAGAATTTTATTCCTTAAAATAAGAAATAATCTTCATACTGAATTTAAAACCTTGTTAGGTGAAGGAGGA------AGGGGTTTATCTATTATATTTGTAACCTTATTTATATTTATTTTATTTAATAATTTATTTGGTTTAATTCCTTATATTTTCACTAGATCTAGACATTTAACTTTTTCCCTAGCCTTATCACTACCAATCTGATTATCATTTATAATATTTGGG---TGAATTAATATAACTCAAAACATATTAGCCCACCTAATCCCACCGGGGACACCTAGAGCTTTAATACCATTTATAGTATTAATTGAAACTATTAGAAATATTATTCGTCCAGGATCATTAGCTGTTCGATTAACAGCTAATATAATTGCAGGGCATCTTTTAATAAGCCTACTAGGTAATAATTTAATTGAAAATTCCAATTTAACAAGAGCCTTACTAATTATAATTCAAATAATATTAATATTATTTGAATCAGCAGTTTCCATAATTCAAGCATATGTATTTTCTGTCTTAAGAACACTTTATTCTAGAATAGCACCTCTATGATGAAGAATTTTATTTATTATATTTATTATTTCTCTTATAGCAACATACTCC---AACTTGTATTTCCTATGAATACCAAGAAATACAAGTAGAATTGTGAATAAAAAT---------------ATAGAGGAGAAAGTAAATTTATATTCCACAAATCATAAAGATATCGGAACTTTATACTTTATCTTCGGACTATGAGCCGGAATAGTAGGAACCTCATTAAGATGATTAATCCGAATTGAATTAAGTCAACCCGGTTCATTCATTGGAGATGATCAAATTTATAATGTAATTGTTACAGCACACGCCTTCGTTATAATCTTCTTTATAGTTATACCTGTCATAATTGGAGGATTTGGAAATTGACTTGTTCCTTTAATAATTGGTGCTCCAGATATAGCCTTCCCCCGAATAAATAATATAAGATTTTGACTATTACCCCCATCATTATCCTTACTATTAACAAGTAGAATTGTAGAAAGGGGGGCAGGAACAGGATGAACCGTTTATCCCCCACTTTCTACTAACATCGCCCATAGAGGAGCATCAGTGGATTTAACTATCTTCTCTCTACACTTAGCAGGGGTATCATCCATTCTAGGAGCTGTAAATTTTATTTCAACAATTATTAATATACGACCTTCAGGTATATCACCTGAACGCATTCCATTATTCGTCTGATCTGTAGGAATTACTGCCCTATTACTATTATTATCATTACCTGTATTAGCAGGTGCTATTACCATACTATTAACAGACCGAAACTTCAATACATCATTTTTTGATCCTTCAGGAGGAGGAGACCCTATTTTATACCAACATTTATTTTGATTTTTTGGTCACCCAGAAGTATATATTTTAATTTTACCAGGATTTGGATTAATTTCCCATATTATTAGTAAAGAGAGAGGGAAAAACGAAACCTTCGGATCATTAGGAATAATCTATGCCATAATTGCAATTGGATTATTAGGATTTATTGTTTGAGCCCATCATATATTTACAGTGGGAATAGATGTTGATACACGTGCATATTTTACATCAGCCACAATAATTATTGCCGTACCCACAGGTATTAAAATTTTTAGATGACTGGCCACTCTACATGGATGTTCAATAAATTTTTCTCCTTCTATATTATGAGCACTAGGATTTGTATTTTTATTTACAATAGGTGGATTAACTGGAGTAATTCTAGCTAATTCATCTATTGACATTATTCTACATGATACTTATTATGTTGTTGCCCACTTCCATTATGTACTATCTATAGGAGCCGTATTCGCTATTATAGGAAGATTTATCCAATGATACCCCTTATTCACGGGGATAACTATAAATCCCCAATGACTAAAAATACAATTTATAATTATATTTACAGGAGTAAATATAACCTTCTTCCCCCAACATTTTTTAGGGTTAAATGGTATACCTCGACGATATTCAGACTACCCAGACAGATTTATGACTTGAAACATTATTTCATCAATTGGATCTACAATCTCACTAATCGGAGTAATAATTTTCATTTTAATTATTTGAGAAAGAATAATTGCCAAACGGCAAGTATTATTCCCAATAAATATAAATTCTAGAATTGAATGACTACAAAGAACCCCTCCTAGAGAACACTCATATAACGAATTACCCATAATTTCTACAATCTCAACCCAAGATGCTAACTCCCCTCTAATAGAACAACTTATTTTCTTTCACGATCATACTATATTAATTCTCACAATAATTACTATTATAGTAATATATTTAATATCAACACTATTTATTAACAAATATATT---------AATCGGTTCCTACTAGAAGGACAGACCATTGAATTAATTTGAACTATTCTTCCTGCCATTACCTTAATTTTCATTGCATTACCATCACTACGAATTTTATATATCATAGATGAAATTAATAACCCATTAATCACCATCAAAGCAATTGGTCATCAATGATTTTGAAGATATGAATATTCAGACTTTAATAATATTGAA---------TTCGATTCATATATAAAGCCATCTAATGAACTAACAATAAATGAA---TTCCGATTACTAGATGTAGATAACCGAATCATTATACCTATCAATACGCCCGTACGTATTCTAGTTACTGCCACAGATGTTATCCATTCATGAACCATTCCTTCGTTGGGAATTAAGATTGATGCAACCCCCGGACGATTAAATCAAGGATCATTTAATATTAAACGACCAACAATTATATATGGTCAATGCTCTGAAATCTGTGGAGCAAATCACAGATTTATACCTATTGTTATAGAATCAGTTAATATCCCTAGATTTATTAAATGACTAAATATAAATAAAAATCACCCCTTTCACCTAGTTAATTATAGACCCTGACCTTTGACAGGATCAATTGGAGCATTAACTCTAACAAGAGGTATAGTTTCATGATTTCAT---TTAAATAATATATCCCTTTATATTTTAGGAATAATTATTATTCTTATAACTATAATTCAATGATGACGAGATATTGTACGTGAAAGTACATACCAAGGTCTTCACACTAATAAAGTAATTATTGGATTAAAATGAGGTATAATTCTATTTATTATTTCAGAAGTTTTCTTTTTTATTTCTTTCTTCTGAGGGTTTTTCCACAGAAGTCTAGCTCCAACATCAGAAATTGGTAGTTCATGACCACCCTCTGGAATTATAACCTTTAACCCCATACAAATTCCCCTATTAAATACTATAATTCTATTATGTTCAGGATTAACAGCTACATGAGCCCATCATAGATTAATA------GAAGGTAACCGAACACAAACATTACAAGGGCTTACATTCACTGTAATTTTAGGTATTTATTTCTCTATTTTACAAGGATATGAGTATATAGAATCACCCTTTGCTATTAGTGACTCCGTCTATGGTTCTACATTTTTTATAGCAACTGGATTCCACGGTTTACATGTAATTATCGGGACAATCTTTCTATCAGTATGTTTATGACGACATTTTATATATCATTTATCAAAACAACATCATATAGGGTATGAAGCAGCAGCTTGATACTGACACTTCGTTGATGTAGTTTGATTATTCCTTTACATTTCTATTTACTGATGATTATGATGAAACTGAGGATCCTTACTCAGACTCTGTCTATTAATTCAACTATTAACTGGAATTTTCCTAGCTATACATTACACAGCTAACATTGAATTGGCATTTAATAGAGTAATTCACATCTGTCGAGATGTAAATCAAGGCTGAATACTCCGCAATTTACACGCTAATGGCGCCTCATTATTCTTTATTTGTTTATACTTACATATCGGACGAGGTATATATTATGGTTCCTATAAATTAATC---ACTACATGGTATGTAGGAGTAGTAATACTATTTGTAATTATAGGAACCGCCTTCCTAGGATATGTATTACCTTGGGGACAAATGTCCCTTTGGGGAGCCACAGTAATTACCAATCTTCTATCAGCTGTTCCTTATTTAGGTAATGATTTAGTTAAATGATTATGAGGTGGTTTTTCAATTGATAATGCCACTTTAACACGATTCTTTACATTACACTTTCTATTGCCCTTCCTAATTTCAGCCCTAGTATTAATCCACCTACTATTCCTACATCAAACAGGATCTAATAATCCTTTAGGACTAAATAGTAATTATGACAAAATCCCATTTCACCCTTACTTTTCAATTAAGGACTCATTAAGAACAACAATTGTATTATTCCTATTTATTATATTAAGAATATTAGAACCTCGACTACTAGGAGACCCTGAAAACTTTATTCCTGCTAATCCATTAGTAACCCCAATTCACATTCAACCAGAATGATATTTTTTATTTGCATACGCAATTCTTCGATCTATCCCTAATAAATTGGGGGGAGTAATTGCTATAGTAGCATCAATCTTAATTATTATAATTTTACCATTAACTAACAAGCCTAAGATACAATCAATAACATTCTACCCTATTAATAAAAGATTATTTTGAATACTATCAGTAAATTTAATTCTATTAACATGAATTGGGGCTCGACCAGCAGAAGAACCATTTATTTTTACAGGACAAATATTAACTATAACTTATTTTTTATATTTCATTGTAAACATTTCTTTTTTATTATTAATTTTAATGGTTTTATTGGCTGTGGCTTTTGTAACTTTATTAGAGCGGAGGGTTTTAGGTTATATTCAGTTGCGGAAGGGTCCTAATAAGGTTGGTTTTATAGGGCTTTTACAACCATTTTCTGATGGTTTGAAATTATTTTTTAAGGAACAGACTTGACCG---GAAGTTTCAAATTTTGTGATTTATTTTGTTTCTCCAATCTTTATGCTTTTTCTTTCTATATTGATGTGGGTTGTGTTTCCTTTTTTTGTTAATGTGGTTAGT------TTTAATTTGGGAGTTGTATTTTTTTTATGTTGTTCTAGATTGGGAGTTTATGGTATTATATTATCTGGTTGGTCATCAAATTCTAATTATGCTATATTGGGAAGTATGCGTTCTGTAGCACAGACAATTTCTTATGAAGTAAGATTATCTTTGATATTAATTAGTTTGCTTATTTTAGTTCAAGGTTTAAGTCTTATTTATTTTTGGATTTTTCAGCAAAATGTTTGGTTTATTTTTTTATCTATC---CCATTATTTTTGTGCTGATTTAGTTCATGTTTAGCAGAAACTAATCGTTCACCTTTTGATTTTGCAGAGGGTGAGAGAGAGCTAGTTTCTGGATTTAATGTAGAATACAGTAGAGGTGGATTTGCATTTATTTTTTTATCTGAATATATAAATATTATTTTTATGAGTATACTGAGAGTTATTTTTTTTATAGGTTGCGATTTAGGTAGATTAATATTTTATTTTAAGGTGGTTTTTTTGGTATTTATATTTTACTGAGTACGTGGTACTTTACCTCGATTTCGTTATGATAAATTAATATATCTTACATGGAGGGGATTTTTACCTGTTTCTTTAAATTATATTTTATTTATAATCTCACTATTAATCCCTGTATTATTAATT------------------------GTAATGTGCACTTTAATTGCAAAAAAATCCAATATAGACCGAGAAAAACTATCCCCCTATGAATGTGGATTTGACCCATACAAATCCGCCCGAATACCATTTTCTATTCAATTTTTCATAATTGCTATTTTATTTTTAATCTTTGATGTAGAAATTGTGATTATTCTACCCGCTTCCATTACCTTAAAATATGGTATGCTAAGAAACTGAATTATTACT---TCTTCATTCTTCATTATTATCCTTTTACTAGGATTACATCATGAATGATATAACGGAATTCTCGAATGAACATTACTTTTAACTCTTTTGAGATTAGAGGTTTTAGTGATTTCATTATTTATTTTTTTATATATATATGTATTATTATATGGGGAAGGGTTTTATTTTATTATGGTTTTTTTAACCTTTTCTGTGTGCGAAGGTTGTTTAGGTCTTTCTATTTTAGTATCTTTAATTCGTTGTCATGGTAATGACTATTCTTGTACTTTAGTTATAACAATTCTTTTTGATTTTAAGTCTTTAATATTTATGTCTAGAGTTATAATAATTTCTTCAATGGTTATTTTTTATAGTTCTATTTACATAGAGGGGGATAAAAATCGAGTACGATTTTTATATTTAGTTTTAATATTTATTATATCAATAATGATAATAATTATAAGACCCAATTTAGTGAGTATTTTGTTGGGTTGAGACGGTTTAGGGTTAGTTTCTTATGGGTTGGTAATTTATTTTCAGAATTATAAATCTTATGCAGCTGGAATATTAACAATTTTAATAAATCGTGTAGGGGATGCTGCTATTTTAATTTGTGTGGGTTGGATAATTAATTTTGGAAGT---TGACATTATATATATTACTCATTCATCTGG---------GATGAATGAGTAATATATATCTGTTTATTATTAATTCTTGCTGCTTTCACTAAGAGAGCCCAAATTCCGTTTTCTTCTTGGCTTCCAGCTGCTATAGCAGCCCCAACACCTGTTTCAGCTCTAGTCCATTCTTCTACTTTAGTTACAGCCGGGGTTTATTTATTAATTCGATTTAGAGGT---GCTTTTGTTAATATAGATTGTTCTTTATTT---GTTTTGTTT---GGTATAATAACTATATTTATATCAGGTTTAGGGGCTAGTTTTGAGTTTGATCTTAAGAGGATTATTGCTTTATCAACTTTAAGACAGTTAGGCTTAATAATATCAGTCTTATTTATGGGTTATATTGATTTATCGTTTTTTCATTTATTAACTCATGCTTTTTTTAAAGCTCTTTTGATTTCTAATATGTCTTTATGTGGTCTTCCCTTTTTATCTGGGTTTTATTCTAAGGATTTT---ATACTAGAA---ATAATAGTTTTTGATTATTATAATTTAACGGTTTTTTTAATTTTTTATATTTCTGTAGGTTTAACCTCATGTTACACAATTCGTGTTTTATATTATTGTATTTTAGGCAATAAT---------------AATATATATGTGTGTCAA---------TTATATAAGGAAAATTATGTTATGATTTGTTCTATAATAATATTGGTTGTTATATCTATTATAGGGGGTTCAATTTTATCTTGG------------------TTAATTTTTAGATTT------CCAGAATTATTAGTTATGACTTTAACTTTAAAGTTAATAGCTCTTTTTTTTATTATATTTGGTATAATATTAATCTTAACTAGAATA---TTATTACCATTACTAAAG---CATCCATTAAGAATAGGACTAATCATTATCTTGCAAACCCTGATCACTGCTATAATTACAGGACAAATAATTAATATATTCTGATTTTCATATATTTTAATATTAACTATAACAAGAGGTATATTAGTTCTATTTATTTATATAGCAAGAATAGCATCA---AAT---GAAAAATTCAAAAGTATAAAGATATCTGTACTA------------------------TCACTTATCTTAATATTACTACCCTTTATAATTATAATTAATTATAAA---------------------------GAATTCTTTATAATTAATTTATTTAATAATAAATATATAACATTATACTTAAATTATGAA------------------CAATCCATAATTGTAACAAGTTTATTCAATAAACAAGCCTTAATAATTACCATTATACTAGTAATATACTTATTTTATTCATTAATCAGTGTATCCTGAATCGTAAATGTATTTGAAGGA

Bothriometopus_macrocnemis CTTTCAGTGTTTGATCCAGGCTCAAGAAATTTGTCTTTT---ATCCCTTTA------AAATGACTAATCTCTTTA---------------------ATTGGGTTTATATTAATT---TGACCAATATGGACAGTTAAATCTGGGCATTTTACTTTCATAAACTTTTTATCTATAGGATGAAAAAGTCAACTTGAAGAACAATTTAAGTTTTACAAA---------TTCCCTTTAATTTTTAGTTTGTCTATTTTTATCACAATTTTTTGTATTAATGAAATGGGAATAGTTCCTTATTTATTCACTCCGACAAGACATTTTAGTTTAAATCTTTGTCTTGCCCTTCCTTTATGACTAACTGGAATTATTTGTATA---TTAAAAAAAAATTGAAAAGCTTTATTTTCTCATTTGGTTCCTGAAGGGAGACCAATAGGGTTGGCTCCACTATTAGTGATTATCGAGCTAGTAAGTTTATTAATTCGTCCTATTTCCTTGAGAATTCGGTTAATATCAAATATTATGGCAGGACATATGATTTTAAGATTAGCATCTAGTGCGGTCTCTTCTATATCAATTAGCTCATCATTTTTAATAGAAATGGTGGTATTTGGTCTAATAAGTTTTGAACTATGTGTCGGAATTGTTCAAGCTTACATTTTTTCTTGCTTATTAGTAATGTATTGATCATTTTTACCAATTTCTTTTACATTGTTAATATTTTTTATATCAATTATTATCATAATTATTGTTGTT---CAAAACTTTTTTACTCCAGTCTTCAGTGAAAGAGATAGAGTGGGAAGGGCAGCC------TATATGATAGACAGACAGTTTGTTTATTTTTTTTCAACTAATCACAAAGATATTGGAATTTTATATATAATTTTTGGGGTGTGATCAGGATTGATTGGTTTTGGGTTAAGAATAATCATCCGAATTCAATTAGGTTCACCTTGGGGGCTACTGTTTGATGGTCATATTTTTAATGTTGTTGTTACAATTCATGCTTTTCTAATAATTTTCTTCATAGTTATACCTATAATAATTGGTGGATTTGCAAATTGGATAGTACCTATTATGTTAGGTGCTCCTGATATGGCTTTTCCTCGAATAAATAATATGAGATTTTGACTTCTTCCTCCTTCATTGATTTTACTTTTAATGGCTACATGCATGGAATCAGGTGTAGGATCAGGATGGACGTTATATCCTCCTCTATCTTCTATCTTAGGAAGACCGGGTTTAGAGGTGGGATATTTAATTTTCTCCCTTCATTTAGCAGGTGTGAGATCAATTATAGGAGCAATTAATTTTATTTCTACTATCCTTAATATGTGGTCTTATTCTTTAGATTTTACCAAAATACCTCTATTCTGTTGGGCTGTGCTTATTACAGCCATTCTTTTACTTCTATCTCTTCCAGTTTTAGCTGGAGCTATTACTATGCTTCTGTTAGATCGAAGCTTAAATACTTCCTTTTTTTCACCAGAATTAGGAGGTGATCCAATTTTATATCAACATTTGTTTTGGTTTTTTGGACACCCTGAAGTATATATTTTAATTATTCCCGGATTTGGTTTAATATCCCACATTATCAATGAGTGTAGGGGAAAACCATCTGCTTTTGGGAGATTAGGAATAATTTACGCTATACTTACAATCGGATTATTAGGATTTTTAGTTTGAGCTCATCATATATTTACAGTAGGTATGGATATTGATAGACGAGCATATTTTACAAGGGTAACTATAGTGATTGCTGTTCCAACAGGAATTAAGGTTTTTAGATGGTTGGGAACAATTTTTGGGAGAAAAATTAATTGATCTCTCTCTTCTTTATGAAGAGTAGGGTTCATTTTTTTATTCACCTTGGGTGGACTCACAGGTGTAGTATTAGCTAATTCTTCAATTGATACTTTCATACATGACACATACTATGTAGTTGCTCACTTCCGCTACGTCTTGTCTATAGGGGCAGTATTTGCTATGTTTGCTAGATTATTTCATTGATTTCCTTTATTTACAGGTTTAACTCTTAATCAAAAGCTTATAAAAATTCACTTCTTTGTAACTTTTATTGGAGTAAATTTAACTTTCTTTCCTCAACACTTCTTAGGACTGATGGGTATACCTCGACGTTACGCGGACTACCCAGATTTATATACTCCATGAAATTCAATTTCTTCAATTGGTAGATGTATTTCTGTGGTCGGATTATCAATATTGATTTATGCTATTTATGAAAGATTAATTTCTCAGCGAAAAGCTATTATTCATTCTATAAATATAAGCTCTGTTGAGTGATTATGAGGGTGTCCTCCTTCAGCTCATTCATTTGAGTCAATTTCTTTTATTTCTAAGATATTATTTATAGATCCAATTTCTTTTTCTGGGGAAATTGTTCAATCAGTTCATGACCATGTAATGATTATTATTACTCTAATTGTTATATCAATTAGATATGTATTTTTCATAATATTTTGTCGTTCGAGGCAGATTATGGGAGAACGATTATTTCAATCCAGAGAAATTTTGGAAACAGTTTGAACTGTTTTTCCAGTGTTAATTTTAGTTTTTGCAGCTATTCCCTCTCTACATAGTTTATATATTTTAGAAGAAGAAAAAAATCCAATTATCTCTGTGAAGATTTTAGGAAATCAGTGATATTGAACTTATGAGTTTAATACTGGCTATCATATTAAT---------TATAATTCTTACATAATTCCTTCATCAGATTTAGGAGATAGAGAT---CTACGGAATTTAGAAGTCGATAATAACTTGGTTCTACCAGTTGGGGTAGAAACTCGAGCAATTATCACATCGAGAGATGTGATTCACTCATGAGCAATCCCACCATTGGGTGTGAAAATAGATGCAGTACCTGGCCGAATTAATCAAACAGTCTTTTCAATTTCAATATCTGGTCTATTTTATGGGCAATGTTCTGAAATTTGTGGATCTCTTCATTCATTCATGCCAATTTGCGTAGAAGCTATTCCTCTAAAAAATTGGTTAAAATGATTAGAATTAACTCAATTTCACCCATTTCATATAGTCCAATTGAGACCTTGACCATTGATTTGTTCTTTTAGACTATTTAGAATAATCATTTTAATATATGATTTTTTTAAT---TCTTCAAGATTAAAAAGTTTAATTTTTGCTGTACTTATTCAATTATTAGTTTTGTTTGAATGGTGACGAGATGTGTGTCGAGAAAGAACTTTCCAAGGTTGACACTCTTCCAATGTCGTTCGAGGATTAAAGATTGGATTTATTATATTTATTTGTTCAGAAGTCTTATTTTTTTTTTCTTTCTTTTTTGGATATTTCTTTCTTTCTCTTAATCCGGATGTGGTCTTTGGGGGAATTTGACCCCCAAAGGGCTTAATAGTAGTTGATTTTTTATCCGCTCCCACTCTTAACTCAATTCTTCTTCTATCTAGGGGAGTAAGAATTACTTGAGCACATCATAGAATTCTT------GAATCTAATTTATCTGAGGCAAAAATGGGATTAGTTTACACAGTTTTTTTGGGAATTATATTCTCTATAATTCAATTAATTGAATATTATGAATGTTCCTTCACTATTGCTGATAGACCTTTTGGGTCTATGTTTTTCTTAGCAACTGGATTTCATGGTATCCATGTTTTAGTAGGAACAATTTTTATTATTATTTCTTTTGTTCGCCTTTTAAATAATCAATTTTCAAAGAACCATCACGTCGGCTTTGAAATGAGTTGTTGATATTGACATTTTGTAGACGTAGTATGATTGTTTTTATTTGTTCGAGTATATTGAATATATATGTGGAATTTTGGATCTCTTTTAGGAATTAATCTTCTTCTTCAAATTGTGAGAGGGATCTTTTTAGCAATACATTATGAAGATACTATTACTTCTGCATTTGAAAGAGTGGTTAGAATAATAAATGATATAAATAGAGGTTGATTAATTCGCTTTATTCATGCGAATGGGGCTTCTTTATTTTTTGTTCTTCTGTACTTTCACATCGGTCGAGGGTTGTACTACGGGAGATATAATTTTACA---GGAACTTGGATTGTGGGAGTGATAATTATATTCATTCTCATAGGAACAGCATTTGTAGGGTATGTCCTCCCATGAGGACAAATGTCATTTTGAGGGGCCACAGTTATTACTAACCTTGTTTCGGCTGTCCCATTTATTGGGACAGATATGGTTATTTGATTGTGAGGTGGATTTTCCGTTGACAATCCCACCCTAGTTCGTTTCTTTTCAATCCACTTTGTCTTACCGTTTGTTATTTTGGCTATAGTTATTCTTCATCTTTTATTTTTACATTCAACTGGAAGATCCAACCCACTAGGATTAGCAAATGACAGGGATAAAGTTTACTTTCATCCCTTATTTTCAATTAAAGATATCCTCGGATTAATTATTGTAACATTTTTCTTTCTTTCCACGGTCTTTTTAAAACCAGAAAGATTGATGGATCCAGACAATTTTACTCCTGCCAACCCTATATCTACTCCCCAACACATTCAACCTGAATGATATTTCCTATTCGCTTATACCATTCTTCGATCGATTTCATCAAAGTTTGGGGGAGTAATAGCTTTGGTGTTTTCAATTCTAATTTTAATATTCTTACCTCTTCTTAATATCAAAAGATCTCATAGAATCTTATAT------------AAATTTTTTTTTTGAATTCAAGTTTCAAACTTTATTCTTTTAACCTGATTAGGTTCCATACCTGTAGAACAACCATATGTGCAACTAGGACAAATAGTTTCAATTAGCTATTTTTCTGTATTTCTAATTTGAATTCAACTTATTTTTTTAGCTGTCGGTGTTCTTTTATCTGTTGCTTTTTTTTCACTTTTCGAACGGAAAGTGTTGGCTATCATTCAAAATCGAGTCGGTCCGGACAAAGTTGGGATCGTAGGAATTCTTCAACCTTTTAGTGATGCAATGAAATTGATTTCAAAAACTGAATCCCCATCTCCAAGAAAAAAGGTTGGAGTATTGTATTTTTTTTCTCCAGCTATTATATTTGTAATTTCTTTTATTGTTTGAATTACTTTCCCTTCGAATTGAGAAATTTATTCA------TTTGATAAGAGGATATTATTTGTTATTGCCTGTATAGGCTCAAGAGTTTACGGTCTTGTAATAACTGGTTGATTTTCTAGGTCTAAGTATTCCTTAATTGGAAGGGTTCGAGCTATTGGAATGTCCATTTCCTATGAAATTATCCTAGTATTGGGCCTAGTGTTAATAATATTTCTTCTTGGAACTATAAGGGTAAAATATATTATTTCTTTTCAAGAACAAATGTGGTTGTTCTTTCCTTTGTGA---TTTGTATTTATTATTCTTTTAATCTCTTTTTTAGCCGAAAGTGGTCGAGCTCCTTTTGATCTCTCCGAAGGAGAGAGAGAGTTAGTGTCAGGATACTCTGTTGAGTATGGAGGTATTTCATACACTTTAATTTTTCTATCAGAAAATAGATCTATTATTTTTAGATCAGTTATCTTATCAAGAATATTTTTTGCG------ATAGGAAGAATTTTAACTCTTCCTGGCTTAATTTTTCTT---------GTCGTTTGAATTCGTGGGACGGTGCCACGAATGCGATTTGATCAATTAATAATAATGTGCTGAGTTAAAATTCTTCCTATTATGTTATTTATTTTTGGAATAATTTTTGTTGTTTCTCTTGTATCTCTTCTGGTT------------------------TTTATCGCAAAATTGTTCGATTCTCACGAAGAAGAA---TCTAGATCTGGAGAAGAGTTTGAATGTGGAATAGAATCAATACACCCTACTCACACTCCCATAAACATACAATTTTTTATAATTGGAATTTTATTTCTTATTTTTGATATCGAAGTGGTAGTAATACTACCTTTAATCATTTTA------TCTTGAAATGAAAGAGCAATTTTGATTATT---ATATCTATGATTGCGGCTATTTTAATTATTGGAATATGAATAGAAATTTTCATGGGTTCGTTATATTGAAAGATAATAATATTTCTTATTACTCTCGAATCTGTGATGATACTACTATTTTTTTATATAAGATGTTTTCTCAACGCT---------CCAATTATTTCATTAGTAACCTTTTTAGTTTTAATAGTTCTGGAGGGGGTGGTGGGGTTGAGAGTGTTAGTAAGAAGTGTGAAAATATCTGTTTCTCCATTTGAACTATCATCAGATATAACTATCATATTTGATCAGTTAAGAGTGAGATTCATATTCATGGTTCTCTCAGTTAGAACTTGCGTTCTAATTTATGCTGTGTGATATATAGAAGGAGAAAAAAATTTTAATAAATTTATTGTAACTTTATTCATATTCATTATTTCAATAATGTTCTTATGTATGAGAACAGATATTTATTGAGTTATAGTAGGTTGGGATGGACTAGGAATCACTTCATTTTTTTTAATTATTTTCTTTCAAAATTGAAAGAGGGTAAGAAGGGGGATAGTAACTCTCCTATCTAATCGAATTGGAGACGTGTTTATTGTTACATCAATTTGTCTGGATGTATTTTATTTCGAA---AGAAAATATTCATTG------------------------------------------------ATTTTAATTGCTCTTGGAGCAATTACAAAAAGTGCACAATATCCCTATTCAGCGTGACTTCCGGAGGCTATAGCAGCTCCCACTCCAGTTTCTGCATTAGTCCATTCTTCTACGTTAGTAACTGCAGGAATCTACCTCCTTCTTCGATTTAATGAC---ATATTTAATAATGAATTAGTAAGACTGTTC---ATTCTTTCAGTTGCGAGAATGTCAGCTTTTTTGTCAATCTCTAGAAGATGGGGTGAGTTGGATTTAAAAAAAATTATTGCTCTATCTACTCTCTCTCACTTGAGAATGATAATTTTATTCATCTCTTGTAAAGATTATCTCTGTGCAATTATCCACATAATTAGACATGCATTTTTTAAATCTTCTTTATTCTCTTGTGCTTCAATGGTGGGTGGACCGTTTTTAGCAGGATTTTATTCTAAGGAAATT---ATAATTATA---TCACTATTTTCATTTTCTCAAAAAACTTGAATTTTATGAATAACAATGTTTTTGGTTTTTGGGTCATGTCTTTATTCTGCCCGCATTATTTTCCGCCTTCTATCAAACTTA------------------CCATTCTATTCCTACTCT------------TCGGATCCAGAAGAGTTTAATTCAACCCCTCTACTCATCGTAACTATTCTTAGAATTTCTGCTGGAAGGATAATCATCTGA---------------CTGACTATTTCTTCTTGA------CCAAGATTATCATTTCAGCCAAAAGAATCTGGGGTGAGAAAGTTATTT---CTTTTTTTATCCATAATATTTGTTGGATTAATGGGT---ATTTTCTGTTTAAATATC---AGTCCAATGATGGGAATGATTTATTTATTAGTGAGTACAACTATTTTTACGACAATTCTTTTAATTGTCAAAGGATCT---TTTGTAGGATTTCTATTTTTTTTGTCTACAATTTCCGGACTATTTATTCTTTTTTCTATTTTTATAATAAGAATGAAA---------ATTAAATTT---TTTTCTAAAACTCTTGTGTTAAAA---------------------AGAAAAATCTTCTTTATAATTATCCTATCATCCTTAACAATAATGAGG------------------------ATATTTTCATCTCCGCCAAATGAATGAGGGACATATTTATGATTGGAGGAACAAATTCTTTCA------------------AAATCAGGGTTAATA------------------------ATTTGAATTTATGTTTTTTTAATTTTTTTATTACTTTTATCTCTTCCAATTGTCGATCAAATCCTCAAAGACATTTCTTCT

Campanulotes_bidentatus TATGGTTTGTGG---ATTGTTTTCTTCGTTTGATCCTTCTTATATAGAATTATATGGGTATGAATCATTACCGTTAAAATGGTTGATGAGATTTGTGCTGGTTTTTTTTTTTGTAAGTGACGAGTTTGGGTTTCTGTTTCTGGAGTTGATTTATTTGATAAAGAAATAGTGAAGTTTTTTTTTGATCTTTTAAAACAATCTATAGGAGGGTTTATG---------AAGATTGGACTGGTCTTATATAGTGTTTTTTTTTTT---TTCTTTTCTAATTTGCTAGGATTAGTTCCTTATGTATTTACTCTTTCTTCCCATTTGTGTGTAAATTTGAGTGTTTCTTTAGTTTTGTGGCTAGGAGGGGTGGTTTATTCG---ATAAAGAAATCTATGGATTCGTTTCTTTCTCATATAGTTCCTTTAGGTTCTCCTGTATTTCTTCTTCCTTTATTGGTTTTAATTGAAACAATTAGGACATTGATTCGTCCGTTAACTTTGGCTATTCGGTTGATGGCTAATGTGATAGCGGGCCATTTAATTATAAGGTTGGTTGGAGGTTTTAGATCAAGACTGGGTTTAGTATCTATT---TTTCCCATGTTTATTGAGTTGGGATTTTTGTTTTTTGAAATGTGTGTGGCTGTTGTTCAGGCTTATGTTTTTAGCAGGTTAATAGTGATATATTTAGCTATAATACCGATTTATTGGTTTTTTGTTTGTTTGATAGTAAATTTATTTATTTATTTTTTTCTTAGC---TTTCATTATTTTAATTTTCTTGTTTTTTCTCATTCTGAAAAAGTTTGTTTT---------AAAAGTGTTAAAGTGAGGTTTATTAGTTTTTTTTCTTCTAATCATAAGGATATTGGTATGATGTATTTAATTTTTGGAATGTGGAGAGGATTATTAGGTTATGGGATAAGGGTAGTAATTCGTACGGAGTTAGGGGAAAGAGGTTCGTTGATTAGAGATTCTCATATTTTTAATGTGTTTGTTACGGCTCATGCTTTTTTAATAATTTTTTTTATAGTTATACCGATTATAATTGGGGGTTTTGCAAATTGATTAGTTCCTGTGATAGTAGGGGCTGTTGATATGATTTTTCCTCGGATGAATAATATAAGGTTTTGGCTTCTTCCTCCGTCATTAGTTTTATTATTAATAAGGAGGGTTATTGATAATGGGGTAGGAACTGGGTGGACGGTTTATCCTCCACTTTCAAGATTTGTTGGTCAACCAGGAAGGGCAGTGGATTATGCTATTTTCTCTCTTCATTTAGCTGGGGTTAGGTCAATTATAGGGGCAATTAATTTTATCTGCACTATTTTAAATATGTGAAGATTTCCGAAGAGGTGGGATTTGGTTCCGTTGTTTTGTTGATCGGTTTTAATTACTGCATTTCTTTTACTTCTTTCTCTTCCAGTATTGGCAGGAGCAATTACTATGCTTTTGTTTGATCGGAATATTAATACTTCATTTTTTGATCCGTCGGGAGGAGGGGATCCTGTCTTGTATCAACATTTGTTTTGATTTTTTGGTCATCCGGAGGTATATATTTTAATTCTTCCTGGATTTGGTTTAATTTCTCATATGTTAAGGGATAATAGTGGAAAAATAGAGGTTTTTGGATCATTAGGAATGATTTATGCAATAGTAGCAATTGGAGTGTTAGGATTTATTGTTTGGGCTCATCATATGTTTACTGTTGGTTTGGATGTAGATAGGCGGGCATACTTTACTTCGGCTACTATAGTAATTGCTGTTCCTACTGGAGTAAAAGTGTTTAGATGAATGGCTACTTTATTTGGAAGACGAGTAAAATGAAGTCCTTCGGAATTATGAGGAATTGGATTTATTTTCTTGTTCACTGTTGGGGGTTTAACTGGAGTAGTTCTTGCTAATTCTTCTTTAGATATTATTCTTCATGATGCTTATTATGTGGTTGCTCATTTTCATTATGTTCTTTCTATAGGAGCTGTATTTGCTGTATTTGGAGGATTTATTCATTGGTTTCCAGTAATTTTTGGGGTAAAAATAGAGTCTGTGTATTTAAAAGTTCAGTTTTTTTGTACGTTTGTAGGGGTAAACCTAACTTTCTTCCCTCAACATTTTCTTGGGTTAATGGGAATACCTCGGCGTTATTCTGATTATCCAGATATGTTTTATTCATGGAATTTTATTTCTTCAATAGGATCGCAAATTACTTTGGTTGGAGTTTCATTGTTTTTCTTTTGTTTAATTGAGGGGTTTTTTAGAAAACGAAGAGTGTTATTTTCGTAT------TCTAGGTCTTTAGAGTGAATGATTGGGTATCCTCCAAATTCTCATTCTTTTGAAATAGGAGTTCAAATTGTAGAATTTTTTTTGCAAGATAGGTGAGGTCCTTTAATAAGTCATATTTCTGGTTTTCATGATCATGTAATAGTTGTCGTTTTAATAATTTTAACTGTGGTTGTATATATTAATATGGTAGTTTTTTTTTTTCCTTGTTAT---------AGTCGGTTTATAAAAAGAAGGGAGGGTTTAGAAACTTTGTGAACTATTCTTCCTTGTATAGTATTAGCTTCTTTAGCTGCTCCTTCTTTGATAACTTTGTATTTGTCTGATGAGTTAAGTAATCCTGTTGTGACTTTGAAGGTTATTGGACATCAATGGTACTGGTCTTATGAATATGAGGATTTGTCGTCTTCTTCT---------TTTGATTCGTATATAATTCCTACTTGTGATTTGTTAAAAGGAGAT---TTTCGTCTTCTTGAAGTAGATAAGAGGGTAAAAGTTCCTTTGAATAGGGAGAGTCGGGTTTTTGTGACATCTTCTGATGTGATTCATTCTTGGACTGTTCCGTGTTTAGGGGTTAAGGTTGATGCTATTCCGGGACGGTTGAATCAGTTGAGTTTATATCCTTCTCGAGTGGGTTTAGCTTATGGTCAATGTTCGGAAATTTGTGGTTCAATGCATTCCTTTATACCGATTTGTTTAGAGGTGGTTCCTCAAGAGGAGTTTTTTCGATGATTATGGAAGTCTGGATTTCATCCGTTTCATGTGGTAGATTTAAGGCCCTGACCATTGGTAATGTCATTGTCTGTTTTCTCGTTAGAATTAAATTTGTATCATTTTTTGAACTTATCTGGAAGGGTGGTATGGATAATTGAGAGATTTTTTTCTTCAATTTTAGTAAGAGCTTTGTGGTGACGGGACGTAATTCGAGAAAGAACTTTTCAAGGACATCACTCTGAGGAAGTTCAAAAGGGATTGGTTCTTGGAGTGCTTTTATTTATCTGTTCAGAAGTAATGTTTTTTTTTTCTTTTTTTTTTGGGTTTCTTTTTTCTGCTTTATGTCCAGATATTGAGATTGGAGAGAGATGACCTCCTTTGGGGATTGAACCACTAAATTTTATGATGGTTCCATTAATGAACACTTTAATTCTTTTATCTAGGGGAGTGTCAATTACTTGATCCCATCATTCTATTATA------GAAGGGGATTGAAAGAATTCTCTTTTTGGGATGGTTATTACAGTATTTCTTGGATTTGTGTTTTCTTTTCTTCAATATGAAGAATATTTTTCTTGTTCATTTACGATAGCAGATAGAGTATATGGATCTTTTTTTTTTCTAATAACGGGATTTCATGGGATTCATGTGATTGTAGGAGTTTTGTTTATTATAGTAAGGTTATTTCGAACTTTAGTTGGGCATTTTTCAAAAAGTCATCACTTTGGATTTGAAGCTGCTGCTTGGTATTGGCATTTTGTTGATGTAGTTTGGTTGTTCTTGTTTGTAACTGTATATTGATGATATTTTTGGAATTTTGGATCTCTTCTTGGGGTTTGTTTAATAATCCAATTAGTTTCTGGGATTTTTCTTTCTTTTCATTATTCTCCTACTATTGAGGAGGCGTTTTCTAGAGTAGTGATGATTGTTGATGATGTTCCGTTTGGATGAATATTTCGAAGAATTCATGCTAATGGGGCTTCGTTTTTTTTTTTTTGGGTTTATTTACACATTGGTCGGGGTTTATATTTAAGGAAATATAAGTTAAAC---CCTGTTTGAATGAAAGGGGTTTTAATCTTTTTTTTTTTAATAGGGACGGCTTTTATGGGATATGTTCCCCCATGAGGTCAAATTTTTTTGGGGGGGGCAACAGTTATTACTAGTCTTTTATCTGCTATTCCTTATATGGGGGGGTTTTTAGTTAAATGGGTTTGGGGGGGATTTTTTGTTAAAGGACCGACTCTTCATCGTTTTTTTTCTCTTCATTATCTTCTTCCTTTGATTTTATCAGTCTTTGTTTTTTTTCAAGTTTTTTTTTTTCAAAGGAAAAGGGGGGTTAATCCCATGGGGGGGAGTTTAAATTCAAAAAAGGGATTTTTTGTTCCTTATTTTTTTTTTGTTGATTTAGGAGGAATTTTTTTTTTTTTTTTTTTTTTTTTTATGTTTGTTTTTGTTTTTCATGATGTATTAATAGATCCTGATAATTTTATTCCTGCAAATCCAATATCTACTCCTCCTCATATTCAACCAGAATGATATTTTTTATTTGCCTATACTATTCTTCGCTCTGTTCCTTCTAAGTTAGGAGGGGTAGTGGCTTTGGTTTTTTCTATTTTATTTCTGGTTTTTCTTCCATTTATTTCTTTATCTAAATCTTTGAAGATTCGAACGGGGTGAGAGTATAAATTTTTGGTGGTTGTTTTGTTTGTAGTATTTTTTTTGTTAACATGAATTGGTTCTATACCTGTAGAATATCCTTATGAGTTAGTTGGTAAGGTATTATCTGTTTTTTATTTTTTTGTTTTAATATTGATTATTGAGGTAGTAGAAGTTTTATTAATGGTTTTATTAAGAGTAGCTTTTTTTTCTTTGTATGAGCGTAAGTTGATAGGGTTAGTGCAGGGTCGAAAGGGTCCAAATAAAGTGGGGGTGGGGGGGGTTCTTCAACCATTTGCTGATGCTATAAAATTAATTAGGAAAAATGAATATGCTCCT---AGGAAGGTAATTAAATTTATTTATGCTGTTTCTCCTATAATTTCATTTTTTATTTCGTTGGTTTTTTGAATTATATATCCTGTGATTTGGAATTTTTTTTCC------TTAAATTTAAGGATTGTTTTTCTTTTAGTTTTGTTTAGAGTTTCGGTATATGGGTTTATTCTTTCTGGGTGATTTAGTTCTTCTAAATATGCTAATATTGGGTGTGCTCGGGCTTTAGCTCAGTCTATTTCTTATGAGGTTGGATTAACTTTGAGGATTATCTTTTTTTGTTTGTTTTTTTCTTGTATTTCTTTGTCAGAGATTTTAGAGGTTCAAGAGTTTTTTTTTTTTTTCCCTTTTTCCTTT------TTATTTGTTTTTATATGGAGGATTTTTTTTGCGGAAACAAACCGTCCCCCTTTTGATTTAGCGGAGGGAGAGAGTGAATTAAAAAAGGGGTTTTGTGTAAAGAAGGGGGAAATAAGGAATCCTTTAATTTTTTTAGGGGAAAATTTATCTGTTTTGTTTGTAAGGATTTTGTTAAAAGTTTTTTTTTTTAGAGGGTCGTTTTTTTTTTTATTATTATGGGGAGGCTTTTTGTTTTTGTTCGTTCTACTCTACCTCGATTTCGGTATGATAAATTGACGTCTATATTTTGGATTGAAGTATTGCCTTTACAATTGTTCTTTTCAGTTCTTTTTCTTATTTTGGTTTGCGAGTGTGGGTATTGTAATTATCTTTTTTTTAAGT------------------------TTTATTAGAAGAATATTTTGTGTAAATGAAAAAACA---GGAATAAATTTGGAAATGTATGAATGTGGAATTGAACCGATTCAAGAAGATAAAGCTCCGTTTTGTATACATTTTTTTTTAGTTGGGGTGTTGTTTCTTTTATTTGATGTTGAATTGATTGTTTGTATTCCTATAGTATGAATG------AGTGTGTATGAAAAGGTTTGAGGATTATTA---TGATTTGTGTTTTTTTTTATTATTTTTGTTGGGTTAGTTTTAGAAATAGTAATGGGGACGTTTGATTGAAAATTTGTAATAATTTTAATTTCTTTTGAAATAATAACATTAACAACTTTTTTGATAATGTTGTCTAAAGTGTGAATT---TTATATAATATTCATTTTTTCACTTCTTTATTAGTTTTTTCTGTAATGGAAGGGGTTTTGGGGGTGTCTATTTTGGTGATATTATTTTCTAATTCTAAGATTTTTTGTGAGAGAAATTTTGAAGTATTGTTAGTTGTTGATTCATTTTCTATAATTTTTTTGTTTACTGTAGGAATGGTTAGGAGATTTGTTTTACTTTATTCTAATTATTATATGATGGGAAGATTGTTTAAAAAGAAGTTTATTTTAGTAATGATGATTTTTATTCTTTCTATATTTGTTTTAAGGTTAAGTGGAGATTTATTTTGGGTAATGATTGGATGAGATGGATTGGGATTTTCTTCTATGTGTTTGATTTTTTTTTTTCAAAATTGGAAAAGGTTTAATAGTTCAATGGTAACTTTTATTTCTAATCGAATTGGGGATTTTTTGATTATCTCTTTTTTTTGTTTTTCTATTTTATTTAATGGG---AGATTATTTTTTGAGAATTATGTTTCCTCT---------------------TCTTTTTTAGGATTGTTTTTGTGTTTTGGTGCTTTGAGAAAGAGTGCTCAAGTTCCATTTTCGGCTTGACTTCCTTTGGCAATAGCTGCTCCAACTCCTGTTTCTTCTTTGGTTCATTCTTCTACTTTAGTTACGGCAGGGGTTTTTTTATTAATTCGTTTTAAGAGT------TTTCTTTGTGAGAAATTATTTAGAGTA---ATTTTTTTGGTTAGCTTTATTACTATTTTTTTGGCTGGGATAAGATCAGTGGGGGAATATGATTTAAAAAAGGTAATTGCTTTGTCAACTCTTTCTCATATTGGATTAATAATAATGTTTGTGGGGATGGAGAGATTTATTTCTGCAAAGATTCATTTAGTAATTCATGCCTTTTTTAAGTCTCTTTTGCTTTCAACTTTTAGGATGATAGGAGCACCATTTTTTTCTGGATTTTTTTCGAAAGAAATT---CTTTTAGGA---TGAGTATATGAAAGGACGGTTAGAATGGTAAGAGTATTATTATTTTTTTTTTCCGTTTCTTTAACTTGTTCATATTCGATTCGTATGTTATTTTATTTTTTTTTTCCCTGTTTT------------------TCTTTTTCTTTTATAATTCCAAGGAGTGAGTATCCCTCTAAGATGTTTTTTTTTATAAGAGTTGTTTCTTCTTTCTTAGCAGGAGTGATGGGGTTTGTTCTAATTGGG------------------AAGGAGGAAAGAGAG------ATTTCTTTTACCGTATTTTCTTCTTTTCGGAAAGTGGGGTTATTAGTAAGGATTGCTATTGGATTTTCTTTTTTATTTATTAGTTTG---TTTTTTTGGTGGTTTTCTTTGTCGAGGAGATTGTCTTTATTGATGGTGTTAGTTTTTTCTGTGATTAGAACGTTGAGTTTTTCTTTTTTTTTAGAGTTTAAGGTCCCGTTGTTTATAGTGGTTTTAACTCTTAGAGGGGGTTTGTTTGTGTTGGTTTCTTTTGTAATCATGTTTTTTCCT---GATGATTATAAGTTTGGGGTAACTTATGCGAAAAAAATCTTT---------------------TATTCTTGTTTATCAATAGTAATATTAGGGATGTCGATGTCTTTTTTT---------------------------TGAAGGGGAGATTTGTTAATAGGGGAGGAATTGGTTGTTGGTGGGTTTAAGGTTTGGGGA---------------------------------------------------AGATTTATTATGGTTTATTTTTTTTTTATTTTATTACTTTTTTTGTTTTTATTTGTAGTTAATAGGGTGGTAAATCTTAGAACTGGG

Haematomyzus_elephantis ATGTCTAGATTTGACCCGTGCTCGAGGGTAACTGGATTA---ATAAATTTATCAGAGAAGTGAATATTTGCTTCA---------------------TCAGGTATCATTCTATCAGGAAGAGTATTCTGACTCTGCCCAAGAGGGGTTCATTTTTTTATTGGCAAACTTGCGAAAGCTTTAACAAGTCTAACGCAACAAGATGTCTTTCATTTTAAA---------AGAGTGGCATTAGCCTGCTGTGCAATTTTTATTAATTTATGTGCAATTAACATAGTGGGTCTTTTACCATATTCTTTTCCACCTTCTAGTCACTTGACATTTAATTTGGCCTTAAGTCTGCCTTTATGGGCAGGGGGTTTGATGTACTCA---ATGAGAACTTCATTGAAGAGGTTTTCTTCTCATTTTCTCCCTGAAGGAAGGCCATTACCCCTCTCCCCCTTTCTAGTAATCGTCGAGATTATCTCTAGACTTATTCGGCCATTCTCTTTAAGAGTTCGGCTTATATCTAACATTATAGCAGGCCATATAATTTTGACTCTTATAGGCCAAGCTGCTGCTTCTTCTACCTTTGTTGTAATTCCA---GCTACGTTGACTCAAGCTGCCTTTATTGGATTTGAACTTGGAGTTAGGGTGGTTCAAGCTTTCGTTTTCATAAACCTGCTTTATCTATATTGACTTATGTTCCCTAGTTGGTGATTATTATTAATGATATTTTCTCTTATTGTTACTTTTGCAGTTATTTCA---ATTGTATTTTTTATGGTGAGGGAGGTTCCAGTTCTTGAGAACTCACGTAACTCA------TCAGCTCAACTTAAAGTGAGATGTCTGTTGTTTTCAACTAATCACAAGGATATTGGAATTTTGTATTTGCTTTTTGGAGCATGGTCTGGAGTTGTAGGTTTCACCTTAAGAATGTTTATTCGTATGGAATTAGGAGAGGCTGGTAAATTAATTTCTGATAGGCACATTTATAATGTCATTGTGACTTCTCATGCATTTCTAATGATTTTTTTTATAATTATGCCAATTATAATTGGAGGTTTCGCCAATTGACTAGTTCCAGTTATGCTGGGAGCCCCTGATATAGCTTTTCCTCGGCTCAATAATATAAGATTTTGGCTTCTACCGCCATCTTTATTCATACTTCTGTTGAGGGGGTTTGTTGGAGATGGAGTAGGCTCGGGGTGGACGGTGTATCCTCCTTTATCTAGAAGGGTAGGTCACCCAAGAGTGAGGGTTGATATTTCAATCTTCTCTTTACACCTTGCAGGAGTTAGATCTATTTTAGGGGCTATTAATTTCATTACAACTATTGTTAATATGTGAAGATTCCCTGCAAAATTTGAATTAATACCTTTGTTTTGCTGGTCAGTTTTAATTACTGCGGTTCTTTTACTTCTCTCTCTTCCGGTCCTTGCAGGGGCGATTACTATGCTGTTACTGGACCGTAACGCTAATTGCTCATTTTTTGACCCTTCAGGAGGGGGGGATCCAATTTTGTATCAACATTTATTCTGGTTTTTTGGTCATCCAGAAGTTTATATTTTAATTCTTCCAGGATTTGGACTAATTTCTCATATTATTGCTGAAGAAAGTGGGAAGAAAGAGGTTTTTGGTAGGCTGGGCATAATTTATGCTATAATTTCAATTGGAGTTTTAGGGTTTGTAGTTTGAGCTCATCACATATTTACTGTAGGTATGGACATTGACAGTCGTGCTTACTTTACAAGTGCTACAATGGTTATTGCTATTCCTACTGGAGTAAAAGTGTTTAGATGACTATCAACCTTATACGGCTCTACTCCATTCTACTCACCTGCCAAGCTTTGAGCAATTGGATTTGTATTTTTATTTACCGTCGGAGGGTTGACAGGAGTGGTTTTAGCCAATTCGAGAGTTGATGTTGTTTTACACGACACTTACTATGTAGTTGCACATTTCCACTACGTCCTCTCCATGGGAGCAGTGTTTGCTGTATTCGCAGCGTTTATTCACTGATTTCCTACAGTGACGGGAATTTCTCTTTCAAGTTCACTACTTAAAGCCCATTTTCTAACAACATTTATTGGTGTAAATGTTACTTTCTTCCCTCAGCACTTTTTGGGGCTGATAGGAATGCCTCGGCGATATAGGGATTATCCAGATATTTTCTTTGCCTGAAATGTAATCTCTTCAGCAGGAAGTGTGATTACCTTGAGAAGAGTAGTCTTATTCTTTTTTTTATTGTGAGAAGGGTTTTCATCGTGTCGAAAACTTACATTTAATAGGGCCTGCCCATCGTCTTTGGAGTGATTTATAGGAGGACCACCTTCTAGTCACTTGTACGAACAGGTGCCTGTGTTAGTTTATATATCATTATCTGACAGGCTTTCGCCTAGAATAAAATTTATTAGAAAAACTCATGATTTCATTTTAATTGTTGTAATTATAATTATTTCTTTAGTGCTTTATTTATCTTATTTTTTACTATTTGGATCTGGCTAC---------AGTCGGAGGGTTGTTGGGAGAGAAGGCCTAGAAGTTTTTTGGGCTTTGGTACCAATATGCCTTTTAGCAAGCTTAGCAGTTCCATCTCTACATTGTTTATACTTCAGAGAAGAGAATTTTAATCCTTTAATATCAATTAAAGCTGTGGGGCACCAATGATATTGATCTTATGAATACAGAGATTTTGATAGAGTTTCC---------TTCGATTCTTACATAATA------AGGGATTTAAATGTCTGAGAT---GTTCGCCTTTTAAGAGTTGATCAGAGTGTGATTCTCCCGGTTCAAGAAAGAATTCGGGCAATTGTTTCATCTTCTGATGTAATTCACTCATGAGCTCTTCCCGCTTTAGGCGTAAAAGTAGATGCAGTTCCTGGACGTTTAAACCAATCCCTTGTTCGCAGAGAGAAGGTAGGAGATGTATTTGGGCAATGTTCTGAAATCTGTGGTAGATTGCACAGTTTTATGCCTATCTGTCTAAGATTTGTTCCTAAGCCTCAGTTCTTAAATTGAGTTAAAAAATTAGGATTCCACAGATTCCACATTGTTGATCAAAGGCCTTGACCTATTATTTTAAGGGTTGGGGTCATAACTTCTATTTCGAATACATTTATTTTATACTTGAGCGATTGTAACTTCATAGGGGCTGTAACTAGGTGGATCGCTACTTGCACTTGCGCAGCTTTGTGGTGACGAGATGTAATTCGTGAATCGTTTTTTCAAGGGTTTCACAGTCACTCAGTTATGGCAGGTCTAAGAATAGGATTCATTTTGTTTATTGCTTCTGAAGTTATATTCTTCATATCTTTTTTTTGAAGGTTCTTCTATGTTTCCTTAAATCCTGATATCGAATGTGGCAGGGTGTGACCTCCCGCAGGGGTTCAAAGGTTAAGGGCATTTAATGTACCTCTTTTAAATAGAATTCTATTGATCAGAAGAGGAGTGTCCATTACCTGAGCTCACCACGCTCTCGTT------ATGAGGAATATAAAAGAAACAGCAGTTGGATTAGGGATTACATTGATATTAGGACTTACATTTTCTGTTGTCCAATCTTTTGAATACTTACACACTAGATTTTCTATAGCAGACAGAGTATACGGATCTGTATTCTTTCTAACTACTGGGTTTCATGGAATTCATGTTTTAGTTGGTAGAATGTTTATTACTGTTAGATTGTGTCGAACTATTTTAGGTCAATTTTCGTGTAATCATCATGTTGGGTTTGAATTTTCAGCTTGATACTGACATTTTGTTGATGTTGTTTGGCTGTTCTTATTCATTTCAATATATTGATGATATATATGAAATTTTGGATCCCTTTTAGGACTTTGTCTCAGAGTGCAGCTTGTAACTGGTTTATTCTTAGCAATACATTATAACCCTTCCGTAATAAATGCTTTTGAGAGGGTTGTAAAAATTATAAATGATGTTAATTGAGGTTGAGAAGTTCGACTTCTCCATGCTAATGGAGCTTCACTATTTTTTGTTTGTTTATTTACCCACATTGGTCGGGGTTTATATTATAAAAGGTACTCAACTAACCCTCTTACTTGAAGGGTTGGGGTCATTATTTTACTGATAGTTATAGCTACAGCCTTTTTAGGGTATGTTCTCCCTTGAGGGCAGATATCTTATTGAGGGGCTACTGTGATTACTAATTTACTGTCAGCCATCCCTTACTTTGGGGAAGATCTTGTTTTATGACTATGGGGTGGTTTTAGGGTTGGCAGTCCAACCTTATCTCGGTTTTTTACTATGCATTTTATTCTTCCGTTTATTGTCTTAAGCTTGGCTATTACGCACATTGCGGCCCTTCACATCAAGGGTAGAAGAAATCCATTAGGGTTGTCGAGGGATTATGATAAAGTTCCATTTCACCCTTATTATTTGGTTAAAGATATAGTCGGATTTGTGTTAGTGAGATTCTTTCTTTTAATAGTGGTATTTACATTGCCGCACTTATTCATAGATCCTGATAATTTTTCGGCAGCCGACCCTCTGAAAACCCCTCCTCACATTCAACCAGAGTGGTATTTTCTTTTTGCATACGCCATTTTACGTTCTATCCCTAGAAAATTGGGGGGCGTAGTGGGTTTACTAGCAAGAATTTTAATTTTACTTGTTCTTCCGGTAATAGAA---AGAAAATTCTCTAGGAGACGGTTTGAGCCAATCTCAAAGGTTATATTCTGGTTCTTAGTAAGAACTTTTGTAACTCTTACGTGAGTTGGGATAATGCCTGTTGAACACCCCTTTGATTTAATTGGAAAGGTATTATCTGTTTTATACTTTTTTGTGTTTATTATTCAAATTCAAATCGTTATATTTGTTGTTTCTGTTTTAGTGAGTGTAGCCTTTTTTTCCCTTTATGAGCGAAAAGTTTTAAGGTTGATTCATATTCGCAAAGGGCCTAATAAAGTTGGAGTTCTAGGCCTATTTCAACCATTTAGTGATGCTATGAAGTTAGTTAGAAAATCCATCCACCCTCCAGTCAAAGTCGAAAGGAGTTTATTATTTAAAATGTCTCCTATTATTTTAATCACAATTGTTGTGATAGTATGAAGAGTAATACCG------ATATATGGT------TACTCTTCTGTTTGAAGGGGGTTATTCTTACTATTACTTTTCAGTTTAACGTCTTATGGCCCTATTTTTGGAGGCTGAATCTCCAATTCATGTTTTTCAGTAATTGGTAGAGTGCGAAGGGTGATTATAATAGCTTCCTACGAGATCACGTTGTCTTTTTCAATATTAAGACTTTTTTTAATAGGGAAATCATTTTCCTTGGAAATCAGTTTCAACATGATAGACCTGCCACTCACTATTTTCTCAGTAGCCCCTTGACTAAGTGTTAGGTTAATTATCTCTCTTTTAGCAGAGAGGGGTCGGAGACCTTTTGACCTTTCAGAAGGGGAAAGTGAACTAGTAGCAGGTTACACAGTGGAGTATGGGGGTATTGATTACACCTTAATCTTCTTGGGAGAAAATATGTCAACCCTATTAATATGTGTGATTGCAAGAATAGTTATATTTAAC---TCCCTGAACATTGTGAGGGTTTTTAGCACAATTTCCCTTGTT------------ATTTTTATTCGAGGAGTGGTACCGCGAGTTCGGTATGACCATATAATTTTATTATGTTGAGTGATTATTTTACCTATTTTAATCAGAAGGGTGAGGTTAATGGTTATTATATTTACCTGTTTAATCATAATA------------------------GTTGTGAGAGGTTTATTCTCAGGGTCCGAATTAAAG---CAAAGGTCAAATGAGCCTTTTGAATGTGGAATGGATGTTTTTATTAGTTCACGGACTCCATATGCTCTCTATTCATATTTAGTCTTAATTTTATTTGTTATTTTTGATATTGAATTAATTGTTTCAATCCCTCTTGTGTTTACA---AACTTATTAACGTGTGATATTTGAAGAATTGTG---TGAACCATCTATTCACTTTTTATGCTTATAGGCCTGACAGTCGAATTTTGACTAGGAAGGTTGACATCTTGTCCTGTAAGGTGTTTAGTTGGGTTAGAAATAATGTCAGTAATTAGATTTTTACTAATAAATTCATCTGGAATTTTCTTTATAAGGCAAGCAACATCCTTAATTTTAATTATTACATTTGTTGTCTGTGAGGGGGTTTTAGGTTTAAGTTTGATCTCACCTTTTATAAAAAGCACCTCTTCATTCATAGCAATGGATGTTTCTGTTTTAATTAAAGTTGATTATCTAAGGTTAGGGTTTTTATTTATAGTAAGGGTTGTATCCTTCACGGTTCTTATTTATTCAAATTTATATATGGCAGGTTCTCCGGATTTTAATAAATTCATTTTAATCTTAGTGAGGTTTATTGCCTCAATAATAACTCTTGCTTTCAGCGGTTCCTTGTTTTGAGCTTTTATTGGATGAGATGGCCTCGGGCTAAGTTCATTTTGTTTAGTTATATTTTATCAGAATTGAAAAAGCTTTAATAGAGCTCTAACCACCTTTATCATAAACCGGGTGGGTGACGCCTTCCTTCTATTAAGGTTGTGGAGGATTTTATCAATAGGCTCT---AATATCTCCATGTTTTCTGCGAGAGCCCCT------------TTTGAATACGTGGTTGCGGGGGTTAGAGGTTTAATTTGTGCTTGCTCTAAAAGGGCTCAAGTGCCATTTTCTGCTTGACTTCCTTTAGCAATAGCCGCCCCTACGCCAGTTTCTTCACTAGTTCACTCATCCACTTTAGTTACTGCAGGCATTTATATGATTATTCGATTTAAATCA---GATTTTTTATATTCAAATTATCAGATTTTCGTGCTGTCTTTACTTTCAGGAATAACAATTCTCCTTGCAGGAGTCAGTTCTCTCGTGGAGTTTGATTTGAAAAAGGTTATTGCTCTTTCCACTTTACTTCACATTGGAGTAATGATTAACTCTGTTGCCCTTCAAATATTTGACCTTGCCTTGTTTCATATAATGGCTCATGCTATATTTAAGTCTTTGTTATTTATGATTTCAAGAATAGTCGGATTCCCTTTTATGAGGGGGTACTATTCTAAAGATTTAATTATCCTATCTTCCTTTAGAGTAGGAAGCTTTATAAAGTTGTGAAGAATAATAACTTTAAGGTCAGCCATCTTATTCTCTTCGGCTTACTCCTTTCGGCTACTTCTATTTTTGTCACTTAATCGTAGAAAGTCAAGCCCATTGCCCATGTTTACTCTTAAA------AAAGAAGTTTCATCTATTGAAATAGTTAAATTTAGACTCTATTTTGGCATTATTCTAAGAGTCTCCTTAGGGAGAGTATTGGAACTACACCAAGATTTAACAAAATGGGCA---------------ATCCTTCCGTCTCTCGTGGAAGTCGCTCTAAAACTGTTTGTCTGGTTAGCCCTTTTTTTAGGTATTTTATTTTGATTTACCCAAATT---TTATTTTGATTGAGGTCA---AGAATCGTGACTGAATTAATTTCTCTGATTAGTTTTACAATTATTGTATCATTTTGAAGGTTTATAACATGAAGATCGGAGTTTGCCCCTCTTATATTTACATATGCAACTTTGGGTGGGCTTACAATTTTAACGACTTTTATAATTATATGAAGGCCTCGTTGA---AGTAAGGTAGGCCGACCTTTGCACTCTCTAAGTAAA---------------------GTAGTCGCATGATTCGTAGTAGTTGTACTTTTTCTATGTCTAACAGAT---AGAATTATGCTAGATTTAAATACAGGAGTTACCCTTCCTTGACTTTGAGTTAATACTAGCTGAATGGGAGTTCAAGATTGAAGCGCTGGAGTTTCGAATCTCCATTGAAGATGAATTGAATCTCATTTTGTATGACCTAATGTGTGTATCATTGGATTTATGCTCATGATTCTACTTTTTAGACTCTTAATTGTTGACTCTTCTACTTACGGAAAAGGTGGT

Psococerastis_albimaculata TTTTCTGTTTTCGATCCATCTACAACTATCTTTAAT------TTATCCATC------AATTGATTAAGAACATTA---------------------CTAATCTTTATTATATTACCCCTCATCTTTTGGTTAGTACCGACTCGATTGAATTTAGTCTGAATAAAAATTATTTTCACTTTACATAACGAATTCAAAACCTTAATTGGTGAAAATAAATTAAATACAGGAAACACCATTATATTTATTTCCCTATTTTCATTAATTCTATTTAATAACGTCTTAGGACTTCTACCTTATATTTTTACTAGAACTAGTCATATATCAATAACATTAGCTCTTTCTTTACCCTTATGAATCGCGTTCATACTTTTTGGT---TGAATTAATTTTAGACAACATATATTCAGCCATCTAATCCCACAAGGAACCCCTGCAATTCTTATACCTTTTATAGTATGTATTGAAATAATCAGAAATATCATCCGTCCAGGAACTCTTGCCATTCGACTGTCAGCAAATATAATTGCAGGTCACCTATTAATAACTCTTCTAGGTAACACCGGTCCTAGTTTATCCCTTATTATATTAAATATCCTTATCTTCACCCAAATTCTTTTATTAACCTTAGAAACAGCTGTAGCTTTCATCCAATCATACGTATTTGCAATTTTATCCACTCTCTATTCAACAATAAATCCTATATGATGATTCTCCTTATTTATTATATTTGTAACAATTCTACTGTCTTCTAATTCA---TTAAATTATTTT------TATTCAAAAAATGCTCTAGATATTAATTCTTTTAAA------AAAAGAAATATTAAAAATAACATTAATTTATTTTCTACAAATCATAAAGATATTGGAACATTATATTTCATTTTTGGTATTTGAGCTGGTATAGTAGGGTCAAGTCTAAGTATCTTAATCCGGTTAGAATTAAGACAACCTGGATTATTTTTAGAAGATGACCAAACCTATAATGTTATTGTGACTGCTCATGCATTTATTATAATTTTCTTTATAATTATACCTATTATAATTGGTGGATTTGGAAATTGATTAATCCCACTAATACTAGGAGCTCCTGACATAGCATTCCCCCGAATAAATAATATAAGATTTTGATTATTACCCCCCTCTTTAACTCTCCTAATTTCAAGAAGCTTAGTAAATACCGGGGCAGGTACAGGTTGAACTGTTTACCCTCCGTTAGCAAGAACATTAGGACACCCTGGAGCATCTGTCGATCTAGCCATTTTTTCTCTTCATCTTGCTGGAGTAAGATCAATTCTGGGAGCAGTAAATTTTATCACTACAATTATTAATATACGATCACAAGGTTTATCATTCGAACGAATACCTCTATTGGTTTGGTCTGTTTTAATTACAGCTGTTTTATTACTTTTATCATTACCAGTATTAGCTGGTGCTATCACCATACTCCTCACAGACCGAAACCTAAACACCTCCTTCTTTGACCCCGCAGGTGGAGGAGACCCTATTCTTTACCAGCACTTATTCTGATTTTTTGGCCATCCGGAAGTATATATTTTAATTCTCCCCGGATTTGGCCTGATTTCTCATGTAATTAGACAAGAAAGAGGAAAAAAAGAAACATTTGGGGTTCTTGGAATAATTTATGCTATAATGGCTATTGGACTTTTAGGATTTGTTGTATGAGCACATCATATGTTTACTGTTGGTATAAACGTTGATACACGAGCCTATTTTACTTCTGCTACTATAATTATTGCTATTCCTACAGGAATTAAAATTTTCAGTTGACTAACAACCCTTTACGGGGCAAATATTACTTTTACTCCGTCAATCCTTTGGAGATTAGGATTTGTGTTCTTATTCACAATCGGAGGATTAACTGGGGTAATCCTGGCCAATTCATCTATTGATATTGTTTTACATGACACCTATTACGTTGTAGCTCATTTCCACTATGTTCTTTCTATAGGAGCTGTATTTGCTATTATAGCCGGTTTTATCCAATGGTACCCGTTATTTACAGGAATAGTATTAAACGAAAAACTTTTAAAAATTCAATTTTTCATTATATTTATTGGTGTAAATATAACCTTTTTCCCACAACATTTCCTAGGCTTAAGTGGAATACCCCGACGGTATAGAGACTACCCTGATGTATATACCTCATGAAATGTAGTCTCTTCAATTGGAAGAATAATTTCTTTAATTGGTATTATCTTTTTAATTTATATCATTTGAGAAAGATTTATA---ATACGCTTACCCTCATTTTATAATTATTCTAACTCATCACTAGAATGAACACAAAAGTTACCTCCTTCGGAGCATTCATACTCTGAATTACCTATAATTTTCAATTTAAATCTTCAAGAAAGTTCTTCTCCTTTAATAGAACAATTAAACTTTTTTCATGATCACTCCATCTTAATCTTACTAATTATTACATGCTCTATTTCATATTTAATAGTTGCAATAATTTCAAATAAAATCACA---------AACCGATTTTTAATAGAAAATCAATTAGTGGAAATGATCTGGACAGTTATCCCTGGGGTAATTTTAATTTTTATTGCCCTACCTTCTCTACGAATCCTTTACTTATTAGATGAAGTGACCGCTCCATCCTTAACCCTAAAAACTATTGGCCATCAATGATACTGATCATACGAATATTCAGACTTCATAAATGTGGAA---------TTTGATTCTTATATAGTCCCTCCTTCTGAAAATAATCAATCAGAC---TTCCGCCTATTAGAAGTAAATAATCGAATTGTATTACCTTATAATACACAAACCCGAATTTTAGTTACAGCCGCAGACGTTTTACATTCATGGGCAATACCTTCACTTGGAGTTAAAGTAGACGCTAACCCTGGCCGTATTAACCAAACAAGCTTTTTAATTAACTACCCCGGCTTATTCTACGGCCAGTGCTCAGAAATTTGTGGATCTGTACACTCATTCATGCCAATTGTACTAGAAAGAACAACTAAAAATAGTTTTATTAACTGATTATTATTAACACAAAATCACCCTTATCATTTAGTCAATGTAAGCCCATGACCTCTTACAGGAGCTATTTCTGCTTTATTTATAACTTTAGGAATAGTTGAATGATTTAAT---ACTCCCCAAAACTACCTAATAAAAATAGGACTTATCATAATAATTCTAACTATAATCCAATGATGACGAGATGTAGTGCGAGAAAGAACCTTCCAAGGAAATCATTCATTCAAGGTATCCCGAGGTATACGCTGAGGAATAATTCTTTTTATTACATCAGAAATTTTCTTCTTCGTATCTTTCTTTTGAGCATTCTTTCATTCAAGATTAGCTCCTAATATTGAACTGGGAATTATATGACCCCCAAAAAGAATTACCCCTTTTAATCCACTACAAATTCCCCTTTTAAACACTATTATTTTACTTTCCTCCGGAATTACTATCACTTGAGCCCATCACGCTCTATTA------AAAAATAATTATTCACAAACAATTCAAGCCATATTAATTACAGTAGTATTAGGAATTTACTTTACTATCCTTCAAGGGTATGAGTATGTTGAAGCTTCCTTTTCAATTGCAGACTCTATCTACGGATCATCTTTTTTCATAGCAACAGGATTTCATGGAATCCATGTAATTATTGGAACAATTTTCATTTTAATAATGCTAATTCGTCAATATAAACCTCACTTTTCTAATATACATCACTTCGGCTTCGAAGCCGCTGCTTGATATTGACATTTTGTAGATGTAGTCTGATTATTTCTATATGTAACAATTTACTGATGAACAATATGAAATTTTGGTTCACTTTTAGGTCTCTGCCTTGGAATCCAATTAATTACAGGTATTTTTCTAGCTATACATTACACAGCCGACATTAATATAGCATTTTCAAGCGTTGTACATATCTGTCGTGATGTTAATAATGGGTGACTTCTCCGAACCATCCATGCTAATGGAGCCTCGTTTTTCTTTATTTGTCTTTACACCCATATCGGTCGTGGTATTTATTATGGTTCTTATAATTTATTA---TTTACATGAATAATTGGAGTGATTATTTTATTTCTAGTTATAGGTACAGCATTCATAGGGTATGTTTTACCTTGAGGGCAGATATCATTTTGAGGAGCAACCGTAATCACAAACCTAGTTTCAGCCATTCCCTATCTAGGAACATCAATTGTACAATGAATTTGAGGGGGATTTGCTGTTGACAACGCTACATTAACACGATTTTTTACTTTCCATTTTATTTTACCTTTTATTGTTTTAGCTATAGTAATCATCCATTTATTATTTTTACACCAAACAGGCTCTTCAAACCCTTTAGGCCTAAAAATAAATATTGATAAAATTTCTTTCCATCCTTATTTTTCATTCAAGGATATTTTAGGCTTCTTAATTATACTCCTAATCTTAACATTGTTGACATTAATAGACCCTTATATATTAAGAGATCCAGACAACTTCATTCCCGCAAATCCTTTAGTAACTCCTGTTCACATCCAGCCCGAGTGATATTTTTTGTTCGCTTACGCAATTTTACGTTCAATTCCTAACAAGCTTGGAGGTGTAATTTTTCTAATTCTATCAATCGCAATTTTATTTATTTTACCTTTTTCTCAAAAAAATTTAATTAAAGGATACCAGTTCTTCACTATTAACAAACTCTTATTCTGAGTCTACGTAAACTTAATTATTCTTCTAACATGAATTGGAGCCCGCCCTGTTGAAGATCCTTATATTTTTATTGGGCAATCTCTAACATTAATTTATTTTCTTTACTTTTTAGTTAACGTGTCTTTTTTATTGATAATAATTTTAGTTTTAGTAGGGGTTGCGTTTTTGACATTATTAGAACGAAAAGTTTTAGGATATATTCAAATTCGAAAAGGTCCTAATAAGGTTGGGGTGATTGGTATTTTACAGGCTTTTAGAGATGCAATTAAATTATTTACTAAGGAAATAACGTATCCT---AATAAGTCTAATTATTTAATATATTACTTTTGTCCTTTAATTTCTTTTCTGTTGATTTTTATTATTTGAAGTGTCACCCCCTTTTTATATGTTATAATAAAT------TTTAATTTTGGATTTTTATTTTTTATGATGTGTTTGAGAATAGGAGTATATGGAATTATAGTGGCTGGGTGGTCTTCTAATTCTAGCTATTCTTTATTAGGAGGACTACGGGCAGTGGCCCAAACTATTTCTTATGAGGTAAGCTTGGGGTTTTTAATAATAAATATGCTTATTTTATGTGGGAGTTATAGTTTTATAGATTTTTTTTTTGGTCAGTATTATTGTTGATTTGTATTTACGAGATTT---CCTTTATTTTTTTTGTTGTTTACTTCTATACTAGCAGAAACCAACCGCACTCCTTTTGATTTTGCTGAAGGAGAGTCTGAGTTGGTCTCTGGTTTTAATATCGAGTACAGAAGTGGTGGGTTTGCCTTAATTTTTTTGGCAGAGTATTCGAGAATTTTATTAATAAGACTGGTTTTAGTATTGATGTATTTAGGGGGTGACACAACTGGGATACTATTTTTTTTTTTAGTAAGATTTATTTCTTTTTCTTTTATTTGGGCTCGAGGTACTTTACCTCGATATCGATATGATAAATTAATGAATGTATGTTGAAAGAGTTTTTTGCCTGTATCTTTATTATATTTAGTCTTTATTTTAGTCGCCATTATTGCAAACCTAATAATA------------------------ATTTTATGTATAATTTTATCAAAAAAATCATTATATGAACGAGAAAAATTTTCTCCCTTTGAATGTGGATTTGACCAAAAATCATCGTCTCGAATACCATTTTCTTTACGATTTTTTTTAATTACTATCATTTTTTTAATCTTTGACGTAGAAATCGCACTCATTTTACCCGCAATTAACCAAATTAATTTATCAACAGCAAATCAATGATTACTAATA---AATACTTTATTCTTAATTATTTTATTAATTGGTCTTTTTCATGAATGAAACCAAGGAGCACTGGAATGATCTTTACTGGTTATATTAGTAAGTTTAGAATTTATAAGACTAATTATTTTTTTTATATTAATGTTTAATTTGTGGATGTTTAGT---GAGAAATATATTTTAATATATTATTTAACTTTCTGTGTTTGTGAAGGGGCATTTGGGCTATCTTTATTAGTGTGTTTAGTTCGGTCTGTAGGTAATGACTATGGGGTGGGGGTAAGAATATGTGTTTATATAGATTGGATATCTAGATCTTTCATAAGATTCGTTTTATTGATTTCTTTTGTTGTTGTTTGTTACAGAATTAGTTATATAGGATCTGATAAATATTCTAGAATATTTATTATATTAGTTTTTTTTTTTGTATTGTCAATAATGTTGTTGATCATTAGACCTAATTTAATTAGAATTTTATTGGGATGAGATGGGTTGGGATTAATTTCTTATTGTTTAGTTATTTATTATCAAAACATTAAATCTTATAATGCTGGGATGGTGACAGCGATAACTAACCGTATTGGAGATGTAATACTTTTAATGGGAATTGCTTGAATAATTAATTTTGGTAGT---TGAAATTATTTATTTTATTTTAATTATTCT---------GATAATATATTTTATATTATTGGAGTATTTATAGTATTGGCTGCAATAACTAAGAGTGCACAAATTCCTTTTTCTTCTTGACTACCAGCTGCAATAGCAGCTCCTACGCCTGTTTCTGCGTTAGTTCATTCATCAACTTTGGTTACGGCAGGTGTTTATTTATTAATTCGATTTGATTAT---ATTTTTAAATATAGTTTTATTTATAACTTT---ATAATTGTAGTTTCTGTTCTGACCATATTTATATCAGGGCTAGGGGCTATTTTTGAATATGATTTAAAGAAAATTATTGCTTTATCAACATTAAGTCAACTTGGACTAATAATTAGAACTTTATGTTTAGGGATAACTGAATTTTGTTTTTTTCATTTGTTGACTCATGCTTTATTTAAATCTCTTTTGATATCTAATTTATCTTTATGTGGAATACCATTTTTAGCTGGGTTTTATTCTAAGGATTTG---ATTTTAGAA---AATATTTTAATGTTTAATATGAATAGACTTATTTATTTTTTATATTTTTTATCTACAATGTTAACAGTTGTGTATACATTTCGATTAATTTATTTTAGTATAGTTAATTCATTT---------------AAATTAATACCTTATCAT------TGTTTTAATGATGAAGATTATTTAATACTTTTTAGTATAATTGTAATAGTTTTTATAGTTATTATTGGAGGTTCTATAATAATGTGA------------------CTAATGTTTATAGAT------TTTAGAGGAATTATTTTAGGGTTGTATTCTAAACTATTGACTTTGGTAGTTTGTCTAACAGGGTCCCTGACTATATTTTTAAACATA---ATTTTCTTTATTTTAATG---ACCCCACTTTCCTTAGGACTAACATTAATTCTCCAAACATTATTACTGAGACTCCTATTAGGTACAATAACATCATCCTTTTGATTCCTTTACTTGCTAGTTTTAATCTTTATTGGGGGGATATTAGTATTATTTATCTATGTGACCTCTATTTTTCCA---AAT---GAAAAATTCTCATTTACACAAAACAACATTTTTATT---------------------CTACTTATCTCAGTTTCTTTACTAAGAACAATTTTATATATTCTAAAT------------------ATAAATTTTATTATAACCCCAAACTTAAACTATTTAGAAAATATCTTATCTATAAAGTCAAATACAATT------------------ATAATCAGAACTATT---AAAATTTTTAACACCCAAGCTAATATAATCTTAATTTTTTTAGTTAATTATTTGTTCTATTGTATAATAATTGTTATTAAAATAACAGCCTTTTTTAAAGGA

Longivalvus_hyalospilus TTCTCTGTTTTTGATCCATCAACAACTATTTTTAAT------TCATCCATT------AATTGATTAAGTACATTT---------------------TTAATTTTTATTATACTACCCTTAATCTTTTGACTCGTTCCTACTCGATTCAATTTAATTTGAATAAAAATTATTTTTATTCTCCATAAAGAATTCAAAACTTTAATTGGACAAAATAAATTAAATCTAGGAAATACTATCATATTTATCTCCCTATTTTCACTCATTTTATTCAATAACGTCCTAGGATTATTACCATATATCTTTACAAGAACAAGCCATATATCTATAACCTTAGCTTTATCATTACCTTTATGAATTGCATTTATACTATTTGGA---TGAATTAACTTTAGTCAACATATATTTAGACACTTAATTCCACAAGGAACTCCTGCTATTTTAATACCTTTTATAGTTTGTATTGAAATAATTAGAAATATTATTCGTCCAGGCACTCTAGCTATCCGTCTTTCTGCTAATATAATCGCAGGTCATCTTCTCCTAACTTTATTAGGTAACACCGGACCTTCTTTATCTATATTCATATTAAACATTCTTGTTTTTACTCAAATTCTTCTTCTCACTTTAGAAACAGCTGTAGCCTTCATCCAATCTTATGTATTTGCAATCTTAACTACTTTATATTCAACAATAAATCCAATATGATGATTCTCTCTATTCCTTATCTTTGTAACCACCCTTACAATCTCTAACTCA---TTAAACTATTTT---------TATTCAAATAATTCTTTATCTTCCTCTTCTTTA------ACCAATAAAAATACAATTAATGTAAATTTATTTTCTACAAATCATAAAGATATTGGAACCCTTTATTTTATTTTTGGAATTTGAGCTGGTATAGTAGGCTCAAGCTTAAGTATATTAATTCGTTTAGAATTAAGTCAACCCGGATTATTTTTAGAAGATGACCAAACCTATAATGTAATCGTAACCGCTCACGCATTTATTATAATTTTCTTTATAATTATACCTATTATAATTGGAGGATTTGGAAACTGACTGGTACCTTTAATACTAGGAGCCCCTGATATAGCCTTCCCTCGAATAAATAATATAAGATTCTGATTATTACCACCATCCTTAACCCTATTAATTTCAAGTAGTTTAGTTAATACAGGTGCAGGAACAGGTTGAACAGTTTACCCTCCATTAGCTAGAACATTAGGACATCCAGGAGCCTCAGTAGACCTAGCAATTTTTTCTCTTCATCTAGCAGGTGTTAGATCAATCTTAGGAGCAGTAAATTTTATTACAACAATCATTAATATACGCTCACAAGGATTAACATTTGAACGAATACCTTTATTTGTTTGATCTGTCTTAATTACAGCCATTCTTCTTCTCTTATCACTTCCTGTTTTAGCAGGAGCAATCACTATACTTTTAACAGACCGAAACTTAAATACATCTTTTTTTGACCCAGCAGGAGGAGGTGATCCAATCCTTTACCAACACTTATTTTGATTTTTTGGTCATCCTGAAGTTTATATTTTAATTTTACCTGGATTTGGTTTAATTTCCCATGTAATTAGTCAAGAAAGAGGAAAAAAAGAAACATTTGGAGTTTTAGGAATAATTTATGCCATACTAGCTATTGGACTTTTAGGATTTGTAGTTTGAGCTCATCATATATTTACTGTTGGCATAAATGTTGATACACGAGCCTATTTTACTTCAGCTACTATAATTATTGCAATTCCCACAGGAATTAAAATTTTTAGTTGATTAACAACCCTTTATGGAGCTAATATTGCATTTACACCTACTATTTTATGAAGACTAGGATTTGTATTTTTATTTACAATTGGTGGTTTAACTGGAGTAATCCTTGCTAACTCTTCAATTGATATTGTTCTTCATGACACCTATTATGTAGTTGCTCATTTTCATTATGTATTATCAATAGGAGCCGTATTCGCTATTATAGCAGGTTTTATCCAATGATACCCTTTATTTACAGGTATAGTTTTAAACGAAAAACTTTTAAAAATCCAATTTTTCGTTATATTCATTGGTGTTAATATAACATTCTTCCCTCAACATTTCCTTGGATTAAGAGGTATACCCCGACGATATAGAGATTATCCAGATGTATATACATCATGAAATGTAATTTCTTCTCTTGGAAGAATAATTTCTTTAATTGGTATTATTTTCCTTATTTACATTATATGAGAAAGATTTATA---ATACGTACACCCTCATTTTATAATTATTCTAATTCATCTCTAGAATGAACCCAAAAATTACCTCCTTCTGAACATTCCTACGCAGAATTACCTATAATCTTCAATTTAAATCTTCAAGAAAGTTCTTCTCCTTTAATAGAACAATTAAACTTTTTTCATGATCACTCTATCTTAATTCTATTAATCATTACATGTTCTATTTCCTATTTAATAATTGCAATAATCTCAAATAAAATTACT---------AATCGATTTCTTATAGAAAATCAACTAGTAGAAATAATTTGAACCGTAATTCCTGGAGTAATTCTAATTTTTATTGCTCTCCCTTCACTTCGAATTCTTTATTTACTTGATGAAGTAACCTCACCATCTTTAACATTAAAAACTATTGGACATCAATGATATTGATCTTATGAATATTCAGATTTCCTAAATGTAGAA---------TTTGACTCCTATATAATCCCCCCCTCAGAAAATAATTCCACAGAT---TTACGATTGTTAGAAGTAAATAACCGAATTGTCATCCCTTATAATACTCAAACCCGAATTTTAGTAACAGCTGCAGATGTACTACATTCTTGAGCTATACCTTCTTTAGGAGTAAAAGTAGATGCTAACCCAGGACGAATTAATCAAACCAGATTCTTAATTAATTACCCTGGATTATTTTATGGTCAATGTTCTGAAATTTGTGGTTCAGTACATTCCTTTATACCAATTGTTGTAGAAAGAACAAATAAAAATAGATTTATTAACTGACTATTATTAACACAAAACCATCCTTATCATTTAGTTAATGTAAGACCTTGACCTCTTACAGGTGCTATTTCCGCCTTATTTATAACATTAGGAATAATCGAATGATTTCAT---TCTCCAGAAAATTATTTAATAAAAATAGGATTTACTATAATATTATTAACAATAATTCAATGATGACGTGATGTTGTTCGAGAAAGAACTTTCCAAGGAAATCATTCTTTTAAAGTATCCCAAGGAATACGTTGAGGAATAATTCTATTCATTACATCAGAAATCTTTTTCTTTATCTCTTTTTTTTGGGCATTCTTCCATTCTAGTTTAGCCCCTAATATTGAATTAGGTATTATATGACCACCTAAAAGAATCACCCCCTTTAATCCACTACAAATTCCACTTTTAAATACTATCATCTTACTTTCATCAGGAGTTACTATTACATGAGCCCACCATGCTCTATTA------AAAAATAATTATTCCCAAACAATCCAAGCTATAGTAATTACAGTAATATTAGGTATTTATTTTACTGTTCTTCAAGGATATGAATATATAGAAGCTTCGTTTTCCATCGCAGACTCCATTTATGGAGCCTCATTCTTTATAGCTACAGGATTTCATGGCCTCCATGTAATCATTGGAACAATCTTTATTCTAATAATATTAATTCGGCAATATAAGCCCCACTTTTCCAACAACCATCATTTTGGCTTTGAAGCAGCTGCTTGATATTGACACTTTGTAGATGTAGTTTGATTATTTTTATATGTAACAATCTATTGATGAACAATATGAAATTTTGGTTCTCTTTTAGGATTATGTTTAGGTATTCAATTAATTACAGGTATTTTTCTAGCTATACATTACACTGCAGATATTAATATAGCATTTTCTAGTATTGTTCATATTTGTCGTGATGTTAATAATGGATGATTACTTCGAACTCTCCACGCCAATGGAGCCTCATTCTTTTTTATTTGTTTATATATTCATATTGGACGAGGTATATATTATGGATCTTTTAATTTATTA---TTTACTTGAACAATTGGAGTTATTATTTTATTTCTAGTAATAGGTACAGCATTTATAGGATATGTACTTCCCTGGGGCCAAATATCTTTTTGAGGAGCTACAGTTATTACAAACTTAGTTTCTGCTATTCCTTATTTAGGTACATCAATTGTACAATGAATTTGAGGAGGATTTGCCGTTGATAACGCCACTCTAACTCGATTTTTTACTTTCCATTTTATTTTACCTTTCATCGTTTTAGCTATAGTAATTATTCATTTATTATTTTTACACCAAACAGGATCATCTAATCCTTTAGGATTAAAAATAAACATTGATAAAATTTCCTTTCATCCTTACTTTTCTTTTAAAGATATTTTAGGATTTTTAATTATATTATTAATATTAATTTTATTAACACTAATAAATCCTTATATATTAAGAGACCCTGATAATTTTATTCCAGCAAATCCTTTAGTAACTCCAGTTCATATTCAACCAGAATGATATTTTTTATTTGCATATGCAATTTTACGATCAATTCCTAATAAATTAGGAGGAGTTATTTTTTTAGTCTTATCAATCGCAATTCTATTAATTTTACCATTTTCACAAAAAAATCTAATCAAGGGATATCAATTTTTTACTCTTAATAAATTCCTATTCTGAACTTATGTAAATTTAGTTATCCTTCTAACATGAATTGGAGCACGCCCTGTCGAAGACCCTTATATTTTTATTGGACAATCATTAACGTTAATCTATTTCTGCTATTTCCTAATTAACGTATCTTTTTTGTTAATGATAATTTTGGTTTTAGTCGGAGTAGCATTTTTAACATTATTGGAACGAAAAGTTTTAGGATATATTCAGATTCGGAAAGGACCAAATAAAATTGGTTTTATAGGAATTTTACAAGCTTTTAGAGACGCTATTAAATTATTTACTAAGGAAATAACGTATCCT---AATAAATCTAATTATTTAATATATTATTTTTGTCCTTTAATTTCTTTTTTGTTAATTTTTATTATTTGAAGAGTTACTCCATTTTTATATTACATAATAAAT------TTTAATTTTGGTTTTTTATTTTTTATAACTTGTTTAAGAATGGGAGTGTATGGGATTATAGTTGCAGGGTGATCTTCTAATTCTAGATATTCATTATTAGGGGGACTTCGTGCTGTAGCTCAAACGATTTCTTATGAGGTAAAATTAGCTTTTCTTATAATAAATGTTTTAATTTTATCTGGAAGATATAGATTTATAGATTTTTTTTTTGGTCAATATTATTGTTGATTTGTATTTACTAGATTT---CCTTTATTTTTATTATTATTTACTTCTATATTAGCTGAAACTAATCGTACTCCTTTTGATTTTGCTGAAGGGGAATCAGAATTAGTTTCTGGATTTAATATTGAATATAGAAGAGGAGGATTTGCGTTGATTTTTTTAGCTGAATATGCTAGAATTTTATTAATAAGATTAGTTTTAGTATTAATATATTTTGGGGGTAATACAAGAAGAATTTTATTTTTCTTTTTAGTAAGAGTTATTGGTTTCTCTTTTATTTGAGCTCGAGGTACTCTGCCACGTTATCGTTATGATAAATTAATAAATGTATGTTGAAAAGGTTTTTTACCTGTATCTTTATTATATTTGGTGTTTTATCTTAACTTCTTTAATTGCAAACCTAATAAT------------------------AGTCTTATGTATAATTTTATCAAAAAAATCACTCTATGACGAGAAAAATTTTCTCCTTTCGAATGTGGATTCGACCAAAAATCATCCTCCCGACTACCTTTTTCTCTACGATTTTTCCTTATTACTATCATTTTCTTAATTTTTGATGTAGAAATTGCTTTAATTTTACCAGCAATCAATAATATTTATTTATCCAACGCAAGTCAATGACTAGTCTTA---AACACTACATTTTTATTAATTTTATTAATTGGGTTATTTCATGAATGAAATCAAGGAATCTTAGAATGATCTTTATTAATTATATTATTAAGTTTAGAATTTGTAAGATTAATTATTTTTTTTATATTAATAATTAATTTATGAATATTTTCT---GAAAAATATGTTTTAATATATTATTTAACTTTTTGTGTTTGTGAAGGTGCTTTTGGATTATCCTTATTGGTAATATTTAGTTCGTTCAGTAGGTATGATTATGGTGTAAGAATTAATATATGTATTTATATAGATTGGATATCGAGATCTTTTATGAGATTTGTTTTATTGATTTCTTTTGTTGTAGTTTGTTACAGAATTAGTTATATAGGATCTGATAAATATTCAAGTATATTTATTATATTGGTTTTTTTTTTTGTTTTGTCGATGATATTATTGATTATTAGACCAAATTTAATTAGAATTTTGTTAGGATGAGATGGGTTAGGTTTGATTTCTTATTGTTTAGTTATTTATTACCAAAATATTAAGTCTTATAATGCTGGTATAGTTACTGCGATGACAAATCGTATTGGGGATGTAATAATTTTAATGGGTATTGCCTGAATAATTAATTTTGGTAGA---TGAAATTATTTATTTTATTTTAGCTGTGAT---------GATAAAGTGTTTTTTATTATTGGTGTATTTATAATGTTTGCTGCAATAACTAAAAGTGCTCAAATTCCTTTTTCTTCATGACTTCCTGCTGCAATAGCGGCTCCTACTCCTGTTTCTGCATTAGTTCATTCTTCTACTTTGGTTACTGCTGGTGTTTATTTATTGATTCGGTTTGATTAT---ATTATGAGGTATAGATTTATTTATAACTTT---TTGCTTTTGGTTTCTGTTTTAACTATATTTATGTCTGGATTGGGGGCTATTTTTGAGTATGATTTAAAGAAGATTATTGCTTTATCAACATTAAGACAGCTTGGATTAATAATTAGAACCTTATGTTTAGGTATAACAAGTTTTTGTTTTTTTCACTTATTAACCCATGCTTTATTTAAGTCTTTATTGATATCTAATTTATCTCTTTGTGGAATGCCTTTTTTAGCTGGTTTTTATTCAAAAGATTTA---ATTTTGGAA---AATATTTTAATGTTTAATATAAATGGGTTGATTTATTTTTTATATTTTTTTTCTACTATGTTAACGGTAGTTTATACATTTCGTTTAATTTATTTTAGAATAATTAATTCATTT---------------AAATTGATGAGCTATCAT------TGTTTTAATGATCAAGATTATTTAATATTATTCAGAATAATGGTAATAGTTTTTATAGTTATTATTGGGGGATCTATAATAATATGA------------------TTAATATTTATAGAT------TTTAGAGGTATTATTTTAGATTTATTTTCAAAGTTATTAACTTTAATGGTTTGTTTAGTGGGGTTTTTAAGAATAATTATAAACATA---ATCTTTTTCATTTTATTA---AATCCTCTCTCCTTAGGATTAGTTTTAATTTTACAAACTTTAGTTCTAAGTATTACTATAGGAACAATAACA---TCTTTTTGGTTCTTATATTTGTTAATCTTAGTTTTTATTGGAGGAATATTAGTTCTATTTATTTATGTAACCTCAATCTTTCCA---AAT---GAAAAATTTTCATTTAATCAAAATATATTTATTATT---------------------TTTTTAATTTCAGCTTTATTTATAATTTTTATATTATCATTTATAAAT------------------ATAAACTTCATAATAAATCTTAATCTAAATAATTTAGAAATCATTTTAAACATAAAATCAAATATAATT------------------ATAATTAGAACTATA---AAAATTTTTAGAACCCAAGGTAATTTAATTTTAATATTCCTAGTTAATTATTTATTTTATTGTATAGTAATTGTTATCAAAATAACAAACTTCTTTAAAGGA

Pediculus_capitis ATATCTTCTTTTGACCCTTCTACTTCTATTATTCTAGGA---TTAAAAGTA------AAATGATTTATTATTCTT---------------------TTTCCTTTCTTTTTTATAACTGGAAGTTATTATTTAATTCACTCAGGCTACCGTTTTTACGTGAGTTTTGTGTTAAATAATTTATTAATAAAACACTACATATCT---------------------------------ATTATTAGGTTATCTGTTTTTATAATAATTTTAACCCTTAATACAATTTCACTTATGCCATTGGTGTTGCCTTGCACTTCGCATTTAAGAGTTAATTTAGGACTTTGTTTACCTTTATGGATAAGAGGGGTTGTTTACTCT---TTAAAAAGCTCTATGCGAGGGTTTCTAGCTCATCTTCTTCCTTACGGGAGTCCAATTATACTAAGTCCGTTCTTAGTGGTAATCGAGTTGTTAAGAGTCTCGATTCGTCCTGTATCTCTAAGAGTTCGACTTCTAGCGAATATCACAGGAGGACATTTAATTATAAATCTTTTAGAAGAAGGCTTATCTTCAGCTGTACTTCTTGTACTTCCTTTCTCAATAGCAGCGTATGTTCTTCTCTTAGCTGCTGAGCTATTTGTCTCATTTATTCAGTCTTACGTTTTAAGTAAACTGGTTTCAATTTACTGAGAACTTTGTCCGTCGATGTGAACGTTGTATTATATAATTGTAATATTTGTATTATATTTTATATTAACT---ATGATTTATTTTATAAAAATTGATAATTCAAATTATTTAGAGAATTTTAAATTA------AAAAATAAAACAATCTCCAATCTTTCTATATTTTCTACAAATCATAAAGATATTGGATTTTTATACTTATGCTCTGGAGTTTGGTTTGGACTTTTAGGCTTAAGGTTAAGGTTAATAATCCGGTTAGAACTTTCTAGAACAGGCTTGCTTTTGTCTGATAGACACCTATATAACGTATTTGTTACTTCTCACGCTTTTGTAATGATTTTTTTTATAGTTATGCCTGTAATAATAGGCGGTTTTGCAAATTGATTAGTTCCTTCAATATTAGGGTCTCCAGATATAGCATTTCCTCGTATAAATAATATGAGTTATTGACTTCTCACACCCTCTGGGATTTTGCTTATTAGTAGCTCATTTGTTCAAGGTGGTGTGGGTACTGGCTGGACTGTTTATCCCCCTCTTAGGTCTCTAGAAGGCCAACCTTCTGTTTCAGTTGATTTAGCTATTTTAAGTCTTCATTTAGCAGGAGTGAGTTCGATTTTAGGATCAGTAAATTTTATTAGAACTATTTTTAACATATGGCCTCAATATTTTGGCTTAGTTCGACTGCCTTTATTTTGCTGGAGAGTGTTGGTAACAGCCTTTTTATTATTACTGTCACTTCCAGTTTTAGCTGGAGCTATTACAATGCTCTTAATAGACCGTAATTTCAATTGCTCATTTTTTGATCCTTTAGGGGGTGGTGATCCTGTTTTATACCAACATTTATTTTGATTTTTTGGACATCCTGAAGTTTATATTCTTATTCTTCCTGGATTTGGTCTTATCTCTCATATGGTGGTAGATTGTTGTGGAAAGAAAGAAGTTTTTGGGTCATTAGGAATGATTTACGCAATATCCGCTATTGGGGCTTTAGGTTTTGTAGTTTGAGCACATCACATGTTTACAGTTGGATTAGATGTGGATAGACGGGCTTATTTTACTAGCGCTACTATAACAATTGCAATTCCAACGGGAGTGAAAGTCTTTAGGTGATTAGGCACTTTGTTTGGCCCAAAATTAAAAAGGAGAATTAGCTTGTTGTGATCTTTAGGATTTATTTTCCTTTTTACAATTGGAGGTTTAACAGGCATTGTTCTTTCTAACTCATCCGTAGATGTTTCACTACATGACACTTATTATGTAGTTGCTCACTTTCATTACGTTTTATCTATGGGTGCTGTATTTGCTATTTTTGGCGCTTGAAACCACTGATTCTCACTAGGGACTGGACTTAAACTTCGTAAGTCTTTTATAAATGTTCACTTTTGGTTAAGATTTGTGGGAGTGAATTTGACTTTCTTTCCTCAGCACTTTCTCGGGTTAGCTGGGATGCCTCGACGTTACTCAGACTATCCTGACGTTTACCTGAGGTGAAACAAAATTTCTTCAATAGGAAGGCTAATTACTACTTTGGGTGTTGTAATCTTTCTTTTAGCTCTTATAGAAAGGTTTTCTAATCCTCAAAAAATTGTATTTAGAGATGCTAGATTGCAAGACCTTCCGCGTCTAATGGGTATACCAGCAAGAATGCACTCTCATTTTACTCTTACATTTACTAGAGTATGTGGATTCCAAGATAGAAATTCTCCTTTAATAGTTTTTGTGTGTGATACTTATGACCTTGTGTCTATTGTTTGTGTGGGGGTGATCTCCTTAGTAATGTACGTGGCTGTTAGTTTCTTTTTTATAAAATCTTGA---------AATTACTATTTTATAGGTCTTGAAAGATTGGAGATTGTTTGAGTTATCTTACCCTCACTCTCTTTAGCAGGGTTAATTTTACCGTCACTTCATTGTTTATACTTAATAGACGAGGTTCTTTCTCCCGCTATGAGATTAAAAGTGGTCGGACATCAGTGATTTTGGTCTTATGAGTACGGGGATTGAGAAAATATTGAA---------TTTGATTCATATATGATAAAATTAGAGGAGCTTGACTCGTCATGTCCTTTTCGACTTTTGGAGGCTGATTTAAGCGTGTTTATCCCTTATTTGACTGAAGTGCGTGCTATTGTAACATCTGCCGATGTTATTCATTCTTGAGCAATTCCTATAATAGGAGTAAAAGTAGACGCTATTCCTGGGCGTTTAAACCATGCACTTATTTACTCATTTAAAATTGGCACATCTTATGGTCAGTGTTCTGAGATTTGTGGTGCTTATCACAGGTTTATGCCGATTAAAGTCACTACTCTTCCAAAAGAAGACTTTATAAAATGAGTTAAAGATTTAAAATTTCACCCATTTCATCTTGTTGATGTAAGACCTTGACCTATTTTTTTAAGATTTTCTCTTTTATTTTCAGCGTCCATAACATTGTGTTGAATTAACGGGCTTTATTCTTTTTATATTTTAATAATTAGAATTGTTGTTTCATCTTTAATTGTTTCTTTTTGGTGACGAGACGTTACTCGAGAGGCTACTTTTCAGGGTAAACATACAATAGAAGTAATTGCTGGATTGCGTTTAGGAATGCTTATGTTTATTGCTTCAGAGGTAATGTTTTTCTTTTCGTTTTTTTATGCTCTGTTTTTTCTTTCTTTAAGGCCTGACGTGTCATTGGGACTTCTTTACCCTCCTGTGGGTGTTAGCCCTGTAGGCGTTTTAGGAGTTCCTCTTTTAAATTCTATTTTATTACTCTCTAGCGGTGTGTCTATTACTTGAGCTCATTATGAGCTTTTA------AGGAAAAATATTTCTTCTAGGCTTATCGGCTTATTAATCACTTTAATTTTAGGTCTAGTGTTTCTAATATTTCAGGCTGTTGAATATAAAACAAGCTCTTTTACTATGGCTGATAGAAGTTTTGGCTCAGTGTTTTTTCTAATAACCGGCTTCCACGGAGCACATGTTTGTGTGGGAGTTGTGTTTATTACAATTAGAACCATTCGGCTTTACTTAAATCACTATAATAATAATCATCACTTAGGGCTTGAGCTAGCTGCATGATACTGACACTTTGTCGACGTAGTGTGGTTATTTTTATACCTAACTCTTTACTGATGAATACTCTTGAAATTTTGGGTCTTATTAGGCTTGTTTCTTTCAATTCAGATTTTAAGAGGTCTTTTTCTGGCTTCTCATTATGAGGCTTCTACTAAT---TCGTTTTGAAGTGTTATTTTAATTGATTTTGATGTAAATAGAGGGTGGTTGATTCGTAGTTTTCATGCTAACGGCGCTTCTTTTTTCTTCATTCTTGTCTACGTTCATATTTGGCGTGGTTTATGATTTGGTTGTTTTACACAAAAA---TATGTTTGATTTTCAGGAATTTCTATTCTTCTTCTTATAATAGCAGCAGCTTTTATGGGGTATGTTCTTCCTTGAGGTCAAATATCTTTTTGAGGAGCGACTGTAATTACTAATCTTTTAAGTGCTATTCCTATTGTTGGAAGAGATTTGGTTATTTGAGTGTGAGGAGGGTTTTCAGTTAGACATCCTACTTTAGAGCGGCTGTTTACTCTTCACTTTCTTTTACCGTTTGTCTTATTGGGGTTTGTTATAGCTCACATTATTCTCCTCCACCAACACGGTTCTAGAAATCCTTTAGGATTGGATTTGGATAGTGATAAAGTTTATTTTTATCCTTACTTTTATCTAAAAGATATTTTAGGAGGTTTTGTGTGTTTATTTTTATTTGTTTTGATTTGCATTTATTCGCCGGACTTCTTCATAGACCCGGATAATTTTGTTGAATCAAACCCGATAATTACACCTCCACATATTCAACCAGAGTGGTACTTTCTATTTGCATATGCAATTTTACGGAGTGTACCTAACAAGTTAGGAGGAGTTGTAGCTTTGCTTCTAAGAATTTTATCTCTGTCTTTAATTAGCATA---------GGAAGCTCTGTCTCAAGTCGCTTTAGAATAAGGCGAATGATTTTAACTTATTCTTTTACGAGAGTTTTTGTTATGCTCTCATGACTTGGCTCTCTTCCTGCTGAGTATCCTTTTACTCTGCTAAGTCAAGTTGTAAGAGTAATTTATTTCATTCAAGTAATTCTATTTATGCAGTCAGTATTTATTGTTTTATCACTTTTAATTTGTGTTGCTTATTTCTCCTTGTTTGAACGTAAACTTCTTAGGTTAGAGCAAATCCGGCTTGGACCAAATAAAGTAGGACCAATTGGTATTCTTCAACCTTTAAGAGATGCTCCCAAATTATTATCCAAGACTATTTGTCCGCAG---AGAGAG------TCATGAGAGTTATTCATTATGCCGTTTATTACATTTATGTTAAGCGTTTCCTGATGGTACCCGCTCTATTTTCCAAAAACTTTATGA------GAATCAAATAACTCACTTTTAATCCTTATTTTTATCTCAAGAGTAAGAGTTTATGCATTAATTTTTACAGGGTCACTGCCAAAATCAAAATATTCGGCTCTAGGAAGACTGCGTGCAATTACTCTTTCTATTTCTTTTGAATTAGTTTTTTCCACTGCTATGCTAAGTATGGCTGTAGTTTTTAATTCTTTTAGCATTAAATTTATAGCAACCAATCAGAGC---GTGCCTAATATTATCTCAATAATT---GTAGTTGGAATTTTAGTATGAACCTCACTAATTGCTGAATGCGGACGAACTCCTTTTGATTTACCAGAAAGAGAGTCTGAATTAGTAAGAGGTTTTAACGTAGAGTACGGAGGTAGGCGTTATGTTTTACTTTATTTAAGAGAAAGATTGTTACTTACCATTTCATCTATTATTATAAGCATTTTATATACCTGC---GGGTATAATCCATTAGTGGTTTTAACATGAATTAGAATTTCT------------ATTGTTATACGAGCTAGAGCTCCTCGCATTCGGTACGATAAATGCATAATATTCGGGTGAGAATTTTCAATTCCTTTAATCTTAATATTTATGAGATTTACTATTTTGATTATTTTGGCTATTATTTTAGCT------------------------TTAGTTACTTTGGTTTTTGTATCTCAAAGTCCTAAATTAACTCTAGATAGGACGCCCTATGAGTGCGGTGTAATGCCTTTTAGAATAAGAACTCTGTCTACACACATTCATTTTTATGTTGTAAGTGTGGTATTTCTTATTTTTGATGTAGAATTAGTAGCTACTTTGCCTGTTGTTACTTCA------AGGCTATTAGAGAAAGATTGATTGTCAATT---TGACTTTTAATTCCACTTATTCTCACCTTAGGGTTGCTTTTAGAACTTCATTATGGAAGTTTAGATTGAAAAATTATATCTTCTCTTATTTCTCTTGAATTAAGTTGGTGCTGGGTTTACGTAATTATACACATTGCACTTTCTGAA---AGGTTAGACACTCTTCTAAGATCAGAGGTTTTAAGTGTTATTGTTTGCGAGAGAGTTGTAGGACTTTCTTTACTTATTAGGCTAACTTATGGGTGAGGAAGGACTGGTTTCAGGGGGGTAGAAATTATTATTGTACTGGATAATTTAAGCCTGACTTTTTTGCTTATAGTATTAACAATTAGGTCGCTTGTTATAGCTTATAGAAACTATTATATAGCAGGTCATAATCTTGGCGGTGATTTTTACGTTTCCATGGTCTTATTTATTGTAAGAATATTACTTCTATCTCTTAGAGGCTCAATGTTCTGATCTTTTATTGGGTGGGACGGTTTAGGAATAATAAGTTTAGTTTTAATTTTATTTAATAAAAGATGGAGTTCTCAAAAATCGGGAGTGATTACTTTCCTAATGAACCGATTAGGTGATTCTTTTATAATTATTTGCTCGTCATACTTAAGAGTGTGAGGGATA---TGTGAA---------------------------------GTTTATTGGTTTTGAATTTTAACTTCTTTGTATTTAATTGGAGGGGCTTCAAAAAGAGCTCAATTTCCATTTTCTAGCTGATTGCCGGAAGCTATGGCAGCCCCTACTCCCGTGAGTAGGTTAGTTCATTCTTCAACATTAGTAACTGCTGGTATTTATGTTTTAGCTCGTTATGGAAGA---ATAATCGATAGTTTTTACATT------------CTTACCTACTTATCTTCTATTTCTATTATTATTTCGGGTGTTTCAGCTCTCTGAAGGAGAGATTTAAAGAAAGTTGTAGCTTATTCAACTCTTTCTCACATTAGCTTAATGCTTTTTTACTTATCTGAAGGAAGGGTGGAAGGAGCTTTAATCCACATGTTGACGCACTCTGTGTTTAAAAGGCTGTTATTTTCTTGCCTTTCAATGGCCGGACTGCCGTTTCTTTCGGGAGGTTATTCAAAAGAGGTG---TTA---------CTTATTTTAAGTTTAAATAGATCAATTATGAAGCTTATTATATTTCTTACAGCTGTAATTTTCACTAGAGGTTATTCGTTTCGCATTATTTACTTACTTTCTAGAAAT---------------------ATTAATATAACACAAAAT------------ATTGCAGTAAGGAGGTTGTTTAGCAGCCCCCTTAAACTGAGGCAAATGCTTAACGTTCTA---------ATTTCAGCTTGAATTTCCTCAAGTCCTGGCTACTTATCTAAACTC------AGAAGGCAG---AGAGTTAATTTAGAGGGTAAAATTATAATACTCTTTATAATTCTATTTGGTATGGTTGCTATGTTTTTTACGTTT---CTTATTTTAGTAGGCTCT---GATCTTTTGCTGAAGCTTTTTTCTCTAGCTATAACTGTTTTTACAGTCGGACTGCTTATTCTTCTAAACTCTTACTCTTCATGGTCTTGGTTGCTTTTATGGTTAGGGATTTTGGGTGGTTTAATTGTCTCACTTTCTATGGCTTTTATTGTAACGCCA---AAA---ATTAATTCCTCAAAGGACTGGTCTAGAAGATTAAGA---------------------TTTATAAGAGCGTTTTGAATTTTAACTTCTGTTATTCTATCTGCTATA------------------------ATATGAAAAGTAGAATTTAAAGAATGATCTGACTTGTACTCTACAGAG---AGTGTAAGAGAA------------------ACTTATAATTCACTA---CTTATAGATCTTAAGGTTTATACAGCTGTGGTTATTCTTTTAATT------TACATTTTAATGCTTCCTGTTATAGAAGTTTTAACGTCACCTTATAGACGT

Heterodoxus_macropus CTATCAATTTTTGATCCTTGTTCAAGAATTTTAAATATT---TTTAATTTT------AACTGAATAGTTATAAGA---------------------TTATGTTTATTAATTCCTTTAAATTCATTTTGAAAAGTTCCAAGAATTTTATTAACATTTTTTGATTATATAAAAAATCTAATGAAATCTATATTTACTAAT---------------------------AAAGTAAATTTTCAAATTTCAAGGATTTTTTTTTTAATTTTAGTGTTTAATATTTTAGGAATTTTTTGTTTTACATTTTCTGTAACTAGACATTTAGTTATTAATTTATCTTTAGGATTTTCTATTTGAGTAGGAACTTTATTATATAGTTCAATTTATAAA---TTAAGTGATTTTTTAGCCCATTTGACACCAATAGGGTGTCCTATGGTTTTAGTTCCTTTTATAGTAGTAATTGAATTTATTAGAATAATAATTCGACCTATTACTTTATCTTTACGACTAATAGCTAATATATTAGCTGGACATATAATTTTATCATTAATTAGAACAGGAGTAAGGTTAATACTTTCTTTATTTATTCCTTCAGGAATATTGTTGTTACTAGGATTTTATTTATTTGAAATTGGAGTAGCAATTATTCAAGCTTACGTTTTTTCAATTCTTCTTTCTTTATATTGAGAAATATTTCCATATCTTTGAATATGAATTTTCATATTACTTTTATCTGTTTTTATTTTGTTTTTTATG---AAATTATTTTTTTCAAATTTAATTTCTAAACGAAATAATAGATATTGTAATAAA------CATTATAAATTTGATAAAAAATTAGAATTTTATTCTTCTAATCACAAAAATATTGGTATTTTATATATAATTCTAGGAAGATGATCTGGACTATTAGGATTTAGATTGAGAATAATAATTCGATTAGAATTATCTGACTCAGAAATATATTTATTTAACCCACATATTTACAATGTTGTTGTTACATCACATGCATTTTTAATAATTTTTTTCTTTATTATACCATTTATAATTGGAGGTTTTGCTAATTGATTAGTACCTATTATAAATGGTAGGCCTGATATATCGTTCCCTCGAATAAACAATATAAGGTTTTGATTATTACCTCCTTCTTTAATCTTTATATTATGTAGAATAATACTAGATGGAGGTTCTGGGACAGGTTGAACTGTTTACCCTCCATTGTCTTCTTTAACTGGACATGCAGGTATGTCTGTAGATATATTAATTTTTTCTCTTCATCTTGCTGGGATTAGATCAATTATAGGGGCTATCAATTTTATTACTACTATTTTTAACATA---GTTTTCTTTAAAAATTTATCAATAATAAGATTATTTAATTGATCAGTATTAATTACTGCTTTTTTATTACTTTTATCTTTACCAGTATTAGCAGGTGCTATTACAATATTATTATTTGATCGAAATTTTAATTCAAGATTTTTTGATCCTATTGGAGGAGGAGACCCTATTTTATATCAACACTTATTTTGATTTTTTGGACACCCTGAAGTTTATATTTTAATTTTACCAGGATTCGGTTTAATTTCTCATATTATTGTTCAAGAAAGAGGAAAATGTGAAACTTTCGGAGTTTTAGGAATAATTTATGCTATATTATCTATTGGAATTCTTGGATTTATTGTATGAGCTCATCATATATTTACTATTGGTATGGATGTTGATACTCGGGCATATTTTACTTCAGCGACTATAATTATTGCAATTCCTACTGGAATTAAAATTTTCAGATGGTTATCTACTTTTTTTGGTAGAAAAATAAAATTTAATTCTTCAGAATTATGAAGAATGGGTTTCGTTTTCTTATTTACTGTAGGTGGTTTAACAGGGGTAGTTTTAGCAAACTCTTCTATTGACATTGTTCTTCATGACACCTATTATGTTGTTGCCCATTTTCACTATGTGTTATCAATAGGAGCAGTATTTGCGGTATTTTCAGCTTTTACACATTGATTTCCATTATTTTTTGGTGTAAAAATAAGAAATGCTTTAATAATTCTTCATTTTTGAATTACTTTTTTAGGAGTTAATTTAACTTTTTTCCCTCAACATTTTTTAGGACTTAGAGGTATGCCTCGACGTTATATTTGTTATCCCGATTTTTATTATTCTTGAAATTTTTATTCAAGAATTGGATCAATAATTACTTCGGTAAGATTATTAATATTTGTTTTCATAATTTTTTATAGATTTTTTGAAAATAAAAAATTATTATTCTACTCTTTTAGAATAAATTCTATTGAGTGAATGCTAGGAACTCCACCTTCTGCTCATTCTTTAAATGAAAGACCTGTCTTAATTGAATTTAATCTTTCTGATGGATGTTCATTAATTATAGAAAATATAGTTGCATTTCATGATTTCACTTTAATAATTCTTTTATTTATTACAACAGTAGTACTAATAATATTAATTTCTATTATAATTACTAATTTAGTA---------AATCGATTTTTAATTTATAATGAAGTATTAGAATTTATTTGAACAGTGATTCCTAGGTTTATTTTATTGATTATTGCATTACCTTCTTTAAAAATTCTTTATTTAGTAGATGAATTACTTAATCCTGAAGTTACAGTCAAAGTCATTGGAAATCAATGATATTGATCATATCAATATTCAGATTTATTTAATATTGAA---------TTTGATTCTTACATAAAGAAATGAGAAGGGCTA------TCTGAT---TTTAAATATTTGGATGTAGATAATCGAACTGTACTTCCTGTAGACACTAATATTCGAATAATTATTACATCTTCTGATGTAATTCACTCATGAACAATCCCTAGATTAGGGGTTAAATTAGATGCCAATCCTGGACGATTAAATCAATTAAATATTTTAGGTAATCGATTAGGATTATTTTTTGGTCAATGTTCAGAAATTTGTGGAATTTTACATTCATTTATACCAATTTGTGTAGAAATAGTAAAACCAGAGTGATTCTTAAAATGATTATATAAAAATGGTTTTTTTTTATTTCATATTGTAGATGAGAGGCCTTGACCTTTATTTCTTTCATTTAGAGTTTTTTTAAATATATTAAGAGCTTTAGTTTATTTAAAG---TTTCATATTTTAATTTATATGTTACTAAGTAATATATTAAGAATTTTAATTTTTTACATGTGAATACGAGATATAATTTCTGAAAGAACTATGCAAGGAATACATACTTTAAAAGTTCAAAATGGAATTAAAATAGGAATAGTATTATTTATTACATCTGAGGTTATATTTTTTTTTTCTTTCTTCTGAAGATTGGGATATTATATAGTAAGTCATGAATATATTCTT---AGAAATTGACCTCTTTTAGGTATTATAAGATTAAATCCATCTACAGTGCCATTATTGGGTACAATAATTTTATTAAGATCTGGAGTATCTGTAACATGATGCCATAATGAGCTTATATTAAGAGAAGGAAATCTCAGAAGAATAAAAAATTCATTATTAATTACAGTAATTCTTGGAATAGTATTTGCAGCTCTTCAAATATGAGAATATTTTATATCTACTTTTACTATAAGAGATGGTGTATATGGGTCGTTGTTCTATATAATAACTGGATTTCATGGATTTCATGTTATTGTTGGAACAATTTTTTTATTTATTATTTTTTTACGATTAAAAAATTATCATTTTTCTAGACACCATCATTTAGGATTTCAAGCAGCAGCTTGATATTGACATTTTGTTGATGTTGTTTGAATTTTTTTATATATTATGTTATATTGAGGTTATATATGAAATTTTGGAAGTTTATTAGGGCTATGTCTCTTTATTCAAATTGGATCAGGTTTATTTTTATCACTTCATTATAATTCAAATGTTGAGTTAGCTTTTAGAAGTGTTATTTATATAATAAATGATGTTAATCATGGATGAATTTTACGTGTAATTCATGCAAATGGGGTAACAATAATATTTATTTTTATGTATATTCATATTGCTCGTGGACTTTACTATAAATCTTATAAACTAACT---TTAGTTTGATTAGTTGGGATCTTAATCCTACTATTAACAATAGGAACTGCATTTTTAGGATATGTTCTTCCTTGGGGGCAAATATCATTTTGAGGTGCTATAGTAATTACTAATTTAATTAGAACTATTCCTTATTTAGGAGTAACATTAGTTGAATGGGTGTGGGGAGGATTCTCTGTTAGAGAGCCAACTTTAACTCGATTTTTTTCATTTCATTTTATTTTACCTTTTGTAATTCTAGGGGCATCTGCTTTACATATTATTTTTTTACATAAGTATTTAAGATCGAACCCCCTTGGTTTA---CCTAAGACTGATATAATTTCATTTCACCCATTTTTTACTGTTAAAGATATCTTGGGTGTAGTATTATTTTTATTTAGTTTATTATTTTTATCTTTAACAGAGCCTTATAAGTTTATAGACCCAGATAATTTCATTTTAGCAAACTCTATAGTTACTCCAGTTCACATTCAACCAGAATGATACTTTTTATTTGCTTATTCTATTTTACGGGCTGTTCCTAATAAATTAGGAGGGGTTATTGGTTTATTAATGTCTATTCTAGTATTAGCATTATTTTTATTTTCTAATAAAAGAAAATCTCAAGAAAGGGTATATTAC---------AAATCATTCTGTTGAGTTCAATTCACTATTTTTATATTATTAACATGAACTGGAAGATTGCCAGTAGAATCTCCTTTTTTAGAAATTGGACAATGTTTATCAGTAATATATTTTTTAAATATATTTTTATTATTACAACATTTTATCCTTGTTATTATAATACTTCTTACTGTTGCATTTTTTACTTTATTAGAACGAAAAATTTTAGGCTATATTCATTTCCGAAAAGGTCCAAATAAAGTTTTATTAAAAGGAGTTTTGCAACCTATTGTTGATGCTATAAAATTAATTACAAAGGATGATTCTCCAATC---ATTTATAGAAATATTTTTTTGTATTATATTTCTCCTATATTTAGATTTATTATAAGAATAATTATTTGAATAATTCTTCCTTTACAATTTATTATTTTTAAT------TGAGTTAATAGATTTTTAATTTTATTTATATTACTAGGAATAGGAGTATATAGAATATTTTTGTCAGGTTGATCTTCAAATTCTAAGTATGCTTATTTAGGAAGACTTCGAGCTGTAAGGCAATCAATTTCTTATGAAATTTTAATAAGGATATTATTTATTGCTTTAATAATAACAACAAAAGGTATAAGAATTTATTATATTTTAAAATTTGACCCT---------TTGGTATTTTTTTTATTT---CCTTTTTTTATTGCATATTTATTTATTGGCTTAGCAGAATTAAATCGATCACCTTTTGACTTATCTGAAGGAGAGAGAGAATTAGTAGCAGGCTATACAGTAGAATATGGAGGAATTATATACACAATAATTTTTTTAAGAGAAAATATTATAATTATATTTTTTTGTTACATAGGTTCATTATTTTTTTTT------TATATTAATAGGACTATGAGAATTATTTTTTCAATAATAATAATTTATTTAGTTTGTTTAATTCGAGGAATTCTTCCACGAATTCGATATGACCATTTAATAATATTTTGTTGAAAAATTATGTTACCTTTAATAGTAATTTTTGTCAATCTTTTACTTAGAAGACTAATTATTTTAGTTTTATAC------------------------TATCTTAGAGTATTATTTATAGATAATAAGAATATTGTTGAAGATGGAAAAAAAGAGTTTGAATGTGGTTTTCGAGCAGAAAATTTATCTCGGTTACCTTTTTCAATGCAATTTTTTAGAATCGCTTTAGTTTTTCTTATTTTTGATGTTGAATTAATTATTATTTTACCTTATATTTTTAATTTTAATCACATATATATA---------------------TTTAGAATTATAATAATTTTGTTATATTTAGGAACCCTTTTAGAATGAATAGAAGGAAGATTAGATTGATATATAATAATTACTTTATTAAGATTTGAATTAATTGGTTTGGTAAATTTTATGATGATTAATTTTTTATTTCCC------------AATATAAAATTGATTTTAATTATAGTAACATTTTTAATGTTAGAAAGAGTATTAATATTAATTCTCTATACATGTTTGATTCGTGAATTTGGAATAGAAAGATTTATTGAATGTAACTTTAATTTTATTTTTGATAAATTTTCTATAATCTTTTTATTTATAGTTTTAATTATTTCAAAAAATGTGTTAAAATACTCTTATTTTTATTTTGTTGGGACTGTATGAACTTTACGATTTATTGGAATTTTAATTTTTTTTATTGTATCAATATTATGATTAATTATATCTTATGATATATTTACTTTTATTGTAGGATGAGATATGTTAGGTGTATCTTCTTTTTTATTAATTTTATACTATAATTCTTATAAATCGAAAAAAAGAAGATTAATTACTTATATTAGAAATCGGTTTGGTGATGGATTTTTTATATTAGCTATAGTATTAGCTAGACCTTTATTTAGC---GAATTTTTTTTATTTAAAGCTCAT---------------------------TATTTTCTTCCAATTTTAGTATTTTGTACTAGAATTACAAAAAGAGCTCAATTTCCATTTTCTAGATGACTACCTGAAGCTATAGCAGCCCCAACTCCTGTATCAACTTTAGTACATTCATCTACTCTAGTTACAGCTGGATTTTATTTTTTATTTCGATTTCAAGAACTTTGAATTAATAATATTTATGCTTTAAATTTA---TTGTTATTTATTTCATTGTTTACGATAACTTTAGCCAGAAGAGCTGCTTTAATAGAATATGATTTGAAAAAAGTTATTGCTCTTTCAACTTTAAGACAGATTAGTTTTATATTTTTTAGTTTAAGTTTGAAGTTAACAACTTTAGCTTTTTTTCATATAGTTATACATGCATTTTTTAAAGCAGCAACTTTTTGTCAAATGTCATTGTCAGGATTTTTATTTCTTTCAGGGTTTTATTCAAAAGATTTA---ATTTATAAATCTTTTTTAGCCTATAATAATTTAAATTATTATTTTATGATTGTTTTTCTTATTTCAATTGTTTTAACTATATTATATTGTTTACGAATATGTTTAATAATAATAAATTTT---------------------TTAAAAATAACTTTTTAT---------TTAAATTTTAAAGATATGAATATTTTATTACCAGTATTAGAATTAATATTACTTTCAATTGTTTCAGGTTCTGTTTTAATATGA---------------------------TATTTA------TGTGAATCAGTAATAATTCCATCAAAATTAGAATTTATGAATTATTTATTATTTATAATTATATTAATTTCTATTTTATCTAGAATTTCATTTTTCTATCTGAGAAATCTTAGAAAGATTATAAGATTAATTTTAGTTCTAATTAATATTATTTCAGTCTCAGGATTAATTATGATTTATTCATTATCTTCTTTTGAAGGAATAATTTTGGTAATTGTTTTTTTAACAGGACTATTTATTTTAATATCTTATTTAGTAAGTGTAACTCCT---GAA---AATCCAAAATTTGTAAATTTCTTTTATAAAGAAAAAGCTCAATCCAAAAATTTTTATTTCATGAAATGAATTCTTTTATTATCAATTTTGTTATTTATTTTATTA------------------GCCTTATATTGAGAGTTTAATAGACAATCTTTATGATTTGAAGTTAAATTTAGA---AAAGAAAATTTT------------------AATTTTTCTTTTTTA---TCTCCATTTAAACACATTTCTATTTATATAGTTAATATCTTAATTTTTTTACTTTTATTTATTGTATGAATATATATACGATTTACTTATACAAAAAAGGGA

Ibidoecus_bisignatus TTTTCCATTTTTGATCCGTGTGTAAGATATACAAGC------TTTCAATTT------AAATGATTGATTTCATTA---------------------AGTTGAATAATAGTTATAACAGTTAAATTTATGAATTTAGATTAT---GTAAAATTATATTATTTGGTGGTTTCTAATTTTTTTATTTCATCATCAAAAACTTTATTAAAAAGAAGATAT------AAAGTTGTATCTGTTAAGATCGTCTCCGTTTTTACAATAATTTTAATATGTAATCAGTTAAGTATAGTCCCATTTGTTTTTGGTCCTACTAGACATTTATCATTTAATTCGGCTGTTGCTTTATCAAGATGGTTAGCAGGGATTATTACGATA---CTTTTAATTTCATTTAAAGATTCTGTTTCACATTTTGTTCCTTTAGGAAGACCAATATTTTTAACTCCTTTTTTATTTATTGTAGAGGTGATCAGTTGTTTAATTCGACCTGTAGCGTTAAGAGTTCGGTTAATATCAAATATAATGGCTGGACATATTATTATTGTATTATTAAGAAACCTTATTTGTAGGTTAAACTCTTAT---------TATTTGATCCCAATTGAATCATTCATTTTTTTATTTGAGTTATGTATTTCTATTGTTCAAGCTTATGTTTTTTCAAGTCTCTTAGCTTTATACTATAAATTATTTCCATGTACGTGATTATTAATTTTTTTTTTAGCTCTATGTGTAATTGTTTTTTGTCTTTCTTTGATAAATTTCTTTGTTCTAATTGATGAAAGTACTTTTAAAAAAACTCCAAAGAAA------GATAAGCAAAAACGAGAGGCATTAACCTTTTATTCGACTAATCATAAAGACATTGGGATACTTTATTTGATTTTTGGAATCTGATCAGGATTATTAGGTTATAGTATAAGACTGATTATTCGAATAGAACTAAGTCAGATAACTAATTATATTAATGATGGTCACATCTACAACGTAATCGTTACATCTCATGCTTTTTTAATAATTTTTTTTATAATTATACCAATTATAATTGGAGGTTTTGCTAATTGGTTAGTTCCTTTAATAATTGGCTCTCCAGATATAGCATTTCCTCGAATAAATAATATTAGATTTTGATTACTTATTCCTTCTTTATTATTTTTATTAATAAGAATTTTTATAGGAGAAGGAACTGGAACAGGATGAACCGTATACCCCCCCTTATCAAGT---------CAAGCATCAATTTCTGTAGACATTTCAATTTTTTCTTTACACTTAGCAGGTTTAAGTTCAATTTTGGGAGCTATTAATTTTATTTGTACTATTATAAATATATGATTATCA------TCTATATTTTTACTACCCTTATTTTGTTGATCAATTTTAATTACTGCTTTTTTATTATTACTTTCCTTACCGGTTCTTGCAGGAGCAATTACTATGTTACTTCTAGATCGGAATATTAATTGTTCTTTCTTTGATCCTATGGGAGGAGGAGATCCAATTTTATACCAACATTTATTTTGATTTTTTGGACATCCTGAAGTATATATTTTAATTCTTCCTGGTTTCGGTCTAATTTCTCATATTATTTGCGAAGAAAGAGGAAAAAAAGAGGTGTTTGGTTCTTTAGGAATAATTTATGCCATGTTATCTATTGGTATTTTAGGATTTGTTGTATGGGCACATCATATGTTTACTGTAGGTATAGATGTGGACAGACGAGCTTACTTCACCGGAGCAACTATAATTATTGCAGTTCCTACTGGAATTAAAGTATTTAGATGAATATCCACCTTATTTGCAAGGAATATTAATTGGTCAGTATCTTCATTATGAAGGTTAGGATTTGTCTTTCTATTTACAATCGGAGGTCTTACGGGGGTAATACTAGCTAACTCATCAATTGATATTGCTCTTCACGATACTTACTATGTTGTAGCTCATTTTCATTATGTTTTATCCATGGGTGCTATAGTAGCATTTATGGCTAGGTTATTCCATTGGTTCCCATTAATTTTTGGAGTTTATCTTAATTCAAAATTTTTAAAAATTCATTTTTTTGTAACTTTTATTAGGGTAAACATAATTTTTTTTCCTCAACATTTCTTAGGATTAGCAGGAATACCTCGTCGGTATATAGATTACCCAGATATATTTAGTTCATGAAATGTAATTTCTTCTTTAGGTTCTACTTTATCTATTATCAGTTTATTTATAATGATGTTCTTAATTTTTGAAAGGCTAATTTCAAAACGACTGGTTGTGTTTAGATGTAAAATTCCTATTTCTATTGAATGAGTAAATGGATTTCCTCCTAGAAATCATTGTAACGAAATAGTACCACAATTAACTACTCTTTGTTTTCAAGATAGAAATTCTCCTTTAATAATACATATTAACCATCTTCATGACCATATTATGGTTGTTATTATTATGATTATTTCTATTGTTATATATGTATTATTGACAATTGTTATAAACCCTTGCTCA---------AATCGATTCTTTTTCGGTAGAGAAGTGTTGGAATTAATTTGAACTTTGGCTCCTAGAATTGTATTAGCAATTTTAGCTATTCCATCACTTCATATTTTATACTTAATAGATGAATTAAAA---CCTATGATTAGTATTAAGTCTATTGGTCACCAGTGGTATTGGTCATATGAGTATGGAGATTTATGTAGAATTGAG---------TTTGACTCATATATGATTATAGAACAAGATTTAGAATTAGGTATA---ATGCGATTGTTAGAAGTAGATAATCGGACAGTAATTCCTGTAGGAATAGAAATTCGGATACTAATTACATCTACTGATGTAATTCACTCATGAACTATTCCTACACTGGGGGTAAAAATAGATGGAGTTCCTGGTCGTTTGAACCAAATTTATTTATCAAGTAATATTTGTGGTTTAATGTATGGTCAGTGTTCAGAAATTTGTGGAAGTTTTCATTCATTTATACCTATTTGTTTAGAAGTTTTATCTGAGTCTCGATTTATATCTTGGTTGAATAATTATGGGTTTCATCCTTTTCATATTGTTTCTATTAGTCCATGACCTATTTTATGTTCTTTTTCTATTATATCTTTTGTGATTAATTCTTTGTATTATATAAATAAATTTTTGACTTTAGATTTACTTTTAGAGTCATTATTGTCTTTAATTTTAGTTATTTTTTGTTGATGGCGTGATGTCATTCGAGAAAGAACGTTTCAGGGTTTTCACATGAAAAAAGTGTGTTTTGGTTTATATATAGGAGTTTCAATATTTATTATTTCAGAAGTAATATTTTTCTTTTCTTTTTTTTTTGGTTATTTTTTTTCTAGTTTAGTCCCAGACGTAGAAATTGGATGTTCATGACCACCAGTGGGAGTTCAATCTTTAAGGTTCATAGATGTTCCATTATTAAATACAATAATTCTTCTATCTAGAGGAATTTCTATTACTTGGTCCCACCATTCTTTATTG------GAAAATAATTTTACTAACTGCTTATTAGGGATAATTTTCACTGTTATCTTAGGTTTGATTTTTACTTTTTTTCAATTTATAGAGTATTTAGAGTGTTCTTTTTCTATAGCTGATAGGGTTTATGGGTCACTTTTTTATATTTCTACAGGCTTTCATGGAATTCATGTAATTGTAGGCACATTATTTATTATTGTTTCTTTTATTCGAATAATGAAATATCATTTTTCCATTCACCATCATTTGGGGTTTGAATTTTCTATTTGATATTGACATTTTGTAGACGTAGTGTGATTGTTCTTATTTTTAAGAGTATATTACTTATTTATGTGAAATTTTGGTTCATTATTGGGGATATGTTTGATAGTTCAAATTTTTTCTGGTTTGTTTCTTTCTATACACTATAATACTTCTATTGATGATGCTTTTAATAGAGTTTTATCTACATGTAATGATGTTAACTTAGGGTGATTGATTCGTTATATTCATGCTAATGGAGCCTCAATATTTTTTATACTTGTATACTGTCATATTGGTCGAGGTTTATATTTTGGGAGTTTCAATATAACC---TTGACTTGATTTTCAGGAGTGATTATTCTTTTATTATTAATGGGTACCTCATTTTTAGGTTACGTTTTACCTTGGGGACAGATATCTTTTTGAGGAGCAACTGTTATTACTAACTTAGTGAGAACTATTCCTTATGTAGGAGATCAGTTAGTTTATTGGTTATGAGGAGGGTTTTCTGTTAGTGAACCTACACTAAATCGATTTTTTTCTATTCATTTTATTTTGCCGTTTGTTTTAATGATAGTGGTCTTAGTTCATATTTTCTCCCTTCATAAAAGAGGAAGAAGGAATCCTTTGGGAATTTCTCCTAATTGTTTAAAAATTTCTTTTCATCCTTATTTTTGAAATAAAGACGTTTTGGGATTTGTTGTTGTATTAATTATTTTTACCGTTACATTAATTTTTCTCCCTGATGTATTCATAGATCCTGATAATTTTTCTGTAGCAAATCCTATATCAACTCCTGCCCATATTCAACCGGAGTGATATTTTTTATTTGCTTACGCAATTCTTCGGTCTATTCCTACTAAATTAGGAGGGGTAGTAGCATTAGTATTTTCTATTGTAATTTTATTTATTATTCCCTTTATTAGAAGAGGAAAAAATAAAAGACTTAATTTTTAT------CATAAAATAATTGTTTTAATACAAGTTTCTAATTTTCTTTTATTAACGTGATTAGGAGCCATACCAGTAGAATTTCCATTTTTAATAATAAGAAAAATTTTTTCTTCCATGTATTTTATTTTTATAATTTTATTACTACAATTCTTGTTAATTATTGTTGGATTATTGTTATCTGTTGCTTTTTTTTCTTTGTTTGAACGAAAAATATTAAGAGTAATTCAATTTCGAAAAGGTCCAAATAAAGTAGGGTTAATAGGTTTTTTTCAACCTTTTTCTGATGCAATTAAATTATTATTTAAAAGTAATGAAATACCA------AATTTAAGGAATATATTTTACTATTTTGCACCAATAGTATTTTTTATTTTGTCAATTTTAATTTGAATTAGTATTCCCAGAAAGTGGAATTTATTCAAT------TTCTCTTCTAGTTTCATTTTCGTTATATTTCTCTACGGAATTCCCATTTATAGAATAATTTTTATAAGATGAATTTCTAATTCTAAATATTCCAAAATTGGCTCAATTCGTTCTGTAGCACAATCTATTTCTTATGAGATTATTTTGTCTTCATGTTTATTATTCTTGATAATAATAGTATATTCTTCTTCTATAAATTTATTATATTTTTATCAATCATACGTGTGACTTTTGTATCCTTGTTTT---CCTATTTTTTTTATTATATTTATTTCTATTTTAGCTGAAAGTAATCGTTCTCCATTTGATCTAACAGAAGGAGAAAGAGAGTTAGTATCGGGGATTTTTGTTGAATTGGGAGGAGTTTGATATATTTTAATTTTTTTAGGAGAAAACTTGTATTTGTTATTTTCTTCATTTTTAATATCTTTTAGAATATTAGGAAATTCATTAATTGTTTTAAAGTTTGTAATTATTACTATATTAATA------------GTTTGAATCCGAGGAACTGTTCCTCGGATTCGCTATGATAAAATAATAGATTTGTGTTGAATTAGAATAATACCAATCTGTATATCTTTTATTTCTATTGTAATCACAGTATTATTAAATATTGTTCTATGT------------------------TTTATTTCTATTATATTTTTAAAAAATGACATAAAA---CAAGACAGAAATGAGAGTTTTGAATGTGGAATGGAAACATTTTTTAATTTTAATTCATTTTATTGCTTGCATTTTTTTTTAATTGGAGTTTTATTTTTAGTTTTTGATATAGAAATTATTATTTGTATTCCAATAATTTTTTTA------AATTTGGAAATGATTAAAATATTATTATAT---TGGAGATTAATAATAATTGTATTAATTGTTGGTTATTATTTAGAGTTAGCTATTGGAACTTTGAACTGGAAAATAATCACAATTTTGATTTCATTAGAAATATTA---GTTTGAACGATTTTTTTGTTTATGATTTATATAGAAAAATCTATTATTTCTATTTCTTTGTTTCTATTGTTTTCATGTATGTTGATTTGTGAAGGAGTAGTTGGTTTAACTATTTTATCAAAGTTATATAAAAACTATAGCTCTTTTAATCAAGTATCATTCTCATTATCAATAATTGTAGATAAATACTCTTTGATTTTTATAATTATAGTTACAATAATCAGAACCATTGTAATAATTTATTCTATCTATTATATAATAGAAGAAAAAATGAAAAAAAAGTTCTTTTTATCGATATTTTTTTTTATTCTGTCGATAATAATCCTTTCATTTTCTGCTAATATTTTTTGATTGATAGTAGGGTGGGATGGTTTAGGTCTCTCTTCATTTATTCTTATTATATATTTTCAAAATTGAAATAGATTCAATAGATCTATAACTACATTTATATGTAATCGATTTGGTGACTTGTTTATACTTATTAGTATTGCTTTAATAGTTAACTTTTTATCT------AATTTTTGTTTATCTTTTTTGAATTCA---------AACTTGTTTAGTTTTTTGTTTTGTTTTTTTATTATTGTTTGTGCTATAACTAAAAGTGCTCAAGTTCCCTTTTCTGTTTGACTCCCTTTAGCCATAGCAGCTCCTACACCTGTATCATCATTGGTTCATTCTTCTACATTGATTACAGCTGGAGTTTTTTTATGCATTCGATTTAGAAGT---ATTCTTATGGAAGTTCACTTTCTTTTTATT---TTATCATATATTTCATCTTTTACTTTTATCATATCTGGCCTGTCAGCAATATATGAATACGATTTAAAAAAAATTATTGCTCTATCAACTCTTTCTCATATAGCATTGATTTTTTTTTTTTTAAGTATAAATAGATTTGAGTCTTCTATGATTCATTTGATTACTCATGCTATCTTTAAATCTTCTTTGATTCCTATTTTTAGAATAATAGGAATTTTATTTTTATCAGGATTTTATTCTAAAGAGTTA---ATGGGAATA---ACTATGGTTTGATATAATACAAAAAACTGTTATATTGGATTATATTTTATTGCTGTAGTTTTAACTTGTATATATTCTACACGTTTGTTATATATTTTAATTAATAACAAAATTAAT------------AGTAACATATTATATAAA---------------GAAGAGAATCAATCAATTTATTATATTTTATATGTTAGAAGAAGAATTTCGATTTTTTTAGGAGGATTTCTATTAAAA---------TTAATTACATCTTTTTGGAAATTT------CAAAAAGATGTAATCTTAATAAAATTGTTATTTTGATTGATTCTCTTCTTTATATTTATTGGAATGTTAATTTTGTCAGTTTTGATC---TTATTTTTTATCTCATCT---TCTTCTGTTATGATATTTTTTTTAATAATCATTAGTTTTTTAATATTTGGAGTTTTGATGTGATCAGTTTCTTTTAATTTTTTTTTATCTTTAATATTTTTTTTGATGACATTAGGAGGATTGTTAATTTTATTTTCGTTTATTCCAATGTTG------------------GAATTTAATTTCAGAATGAAAAATTCA---------------------------TTTATGAAATTAGAATTATTGTTTTATTTATTTATTATAATTTTTGTA------------------------TGGATAAAAATTAATTCTTTGGATTTAGTCAATTTTTATTTAAATTTT---GATGATATTATA------------------AAGTATTATATAAAA---------------ATAGACTTCACTGGTTTGTCGTTGTTTTTAATATTAATTATATTTATAATATTATTTATTTTGGACTCGATCGTCAGAACCTCTGAGGGT

Lepidopsocid_sp TTTTCATCTTTTGATCCTTTAACTAATATCTTCAAT------TTACCTTTA------AATTGACTAAGATCTTTA---------------------TTATTTATTATATTTATCCCTTCATTGTATTGAATTTTACCTTCACGAATACAATTAATTTGAAATTTAATTATTAAAACATTACATAATGAATTTAAAACACTTTTAAAAAATTCATCA---AATTTTGAAAGAACAATAATTTTTATTTCAATTTTTAGATTTGTTTTAATTAATAATTTTTTAGGCTTATTTCCATATATTTTTACTAGAACAAGTCAATTAGTTTTAACTTTAACTCTTTCACTACCTTTGTGATTAAGATTTATAATTTACGGT---TGATTAAATAAAACCAATCACATATTTGCTCATTTAGTTCCAAATGGAACACCAGGTATTCTTATACCATTTATAGTATGCATTGAAACTATTAGAAATATTATTCGTCCGGGAACATTAGCAGTACGATTGACAGCAAACATAATTGCTGGACATTTAATCTTAACTTTATTAGGAAATACAGGATCAAATATAACAATTTTTTTAGTAACCTTCTTAATTATTATTCAAATTGCATTATTAATATTAGAAATAGCTGTAGCATTTATTCAATCTTATGTAATTGCAATTTTAATTACTCTTTATTCGAGAATAATACCTATACCTTGATTATCATTATTTTTTCTTTTTCTATTAACCTTATTAATAACTAATTGT---ATTAATTATTTT------------TATTTTCAACCTAATATTTTTTTTTCAAAA------AAATATTTAAATATTAAAAAAAACATTATATTTTCTACAAATCATAAAGATATTGGTACTTTATATTTTCTATTAGGAATTTGAGCAGGAATAGTTGGAACTAGTATAAGAATTTTAATTCGATTTGAATTAGGTCAACCAGGTTTATTTTTAGAAGATGATCAAATTTATAATGTTATTGTAACAGCTCATGCATTTATTATAATTTTTTTTATAATTATACCTATTATAATTGGTGGATTTGGTAATTGATTAATTCCTTTAATATTAAGAGCACCTGATATAGCATTTCCTCGAATAAATAATATAAGTTTTTGATTATTACCCCCTTCATTAACTTTATTATTAATAAGTAGAATAACAAATGTTGGTGCTGGAACCGGATGAACAGTTTACCCTCCTTTATCAGCAGCAGTCGCTCATGCAGGTGCATCAGTTGATTTAGCAATTTTTTCTTTACATTTAGCTGGAATTAGATCAATTTTGGGAGCAGTAAATTTTATTTCAACAATTATTAATATACGATCTAACGGATTAACTTTAGAACGATTACCACTATTTGTATGATCCGTTTTCTTAACAGCAATTTTATTATTATTATCTTTACCTGTATTAGCAGGGGCAATTACTATATTATTAACAGATCGAAATTTAAATACCTCATTTTTTGATCCTGCAGGTGGAGGAGATCCTATTCTTTATCAACATTTATTTTGATTTTTTGGTCATCCAGAAGTTTACATTTTAATTTTACCAGGATTTGGTATTATTTCTCACGTTATTAGACAAGAAAGAGGTAAAAAAGAAACATTTGGAGTTTTAGGTATAATTTATGCTATAATAGCAATTGGATTATTAGGTTTTGTAATATGAGCACATCACATATTTACAGTAGGTATAGATGTAGATACTCGAGCTTATTTTACATCTGCAACAATAATTATTGCTATTCCAACAGGTATTAAAATTTTTAGTTGATTAGCAACTTTACATGGGTCAAAAATATTTTTTTCTCCATCATCTTTATGATCTTTAGGATTCGTATTTTTATTTACCATTGGTGGTTTAACAGGTGTTATTTTAGCTAATTCTTCTATCGATATTGCTCTACACGATACATATTATGTAGTAGCACATTCCCATTATGTACTATCTATAGGAGCTGTATTTGCTATTATAGCTGGGTTTATTCAATGATTTCCTCTTCTTACTGGATTAACTTTAAATAATAATTGATTAAAAATTCAATTTATAATTATATTTATTGGAGTAAATATAACTTTTTTTCCTCAACATTTTTTAGGTCTTATAGGAATACCACGACGATATAGAGATTATCCAGATATTTATACTTCATGAAATATAATTTCATCTTTGGGATCTACAATTTCCTTAATTGGTATTATATTTTTCATTTTTATTATATGAGAAAGTTTTATTTCTAATCGAAAGCCTATTTTTTCTATACATATATCTTCTTCAATTGAATGATTACAAAAATATCCTCCTTCTGAACATTCTTATAATGAATTACCAATTATTTATAATATTAATTTACAAGAAAGTGCTTCACCATTAATAGAACAACTAATTTTCTTTCATGATCATTCATTATTAATTATTACTATAATTACTGTAATAGTCTCCTATATTATAGCATCTTTATTTTTTAATTCATTTACT---------AATCGATTTTTATTAGAAAATCAAACAATTGAAATAATTTGAACTATTATTCCAGGAATCGTATTAATTTTCATTGCTTTACCTTCTTTACGACTTCTTTATTTATTAGACGAAACTAAAATACCATCTATTACTTTAAAAACAATTGGTCATCAATGATACTGAAGATATGAATATTCAGATTTCAATAATATTGAA---------TTTGATTCATTTATAATTCCTTCAAATGAAAATTTTAATTCAGAT---TTTCGACTTTTAGAAGTAAATAATCGAACAATTTTACCATTCAAAACACAAATTCGTATTTTAGTTACAGCAGCTGATGTTTTACATTCATGAGCTATACCATCTTTAGGTGTAAAAATTGATGCTAATCCTGGTCGATTAAATCAAACATCTTTAAATATTAATCGTCCGGGATTATTTTATGGGCAATGTTCTGAAATTTGTGGAGCTGTTCACTCATTTATACCTATTGTTATTGAAAGAGTTCATAAAAATAGATTTATTAATTGACTTAATTTACATTCTAATCACCCTTATCATTTAGTAGATGTAAGTCCATGACCATTAACAGGTGCAATTGGAACTATAATTTTAACATCAGGTGTAGTTAAATGATTCCAT---ATATTTAATATATTTTTATTCTTTATTGGAATAATAATTATTTTATTAACTATATTTCAATGATGACGAGATGTAGTACGAGAAAGTACCTTTCAAGGTAAGCATTCAATTTCAGTATCTAATGGAATACGATGAGGAATAATTTTATTTATTACATCCGAAGTATTTTTTTTTATCTCATTTTTTTGAGCATTTTTTCATAGAAGATTATCACCATCTATCGAAATTGGAATAATTTGACCTCCTAAGGGAATCCAACCTTTTAATCCATTTCAAATTCCTTTTCTTAATACTGTAATTTTAATTTCTTCAGGAATTACCATTACATGAGCTCACCACTCACTATTA------AAAAATAATAATAGTCAGACCATTCAAAGATTAGTAATTACCATTATTTTAGGATTATATTTTACTATTCTTCAAGGAATTGAATATTGAGAAGCACCATTTTCAATTGCTGATGCTATCTATGGATCTTCATTCTTTATAGCAACAGGTTTTCATGGAATTCATGTAATAATTGGAACTACATTTATTTTAATAATACTTATTCGACAAATAAATAATCATTTTTCTAATTATCATCATTTTGGATTTGAAGCAGCAGCATGATATTGACATTTTGTTGATATTGTATGATTATTTCTTTATATTTCAATTTACTGATGAACTTGATGAAATTTTGGATCTTTATTAGGATTATGTTTGATTATTCAAATTTCTTCAGGTCTATTTTTAGCAATACATTATTCAGCCCATATTGACTTAGCCTTTTCAAGAATAATTCACATTTGCCGGGATGTAAATAATGGTTGAATTTTACGAACAATTCATGCAAACGGAGCTTCTTTTTTTTTCATTTGCTTATATTTACATGTAGGACGAGGAATTTATTATAGTTCTTATAATTTACAT---ATAACCTGATTTATTGGAATTTTAATTTTATTATTAACTATAGCAACTGCCTTTGTAGGATATGTTTTACCTTGAGGACAAATATCTTTTTGAGGAGCTACGGTTATTACTAACTTATTATCCGCCATTCCGTACTTAGGACAAATATTAGTTCAATGAATTTGAGGTGGATTTGCAGTTGATAATGCCACATTAATTCGATTTTTTACATTCCATTTTATTTTACCATTCATTATTTTAGCAATATCAATTATTCACTTATTATTTTTACATCAAACAGGTTCTAATAATCCATTAGGAATTAAAATAAATATTGATAAAATTCCATTTCATCCTTTTTTTTCTATTAAAGATTTATTTGGATATATAATTATACTTCTAATTTTAATTTCATTAAATTTTAGTATACCTTACATTTTAGGAGACCCAGATAACTTTACTCCAGCAAATCCACTATCCACCCCTGTTCACATTCAACCAGAATGATATTTTTTATTTGCTTATGCTATTTTACGATCAATCCCCAATAAATTAGGAGGTGTTTTAGCTTTATTATTTTCAATTTTAATTTTATATATTTTACCCTTATTTAAA---AATAAATTTCGATCTACACAATTTTATCCAATTAATAAAATTTTATTCTGATCATTCACAACAATTTTTATTTTATTAACGTGGGCAGGAGCTAAACCAGTCGAAGACCCATTTATTTTTACAAGTCAAATTTTGACAGTATTATACTTTTCATTTTTTATTATTAATATTAATTTTATCTTATTATTAATTGGAATTCTTGTAGGAGTAGCATTTTTAACTTTATTGGAACGAAAAGTATTAGGATATATTCAAATTCGTAAAGGCCCTAATAAATTAGGTTTTATTGGATTATTACAACCTTTTAGTGATGCAATTAAGTTGTTCACTAAAGAACAAGTATTTCCC---AATATAAGAAATTATTATCCTTATTATTTTTCTCCTGTATTTTTATTTTTTTTGTCATTAATTTCTTGAATGATTATACCTTATTTTTGGGTTTTGAAAAAT------TTAAGATTAGGTTTATTATTTTTTATGGTATGTTTAAGTTTGGGGGTTTATGGTATCATAATTAGAGGGTGAAGATCTTTTAGATTATATGCTTTGTTAGGAAGCCTTCGTTCTGTAGCTCAAACAATTTCTTATGAAGTAAGATTGTCTTTTATTTTAATTAGTATATTAATATTAATTGGTAATTATAGATTTATAAGTTTCTATTTTTATCAAAAATATTTATATTTATATATATTTGGTTTT---CCTTTATGTTTAATATGAATGGTATCTATGTTAGCTGAAGTTAATCGAACACCTTTTGACTTTGCTGAAGCGGAGTCTGAATTAGTTTCAGGATTTAATATTGAATATGGAATAGGTGGATTTGCATTAATTTTTTTAGCTGAATATTCTAGAATTTTTTTAATAAGAATAGTATTTACATGTTTTTTTTTAGGTGCTGATATACTAAGATTATTTTTTTTTTTTAAGCTATTATTTGTTTCTTTTTTTTTTATTTGAGTACGAGGTAGATTACCACGATTTCGTTATGATAAGTTGATAAATTTATGTTGAAAAAGATTTTTATCTTTATCTCTTTTTATTTTAAGTGTTACATTTACTATTTTTCTTTCAATATTATTAAAT------------------------TTTATAGCTACATTTCTTTCTTATAAAACAATTGAAGATCAAGAAAAAATATCCCCATTTGAATGTGGATTTGATCCTATAAAATCTTCGCGTATACCATTTTCTCTTCGTTTTTTTTTAATTACCATTATTTTTCTAATTTTTGATGTAGAAATCACTTTTATTTTACCTATAATTTTAAATTTTAATAAATCAAATATATTGTTATGAATTTTTATT---ATCTTTGTATTCATTTTAATTTTAATTTTAGGTTTATTACATGAATGAAATCAAGGAGCTTTAGAATGATCAATTTTGATAATATTAATTAGTTTAGAATTTATAAGATTAATTTTATTTCTTTTAATTTATTTTAGTTTATTTAATTTAAATTTAGAAGGATATTTTTTAATAATTTTTTTGACTTTTTGTGTTTGTGAAGGTGTAATTGGGTTATCCTTACTAGTAGGAATAATTCGTTCTCATGGAAATGATTTATCTGTAAATATCCAAATTGTAATTTTATTTGACTGAATATCTATATTTTTTTTAAGTTTAGTAACTTTTATTTCTTGTTTAATTTCAAATTATAGAAATTCCTATATATTAGGAGATAATAATTCAAAATTGTTTATATTTTTAATTATTATGTTTGTATTTTCAATAATATTAATAATTGTAAGACCTAATATAATTAGAATTTTATTAGGTTGAGATGGTTTGGGATTAGTATCTTATATTTTAGTAATTTATTATCAAAATGTAAAATCTTATAATGCTGGTATATTAACTGTATTATCAAATCGAATTGGTGATATTATAATTTTAATTTGTATTGGTTGACTATTTAGATTTGGTAGT---TGAAATTATTATTTTTATATTAATATAATTACTACCGATGATTATTTAATTAGATTAATTGGTTGATTTATTATTATTGCAGGTATAACTAAAAGAGCTCAAATTCCTTTTTCTTCTTGATTACCGGCTGCTATAGCAGCCCCTACTCCTGTATCTGCATTGGTTCATTCTTCTACATTAGTTACAGCGGGGGTTTATTTATTAATTCGATTTTTTCCT---TTATTTAATATTAGAAAATTTAGAATGATT---TTATTATTCATTTCTGGTTTAACTATATTTATATCTGGTTTAGGAGCTAATTTTGAATTTGATTTAAAAAAAATTATTGCTTTATCAACTTTAAGTCAATTAGGATTAATAATAGGAAGTTTATCTATAGGATTAACTAATTTTTGTTTTTTTCATTTATTAAGACATGCTTTATTTAAAGCATTATTAGTTTCTAACTTATCTTTATGTGGAATTCCATTTTTATCTGGATTTTATTCTAAAGATTTA---ATTTTAGAA---TTAATTTCTATAAGAGAAATTAATTTAATTTCTTATTTTTTTTTTTTTGCATCTACAGGATTTACAATTAGTTATTCTTTTCGATTATTTAGTTTTTTAATATTAGGAAACTTT---------------AATTTATTTAGATTCCAT------TGTATTGAAGATAAAGATTATATTATAATAAAATCTATATTTGGTTTATTTTTGGGATCTTTATTTGGAGGAAGAATATTAATATGA------------------TTGATTTTTCCATTT------AGAAAGATAATTTTGTTACCTTTATTAATAAAATTTATAGTTTTGTTTTTAATTTTAATTGGTACTTTATTATCCACCTTTTCTATT---TTATTTTTATTTATAAAA---CATCCATTATCAGCAGGTTTAATTTTAATTATATCAACCATTTTAGTTGCCCTTATAACAGCCTATATACTTCAAACTTTTTGATTTTCGTATATTTTAACCTTAATTCTAATTGGAGGAATATTAATTTTATTTATTTATATAATTAGTTTATCTCCT---AAC---CAAAAATTTATAATTTCATCTATTCTTTTTTTAATTCCCTTA---------------TTTTTAATTATTCCAATAATAATAAATATAATTGATCCAATAATTTTA------------------------------------------ATAGAATTATCACAAAAAATTAATGATCAA---ATTAACTGAAAT------------------TTACCCCAATCAATT---AAATTATTTAATACAAATTCAAGAATTTTAACTATTATTAGAATTAATTATTTATTCTTAATTATAATTATTGTTACAAAAATTACATCTAGATTAAAAGGA

Liposcelis_bostrychophila TTTTCAATTTTTGACCCATCTAGTAAGCTCGTGTCT---------------------AATTGATTTATTTGTCTA---------------------ATCTTATTAATTTTTAAA------------------GTGAAAACACCATCAAGGCTTGTAGATGTCTCTTTAAGTAATTTTCTT---TCTGTGTTCATTAAAGAAATGAAAATTATTAAA------ATTCACTTATTTTTTTTCCTTTTGCCTATCTTTATTTTGATTTTGGCTTTCAACATCTCTGGGATCTTTCCTTTTACATTCACCCTAACTAGGCATATAAGAATAACTTTTTCTTTAGCCTTTCCTATATGGTTAAGACTGATAATTATAGGA---TGATTGAAA---TTCAATAGTATATTCGCCCACCTTGTCCCTTTAGGGTGTCCCACAGTTTTAATACCGTTTATGGTATTAATCGAAACAATTAGTCTAATTATTCGTCCGCTAACTTTAGCTGTTCGTCTCGCAGCCAATATAATTGCTGGGCATATGATTTTATCGTTAATCAGGATAAGGGCCTTCAACTCCTCTATTGTATTCGTATCC---TCACTATTAGCTGAGTCTATAATCTTGCTTTTAGAATTGGCAGTCGCAATAATTCAGCCCTATGTCTTCTTCATCCTATTAACCCTTTATAGCCAGATAGCCCCGTTATGATGACTAGGAAGACTAATCTTTATATTTTTTGGTTTAACCATCTTATTCCAA---ATTTTATACTAT------------ACAACTGAGATCTCAACCCTTGAGAAATTC------AATGATAAGATCACTTTTTTCAATTTTCTTTTATCGACAAATCATAAGGATATCGGATCGCTATATTTTATATTTGGGGTTTGATCGGGTCTATTAGGCCTAAGACTTAGGCTTTTGATACGAGTAGAACTTTCCTTCAATTCTAGGAGGCTGAGCTCTTCT---GTGTTTAATAGACTAATCACCTCACATGCTTTTCTAATAATTTTTTTTTTTATTATACCTATATTAATTGGGGGCTTTTCTAATTGGATAATCCCACTTTTGATTAGATCACCTGATATAGCCTTCCCACGGTTGAATAATTTAAGATTTTGATTTCTCCCCCCTTCTTTACTTTTAATTTCATTCAGAATAATCGTTGGACCAGGCGCAGGAACCGGGTGGACAGCCTACCCTCCTCTGTCAGCTATCGAAGCTCATTCAGGTTTCAGTGTAGATCTAGTAATTTTTTCCCTACATTTGGCTGGAATTAGTTCCATCCTGGGAGCCATCAATTTTATCACTACTTCAATTAATTTATGAATCGAGCCCCGGCAATTCGAGTTACTGCCTTTATTTAGATGGTCTGTGTTAATCACAGCATTTCTTTTGCTTCTTTCTCTTCCCGTTTTGGCTGGAGCAATTACTATACTTTTATTTGATCGTAATTTAAGAACTTCATTCTTTGATCCATCTGGAGGGGGGGACCCAATTCTCTTTCAACATTTATTTTGATTTTTTGGACACCCTGAAGTCTATATTTTAATTTTACCAGGATTTGGGCTAATCTCTCACATCATCTCTCAAGAGAGA---ATAAAAGATGTATTTGGCAGATTAGGCATGATCTATGCCATGCTCTCAATCGGAGCTCTAGGTTTCATCGTATGAGCCCACCACATATTCACTGTGGGCATGGATGTGGATAGACGAGCATATTTTACCTCTGCAACAATAATTATCGCGATCCCTACCGGAGTTAAAGTTTTCTCTTGGTTAACTACTGTTTATGGGAGAACAGTTACTCCTTCTTCTTCGACTTTATGAAGTCTAGGATTTATTTACTTATTCACCATCGGGGGTTTAACTGGGATTATCCTATCGAATTCAAGAATTGATGTTATTCTACATGATAGATACTATGTTGTTGCCCACTTCCACTATGTTCTTTCCATGGGGGCTGTATTTTCAATCTTTAGAGGATTGAATTTCTGATTGCCTCTTTTTCTGGGGGGGTCAGTTAATGAATTAAAAAACAAAGTTCACTTCTTCTTAACTTTTATCGGAGTTAATCTCACATTTTTCCCCCAACACTTTCTAGGTCTATCTGGTCTCCCACGACGATACTCGGACTACCCAGACCACTACACATATCTAAATCTGATTTCTTCGATTGGTTCATGAATCAGAATAATTAGCATTATCTGGTTAATTACTCTTATTTTTGATGGTGTAATAAAAAAAAATTCTGTTATTTTTATATTAACCCCTTCTTCAAGAATCGAATGAATCGAGGGGCAGCCACCTAAATTTCACACATGTTTATACTCGCCTCAAATTTTTAATTTCATATTATTAGAAAGGAGGGGGCCTGTAATAGAACAAATGAGGGAGTTTCATGATCATGCTATAATGATTCTTTTCCTCATCGTTTCTTTTTTAACTATTGTTTTTTGAGTTACTTTAACTAATAAAGGATTA---------AACCTTAACATTTTGACAAGAGAAGTTCTTGAGATATTTTGGTCATCTCTGCCTGTTTTCATCTTATTAATTCTTGCTATTCCCTCTATTCAAGTTCTGTTTATAATAGAAGAAGTTATCTCCCCCCTGATAACAATTAAAATCATAGGCAATCAATGATTTTGAACGTACGAATATAGTGACTTAACTAATGTAAAA---------TTTGATTCGGTAATTAGTAAGACTAACATT------------------TTCCGTCTTCTTGATGTTAATAAAGCTCTAATTCTACCAATTACCACCCATGTCCGTCTTCTCTTATCGTCAAATGACGTGATCCACTCTTGAACTCTGCCCTCATACGGGTTAAAAATTGACGCCAATCCAGGCCGCCTAAATATAGGCTCTCTTTACAGCTATCGTTCGGGCTACTTTTATGGTCAGTGTTCAGAAATCTGTGGGGTTGACCACTCATTTATACCTATTAAAGTTGGTTTCACTTCTCTGGACTGGTTTAAGAATTTTATAAAGAGGGTAAAACTGAGAGACTTTCATCTAGTAGATATCAGGCCGTGGCCTTTAATAATAAGCTTGACTACAGCTAACACCATTCTGTCTCTATACATCAAAATGAATTTTTTGTCATGTCTATTATTGATGACCATTTCTATAATT---------TTTGTATTTTTCCTATGATCACGAGATATCATACGAGAGAGAACCTTTCAGGGAATACACCCTTTAAAAGTTCAGCTTTCACTTAAATACGGAATAATCCTGTTCATTACTTCTGAAGTTATATTTTTCTTGTCCTTTTTCTGAACATTTTTACATTCGGCCCTTAGCCCCACAAATGAAATTGGTAACTCTTGACCAAGATGGGGTGTTGAGCCTATTAATCCCTTTGGAATCCCTTTGCTGAACACTCTAGTCTTAGTTTCATCAGGGGTTTCTATTACATATTCTCACCATAGAATATTA------AATCAAAATTTTAATTTTACCATTCTCTGAGTAGTAATTACAGTTCTTTTGGGAGGGTATTTTACGATTCTACAGCTCATGGAATATATGACATCATCATTTTCTATCATGGACTCAGTTTATGGCTCAATTTTTTTTATCTCTACAGGCTTTCATGGCATCCATGTTCTAGTTGGCACTCTGATGATTCTATATTCCCTCATTCGTCTATTCAGCTTTCAATTCAGCTCTGCTCATCATTTAATGTTTGAATTCTCTTGTTGATACTGACACTTTGTTGATTTAATCTGACTTTTTCTTTTTCTATCAATTTATTGATGGTACTTCTGAAACTTGGGGTCGCTTCTCGGGCTATGTCTTTCTATTCAAATCTTAACAGGAGTGTTTTTAACAATATTCTTTAAAGCAGATCTCAGGCAATCTTTTACCAGAGTAGTAAGAATTATAAATAATATTAATAACGGGTGAATCATCCGGTTTATTCATTCCACTGGGGCATCCATATTTTTTATTATCTGCTACGCCCATGTCGGAAAAGCACTATTTTTCTCTTCATTTTACTTTTGA---AAGGTTTGAGTTTCTGGACTAGTGTTAATTCTTTTACTCATAATAGAGGCTTTTCTAGGCTATGTCTTACCATGAGGACAGATATCATTTTGAGGAGCTACAGTAATCACAAATTTAATTTCTGTTATTCCTTACTTTGGCCCCCTTGCTGTTCAGTGACTGTGGGGGGGCTTTAATGTTGGTGATCCCACACTAACACGATTCCTATCATTTCATTTTATCATTCCATTTATCATGATTGCTATAAGAGGAGTACATTTAATTCTTCTTCATGAGACTGGGTCATCAAACCCTCTAGGCATGCCTTTGAACATAGATAAAGTTAGATTCAGAAAATTTTTTATTATTAAAGACCTAGTTACCCTAGCTCTGGTTTTGTTAGGGCTAATTTTACTTAGAACAATATCTCCTTTTATATTTATAGACCCTGAAAATTTTCTTAAAGCTAACCCAATGGTGACACCCATCCATATCCAGCCTGAGTGATACTTTCTGTTCGCCTATGCAATCTTACGATCTGTTCCTAATAAACTTGGGGGGGTGTTAATGTTAGCTTTATCCATCATTATTATTTTAATTCTCCCTCTCTTATCTAAAAACAAGATAAAAGGACTTAAGTTTAGATTTTTG---AAATGACTTCTTTACTTCCACTTTGGATCATTCTCTATTTTAACATGACTGGGCATGCAACCTGTTGAAGATCCCTTCATTTACTTAGGTAAAGTTTACTCAGTTCTTTATTTCATTTTTTACTTCCTCTTTATCAATTTAATCTTACATATCGTCTTGATACTTTTAAGAGTAGCTCTGTTTACACTATTTGAACGTAAAATTCTAGGGCTCATTCAACTTCGTAAAGGGCCTTGTAAAGTAGGGCCTTTAGGCCTTTTACAACCTTTCTCAGATGCTTTAAAACTTTTTTCAAAATTTTCCTCAGCTCCA---ATCAAAGGAAACTTAATACTTTATTACTTCACTCCTCTGTACTTTCTTATTCTTTCATTAGTCTTTTTTTTGAATAAACCATTCTTATCAACTTCTTTT---------TTTACTTTATCAATTTTAGTTTTACTCTTTCTATATACCACCAGGGTTTACACTACTTTAGTAACCGGATGATCTTCTAACTCAAAATATTCCCTTATTGGGTCTATGCGTAGAATTGCCCAGTCCCTCTCATATGAAATTACCCTCGGTCTTCTTTTCTTCAGCTTTGCCTTCATCATGTCTTCAACTTTAATATTTAAAATTATAAATTTTAACTCATCAATACTTCAACTATTTTATTGTCCT------CTTTCTATGATTTTATTTCTGAACTACTTGATTGAAAGTAACCGAACTCCTTTTGATTTATCTGAGTGTGAATCAGAGCTAGTCTCTGGGTTCAACGTTGAATTTGGAGGGGCTGAATTCTCTTTAATTTTTTTAGGGGAAAATTTAATATTAGTTTTCAACTCTTTAGTATTATCCTTCTTTATTGGCAGTATCTCAGTATATCTAATTTTTTGACTTGTGATTATTTTCATCAAA------------GTCTCTATCCGAGGTGCATACCCACGCTACCGATTAGATAGAATGATGGAATTATGCTGATTGATCTACCTCCCCCTAACCATTGTTCTTCTAAGTGTTTTAACTTTAATCCTCATAGTGGTTTTCTTAATT------------------------TTTTTTGTTCATATAGTTAACAGAGTAGGTAAAGGTGAGAGATACACAGATCATCCATTTGAGTGTGGAATTAGAAGAAATTTTTCTTCACGTATTCTCTTTTCCCTCCCCTTTTTTTTAATCACTTTACTTTTTTTAATATTTGATGTTGAGATTATCCTGCTTTTTGTTTTAATTTTTTCT------GATCTGTCAGTGATATTCTTTCTTTTATATATCGTGATTTTATTC------CTTCTTATTGCGAGACTCCTGATAGAGTGATATTATGGATCTCTAGTATGGATGCTTCTTATAATAATCCTGAACATAGAAATAATCATGGTCTTGATATTTTTATTTATGTTTAATTATAAGATTAAA---------------ATTGTGATGATTATATTTATAGTAATGATAGTATGTGAAGCCATTATCGGGCTAATCTATTGTGCTTGCTGGTCATTAATCTTCAACAATCTTAAATCAATTTCATGAAGATGATTGTTTTTTTTTGATGCCTATTCAGCTTCCTTCTCTTCTGTGGTTCTTTTAATCTCAGCATCAATCGTGTTTTATTCCATATCTTATATACAACAAGAAAAGGAAAAAATCAAGTTTTTTTTAACCCTATATATGTTCATTCTATCAATGCTAATCTTAATTTTCTCTTTTAATATTTCTTCCCTTCTAGTCGGTTGAGATGGGTTGGGTGTAACTTCCTTTCTATTGATTTATTATTACCACTCATTAAAAAGAACAAACTCCTCTCTCATTACATTAACATTAAATCGGGTTGGAGATTTAATAATTATCTTTTCAATTACTATAGGACTCACAGTTTATACT---TGAAATTTTTTCTTTAGGGTAGAGACAATT---------------------AAAGTTTTTAATTTTCTTTTAATAACTGCAGCATTAAGAAAAAGAGCTCAGCTCCCCTTTTCTTCCTGGCTACCTCTAGCTATAGCTGCCCCCACCCCTGTTTCCTCTCTTGTTCACTCTTCAACTCTAGTCACTGCTGGTGTGTATCTCCTCTATCGA---GCTCCT---TTATCTATAATTAGATGCCTCAGAAATTAT---ATTTTTTTCATTACGAGCCTCACGCTAATTATGAGAAGGGTTTTAGCATTACAAAGATTCGACTTAAAGGAAATTGTCGCCTTTTCAACAATAAGGCATATCAGCTTAATGATAATAGGAATCTCAAATGGTCTTTATAAATTTTCATTTTTTCACCTTTGTACCCATGCTTTATTTAAGGCACTTTTAATCTCAAGATGCTCAATAATGGGGCTTCCATTTATGGCGGGGTTTTACTCTAAAGACGGG---CTAATTGAT---GAAAGCTCATTT------------TTATCAGTTTCTATGCTTTTTGTCATTCCTGTCCTTTTATCTTCCCTATACACCATACGCTTATTATACTATATTTGTTTATCTAAAACT---------------TCTGGGATAAGATTAAAA------------------ACTAATGACATGATAAGGTTTTCAATATTTTCTTTAGCATGATTTTCTATTTTTAGTGGAGCAGCAATTCAATGA------------------AGACTATTTCCTCTC------CTTTTTATTTCAATAATGCCT---CAGTTAAAAACCTTAATTTTAATTATATTAATTATAAGAATAGTGATATTATATATTACGGTT---ATATTTACGTTAGAGAGT---TCAATATTTAGGGCCTTAGTTTATATGTTTGTAATAATTTTTTTAGGCAGAGGGATTATAGCCACTGTTTCAGGAAGAGTTTGAATGAGAATGATTTTTTTAATTTTTATAATTGGGGGGTTGATGGTATCATTTTTCTATATAGTAAGGTTGACCCAC---AAT---ATAGTATTTAGAATTTCCCCCCTAGTATTA---------------------------TTTATCAGGGTATGTCTAGTCCCTTTTAGGCTTGCAAGTTTAGGTAAT---------------------------------------TTTCATAATTGTTTAGACTATTTTTTATTTTCT------------------------------------------------------------TCTCCAAGCTATCTGGTGGTTGTTTTGTTTATATTATTCTTGCTTTTCTTAATTCTGTTTATAATTGATTTTAAACTTAAGGCTATAAAAGGA

Pediculus_humanus ATATCTTCTTTTGACCCTTCTACTTCTATTATTCTAGGA---TTAAAAGTA------AAATGATTTATTATTCTT---------------------TTTCCTTTCTTTTTTATAACTGGAAGTTATTATTTAATTCACTCAGGCTACCGTTTTTACGTGAGTTTTGTGTTAAATAATTTATTAATAAAACACTACATATCT---------------------------------ATTATTAGGTTATCTGTTTTTATAATAATTTTAACCCTTAATACAATTTCACTTATGCCATTGGTGTTGCCTTGCACTTCGCATTTAAGAGTTAATTTAGGACTTTGTTTACCTTTATGGATAAGAGGGGTTGTTTACTCT---TTAAAAAGCTCTATGCGAGGGTTTCTAGCTCATCTTCTTCCTTACGGGAGTCCAACCATACTAAGTCCGTTCTTAGTGGTAATCGAGTTGTTAAGAGTCTCGATTCGTCCTGTATCTCTAAGAGTTCGACTTCTAGCGAATATTACAGGAGGACATTTAATTATAAATCTTTTAGAAGAAGGCTTATCTTCAGCTGTACTTCTTGTACTTCCTTTCTCAATAGCAGCGTATGTTCTTCTCTTAGCTGCTGAGCTATTTGTCTCATTTATTCAGTCTTACGTTTTAAGTAAACTGGTTTCAATTTACTGAGAACTTTGTCCGTCGATGTGAACGTTGTATTATATAATTGTAATATTTGTATTATATTTTATATTAACT---ATGATTTATTTTATAAAAATTGATAATTCAAATTATTTAGAGAATTTTAAATTA------AAAAATAAAACAATCTCCAATCTTTCTATATTTTCTACAAATCATAAAGATATTGGATTTTTATACTTATGCTCTGGAGTTTGGTTTGGACTTTTAGGCTTAAGGTTAAGGTTAATAATCCGGTTAGAACTTTCTAGAACAGGCTTGCTTTTGTCTGATAGACACCTATATAACGTATTTGTTACTTCTCACGCTTTTGTAATGATTTTTTTTATAGTTATGCCTGTAATAATAGGCGGTTTTGCAAATTGATTAGTTCCTTCAATATTAGGGTCTCCAGATATAGCATTTCCTCGTATAAATAATATGAGTTATTGACTTCTCACACCCTCTGGGATTTTGCTTATTAGTAGCTCATTTGTTCAAGGTGGTGTGGGTACTGGCTGGACTGTTTATCCCCCTCTTAGGTCTCTAGAAGGCCAACCTTCTGTTTCAGTTGATTTAGCTATTTTAAGTCTTCATTTAGCAGGAGTGAGTTCGATTTTAGGATCAGTAAATTTTATTAGAACTATTTTTAACATATGGCCTCAATATTTTGGCTTAGTTCGACTGCCTTTATTTTGCTGGAGAGTGTTGGTAACAGCCTTTTTATTATTACTGTCACTTCCAGTTTTAGCTGGAGCTATTACAATGCTCTTAATAGACCGTAATTTCAATTGCTCATTTTTTGATCCTTTAGGGGGTGGTGATCCTGTTTTATACCAACATTTATTTTGATTTTTTGGACATCCTGAAGTTTATATTCTTATTCTTCCTGGATTTGGTCTTATCTCTCATATGGTGGTAGATTGTTGTGGAAAGAAAGAAGTTTTTGGGTCATTAGGAATGATTTACGCAATATCCGCTATTGGGGCTTTAGGTTTTGTAGTTTGAGCACATCACATGTTTACAGTTGGATTAGATGTGGATAGACGGGCTTATTTTACTAGCGCTACTATAACAATTGCAATTCCAACGGGAGTGAAAGTCTTTAGGTGATTAGGCACTTTGTTTGGCCCAAAATTAAAAAGGAGAATTAGCTTGTTATGATCTTTAGGATTTATTTTCCTTTTTACAATTGGAGGTTTAACAGGCATTGTTCTTTCTAACTCATCCGTAGATGTTTCACTACATGACACTTATTATGTAGTTGCTCACTTTCATTACGTTTTATCTATGGGTGCTGTATTTGCTATTTTTGGCGCTTGAAACCACTGATTCTCACTAGGGACTGGACTTAAACTTCGTAAGTCTTTTATAAATGTTCACTTTTGGTTAAGATTTGTGGGAGTGAATTTGACTTTCTTTCCTCAGCACTTTCTCGGGTTAGCTGGGATGCCTCGACGTTACTCAGACTATCCTGACGTTTACCTGAGGTGAAACAAAATTTCTTCAATAGGAAGGCTAATTACTACTTTGGGTGTTGTAATCTTTCTTTTAGCTCTTATAGAAAGGTTTTCTAATCCTCAAAAAATTGTATTTAGAGATGCTAGATTGCAAGACCTTCCGCGTCTAATGGGTATACCAGCAAGAATGCACTCTCATTTTACTCTTACATTTACTAGAGTATGTGGATTCCAAGATAGAAATTCTCCTTTAATAGTTTTTGTGTGTGATACTTATGACCTTGTGTCTATTGTTTGTGTGGGGGTGATCTCCTTAGTAATGTACGTGGCTGTTAGTTTCTTTTTTATAAAATCTTGA---------AATTACTATTTTATAGGTCTTGAAAGATTGGAGATTGTTTGAGTTATCTTACCCTCACTCTCTTTAGCAGGGTTAATTTTACCGTCACTTCATTGTTTATACTTAATAGACGAGGTTCTTTCTCCCGCTATGAGATTAAAAGTGGTCGGACATCAGTGATTTTGGTCTTATGAGTACGGGGATTGAGAAAATATTGAA---------TTTGATTCATATATGATAAAATTAGAGGAGCTTGACTCGTCATGTCCTTTTCGACTTTTGGAGGCTGATTTAAGCGTGTTTATCCCTTATTTGACTGAAGTGCGTGCTATTGTAACATCTGCCGATGTTATTCATTCTTGAGCAATTCCTATAATAGGAGTAAAAGTAGACGCTATTCCTGGGCGTTTAAACCATGCGCTTATTTACTCATTTAAAATTGGCACATCTTATGGTCAGTGTTCTGAGATTTGTGGTGCTTATCACAGGTTTATGCCGATTAAAGTCACTACTCTTCCAAAAGAAGACTTTATAAAATGAGTTAAAGATTTAAAATTTCACCCATTTCATCTTGTTGATGTAAGACCTTGACCTATTTTTTTAAGATTTTCTCTTTTATTTTCAGCGTCCATAACATTGTGTTGAATTAACGGGCTTTATTCTTTTTATATTTTAATAATTAGAATTGTTGTTTCATCTTTAATTGTTTCTTTTTGGTGACGAGACGTTACTCGAGAGGCTACTTTTCAGGGTAAACATACAATAGAAGTAATTGCTGGATTGCGTTTAGGAATGCTTATGTTTATTGCTTCAGAGGTAATGTTTTTCTTTTCGTTTTTTTATGCTCTGTTTTTTCTTTCTTTAAGGCCTGACGTGTCATTGGGACTTCTTTACCCTCCTGTGGGTGTTAGCCCTGTAGGCGTTTTAGGAGTTCCTCTTTTAAATTCTATTTTATTACTCTCTAGCGGTGTGTCTATTACTTGAGCTCATTATGAGCTTTTA------AGGAAAAATATTTCTTCTAGGCTTATCGGCTTATTAATCACTTTAATTTTAGGTCTAGTGTTTCTAACATTTCAGGCTGTTGAATATAAAACAAGCTCTTTTACTATGGCTGATAGAAGTTTTGGCTCAGTGTTTTTTCTAATAACCGGCTTCCACGGAGCACATGTTTGTGTGGGAGTTGTGTTTATTACAATTAGAACCATTCGGCTTTACTTAAATCACTATAATAATAATCATCACTTAGGGCTTGAGCTAGCTGCATGATACTGACACTTTGTCGACGTAGTGTGGTTATTTTTATACCTAACTCTTTACTGATGAATACTCTTGAAATTTTGGGTCTTATTAGGCTTGTTTCTTTCAATTCAGATTTTAAGAGGTCTTTTTCTGGCTTCTCATTATGAGGCTTCTACTAAT---TCGTTTTGAAGTGTTATTTTAATTGATTTTGATGTAAATAGAGGGTGGTTGATTCGTAGTTTTCATGCTAACGGCGCTTCTTTTTTCTTCATTCTTGTCTACGTTCATATTTGGCGTGGTTTATGATTTGGTTGTTTTACACAAAAA---TATGTTTGATTTTCAGGAATTTCTATTCTTCTTCTTATAATAGCAGCAGCTTTTATGGGGTATGTTCTTCCTTGAGGTCAAATATCTTTTTGAGGAGCGACTGTAATTACTAATCTTTTAAGTGCTATTCCTATTGTTGGAAGAGATTTGGTTATTTGAGTGTGAGGAGGGTTTTCAGTTAGACATCCTACTTTAGAGCGGCTGTTTACTCTTCACTTTCTTTTACCGTTTGTCTTATTGGGGTTTGTTATAGCTCACATTATTCTCCTCCACCAACACGGTTCTAGAAATCCTTTAGGATTGGATTTGGATAGTGATAAAGTTTATTTTTATCCTTACTTTTATCTAAAAGATATTTTAGGAGGTTTTGTGTGTTTATTTTTATTTGTTTTGATTTGCATTTATTCGCCGGACTTCTTCATGGACCCGGATAATTTTGTTGAATCAAACCCGATAATTACACCTCCACACATCCAGCCAGAGTGGTACTTTCTATTTGCATATGCAATTTTACGGAGTGTACCTAACAAGTTAGGAGGAGTTGTAGCTTTGCTTCTAAGAATTTTATCTCTGTCTTTTATTAGCATA---------GGAAGCTCTGTCTCAAGTCGCTTTAGAATAAGGCGAATGATTTTAACTTATTCTTTTACGAGAGTTTTTGTTATGCTCTCATGACTTGGCTCTCTTCCTGCTGAGTATCCTTTTACTCTGCTAAGTCAAGTTGTAAGAGTAATTTATTTCATTCAAGTAATTCTATTTATGCAGTCAGTATTTATTGTTTTATCACTTTTAATTTGTGTTGCTTATTTCTCCTTGTTTGAACGTAAACTTCTTAGATTAGAGCAAATCCGGCTTGGACCAAATAAAGTAGGACCAATTGGTATTCTTCAACCTTTAAGAGATGCTCCCAAATTATTATCCAAGACTATTTGTCCGCAG---AGAGAG------TCATGAGAGTTATTCATTATGCCGTTTATTACATTTATGTTAAGCGTTTCCTGATGGTACCCGCTCTATTTTCCAAAAACTTTATGA------GAATCAAATAACTCACTTTTAATCCTTATTTTTATCTCAAGAGTAAGAGTTTATGCATTAATTTTTACAGGGTCACTGCCAAAATCAAAATATTCGGCTCTAGGAAGACTGCGTGCAATTACTCTTTCTATTTCTTTTGAATTAGTTTTTTCCACTGCTATGCTAAGTATGGCTGTAGTTTTTAATTCTTTTAGCATTAAATTTATAGCAACCAATCAGAGC---GTGCCTAATATTATCTCAATAATT---GTAGTTGGAATTTTAGTATGAACCTCACTAATTGCTGAATGCGGACGAACTCCTTTTGATTTACCAGAAAGAGAGTCTGAATTAGTAAGAGGTTTTAACGTAGAGTACGGAGGTAGGCGTTATGTTTTACTTTATTTAAGAGAAAGATTGTTACTTACCATTTCATCTATTATTATAAGCATTTTATTTACCTGC---GGGTATAATCCATTAGTGGTTTTAACATGAATTAGAATTTCT------------ATTGTTATACGAGCTAGAAACCCTCGCATTCGGTACGATAAATGCATAATATTCGGGTGAGAATTTTCAATTCCTTTAATCTTAATATTTATGAGATTTACTATTTTGATTATTTTGGCTATTATTTTAGCT------------------------TTAGTTACTTTGGTTTTTGTATCTCAAAGTCCTAAATTAACTCTAGATAGGACGCCCTATGAGTGCGGTGTAATGCCTTTTAGAATAAGAACTCTGTCTACACACATTCATTTTTATGTTGTAAGTGTGGTATTTCTTATTTTTGATGTAGAATTAGTAGCTACTTTGCCTGTTGTTACTTCA------AGGCTATTAGAGAAAGATTGATTGTCAATT---TGACTTTTAATTCCACTTATTCTCACCTTAGGGTTGCTTTTAGAACTTCATTATGGAAGTTTAGATTGAAAAATTATATCTTCTCTTATTTCTCTTGAATTAAGTTGATGCTGGGTTTACGTAATTATACACATTGCACTTTCTGAA---AGGTTAGACGCTCTTTTAAGATCAGAGGTTTTAAGTGTTATTGTTTGCGAGAGAGTTGTAGGACTTTCTTTACTTATTAGGCTAACTTATGGGTGAGGAAGGACTGGTTTCAGGGGGGTGGAAATTATTATTGTACTGGATAATTTAAGCCTGACTTTTTTGCTTATAGTATTAACAATTAGGTCGCTTGTTATAGCTTATAGAAACAATTATATAGCAGGTCATAATCTTGGCGGTGATTTTTACGTTTCCATGGTCTTATTTATTGTAAGAATATTACTTCTATCTCTTAGAGGCTCAATGTTCTGATCTTTTATTGGGTGGGACGGTTTAGGAATAATAAGTTTAGTTTTAATTTTATTTAATAAAAGATGGAGTTCTCAAAAATCGGGAGTGATTACTTTCCTAATGAACCGATTAGGTGATTCTTTTATAATTATTTGCTCGTCATATTTAAGAGTGTGAGGGATA---TGCGAA---------------------------------GTTTATTGGTTTTGAATTTTAACTTCTTTGTATTTAATTGGAGGGGCTTCAAAAAGAGCTCAATTTCCATTTTCTAGCTGATTGCCGGAAGCTATGGCAGCCCCTACTCCCGTGAGTAGGTTAGTTCATTCTTCAACATTAGTAACTGCTGGTATTTATGTTTTAGCTCGTTATGGAAGA---ATAATCGATAGTTTTTACATT------------CTTACCTACTTATCTTCTATTTCTATTATTATTTCGGGTGTTTCAGCTCTCTGAAGGAGAGATTTAAAGAAAGTTGTAGCTTATTCAACTCTTTCTCACATTAGCTTAATGCTTTTTTACTTATCTGAAGGAAGGGTGGAAGGAGCTTTAATCCACATGTTGACGCACTCTGTGTTTAAAAGGCTGTTATTTTCTTGCCTTTCAATGGCCGGACTGCCGTTTCTTTCGGGAGGTTATTCAAAAGAGGTG---TTA---------CTTATTTTAAGTTTAAATAGATCAATTATGAAGCTTATTATATTTCTTACAGCTGTAATTTTCACTAGAGGTTATTCGTTTCGCATTATTTACTTACTTTCTAGAAAT---------------------ATTAATATAACACAAAAT------------ATTGCAGTAAGGAGGTTGTTTAGCAGCCCCCTTAAACTGAGGCAAATGCTTAACGTTCTA---------ATTTCAGCTTGAATTTCCTCAAGTCCTGGCTACTTATCTAAACTC------AGAAGGCAG---AGAGTTAATTTAGAGGGTAAAATTATAATACTCTTTATAATTCTATTTGGTGTGGTTGCTATGTTTTTTACGTTT---CTTATTTTAGTAGGCTCT---GATCTTTTGCTGAAGCTTTTTTCTCTAGCTATAACTGTTTTTACAGTCGGACTGCTTATTCTTCTTAACTCTTACTCTTCATGGTCTTGGTTGCTTTTATGGTTAGGGATTTTGGGTGGTTTAATTGTCTCACTTTCTATGGCTTTTATTGTAACGCCA---AAA---ATTAATTCCTCAAAGGACTGGTCTAGAAGATTAAGA---------------------TTTATAAGAGCATTTTGAATTTTAACTTCTGTTATACTATCTGCTATA------------------------ATATGAAAAGTAGAATTTAAAGAATGATCTGACTTGTACTCTACAGAG---AGTGTAAGAGAA------------------ACTTATAATTCACTA---CTTATAGATCTTAAGGTTTATACAGCTGTGGTTATTCTTTTAATT------TACATTTTAATGCTTCCTGTCATAGAAGTTTTAACGTCACCTTATAGACGT

Haematopinus_suis ATATCAGTGTTTGACCCCTGTTCTACTTTGTTGAGGTTAAATTTACCTTTG------AAATGGTTACTTGTGGTT---------------------TTAGTCGTATTAAGACTAAGAGGGCGTTATTGAATTCTATCTTCAGGGTTGCAGTGTGTAATAGTGTGGGTTAAAAATGGACTAATTCATGGATTACGTGAGTCATATAAAAACTACAAA---------CAGTTTATACTCATTCTTCACACACTGTTTTTTTTTATTTTTTCTAGTAATTTTATAGGACTATCACCTTTCATGTTTACTCTCTCGTCCCACTTGGTTTATAATTTAAGATTATGTTTCCCATTGTGGTTAGGAGGAATTTTATATTCG---TGATCAAAATGTTGAAAGAAAACATTGGCACATTTAACCCCTGTAGGTAGTCCAGTAGCTTTAGCTCCCTTTTTAGTGTTGGTAGAAACTGTAAGGTTGATTATTCGACCAATTAGTTTAAGTGTTCGATTGATGGCTAACATGACAGCAGGTCACATAGTAATTACTCTTGCTGAACAAGGGGCCATGTCTGTAGCTTCTTATGTTGGGAGGTTTTATGTGTTGTTAGTAATAGTATTATTATTGTTTGAGTTAGGGGTGGCTCTAATTCAGGCTTATGTGTTCATAAGACTAATGTCCTTGTATTGGGAAATGTCTCCTATGTGATGGTTACTGCTTGAAGTTTGATTTTTTGTGAGTTTTATAAGGTGTTGTAGA---TGTTTATATTGGGAATTGTTTGTAGAAAGATTAGCGTCTCCAAAAGTGTTTTTG------AAAGTCTTAAAAGAAGGTGATTACGTGTTACTTTCAACTAACCATAAGGATATTGGTGTTCTGTATTTGATTTTAGGTGTTTGAGCAGGTTTGTTAGGGACAAGAATAAGGTTGTTGATTCGCGTTGAGTTGGGTAGAGTGAATAGATTAATTTCTAGTGGACATGTTTATAATGTATTTGTTACAGCTCATGCGTTTTTGATAATTTTCTTCATAATTATGCCAGTAATAATTGGAGGTTTCGGAAATTGGTTAGTACCAACTATGTTGGGGGCTCCTGATATGGCATTTCCTCGAATAAATAATATAAGATTTTGGCTATTACCACCATCACTTTTTTTATTAATCTCAAGATTGATTGTTGGAGGAGGTGTTGGTACTGGCTGAACGGTATACCCCCCACTAAGGGGGTTGGTCGGACAGCCCAGAAGGTCTGTAGATTTAACTATTTTTAGACTACATTTAGCGGGTTTAAGTTCCATTATGGGTGCTATTAATTTTATTTGTACTATTGTAAACATGTGGGTTTGTGGAAAAAAGTTAGAACTATTACCTCTGTTTTGTTGGTCAGTTTTAATTACTGCTGTCCTACTGTTACTTTCTTTGCCGGTTTTAGCGGGTGGGATTACTATATTGTTGATAGATCGTAATATAAATTGTTCGTTTTTTGACCCTTTGGGTGGGGGTGATCCTGTGTTGTACCAACACCTATTCTGGTTTTTTGGCCATCCTGAGGTTTATATTCTCATTTTACCTGGTTTTGGATTGATTTCACATATAATCATGGAGGAGAGAGGGAAAAAAGAGGTTTTTGGAACGTTGGGAATAATTTATGCTATGGTCGCTATTGGATTATTGGGGTTCATTGTATGAGCACACCGTATGTTTACAGTAGGGATGGATGTAGACAGTCGGGCATATTTTACCAGAGCAACTATGGTTATTGCCATTCCCACAGGAGTAAAAGTGTTTAGCTGGTTGGCAACTCTCTTTGGAGGTAAGCTTGTTATGTCAGTAACACTATTGTGATGTTTAGGGTTTATCTTTTTATTTACTGTTGGTGGTTTAACAGGTTTAGTTTTAGCTAATTCTTCAGTTGATGTAGTATTACACGATACCTACTATGTAGTAGCACACTTTCATTACGTGTTATCTATGGGGGCTGTATTTGCAATAATTGGAGCATTTAATCATTGATTTCCCATCATTAGCGGTGTAAGGTTAAATCAGAAGTTGATGAAAGTTCACTTTTGGATAACGTTTATTGGTGTAAACATGACGTTTTTCCCACAACACTTCTTAGGTTTGAGAGGCATGCCACGTCGATATGTGGATTATCCTGATGTTTTTCTATGTTGAAACATATTATCGTCAATTGGTAGACTAGTTAGAGCTGTGGGAGTGTTGTTATTAGTATATGCAATTTATGAAAGGCTAGTGAGAAAACGGGTTGTTGTGTACAGTCTATCT---GGGAATTCTTTAGATGCTATATTTGGCTGTCCACCTAATGCTCACACACATGAGAGGGTGCCTTTGGTCTTTAACATAGGATTCCAGGATAGTTCCTCTCCAATGATGGGTTATATTACTGGAGTGCATGATTGGATTATAATTGTGGTATTAGTTGTTGTTTCAATTGTTATATATGTGTTAGGAGGAATAATACTAACAAAGGGGTGG---------GACCGTTTTCTTGTAAGTGCAGAGACTTTGGAGTTTATTTGAGCGGGACTACCAGCTATTTCTTTGGGGTTGTTGGCTATCCCTTCCCTTCATTGTCTCTACCTAATAGAGGAAGCTTATTCTCCTTTTTTAAGGTTTAAAGTTGTAGGCCATCAATGATACTGGTCATATGAATATTCTGACTATTCAAATTTGGAG---------TTTGATTCATACATGCTAAGTCAAGATTCCTTG---------------TTTCGCCTCTTGGAAGTAGATAATGCAGTTGCTATTCCAATGGATTGTGAAGTACGCGTTTTAGTTACATCTGGAGATGTAATTCACTCTTGGACAGTACCTTCAATGGGTGTAAAGAGGGACGCTATTCCAGGTCGGTTAAACCAGCTAGTACTAATTGGATCAAAATTGGGATCTTATTACGGCCAGTGTTCAGAAATGTGTGGGGCTAATCATAGTTTTATGCCGATTAAGGTTGATGTTCTTACGAAAGATTTATTTATGAATTGATTATTAAAGGGAGGATTCCATCCATTTCATCTAGTGAGACCTAGACCATGACCTCTATTGTTGAGAGTTTCCACTTTTTCCCTAATAGTTGGATTTTATGTATGAATGTCG---AGTATAGGGAGGGTGTTGATAGTATTAGGTGTGTTTAGAGTTATGTTGAGGTTGTTTTGTTGGTTGCGAGATGTGATTCGTGAAAGGACCTACCAAGGGTGTCATACAATACGGGTCATGAAAGGTTTACGTTTGGGTATTGTTATATTTATTATCTCAGAGGTAATGTTCTTCTTTTCCATTTTCTTTGGAGTGTTTTTTTTATCTTTAAACCCTGACGTAGTTTTAGGAAGATCCTACCCACCCGTAGGTATTCAACCTTTAAATTATATAGGAGTACCATTTTTGAATACTATGATTTTACTGTCGAGAGGTGTAACTGTAACTTGATGTCATCATGGAATTATG------AGAGGTAATAAACACCATAGAGTGTTGGGTTTGACTATTACAGTGGTGTTGGGAGTGTTGTTTGTTATGTTTCAGTTTGAGGAATACTACGAAAGTTCATATACTATTGCCGATAGTGTATGTGGTTCATTGTTTTACATGTCGACTGGTTTTCATGGAATTCATGTTATGTTAGGTACTGTAATATTAATTGTAAGTTTAGTCCGATTAACAATAAACCATTTTAGAAGAATTCATAATTTAGGTTTTGAGATATCGGCGTGATATTGACATTTTGTGAGTGTTGTT---------TGTTATGTTTCAGTT------TGATACTTGTGAAACTACGGTTCCTTGTTATTGATTTGTTTAGTAATACAAATTGTTAGTGGTATTTTTCTATCTATGCACTATGAGGCATCAATACTAAATGCATTCTCCAGAGTAGTATCAATGGTAAATGATGTAAACTGAGGTTGACTTATTCGAATGGTTCACGCTAATGGGGCATCATTTTTCTTTATTGCTATTTATATTCATATCGGACGAGGTTTATACTATGGGAGATACCGAATAGTA---GGAGTCTGGTTAGTAGGGGTGGTATTGTTGTTTTTACTTATGGCAACAGCATTCTTAGGTTATGTTTTACCTTGAGGTCAAATGTCATATTGAGGAGCAACTGTAATTACAAATTTGCTATCTGCAATTCCTTATTTTGGTGAGGCTATAACAGGATGGTTGTGGGGAGGTTTTTCTGTTGGTAATCCAACTTTGGTACGGTTCTTTTCATTTCATTTTGTGTTGCCTTTTGTTATTTGTTTGTTTGTGTTATTTCACTTGGTTTTCCTTCACTGGTTTGGAAGTTCAAACCCTTTAGGGTTATCCAACAAATCAGATATGATTTATTTTCACCCATATTTTAGTGTAAAAGATGTTCTGGGATTAGTTGTGGCTTTGTTTATTGTTTGTGTTGTAGTTCTTCTTTTTCCTGATTTGTTTATAGACCCTGATAATTTTATTGAAGCAAATCCGATAAACACACCCCCTCATATCCAACCTGAATGATATTTTCTGTTTGCATATTCTATCCTTCGTTCGATCCCTAACAAATTAGGTGGAGTTGTGTCTTTGTTGGCAAGGGTGATAATTTTAGCATTACTTCCACTTTAT------GGAAAAGGGTTTAGATTTCGCTTTATAGGACTAAAGAAGATTTTATATTGATTTCACGTGATGGTATTTTTTATATTGACAGTGTTAGGTTCAATGCCTGTAGAATATCCATATACTGCTATTAGCCAAGTGATGACTTTAATTTATTTTGTGAATTTTATATTCCTAGTACAAATGTTATTTACTATTATATCAATTTTTATTGCTGTAGCTTTTTTCTCTCTTTTAGAACGGAAGATATTGAGGATCTCTCAAAACCGAGAAGGTCCAAATAAGGTTGTTCTGAAGGGGTTTTCTCAACCAATTGGTGATGCAGTTAAACTTTTATCGAAATCTACTAGTTTGCCA---AACTTAGGCTTCTATTCGGTATACACTTTAGGTCCTCTAGCTCTGCTTAGAATTAACACCTTCTTATGGATTACAACTCCA------TTTCTTTCCAAGTTTATCCACTTAAACCATTCCGGCATAGTTATACTTCTAATCCTAAGTGTTACAGCCCTACCTACCATTTATAGAGGTTGATTCTCTAATTCAACATTTAGAACTATGGGGGCAATTCGCTCTGTTGCTCAATCTCTCTCATTCGAGATTACATTTAGATTCAGACTATTTATTAGATTCCTTATAATTCAATCATTATGTTTGGAAAACCTTCCTAAGTTTCAAAGATGAAGCTGA---CTGTTTTGATGTATTCCGTGAATTTCTCTTGTAACGTTGATTTGTTTTTTAGCAGAAAGAGGACGTAGACCTTTTGACCTTCCAGAAGGTGAGAGGGAGTTGGTAAGTGGTTACACTATTGAATTTGGTGGATTGCATTATACATTAATTTTTCTTGGAGAGAATCTTGCTGTAATATTTATAACCATAATTTTCTCCACCACCTATCTTGGT---GGGTTTTCTTTGTGAAAAGCAAGTACACTAGTATTAATTATT------------GTTATAATTCGGAGGTCTTACCCCCGTATTCGATATGACCAACTTATACAGTTAAATTGAGTCGGGATCTTACCTCAACTTATCTCTTCAGTTTGATTGGTGCTTGTGTTATCTATTATGTTATTCTTATTA------------------------GTACTGTCTGTGTTAATTTCTTCTATATCAGAGACAAGTTTTGATACAAATGAGTCTTTTGAGTGTGGATTTTTTACAGGAAGGGATATTCACTTACCGTTTTGTGTTCATTTCTTTATTGTAGGTATCCTTTTTGTAGTATTTGACATGGAATTGGTAATTTCACTACCACTAATTGTGGCA------AATCTGAGAGAACCAGTATGGTTATTATGA---TGGTTAGTATACAGCATTATTTTGTTTATTGGAATTCTATTAGAAGTAACATGTGGTTCAATTGATTGGGGTATAATAATTGCTCTATTAAGAGTTGAGTTTCTATCTGTCAGCCAGTTTTATGCTGTCCTTTTTCTTGTTAATCCAAGATCGTTAAATTTTAATAGATGTTTGGTGTTACTGTCAATTCTAGTGTTGGAGGGAAGCTTAGGTTTGACCATTCTTGTTTCGACAAGTCTAAAAATTGATTCTACAATAATTTGAGATGTTGGTTGTGTAATGGTTGTAGATGGTTTAAGGTCGTTATTTTTGTTTACAGTTTTTCTTGTAAGATTTATAGTTTTCAATTATAGAGTGCATTATTTTTCTTATTTAGAAGGGTTTAATAAGTTTATTATTACGCTGTTTTTGTTTGTCATATCAATATGTATTCTTTGTTTAAGACCAAGAATGTTTTGAGTGATAGTTGGTTGGGATGGTTTAGGATTGACTTCATTTGGCTTAATTATTTTCTATCAAAATTGAAGGAGGTTTAGGAGGGGTTTGTTTACATTTTTAATAAATCGTATAGGCGACATGTTTATGATTTCGGTCATTGTGATACTGTCAAGA------------TGCAACATGTTGAAAAGAGTGTCAATAAGG---------------TCTGCTGTTCAAATTTCTGCACTCTTACTGTTAGGTGCAATAACAAAAAGAGCTCAATTGCCCTTTTCTTCATGACTTCCACTAGCAATGGCTGCCCCTACTCCGGTGAGGAGGTTAGTTCACTCGTCAACTTTAGTAACTGCTGGAATCTATTTGCTAATTCGCTTTGAAAGT---TTATTCCCATCGGAGGTGTTACAAGCA------CTTAAGGTAGTGTCAATCATGACGATTGTTTATGCGGGAGTATCAGCACTTTGTGAAGTGGATTTAAAAAAGGTAGTAGCTCTTTCTACACTAACCCATTTAGGAATTATAACACTATATGTATCTATCGGGAGTGTGACTGCTGCTACAACTCATTTAGTTTTCCATGCATTCTTTAAGAGAGCATTATTCATACTATCTTCAATAGCAGGTCTGCCTTTTTTGACTGGATTTTACTCGAAAGAAGTA---------------ATGGTGATGTTAGCAGAAGATTCTTTGCTTACACTGGTTTCATTTTTGTTAGGGGTAATGTTAACTTCTGGTTATTCAGTTCGATTAATAGTACTAATTTTTAAATCA---------------------CCCAATTTTATTTCTGATAAAAAGGATTTTACCCAAGTAAGAGAAGGTTTGGTTAGAGCAAGGGTGCACGGGTTTATGATTAGAGTACTTGGCGGTAGAATGTTACTGTGG------GTTGTAATACCTATTACGATGGTTATG------GGTTACAATTTAAGATGTAGGGTTTTAAATAAGCTATTGATTATTGTGTCGCTTGCAGGAGGACTTATTGCAGCATTATGTTCCTTA---TACTTGTGGATAGCTTCA---AGGTGTAGAATGGTAGTTCTGTCTTTATCTGTGCAAATTGTTATATACAGGGTAATGTTGGCAATGAGGGTTACTCACTATTGAATTGTAATTTTAAGGATTATAGGCTTGTTGGGAGGTTTGATTGTCTTTCTGTCATTTATCCTTATAATACTTCCT---AATCCAGATTTAGGTGTTTTTAATAAATGAAAT------------------------------TATGTAGGATTATCAATACTTATATTATTAATACTGACTAGTATTTAC------------------------------------------------------------CTACCAGTAGTAGATAAGAATGAGCCT------------------GAACTGTTGCCAATT---TCACTACATTTATATGCTTACGGCCTATGTTTCTTTTTATTGCTTTTTGTGTTACTAATTATAGTAGGTATTATAAATTATGGTTATAAATCTATCTATCTA

Pthirus_pubis ATGTCTATATTTGATCCTAGAATGATCTTAGTAACTGGG---TTACCTGTA------AAGTGGCTGGGTGCAACC---------------------TTAGTCTGTCTGTATGCGGGAGGCGAGTTTTGGCTTGTTAACTCTGGGTACCGGAGGGCTGTTACTAAAGTGTGCTTATTCCTTCATGAATTGCAT---------------------------------TTTATTCCGTTTTTGGGTGTTGTAAGATTTTATTGTGTATTGAGCATAAATGTTATCTCGTTAATTCCTTTTACTTTACCTTTTACCTCTCATATTTCTGTTAATTTGGGGATATGTTTGACTCTGTGGTTAAGTGGCTTACTTTACTCT---TTTGGAAGTTCGCTCAGGAAGTCTTTGGCTCATTATTTACCTTTGGGGAGTCCAATGGTTCTGGGTCCGTTCCTAGTTCTAATTGAGGTAGCCAGGGTTCTTATTCGGCCAATTAGGCTAAGAGTGCGGCTGATGGCTAACATTTTAGGTGGCCATATTATTATGAGTCTAATCGAGGAAAGGTCCTTTGGT---AGCTTGATTAGAGGGATTTTCACTTTACTAACATATTCTCTAATAGTTTGTGTAGAGATGTTTGTAGCTACTGTACAGGCTTATGTACTCAGAAAGCTCTTAAGAATCTACTGAGAGCTATCCCCCTTACTTTGGTTAAATATTTTTATTTTTATGGTGTTGGTGTTTATGGTTATGTTGTCT---GGATCTTATTTTTCTATGTCCACTGTTGAAGTTAAGTTTCGGTATGAAGCTCGG------GTTATTTTAACGTTGGAAAAGTGTATACTATTTTCTACTAATCACAAGGATATTGGACTTTTGTATCTGCTTTCTGGAATTTGATTTGGTTTAGTTGGCTTGTCAATGAGTCTAATTGTGCGTGTAGAACTTTCATCAACCAGGTCATGATTGGTTAATAGACATACTTATAACGTGTTTGTTACATCTCACGCTTTTGTAATGATTTTTTTTATGGTTATGCCTGTTATGATAGGAGGTTTTGCTAACTGGCTAGTTCCTTTATTTTTAGGTGCTCCTGACATGGCTTTCCCCCGGATGAACAATATAAGCTATTGGCTGATTATACCCTCTGGGGTTCTGTTAATTGCGAGTTCAATAATCCAAGGTGGAACAGGTACTGGCTGGACTATTTATCCTCCGCTAAGTCCTTTAGAAGGCCAACCTTCTTTATCAGTGGATTTTACCATCTTTAGCCTCCATTTAGCTGGAGTAAGTTCTATTTTAGGCTCAGTAAACTTTATTAGGACAATTTTAAATATATGACCTTCTAGGTTAAAGATTTACCGGTTACCTTTGTTCTGCTGGTCTGTCTTAATCACGGCCTTTTTGCTTCTACTCTCTTTACCTGTACTTGCGGGTGCTATCACTATGCTACTATTAGATCGGAATTTTAATTGTTCCTTTTTTGACCCTCTAGGTGGAGGTGATCCTGTTCTTTACCAGCATCTTTTTTGGTTTTTTGGTCATCCTGAGGTTTATATCTTGATCTTACCTGGGTTTGGATTGATCTCTCATATAGTTGTTGACTTGAGAGGTAAGAAAGAAGTCTTTGGTTCATTAGGAATAATCTACGCTATAGTATCGATTGGTGTTCTAGGGTTTGTTGTTTGAGCCCACCACATGTTCACTGTTGGTCTAGATGTAGATAGACGTGCTTACTTCACTAGAGCTACTATGACCATTGCTATTCCAACGGGAGTAAAAGTCTTTAGCTGATTAGGTACTCTATTTGGCCCTAAACTTCACTTAAGAGTGAGGTTGCAGTGGTCTCTGGGATTCATCTTTTTATTTACTGTCGGGGGCCTGACAGGGATTATTCTATCTAACTCGTCAGTTGACGTTCTTCTTCACGATACCTACTATGTTGTAGCTCATTTCCACTATGTATTGTCTATGGGTGCAGTGTTTGCTATCTTTGGTGCGTGGAACCATTGGTTTTCTTCTTTAAGAGGCTGCTACCTTAACCCTAAAGTTATATCCACTCATTTTTGAGTAAGGTTTATCGGGGTAAATTTAACCTTTTTCCCTCAGCATTTCCTCGGTCTCAGGGGGATACCTCGACGTTACTCAGATTACCCGGATGCTTTCTACGGGTGGAATAAAATCTCCTCACTAGGAAGTATACTAACTTTTGTAGGAGTGCTACTATTTCTCTATGCTTCGTTTGACAGAGTTGCTAGAGCAAATAAAGTACTGTTTGAGAAAGTTAGAGTACAGGATCTTGTGGGTCTTGTTGGTAAGCCGTCAGGGTTCCATACATTTACCACTAGATTACATCTGTCAGATTTTACTTTTCAGGATGGTAGATCTCCAGTTATAGCGTTTGTTTCTAATACTTACGACCTTGTTTGTGTTGTTTGTGTCGGTGTTATCGCTTTAGTGGCTTATGTCTCTCTATTCTTTCTTTTGAACAAGTCGTGA---------AATTATTACTTTGTAGGCCTTGAGAGGTTAGAAACTATCTGGGTAATTTTACCGTCTTTAGCGCTAGCCTCTTTGGTCTTACCTTCTTTGCACTGTTTATATTTGATGGATGAAATCTACACTCCTTCAGTTACCCTTAAGGTTATCGGCCACCAGTGGTATTGGTCCTATGAGTACGGAGACTGAGATAGAATTGAG---------TTTAATTCTTACATGTTAAGTGAAGGTGACTTAAATAACGTCGAGTCATTTCGTCTTTTAGAGTCTGATTGCAGGGTGTATATTCCTTCTAGAACAGAAATTCGTGCTATCATTACTTCTTCGGATGTCATTCATTCTTGGGCTATTCCTAGACTAAGGGTTAAGATAGATGCTGTCCCTGGACGGCTAAACCATTCTGTCATTTACTCTCATAAACTTGGGAGTCTGTATGGTCAATGCTCTGAGATGTGTGGGGCTTACCACAGGTTCATACCAATTTCTGTCAAGACTGTTCCTAAGGCGGTATTTATCGCATGAGTGAAGGAAAGTAAGTTTCACCCATTTCACCTGGTTGATTTTAGCCCTTGGCCTTTATATTTGAGTCTATCCTCTTTATGTTTTGCAGTGGCTGTTCTGTGTATTTTAGGTTCATTAAAAGGGTTT------TTAGCTTTTTCTGTAATTGTGTTTGTGCTTGTGCTTTCTTTGTGGTGACGTGATGTAATTCGTGAATCAACTTTTCAAGGTAAACATACTATCGAGGTTAAGCGGGGTATCCGTTTAGGAATAATTTTGTTTATTATGTCTGAGGTAATGTTCTTCTTCTCGTTTTTCTATGGTCTTTTCTTTCTAGCTCTTAACCCAGATGTAACTTTAGGTGGTCGGTTTCCTCCGGTGGGTCTAGCGTCAATAGGGCTACTTGGTGTGCCTCTGTTAAACTCGTTTTTACTCTTATCAAGTGGGGTCAGGGTAACTTGATCTCACTACGAGATTCTC------CGGGGTGATGTACCAAAGTCTTTACTGGCGTTGGCTATGACTCTAGCTCTAGGTACTGTGTTTTTAGCCTTTCAGTATCTGGAGTACAAACTTAGGAGGTTTAGGATCTCTGATAGTGCTTATGGGTCTATCTTTTACCTTATAACTGGCTTTCATGGGTTTCACGTTATGGTGGGAGTGGTATTCATTTTAGTAATAACAGTTCGTTTAGCTAAGGGTCATTTGAACTCAAGACATCATACAGGGTTTGAGCTATCAGCCTGATACTGACATTTCGTGGATGTAGTATGGTTACTCTTATTTGTGTCACTTTACTGATGGTATTCTTGAAATTTTGGTTCTCTTTTGGGTTGTTTTTTAGTTGTTCAAGTGTTTACTGGATTTTTGTTGTCTACTCATTATGAGGCTTCTTTGTCT---TCATTTGAAAGGGTACTCTTGATCGAGTTTGATGCTTTAGAAGGGTGATTAGTTCGTAGCTTACATGCTAACGGAGCTTCTTGATTTTTTATTCTGGCTTATTTCCATATTTGACGCAGTCTCTGGTTTGGTTGTTTCTCTCAAAAG---CTCGTTTGAGTGTCAGGTATCCTAATTTTATTGCTTATAATGGCTATCTCTTTTTTGGGTTATGTCCTTCCTTGGGGGCAGATGTCGTTTTGAGGGGCTACTGTTATCACAAATCTTTTCAGGGCTCTACCTTTTGTAGGCTCAGAGTTGGTTACCTGGATTTGAGGTGGATTCTCAGTGGGGTCGCCCACGCTAGAGCGATTTTTTAGTTCCCATTTTATGCTAAGAATGGTTTTGCTGTGCTTTGTTATTTTCCACATTACTTTTCTTCATGAGAATGGCTCTAGAAACCCTTTAGGGTTGGATCTACAATCAGACAAGGTGTATTTTTATCCTTACTTTATGCTTAAGGATTTGCTTGGAGGCTTAGTTGCGATGACTATCTACTTCTCTCTAGGGCTTTATTCGCCTGACCTATTTATGGATCCTGATAATTTTATAGAGGCTAACCCTTTAGTGACTCCTCCGCATATTCAGCCAGAGTGATATTTCCTCTTTGCTTATGCCATTTTACGGGCTGTGCCTAGAAAGCTTGGCGGGGTAGTTGCTTTAGTAATGAGCATTGTATCTTTGGTAGTTTTACTGCTA---------GGTAAAAGTTATTCAGCACGGTTTAGGGTAGCACGGAAAGTACTAGTTTACTCTCTGGTAGTTAGAGTGCTTATTCTCTCTTGACTAGGAGCTATACCTGCTGAAGTGCCATTTGTAAAGGTTAGACAAGTTGCAAGGGTAATTTACTTTACTCTAATCTTACTAATTTTGCAAGGGTTTATAACAGTTGTAATATTGATGGTTTGTGTAGCTTATTTCTCCTTATTCGAGCGAAAGATACTAGGTCTAACTCAGCTCCGGCTAGGACCAAATAAAGTAGGCCCTACAGGTGTTCTTCAACCATTAAGTGATGCTCCTAAGCTTCTAACTAAGTCTATTCAAGCTCAT---ATGGAC------CCTTTTATTTACGCATTTGTTACTTTTCTCTCTTTTTTACTTTCTTTAACTTATTGATTTAGTATTCCGATTAAGTTTACGCCATGGAGGTGTGAAAGAGATAACATGGGTATCTGGATTATTATCGGCTTGAGCCTGGTTGTTTATGGTCCTGTTTCTTGTGGTTGATTCTCGCACTCTAAGTTTTCAGTCTTAGGTAGAACCCGTGCTATCGCTCAGTGTATTTCGTTTGAACTTGTCCTTTCAGTTATCTTACTTTCGGTATTTAGGTTTTCATCCTGTACTTCCGTTCTAGGAATTTGTATAGCACAGCTTTATGCTCCTAACTTACTGACAGCCCCG---CTACTAGCTTTCGGGGTCTGAATTTCTCTTCTTGCTGAAAGAGGTCGTAGCCCATTTGATTTACCTGAAGGTGAAAGGGAATTGGTAAGAGGGTTTAATGTGGAGTACGGAGGTGTCCGGTACATTTTAATTTATCTGAGAGAAAGTGTGACTCTTATGGTATCTTGTATGATTATGAACAGTTTATTTTTAGGA---GTAACCTACTTGCTACCTATACTAATTTGAGTGGTTATGAGT------------ATCCTTCTACGATCTAGATTACCTCGGATGCGGTTTGACCATTGTATAGCGCTAGGGTGAGTAACTTTATTATCTTCTGCTATCTCTTATGCTTTATTAGTTGTGGTTGTTGCCCTAGTGGCTGTGTTTGCG------------------------TTTTTATCAGTTACTCTCTCTTCACAGGAGGTAGAA---GGATACGACCATTCCCCTTTTGAGTGCGGTGTTATGCCTTATTCTACTTCGTACTCACCATTTTATGTCCAATTTTACTGCATAAGAGTTATTTTTTTGGTATTTGATGTAGAAATTGTTATCTTATTGCCTATAGTTGAGGCT------TCTTTACCTGAAGTTAGAGGAGTTGTATTT---TGGTTGCTTACGTTCCTACTTTTATTTATGGGATTGTTAATTGAAATCGGCTACGGGTCTTTAGAGTGGAAGTTTATGTCTTCTTTAATTTCTATTGAAAGATGTTGATGTTGGTCGTACTTTGCTCTTATGTTCACTGTTGGTCTA---GATGTAGATAGGCTAATTATTTCAGAGATTTTATCTGTTATGGCTTGTGAAAGAGTAATTGGGCTAACTCTCAGCTCTAACCTCTCAAGCAGTAGTTGTTACTCTTTTCTTCTAGATTTAGATATAGTATTTATTTTTGATCGGGTATCTGTCATGTTTTTAATGATGGTGTTAGTCGTTAGGGCAGTGGTGATAGTTTTTGGTAGATTCTATATGACTTCTTCTTTACATGGTGTTGCTTTTACATTTAGGATATTGATTTTCATCGCCAGGATGGTTATTTTATCTGTCACAGGATCTTTGTTTTGGTTATTTTTAGGGTGAGACGGTTTGGGCTTATCTAGGTTCATTTTAATTATTTTTAACAAGAATTGATCTTCCTCTAAATCAGGGTTAATTACTTTTTTAATAAACCGGTTAGGGGATGTGTTGATAATCGTAGCCTCTTCGTGGTTACTTATTCAAGGCTCT---------------TTCGGTTCAGGGCTTTCT---------------------GCTCTTGGAAGAGTTGTATTCACTATCGGTTGTTTGTCAAAGAGAGCTCAGTTTCCTCTTTTAAGATGGTTACCCGAGGCTATAGCAGCTCCTACCCCTGTAAGAAGGCTGGTTCACTCTTCAACTCTGGTTACTGCAGGCATTTATACACTAGTTCGGTTTGGTAGC---GAAGTCAGAAATTGGGGTTCT------------ATCGTTGCATTTTCTTTTTTTTCGTTAGTTGTATCAGGGCTTTCTGCTCTTGTTTCAACGGACTTAAAGAAGGTAGTAGCATATTCTACCCTCTCTCACATTAGCCTTATAATTCACTATCTCTCAGAGGGCTGTATTGACGCTTGTATACTTCATATAGTTATGCACGCAATCTTTAAGAGTCTTTTGGTTTCTTTAATTTCGATAGCAGGGCTACCTTATTTAAGAGGTGGATTCTCTAAAGAGGTT---GTT---------TTAATACATAGCCTTAAGTTCGGGTTGTTTAGAGCAAGGTTCTTTCTGGTTTCTGTATTCTTAACTAGGTCTTATTCTGTGCGAATTATGTGGATCTTATTTAGCCCC---------------------TCTAACTTTGGGTTAAAG---------TTAGGCTTAAACACAGCCCTATTCTCATTCTCTTTGAGTCTAGGTGTGTACCTCAATATGATGGTGGTATTCTGAGCAGTCTGA---------ACTCCTTTTTGAATGGGGTTTATA------GCCCATGAGTCGAGGGTCTCTGTACTAAGAAAGACTCTAATGATTAGAGTGGCGTCACTAGGATTATTGAGATTAGTGTGTTTAGTT---TACATGAGGTTGACAAAA---AGGTTAGTTATTATGTTATTGGATATAGCGTTTATGGGGGTGATTATTAAAGTTCTTCTCTCGTTGTATGTTACTTCTAACTGGCCTTGACTTTTACTCCTGCTGGGTGTCGTCGGAGGTTTAATCGTAGTGATTTCTGTTATTCTGGTTGTTATGCCT------------GGGCCTAGCGCCTTACACTGGAGGGTTGATAAA---------------------GGGTCTACAGTGACTTTTGTGCTTGTGCTAATTTTGGCTGTGGCTATT------------------------AGCTAC---------TTAGACGAGTTAGATAGCTTTTTTAGCCTGACT---TATAGGACATGC------------------CGTTTAAATATACTG---TCAAACTCTTCATTCTTGTACCCTGAGGGGGTGTTAGTACTGCTAATTGGGCTACTTTTAATACTTCCTGTCATAGAGGTCTTGCTTTCGTTTTATGGGCGT

Polyplax_asiatica ATGTCAATTTTTGACCCAAGGCTTGGGGTTGAGGGG------CTTTCGGTA------AAATGGATTTGAGGGTTA---------------------GCCCCTTTCTTGCTTTTGAGCGGAGGCTTTTGGGCCTTGTTGTCCGGCGTGACAGGAGTTCTTTCTATGTTTGGAAAGCAAGTATCAAATTCAGTTCTACCTTCGCTGAAGGAAGGTAAG---------CCTTTAGCTTTGAGTCTAATGGGGGTTTACATGGTGCTCCTAACAATGAATTTATTAAGATTGAGCCCATTCTCTTTTTGCCCCCCTTCCCATTTAAGCTTTGGGCTTAGGGTGTGCTTTCCCCTTTGGGTTGGGGGTTTAATTTCTTCT---TACAAGAAAAGTAAGGATAAATTTCTATCTCACTTTCTTCCACTAGGAACGCCTCAAGGTTTAAGGTGATTTTTAGTGCTAATCGAGTTAACTAGCCAAGCCGTTCGGCCATTGAGGCTGAGGGTTCGGCTAATGGCCAACCTAACGGCTGGACATATAATCATGAGTCTTGCCGAGAAAGCCTCTTTACTATTTCCATTATTATTTAAACTCCAGTTCTTGATAATTCTATCACTTTTACTTGTGTTTGAGTTTGGAGTGGCTTTAATTCAGGCTTATGTGTTTATAAGCCTGCTCTCTCTTTACTGAGATATAGCTCCAACTTATTGAGTTATTTTACTTTTATTAATTCAAACTATCTTCCTAATAGGGAAAATG---GGTTTATATTTTTTATTAGTAAGCAGGCTTGCTGAGGGGCAAGGAGTTAAGTCTCATAAGTCTTTGGTTAATCAGGATTTTTTGCTCTTCTACTCTACAAATCATAAAGATATTGGAGTATTATATTTAGTATTTAGGGCGTGAGCAGGTTTGCTGGGCTATGGGCTTAGCTTCTTGATTCGCTTAGAACTTTGTCATCCTGGGAATTTAATGGAAGACGGGCATGTCTTCAATAGTTTAGTGACTGCTCATGCTTTTGTGATAATTTTCTTTATAGTAATACCGGCTATGGTAGGGGGGTTTGGCAACTGGCTAATTCCCTTGATAATTGGGTTTCCGGATATGGCCTTTCCCCGAATAAATAATATGAGTTTTTGGCTTCTACCCCCTTCTCTTTTTCTTTTGCTTAGAAGAGCTTTAATCCAGGGAGGAGTGGGGACTGGGTGAACGGTTTACCCTCCTCTTAGAAGGAGGGTTGGTCAGCCCGGAGCTTCAGTGGATTTGGCTATTTTTAGCCTTCACCTCGCGGGGGTGAGGTCAATCCTTGGAGCAATTAATTTTATTTCAACAATTATAGTTGGGTGGAAATTTTCC---CAAATTGATAAGCTTCCATTATTTTGTTGGGCAATATTGATTACAGCGTTTCTCTTGTTACTTTCGCTCCCAGTTTTAGCCGGGGCTATTACTATACTTTTATTTGATCGAAACTTAAACTGTTCATTCTTTGACCCTTCCGGGGGAGGGGATCCAGTTTTGTACCAACATTTGTTTTGATTTTTTGGTCACCCTGAGGTTTATATTTTAATTTTACCTGGGTTTGGACTTATTTCTCACATAATTTCCGATGAAATGGGGAAGAAGGAGGTTTTTGGGAGTTTAGGGATAATTTATGCCATGAGGGCAATCGGGGCTATAGGATTTGTTGTTTGGGCCCATCACATGTTTACTATTGGGATAGATGTAGATAGTCGGGCTTATTTTACAAGGGCCACTATAATTATTGCAATTCCAACGGGAATTAAAGTATTTAGGTGGCTAGCCACCATTATTGGCGGGAAAGCCCCCCGGAGCGTTTCAATGCTTTGGGTAGCCGGGTTTATCTTTCTTTTTACAGTTGGGGGCCTAACAGGTTTGGTTTTAGCTAACTCTTCAATTGATGTAGCCCTCCACGACACTTATTATGTTGTTGCTCACTTTCATTATGTTCTTTCCATGGGGGCAGTCTTTGCCATCTTTGGCAGATTTAACCACTGATTTCCCCTTCTCAGAGGGGTTGTCTTGCCCCAGAAATGAATAAAGGCTCACTTTTGAGCTACCTTTCTTGGAGTAAATTTGACTTTCTTCCCTCAACATTTCTTAGGACTAAGGGGGATGCCCCGTCGATATAGGGATTATCCGGATTCTTTTAGGTGCTGAAACACTCTTTCCTCAGCTGGAAGGTCAATTACCTCAGTCAGGATATTAGTGTTCATTGCCTGCCTTTGAGAGGGCCACCTCTCCTGCCGTGTCTCCCTTTGACACGGAGCC---TGGGCTAGGCTGGATTGATTTAGGGGGTTCCCTCCCTCGGAGCACTCACACGAAAGACCCCCTCAGATTTTCCTAGTTAGCCTTCAAGACTCTAATTCTCCTTTGATGGGACATATTGAGAGGGTACATGATTGACTAATAGTTGTACTTGCTGGTATCATTTCCGTAGTCATTTATGTAAGAATTTGGACCTTTTTGAGAAAGGAATGA---------AACGTTTTCTTTTTTGACAGGGAGTGGTTGGAAGTCATTTGGATTATGTTCCCGTCTATTGTTCTTCTGACTTTAGCCTTTCCATCCCTTCAATGTCTTTATTTGCTAGAGGAGGTAAGACTTCCCAAGTCAACTATTAAGGCAGTAGGGCACCAGTGGTACTGGAGGTATGAATTGGTCTCTCCTACGAGCCTAGAGACCTTCTTGTTTGACTCGTACCTTTTGCCT---AAGGAGTGGGAGACTGGGGCTGCACCTCGGCTCTTGGACTGTGACTCCTCAATTCTTCTTCCAGTAGGAGAAGAGACTCGCTTAGTAGTAAGAAGAGGAGATGTAATTCACTCTTGGGCACTTCCAAGAATAGGAGTAAAGGTGGATGCAATCCCGGGTCGCTTAAACCAAGTTATTCTTTACCCATTAAAAAGGGGAATTTCATTTGGCCAGTGCTCCGAGATTTGCGGGGCAAATCACAGATTTATGCCAATTAAAGTTGAAGCCATTCCTCGAGAGGAATGGATTTCCATTCTCAAATTGAGGAAGTTTCATCCTTTTCATATTGTCAGAGTAAGGCCTTGACCTTTACTCTCTAGGGCTTCGGGGTTTACGCTGGCTGTTGGCTTAGTTGAGGCATTCTCG---GGAATAGGTTGATTCTTGGTAGGCTGGGGAAGCCTAAGTAGAGGGGCTTTAGCTGGCCTATGGTGGCGGGATGCCATTCGGGAGTCCTTCTTGCTCGGCGAGCACACGATGGAAGTGACTCGAGGCTTACGAGTCGGAGTAATTCTCTTCATTCTATCGGAAGTAATGTTCTTTTTTTCCATTTTTTTTGCATTCTTCTTTTTATCTTTGAATCCCGATGTTAGACTGGGGGGCCAGTGGCCTCCGGAGGGGCTCTCTCCAGTGCCGTACATGGGAGTTCCATTAATAAACACTGTCCTTCTATTATCTAGAGGGATTTCTTTAACATGGTCCCACCATGCCCTAATT------GGGGGCATGGCCTTTCGCTCTGCTTTTCCCCTGCTGATTAGGGTTCTTTTAGGAGGCGGGTTTTTACTTCTTCAGGCGGAAGAATATAGGGAGTGCTCCTTTTCTATCTCTGATAGAAGATTTGGCTCTTTATTCTTTGTAAGAACGGGGTTTCATGGGGTTCATGTGATAATTGGGACAGTTTTTCTGTCTGTCAATCTTGTTCGAGTTATTTCTGCCCATTTCTCCCCCCACCATCACCTGGGGTTTGAGGCGGGGGCCTGGTACTGACATTTTGTAGATGTTGTTTGACTATTCTTGTTTATTACCATTTACTGATGGTTTATGTGGAATTATGGGTCTCTGCTGGGCTTGTGCTTGGGGTTACAACTTGTAACTGGGTTATTTCTCGCTATACAGTTTTCAGCTGCCCAAGGTCTCTCTTTTGAGAGAGTTTTAAGAATTATAAATGATGTAAAAGGGGGATGGGCAATCCGGCTTCTTCATGCCAATGGGGCCTCCTTGTTTTTTATTCTTATCTACCTCCACCTAGGCCGGGGCCTATATTATGGAAGGTATCGGCTGATA---GGGGTTTGGATGGTGGGGGTTATAATTGTCTTTACCTTAATGGGTACTGCATTTTTAGGGTATGTTCTGCCCTGAGGACAGATGTCCTTTTGGGGCGCCACTGTAATTACAAATTTAGTGTCAGCTATTCCCTACTTAGGAGAATCTCTCGTAACCTGGATTTGAGGGGGGTTTAGGGTTGGAAACCCTACTTTAACCCGGATATTTTCACTCCATTTTATCCTTCCATTCATAATTGTGTTTCTGGCTCTCTCTCACCTTTTCCTTCTTCATGAGAGCGGAAGCTGGAACCCCCTGGGGTTAAGGGAGGATTCCGACAAAGTGGCCTTTCACCCTTATTTCATCTCTAAAGATTTCTTAGGGGTGTCCGTTTTAGGGAGGTTAACTTTGGCTTCCCTATTCCTCTTTCCTGATTTGTTTATAGATCCAGATAATTTTACCCCTGCTAACCCCTTAGCCACTCCAGCCCATATTAAGCCCGAGTGGTACTTTCTTTTTGCTTACTCAATCCTTCGTTCAATTCCTAGGAAGCTTGGAGGGGTGGCCGCCTTAGTTTTAAGGATTTTAGTCTTAGCCTTTCTTCCATTA---------ACGAAATCCTCTAGCGGCCGGTTTTCCTTCCTTCACTCCTTTACCTTTTGATTTCAGTTAAATAATTTCTTAATCTTAACGTGGCTAGGAGGTGCAGCCGTAGAGCTGCCCTTTACTCTTGTAAGTAAATTTATTACTTTCTCCTACTTTTCAGTATTTATTAGGTGACTTCCCTTGCTAATTTCATTACTAGCGGTTTTAATCATAGTTGCATTTTTTACTCTTTTTGAGCGAAAATTCCTTGGAATCCTTCAGACCCGCAGGGGGCCAAGGAAGGTTGGGTTTTGAGGCCTCTTGCAACCTTTTAGGGACATGTTAAAGCTTGTCACTAAGACTACGCCAAGCCCG---GGAAAAGGCTTACCTGCCGGATACCTTTTGGGACCTTTTGGAATGCTGATTCTCAGGGGATTAGTCTTCGCCTTTTTACCATTTAAGTTTTCTAAAGAA---------AATGAATTTAGAGCAGTAACAGTTTTGATGGTCCTAAGAGTCTCAAGATTTCCTCTTATCATTAGAGGATGATTCTCTCGTAGGAAGTACTCAGTGCTCGGGAGACTCCGAAGAGTCTCTCAGCATATCTCATTCGAAATCCCACTCTCCACGTCAATTATTTCCATTGTTATTTTAAACCAAACAAGAAGGATTCAT---GAAATTTTAATCCACAGGGGGGTCTGAGTCTCCTTTTTTTGCCCC---ACTCTTCCAGTTCTGATAACTTTAAGTCTTAGGGCCGAAGCCTCCCGATTACCATTTGATCTACCAGAGAGAGAGAGTGAATTAGTCAGTGGGTACTCCATTGAGTACGGCGGAGCAATGTTCACAATTATCTTTTTAGCTGAAGCTTGCTCCCTCTTACTACTTAGGGGCCTATTTTCTTCCCTCCTTTCCGGA---AGGATTAGACCACCCCTTTCCCTTTCACTGCTCTTCTTAGCA------------GTCTGAGCCCGGGGGGTCCTCCCTCGAGTTCGCTACGACCTAATAATGGAAGTTTGTTGAGTAGAGCTCGTCCCTATAGCCCTGTCTACTCTATGGATAGCGAGTTTGCCCGGATTAGTGATTTTTCTTCTTTCTTTGGTAGCTTTGGCTATTGTCTTAGTAGCTTGCATGTTCTCTGAAAAAAATATTTACTCTAAGCAAATGTTAGAGGCTTTTGAGTGCGGGTTTAGCCCAATTGGTCACCCTCACGGGCCCCTAAGGGTTCAGTTTCTACTTGTAGGAATCTTATTTCTAATCTTTGATATTGAAGTGATTATCATTCTTCCAGTTTTATTTTTA------AAGGTTAATTTTCTTCAGTGAGTTTTGTTC---TGGACATTTTACTTTATAATCATGCTTTGAGGCTTACTAGTTGAAGTAGAATTTGGAACAATCAGCTGAGGAGCAATTACTGCTTTGGTTAGCGCTGAAATTATTATGAATGTAAGGTATTGTTTAGGAACAATAAGCTTTGAAGGGACGGGAGTCCAGGGGTTTATTTGCTTAACTATGCTGGTTTTCTCAGTGGCAGACAGGGTTATAGGGCTTACTACCCTAAGGACTTCCTTTATAAGAAGAGCTCACTTAGCACCTTCAGAGCCCTATTTAGGTTTAATGCTGGATTGGATTAGATTTCTATTCCTCTTAATGGTTTTAGGGGTTAGTAGAAGGGTTTTTCTATATTCTAAGGGGTATTTTAAGAAGGATGAACAT---AACAAGTTTTTCCCAATTCTTAGCTCATTTGTGGTTTCGATATTGGTTTTAGTTTCGAGGAAGGGGTTCTTTATAGCCTTGGTAGGCTGGGACCTGCTGGGCATTAGGTCCTTGTGCTTAATCTTTTACTTTAAGTCATGGTCTTCTTACAATGGAGGACTGGTAACGTTTTTAAGGAATCGATTTGGGGATCTACTCTTGTTTAGGAGCCTAGGTTTACTCCTTGTTGGAGGGAGGGAGTGAAGGTTTCCCCTCAGTTCACCAGGACCA------------------------TGGGGCAGGGGTCTCATGCTCTTAGGGGCAATAACTAAGAGGGCCCAATATCCATTTTCAGCGTGACTTCCTTTGGCCATAGCGGCACCTACCCCAGTTAGAAGTTTAGTTCATTCCTCCACCTTGGTTACTGCTGGTCTTTTCATTGTTGTTCGACATAGTCCTTCAACCTTTCCTTCAGTATCGTGGTTAGGGGTT---------TTAGTTTCTTTTGCCAGAGTTATCTACGCTTGCTCTAGAGCTTTAATCGAGGTGGATTTGAAGAAAATCATTGCATTTTCCACTTTATCTCATTTAGGGCTAATGGTTCTCTTTGTATCAATGGGGAGAATTGAGGCGGCCCTTGTCCACATGCTTAGCCATGCTAGGTTTAAAAGGATGACTTTTTCTTTAACCTCAATAGCCGGTTTACCTTTCCTAAGGGGATTTATCTCAAAGGAAAAT---CTGTTTTGC---AATTTAAATTCTCAATGGGAAGGCCTTTTGCCAGCCCTGACCTTATCCCTAGGGACTGTGGGGACAGCGGCCTACAGCCTCCGGATCCTTCTCTTCTTACGAAACTCTCAA------------------GCCTTTAGCCCGTTTTCTTCGAATTCCTTTGGGGAATCAGAGAAATGAATGTCTCGAGGCATAATTCTTGGACTCATTATTAGATTATCTCTTTGTATTTTCCTTCCTTGG---------------GGAATCAGTGAGGAAAAG------AGCTCTCCCCTCTTAATAGAGAGGAGGACTAAGTCCTCGGTTTTCATTTTTATGCTTATCGGGATTTTATTTGTTTTTTTCTCAATT------CACTGAATGACTGAA---AGAGTTGTCACTAGTAGAATTTTTATAATTCTTTTTTCCTTAGGCATCTCTTGAGAGGTTTTCAAGCTCAGAAGATCAGTCTGACCGTCTATCTTATTCTTTCTAGGTATTTTGGGAGGACTGGTTGTCCTACTGGCTTATTCGTTTATGCTGTTCTCT---TGA---AAAGAGCAGGGGAAAGCTTTCTCAAAGGGCTTTGAG---------------------TTTAGGGGGCTACAAATGGTAGTATATTCGCCTCTAGCTTTAATCCTT---------------TGGATCCTTGAAAAGGATTCTCTTACTCTTATCGGAGGGTCTAGCAGTAAATTCCAGCCTTCTGCTTGAAGG------------------GTAGCTTCAATAAGC---TTTGACTATAGGAATCACTATATCTATTCAATTCTCTTTTTATTTAGAACTTTATTTATCATTTTATTTTCGATTGATGAAATTATCAAAAATTTCAAAAAC

Polyplax_spinulosa ATGACTATTTTTGACCCTTCGTCA------TTTAATTCCCTTGTACCTTTG------AAATGGGGCAGAAGAGTT---------------------TTTTTTCTAGTTATCTTGAGAGGCGGTTTCTGAGTCGTCAGGACTGGTTTTAAATTGCTAGTAGAGATGTTCTTATTTAACCTATGTCAAGGATTTAAAGTAATGTTTCTAAATTGGAAA---------CAACACTCTGCCATGTTAATTGGTCTGTTTTACTTAATTTTAACAATAAATGTAGTAGGGCTGTTACCTTTCTCATTTTCTGTAACAGCTCATTTGTCTTGGAGATTGACAATTTGCCTCCCCATATGATTGGGAGGGGCTATCTACATG---TTCTCAAAGGACAGAGAAGGAGCCTTGGCTCATTTTCTCCCTCATAGGGCGCCCATAGGCTTAGCGCCATTTTTAGTGATCGTAGAAATGGTGAGAATGCTTATTCGCCCGTTGAGGTTAAGGGTTCGGCTGATGGCTAATATCACAGCTGGACACATAATTTTGAGGTTGATTGAGACTCTTATCGTTAGG---AATTCTCTTTCTGTTAAGTGATTTACTCTCTTAATAGCAGGATTTCTCCTTTTTGAGTTAGGAGTGGCTTTGATCCAAGCTTATGTGCTGATAAACCTTTTGTCTCTATATTGAGAGATAGCTCCAATGATATGGTTATTTTTTAGCTTAATAACTAATTTAATTTTTCTTTTAATAATTGTT---TTAGTAACTTTCGATAAGTTTTTCTTCTGTTACCCAGAAGGTGAGAAGAAATATTCTCCTAAGTATAAAACTTGTAGTAAAATTTTCATATTTTCCACTAATCACAAGGACATTGGCGTTTTGTATTTACTATTTGGGTTTTGGGCAGGTTTAGTAGGATTTGGTATGAGAGTAATTATTCGAATTGAACTTTCTCAACCAGGTTTATGGATAGAAAGAGGACATGTTTTTAATTGCTTGGTCACTTCCCATGCATTTGTAATAATCTTTTTTATAGTTATGCCAATCATAATTGGAGGGTTTGCCAATTGGCTTGTCCCTCTGATACTTAGTGCTCCCGATATGGCTTTCCCTCGAATAAATAACATGAGGTTCCACCTTCTTCCACCGTCTTTTGTTCTTCTCTTATTGAGCTCTCTTATTCAAGGGGGAGTGGGCACGGGATGGACTGTGTACCCGCCTCTAGCTAGAGGGTTGGGGCAGCCAAGAATTTCGGTAGATCTGGCTATTTTTAGGCTCCATTTGGCAGGGGTCAGGTCTATCATAGGAGCAATCAATTTTATTTGCACGGTTATCAATTTTTGAAAGCAA------ACGATAGAAAGATTGCCTCTTTTCTGTTGAAGGGTACTAATCACTGCCGTTCTATTACTTCTTTCTTTACCCGTATTGGCAGGAGCTATTACTATGCTACTTTTTGATCGAAATGTTAACAGGTCCTTTTTTGACCCTTCCGGGGGAGGGGACCCTATTCTCTATCAGCATCTGTTCTGATTTTTTGGGCACCCTGAGGTATATATTTTAATTCTCCCAGGATTTGGTCTTATTTCCCACATAATTGTTGATGAGAGAGGTAAAAAGGAAATTTTTGGAAGTCTAGGGATAATCTATGCTATATCTTCTATTGGAGTGATGGGATTTGTGGTGTGGGCGCACCACATATTCACTGTTGGGCTAGATGTAGATAGGCGAGCTTATTTTACTAGGGCAACGATGATTATTGCTATTCCAACAGGAATTAAAGTCTTCAGGTGATTAGCCACTCTTTTTGGAGGATCTTCATTTCAAAGAGTCTCTGGTCTGTGGAGAATGGGATTTATTTTCCTTTTCACTGTTGGAGGGTTGACAGGGTTAGTCCTCTCTAACTCCTCGATTGATGTAGCCCTTCATGATACTTATTATGTAGTGGCTCATTTCCACTATGTCCTATCTATGGGGGCAGTATTTGCAATTCTTGGCAGGCTTAATCATTGATTCCCATTGGTCAGAGGGTTGGTACTAAACCAGAAATGAATGGCTATTCACTTCTGAGTGACATTCCTGAGGGTCAATTTAACCTTTTTCCCTCAACACTTCTTGGGTTTATCCGGAATGCCCCGCCGGTACATTGACTACCCTGATTCGTACAGAGCTTGAAATCTAACCTCGTCAATAGGAAGCTCTCTATCCATCGTGAGAGTGGGAATTTTGATTGGGTCTTTACTCGAAGGTTTCATGGCAAAGCGTTTACTTTTATTCCCCTTGTAT---CAAAGAAGGGTAGAAAATATTTTAGGAACCCCTCCTAGGTTTCATAGAAACGAGAGGGCACCAGCCTATTCTATTGTTAGCCTTCAAGACTCTAATTCTCCTTTGATGGGACATATTGAGAGGGTACATGATTGACTAATAGTTGTACTTGCTGGTATCATTTCCGTAGTCATTTATGTAAGAATTTGGACCTTTTTGAGAAAGGAATGA---------AACGTTTTCTTTTTTGACAGGGAGTGGTTGGAAGTCATTTGGATTATGTTCCCGTCTATTGTTCTTCTGACTTTAGCCTTTCCATCCCTTCAATGTCTTTATTTGCTAGAGGAGGTAAGACTTCCCAAGTCAACTATTAAGGCAGTAGGGCACCAGTGGTACTGGAGGTATGAATTGGTCTCTCCTACGAGCCTAGAGACCTTCTTGTTTGACTCGTACCTTTTGCCT---AAGGAGTGGGAGACTGGGGCTGCACCTCGGCTCTTGGACTGTGACTCCTCAATTCTTCTTCCAGTAGGAGAAGAGACTCGCTTAGTAGTAAGAAGAGGAGATGTAATTCACTCTTGGGCACTTCCAAGAATAGGAGTAAAGGTGGATGCAATCCCGGGTCGCTTAAACCAAGTTATTCTTTACCCATTAAAAAGGGGAATTTCATTTGGCCAGTGCTCCGAGATTTGCGGGGCAAATCACAGATTTATGCCAATTAAAGTTGAAGCCATTCCTCGAGAGGAATGGATTTCCATTCTCAAAAAAACAAAATTTCATCCCTTCCATATTTTGGATAATAGGCCATGGCCTGTGATTATAAGATTAAATATTTTAAGGGTTGCTTCCCTTCTGTCCAGATCTTTCTTT---TGAGGAGTTTCCTTACTTTTAATTGTTCCGGTCCTCGGGCTATTGTTCTCGGTATCCTTGTGGTGACGAGATGTGGTAGCCGAAAGGCTGTTTCAAGGGAATCACACAAAGGAGGTAGTTAGGGGCTTGCGAGCAGGTGTCTTAATATTCATTTTGTCCGAGGTTATATTCTTTTTTTCTATCTTTTTCGCCTTTTTTTTTATCTCTCTTTCCCCTGATGTGAGAGTAGGGATGGAATATCCTCCCGTAGGAGTGGGCTCTTTGAACATTTTTAGTGTTCCTTTGTTAAACACAATTATTTTGCTATCAAGAGGAGTTTCGCTAACTTGGAGGCACCATTCTTTACTC------GAAAAAAACTTGTTTAATTCAAACCTAGGTCTGTTAATTTCAATTTCTTTGGGGTCATGATTCCTATTTCTGCAGAATAAGGAGTATCTAGACTGTCCATTTGATATTTCTGACAGAGTGTTTGGTTCTTTATTCTTCATAGGGACTGGATTCCACGGTCTTCATGTTTTAATTGGGACAATTTTTCTGCTTATCAGATTAATTCGTTCGATAATAGGACATTTCTCTCCCTGTCATTGCTTGGGGTTCGAGGCTAGGGCTTGATATTGGCATTTTGTTGATGTCGTTTGGCTCTTCCTGTTCGTCACAGTGTACTGGTGATATCAATGAAATTATGGCTCTCTTCTGGGGTTTATCTTTTTAATTCAGCTTTTTAGAGGATTTTTTTTAGCTTTGCAATATGAGGGCTCCTCGATGTTATCTTTTAAAAGAGTAATCTCTTATATACAAAGAGTTGAGGGGGGCTGGGCTATTCGATTTGTTCACGCAAATGGAGCGTCTTTCTTTTTTGTGCTCATTTATTTGCATATTGGCCGAGGCATTTACTACGGAAGGTACAAAAATCTT---CTTGTTTGGGTCACAGGGGTAATAATGATTTTTATTTTGATGGGGACAGCGTTCCTAGGCTATGTGTTGCCTTGAGGGCAGATATCCTTTTGGGGAGCTACGGTCATTACTAATCTACTTTCTGCCTTTCCATACATTGGTGAGAGCCTGGTTTATTGATTGTGGGGCGGGTTCAGAGTGGGCAGGCCTACCTTGACTCGAATGTTTTCTATCCACTTTCTTATGCCATTTATCTTACTAGTTGTTGCCCTTTCTCACATTGCTTTTCTCCACGAAAAGGGAAGATCAAACCCCTTAGGGCTAAGTCCTCATTCCCATAAAGTAGCATTTCATCCTTATTTTGTGGTAAAGGATGTTGTTGGGCTAGTTGTGGTAGGGTTGCTGTTTGGGGCAATAATTTTGCTTAGTCCTGATTTGCTGATGGACCCAGATAACTCTATTGAGGCAAACCCAATGGTCACTCCTCCGCATATTCAACCCGAATGGTACTTTTTATTCGCTTACACCATTCTTCGGTCAGTGCCCAGGAAATTGGGAGGGGTGATGGCTATAATCATAAGAATCTTGATTTTGTTGATTTTGCCCTTT---------TCTGATGCGTCATCTGGCCGATTCAGGCTTATCCGCAGGGTAAGAACTTGAATTCAAATCAATAACTTTTTCCTTCTTACGTGGCTTGGAAGAATGCCAGTGGAAGCTCCTTTTGAGATAACAAGAAAGTATGTTACTTGTTTTTACTTTCTAACATTCCTCTTTTGATTTGCAGAGTTGATTTTATTAGTCTCGTTTCTTCTTATAGTTGCCTTTTATACTTTATTTGAACGAAAGGTCATGGGCCTTTCCCAGAGACGGCTTGGGCCTTCCAAAGTCCTTTTAAAAGGAGTGGGACAGCCATTTAGGGATGTGATAAAACTTTTATCAAAAATATCTCTTTCTCGG---AGAAATGAAGAAGAATTATGATACACCCTGGCTCCTTGCATGATAATAATTGTGTCAGTGAGGGTATTGGGGGCTTTGCCCTTTTATTTTTTCCCTTCA---------TACCAAACCTCTGGGGTCATTATCATATTTTTATTAAGAGTGAGGGCCTTTTGGCTCACATTATCTGGTTGATTTTCTAATTCGTCCTACTCAACTTTAGGGGCATGCCGAAGAGTCTCTCAATCTTTATCATTTGAAATTCCTTTAGCACTTTGTTTTATTTCCCTGTTCCTCATCTCTAAAAGGCTTAGTGTTCGG---GATTGAGGT---CAACTCGAGTTGACCGTTCTACTGGTTGCGCCC---TGAAGAGGATTAATTCTTTTGTTTTCATTCATTGCAGAAGCAGGACGGAGACCATTTGATCTTCCCGAGAGAGAAAGAGAATTGGTCAGAGGCTTCAATGTTGAGTACGGAGGCTTACTTTTTACTCTTATTTTTCTAAGTGAAACTCTCATAATACTAGTCATCAGTAGAATATTTTCGATTATTTTCCTCCAC---CAGTGTGAAGGGTGACGATTCGTTTTATCTTTATTTATTTTG------------ACTCTTATCCGTCCGTCGGTCCCTCGTATTCGATTCGACCAGGCCATGACGGCAGCGTGGCTTTCCATGACCCCGACTGCAATTAGAGCAGTCTTCTTTTTTGTACTCATTGCATTTTGTACCTTACTAATTTCACTGCTAAGG------------GCAGTAGCCCTTCTTTTCCGAAGAAAGAGGGGTAAA---ATTATCTCCTTAGAGCATTTTGAATGCGGGTTTACTCCGTTTCATCCTTCCCGAATAGTATTCTCTATTCATTTCTTTCTCATTGGTGTTTTATTTTTGATCTTTGATTTAGAGGTCGTTTCAACTTTACCAGCTATCTTTATC------CATGTCTCAATTACTCAGTGGGTGATATTC---TGGGTATTTTACTTTATAGTCATGCTCGTTGGACTTGTCTTGGAGTTCTACTGAGGGACATTTCTTTGAATAATTCTATCAGTAATTGCAGGATTAGATTTTATCTCTTCTTTAATTTTCATTGAATTATTTACTCATAACTTTTTATCGGGCTGTGTGGTCCTGGTAGCTGTCATATTTGTCACAATTGTAGCAACAGAAGGGGTGATGGGATTAAGAATCCTCAGTGCGTCAATTATTCGACTAGAAAGGTCCAGGCTGACAGGCGAGGAGGTTTCTTTTTGTTTTGATCAGACGAGTGCTGTTTTCTTATTTATAGTACTAGTTGTATCCACCAGAGTAACTAAGTACTCAAGATATTATATT---AATAGAAAAGATTTACTAAAGTTTCTGTCTCTACTAAGATTTTTTATTCTGTCAATGATTTTGTTGTGTTTGAGAACTAATTTTATCTGAAGATTAGTGGGGTGAGACGGGTTAGGGCTCACTTCTTTATTTTTGATTCTTTATTACAGAAATTGAAATTCAAGAACAGGAGGACTTGTGACTTTTTTGGTTAATCGCCTGGGAGATTTATTTCTTCTATCTTCTATCTTCTTACTGAGAGTGAGAAGGGGC---ATGTTGTGGATAGGGGGAAGAAACAAAGAG---------------------AGTTTGTTTGGCATCTTGTTTATTCTAGGAGTTTTGGCCAAAAGCGCCCAGTTTCCTTATTCATCTTGGCTGCCGCTGGCAATAGCTGCCCCAACCCCTGTCAGGAGGTTAGTTCATTCATCAACTTTAGTAACTGCCGGGGTCTTCATAATTGTCCGGAGCTATTTT---CATTTTAGGGAAGCAACCCTTTTCCTG------ATAAAGATTATGTCATTTTTAACAATCTTTTATTCAGGACTGAGGGCTATTGTTGAACAGGATTTGAAAAAAATCATTGCCTACTCGACACTCTCTCATTTGAGGATCATAGTTTTTTTAATTTCGATGGGCAGCCTAGAAGCTGCTTTATGCCATATGTTTATTCACGCTCTTTTTAAGAGCATGTTAGCTTCTATTATTTCCATGATAGGAATTCCTTTCTTGAGAGGATTCACTTCGAAGGAGATG---ATGATAAGT---ATGTCATTTAGGAATTTTGATTCTTTTTGAAATGTATTGGGGATGATTAGGAGAATCTTCTTCTCCTCCGCATATTCAACCCGAATGGTACTTTTTATGAGCCAGAGGGCTCAT---------------CCTAGAGTTTCTTTGAGG------GGCTTATCCAAGCCGAGAACTAAAATGTGCTCATCTCTTTGAGTGTCATTTAAATTAAATCTACTCAGAGGGACTTTTATTTTACCT------------------ATCCTGTTCCCAGACCTGTGTCCGCCCGCCCCAGAGGTAGAAAGGGGAGTTAAGGTGTTAGTCGTCTTATCTCTTCTCACGGGAATATTTCTTATTTTTGTAACTTTC---TTCCTAGCTTTAGGGGCT---TCTCCT---CAGGGAGTGGTAAACTTGATTGCCATGACTTTTTTCTCTGGCTTGTTCATTTTAGCATATGCAGAGTCGGTGTGGCCAGGGTCTATGTTCTTGTTAGGGATCGTAGGGGGCTTAGTTGTCCTGATAAGATTCACTTTCATAATGTTCCCCAAGGAAAGATACAAAGAGAGTTTTAAGTTT---AATCTCTTGAAA---------------------CATTCGTGGCTAACAATAGCCCCTCTGGGG---ATGTTTTTATTCCTGTCGTGAGAGACAGGAGAGAGGACATCGTGAGCTAGAGATTCAAAGGCCTCTTCTTCTCTTAGTCCTTCTTCG------GTTTGGAGG------------------ATGAGAGATAGTCTC------------ATTCTCTTCTCTCCGTTCTTATTAGTATTTATTTTTATGTTTTTATTAATTATTCTTCTTGCGGTTGAGTCAGTGACTAAGTTA---ATTGGC

Haemapinus_apri ATATCTGTGTTTGACCCCTGTTCTACTTTGCTGAGGTTAAATTTACCTTTG------AAATGGTTACTCGTGGTT---------------------TTAGTTGTATTAAGATTGAGAGGGCGTTATTGAATTCTGTCTTCTGGGTTACAGTGTGTAATAGTGTGGGTTAAAAATGGACTAATTCATGGATTACGTGAGTCCTATAAGAACTACAAA---------CAGTTCATACTTATTCTTCACACGTTGTTTTTTTTTATTTTTTCTAGTAATTTTATAGGATTATCGCCTTTTATGTTTACTCTTTCATCCCACTTGGTTTATAATTTAAGATTATGTTTCCCATTATGGTTGGGAGGAATTTTATATTCG---TGATCAAAATGTTGAAAGAAAACATTAGCGCATTTAACCCCTGTAGGTAGTCCAGTAACTTTAGCTCCCTTTTTAGTGTTGGTAGAGACTGTGAGGTTGGTTATTCGGCCAATTAGTTTAAGTGTTCGATTAATGGCTAACATAACAGCAGGTCACATAGTAATTACTCTTGCTGAACAAGGAGCCATGTCTGTAGCTTCTTATGTTGGGAGTTTTTATGTATTGTTAGTAATGGTACTATTACTGTTTGAGTTAGGGGTGGCTCTAATTCAGGCTTATGTGTTCATAAGACTAATGTCCTTGTATTGGGAAATGTCTCCTATGTGATGGTTACTGCTTGAAGTTTGATTTTTTGTGAGTTTTATAAGGTGTTGTAGA---TGTGTATATTGGGAATTGTTTGTAGAGAGGTTAGCGTCTCCAAAAGTATTTATAAAAGTCCCTGTGTTTAAAGAAGGCGATTATGTGTTACTTTCAACTAACCATAAGGATATCGGTGTTCTGTATTTAATTTTAGGTGTTTGAGCAGGTTTATTGGGGACAAGGATAAGGTTGTTGATTCGTGTTGAGTTGGGTAGAGTGAGTAGATTAATTTCTAGTGGACATGTTTATAATGTATTTGTAACAGCTCATGCATTTTTGATGATTTTCTTTATAATTATACCAGTAATAATTGGAGGTTTTGGAAATTGGTTAGTGCCAACGATGTTGGGGGCTCCTGATATAGCGTTTCCTCGAATAAATAATATAAGATTTTGATTATTACCGCCATCACTTTTTTTATTAATCTCAAGATTAATTGTTGGAGGGGGTGTTGGTACTGGGTGAACGGTATACCCCCCGCTTAGGGGGTTGGTCGGACAACCAAGAAGGTCTGTAGATTTAACTATTTTTAGATTACATTTAGCGGGTTTAAGTTCTATCATGGGTGCTATTAATTTTATTTGTACTATTGTAAACATATGGGTTTGTGGAAAAAAATTAGAACTATTACCTTTGTTCTGTTGGTCAGTTTTAATTACTGCTGTCCTACTGTTACTTTCTTTGCCGGTTTTAGCGGGTGGGATTACTATATTGTTGATAGATCGTAATATAAATTGTTCGTTTTTTGACCCTTTGGGTGGGGGTGATCCTGTGCTATACCAGCATCTATTCTGGTTTTTTGGTCATCCTGAAGTTTATATTCTTATTTTACCTGGGTTTGGGTTGATTTCGCATATAATTATGGAGGAGAGAGGAAAAAAAGAGGTTTTTGGAACATTGGGAATAATTTATGCTATGGTTGCTATTGGATTGTTAGGGTTCATTGTATGAGCACACCATATATTTACAGTAGGAATGGATGTAGACAGTCGGGCATATTTTACCAGAGCAACTATGGTTATTGCCATTCCCACAGGAGTAAAAGTGTTTAGTTGGTTGGCAACTCTCTTTGGAGGTAAGCTTGTTATATCAGTAACATTGTTGTGGTGTTTAGGTTTTATCTTTTTATTTACTGTTGGTGGTTTGACAGGTTTAGTTTTAGCCAATTCTTCAGTTGATGTAGTATTGCACGATACTTATTATGTAGTAGCACACTTTCACTATGTGTTGTCTATGGGGGCTGTATTTGCAATAATTGGAGCATTTAATCATTGATTTCCCATCATTAGCGGTGTGAGGTTAAATCAGAAGTTGATGAAAGTTCATTTTTGAATAACGTTTATTGGTGTAAACACGACGTTTTTCCCACAACACTTCTTAGGTTTGAGAGGTATACCACGCCGATATGTGGACTATCCTGATGTTTTTCTATGTTGAAACATGTTATCATCAATTGGCAGACTAGTTAGAGCTGTAGGAGTACTATTATTAGTATATACAATCTATGAAAGACTAGTGAGAAAACGGGTTATTTTGTATAGTTTATCC---GGGAATTCTTTAGATGCTATATTTGGTTGTCCACCTAATGCCCACACACATGAGAGCGTGCCTTTAGTCTTTAAAATAGGATTTCAAGATAGTTCCTCTCCAATGATGGGTTACATTACTGGAGTACATGATTGAATTATAATTGTGGTATTAGTTGTTGTTTCAATTGTTATGTATGTGTTAGGAGGGATAATACTAACAAAGGGGTGG---------GACCGTTTTCTTGTAAGTGCAGAGACTTTGGAGTTTATTTGAGCAGGACTGCCAGCTATTTCTTTAGGGTTGTTGGCCATTCCTTCCCTTCATTGTCTCTATTTAATAGAAGAAGCTTATTCTCCTTTCTTAAGGTTTAAGGTTGTAGGTCATCAATGATATTGATCATATGAATATTCTGATTATTCAGATTTGGAG---------TTTGATTCATACATGTTAAGTCAGGATTCCTTG---------------TTTCGCCTTTTGGAAGTAGATAATGCAGTTGCTATTCCAATGGATTGCGAAGTACGCGTTTTGGTTACATCCGGAGACGTAATTCACTCTTGAACAGTACCTTCGATGGGTGTAAAGAGGGACGCTATTCCAGGTCGGCTAAACCAACTAGTATTAATTGGATCAAAATTGGGATCTTATTATGGCCAGTGTTCAGAAATATGTGGGGCTAATCATAGTTTTATACCAATCAAGGTTGATGTTCTCTCGAAAGATTTATTTATGAATTGATTATTAAAAGGAGGATTTCATCCATTTCATCTAGTTAGACCTAGACCATGACCTCTATTGTTGAGAATTTCCACTTTTTCTCTAATAGTTGGATTTTATACATGAATGTCA---AGTATGGGTAGGGTGTTGATAATGTTAGGCGTGTTTAGAGTACTGTTGAGATTGTTTTGTTGGTTGCGAGATGTGATTCGTGAAAGGACCTACCAGGGGTGTCACACAATACGGGTCATGAAAGGTTTACGTTTGGGTATTGTTATATTTATTATTTCAGAGGTAATATTCTTCTTTTCTATTTTTTTTGGAGTGTTTTTTTTATCTTTAAACCCTGACGTAGTTTTGGGAAGATCTTATCCGCCTGTAGGTATTCAGCCTTTGAATTATATGGGAGTACCGTTTTTGAATACTATGATCTTGCTATCTAGAGGTGTAACTGTAACTTGATGTCATCATGGAATTATG------AGGGGTAATAAACATCATAGAGTGGTGGGGTTGATTATTACAGTGATGTTGGGAGTGTTGTTTGTTATGTTTCAGTTTGAGGAGTACTACGAAAGCTCATATACTATTGCCGATAGTGTATGTGGTTCATTGTTTTACATGTCGACTGGTTTCCATGGAATTCATGTTATGTTAGGTACTGTAATATTAATTGTAAGTTTGGTCCGATTAATAATAAACCATTTTAGAAGAACTCACAATTTAGGTTTTGAGATATCGGCGTGGTATTGACATTTTGTAGACGTTGTATGGTTATTTTTATTTATTTCTATCTACTGATGGTACCTTTGAAACTATGGTTCTTTGTTATTGGTTTGTTTAGTGATGCAGATTGTTAGTGGTATTTTCCTATCTATGCACTATGAGGCGTCAATACTAAATGCATTCTCTAGAGTAGTGTCAATGGTGAATGATGTAAACTGAGGTTGACTTATTCGAACAGTTCACGCTAATGGGGCATCATTTTTCTTTATTGCCATTTATATCCATATTGGACGGGGTCTATACTACGGAAGGTATCGAATAGTG---GGAGTCTGATTAGTAGGGGTGGTATTGTTATTTTTGCTTATGGCAACAGCATTTTTAGGTTATGTTTTACCTTGAGGTCAAATGTCATATTGAGGAGCAACTGTGATTACAAATTTACTATCTGCAATTCCTTACTTTGGTGAAGTAATAACAGGATGGTTGTGGGGAGGTTTTTCTGTAGGAAATCCGACTTTAGTACGGTTCTTTTCGTTTCATTTTGTGTTGCCTTTTGTTATTTGTTTATTTGTATTATTTCACTTGATTTTTCTTCACTGGTTTGGAAGTTCAAACCCCTTAGGATTATCTAACAAATCAGATATGATTTATTTTCACCCGTATTATAGTGTAAAAGATGTTCTAGGATTAATTGTGGCTTTGTTTATTGTTTGCGTTGTAGTTCTTCTTTTTCCTGATTTGTTTATAGACCCTGACAATTTTATTGAAGCAAATCCGATAAATACACCCCCTCATATTCAACCTGAATGATATTTTCTGTTTGCATATTCTATCCTTCGTTCAATCCCTAATAAATTAGGTGGAGTTGTATCTTTGTTAGCAAGGGTGATAATTTTAGCGTTACTTCCACTTTAT------GCAAAAGGGTTTAGATTTCGCTTTATAGGATTAAAAAAGATCTTGTATTGATTTCACGTGATAGTATTTCTTATGTTGACAGTGTTAGGTTCAATGCCTGTAGAATACCCCTATACTGTTATTAGCCAAGTGGTGGCTTTAATTTATTTTGTAAATTTTATATTCCTAGTGCAAATGTTATTTACTATTATATCAATTTTTATTGCTGTAGCTTTTTTCTCTCTTTTAGAGCGAAAGATATTGAGGATTTCCCAAAACCGAGAAGGTCCAAATAAGATTGTTCTAAAAGGGTTTTCCCAACCAATTGGTGATGCAATTAAACTCTTATCAAAATCTACTAGTCTGCCA---AACTTAGGCTTCTATTCGGTATACACCCTAGGCCCCCTAGCTCTACTTAGAATTAACACCTTCTTATGGATTACAACTCCA------TTTCTTTCCAAGTTTATCCACTTTAATCATTCCGGTATAGTTATACTACTAATCTTAAGTGTTACAGCTCTACCTACTATTTATAGAGGTTGATTCTCTAATTCAACATTTAGAACTATAGGAGCAATTCGCTCTGTTGCCCAATCTCTCTCATTCGAGATTACGTTTAGATTCAGACTATTCATTAGATTCCTTATAATTCAATCATTATGTTTGGAAAACCTTCCCAAATTTCAAAGATGGAGCTGA---CTGTTTTGATGTATTCCGTGAATTTCTCTTGTAACATTGATTTGTTTTTTAGCAGAAAGAGGACGTAGACCTTTTGACCTTCCAGAAGGTGAAAGGGAATTGGTAAGTGGTTATACTATTGAATTTGGTGGATTACATTACACATTAATTTTCCTTGGAGAGAATCTTGCTGTAATATTTATGACCATAATTTTCTCCACCACCTATCTTGGT---GGGTTTTCCTTGTGGAAAGCAAGTATGTTAGTATTAATTATT------------GTAATAATTCGAAGGTCTTACCCTCGTATTCGATACGACCAACTTATACAGTTAAATTGAGTCGGAATTTTACCTCAACTTATCTCTTCAGTTTGATTAGTGCTTGTGTTAATTATTATGTTATTCTTATTA------------------------GTATTGTCTGTGTTGATTTCTTCTATATCAGAAACAAGTTTTGATACAAATGAGTCTTTTGAGTGTGGATTTTTTACAGGAAGGGATATTCACTTACCATTTTGTGTTCATTTCTTTGTTGTAGGTATCCTTTTTGTGGTATTTGACATGGAATTGGTAGTTTCACTTCCACTAATTATAGCG------AATTTAAGAGAATTAGTATGATTGTTATGA---TGATTAGTATACAGCATTATTTTGTTTATTGGAATTCTATTGGAAGTAATATGTGGTTCAATTGATTGGGGTATAATAATTGCTCTGTTAAGAGTTGAGTTTCTATCTGTCAGTCAGTTTTATGCTGTCCTTTTTCTTGTTAACCCAAGATCATTAAATTTTAATAGATGTTTAGTGTTATTGTCAATTCTAGTGTTAGAAGGAACCTTAGGTTTGACCATTCTTGTTTCGACGAGTCTAAAAATTGATTCTACAATAATTTGGGATATTGATTGTGTAATGGTTGTAGATGGTTTAAGGTCGTTATTTTTGTTTACAGTTTTTCTTGTTAGATTTATAGTTTTCAACTATAGAGTGCACTATTTTTCTCATTTGGAAGGGTTTAATAAGTTTATTGTTACACTGTTCTTATTTGTCATATCAATGTGTATTCTTTGTTTAAGACCTAGAATGTTTTGAGTGATAGTTGGTTGAGATGGTTTAGGACTGACTTCATTTGGCTTGATTATTTTCTATCAAAATTGAAGGAGGTTTAGGAGGGGTTTATTTACATTTTTAATAAATCGAATAGGCGACATGTTTATAATTTCGGTTATTGTGATACTATCGAGA------------TGCAACATGTTGAAAAGAATATCAATAAGG---------------TCTGTTGTTCAAATTTCCGTATTCTTACTGTTAGGAGCGATAACAAAAAGAGCCCAATTGCCCTTTTCTTCATGACTTCCGCTAGCAATGGCTGCCCCCACTCCGGTTAGGAGGTTAGTTCACTCGTCAACCTTAGTAACTGCTGGAATTTATTTGCTAATTCGTTTTGAAAGT---TTATTCCCGTTAGAGGTGTTACAAGCA------CTTAAGATAGTATCAATTATAACAATTGTTTATGCAGGAGTGTCAGCACTTTGTGAGGTGGATTTGAAAAAGGTGGTAGCTCTTTCTACACTGACCCATTTAGGAATTATAACTTTATATGTGTCTATTGGGAGTGTGATTGCTGCTACAACCCATTTAGTTTTTCACGCATTCTTTAAAAGAGCATTGATTGTACTATCTTCAATAGCAGGTCTGCCTTTTTTAACTGGATTTTATTCAAAAGAAGTA---------------ATGGTGATGTTGGCAGAAGATTCTTTACTTACGCTGGTTTCGTTTTTGTTGGGGGTAATACTAACTTCTGGTTATTCAGTTCGGTTAATAGTATTAATTTTTAAATCA---------------------CCCAATTTTATTTCTGATAAAAAGGATTTTATCCAGGTAAGAGAAGGTTTGGTTGGAGTAAGGGTGAACGGGTTTTTAATTAGAGTGCTTGGTGGAAGTGTGTTACTATGG------GTGGTAATGCCTGTTACGATGGTTATG------AATTACAATTTAAGATGTGGGATCTTGAATAAGCTATTGGTTATTGTGTCACTTGCAGGAGGACTAATTGCAGCATTGTGTTCCTTA---TATTTGTGGATAGCATCA---AGATGTAGAATGGTAGTTTTGTCTTTATCTGTACAAATTGTCACATACAGGGTAATATTGGCAATGAGGGTTACTCATTATTGAATTGTAATTTTAAGGATTATGGGTTTGTTGGGAGGTTTGATTGTCTTTCTATCGTTTATCCTTATAATACTTCCT---AATCCGGATTTAGGTGTTTTTAATAGATGAAAT------------------------------TATGTAGGACTATCAATACTTATATTATTAATAATATCGACTTGTACC---------------------------------------------------------TACCTACCAGTAGTAGATAAGAATGAGCCT------------------GAATTGTTACCAATT---TCACTACATTTATGTGCTTATGGTTTATGTTTCTTTTTATTGCTTTTTGTGTTACTAATTATAGTAGGTATTATAAATTATGGTTATAAGTCTATCTATCTA
