# Supplementary material for: Fragmented mitochondrial genomes in two suborders of parasitic lice of eutherian mammals (Anoplura and Rhynchophthirina, Insecta)
Source: Sci Rep. 2015 Nov 30;5:17389. doi: 10.1038/srep17389 (PMC4663631; doi:10.1038/srep17389)
Supplement: Supplementary Dataset 7 [file srep17389-s8.doc]

17 5360

Alloeorhynchus_bakeri TTTCACTTGACCAGACGGCTTT------TTTCTT----AATGACAGACTT--------------ATATATTTCTTTCCAACTTATGCTTTCCACCGAAAGATTTTTCTAAATAGAAAACTCAACGATTAAACTTTTGGGAGGGG----AGGGTTTCATATTTGTACTTTTATTTATTTTTAAAATTTTGGTTATCCTAATTTACAGTCAGCATTACTTTCCTGCTTTCCTCCATTGTTTCTTATATTTGG--TGATAAATACCAAAATTTGCCACTATCCCCGGACCCAGGCTTATCCTTATGTTTATGAACATAGAAATATCGCCGGTCTTGCGTCGTTACGCAAATATGCGGCACTTTATAGCTCTGGAAAATTATGAAATCAATTACAGGCTTCTATATATCAATATTTATTTTTGATCGCGTTCATATCAGCTAGTTTTCGTTTAGACCTTATCAGATGCCCCTTGTGAGATTTTTATATTTATATTCCTATGCACTATC--AATTTATTCTTGATCCAGAAACAGAGATGTAAAAAA----------ATGAGAAAGTAATTTATCACAACAAAGAATGGACTTTATTATTTGGCTTGGCGGATGTGGACTCTTAGTGTTATCGATGATTAGCACCGGTCTTATGGGAGACAATTAAAGTATGTACGCCAGCTTGTATATTTTTATGTATCCGTATATGGGGTTGGAATGCTGTCCTTATATGGGCCCGAATGCTTCCCGATAAAAATAGTTTGCTTTCCCCTCTTTCTTCTTTACAGAGATGTGAAGGGGCGGACGGTGACGTTACCCCCTTCACAAATGCCAAGGGGCTCGTGATTACATTTTCCTCATTGCGGGTTCTCATCTGGGCGTAATTATTCACATATAAATCGCCTCGGATTCCCGACGATCCTTTTGTTGTCGTGGATACGCCTTTCTTTTTTCTTCCGTTTGCGGGCATACATCTTTACGACGAATTAAACTCTTTTGACCTCGGGGGGGACCATTTTACACATTTTTGTTTTGGCACCGAGTTAATTTATTTCCGGTTGGTTATTCCAATATAGAAGAAGGGAAAAGAACTTGGTCTTGGATATTAGCATATGCATGGTTTTGGTTATGTTGGCCACAATTTACGTGGATGAGTGAACCGGCTATTACTCGCACATATATGCGTCCACGGATAAATTTAGTGCTGCACCTCAGGTGTCATAATTTCCCTCATTTTGGCCTGGTTGTTTTTTTACATGGGGTTACGGGTATCTGCAATCTCATGAATATCTCAGAACTATAGTGTGCCATTCATAGTCTTCATGGGCGTTTGCATATGGAGTTATCATGTACCTTTTACGGATACATAACCCATGCTAAATCATTATATATTTACGGGTAAATACTTTTCCCACATTTTGGTTAAGGATCCCGCGTATCGATACCGAAGTTATACTGAAATATTCTCATGGTCACATTCCTATGGGTATATTTATTTATATTGGAAGATATGCAACGCAGTTTTTCCATAAATAATCAGATGATGCTCAAGACCCCCAGGACATCTAAAGATTCCATATTCACATTCACCAGAGCAATCCCCTATGACACTATTTTTCAGACAACATTTATCTACATATACATATGTATTATTATTCACCTTTATAAAATAAT------AACGTTCTCTGAGGCAACATGATTATTGACATCTCCGCATACTTATTTATGCTTCCTCCTCGATTTTAATATGAGAATAAAACCTTATACATAAGCATGGCACATGTTTGAGTAGATATCGATTAAAAATGA------TTGATCTAATAACCTCAAGACTACATAAGA--TTCGTTCTGAGTGAAACGATATATCCATAAACCCGTCGATCTGTACGCACGAGTATCATCTGACATCCTCTTGGATAAATGAGCACCCGGCGTTAACAGGTCTTAAATAACGCCACATATTAGGCATGTCGAATTGGGGCAACAAGTTATCCATGTATGATCGTAAATCCAGTTATAATGCTAAATAAAAAACACCTTCACTGTAATAAGCCTGCCTTACGGTCATGGGCTTACCTACAGGGATGTTCTGTTCA--TTAAAAATTCCTTAATTTGGATATATATCTATACATATCATGTGCGGAATGTCGGAAGACTACAGGCTCAACAAAAGTATATGGTTAATGGGATATCTTTATATTCGAGTTTTTTTATTCTTTTTGGGTTTTCAAGAGCTGCCCACTCGAATGGAGTCTGCCCCTCGGATATACTTAACCATCAATCCCTTTAAACATATCTTTTGTCGGTTACGCACTGGCCACAAGTTAT----GAGGAACGACCAACTTCAGGCTACTTACGTATTTGGATTATTTCATTTCAGGTAGATAATGATCCCTTGCATAGGATCGTTAGGTCACTTTTATGCACGGTTCAGGTTCAGTATATGGACATTTCTTCGTTGTTTGCGCATTATTACATTTCAACACACAATGGTAGAGCGCGCTGTATGCATTGTGAGTGTTGTTTTCTTAATTCATTATGTGTTTGTGAATGGGTCTTCTAGCTTGCTTTATCACTTTACGGATTTCTGCATCATAACGCAAATGATTGCTTAAAGGTATCAATTGCGGAGTAACAGGTGATCTCGAATTCAGCAAGGGCTCTTTTTTATTGTTTATTCAATGGCGGGATTATAGGTCTAAATTAT--ACACTGTAGTGGGTGTATCTTTGTATATGGACGCTTCTGGTAGTTTCCTGGGCAATTCCTTGGGGCACGTATACAACTCTTCGCGTCCTATTGGAAGATTGTAATGTTTGGGGGTTTCATGAAAGCACTTACCGTTTTACTTCATTCTTTCCTTCTATTCGCCTGTTTATCACTCTTTCTCACAACGGTCAAAACCTTGGCTAAAGAATAGAAAATCCTTCACCTATTTCATAAGATCTTAGACACATGTTTTTCTTTATATTTAGATTTGACCCGCTCTGGGACCGAAATTATCCGCAACCTTGTACCCATCAATCACCGATGTATTTTTTGCTAGCATCTCGTCATCCAAAATTGGGGGTATGCATGTGCTCATTTATATATATTTCCTTACAAAACCAAATCATCATACTTTACCATAAAAAGTTTTTGATCTTCGTAATTATCTTTACTGATGGGCCGCCGCGAGACCTTATTTACGGCAATTTACATACTATTTTTATTATGTAAATTCTTTTTTTTATTTATGTTTTTGCGTGCTTGTACTTTTGACGAGGTTTGGTAATCATTCGAAGGCCAAAAGTGGTTATGGCTTTCACCTTTCGAGGTTAATTTTTTAAGACAACTGCC--GAGTTCAATTGTATTATTGTTCCCATTTATCTTTCTTCATTTATTGGTGTTTCCTTTTGTAAGTGTAG----TTAATTGGGTGTTTTTTTTGTGTCAGTTGGGTTAGGATATTTTCGGTGTCTCAATCAATAGCATTTGGAGATCGTCGTGCCAACATTCTAGAGTAGTTTCTTATTTATAGTTCTATTTGTCAGGTTAGCTATTATTTGATTTCACAAAGTTGTTATTTTTTCAT--CCTTTTTTTGTGTTAGTCTGTTGCGAACAACGTCCCTTGATTGCGAGGGAAGGACTGTTCGGTTAAGTGATAAGAGGGGGTTGCTTATTTTTTCGATAATAAATATTTATAGATCTAGGTATTTTTATGGTGGATTGGAGTTATTTTATTAAGTGTTTTTGTTTATTTTATGGTCGGGACTTCCCGTTCGTAGAAATTATTACTACTGAGGGTTTTCCGTTCTTAATAATTTTTATATTCCTTTATCCGTTTTTAT----------------GTATTGACTTATGCAAAATCAAATGACGGAAACTTCCCTAGATGGGTTGACCTAAATCGCCGATCCTTTCATCATTTTATATGCATTTTTTTATTTGAGTGAATGTATATCTCCGCTCATACTTAATAGGATCTAGAATGATATAC--TCTCTTTTATATATCTTTCTGGTTCACAGATGTAAAGGATCTGATGACTTCTTTACCTTTAGTTGAGTTTGTATTCTTTTATTTTTTAATTAGTTTTTTAGGGAGGTTTATTATATGTTTTTACTTTCGTTGGAGGTGTTGGCTTCATTTGTTCTTATCGTGCAGGAAGATATCTGACTTGTATACATCTTTGATTAATCTTATTTATTCAGGTATATATTCTCATGTATTTTAAGTCATTAATGAGGGAAAAACGGTCGTTTTTATTGTTTATTTATATTCATATATATATATAGCCAATTGTAGATTTTTGGTGGAGGTTGGTTGTTCTAGGTTGTATTATTCAAATAAATCTAGCGCGGATTTACATTTATAACGGTGGGAGCGCATTTATTGGTGGTGATATAATTGGAG--TGCATAATTATATCTTATTG------GAGATGGTATTAATTGTTTTTTATCTGCGCTTACAAAGGCCAATCCTTTCTCTGCTCCGCGCATGCGCCCACCCGTTCGCCTGTCATCTCACTTGTACGCGGGTTATTTTATCGTTAGGG--GCTTGTAAATGATGTCTTTT--GTTTTT--GGATATACATTTATTCGGTTGGGCAGTTGATTGACTAAAGATATGCTTTCACTTAGCATTGGTTATATTCGTTTTTATGGTAATGATTTCTTTTCATTTTACCAGCTTTTAAGCCTTTATTCAAATTCTTTGGGCTCCTTTTTCGGTTTATCAAGATT--ATCTGA--ATATGTTTGATATAAATTACGTTTTTATTTTAATTCGTGGTTACTCTGTAACATCGGTTTTATATGATTTGGAAAA----------AAATTAGTTGCA------TTTAAAGAAATAGTATATTGTCATATATTTGTGTATTCATATGGGGTCATTTTCTG------------TTATTTAGTT----CCGATTTTGTATACTTACTTAATTATGCCTTTTTATATTTGGATATTTATTTACAGAT--TTTTCCTTCTAA--CACCTTAGATGGCTATATATTTCAACCTATACGCATATACGGCAATATAAATTTTGTTTCTAATTTATTTACATACAGGGATTTGTCTTTATTAATGCAGATGCTC--AA--GAAATTAAAGATAAATTCGTCT----------------TCCTATTTATTTCTCCTTATATATATAATAAA------------------GATTTTATATAATTTTAAAAAATAATACTTTATTAATAGA------------CATCATATGTACAGTTTTAAAACAGCTTATATACATATCTGTATTATTTTTATCTTATAGGTTCTGATGTAAGTTTGAGG

Bothriometopus_macrocnemis CTTCGTTTGACCGGTCAGAATTTCTT--ATCCTT----AATGCTATTCTT--------------ATGGTTATTTAT--TGCCATTGACGTAATCGGCATTACTTATAATTTTTCATGGTGAAAGCACTGAGACATTAATTTAAA------TTCCTTATTTAGTTTCATTTATACATTTTGATAAGAATGGATGTCCTATTTTACCCACAGCATTAGTTAACTTGCTGCCTCCTTTGCTACGGATATTGAT--TTAAAAAATGAAGCTTTTTCCATTGTCCGAGGAGCCATGGTTGCCCCTTTGTATATGACTGTAGTTTTATCGCCATTCTTAGATCGTTATTCAAATATGCGGCAATATTTAGTTGCTCAGGCGTTCTCATTCATAGTCTCTTTTATGAATGTGTTTGGCTATAGTTGACTTGGTGGATGTCAGCTAATTTTCTGTTTTGTATTATGTCTTTTCCATTCTTACTTTTATTTTTATTCATATATATATATGTGT--CAAATTTTACCCGTTTAGGAAGGAAGGTGGAGGCGC----TAATATGAAGCATTGTTATTTTTCACAACAAAGAATGGATTTTAATATTTGGGTTGTCGGTTATGGTTGGTTAGATATATCGATCATTGGTCCCTGGGCTCTTTGAGGCAATTTAAGTGTGTACATCAGCTTCTATATTTTTATGTATCCATATATGGGGTTGCAATGATGTCCATATTTGGGCCCGAATGCTTCCCGATAAAAATAGTTTGCTCTCCCCTCTTATTTCTTTATGCACTGATGATCGGGTGGTCGGTGACTTTACCCCCTTCTCATTTGGAGCCGGTTGAGTGGTATTATTTTCCTCATTGCGGGTAGTCATATGGGCATAATTATTCACATCTAAATTGTCTATCTTGATTACAAATCCCTTTTGTGGCGTCTATACGCATCTTTCTCTTCCTCCGTTTGCGGGCATACATCTCTTTGACGAGTTAAACTCTTTTTCCCGATTGGGGGACCATTTTACACATTTTTGTTTTGGCACCGAGTTAATTTATATCCGGTTGGTTATTCCAATATAAGATGAGGGAACCTCGCTTGGAGTTGGATATTAGCATCTACATGGTTTTGGTTTTGTTGGCCACAATTTACGTGGATGAATGAAGCGGCTATTACAGGTACATGTATGCGTCCACGGATAAGTTTAGTGTTGGACATTTGGAGAAATAATGTCCTTCTCTTTGAGGTGGTTATTTTTTTACTTGGGGCTACGGGTGTTTGCAATCTCATGAACTTATCAGAACTATAGTGTGCCATTCGTAGTTTTCATGGGCGTTTGCATTTGCAGTTTTCATGTTCCTTTTACGGTTACCTAACAAACTATAAATCATTTTGTACTTATGGGTAATTACTTTTCCCACATTTTGGCTATGGATCCCGCGTAGCGATACCGATTTAACCCTGAATCATTCTCATGGAGTGATTCGTGTGGTTTCATTTATTAGCATTAGAAGTTATTCCACGAAGCATATCATCATAAATAGTCGTGATGTTTGGGTGCCCCTCGCCATCTTGATCATTCTTATTCAAATTTTTATGACCATTCTTTCGGGAATGTCATCGTCAGACAGTATATATATACCTATGTATTCATAGTAGTTTTTATATTTTGCGTCAGCAATATGGGACGTTTTCATCAGGAATTTGAACGTTGACGTTTCCGTTTATTTGTTTGCGCATCCTCCTCAAGTTTAATTTGAGAGAAAAACCATATTCGTAAATTTGGAACATGTATGACTAGATTAAACGGTACAATAA------TAAATCTAATATCCTCTCGATTGGGAAGGA--CTCGAATTGAGTGAAAAATTGTCTCCGTGGGTGAACCGGCATATACTCAGGAGTATCATCTGGCATCCCCTTGGGTAAATGAGCGTCCGGCGATAACAACGTTTTCATTCATTCGGCTTTTAGGCATGTCGAATTGGGTCCTCATCTTATCCATTGGTGAGCATCCCTAAAATGTTAATGTTGATTACCATTCACCTTCAATGTCATTAGCCTGCCTTATTGTCTTAGCTTTAGATATATTTATTAGATTTTAA--TCTCAGTTAAAGTTATTTGCGTCTATCATTTTGTTTTTGATGTGCGGAGTTGCGGAAGACTTCAGGTGCATCTCAAGTGTCGGGTTAAATGGTTATATTTATTGTCGAGTTTTTTTTTTCTTTTTTGGTATTTTCTTCCTAACCGAGTGTTTGGGGATTGCCCCAAGGTTATGTGTGATTTTTCGCCCACCTAATCATCTCTCTTCAGGGGTAGATACTGGCCACAAGATCT----GATCAATTTCGAGCAAATGGTTGTTAACGTTTTTGGATATTTTCATATCATTATGATATAGATGTCTTACATGCGAAGCCTTGGTCATTTTTTTGCACGGTTCAGGATCAGTTTGTGGACATTTATATATTCTTGTCGCTTTAAAACATTTCAAAACACAGTGGTTGAATAGTGTGTATGCATTGTGAGTGTTGTTTTTTTTGTCGGTTATGATTAATTGAATTGGTCCTTTGGATAACTCTCTCAATGTAGGGATTTTTGCATCATAGAGAACATACTCGCTTGAAGGTGTAGATATAAGAATAAAGGGTGTTATCGTTATCAGCAAGGGCTCTTTTTTGTCTCTTATTCAATGGCGGGTTTATAGGAGTAAATTAC--GGACTGATGTGGGTATATATTTATCTATGGACGCTTGTGGTAGTCTCCTGGGCAATTCTTTGGGGCACGTATACAACTGTTCGCGTCCTTATGGACGAATGTATTGTTTGGGGGTTTCGTGAAACCACCTGTCGTTTTTCATCATTGTTTCCTTGTATTTGCATGTATCTCACTTTTTTTCATCACGGAGTCAACCCTGGTTGCAAGAAGGAAAGTTATTCACCTTTTTCATAAGAATCTGGTTATATGTACTTTTTTCTTCACGTTTTTAACCGAAGTTATGACCGAAATTACCCGCAACCATTCACCCCACAATCACCGATGTATTCTTTGCTAACATCTCGTCATTCTCAATTGGGGGTATGCTTGTTTTCATCTATTTATTTTTCCCTCTAAATAAAGTCCAAGATTTTA--------AATTTTTTTGATCAGTTCAATTATCTTTACTGTTGGTCATCCGTGACACCTAGTCACTGGCAATGTTCATAGTATTTCGTTTCTATTGATCACTATTTTTGCGTGGGTCTTTTCGTGCTTTTTCCTTTGACGAAGTTTGCATATCAAACGGTGGCCGAAAGTGGATGTGGATCTCACCTTAGGAGCATAATTATTCAAACGATCCCTCCCAGAAAAGTGGGTTTTATTTTTCCCGCATATTTGTATTCTTATGTTGATACTTCCTCAATGGAATTATC----TTGAAAAGATTTTTGTATGCTGATGGTCAGGTTAGGCTGTATACGGTGTTTCAGTCAATATCTTATGGAGGTCGGCATGGATTCATTCTAGAATATCTGTTTGGCTGTTTATATTTCTCTGGACATAGGTAATAATATTCTTCAGACAATTGTTTTTTCCTTTG--TTGTTTATATCTTTATTCTTTTGCGAAGGGCGGCCCTTGACTTCGAGGGAAGGATTGTTCGGTATCGTGATAGGGGATTCTAACTTATTTCTTCGAAAAGTCATATTTAGTCGTATTTTCAGATTTTTGC----ATGGAGATTTACCTCCGGTTATTTCT------GTGTTGATCGGGACGTCCCGATCGTTGACATTATATATTGTGGTAAATCTCCATATTTTTATTTGGATATTTGTGTTCCTGTTCCTCTGT----------------TTATGCAATTTTGATCCAGAGAGA--TCAGTCGGGAGATTGATGGGATGATCATCACCACCAACCCATAAATCATTTTATATGGATTTTTCTATTTGAATGAGTGTGTATCTCCTTATATTT----TCTGAAGAAGGCATTTATAT--ATTCATATGCGCATTTATATGGATTGATGAATTTATGGTCTTTATGAAATATATTTCTATACCTGATCGTATATCTCTTTTTTAATAGTGTTCTAAGC------CCATATTCTTGTACTTTTGTTTATGTCTGAGGGTGTGGTTAGGTTTGTAGAGGTAAATTCGTTCCCTTGACTTCTCGAATACATATTTGACATTAGGTAGTTATTTATGTCTTCGTAGACTGGTCTATTAGCGTTGTAATGAGGGAAAAATTAAAATTATGTACTTTTATTTATATTCATATTTTTTGATAGACGAATTATGGTATGTGGTGGAGGCTGGATACTCTTTTTTATATTTTTCAAATGAAAGGTAGAGGGATGTACCTCTTCAACGATGGGAGTTTATGTACTCATTGCTGAGTTTTATTGA--AGAATATCTT--------------------------------ATTTATGCCTGGGCATACAAAGGCCATACCTATCGCTGCTCCGAGCATGCGCCCACCCGTTCGCTTGTCATCTCACTTGTACGCGGATTACTCTCTCGTTAAGA--ATTTAAAAGATTGTAGCTTT--ATCTTCGTGCAGATTCGCTTTTTCATTCAGAGTGGGGATTGATTAAAAATATGCCTTCACCTTCCATTAGATATATTTTTATTCTGAAGATACTTGGCATATCAATATAGCAGCTTTTAATCTCTTTTTCTGGCTCATGTGGGGCCTTTTGCGGTTTATCAAGAAT--ATATAT--TCCTTTTCTTTCCAAAACTGATTTTGATACATTTTTGTTTGGTCTGCTTATCGCCGATATTTCGCTCTTCAATT------------CCTTTATCTATC--------TCGACCGAGATTAATCACCCCTCTATGTACATCTAGATTCGCGGAGATATATTG----------CTACATTCTCTG----CCAGTTTCTTCACCAAGATCGGGTAGAATTTT--CTTTTTTCATATTTGTGGTTATGG--ATTTTGTTAAAT--AGCCATATGGATATTATTTTGTAGACACATTTACACATCTTTATGTAAGGTC--TTGTGGTTCTTTTTTTTCACATTCGGCTTTATCTTTTCATTTATATAGATAA------ATAATT--TTTCAAACCTGTTTAA--------------AGAAATTTTTATATATCTTCTCTTACATATAG----------------ATTTTCTCCCCCAAGATGGGACTATTTGTTGAGACAATCTTC------------AATCGGTTAT----------------ATTGATTAGTTTTTATTTTTTTCTTTTCCTCCATGTGACAATCTAAGAATTCTC

Campanulotes_bidentatus TAGGTTTG--ATGTTTTTGTTGTCTTTTTAAGATATTGGTTGATATACGTAAATGTGAGAATTGGCGGTTTTTTTGAATGCGGTTGGTTCGTTCGGGTGATTTTGAAAGAATGTAATTTTTTGACTTTAACATCATGGGGTTAT------AAATGGCTGTTTTAAGGTTTTTTT--TTTTTCAATTCTGGTTGTCCTAGTTTACCTTCTCCATTTGGTAATTAGGTTCTTGTTTTGCTGGGGGTGTTATC--ATAAAATCATGATCTTCTTCCAATGTCCTTGGTCCCGTTTCTCTCCTTTTGTTTATGAACATAGACTTATCGCCTTACTTGCATCGTTATGCAAGTATGCGGCATTATATAGTTGTGGGGTTAGTCAGCTGGTTGTTCAT--TTCCATTTATGATTGGTTTTTTTTGAATTGGTGCGTGTCAGCTAGTTTAGAGTTATGTATTATTGCATATCCATTATGTTTTGTTGTTATGTAATTTTATTATTTTCTAG--TTCATATTAATTCTGTTTTCCATCGAAAGTTGTT------AAAGGTAAGTAGTTATAGTTTTTCTCAACAAAGAATGGATATTATTATTTGGATTGAGGGTTTTGGTAGGATAGGTGTATCGACGATTGGGAAGGGTCTTATAGGATCCAATTTAAGTTTGTACGCCAGCTTTTATATTTTTATGTATCCATATATGGGGTTGCAATGTTGTCCGTATGTGGGCGTGAATATTTCCCGATAAAAATAGTTTGCTCTCCCCTCTTGTTTTTTTATAGAGGTATGAAAGGGTGGACGGTGACGTTACCCCCTTCAGTTGTGGCACCGGAGGCGTGATAGCATTTTCCTCATTGCGGGTAGTCATATGGGCATAATTATTGACATTTAAATTGAGTTCCAAAGTGGATTGTCCTTTTTGTGTCGTTTATACGCTTCTTTCTCTTCCTCCGTTTGCGGGCATACATCTTTTTGACGAAATAAACTCTTTTGACCTCGGGGGGGACCGTTTTACACATTTTTGTTTTGGCACCGAGTTAATTTATCTCCGGTTGGTTATTCCAATTTAGGAAAAGGGAAATGAGTTTGGTCTTGGATATTAGCATGTGCATGGGTTTGGTTATGTTGGCCACAATTTACGTGGTTGAGTGAAGCGGCTATTACTCGCACATGTATGCGTCCACGGGTAAGTTTAGTGATGCACTTTTGGAGCGGTAATGAGCCTCGATTTGGGATGGTTATTTTTTTACGTGGGGTTACGGGTGTCTGCAATCTCTTGAATATCTCAGAGCTATAGTGTGCCATTCATAGTCTTCATGGGCGTTTGCGTTTGGGGTTATCATGTTCCGTATTTGGGTAAATGATCGTTATTAAGTCATTTTTGACTTGTGGGTAACTACTTTTCCCACATTCTGGTTATGGATCCCGCGTATCGATACCGAATTTTATCTGAATTATTCTCATGGTCCAATACTTGTGGGTTCTTTTTTTTTGTTATGAGGTTTTAGAACGAGGTTTTTTCTA----TCAGTCTTGATGATATGGTACCCCAATCCATCTTGAATGGGTCAATGTGATTTTTTCAGAAGTGGGCCTTATAGCAATTCGGTTCAGACAGTATGTGTGTTTATATTTACGTGTGTTAATAAATGTGTTTTTTTCCTGTA------AGCGTTATAAAGAGGAGGTTGAACTTTGACATCTCCTGATGTTTGCTCTTGCGCCCTCTTATACTTTATTTCGAGATTAGAACCGTGTACTTAAGTATGGCACATGTATGTCTAGATAGAGATTTCTCTCTC------TTGATCTAATATCCACTGGATTTTAAGGGA--TTCGCTCTGAGTGAAAAGGTAAGTCCTTAAAGGAAGCGGTTTGTACTCTCGAGTATCATCTGACGTCCTGTTGGGTAAGTGAGCATCCGGCGTTAACATTAGTTTACCTCCGGTGGTTGCTAGGCATGTCGAATTGGGTCATCATCTTATCCATTGTTGAGTGTCCCAGAGATTTTCGTGTTTGAATCGGTTCACCTTCAGTGTGATTAGCCTGCCTTGTATTCTTTCGTTTTCTTGATTAATTTACATTTTAATTTCGGAGGTGTTGATATGAAGTTTTTCTCATTTGTAGGCTTTGTGCGGAGTATCGGAAGACTTCAGGCACATCGAGAGTCAAAGGTTGTCTGGGTCTTTTTATTGTCGAGTATTTTTTTTCTTTTTTGGTTCTTTTCGCTTTGCCGAATGAATGGGAAGTGCCCCTTGGATGACCCTAATTATATGTCCTTATAAACTTATCTTTTCAGGGGTTCATACTGTCCACATCATAT----GAGGGATGAAAATCCTTTGGATGTATACGTTTCTGGTTGTTTTCTTCTCATAGAGATATTTCTGTCTTACATGCGAAGGTTAGGTCTTTTTTCTATACGGTTCAGGATCAGTATGTGGGTTTTTATATGTAGTTTTCGACTTGTGGCATTTCAAAGCACATTGGTTGAGCGCGCTGTATGCATTGTGAGTGTTGTTTTTTTTGTACGTTATGTGTATTTGAATTGGTCCTCTGGGTTGTTATATCATTGTTCGGATTTCTTCTTCATATCCCACATGAGAGCTTTCAGGTGTATATGTGAGAGTCCTTGGTGATTTCGAGATCAGCAAGGGCTCTTTTTTTTTGGTTATTCAATGGCGGGTTTATTAGAATAAATTAA--CCGTTGATAAGGGTTTATTTTTTTTTATGGACGCTTATGGTAGTCCCCTGGGCAATTTTTGGGGGCACGTATACAGCTTTTCGCATCCTAATGGGGTTTTGTAATGGTTGGGGGTTTTGTAAGGCCACCTCACGTTTTTCCTCATACTCTCCTTATTTTCGTTTGTTTTTCAGTTTTTTTCAAGAAAGGGGTAACCATGGGGAGTTAATCAAAAGGTTTTGTCCTATTTTTTGTGATTGGGGATTTTTTTTTTTTTTTTTATTTGTTTGTTTCAGAGTTTATGACCGAAATTATCCGCAACCATTCACCCCCCAATCACCGATGTATTTTTTGCTAACATCTCGTCGTCCTCAATTGGGGGTGTGCTTGTTTTCATTTTTCTGTTTCTCCTTATTCTTTCAATCTTAAATCGACGGTGGATAAATTTTGTGTGTTTTTGTGTTTTTTTTTACTGATGGTCATCCGTGATACCTAGATTGTGGAAGTTTTCGTTTTATTTTGTTTATTTATATGAGTGTGAGTTTTTATGTTTTTAGGTGCTTTTTCTTTAGACGAATTATGGTTGTCAGGCGAAGGCCAAAAGTGGGTGGGGGTCTCACCTTGCGAGCATAATTATAGAAAAGATAGCCC--AGAAGTATAATTATTAGCGTTCCCATATTCTTTTATTCTTGTTTTGATATTACCGTATTGAATTTTTC----TTAATTAGATGTTTCTTTGTTTTTAGGTTCGTTAGGTTATCTTCGGTGTTAGTCTCAATAGCAAATGGTGGCCGGCTTGCCATCATTCTAGAGTGGTTACTTAGATATTTTTTGTTTTTTTCTGATTCTTTCGAATTTGAGTCAGATTTTTTTTTTCCTTTCTT----TTTTGTTTATTGAGATTTTTGCGAACAACGCCCCTTGATTGCGAGGGAAGGATTAAAAGGTTTGGTAAAAGGGAATAGAACCTTATTTTTGGGAAATTTCGTTTTTGTAGATTTTTAAGTTTTTTTAGGGTCTTTTTTTTTTTTTGGGGGTTTTTTTTTTGTCTCTTACTGATTGGATATAATGCGCTTATTGGTTAATATGCTTAAATGTCTTCATTTTTTTTTTTGTTGCAGGTGGATGTATATTTTTTTAG----------------TTATAGAGATTTTGGTAAGAAAAC--GGATAATTGAATTAGATGGGATGACCATCAGAGAAAGCCCTTTGATCATTTTTTGTGGGTTTTTCTTTTTGAGTGATTATGTTGATCCATGTTGAT----AGGTTAGAAAGTTGGGTTTT--TGTTGTTTTTTTATATTTGTGGTTGTTTGAATGTATGGACTTGATGAATTGTATATTTATTCTTGAATATACTTACACTTTTATATTTTCAAGTTGAT--TTTAAAATCATTTTACTCTTTTGTTTTCGTATGAGGGTTTGGGTTCATTTGTATTTTTTCAATCAAATTTTGGAAGAATTGAGTTTTTGTGTGATCTTTCATATTTTTTTACGTGGATGTAGAGTTGTTTCTTATCAATATAATATGGAGTTTTAAAAAATTATTTGTATATATTTATCTTCATTTGTTTAGTTAGGGGATTTTTGGTATATGGTGGAGGTTGGTTTCTCATTGTTATTTTTTTCAAATGAAAGTTAAAGTCATGTACTTATTCAACGATGGGATTTTATATTCTTTTTGTTTCATTTTTAAGG--AGTTTTTTGAAATAGTTCTC--------------TCTTTTGGTTTTTTTGTTGGGCTTAGAAAGGCCAGTCCTTTCGCTGCTCCTTGCATGCGCCCACCCGTTCTCTTGTCATCTCACTTGTACGCGGGTTTTTTTATCGTTAAAG----TTCTTGGAAATTTTAGGT--ATTTTTGTAGTTATACATTTTTGCGGATAGTCGTGGGATAGATTAAAAGTATGCTTTCACCTTCCAATGGTTATATATTTGTGGATGAAGTTATTCGCAAATCATTGTATCAGCTTTTAATCCTTTCTTCACTTAGATATGGGCCCTTTTTCGGTTTTTCAAGAAT--CTTTGG--TGGTTAGAAGACGTAGATGTAGGTTTTTTTTTTTTCGTTCTTACTGTCTATCATCGATTTTTTATTTTTTCCTGTT------------TCTTTCTTATATCCAGAGGATACCTCAAATTTTTTTATAGGTGTTCTCTTTTGCGGGTATGGTTGTCTATGG------------AAGAGAAGGA----ATTCTTACGTTTTCTCTTCGAAGTGGTTTTGTAGATGCATGGTTTCTTTTTTATAGTT--TTTTTGTGTTTCTTTCAGAGTTTCTTTTATGTTTGTTTTCGTATAGACTTAGTTTCTTTTTTGATTAAGTCCTTTTATGTGTTTACCTAGGGGGTTTTGTTTGTTCTTGTATATTTTTCC--GAGATAAATTGGGTACTAGCAAAAATTT--------------TATCTGTTTCATGTATTTGGATTCATTCTTTT------------------TGAGGGGATTTTATGGGAGATTGTGTGGGGTTAAGTTGGG----------------------------------AGTTATATGTTATTTTTTATTTTTCTTTTTTTTTTTGTGTAAAGGTGTAACTAGACGG

Haematomyzus_elephantis ATTCAGTTGACCTGTCAGGTACGGTT--ATAATTTCGAAATGATTTGCTC--------------TCGGATATCTTCGGAGGTTTTGCTTGCCAGGGGTCATTTTATGGAACTGCAAGCTTACAGCTACCACAGAGTTTCATTAA------AGGTGCTTGCTGTGGCATTTATAATTTGGCATAAATGTGGCTTTCCTATCTTCCCCTCAGCATTACTTAATTGCTTAGCTCCTTTGGCGGGGTTATTATC--ATAGACTCTTAAAGTTTCTCCATTCTCCGAGGAGCCTTCCCTTCCCTTCTGTATGTGAATATTCAGCTATCGCCTTTCTTAGGTCGCTATTCAAATATGCGGCAATATTTACCTATGGCAGCGCGCTCTCACTTGTGTATCC--GCACTTACCAGCGCTTATGGTTGACTGGGTAGGTGTCAGCTTGTTTATAACTCTTACTTATGCTATTTCCAGTGTGTTTTTTATATTTTCCTATGTACTTGCGTATTC--ATGTTTTTATGTAGGAGTCCGTCTGAAATCCGAATC----TCGCCACTAAGTAGTGCTTTTTTCACAACAAAGAATGGATTTTATTCTTTGGGCTGTCGGGTGTGGTTACTTAGATTTATCGATGATTGGGAGCGGAATTATTCGAAGCAATTAAAGTATGTACTCCAGCTTCTATATTTTTATATATCCATATATGGGGTTGCAATGCTGTCCGTATCTGGGCCCGAATGCTTCCCGCTAAAAATAGTTTGCTCTCCCCTCTTTTATCTCTTTAGGGTTGTGGGAGGGTGGTCGGTGACGTTACCCCTTTCAGAGGTGGCACCAGGTAGGTGAATTCATTTTCTTCACTGCGGGTAGTCATTTGGGCATAATTATACACATGTAAATTGAGTTCCGCAATTGATTATCCTTTTTGTGTCGTTTATACGCGTCTTTCTCTTCCTCCGTCTGCGGGCATACATCTTTCTGACGAAGCAATGTCTTTTGACCTCGGGGGGGACCATTTTACACATTTTTGTTTTGGCACCGAGTTAATTTATCTCCGGTTGGCTATTCCAATATGCGAGAAGGGAAAAGAGTTTGGAGCTGGATATTAGCATATTCATGGGTTTGGTTGTGTTGGCCACAATTTACGTGGATGAATGAAGCGGCTATTACAGGCACATGTATGCATCCACGGGTAAGTTTAGTGCTTCACTTTAGGTCACCCTTTATCCCGCAACTTGGCATGGTTGTTTTTTTACGTGGGGTTACGGGTGTTTGCAATCAGGTGAGTGTTTCAGAACTATAGTGTGCCATTCATAGTCTTCATGGGCGTTTGCGTTTGCGCTTATCATGTTCCACGTACGGATTCCTTCAGTCCTCTAAGCCATTCTACACTTATGGGTAAGTACTTTTCCCACATTTTGGCTATGGATCCCGCGTAAGGATACCGAATTTTTGCTGAAGTATTCTCGCGGAGGTATACTTAGAGGTGTTTTTTTTTTTTTTGGAGGTTTCTCTGCGAACTACTTAAAGGCTGCCTCTCTTGATGTTATGGGGCCCCTCAGCATTTAGACAGTCCGTTTGTTAATTCTTTCGAAGCTTCCCAGATAATTATAGAAACCAGATTATTTATGTGTATATATATTCTTGTCTTATTTCTATTTTCTTTGGTCGGTA------AGCGAGGTGTGGAGGAGGCTGAGTTTTGGCTTGTCCATTGCTTTGCAGTTGCGTCCTCCTCATGTTTATTAGGAGAAATTAACCTTATTCATAAGCGTGGCACATGTATGTCTAGATAAGGATTGAAGGTTC------TTGATCTAATAT----AGGATTAAGTTGGA--GTCGCTTTAGGTGACAAGGTATCTCCGTCAGAAGATCGGCATGTTCTCTCGAGTATCATCTGGCCTCCGCTTGGGTAAGTGAGCGTCCGGCGTTAACATCCTGTCGAGGAAAGTGGGAGTTTGGCATGTCGAATTGGGAGTTCAAGTTATCCATTGCTAGTTGTCCAACCCATTTTAATGGTAAAATTGGTTCAAGTTCAATGTGACAAGCCTGCCATATTTAGGTGGGTATACTCATTCAAACTTATTTTATTAGGATGAATTATGGGCGTACAGTGATGCACTGACTGGCGCTTTGTGCGGAGTATCGGATCTTTTCAGGTTCAAGCATCGTATGCGGCTAGATGGTTATTTTTATGCTCGAGTATTTTTATTCTTTTTGAGTTTTTAGTTCTTAACCGAATGATGGGAGGTTGCCCCGCGGGTCAAGTTAGGCTTAAGTCCCTTTAAAGATCTTTATAGAGGGGTTCATACTGGCCACAGCCTGT----ATAGAAATAAGAACGCGTGGTTGGATACTTATTTGGCTACTTTCGTGTCATCTTGATATTCAACAGTTTCATGCGAAGGTTAGGTCGTTTTTCTACACGGTTCAGGATCAGTTTGTGGAGATTTATACGTAGTTTGCGACATTTGGCATTTCTGAACACAGTGGTTGATTTCGCTGTATGCATTGTGAGTGTTGCTTTTTTTATTCATTATGTGTAATTGAATTGGTCCTTTGGCTTGCTAGGTCACTGTACGGTTTTTTGCATCATAAACCTCGTATAAGCTTGAAGGTGTAAATATAAGAGTAATGGGTGGAGTCGCTCTCAGCAAGGGCTCCTTTTTGTTGTTTTACCAATGGCGGGTTTATAAAAGTATCACAACCCTACTGAGGTGGGTATATTTCTATGTATGCACGCTTTTGGTAGTCTCCTGGGCAATTCTATGGGGCACGTATACAATTCTTCGCATCCTATTGGGAGACTGTTTTGCTTGGGGGTTAGGTGGAGCCACTTTCCGTTTTACATCATTATCTCCTTATGTTTAGTTGCATACCAATGCGCCTCAATAAGGAGAGAACCTTGGTTTCAGGATAGAAAGTCCTTCACCTATATTGTAAGAATGTGGTTGTTTGTAGTTTTCTTTATGTGTTTACTTCCCATTTTATGACCGAAATTTCGCGCGACCCTAAACCCCCCAATCACCGATGTATTCTTTGCTAGCATTTCGTCATCCAGAATTGGGGGTGTGGTTCTGCAGATTTATTTCTGTCTCCGTATGA--AGAATTTCAGAGCGTTGACCATTCAAGTATTTTGTTTTGTAGACTTGTACCTACTGGTGGATATCCGTGACACCTTGATTATGGAAGTTTTCGTTTTATTTTGTTTATATCAATCAATGTATTTGTGTTCGTTTGTAGGTGCTTTTTCCTTAGACGAAGTTTAGTTATCAATCGAAGGCCAAAAGTGGGTCTGGCTTTCACCTTAGGAGCATAATTGTAGAATCATCACCCCGTAAGTGAAGAGTTTTTTAAATTCCCATATTTATACATGTGTATGTTGAGGTATCC----ATTAGG----TATCTCGTTGAGGGTTTTTTCTTTCTTTAGTTACTCTAGGCCATTTGGGGTGATTCAATCTGTTTCGTATGGAGGTCGAGGTATATATGCTCTAGAATACTTTCTTTCATTTAGCTTTTTATGGAATCTTTCTTGAATAGTTAAATATGACTCCCTACATTTTCGTGCCCTGCTAGGTAGTTATATTCCTTTGCGAAGGGCGAGCCTTGACTTCGAGGGAAGGACTGTGCGGTAACGTGATAGGGGATGATAACTTATTTTTGGGAAAATTCACCTTTATTGGTATGCAGATGTATTTAA--TCCTAAATGTAGGTTTAGACATTCCTGT--------ATTTATCGGGGTGTCCCGGTCGTAGACAATATTTTTTGTGGTATATTTCCATTTATAGAGGTAGTTATGTATATTTACTGTTATATAT----------------GTGTAGGGTTTTTCGGTCGATTAA--CAAGTCAAGACCTTGATGGGATGAGTTTATAGTCCGACCCTAGCCTTATCTATTGTTTATTTTTGTATTTGAATGATTATGTTCATCCCTGTTTAC--AATTTTACTGGAATTGAGATGT--TGACATTATCCTTTATCTATGGCTACGTGATTTGCTGGAGTTACTCTGCCGTAGTGTTGTGGTTGAATATTCGTATAGTTTTCTATAATCTCGGATTTTTATAGCAGCACTCTTATTTATATACTTGTGTTGGAGGGTTTGGTTAGTTATTCCCTTATAAAGACTCTCTTATGCATGAGTTCGTTTATAAGTGATACTAGTTGGTTTTTTATGTAGGTGTTCTTACGTCTATTATCAATTTAATGCGGTCCCGATTAAAATTATTTATTTGTAGTTATGCTCATATACCTGCTTAGGGTCTTTTTGGCTTATGGTGGAGGCTGGCTAGTCTTTGTTGTATTTTACAAATGAAAGTTAAAGGCCTACACTTATATAACGGTGGGAGCTTCTCTTTAGTTTGAGATTTTCATGGTC--AAATTCATTTTCGCAGGCCC--------TTGATAGTGTGCGGGTAGGGTTATTGGCTGTCAAAGGCCAGTCCTTTCGCTGCTCCTTGCATGCGCCCACCCGTTCTCCTGTCATCTCACTTGTACGCGGATTAATATATCGTTAATC--GATTTTTATCAATACAATTTGTCTTCTTCTTCGGATACATCTCTGCGGGTAGTCCTGTGATTGATTAAAAGTATGCCTTCACTTCTCAATGGGTATATAATCGTGCCTCAATTTGACTGCTTTTCAATATGCCAGCATTTAATCTTTTTTATATTCAGATGTGGTTCCTTATAGGGTATATCAAGATTATATCTTCTCTTAGGTGGAGTTATAATTTGAGATATACTTAGTCGCATTTTTTCTCGCTATCTTCGCTCTCTTTTTTCCTAACGAGAATCAGCCTTCCATTTACCTAA----AAGAGTTCTCATGAATGTAATTAGCTTATTGGATATCTAGGTTCTTGGAGGTTTGACTCACAGATTACAATGGC----------ATCTCCTCCTGTGAGTGCCTAACTTTGTTGTTGCCTTTTTGGATTTTTTGTTACCAAT--TTTTTGTTAGTC--AGATGTACGATTATTCCTATAGTTACATATGTTCTTTGAGTTATACTGAGTCGATTGCCCCTATTTACTAGCACTTGGGGCTACATTTACACTTATATATTGAGCCCGTG--AGAAGTGGCGCCTTCATCCTAGAA--------------GTGTGCTGTTGTGTGTGTCTTTCTTGCTACGA--AGATATCTGATTAAACGGGTACCTCCTGCTTGGTAAACAGTGATGGGTCAGATGAGGCGGGTTCAACTCATGAGTGATGATCCATTGTTGCCAAGTTGATATGGTTATCTATATCTCTTTAGCTTTATGTGATCTCACTAGGAAGGGG

Psococerastis_albimaculata TTTCGTTTGACCTCACACATTTAA----TTTCAT----AATGTTAGACTT--------------CTATTTATATTTCCCTATTTTGTTGTCCACCGTTAATTGTTGATAAATATTTACTTCAAAGATTAAACTTATGGGAAAAATTAAACGGAAACATATTTATTCCTTTTCTTATCTTTAAAAGTTTGGCTCTCCTAATTTACAGACAGCAATTCATACTTGCCTTCTTCCTTTGATGCTTATCTTTGG--TGATAATTAGCACAATTTAGCACTATCCCAGGACCCGCATCTATCCTTATGTTGATGAATATAGAAATATCGCCGGACCTGCATCGCTTCGCAAATATGCGGCACTTTATACCTCTGGAAACGGCCAGTTTCCTATATTTAAATCTATTTACCAATCTTTTTACTTGAACGCGTGCTTATCATCTAGTTTGCATTTTCACCTTATCACATAACCATTGTGTTTCTTTTATATTTGTACATCTCTTCTCAATC--TTAATATT----TATCAAAAGCCTGAATAATCTTAA----AAAGAAATAAAAAAATAATTTTTCACAACAAAGAATGGACTTTATTATTTGGATTGGCGGATGTGGTCAGCTAGATTTATCGTTGATTAGCACCGGTTTTTTGAGAGACAACTAAAGTATGTACGCCAGCTTATATATTTTTATATATCCATATATGGGGTTGGAATGTTATCCCTATCTGGGCCCGAATGCTTCCCGATAAAAATAGTTTGTTTTCCCCTCTTACCTCTATTCAGAGTTGTAAACGGGCGGACGGTGACGTTACCCCTTGCAGACTTGGCACCGGGCTCGTGACTGCATTTTCCTCACTGCGGGTAGTCATCTGGGCGTAATTATACACATATAAATCGTCCAGGTTTCTTGACGATCCCTTTGTTGTCGTTTATACGCGTTTTTCTTTTCTTCCGTTTGCGGGCATACATCTCTACGACGAACTAAACTCTTTTGACCGCGGGGGGGACCATCTTACACATTTTTGTTTTGGCACCGAGTTAATTTATCTCCGGTTGGCTATTCCAGTATAGCAGAAGGGAAAAGAACTTGGGTCTGGATATTAGCATATGCATGGCTTTGGTTGTGTTGGCCACAATTTACGTGGATAAGTGAACCGGCTATTACTCGCACATATATGCATCCACGGATAAATTTAGTGCTACACCTTAGGGCAAATACTTACCCTCATCTTGAGTTGGTTGTTTTTTTACATGGGGTTACGGGTATCTGCAATCTCATGAATGTTTCAGAACTATAGTGTGCCATTCATAGTCTTCATGGGCGTTTGCATATGCGGTTATCATGTACCTTTTACGGATGTTTAAGAAACTTTAAATCATTTTATATTTATGGGTAAATACTTTTCCCACATTCTGGTTAGGGATCCCGCGTAAGGATACCGAGTTAACTCTGAAGTGTTCTCATGGAGATATTCTTATGGATATTTTTATTAATATTGGAAGTTAT--ATCGTTCCTCTTTAAATATCAATCTCCTGATGACCAAATTCCCCTCGACATCTATCGATTCCATATTTAATTAACTCAGAAGTCTCCCTTATGACATTAATTTTCAGACATCATTTATTTCTATATACTGTCATTCTATTATGTGCATATTCAAAAATAC------AACGTTTTATGAAACATTGTGAATATTGACGTATCCGGGTATTTATTTATGCCTCCTCCTCGATCTTATTTTGAGAGTACGCCCTCTTACCTAAACATGGCACATGTATGTCTAGATATCGATTATAAGTGA------TTGATCTAATGTCCCCTCGAAAAACATCGA--TTCGCTTTGAGTAAAACGATGTTTCCTAAAACCAACCGATTTGTACGCGCGAGTTTCATCTGGCATCCTCCTGGGTAAGTGAGCAACCGGCGATAACAACAGTTTTATAATACCGGTTTTTAGGCATGTCGAATTGGGTCGTCATCTTATCCATGTCTGAAGACACAAAAAGTTATAATGTTTTTTACCAAACACCTACATTGTAAGTAGCCTGCCCTACGGGCATTCGCTTTTATACTTGGATGTGATGTTAA--ACCCCAAATACTATAAATGGCTATATATATCTACATATCATGTGCGGAGTGTCGGAAGACTTCAGGAACATCTTAAGTTCCGGGATCGTGGGATATCTTTATACTCGAATTTTTTTGTTCTTTTTGGCTTTTCATCAGTTGCCCAAATGACTGGATATTGCCCCAAAGATACCCTTAACCCTCAATCCCTTTAAACATATTTCTTCTCGGATACATACTGGCCACAGCCTTT----AAAAAATATCCAACATCAGCATTTATACGTGTTTGGATTATTACATCTCAGGTAGATAGTGAGCTCTTTCATGCGATCATTAGGTCTCTTTTATGCACGGTTCAGGATCAGTATATGGACATTTATTTATATCTATCGCATAAACCCATTTCAAATCACATTGGTTGAGCGCGCTGTATGCATTGTGAGTGTTGTTTTCTTAGTACATTATGTGACATTGAATTGGTCCTTTGGCTTGCTGGATCATTATACGGATTTCTGCATCATAACGCGAATAAATGCTTTCAGGTGTCAATTGCGGAGTAAAAGGTGCTCTCGACATCAGCAAGGGCTCTTTTTTATTGCTTAACCAATGGCGGGATTATAGGTCTAAATTTT--TTACTGATATGGGTATATTTTTCTGTATGGACGCTTATGGTAGTTTCCTGGGCAATTCTTTGGGGCACGTATACAACTGTTCGCATCCTACTGGACTCATGTCATGATTGGGGGTTGCGTGAAAGCACTTACCGTTTTACTTCATTATTTCCTTATGTTTGCATGTATATCATTTTTTTTCACAACGGTCTCAACCTTGGCTAAATAAATGAAAATTCTTCACCTATTTCTTAAGAATTTGGTTTTATATCTCTATTTACTTTTACTTATGACCTAATTTAGGACCGAAATTATCCGCAACCTTGTACCCGTCAATCACCGATGTATTTTTTGCTAGCATTTCGTCATCCAAAACTGGGGGTATTTCTATCTTCATGCATTTTTATTTCCTTTCCAAAAATTATAAGGTACATTTTACATAAAACTTTTTTGGTTAGTAATTATATCTCTACTGATGGGCCGCCGTGAGACCTAATTTATGGCATCCTACTTATTATTCTTATTTTGTAAGTTCTTTTTTATATATTTGTTTGTGGGTGCTTTTACTTTTGACGAAGTTTGGTAATCAATCGAAGGCCAAAAGTGGGTATGGATTTCAGCTTAGGAGCATAATTTTACAAGAATACTACC--AAAATCAATATTATTATATTTGCCTTATTCTTCTTTATTTATATTGAGGTACCCTTTTTAGTATATAA----TTAATTGGTTTTTTTTATATTGTTAGATGGGTTAGGATATGTGCGGTGTCTCAATCAGTATCTTTTGGGGCTCGGCGTGCCAACATTCTAGAGTAGTTGGTTTTATATAAATCTATTTTGGGAGTAAGTTATGATTTTTTGGCATATATGTGTTGTTTACAGTT--CCTTTTTTTTTTTTACTCATCTGCGAACAACGACCCTTGATTGCGAGGGATCGATTGTTCGGTTAAATGATAAGAGGGGGTTGCTTATTTTTGCGATATCAGATTTTTATAGCTGTTTGTTTATTATTGGGGGAACACGGATCTTTTTTTTTGTAGTTATTCTTTCTTATTGGCCGGGACTTCCCGTACGTAGAAATTATAAGTTGTGAAAGTTTTCCGTTCTTTTTATTGTTTATTTGTGCATATGCAACTATAT----------------ATTTTGATATTTTCAAAATCTTTAGACGGAAATTTCCCTTGATGGGTTGACAAATCTCTCCGATCCTTTCTTCGTTTTTTATACATATTTTTATTTGAGTGAATGCCTATTTCCGCATAACAATAATTTCACGCAACATGTTCTAT--AAACTTTTTTATATTTTTATGGCTTTCAGATGAACAGGGCCTGATGTCTTCTGTATTTGTAGTTGATTATAGCTATATTTTTATTTATTTAATTTGATTTAG--GAAATAATTTATTATATTACTTTGGTTGGAGGGCTTGGCTTCTTTTGTTGTTGTCGTCGTGGAAGATAGGGTGGGTAGATTGGTTAATGATGATTCAGTCTTATAGTTGTTTTTATTCTTGTGTGTTGTAAGATAGTAATGGTCGAAATATCAGATTTATATTTGTTTTTTTGTTTTCATATTTTTATATAGCCAATTATAGATTTTTGGTGGAGGTTGGTTATTCTATGTTGTATTATACAAAATAATCTAAAGCGGATGTACGCATACAACGATGGGAGTATCTTTATGGATGCTGATATAATTGGAG--TGAATATTTTTATTAATATC------GAAAATTTTAATATGGGTTTATGTTTGCGCATACAAAGGCCAATCCTTTCTCTGCTCCGCGCATGCGCCCACCCGTTCGCTTGTCATCTCACTTGTACGCGGGTTATTTTATCGTTGATA--ATTTAATAAGTTATTAAATT--ATATGTGTTCGTCTACATTTATTCGGCTGGGCATTTGATAGATTAAAAATATGCTTTCACTTAGCACTGGCTATATAGACTTTGTTGGATACGATTTGTTTTCATTTTACCAGCTTTTAATCCTTTATTCAATTTCTTTGGGATCCTTTTGCGGTTTATCAAGATT--ATTTGA--AAATTTATTTAAATAAAGCTATTATTTTTATTTTTCACATTTACGTGTTAACTTCGTTATTATTAGATGTAATCTT----------AATTATCCTACA----TGTTAAGAGAGATATTATCTTTAGATATGTATGTTTATGTATATGGGGTCATATATTG------------CTATTTATGA----TTAGGGATATTTGGTTTATCAACTTTACTTGTGTTGCTACGGTCCTACATTTTTAAAT--ATTTTTATTTAT--ACCCCTTCTTGGCTACTTATCTCAACTTTTCTAGCTCTTTGGACATACTCTCTTTGTTCTTATTCTGTTTATTTATGGGGATTTGTTTTTATTAGTACTCATTTCC--AA--GAAATTTCTTACCAAAAAATTTAT--------------CTCTATTCGTTCTTCTAGACATTTTAATCTAA------------ATAATTATATACCCAATTAATATTGAAAATTTTCATAATCAAACAT------------ATATAGACAT--AAATTTAAACCAGCAAATATTTATTTTTGTAATATTTTTATGATATATGTATAAATACGCTTTTAAGG

Longivalvus_hyalospilus TTTCGTTTGACCTCACACATTTAA----TCTCAT----AATGTTAGACTT--------------TTATTTATATCTCCTTATTTTGCTGTCCACCGTTAATTATTGATAAATATTTATCTCAAAGATTAAACTTATGGCAAAAATTAACTGGAAACATATTTATTCCTTTTCCTATTTTTAAAAGTCTGGTTTTCCTAATTTACAGACAGCAATTCATACTTGCTTTCTTCCTTTGATGCTTATCTTTGG--TGATAATTAGCACAATTTAGCATTATCCCAGGACCCGCATTTATCCTTATGTTGATGAATATAGAAATATCGCCGGACCTGCATCGCTTCGCAAATATGCGGCACTCTCTACTTTTGGAAACGGCCTCTTTCATTTATTTAAATCTGTTTACCAATCTCTCTACTTGAACGCGTGCTTATCATCTAGTTTGCATTTACACTTTATCACATAACCATTGTGTTTCCTTTCTATTTGTACACCTACATTCAATC--TTAATATT------TATCAAAATCTTTCTCTCTCTT----ACAAAAAAACATAAGTAATTTTTCACAACAAAGAATGGACCTTATTATTTGGATTGGCGGATGTGGTCAGTTAGATTTATCGTTGATTAGCACCGGTTTTTTGAGAGACAACTAAAGTATGTACGCCAGCTTATATATTTTTATATATCCATATATGGGGTTGGAATGCTGTCCTTATCTGGGCCCGAATGCTTCCCGATAAAAATAGTTTGTTTTCCCCTCTTACCTTTATTCAGAGTTGTAAACGGGCGGACGGTGACGTTACCCCTTGCAGACTTGGCACCGGGCTCGTGACTGCATTTTCCTCACTGCGGGTAGTCATTTGGGCGTAATTATACACATATAAATCGTCCAGGTTACTTGACGATCCTTTTGTTGTCGTTTATACGCATCTCTCTTTTCCTCCGTTTGCGGGCATACATCTTTACGACGAATTAAACTCTTTTGACCGCGGGGGGGACCATCTTACACATTTTTGTTTTGGCACCGAGTTAATTTATTTCCGGTTGGTTATTCCAGTATAGCAGAAGGGAAAAGAACTTGGGTTTGGATATTAGCATCTGCATGGCTTTGGTTGTGTTGGCCACAATTTACGTGGATAAGTGAACCGGCTATTACTCGCACATATATGCATCCACGGATAAATTTAGTGTTACACCTTAGGGCAAATGCTTACCCACATTTTGAGCTGGTTGTTTTTTTACATGGGGTTACGGGTATCTGCAATCTCATGAATGTCTCAGAACTATAGTGTGCCATTCATAGTTTTCATGGGCGTTTGCATATGCGGTTATCATGTACCTTTTACGGATGTTTAAGAAACTTTAAATCATTTTGTATTTATGGGTAAATACTTTTCCCACATTCTGGTTAGGGATCCCGCGTAAGGATACCGAGTTAACTCTGAAGTATTCTCCTGGAGATATTCTTATGGATATTTCTATTAATATTGGAAGTTAT--ATCGACCCTCTTTAAATATCAATCTCCTGATGACCAAATTCCCCTCGACATCTAGCGATTCCATATTTAATTAACTCAGAAGTCTCCCTTATGACATTAATTTTCAGACATCATTTATCTTTATATACTGTCATTCTATTATATGCATATTCAAAAATAC------AACGTTCTATGAAACACTGTGAATATTGACGTATCCGGGTATCTATTTATGCCTCCTCCTCGATCTTATTCTGAGAGTACTCCCTCTTACTTAAACATGGCACATGTATGTCTAGATATCGATTCTAAGTGA------TTGATCTAATATCCCCTCGAAAAATCACGA--TTCGTTTTGAGTAAAACGATGTATCCTAAAACCAACCGATTTGTACGCGCGAGTCTCATCTGGCATCCTCTTGGGTAAGTGAGCAACCGGCGATAACAACAGTTTTATAATACCGGTTTTTAGGCATGTCGAATTGGGTCGTCATCTTATCCATGTGTGAAGACAAAAAAAGTTATAATGCTTTTTACCAAACACCTACATTGTAAGTAGCCTGCCCTACGGGCATTCGCTTTTATACTTGGATATGATGTTCA--TCCCGAAATATTATAAATGGTTACATATTTTTACATATCATGTGCGGAGTGTCGGAAGACTTCAGGAACATCTTAAGTTCCAGGATCGTGGGATATCTTTATACTCGAATTTTTTTATTCTTTTTGGCTTTTCATCAGTTGCCCAAATGATTGGATATTGCCCCAAAGATACCCTTAACCCTCAATCCCTTTAAACATATTTCTTCTCGGGTACATACTGGCCACAGCCTTT----AAAAAATATCCAACATCAGCATGTATACGTATTTGGATTATTACGTCTCAGGTAGATAATGAGCTCTTTCATGCGATCATTAGGGCTCTTTTATGCACGGTTCAGGCTCAGTATATGGACATTTATCTATATTTATCGCATAAACCCATTTCAAAACACATTGGTTGAGCGCGCTGTATGCATTGTGAGTGTTGTTTTTTTAGTACATTATGTGACATTGAATTGGTCCTTTGGTTTGTTGGATCATTATACGGATTTCTGCATCATAACGCGAATAAATGCTTTCAGATGTCAATTGCGGAGTAAAAGGTGTTCTCGACCTCAGCAAGGGCTCTTTTTTATTGTTTAATCAATGGCGGGATTATAGGTCTTAATTTT--TTACTGACATGGGTATATTTTTCTGTATGGACGCTTATGGTAGTCTCCTGGGCAATTCTTTGGGGCACGTATACAATTGTTCGCATCCTATTGGACTCATGTCATGATTGGGGGTTGCGTGAAAGCACCTACCGTTTTACTTCATTATTTCCTTATGTTTGCATGTATATCATTTTTTTTCACAACGGTCTCAACCTTGGTTAAATAAATGAAAATTCTTCACCTATTTCTTAAGAATTTGGTTTTATATTTTTATTTATTTTTACCTATAACCTAATTTAGGACCGAAATTATCCGCAACCTTGTACCCGTCAATCACCGATGTATTTTTTGCTAGCATTTCGTCATCCAAAATTGGGGGTATTTTTGTTTTCATGCATCTTTATTTCCTTTCCAAAAACTATAAGGTACATTTTACCTAAAATTCTTTTGACTAGTAATTGTATCTCTACTGATGGGCCGCCGTGAGACCTAATTTATGGCATCTTACTTATTATTTGTATTCTATAAGTTCTTTTTTATATATTTGTTTGTGGGTGCTTTTACTTTTGACGAAGTTTGGTAATCAATCGAAGGCCAAAAATGGTTATGGATTTCAGCTTAGGAGCATAATTTTACAAGAATACTACC--AAAATCAATATTATTATATTTGCCTTATTCTTTTTTATTTATATTGAGGTACCCTTTTTATAATATAA----TTAATTGGTTTTTTTTATACTGTTAGATGGGTTAGGATATGTGCGGTGTCTCAATCAGTATCTTTTGGGGCTCGGCGTGCCAACATTCTAGAGTAATTGCTTCTATATAAGTTTATTTTCGGAGTAAGTTATGATTTTTTGGCATATATGTGTTGTTTACAGTT--CCTTTTTTTTTTTTACTCATTTGCGAACAACGACCCTTGATTGCGAGGGATCGATTGTTCGGTTAAATGATAAGAGGGGGTTGCTTATTTTTGCGATAGCAGATTTTTATAGTTGTTTGTTTATTATTGGGGAAACAGAGATTTTTTTTTTTGTAGGTATGGTTTCTTATTGGCCGGGACCTCCCGTACGTAGAAATTATAAGTTGTGAAGGTTTTCCGTTCTTTTTATTGTTTTACTAATTTTAATGAACCAAAA----------------AGCTATTAAATTATAAAAATACCTTGCGGAAATTTCCCTTGATGGGTTGACAAATCTCTCCGCTCCTTTCCTCGTTTTCTATACATATTTTTATTTGAGTGAATGCTTATTTCCGCATAAAAATTATTTCAAGCAGCATGCTGTTT--AAACACTTTTTTATTTTTATGGTTTTCAGATGAACAGGATTTGATGTCTTTTATATTTTTAGTTGATTGTAGTTATATTTTTATTTATATAATTTGATTTTC--GAAATAGTTTATTATATTACTTTGGTTGGAGGGCTTGGTTTCTTTTGTATTTAGTCTTAGAGTAGATAGGGTAGATAAATTGATTAATGATGATTCAGTCTTATAGTTGTTTTTATTCTTGTGTGTTGTAAGATAGTAATGGTCGAAATATCAGATTTATATTTGTTTTTTTGTTTTCATATTTTTATATAGCCAATTATAGATTTTTGGTGGAGGTTGGTTATTCTATGTTGTATTATACAAAATAATCTAAAGCGGATGTACGCATACAACGATGGGAGTATATTTATGGATGCTGATATAATTGGAG--TGAATATTTTTATTAGTGGA------GAAAGTTTTTATATGGGTTTATATTTGCGCATACAAAGGCCAATCCTTTCTCTGCTCCGCGCATGCGCCCACCCGTTCGCTTGTCATCTCACTTGTACGCGGGTTATTTTATCGTTGATA--ATATAGTAAGTTATTAAATT--TTCTTTGTTCGTTTACATTTATTCGGTTGGGCATTTGATAGATTAAAAATATGCTTTCACTTAGCACTGGTTATATAGACTTTGTTGGATACAGTTTGTTTTCATTTTACCAGCTTTTAATCTTTTATTCAATTTCCTTGGGATCCTTTTGCGGTTTATCAAGATT--ATTTGA--AAATTTATTTAAATAAGGTTATTATTTTTATTTTTCACATTTACGTGTTAACTTCGTTATTATTAGATATAATCTT----------AATTATAGTACA----TGTTAAGACAGATATTATTTTTAGATATGTATGTTTATGTATATGGGGTCATATATTG------------TTATTTATGA----TTAGGGATATTTGATTTTTCAATTTTACTTATGTTGTTGTGGTTTTAGATATATAAAT--ATTTTTATTTTT--AACCCTTCTTGGTTGTTTATTTCAACTTGTCTAGATACATGGACATAC--TCTTTGTTTTTATTTTATTTGTTTATGGGGATTTGTCTTTATTAGTACTCATTTCC--AA--GAAATTTCTTAACAAAATTTATAT--------------TTTTATTCGCTTTTATATTTATTTTCTTATAA------------ATAATTATATAACTAACTAAAATTGAATATTTAAATAATCAAATAT------------ATATAGACAT--AAATTTAGACCAGGAATTATTTATTTCTGTAATATTTTTATGATGTATGTATAAATACAATTTTAAGG

Pediculus_capitis ATTCTCTTGACCTCACTCATATCTGG--TTAAGT----AATGTTATATCT--------------TTCCTTTTTTATACGGAGTATATTATCATCGGTACGTTTAGTAGTTGTTTAAAATTTTATAACATAATTC----------------------ATATAGTTTCGTTTATATATTTACCTAAACATTCCTATCCTTGTTTCCTGACTCCATTAGGTAATTGGCTTGTTCCTTTGATAGGGGTGTTATC--TTAAAGTCATCGGGTTCTGCCACTCTCCTAGGAGCCATATCTAGCCTTTTGTGTATGATTTTAGGTTCATCGCCGTTCCTAGGTCGCTCTGCAAATACGGGGCATTATATAACTTTGAGAGGTTTCTCGCGTCTCTGTCTCCTTTCATGCGCTAGTCTCTTTGCGCGACTTTGTTCTTATCATCTAGTTTAGAACTGTTCATTATGGACTTGCCTCATTGACTTTATAATATGTATTTGTTTTATTATTTAC--ATATTATTATAAATGAAATCAATATTGAAATTAATT----AAAAAAACATTCAACTTCATTTTCACAACAAAGAATGGTTTTTATTTGTCGGGTTGTTGGCTTTGGTTAGTTAGTTATATCGTTGACTTCAGACGGTTCTTTTCGAAGCACTTAAAGTTTGTACTCCAGCTTGTATATTTTTATGTATCCGTATATGGGGTTGCAATGTTGTCCTCATTTGGTCCCGAATGCTTCCCGATAAAAATAGTATGCTCTACCCTCGGATTTCTATAGAGTCTTGTCAGGGGGTGGACGGTGACGTTACCCCCTAGTCCTGAGGCACCTCGTTCGTGATTGCATTTAGCTCATTGCGGGTAGTCATTTGGTCGTAATTATAGACATTTAAATTGCCCATATTGGTTGTCGCTCCTTTTTGTGAGGTTTGTACGCTTTTTTTTCTTCCTCCGTTTGCGGGCATACATCTTTATGACGAATTAATGTCTTTTGACCTTGGGGGGGACCGTTTTACACATTTTTGTTTTGGCACCGAGTTAATCTATCTCCGGTTGGCTATTCCAATGTGTGATGTGGGAAAAGAGTTTGGTCTTGGATATTAGCATTCGCATGGGCTTGGTTGTGTTGGCCACAATTTACGTGGTTGAGTGAAGCGGCTATTACAGGCACATACATGCATCCACGGGTAAGTTTAGTGTTGGACTTTTGGCCAATTAAAGAGATAGTTTTTGTCTTGGTTATTTCTTTACATGGGGTTACGGATGTCTTCAATCTCGTGAGTTCCTCAGAACTATAGTGTGCCATTCATAGTTTTCATGGGCGTTTGCATTTGGGCTGAACATGTTTCCTGGACGGCTAACTCGAATCTTATAAGTCATTTGTTAGTTGTGGGTAATTACTTTTCCCACATTCTGGTTGCGGATCCCGCGTATCGATACCGAGTTACTAGTGAAAAATTCTCATGGAGCTATACACTTGGGTGTATTTCTTTGCCTATGAAGTTTCAACCCAAAATGTTTAGGAGCAGTTCAGACTCCCGCTATGGATCCGCAGATCATCCATTACCTACTTACAGGTTGGGTTCAGAAGAATCCCTTATGTTTGTTGGAACTAGACTGTTCATGTTGGTGGGTATTCTTGTATTAGTGCGTAGTTTTTTATAATCTG------AATATATTATGGCTGAAGTTGAATGTTGGTATTTCCTCCTTCTTGCGGTTATTTCCTCCTCATGTTTATTATGAGAGTCTTCCCGCATAGTTAAGTGTGGCACATGTTTGTCTAGATAGGGATGGAAAATGA------TTGATCTAATATAATTGAGACTGATCTCTGCCTTCGCTTTGAGCGATTAGGTTTATCCTATTACGAGTCGGCATGTACTCGCGAGTATCATCTGGCATCCATATGGGTAAGTGAGCATCCGGCGTTAACAGCCTATTATCTTAAATGGACTCTAGGCATGTCGAATTGGGGCTACAAGTTATCCATAAGTACACCTCCAAGAGATTATAATGGTAAGATTAATTCACCTTCACTGTGAGTAGCCTGCCATTTTTAGTTTCCTTTTTTCGCTCATACTTTGTGATAAGGCTTATCTTTAATTTATATAGATGTGTTCTCTTATGTTCTTTGTGCGGAGTACCGGAGCACTTCAGGAACAACATGAGTATGCGGTTCGTTGGATCTATTTATGCTCGAGTATTTTTTTTCTTTTTAGCCTTTTTCTTCTTAGCCGAGTTCTTGGCTCTTACCCCGTGGGTAGCCGTGGGTTTGGGTCCCTTTAATCATTTTTCTTCAGGGGTTCATACTGGCCATAGACTTT----AGAAAAATTCTCAGCTATGGTTTTATACTTATTTGGCTGTTTCTATTTCAGCGTGATAAAACAGTCTTACATGCGAAGAGTTGGTCGTTTTTCTATACGGTTCAGGGCCAGTTGGTGGGTGTTTATACATAGACATCGCTTATTAACATAAAAAAACACATTGGCTGACTGCGCTGTATGCATTGTGAGTGTTGTTTTTTTACTACCTTATGTGATCTTTAATTTGGTTTTTGGTTTTCTTCATCAATTTAGGGCTTTCTGCTCCATAGAGCTCACAA--TCTTTGAGGTATTTATGATTGAGTAAAGGGTGTTATCGAGTTCAGCAAGGGCTCTTTTTTATCTGTTAGTCAATTGCGGGTTTGTTGGTGTTACCAAA--TAGTTGTTTCGGATTCATCTCTCTATATGCGCGCTTATGGTAGTCTCCTGGGCAATTCTTTGGGGCACGTATACAACTTTAGGCATCCATGTGGAGGATTGTATTGGTTGGGGGTTTCGTAGCACCACTTGACGCTTTACCTCATTCTTTCCTTGTTTTTGGTTGTATGCCAATATCTCTCACACAGGTCAGAACCTTGGTTGATTGAAGGAAAGTTATTTACCTATTTACTAAGAATTTGGGGTTGTTGTTTTTTTTGTTTATTGATTATCCCGATTTTATGACCGAAATTGTGATCAACCATATACCCCCCAATCACCGATGTATTCTTTGCTAGCATTTCGAGGTCCAAAATTGGGGGTGTGCTTCTCTAGATTTTCCTTCTTATAGAT------GGAGTCGTTCAGCGTTAGATAGCGATATTTACTATCTTACAGGTTTGTATCTTCTGCTGGTCCTCCGCGATACCTTACCTCTAGCAGTGTAGGTATTATTATCAGTATCTTTATCATCGTTTATGTTTTCCTTTATTGGTGCTATTTCTTTTGACGAACTCTAGTTGACAATCGCTGGCCAAAAGTGGCCATGGATCTCACCTTAGGAGCCCAATTTTTCAAACATTGCCCA--AGGA----TCTGGATTTTATATCCTTATACTTATTTAGGTTCTGTGTACCCTTATTCCAAACTTTG----GATCAAAATCCTTTATCTATTTATTCAGGTAGGTTAGCTTATTTACGGTCCTCCAATCAATATCGCCTGGAGCTCGGCATACCTTCATTCTTGATTGTTTTCACGCATCTAGATGCGTGTTTAATCTTAGATAATTATGCACAACAAG--GTCCAAATATTCATAT--GTGTGGATTTGTTGACTCCTATGCGATGGGCGACCCTTGATTCCGAAGGATCGATTGTAGGGTTAAGTGATAGGGGAGCGTAGTTTCTTATTAGGAAGTTTTCTACATTCTCATATATAGATTTTAACTG--GGTAAACCTTGTGTTTACTGATAGATTC--------ATGTATCGGCAGGCCCCGATCGTAGAAATGATATTTGGTGGATTTCATCCTTATTTATTTATAGTTACATTTATATTTGCATATTTGC----------------TTGTACTTGTTTGTTCCAAGCCAATTACCTGAAGACCCTAGATGGGGTATCCTTAGATAGACCTTCACCAATCATTTAGTGTAGGTGTTTCTATTTGAGTGATTGTGCACTTCCGTGTACTC----AGCTTTGAAAGATGTTTCAT--TGCTTTATCCCTATCTACTTGGTTCTTTGACTCATAGGAGTTGATGAAATATTCTCCTATTCCTGATTAGTGTGTGGTTAGTATATCAATGCCTTCGA--AGTTGAACCTCTAGTCGAGTTTAGGTATGTTGGAAGGTGTGGCTTCTTCTATAGCTACTAGGTGGGAGACGGTTAGGGGTGAATATATGTCTGAAATTAGCTACTTTTCTATGTTTACATAGTCCTGTATGCTAAGAATATAATGCGGCAAACTGGGGGATTTAGTTCATGTTTTTATGTAGATTTCTCTTCCTAGGGTCATTTTGTCTTATGGTGGAGGTTGGATATAGTTGTTTATTTTTAAAAAGTGAGTCCAAATCGGGTATACTTCTATAACGTTGGGATCTTATATATTGTCTCTATTAGGTTGGGAT--TGGA----------------------GTTATGTTTGATTTACTCTTTATTATGGGGGCTCAAAGGCCATTCCTTTCAGTGTTCCGAGCATGCGCCCACCCGTAGAGTTGTCATCTCACTTGTACGCGGATTAGTTTGCCGTAGGAG--ATATGAAGTTTAAT--------CTACTATTTCTCATTCATATATTCGGGTTCGCCTTGAGAGGATTAAAAGTGTGCTATCACCTTCCAATAGTTATCTTTTATTTCGAGGAGGTGAGGGCTTATCAATTTACCATCGTTTAAAGCTTTTTTCTGCTTCATGCGGCTCCTTCTTCGGGGTATCAAGAGT--TT------CTATTTAGTTAAAGTCATATAACTATATTTCTACGCGTATTTACAGGGTATCTTCGATATTATTCTTCAGAA--------------ATAAATACCAAA--------ATGCGTAGAGTTTTAGAGCCCTAACTAGCAATCTAAGTCT------ATTCGCTGATTCTCAGCCGGTATTTCAACT----AGAGCA--AGGTAATTGAGGAAATATATCTTTATATCTTTGGATGTGCATTTTTACTT--CTATTTGTGGTC--GACTTTCTAACTTTTCCTGCATACGTTTACGTGGCTCTATCTCTAATCTATCTCTGTCTGTTCTTTTGTTGGATTTGGGGTTATGTTCCTTCATGCTTATGTACCC--AA--ATAATCTCAAGATGTCAGAGTTAG--------------TTATAGGCTTTGATTTACTCGTATCTTCGCAT----------------ATTGAAGTGATTAAGATGTCGATTTATCACGA--AGGTAGGA------------ACTAAATCCT--CTATGACTAAGTTAACGCGTGTATCTTTAT----TAATTTATCTCCGTATGAGTTTACTCCCTAAGCG

Heterodoxus_macropus CTTCATTTGACCTGTCAGATTTAAAT--TTAATT----AATGATGTATAG--------------TTTGTTTTATCCTTAATCTTTGAAGTCCAGATTTTTACTTTTGATAATAAAACTATAATCATTTACAA------------------AAGTAATTCAATTCAGATTTTTTTATTTGTTTAAATTTGGATTTTGTTACTTTCGTACAGCATTGTATAATTTCTTGGTTTCATTGGTGGACTTTTTAAGTCATTAAA--TTAGGATTTTGCCATTACCCATGGTGCCATGTTTGTCCTTATGTGTATGATTATAGATATATCGCCATACTTTCTTCGCTATGCAAATTTGCGGCAATATTTTCTTATAGACGGGTAGTTATCTTCTTTTATCCTCGGATTTTTTTCTGGTTTATTTTGAATGGGTGCATATCAGCTAGTTTTCATCTCTTCTTTATGGAATTTCCTACTTGATTGATTTATTTCTTTTCGTTTATTTTTTTAT--AATTTTTTTCAATTATTCAACGAAAAAGTATGAAAA----CATAAATTGAAAAATTGATTTATCTCAACAAAAAATGGATTTTAATATCTGGAGTGTCGGCTTTGGTTAGTTAGATATATCGTTGATTTCGATCGAATTATTTTAACCCAATTAAAGTGTGTACTCCAGCTTTTATATTTTTTTATATCCTTATATGGGGTTGCAATGTTGTCCATATAAGGAGCCGAATTCTTCCCGATAAAAATAGTTTGTTTTCCCCTCTTATTTATTTTGAGATATCTGAGGGGTCGGACGGTGACGTTACCCCTTTCTCTTACGGCAGCGGATTCGTGAATTTATTTTCCTCACTGCGGATAGTCATATGGGCATAATTATACACATTTAAAT--GTTTTTAAAATTTCATATAGTTTTAATGTCGTTTATACGCTTTTTTCTTTTCTTCCGTTTGCGGGCATACATTTTTTTGACGAATTAATCAGTTTTGACCATGGGGGGGACCATTTTACACATTTTTGTTTTGGCACCGAGTTAATTTATTTCCGGTTGGTTATTCCAATATGTCAGAAGGGAATGGAACTTGGGTTTGGATATTAGCATTTTCATGGATCTGGTTATGTTGGCCACAATTTACATGGATGAGTGAACCGGCTATTACTCGCACATATATGCATCCACGGATAAATTTAGTGTTTCACTTTTGGAGAAATAATTAATCTCGATTTGAGATGGTTGTTTTTTTACGTGGGGTTACGGGTGTTTGCAATCTCATGAATGTCTCAGAACTATAGTGTGCCATTCATAGTTTTCATGGGCGTTTGCGTTTTCGCTTACCATGTTCCTTTTTTGGGTAAATAGAAGCTTATATCTCATTTGATACTTTTGGGTAATTACTTTTCCCACATTTTGGCTAGGGATCCCGCGTAATTGTACCGATTTATATCTGAATTTATCAGATGGTCATATACTCGTAGTTTTATTTGTTTATATTTTAAGTTTTGAAAAAAATTTTTTTATCTTAGATAATCATGATGATCTGGACCCCCTCGCCATCTTAAGAAGCCGTTTATGATTAACTTCGAGGTGTCTTATATGAAAATGTGCTTCAGATTACTTATATCTTTTTATACACGTGTCTATATTTATTCATATATACAATTGT------AACGTTTTATTAAAGAGTTTGATTATTGACGTATCCAGTTATTTTTATATGCTTCCTCTTAAATCTTATTGTGAGATTCTAACCGAGTACGTAAGTATGGAACATGTATGTCTACATATCGATTTTAAATGA------TTGATCTAATAAAATGGAGGCT----TCGA--TTAATATTGAGTGAAACGACGTCTCCGTGAACAAATCGATATATACTCTCGAGTATCATCTGACATCCAGTTGGGTAATTGAGCAACCGGCGTTAACATTAAATTTGGAACGTTGGTTTTTTGGCATGTCGAATTGGGATTTCATCTTATCCATTGGTGAATGTAACCGATGTTTTAATGTTTAAAAAGGTTTTTTTTCAATGTGAGAAGCCTGCCTTTTCTTCTTAGGTTTTTAAATTTAGGCTTGTTATTAA--TTCAATTTATTAATTTCTAGAAATTTAGATTTATTTTAATTGATCGGAATATTCGAAGACATCAGGATCAACTTAAGTCAAAGGATAAATGGATGTTTTTATACTCGAGTATTTTTTTTCTTTTTGAGTTGGTATAATGTAGCAGATAATCT--AGAATGCCCTTTGGATATAGTTAACCTCACGTCCTTTTGGACATATTTTTAGTCGGGTTCGTACTGTGCAAAGACTATTTAGGAGGAACTAGAGATAAAATCTTTTATACGTATCTGGATGTTTGCGCCTCAATTGGATATTATTCACTTACATAGGAGGGTTAGGTCTTTTTAATATACGGTTCAGGTTCAGTATGTGGACATTTTTTTATATTTTTCGTTAAAATACATTTCAGCACACATTGGTTCAGCGCGCTGTATGCATTGTGAGTGTTGATTTTTTAATATTTTATGGGTAATTGAATTGGAGTTTTGGCTTGCTTTATCAATGGTCGGTTTTTTTCCTCATAAATCAAGTGATTGCTTAGAGGTATTAATATAAGAGTAACAGGTGATTTCGGTATCAGCAAGGGTACATATTTATTTATTAATCAATGCCGGGCTTATAAATCTAAACTAC--TTGTTGTTGTGGATTTATCTCTTTACATGGACGCTTTTGGTAGTCTCCTGGGCAATTCTTTGGGGCATGTATACAATTATAGACATCCTATTGGGTACTTGTGATGGTTGGGGGTTTCGTAGGACCACTTACCGTTTTTCTTCATTATTTCCTTGTATCTGGGCTCGCTTCAATATTTTTCAAATATTAGTCAACCCTGGTT--CCAAACGAATATTCTTCACCTTTTACGTAAGAATTTGGGTGTTTTTTTTTAGTTTTTTTTTCTTACGACCTAAATTATGACCGAAATTATTTGCAATCATGTACCCGTCAATCACCGATGTATTTTTTGCTATCATTTCGGCGTCCAAAATTGGGGGTATGGTTTTATTCATCTGTTTGCTTTTTTTTTCAAAAAGAATCCAGAAGGTTATA------AATCTTTGTGGTCATTACATTTATTTTTACTGACGGAGTTCCGTGATCCCTTTTGAATGGCATGTTTCGTATTATTTTAAATTTTTTTTTCACATTATCTGTATATATCTCTACGTGCTTTTACTTTTGACGAAATTTGGTAATCATTCGAAGGCCAAAAGTTTTTAAGGGTTTCACCATGTGAGCATAATTATACAAGAGATCCCAT--ATTAAGAAATTTTTTATAATTCCCATTTAGTTATATAGATATATTGATATCTCCTTCATTATATTTAA----TGGTAAAGTTTTATTTTTATTTCTGGATGGGTTAAGATTTTTTCGGTGTCTCAATCAATAGCTATTGGAGCTCGGCGTAGCATCATTCTAGAATTTATAGATTTTTATGCTTATATACACAAGGATAGATTATAATTTAATTGACC------TTGTTTTTTTTT--CCTTTTATGCTATTTTATGGTTGCGATTAACGTCCCTTGATTTCGAGGGAAGGATTGTGCGGTAACGTGATAGGGGATATTAACATATTTTTAGGAAAATATATATTTTTTGTAATGGTCTTTTTTTT----TAATAAAGACATAGATATTTTCATATATATTATTGTTGTTATCGGGATCTCCCGATCGTAGACATTATATTTTGTGAAATATTTCCTTATGTATTTGTAACTTTCTAGAGCTATATTTGTTTTA----------------TACTAGGTTTTTATGAAAAAAAATGTGAGAGGAAAAGATTGATGGGTTCGGCGAAATTTCCGTTCCTTTCATCATTTTAGATGCTTGTTTCTATTTGAGTGATTATATATTTCCTAATTTAATTAACAATTAAT--------------TTAGATATATATTTTTTATTGGACCTTTGATGATGAGGAGTTGATGTAATATATACTTTTAGTTGATTATGGTTGTAATTATATATAATTTTTTCC--------AAATAATTATTTATATGTACTTTTATTTGAAGGTTTATTTATCTTAACTGTTATCGGATTGGATGAAGTTATGATGAATTAATTATTTGAAATTTCATATTTTTTTATGTTTATATTCAAAAGTTTAATATCTATTTATTGTGGACGTTGACTTCGTTATGGATTTATTTTTATGTTCATTTTGTTATATTCTAGAATTTACTTATGTGGTGGAATTTGGGTTCTCTTTTTTATTTTATAAATCTAAATCAAAAAGAGTTATACTAATAGAACGTTGGGAGGTTTTATTTGCATGTTTGCAGCCTTTTAG--GATTTTTTTTAAGCCA------------------TATTCTCCATTTGTTTTGACAGATACAAAGGCCATTCCTTTCAGTGCTCCGAGCATGCGCCCACCCGTTCACTTGTCATCTCACCTGTACGCGGTTTATTTTTTCGTTCAGACTTGATAAAAATTAGCTTAATT--TTTTTTATTCTTTTACATACTTGCAGAGGCGCTTATGATAGATTAAAAGTATGCCTTCACTTAGCAATAGTTATTTTTAGTTAGTTAATTACACTTGCTTTTCAATGTATCAGCTTTTAAGCGCACTTTGCAATTCTTTCGGTTTTTTCTTCGGTTTATCAAGATT--ATTAAATCTTTTGCTAAAAATTAATATATTATATGTTTCTATTCATGTTTACATTTTATGTTCGATTGTTATATATAATT--------------TTAAATACTTTA------TTAATTAAGAATAAATTTTTCCGTTTGATTATTTCTTCATGTTCGGTCGTTTATTG------------------TATT----TGGATCGTATATCCTCAATTGATTATAATATTTTTTATATATTTATTCATTTTCAGATTCTTTTTACTAGAACTAGAAATATAGTTATTTGTCTATAAATATTCGTTCGGTTATATATTATCTTTCTCTTGAGGATATTTGTATGTTTTTACGGCTTTATTTATTCTATTGTAGGTACCC--GA--AACCAATTGTAATTTTTAAAGAAAGCCATCAAAATTTATTATAATGATCTTTTTTCATTTTTTTATTTTT------------GCTTTATGGATTAAAGCATCTTTGTTGAGTAATTAG--AAGAAATT------------AATTTCTTTT--TCCCTTAACAATTCATTAATGTAAATTTATTTTTCTTTTTATGTTGATTAATCGTTACTAACAAAAGG

Ibidoecus_bisignatus TTTCATTTGACCTGGTAGTAACAG----TTCATT----AATGTTATTCTT--------------AGTGATATGTATACGTAATTATAATTGATA--GTAATTTATATTGTGTTCAATTTTATTCTCTCAAACTTTTAAAGAGTA----AAGTGTTCGTAAATGTTCGTTTACATATTTATTGAACATTAGATGTCCTTGTTTGGCCACAGCATTTCTTAATCGCGTGCTTTCAGTGTTGCGGATATACAT--CTTTATTCTTAAGATCGTTCCATTGTCCTTGGAGCCATTTTTACCCTTTTTTATGTGAGTATAGTGTTATCGCCGTGCTTAGGTCGTTATTCAAATATGCGGCAATATATGTTTTTAGAACTATTGAGTTAATCTA------TATTATCCATGATCTTATTTTTTTGATTTGATTCATGTCAGCTAGTTTTCAGCTTTGCTTTATAAATTTTCCTGACTGTTTTATTTTTTTGCCTTGGTATGTTTTGCTTCTTATAATTTTGTCTATGAGAAGACTTAAAAACCCAAAA----GAAACAAACGGAGCTTACTTTATCACAACAAAGAATGGATCTTATTATTTGGATTGTCGGTTTTGGTAAGATAGCTATATCGATGACTAGCAATACAATAATAAGAGGCAATTAAAGTATGTACTCCAGCTTTTATATTTTTATATATCCATATATGGGGTTGCAATGTTGTCCTTATATGGTCCCGAATGCTTCCCGATAAAAATAGTTTGTTCTATCCTCTTTTTTTTTTATAGATTTATGGGAGGACGGACGGTGACGTTACCCCTTTCAG------CAGCTCATTCGTGAATTCATTTTCTTCATTGCGGTTAGTCATTTGGGCATAATTATTGACATATAAATTGTTTC----TCATTTTTCTCCTTTTTGTGTCATTTATACGCTTTTTTTTCTTCTTCCGTCTGCGGGCATACATTTCTCTGACGAAATAATGTCTTTTGACCATGGGGGGGACCATTTTACACATTTTTGTTTTGGCACCGAGTTAATTTATCTCCGGTTGGCTATTCCAATATTGGAGAAGGGAAAAGAGTTTGGTCTTGGATATTAGCATTTTCATGGATTTGGTTGTGTTGGCCACAATTTACGTGGATGAGTGAAGCGGCTATTACGGGCACATATATGCGTCCACGGATAAGTTTAGTGATTCACTTTTGCAGAAATAATGTCGTTCTCTTTGAGTTGGTTGTTTCTTTACATGGGGCTACGGGTATCTGCAATCTCATGAATGCCTCAGAACTATAGTGTGCCATTCATAGTTTTCATGGGCATGTGCTTATGCAGTTTTCATGTTCCTTATTTGGGTTACTAATCAATTTTAAATCATTTTGTACTTATAGGTAAATATTTTTCCCACATTTTGGTTGCGGATCCCGCGTAATGATACCGAATTTAGTCTGAAGTATTCTCTTGGTCACTTTCATATAGTTTTATATATTTTTATTTGAAGCTATTCAACGCTGTGTTTAGTGAAATCCATTCATGATGGTAAGGTTCCCCAGAACATGAAGAATGTCCCATTACACCTTGTTCAGAAGAATCCCTTATATCAATAACACTCAGACAATATGTGTATATATATATTCATGTATTAGTTTTTACATGTATAACCTGTC------AACGTTTTTTGGAGGAGTTTGATTATTGACTTGCCCAGATGTTTGCATTTGCATCCTCCTCAATTTTATTATGAGATTAA--CCATATAGATAATCATGGCACATGTATGTCTAGATAGGGATTTGAGATGA------TTGATCTAATATATGACAGATTGATTGGAT--ATCGTTTTGAGTGAAACGACGTATCCGTGGATGAATCGATCTATACTCACGAGTATCATCTGACATCCACCTGGGTAAATGAGGGTCCGGCGTTAACAATTATTTCAGAAATTGGGTTATTAGGCATGTCGAATTGGGAGTTCATCTTATCCATTGTTGAGTTTTCGATCCGTTATTCTGTTAAAATAGGTTCACCTTCAATGTTCATAGCCTGCCATTTTGTCTTTCATATTCTTGTATAATCTTTATAATAAAATTTTACTTGATTCTTTGATCTTTTTCTTATTTGTATTTTGTGTGCGGAGTATCGGAAGACTTCAGGTTCAATAAAAGTTGTTGGTTTAATGGGTTCATTTATATTCGAGTATTTTTTTTCTTTTTTGGTATTTTTCAGTTGTCCGAGTGAATGGTGTCTGCCCCGTGGGTCATCTTAGTTATGAGTCCTTTTAAACATATCTCTTCAGGGATTCATACTGTCCACATCTTTT----GAAAAATTACAATGTTTTGGATATTTACGTATTTGGTTATTTACTTTTCATTATGATATTGATGTCTTTCATGCGAAGGTTAGGTCCTTTTAATTCACGGTTCAGGATCAGTATGTGGACTTTTATATGTTCTTATCGATATAATACATTTCATCACACATTGGTTGATTTCATTGTATGCATTGTGAGTGTTGTTTTTTTTTTAGGTTATATTTTATTGAATTGGTCTTTTGGATTGTTATGTCAATTTTCGGTTTTCTTCATCATAAAACTCATGAGAGCTTAAAGGTTTTCACTGAAGAGTAATTGGTGTTATCGTAATCAGCAAGGGCTCATTTTTATCTGTTATGCAATGGCGGGTTTATTGGAGTTAAATAC--TTACTGTTTCGGGTATATCTTTTTTTATGGACTCTTTTGGTAGTTTCCTGGGCAATTCTTTGGGGCACGTATACAATTGTAGACATCCTAGTGGGACATTGTTATGTTTGGGGGTTTCGTAGGACCACCTAACGTTTTTCATCATTATTTCCTTGTTTATATGTGTTTGTCAATTTTCCTCAAAAGGGAGAGAACCTTGGATTCCCAATGTTAAATTCTTCACCTATTTGAAAAGAGTTTGGTTGTGTGTTTATATTTACGTACTTATTTCTCCGAGTTTATGACCGAAATTTCGTGCAACCATTCACCCGCCAATCACCGATGTATTTTTTGCTAGCATCTCGTCATCCACAATTGGGGGTGTGCTTGTTTTCATGTATTTTTATATCCTTATAGAGGGAAAAAAAGCTAATTTA----CAAAATATGTTTATCAGTTCAATTCTTTTTACTGTTGGGCATCCGTGATTCCTTTTATATAGAAATTTTCTCATTATTATTTATATTTTTCTCATTTTTTATATGTGGTTTTTTTCGTGCTTTTTCTTTTGACGAAATTTAGGTATCATTCGAAGGCCAAAAGTGGTTATGGTTTTCACCTTTCGAGCATAATTTTTTAAAGAAGAATCC----AATTAGAAATTTTATATTGCCCATGTTTTTATTTTCATTTATTGATAGATCCAGAATGAATTTTAA----TTTCTCAGTTATTTGTATTTCTTAGGATCCATTAAGATATTTATAGTGATTCAATCAATATCAAATGGTCATCGTCGTGCCATCATTCTAGAATATTTTCTCTGTTTTTTTTATATATGTTATCTCTCATAATTTTTATTTACATCTAGTTGCTTTTACCTGTT--CCATTTTTATATTTATTCATTTGCGAAGAACGTCCCTTGACTACGAGGGAAGGATTGTTCGGATTTGTGATTGGGGGTTGTAATTTATTTTTGGGAAATTTATTTTTTTCTCTTTTATTCTTAGATTTGGAATCTTATGTTTAATTGTATATACATTTAT--------GTTGATCGGGACGTCCCGATCGTAGAAAATATGATTTGTGATAGATATCCATTGATTCTTATTCATGTATACGTTTTTAAATGTCTTG----------------TTATTCATATTTTTAAAAGAATAA--CAGAAGAAGAAGTTGATGGGATGAACTTTTAATTAATCTTTATGTTCATTTTTTATGGGTTTTTTTGTTTGAATGAATATATTGATCCATATTTTT----AATTGAATATAAATTTTTTA--TGAGTTATATATGTTTATGTGGTATATTGATTGCATGGACTTAATGAAATATACATTTATTCTTGAATTT--GTTGACATTTTTTTATATTAATGAAATCATATTCATTCTTTTCTTTTTTCTGATTTATTGGAGGGTGTGGTTACATTTTCAATTTAAAAATAAGTCTTAACAGTTCTTTCTTTCATATGTGAAATATCTTATTTATATATGTACATATAGACATGTATATTATCATTATAATATGAGAAAATAAAAAATTTTTTTCATTTTTTTATCTTCATATATCTTCTTTCGCAAATTTTGTTATGTGGTGGAGGTTGGCTTCTCTTATCTATATTATTCAAATGAAAGTTAAAGTCATACACTTATTGAACGTTGGGATTTTATCTATAGATGCTTATGTAATTTTTC----AATTTGTTTCTTTTAATC------AATTTTAGTTTTTTTGTTTTATATGTTGGCATACAAAGGCCAGTCCTTTCGTTGCTCCTTGCATGCGCCCACCCGTTCTCTTGTCATCTCACTTATACGCGGGTTTTTTGATCGTTAGAG--ATCTATGAGTCATTCTTTAT--TTTCTAATTCTCTTACTTATATTCGGCTTCGCATTAGATAGATTAAAAATATGCCTTCACCTTCCAATGCTTATTTTTTTTTAGATAAAGTTGATCTCATATCATTATACCAGCATTTAATCTCTTATCCATTTAGATATGGATTTTTTTTCGGTTTATCAAGATT--ATGGAT--ACATGTTGTAAAACAAAATGTAATGGTTTATTATGCGTGTTTACTGATTATCACCGTTTTTAATTTATAAAAAAATAA--------AGAAATTTTAAA----------GAGAAACATCATTATAATTTTAGTAGAGAGATTCATTTTTGGGGTTCTTTAA------TTATACTCTTTGAATT----CAAAGAGTATTTATAATTTTTTTGTTATCTTTTTATTTATGGATTTATTTTCGTTTAT--TTTTTTATTCTC--TCTCGTATATTTTTTTATATATAGTTTTATTTGGGTTTATTGTCGTTCTTAATTTTTTTCTTATTTTTTTATACTTGGGGTTTTATTTTTTCTTATCCATTT------------GATTAATTAGATAAAATC------------------TTATAATTGATTTTTTTATTTTATATATTTGT----------------TGATAAATAATCTTGATTGTAATTTATTAATT--GAGAATAT------------AATATAATAA----------ATGATTACGGTTTCTTTTTTATTTATATTTATATTTTTATTTGATCATGTAGACTCGAGG

Lepidopsocid_sp TTTCTCTTGACCTTACAAATTTAA----TTCCTT----AATGCTAGTCTT--------------TTTTATATTTATCCTCTTTATGATTTCCTCCGATCATTATTGAATTATATAAACTTCAAAGATTAAACCTTTAAAATCTC--AATTGAAGACATATTTATTCATTTAGTTGTTTATAAAATTTTGGTTTTCCTAATTTACAGACAGCATTGTTTACTTACCTTCCTCCTTTGTTAGTTATATTAGG--TGTTAAAAACAACAATTTGCCATTGTCCAAGGACCCGGATCTATCCTTATGTTGATGAACATAGAAATATCGCCGGACTTGCGTCGTTACGCAAATATGCGGCATTATTTACTTTTGGAAACGGTCAAATACATTTTTGTACTTTTATATATCAATGCTTTTATTTGAATGCGTGCTTATCATCTAGTATGCATTTATACCTTATCAGATATCCATCCTGTTTCTTTTTTCTTTCTTTACTTTTATACAATG--ATAATATT--------TATTCACCAAATTTTTTCAA----AATATTAAATAAAAAAATATTTTCACAACAAAGAATGGACTTTATTCTTTGGATTGGCGGATGTGGACAGATAGATTTATCGTTGATTGGCACCGGTTTTTTGAGAGACAATTAAAGTATGTACGCCAGCTTATATATTTTTATATATCCATATATGGGGTTGGAATGTTATCCTTATTTAGGCCCGAATGCTTCCCGATAAAAATAGTTTGTTTTCCCCTCTTACTTTTTTATAGAGATACAAGTGGGCGGACGGTGACGTTACCCCTTTCGCGCGTGCCAGCGGGCTCGTGATTGCATTTTCTTCATTGCGGATAGTCATTTGGGCGTAATTATTCACATATAAATCGTCAAGGTTACTTGACGTTCCCTTTGTTGTCGTTTTTACGCATTTTTTTTTTCTTCCGTTTGCGGGCATACATTTTTACGACGAATTAAACTCTTTTGACCGCGGGGGGGACCATCTTACACATTTTTGTTTTGGCACCGAGTTAATTTATTTCCGGTTGGATATTCCAGTATAGCAGAAGGGAAAAGAACTTGGGTTTGGATATTAGCATATGCATGGTTTTGGTTGTATTGGCCACAATTTACGTGGATGAGTGAACCGGCTATTACTCGCACATATATGCATCCACGGATAAATTTAGTGTTGCACTTCAGGTCAAATTTTTTCCCTCTCTTTGTCTTGGTTGTTTTTTTACATGGGGTTACGGGTATTTGCAATCTCATGAATGCCTCAGAACTATAGTGTGCCATCCATAGTCTTCATGGGCGTTTGCATATGCGGTTATCATGTTCCCTCTACGGTTACTTAAAAAATGTTAAATCATTATATATTTATGGGTAAATACTTTTCCCACATTTTGGCTATGGATCCCGCGTAAGGATACCGAATTAACTCTGAAATATTCTCTTGGTCACATTCTTATGGATATTTTTATTTATATTGGAAGTTATTCAACGAACCATTTTCATCAATTCTCTCATGATGTTCAAATACCCCTCGACATCTAAAGATTCCATATTAAAATAATTCAGAAGGCTCCCTTATGACACTATTTTTCAGACATCTTTTATATACATATACGTATGTTCTAATATGCTCTTTTTTAATCTTAC------AACGTTTTTTGAAACAACATGAATATTGACATATCCGGATGTTTATTTATGCTTCCTCTTCGCTCTTATTTTGAGAACAAATCCTCATACTTAAACATGGCACATGTATGAGTAGATATCGATTAAAAATGA------TTGATCTTATATCCTCAAGAAATTAATCGA--TTCGCTTTGAGTAAAACGACATTTCCTTAAACCAATCGATTTGTACGCGCGAGTTTCATCTGGCATCCTCTTGGGTAAATGAGCAACCGGCGTTAACAACTCTTAAATAACGCCGGTTTTTAGGCATGTCGAATTGGGGCGTCATCTTATCCATGTATGAAGGTCAAAAAAGTTATAATGCTAATTCATCAACACCTACATTGTGAGTAGCCTGCCTTACGGGCATGGACATATTTACTCGGGTGTAATGTTCA--ATTTAAATTTTTTTTTATGGATATATATTTTTACATTTCATGTGCGGAGTGTCGGAAGACTTCAGGAACATCATTCGTTCAAGGATCGTGGGATATTTTTATACTCGAGTTTTTTTATTCTTTTTGGCTTTTCAAGAGTTTCCCTCATGAATGGATATTGCCCCAAGGATCACCTTAACCTTCAATCCTTCTAAACGTATTTATTCTCGGATACATACTGGCCACATCCTTT----AAAAAAAAAGCAACATCAAGTTGTATACATATTTGGTTTATTACATCTCAGGATGATATGGAGCCCTTTCATGCGAGCATTAGGTCTCTTTTATGCACGGTTCAGGATCAGTATATGGACACTTATTTATATCTATCGCAATAAAACATTTCAATACACATTGGTTGAGCGCGCTGTATGCATTGTGAATGTTGTTTTCTTAATTCATTATGTGACTGTGAATTGGTCTTTTGGTTTGTTATATCAATTCTCGGCTTTTTGCATCATATCGCCAATGATTGCTTTCAGATATCAATTGCGGAGTAAAAGGTGATTTCGACATCAGCAAGGGCTCTTTTTTATTGTTTATTCAGTGGCGGGATTATAAGTCTAAATTCA--ATACTGTTATGGATTTATTTTTTTACATGCACGCTTGTGGTAGTTTCCTGGGCAATTCTTTGGGGCACGTATACAATTTTTCGCATCCTATTGGCAATTTGTCATGATTGGGGGTTGCGTGAAAGCACTTATCGTTTTACTTCATTATTTCCTTATATTTGCATTCATATCATTTTTTTTCACAACGGTCAAAACCTTGGATAAATAAATGAAAATCCTTCACCTTTTTCATAAGATTTTGGTAATATATCTCTATTTATTCTTAATTAGATCCTAATTTGGGACCGAAATTACCCGCAACCCTTCACCCGTCAATCACCGATGTATTTTTTGCTAGCATTTCGTCATCCAAAATTGGGGGTTTGCTTTTTTTCATTTATTTTAATTTCCTTTTAA--AAAATTCGTCACCATTTACCATAAAAATTTTTTGTCTTACACATTTATTTTTACTGGCGGGCAACCGTGAGACCTTATTTACAGCAATTTACGTTTTATTTCTTTTATATAAATAATTATTTTTTTATGGATCTGTGGGTGCTTTTACTTTTGACGAAGTTTGGTAATCAATCGAAGGCCAAAATTGGTTATGGTTTTCACCTTAGGAGCATAATTTTACAAGACAGTTTCC--AAATAGAATATACCTATATTTCCCGTTTTTTTTTTTTCTTATTCTGATATATCCTATTTGGTTTAAAA----TTAGTTGGTTTTTTTTATGTTGTTAGTTGGGTTAGGATATATAGGGTGAGTCTTAGTTTAGCTTTTGGAGCTCGTCGTGCCAACATTCTAGAGTAGTTTCTTATTTATAGATTTATTTATGGAATAAGTTATAGTTTATTTACAAATATTTATTTAATTTGGTT--CCTTTGTTATTGATGTTCATTTGCGAGTAACGACCCTTGATTGCGAGCGATCGATTGTTCGGTTAAATGATAGGATGGGGTTGCTTATTTTTGCGATATCAGATTTTTATAGATGTTTACTGTTTTTTGGGCGAATCTAGTTTTTTTTTTAACTTTTTGTTCTTTTTTATTGGTCGGGAGTTCCCGTTCGTAGAAATTATAATTTGTGAAAGTTTTTCTTTCCTTTATTTAGGTACTTACATTTCTTCATTTTTAA----------------TTATGCACTTCTTCTAAAACATGAGACAGAAAATTCCCTTGATGGGTTGACCATAATCTCCGATCCTTTCCTCGTTTTTTATACATATTTCTATTTGAGTGAATACTTATTTCCATATTTAATTAAAATCAAATTTTTTGATTTAT--ATTTGTTTATTTATTTATTTGGTTTTCAGATGAACAGGGCTTGATGTCATTTATATTTATAGTTGATTATAGTTATTTTTCTTTATTATTAGTTTTAATTAATTGAGGTATTTTATATTTTTACTTTGGTTGGAGGGTATGGTTTCTTCTGTGGATATCGTCCAGGAAGATTTCGTAAATCAATGTATTTTTGATGATTCATTTTTTTAGTTGTACTTATTCTGTTATTCAATAAGAATCTAATTTGGGAAAAATCAATTTTATTTTTATATATTTGTTTTCATATTTATATGTAGCCAAATATAGATTTTTGGTGGAGGTTGGTTGTTCTAATTTGTATTATACAAAGTAATCTAAAGCGGATTTACGTTTTCAACGATGGGAATATATTTATTGATGGTGCTTTAGTTGGAG--TGAATATATTTAATAAATATACACGAGATATTATAGTTATGGTGTTATATATGCGGATACAAAGGCCAATCCTTTCTCTGTTCCGCGCATGCGCCCACCCGTTCGCTTGTCATCTCACTTGTACGCGGGTTATTTTATCGTTTTCC--TTTTAAATAGAATTAGATAT--TTTTTTATTCGGTTACATTTATTCGGTTGGGCAATTGATTGATTAAAAATATGCTTTCACTTAGCATTGGTTATATGGAGTTTCATGGTTACAATTTGTTTTCATTTTAGCAGCTTTTAAGCTTTTGTTCAATTTCTTTGGGATCCTTTTTCGGTTTATCAAGATT--ATTTGA--TTATTCATAGGAATAATTATTCTATTTTTTTTGCTCACGGTTACATAGTATCTTCGTTTTAGTTTTATTTGGAATT----------AATTTTAGTTCA----TGATGAGAAAGATAATATATAATCATTTGGTTTTTTGGTCTTTTGGGGAGATTTATTG------------TTATTTCCTT----AGAAATATTTTTCCTTTTATAATTATGTTTTTTTATTTATGGACTTTTTCACTTTCAT--TTTTTTTTATAA--CACCTTTCGCGGTTATTTATATTCACATTTGTGCCTATACGCTAATCTCAACTTTGTTTCTAATTTACTTATCTATGGGGATTTATTTTTATTAATATAGTTTCCC--AA--CAAATTATATTCTCATCTTTTTATCCTT----------TTTTATATCCATATATAAATATGACCATATTT----------------------------ATGATTTCCAAAATAAGACA--ATAATGAA------------TTCCCATCAT--AATTTTAAACAATCAGATTTACATATAGATAATATTTTTTATATATATGTACAAATACTCAGTTAAGG

Liposcelis_bostrychophila TTTCATTTGACCTCAGAACTGTTC--------------AATGTTATTGCT--------------ATTTTTATTTAA------------GTAAACCCTCAGCTGTGAGTTCTTAGAATTCT--TCGTTTATAAGAATAAATATAA----ATCATTTTTTTTCTTTCCATTTATTTATTTGCTTAAATTCGGATTTCCTTACTTACCTACAGCAATAGATACTTTCTTGCTTCCATTGTTAGCTATATATGG--TGTTAA--TTAAAGATTTGCCACTGTCCTTGGTGCCACGTTTATCCTTATGTTTATGAACATAGCTATATCGCCCTACTTGCGTCGCTGCGCAAATATGCGGCAATATTTTCTTATAGATAGGCTTAATCTCATGTTTGTTC--TCCTTTGCGATCATATTTCTTTGATTGCGTGCATATCACCTAGTTTTTATCTTTACCTTAAGCAATGCCCTTTGTGCTGGAGCTATTTATTTTTGGTTACATTTTTCA--ATTTTATA--------ACACGAATTCACCTGAAATT----AAGAAAATACTTTTAATTCTTTTCACAACAAAGAATGGTCCTTATTATTTGGGTTGTCGGCTTTGGCTAGCTAGCTTTATCGGTGACTTCTTAATCAGAGCTAGTCTC--GTTTAAAGCTATACTCCAGCTTCTATATTTTTTTATATCCATTTATGGGGTTTCAATGATATCCCTTTATAGTCCCGAATGCTTCCCGTTAAAATTAGTTTGTTCTCCCCTCTTCTTTATTCTTAGATATGTGGCCGGGCGGACGGTGACGCTACCCCCTTCGCATGAGCCATCGGTTAGGTGACTGTATTTTCCTCATTGCGGATAGTCATCTGGGCATAATTATACACTCATAATTTGATGACCCGCATTGATTCTCCTTTTAGTGTCGTTTATACGCTTCTTTCTCTTCCTCCGTTTGCGGGCATACATCTTTTTGACGAATTAGACTCTTTTGACCTCGGGGGGGACCATCTTTCACATTTTTGTTTTGGCACCGAGTTAATTTATTTCCGGTTGGCTATTCCAATATTCCAGAAG--ATAAGAGTTTGGAGTTGGATATTAGCATCTTCATGGGCCTGGTTATGTTGGCCACAATTTACGTGGATGAGTGAAGCGGCTATTACTCGCACATATATGCATCCACGGGTAAGTTTTCTGTTACACGTTAGGAGACGTACCCTCTCTCACTTTGAGCTGGTTATTATTTTACATGGGGTTACGGATATCTTCAATCAGATGAGTATCTCAGAAGTATAGTGTGCCATTCATAGTCTTCATGGGCGTTTTCATTTAGGGTTAATTTGTTCCCTTTCTGGGGTCGTAAGATTAAAAAAGTCATTTTTTACTTATGGGTAACTACTTTTCCCACATTCTGGCTTCGGCTCCCGCGTATCGATACCGACATAACTACTAACTATTCTCATGGTCTGATAGATATAGATATTGTTATACCTATTTGAGGGTATAAAAAATCGTATTTATTTACCCTCTCAGATGATGATGAGGCACCCCAATTCAACTGTTTATCCCCAATTTAATTATTTTTGAAGAGGGCCGTATGACAATAGGATTCAGACAGCATATATCTTTCTATGTTCTTTTACATGTTTTGGTACTTACAAAAGGTT------AACTAAATTTACAGGAGTCTGAATTTTGTCTCCTCCGTTTATTTTTATCTGCATCCTCATCAGTCTTTATATGAGAGTATTCCCCTATACATAAATATGGAACATGTTTGACTAGATAAGGATTACAAGTAA------TTGATCGTATAGAAACAAAT------------TTCGCTCTGAGTAAAAGCCTATCTCCATACACCAGTCGCTCTTTTCTCAAGAGTATCATCTGACCTCCTCTAGGTTAAATGAGCAACCGGCGCTAAATGGTCCTTAAGTACGTCGGTATTTAGGCATGTCGAATTGGGGTGACATCTTATCCATAAGTGGTTACTCCTGATGTTAAAATTATAAAGGTAACTAGGATTCACTGTGAATAGCCTGCCTTATATAGTTACACGCAAACATCTTCCTTAATAAATAATTTTTCTGCTTTTTATACATTCATAT------TTGTTTTTCTTGTCCGGAATATCGGAAGACTTCAGGATCACCTTAAGTCACTTCCTAATAGGATATCTTTATACTCGAGTATTTTTTTTCTTTTTGACTTTTCATCGCCTAGCCACAAGAATGGAATCTGCCAGTGGGGTGACCATAACCTTGGATCCTTCTAAACCTGTTTGTTCTCGGGTTCATACTATCCACAAGATTT----AACAAATTAATTACATCTTGGTGTATACGTCTTTGGGGTATTACATCTCACTATGATAATACTCTCTTTCATATGATCGTTAGGTCATTTTTATTCACGGTTCAGGATCAGTCTGTGGACCTATATCTTATCCTATCGCTTTAGTTCATTAGTCGCCACATTATTTGATTTCTGTGTATGCATTGTGATTATTGCTTTCTTTCTTCATTATGTGTATTTGAATTGGTCCTCTGGCTTGCTTCATCAATTTACGGGTTTTTACATTTTTAAGCGACTAGCATCTTACAGGTGTAGATATAAAAATAAAAGGTGATATCGTTATCATCACGGGCTCATTTTTATATTGTAGCCAGTGGAAGCCTTTTTTCTCTTTATTTG--AAGTTGGTTCGGCTGTTTATCTTTCTATATGAGCTTCTGGTAGTTTCCTGGGCAATTCTTTGGGGCACGTATACAATTATTCGTATCCTATTGGCCCTGCGTCATGCTTGGGGGTTAAGTGGGACCACCTACCGTTCTTCTTCATTATATCCTTATATATGCATAGGGGTCATTATCTCTCAGAACGGTCTCAACCCTGGATCCTTAAATGAAAGTAGTTAGAATTTTATATAAGACTGTACCTGCCTGTTTTTGGCTATTTCTAGACATTCCCTTATTTATGACCGAAATTCTAAGCAACCATGTACCCATCAATCACCGATGTATTCTTTGCTAGCATTTCGTCGTCCAAAACTGGGGGTTTATTTGCTTTCATATATATTTATCTCCCTTTTCAAAAAAATAAGGCTAATTAGTTTT--AATGCTCTTATTCATTGGTCTTTCATTTACTGCTGGATCACCGTGAGACCTTATTATTGGAAGTTATCGTCTTATTATTTTATTCTTTATAATTATTTCAATGTTTATCTTTAGGTGCCTTTACCTTTGACGAAATCTGGCTATCACTCGAAGGCCTGAAGTGGCCTTGGCTTTCACCTTTCGAGCTTAACTTTTCAATTTCTCGCCC--ATAAGGAATTATCTTATATTACCCCTTATTCTATCTTCTTGTTTTTTTAAAACCTTTTTCACTCTT------TTACTTTCATTTGTTTCTTTCTTAACACAGGTTAACACTTGTACGGTGTCTCAATCAATATCCTATGGTCATCGAGATGCCATCCTTCTAGAATACCTGGCTCTTTTTAGTTGCTTATATTCTCACTTATTTAAATATAATTAATCTCATCTCACTTTTATGCC----CTTCATATTTTTCTAATATTATGAAGAACGACCCTTGATTTCGATGGATCGACTGTTCGGTTAAGTGATTGGGGGCGATTTCTTATTTTTGGGAAATTATTTGTTTAATCTTGTTTTCTTTTATGGAGATTCGTTACTATTTTGCTGTATATTTATAA--------GTTCATCGGGGCTACCCGTACGTTGAAGATATGATTTGTGTTATTACTCCCTACATGTCTCTAGGTTTACTTATCTATGTGTTTTTAT----------------TTTTGTCAATGTAAAGGTGGAAGGGAAGTAACGACACCTTGATGGGATAGAGAATTTCTCCGATCTTTTCCTCCTTTTTTATACTTCTTTTTATTTGAGTGAATATCTCTTTGTTTATTTTC----GACTTCGTATTTTTCTTTTAATGTATTTTT----CTCTATGCAGCTCTATGATGTATAGGTCCTGTTGATCTCTATATATCTAAATGAATATATGTTTATTTTTTTATTTAATAAAATAA----------ATGTATATATTTATGTATATGTTGGAGCATATGGCTATTATGGCTGTGTCTTATTTAAAACTAATCATTCTGAGTGTTTTTTTTGAGCTATCGCTCTTTCTCGTGTCTTTATTCGCTCATGTTTTATCATTCTAATCACAGAAAGAAAATAATTTTTTACCTTAATTTATCTTCATCTATTTATTTTCTTAAATTCTCCTCTGTGGTGGAGGTTGGGTACTCTTCTTTATTATATACATCTTAAAGACAATCTCCTATACTTACTTAACGGTGGGATTATATATTTTCATACATGGCTACGTTAAC--TGAATTTTTTAGGTGAACAT--------------AAGTTTAATTCTTTATACGCGCTTAGAAAGGCCACTCCTTTCTCTGCTCCCTGCATGCGCCCACCCGTTCTCCTGTCATCTCACCTGTACGCGGGTTACTCTTACG--GCCC--TTTCATATAGTGCTAGAATA--ATTTTTATACAGCTACCTATATAGAGGTTTGCTTCAAGTTGATTAAGAATGTGCTTTCACATAGCAATAGTTATATATGGATTCAAGGCTTAAATTTCTTTTCACTTGACCAGCTTTTAAGCCTTTATTCAGTGTCATATGGCTCCTTATGCGGTTTATCAAGAGG--CTATGA--GAAGTCTT--------TTTCGTTCATCTTTGTATCCGTCTTTTCTCCTTAACATCGTTTTTATAATTGTTTCAAAC----------TCGGATAGTTAA------------ACAAGAATATAGTTTCATTTTCTTGCTGTTTCATTTAGGGGCGCATCATG------------AGCTTTCCCT----CTTTATTCATATCC--CATTAAACTTATTTATATTTATATAGATGTATTTTAATACGT--ATTTACTTGAAG--TCATTTAGGCTTGTTAATTTGTATATTTTTGGAGGGATATGCACGTTCGGAGGTTGATAGATATTTTTATTTATATGGGGTTATGTTCTTTTTAATGTAGTTACCA--AA--ATGTTTAGATTCCCCTGTTT------------------TTATAGGTTGCTGTCCTTAGCTGCAGTTGGAA--------------------------TTCAAATGTTGATATTTTTTTC----------------------------------------TCCCAGTACTGTGTGTTTTTATTTTTTTCTTTTTATCTTTATATGATTAACTAAGCATAAGG

Pediculus_humanus ATTCTCTTGACCTCACTCATATCTGG--TTAAGT----AATGTTATATCT--------------TTCCTTTTTTATACGGAGTATATTATCATCGGTACGTTTAGTAGTTGTTTAAAATTTTATAACATAATTC----------------------ATATAGTTTCGTTTATATATTTACCTAAACATTCCTATCCTTGTTTCCTGACTCCATTAGGTAATTGGCTTGTTCCTTTGATAGGGGTGTTATC--TTAAAGTCATCGGGTTCTGCCACTCTCCTAGGAGCCACATCTAGCCTTTTGTGTATGATTTTAGGTTCATCGCCGTTCCTAGGTCGCTCTGCAAATACGGGGCATTATATAACTTTGAGAGGTTTCTCGCGTCTCTGTCTCCTTTCATGCGCTAGTCTCTTTGCGCGACTTTGTTCTTATCATCTAGTTTAGAACTGTTCATTATGGACTTGCCTCATTGACTTTATAATATGTATTTGTTTTATTATTTAC--ATATTATTATAAATGAAATCAATATTGAAATTAATT----AAAAAAACATTCAACTTCATTTTCACAACAAAGAATGGTTTTTATTTGTCGGGTTGTTGGCTTTGGTTAGTTAGTTATATCGTTGACTTCAGACGGTTCTTTTCGAAGCACTTAAAGTTTGTACTCCAGCTTGTATATTTTTATGTATCCGTATATGGGGTTGCAATGTTGTCCTCATTTGGTCCCGAATGCTTCCCGATAAAAATAGTATGCTCTACCCTCGGATTTCTATAGAGTCTTGTCAGGGGGTGGACGGTGACGTTACCCCCTAGTCCTGAGGCACCTCGTTCGTGATTGCATTTAGCTCATTGCGGGTAGTCATTTGGTCGTAATTATAGACATTTAAATTGCCCATATTGGTTGTCGCTCCTTTTTGTGAGGTTTGTACGCTTTTTTTTCTTCCTCCGTTTGCGGGCATACATCTTTATGACGAATTAATGTCTTTTGACCTTGGGGGGGACCGTTTTACACATTTTTGTTTTGGCACCGAGTTAATCTATCTCCGGTTGGCTATTCCAATGTGTGATGTGGGAAAAGAGTTTGGTCTTGGATATTAGCATTCGCATGGGCTTGGTTGTGTTGGCCACAATTTACGTGGTTGAGTGAAGCGGCTATTACAGGCACATACATGCATCCACGGGTAAGTTTAGTGTTGGACTTTTGGCCAATTAAAGAGATAGTTTTTGTCTTGGTTATTTCTTTACATGGGGTTACGGATGTCTTCAATCTCGTGAGTTCCTCAGAACTATAGTGTGCCATTCATAGTTTTCATGGGCGTTTGCATTTGGGCTGAACATGTTTCCTGGACGGCTAACTCGAATCTTATAAGTCATTTGTTAGTTGTGGGTAATTACTTTTCCCACATTCTGGTTGCGGATCCCGCGTATCGATACCGAGTTACTAGTGAAAAATTCTCATGGAGCTATACACTTGGGTGTATTTCTTTGCCTATGAAGTTTCAACCCAAAATGTTTAGGAGCAGTTCAGACTCCCGCTATGGATCCGCAGATCATCCATTACCTACTTACAGGTTGGGTTCAGAAGAATCCCTTATGTTTGTTGGAACTAGACTGTTCATGTTGGTGGGTATTCTTGTATTAGTGCGTAGTTTTTTATAATCTG------AATATATTATGGCTGAAGTTGAATGTTGGTATTTCCTCCTTCTTGCGGTTATTTCCTCCTCATGTTTATTATGAGAGTCTTCCCGCATAGTTAAGTGTGGCACATGTTTGTCTAGATAGGGATGGAAAATGA------TTGATCTAATATAATTGAGACTGATCTCTGCCTTCGCTTTGAGCGATTAGGTTTATCCTATTACGAGTCGGCATGTACTCGCGAGTATCATCTGGCATCCATATGGGTAAGTGAGCATCCGGCGTTAACAGCCTATTATCTTAAATGGACTCTAGGCATGTCGAATTGGGGCTACAAGTTATCCATAAGTACACCTCCAAGAGATTATAATGGTAAGATTAATTCACCTTCACTGTGAGTAGCCTGCCATTTTTAGTTTCCTTTTTTCGCTCATACTTTGTGATAAGGCTTATCTTTAATTTATATAGATGTGTTCTCTTATGTTCTTTGTGCGGAGTACCGGAGCACTTCAGGAACAACATGAGTATGCGGTTCGTTGGATCTATTTATGCTCGAGTATTTTTTTTCTTTTTAGCCTTTTTCTTCTTAGCCGAGTTCTTGGCTCTTACCCCGTGGGTAGCCGTGGGTTTGGGTCCCTTTAATCATTTTTCTTCAGGGGTTCATACTGGCCATAGACTTT----AGAAAAATTCTCAGCTATGGTTTTATACTTATTTGGCTGTTTCTACTTCAGCGTGATAAAACAGTCTTACATGCGAAGAGTTGGTCGTTTTTCTATACGGTTCAGGGCCAGTTGGTGGGTGTTTATACATAGACATCGCTTATTAACATAAAAAAACACATTGGCTGACTGCGCTGTATGCATTGTGAGTGTTGTTTTTTTACTACCTTATGTGATCTTTAATTTGGTTTTTGGTTTTCTTCATCAATTTAGGGCTTTCTGCTCCATAGAGCTCACAA--TCTTTGAGGTATTTATGATTGAGTAAAGGGTGTTATCGAGTTCAGCAAGGGCTCTTTTTTATCTGTTAGTCAATTGCGGGTTTGTTGGTGTTACCAAA--TAGTTGTTTCGGATTCATCTCTCTATATGCGCGCTTATGGTAGTCTCCTGGGCAATTCTTTGGGGCACGTATACAACTTTAGGCATCCATGTGGAGGATTGTATTGGTTGGGGGTTTCGTAGCACCACTTGACGCTTTACCTCATTCTTTCCTTGTTTTTGGTTGTATGCCAATATCTCTCACACAGGTCAGAACCTTGGTTGATTGAAGGAAAGTTATTTACCTATTTACTAAGAATTTGGGGTTGTTGTTTTTTTTGTTTATTGATTATCCCGATTTTATGACCGAAATTGTGATCAACCATATACCCCCCAATCACCGATGTATTCTTTGCTAGCATTTCGAGGTCCAAAATTGGGGGTGTGCTTCTCTAGATTTTCCTTCTTATAGAT------GGAGTCGTTCAGCGTTAGATAGCGATATTTACTATCTTACAGGTTTGTATCTTCTGCTGGTCCTCCGCGATACCTTACCTCTAGCAGTGTAGGTATTATTATCAGTATCTTTATCATCGTTTATGTTTTCCTTTATTGGTGCTATTTCTTTTGACGAACTCTAGTTGACAATCGCTGGCCAAAAGTGGCCATGGATCTCACCTTAGGAGCCCAATTTTTCAAACATTGCCCA--AGGA----TCTGGATTTTATATCCTTATACTTATTTAGGTTCTGTGTACCCTTATTCCAAACTTTG----GATCAAAATCCTTTATCTATTTATTCAGGTAGGTTAGCTTATTTACGGTCCTCCAATCAATATCGCCTGGAGCTCGGCATACCTTCATTCTTGATTGTTTTCACGCATCTAGATGCGTGTTTAATCTTAGATAATTATGCACAACAAG--GTCCAAATATTCATAT--GTGTGGATTTGTTGACTCCTATGCGATGGGCGACCCTTGATTCCGAAGGATCGATTGTAGGGTTAAGTGATAGGGGAGCGTAGTTTCTTATTAGGAAGTTTTCTACATTCTCATATATAGATTTTTACTG--GGTAAACCTTGTGTTTACTGATAGATTC--------ATGTATCGGCAGAACCCGATCGTAGAAATGATATTTGGTGGATTTCATCCTTATTTATTTATAGTTACATTTATATTTGCATATTTGC----------------TTGTACTTGTTTGTTCCAAGCCAATTACCTGAAGACCCTAGATGGGGTATCCTTAGATAGACCTTCACCAATCATTTAGTGTAGGTGTTTCTATTTGAGTGATTGTGCACTTCCGTGTACTC----AGCTTTGAAAGATGTTTCAT--TGCTTTATCCCTATCTACTTGGTTCTTTGACTCATAGGAGTTGATGAAATATTCTCCTATTCCTGATTAGTGTGTGGTTAGTATATCAATGCCTTCGA--AGTTGAGCCTTTAGTCGAGTTTAGGTATGTTGGAAGGTGTGGCTTCTTCTATAGCTACTAGGTGGGAGACGGTTAGGGGTGAATATATGTCTGAAATTAGCTACTTTTCTATGTTTACATAGTCCTGTATGCTAAGAAAATAATGCGGCAAACTGGGGGATTTAGTTCATGTTTTTATGTAGATTTCTCTTCCTAGGGTCATTTTGTCTTATGGTGGAGGTTGGATATAGTTGTTTATTTTTAAAAAGTGAGTCCAAATCGGGTATACTTCTATAACGTTGGGATCTTATATATTGTCTCTATTAGGTTGGGAT--TGGA----------------------GTTATGTTTGATTTACTCTTTATTATGGGGGCTCAAAGGCCATTCCTTTCAGTGTTCCGAGCATGCGCCCACCCGTAGAGTTGTCATCTCACTTGTACGCGGATTAGTTTGCCGTAGGAG--ATATGAAGTTTAAT--------CTACTATTTCTCATTCATATATTCGGGTTCGCCTTGAGAGGATTAAAAGTGTGCTATCACCTTCCAATAGTTATCTTTTATTTCGAGGAGGTGAGGGCTTATCAATTTACCATCGTTTAAAGCTTTTTTCTGCTTCATGCGGCTCCTTCTTCGGGGTATCAAGAGT--TT------CTATTTAGTTAAAGTCATATAACTATATTTCTACGCGTATTTACAGGGTATCTTCGATATTATTCTTCAGAA--------------ATAAATACCAAA--------ATGCGTAGAGTTTTAGAGCCCTAACTAGCAATCTAAGTCT------ATTCGCTGATTCTCAGCCGGTATTTCAACT----AGAGCA--AGGTAATTGAGGAAATATATCTTTATATCTTTGGGTGTGCATTTTTACTT--CTATTTGTGGTC--GACTTTCTAACTTTTCCTGCATACGTTTACGTGGCTCTATCTCTAATCTATCTCTGTCTGTTCTTTTGTTGGATTTGGGGTTATGTTCCTTCATGCTTATGTACCC--AA--ATAATCTCAAGATGTCAGAGTTAG--------------TTATAGGCTTTGATTTACTCGTATCTTCGCAT----------------ATTGAAGTGATTAAGATGTCGATTTATCACGA--AGGTAGGA------------ACTAAATCCT--CTATGACTAAGTTAACGCGTGTATCTTTAT----TAATTTATCTCCGTATGAGTTTACTCCCTAAGCG

Haematopinus_suis ATTCGTTTGACCTGTCACTTTTAGTTAATTCCTT----AATGTTCTGTGT--------------TTGTGTTTAGCTAGGGCGTATGATCTTCTCGGTTCATGGTATGTTGGTAAAAGGCTATCAGGTTCGGATCTAAAAATAAA------CATTATCTATCTCAACCTTTTTTTATTTTCAGAATTATGGCTTCCCTTATTTACCTTCTCCATTGTTAAATTAGTTTGTTCCTTTGTTGGGGATTTTATC--TGTCAATGTGAAAAACTTGCCATTACCCGTGGAGCCGTGCTTGCCCTTTTGTTTGTGAACGTAGTTATATCGCCATAGTTAGGTCGTTATGCAAATACGCGGCAATGTATACCTGCGACAGGGCATTCGTGCTCTAGTGGAGTTTAGTTTTTGTATGTTTTTTTTTGATTGGGTGCCTATCAGCTAGTTTATAGCTATTCTTTATGGAATTCCCATTGTGTTCTCTGAGTTGTTTTGTAGTTATAGTGTGAG--TGTTTATGGATTTTGTGAAGTTGCTCCCAAGTTTTT----AAGTTTAAGAGGGATAGTTTCTTCACAACAAAGAATGGGTCTTATTATTTGGGTTGGCGGTTTTGGACAGATAGTTTTATCGGTGATTGGAGGTAAAGTTATTCAGGGCAGTTAAAGTTTGTACGCCAGCTTTTATATTTTTATATATCCGTATATGGGGTTGGAATGTTGTCCACATTTGGGCCCGAATGCTTCCCGATAAAAATAGTTTGCTTTCCCCTCCTTTTTTTATTCAGTTATGTGGGGGGGTGGACGGTGACGTTACCCCCTAGGGTTGTGGCACCAGAGTCGTGATTACATTTAGCTCATTGCGGTTAGTCATATGGGCATAATTATTGACATGTAAATTGGTTGGGAAAATTGACTTTCCCTTTTGTGTCGTTTATACGCGTCTCTTTCTTCTTCCGTTTGCGGGGATACATTTTTATGACGAAATAATGTCTTTTGACCTTGGGGGGGACCGTTTTACACACTTTTGTTTTGGCACCGAGTTAATCTATTTCCGGTTGGTTATTCCAATATATGAGAAGGGAAAAGAGTTTGGACTTGGATATTAGCATGTGCATGGTTTTGGTTATGTTGGCCACGATTTACGTGGATGAGTGAAGCGGCTATTACAGGCACATGTATGCATCCACGGGTAAGTTTAGTGTTGCACCTTTGGGGAACTGTATTCGTACCTTTTGTGTTGGTTATTTTTTTACGTGGGGTTACGGTTGTTTGCAATCTCGTGAGTGTTTCAGAACTATAGTGTGCCATTCATAGTTTTCATGGGCGTTTGCATATGGGCTTAACATGTTCCATATAGGGGTAGTTAACAAATTATAAGTCATTTGATACTTATGGGTAAATACTTTTCCCACATTTTGGTTAGGGATCCCGCGTAGTGATACCGAGTTTCTTGTGAAATTTTCTCATGGAGCTGTAGGCGTGGGTTTTTTTGTTAGCATTAGAAGCTGTAGAACGGTGTGTTAAGCTTC--GGAATCTTGAGCATTTGGTGCCCCAAGCCAACCAGAAGGTCCTTGTTTAAATGGTTCAGAAGTCTCCCATATGGTAATACGGGTCAGATGATATATGTGTTTGTGTGTTCATGTATTAGTTTGGGGATATCTACAAGGTG------GACGTTCTGTAGGCGAACTTGATTATTGGCGGCTCCGCATTCTTGGTTTTGCATCCTCCTCATGCTTACTATGAGAGCTATCCCTTTTAGTTAAGTGTGGCACATGTATGTCTAGATATCGATATCAATTGA------TTGATCTAATCTAGCAGATCTT----------TTCGCTTTGAGTGAAAGCGTGCATCCATGATGGAGTCGGTTTGTACTCGGGAGTATCATCTGACGTCCTCATGGGTAAAGGAGCATCCGGCGTTAACACTGTCTATGGTCAATTGGTCTATAGGCATGTCGAATTGGGGCAACAAGTTATCCATAAGTGAGTCTACAAGATTTTATAATGTTTTAAGGGGTTCACCTTCACTGTAGCCAGCCTGCCCTTTTTAGGTTCACTTTCCTATGTGGTTTAGTTGATTC--AGATGGAGGTTTATGTTTGGGTTTAGGTATTTAGTTTTTGTGTTCGGAGTATCGGAAGACTACAGGTGCAACATCGGTATAAGGTTCGTTGGATGTATTTATATTCGAGTATTTTTTTTCATTTTTGGGTTTTTTTTCTTAACCGAGTGTTTGGAGTCTACCCCGTGGATCACCTTAATAATGGGTCCTTTTAAACATATTTCTTCAGGGGTACGTACTGTGCACAGGATAT----AGGGAAAACACAAGGTTTGGTTACATACGTGTTTGGGTTTTTGTATTTCATTGAGATATAGAAGTCTAACATGCGAAGGTTGGGTCTTTTTAATTCACGGTTCAGGATCAGTATTTGGACGTATTTATGTAGTTGTCGTTACATAACATTAGAGATCAAATTGGTTGAATTCGCTGTATGCATTGTAGGTGT------TGTAGTTCGT----TGTATTTGAATAGGTCTTTTTTATTGTTGTATCAATGTAGGGATTTCTTCATCATAGAGCTCATCTAAGCTTTCAGGTGTTCATGTAAGAGTAATGGGTGCTATCGATGTCAGCAAGGGCTCTTTTTTATGCATTAATCAATGGCGGGTTTATAGGAGTACGATGT--GGGTTGTTGTGGGTGTTTTTTTTTCTATGCACGCTTTTGGTAGTTTCCTGGGCAATTCTATGGGGCACGTATACAATTCTTCGCATCCTATTGGGAGCATACGGTGTTTGGGGGTTTCGTGGAACCACTTGTCGTTTTTCTTCATTGTTTCCTTGTATTGTTTTGTTTTTCATTGTTTCTCATGTTGGAGTCAACCTTGGTTTCAAAATCGAATATTATTCACCTATTAGGTAAGAGTCTGGTTGTGTGCTTTTATGTTGGTGTGTCTCTTTCCGATTTTATGACCGAAATTATGAGCAACCATAAACCCCCCAATCACCGATGTATTCTTTGCTATCATCTCGTCATCCAAAATTGGGGGTGTTCTTTTGCAGGTATATTTGCTTCTCCCTTA----GGAAGGTTAGTTCGTTATGGCTAAAAATTTTATGTTCAGTATGTTTTTATTTACGTTTGGTCATCCGTGATACCTAACGCATAGCAGTATACTTATTATTGTAATTATTTCTGTCAATTTTTACATATTCATTTATGCGTGCTTTTTCCTTTGACGAAATTTAGATTCCAAACGGAGGCCAAAAGTGTCTAAGGTTTCCACCATGGGAGCGTAACTTTTCAATCACAGTTCC--AATTGGTTTATCGTTAACTTGGCCCTGCCTCTAGATAAACTTTTTGATACACCC----TTCTTCAATTATCATTAACATCGGATGTATCTCTATCTAGGTACGCCTCCACATTAAGGGTGTTTCAATCACTTAGACATGGGCATCGTCGTGCCATCCTTCTTGAATACTTAGTTAGCTTTATAGTTCTATATCATCTTTGTTGAAACTCCAATTCAAGTGAGTG--CTTTTGTGATCCTGATTCCTGTACTTATTGTTTTGCGAAGGGCGAGCCTTGACTCCGAGGGAAGGATTGTAGGGTAACATGATTGGGGTTCATAACTTATTTCTGGGAAACTGCGTATTTATACATATTTTCACACTACTGG--GGTTTCTTTGAAGCAGACCTGTTTATAT--------GTATATCGAGTCTACCCGATCGTAGACACTATCATTAATGGTGGATTTCCCACTATTCTCGTTGTTGTCTGTTTTCATATTTTTTTTT----------------GTCTTCGTTTATTCTCATTCGAACAGTTGAACAAGATCTTGATGGGTTTTACGGAGGAATCATTCCTTTGGTCATTTTATGTGGATCTTTGTGTTTGAATGATTGTATTCCTCCCTATGTGC----AACTAGGACCGTTGTTTTTG--TGTTGTTAAGATATTTTTATGGATCTTTGAGTACTGGGTCATGATGGGATATATGCCTTTAGGTGATTCTTCGTAGCATTTAGCGTCTTTCTGTAACCAGTCTTAATTAAAGTGTTGTTTCTTCATCTGTTTGAGGAGTTGGTTACATCTGTTCACAGCTAAATGATCACATATTGGAGTGGTGGTATGTGTGAGGTTAGTCTTTTTTTTACGTTTCTGTAGTTATGTTTAATAAGGTCATATTTCTATTGAGGTTAAAATTATATACCTTTTTTTGTATTCATTGATCTTGTTAGCCAGATTTTGGTATGTGGTGGAGGTTGGTTACTCTTGGTTATATTTTACAAATGAGAGTTAGAGGGTTTTACTTTTATAACGATGGGAATTTATATTCGTATGTATCTTCAG--------TGAAATTTAAAGGTTCATAG----------TCGCGTCAATTCGCCTTTCTTTGGGCATACAAAGGCCATTCCTTTCTCTGCTCCCTGCATGCGCCCACCCGTAGAGTTGTCATCTCACTTGTACGCGGATTATTCTATCGTTGAAG--TTTTCCTCGAGTTTCAGC----CTAAGTGTTCATATACATGTTAGCGGGTTCGCCTTGGAGTGATTAAAAGTGTGCCTTCACCTACCATTGGATATACCTTAGTTCATGGAGGTACGCGCACACCATTGTTTCAGCTTTTAAAGGCTTTTATCTTCTCATGCGGCTCCTTTTACGGTTTATCAAGAGT----------ATGTATTTGCGAGATCTTCTACCTGTTCTTTTTTGGGTATTTACTCGGTATCGTCGTTATGTCTATTTAATC--------------CCAATTATTCGAAAAAGATTACCAGTAGGAGGTTGTAGGCAGGTCAGGTTATATAGGTCTGGGGAGATTTCTTG----GTGTATCCATACATGTAT----GGTAAATTAGTGAGGTTTAAAACTTTATATGTTCCTGCGGGGCTATGCGCTTTGTCTT--TATTTGATGCTC--AGTGAGATGTGTCTTCTTTCGTCAATGTATTAAGGTATTTGCATAGGTACCATATGATGTATTTAGATATGGTTTTGGGGTTATGTTTCTTCTTATCTATATCTCC--AACCGATTGGGTTTAAAATGAA--------------------TAGTGGTTTCATCTATTTTTATCTACAGATTA----------------------------------------CTCCGTGTGAAAAAGACC------------GACTTTCCAT--TCCTCATTTAGCTAGGCTTGTTTTTTTTCTTTGTTTCTATATGTGGATATAATAGGTAAATCATTACT

Pthirus_pubis ATTCATTTGACCAGATATTTGTACGG--TTCCGT----AATGCTGGGCAC--------------TTGTTGCTTAGCGGGGGATTTGCTGTAATCGGTACGAGGCGTACAAGTTGTTTTCTCAGATTCA----------------------TTATCCTTTTGGGTGTAGTTTATGGTTTAGATAAGTATTCTTATCCTTACTTCCTTACTCCAATTCGTAATTGGATTGTTACCTTGTTAGGGTTCTTATC--TTGGAGTCCTAGAATCTTGCCATATTCCTTGGAGCCATGTCTGGCCTTCTGTCTATGAGTGCAGGTCTATCGCCATAGCTAGGTCGCTATGCAAATTTGGGGCAATATATAGCTATGAGAAGTCTTGG--AGTTATAGGGATTTACTTCTACTATCCTATGTTGGTGAATTTGTGCACGTCAGCTAGTCTAGAACTTTAGATTATGGACTTCCCTTCTTGTTAAATTTATTTATGTTTGTTTATGTATTTTC--GGTCTATTTCATTCACGTGAGTAATTCGTAGAGCCG----GTATTTACTTGAAATGATCTTTTCACAACAAAGAATGGCTTTTACTCTTCGGATTGTTGGTTGTGGTTTCATAGCTATGTCGGTGACTTCTCACAGTCTGTTGTAAAGCAACTAAAGTTTGTACTCCAGCTTGTATATTTTTATGTATCCGTATATGGGGTTGCAATGCTGTCCTTTTTTGGGCCCGAATGCTTCCCGATAAAAATAGTATGCTATATCCTCGGGTCTTTATGCAGTCATATCAGGGGACGGACGGTGACATTACCCCCTAGCCTTGAGGCACCTCTTTCGTGATTACATTTAGCTCATTGCGGGTAGTCATTTGGTCGTAATTATAGACATTTAAATTGCCTCAGTTAAATTACGTTCCTTTTTGTGTCGTTTATACGCTTTTCTCTCTTCTTCCGTCTGCGGGCATACATCTCTTTGACGAATTAATGTCTTTTGACCCTGGGGGGGACCGTCTTACACACTTTTGTTTTGGCACCGAGTTAATTTATTTCCGGTTGGTTATTCCAATGTGTGATTAGGGAAAAGAGTTTGGTCTTGGATATTAGCATGTTCATGGGTCTGGTTGTGTTGGCCACAATTTACGTGGCTGAGTGAAGCGGCTATTACAGGCACATACATGCATCCACGGGTAAGTTTAGTGTTGGACCTTTGGCCAACTCATTAGGTAGTTCATGTCCTGGTTATTTTTTTACGTGGGGCTACGGATATCTTCAATCTCGTGAGTCTCTCAGAACTATAGTGTGCCATTCATAGTTTTCATGGGCGTTTGCATTTGGGCTGAACATGTTTCTCTTAGGGTGTACTAACCAAGTATTCACCATTTGGTAGTTATGGGTAATTACTTTTCCCACATTCTGGCTAGGGATCCCGCGTATCGATACCGAGCTTTAGGTGAAAAATTCTCCTGGAGATCTACTTGTGGGTCTCTTTCTTAGCTCTTGAAGGTGCAGGCAAAAGTCTTTGAAAGTAGGTCAGACTGTGGCTGTGGAACCTCGGTTCAACTTACACAGTTCACTTCGATTACTTCAGAGGAGTCCCGTATGCTTGTTCAAACTAGACTGTTGGTGTTGGTGGGTATGCTTGTGCTAGTTCCTTTTTCTTTAAAATCTG------AATATATTGTGGCTGAAGTTGAACATTGGTATTTCCTCTTGCCTGCTCTTGTTTCCTCTTCATGTTTATTATGAGAATTAACCCTCGTACCTAAGTATGGCACATGTATGTCTAGATAGGGATGGAAGATGA------TTAATCTAATTTAGGAGGGATTAAAAGTGATCTTCGCTTTGATCGATGAGGTTAATCCTCAGACGAATCGGCATATACTCTCGAGTATCATCTGGCATCCAGCTAGGTAAATGAGCGTCCGGCGCTAACATCGTATTATCCAAACTGGAGCTTAGGCATGTCGAATTGGGGCTACAAGTTATCCATTCGTAAACGTCCAAGCGTTTATGCTGGTAAGAAGAATTCACCTTCACTGTGATTAGCCTGCCTTTATTAGCTTCTCTTTGTTGCGTGCGTCTTGATTTGGTCTTAAGGTT----TTGCTTTCGTATGTTTGTCTGTCTTCTTTGTGCGGAGTATCGGATCACTTCAGGAACAACATGAGTAACGGGATCGTTGGATATTTTTATATTCGAGTATTTTTTTTCTTTTTAGGCTTTTTCTGCCTAACCGAGTACTTGGGGCGTTCCCCGTGGCTGCTCATGGCTCTGGGTCCCTTTAATCTTTTCTTTTCAGGGGTAGGTACTGTCCATAGAATCT----CGGGGAGTCCAATCTTCTGCTTGCATACCTGCCTGGACGTTTTTGCTTCATACTGATAAACTAGAGTTAGATTCGAAGGCTAGGTCATTTTACTATACGGTTCAGGTTCAGTATGTGGGTGTTTATTTGTATACGTCGTTGCAAGGCATTAATCAGCACAACGGTTGACTTCGCTGTATGCATTGTGAGTGTTGTTCTTTTTGTTCCTTATGTGTATCTGAATTGGTCCTTTGGTGTTTTGTGTCAGTTTACGGTTTTTTTCACCATAGAGCTCTTTC--TCTTGAAGGTCTTTATGATTGAGCTTGAGGTGTTGTCGAGTTCAGCAAGGGCTCTGTTTTATCTGCTATTCAATTGCGAGCTTGTTGGTGTTTCCAAA--CTGTTGGTTCGGATCTATTTTTCTATATGCATTCTTTTGGTAGTCTCCTGGGCAATTCTTTGGGGCACGTATACAACTTTAGGCCTCCTTGTGGTCGATTGTACTGATTGGGGGTTTCGTGGTCCCACCTGACGTTTTAGTCCATTATCTAGATGTTTCTTGTTGTATTTCAATACTTCTCAGAAAGGTCAGAACCTTGGTTGACTCATCGAAAGTTATTTACCTATTATCTAAGATTCTGGGGTTGTGCATACATTATTTCCTGGCTTATCCCGACTTTATGACCGAAATTATGAGCAACCTTGTACCCCCCAATCACCGATGTATTCTTTGCTAGCATTTCGGCGTCCAGAACTGGGGGTGTGCTTGTATAGATGTTCTTGTGTTTCTCT------GGAAAGTATCGCCGTTAGGTGCCGAAGTCTGTTATCCTGTGTAGGTCTATCTTCTGCTGGGCATCCGCGAGTCCTTGTAAGTAGCAGTGCAGGTATTATTACCTATTTCTATTTCAGGTTATACGTGTATTTATGTTGGTGCTATTTCTTTTGACGAAATCTGGCTACCACTCGCTGGCCAAAAGTGGCCACGGGTCTCACCTTAGGAGCCCAACTCTACAATCATCAGCCA--ATGA----CCTTATTAGCTTGTACTTCTTCTTTTCTTCTTACTATGTTAGATCCATAATTACCCTGAGTGGAAGGAAAATGGATTGATATATGGTTAGCTGTGTTAGGCCGTTCTGGGTGTTTCCATCAATTTCGTTTGGAGACCGGCATGCCATGATTCTTGACTGTCTTCGTATTTCTTCGTTTAGTTTCTCTGACTCGTCTGGATTGATGCCACTTAGCCCAATTCTACGCCC--CTCTGCTTGGGTTGATTCCTCTGCGAAGGGCGAGCCTTGATTCCGAGGGAAGGATTGTAGGGTTAAGTGATAGGGGGTCGTAATTTATTACTAGGAAGGTACCTATGTTCTGATATATAAAGTTTTTTGG--GTACTATTCTCCATCTATTGGTGTATAG--------ATCTCTCGTCAGTTCCCGATCGTTGACATGATGCCTGGTGGTACTTTTTCTCGCATTCTAGCTTTTGTGTGTGTGCCTGTGCGTTTGC----------------TTTTTCGTACCTTCTCCAGAGTGA--GGTAGACATCCCTTGATGGGGTATCCTATCACTCTATCCCTTTAGTCATTTATGATAGGTATTTTTGTTTGAGTGAATGTATTTTTCCATGTGAGC----TCTTCCGAGTAGGGGTGTTT--TGTTCTACTTCTCTTTTTATGGTTTTATGAATGGTAGGTCTTGATGAATTATTCTCTTATTCATGAAGTGTGTGTGTCTATTGCCTATTTACGTGGCT--GAGTGAAGCTATATTCGAATTTTCGTATGCTGGAAGGTATGGCTACCTAGTCAACTTCAGAGAGTGTATCTTCTCTGATTGAATGTTTATTTGACGGTTCGTATTTTTATATGTTTGTGTAGGCGTGTATGTTTGGAGTTTAATACTCTCTTCAGGGTGCTTACTTAGATTTATTTATGCAGATGTATTTTCGTACGGTCTTTTTGTTTTTTGGTGGAGGTTGGTTTCAGTTATTTATATTTAAAAAATGTCTCTCAATCGGTTATACTTTTATAACGTTGGGAGTTTATATGTGCTCTCTGTTCTATCAGGTC----------TTGGTCGGCTTC--------------GCCTGGAGGTGTTTACATGGTGTTTCAAAGGCCATTCCCTTTAGTGTTCCGAGCATGCGCCCACCCGTAGAGCTGTCATCTCACCTGTACGCGGATTAACCTGTCGTTGGAG--GAGTAGAATGGGTC--------ATGTGCTTTCTTTTTCTTGTGTTCGGCTTCGCCTGTTCACGATTAAAAGTGTGCTATCACCTTCCAATAGCTATATCATACTTCGAGGTGATGAGCTGATCTCAATGTATCAGCATTTAAAGCTTTGTTCTTATTCATGCGGCTCCTATTAGGGGGTTTCAAGAGT--GT------TTATCAAGCTAATTGGTTTTAGGCAGTTTTCTGTTCGTTTTTACAGTCTATCGTCGATATTGATTTTTAGCC--------------TCAATTGGTTAA------TTGGTTAAACGCCTTTTCTTTCTTAGCTGGGTTACTAAATATGTGTTTTGGCGTTG------ACCCTTTGATGGTTAT----GCCAGATCAGGTTCGTCTAGAAACCTATATAGGTGCTCCTGGTTTTAGTTGTTGTTGT--TAATAGTTACAA--AGTTGTATATTTTTGAATGCTTATGGGTATATAAGTCTCTTCTTTAGTACTCAATGCCTGCTTTCTCTCTGGGTGTGGGGTTATGTGTATTCGTATCTGTGTATCC--------GGCCAGGCTTCATGAGGTGAAA--------------GGTCACGTACTTGTCTGTCTATTTGCGTGCAT----------------AGTA------TTGAGATTGAAGTTTTAGCTAC--TAAGACTG------------CGTTAAATCT--TCAATCTCTTTTTACCGAGGGTTTGTCTCTATGGCTCTTTATCTCCGTATGAGTTTCTTCTTTAGGCG

Polyplax_asiatica ATTCATTTGACCAGCTGGGTGAGG----CTTCGT----AATGATTGGGTT--------------GCCCTTTTCTTTAGGGGGTTTGGCTTTTTCGGGTACGGGTCTTCATTTGGAACAGTTCAATCGTCTCCTCCTAAGAGGAA------CCTTGCTTAGCTATGGGTTAATGTCTCTACATAATTTTAGTTAGCCTTTCTTTGCCCCTCCATTAGTTGGCTAGGTTGTTCCCTTGGTGGGGTTATTCTC--TAAAAAAGAAGAAATTCTTCCATTCTCCCTGGACCCCAGGTTAGTGTTTTGTCTATGATTACAGCAGCGTCGCCTTAGCTAGGTCGCTATGCAACTACGCGGCAATATATAGCTGCGAAAGCTCTTCTTTCCTTTTTTAACTCATTTTATATCTTCCTTTCTGTTTGATTGGGTGCTTATCAGCTAGTTTATAGCTCTTCCTTATGGAATGCCCACTATGGTATTTCTTTTTATCAACATTTCTATGGAAAT--GGTTTATTTTTTGTAGAGCTGCGAGGCAGGGTAATCCAAATCTTGTAACAGATTTTCTTTTATCACAACAAAGAATGGGTTTTATTGTTTAGGCTGGCGGTTCTGGTAGGCTAGTTTTATCGTTGACTTGCACCGGAATTATGAGAGGCAGTTTAAAGTTGTACGCCAGCTTGTATATTTTTATGTATCCGCATGTGGGGTTGGAATGCTATCCTTATATGGTTCCGAATGCTTCCCGATAAAAATAGTTTGCTCTCCCCTCCTTTCTTTCTAGAGGCTTATCAGGGGGTGGACGGTGACGTTACCCCCTAGAGAGGTGGCACCGGGCTCGTGATTGCATTTAGCTCACTGCGGGTAGTCATCTGGGCATAATTATTCACATATGTGGTGAATTTC--CAATGAAACTCCTTTTTGTGGCATTTATACGCTTCTTTTTCTTCCTCCGTTTGCGGGCATACATCTTTTTGACGAATTAATGTCTTTTGACCTCGGGGGGGACCGTTTTACACATTTTTGTTTTGGCACCGAGTTAATTTATTTCCGGTTGGCTATTCCAATATTCGAGAATGGAAAAGAGTTTGGAGTTGGATATTAGCATAGGCATGGGCATGGTTGTGTTGGCCACAATTTACATGGATGAGTGAAGCGGCTATTACAGGCACATATATGCATCCACGGATAAGTTTAGTGCTGCACATATGGGGAAGCCCCGAGGTTCATCTTGGTGCGGTTATTTCTTTACGTGGGGCTACGGTTGTTTGCAATCTCATGAGTGCCTCAGAACTATAGTGTGCCATTCATAGTCTTCATGGGCGTTTGCATTTGGAGTTAACATGTTCCCTCTAGGGGTGTTTCCCAAATGATAAGCCATTTGGCACTTCTGGGTAATTACTTTTCCCACATTTTGGCTAGGGATCCCGCGTAAGGATACCGATCTTAGTGTGAAACCTTCTCGCGGAGTCATACTCGTAGATTTGTTTATGCTGCTTGGAGGCACTTCTGCGGTTCCTTGCAGGGC--TGGCAGCTGATGTTAGGGTTCCCCTCGACATCCAGAAGCCCCCAATTTCTGTAGCTCAGATCAATCCCTTATGGCAATGAAGGTCAGATGCTATGTGTCTGCGGATATTCGTGTATTAGTAGATTGACTTTTAGAAGATG------AAGTTTTTTTGAAGGATGTTGAGTATTGATATTTCCTCATGTCTCTACTTGCTTCCTCCTCATGCTTATTCTGAGAGTAGCTCCAATCACATAAGCGTGGCACATGTATGAGTAGATTGTTCCCACAGCTGAACTTTTTTGATCTACTTTCC--AAGATGGAACGGGCGCCCCGCTTTGATGGATCTCATCTCTCCGTGGGAGAACCGTTGTGTAGAGGGGAGTATCATCTGGCCTCCAGATGGGTAAGTGAGCATCCGGCGTTAACAGTATCTTACCTTAAAGGGATTCTTGGCATGTCGAATTGGGGCAACAAGTTATCCATAAGTGAGCATCCCGGAGATGATTCATCTAATTAGAATTCACCTTCAATGTAGGTAGCCTGCCTTCTTCAGGCTCGGTTACCTGCGTGGTTGTGAGCTTTC--GGATGGTGTTTTGTGGTGGGAGCTAGAGGGGCTTGCGGCTTGTGCGGAGCATCGGATCTTTTCTGGGACAACATGAGTACCGGGTTCGGTGGGTATCTTTATCTTCGAGTATTTTTTTTCATTTTTGCTTTTTTTTTCTTAACCGAGTAGCTGGGGCATGCCCCGAGGCTTCCCGTCCTAATGGGTCCTTATAAACGTCTCTTTTCAGGGATTCTTACTGTCCACAGCCTAT----GGGGATGCTTCGTCGCTTCCCTCTATAGGTCTTTGGGGGGTTTTCTCTCAGCGAGATAAGGATGTCTTTCATTCGAAGAGTTGGTCTTTTTTGTAGACGGTTCAGGGTCAGTATATGGACGTTTCTTCGTAACTGTCGGTATTCGCCATTTCCCCACACACTGGTTGAGCGGGCTGTATGCATTGTGAGTGTTGCTTTTTTTATACATTATGTGTTATTGAATAGGTCCTCTGGTTTGTTGGTTCACTGTACGGTTTTCTGCATCATTTCGCGCCAGGCTTCTTGAAGGTTTAGATATAAGAGTAAGGGGTGGCATCGCTCTCAGCAAGGGCTCTTTTTTATCTATTACTCACTGGCGGGCTTATAGGAGTACGCTAT--GGGTTGATGTGGGTATATGTTTACTTATGGACGCTTTTGGTAGTCTCCTGGGCAATTCTTTGGGGCACGTATACAATTGTTCGCATCCTATTGGGATCCTGTACTGATTGGGGGTTAGGTGGAACCACTTACCGATTTTCCTCATTATCTCCTTATATGTTTCTGCCTTCCACTTTCTCTCAGAAGGGAGTGAACCCTGGTTAGGAGATCGAAAGTGCTTCACCTATTATTCAAGATTTTGGGTTCGTTTGGAGTTACTTGCTCCTTTCTTTCCGATTTTATGACCGAAATTACCCGCAACCTTGCACCCGCCAATAACCGATGTATTCTTTGCTATCATCTCGTCATCCAGAACTGGGGGTGCGCTTGTTTAGATTTGTTTGCTTCTCCTT------ACAATCTCAGGGCGTTTCTTCTCATCTTACTTTGTTCATTAAAATTTTATTTACTGCTGGGGGCGCGTGACTCCTTACCTGTAGAATTATACTTTCTATTTCGTTTATAGTGCTCCTTCTATTCTTCTGCGTTTATATGTGCTTTTACCTTTGACGAATTCTGGATCTCAACCGAGGGCCAGAAGTGGTTTGGGCTTTCACCTTAGGAATTTAACTGTACAAACACCCAGCC--GGAAGGTTCCGCGGTACTTTGGCCTTGGATCTATCTAGGGTTGTTTGCTTTTCCTTAATTTCAAGA------AAGATTAGGCGTACGTTTATGTCTAGGTTCAGTTCCCTATATAGGGTGTTTCCGAGAATATCGTCTGGAGCTCGAGGTTCCACAATTCTTGAATCCCTTCACTCATATTCATGTATTTAACAACAGAGATCA--GAATTTATCAAGGGGTTGGTTCTTTTTGCC--ACCTCCGTCTATACTTAGCTAGGCGAGCTCCGTTCCTTGACTCCGAAGGAAGGATTGTAGGGTATCATGATAGGGGGCATTTACATATTTTTGCGAGCTGTCCTTTCTCTAGGGCTTTTCTCCTCTTCGG--AGATAGCCCCCTTCCTTCCTCTTTTTGC--------GTTGGCCGGGGTCTCCCGGTCGTAGACTATATGAGTTGTGGTGACTGTCCATGCCTTCACCTTGATGCAGTTCCGGTTGTATTTCTCTTCTTGTGCTTGCATGTTTGTGCTGATTTTCGAAAAAATTATCAACAATTTGAGCTTGATGGGTTAGCCATGGCACCCAGGCCCTAGGTCATTCTCTGTGGATTTTTCTATTTGAATGAGTATATATCTCCGTTTTTTT----AAGTAATTCTCATGGTTTTT--TGACTTTATTATATATCTTGGGTTCTGTGAGTGATTGGACATAGTGGGGCATACGCTTGTAGGCGAATATATAAGTAGTATGTTGGACATAGTTGAGGACGGGTCAGGTTATTGTTACATCTGTTTTCGTGCGAAGGTATGGCTACACCTAGACTCTTATAGAGGCCATTGCCCTCGACCTATTGGTTATCTGATGATAGTTCTTTCTTTATGTTTGGGTAGAGAGGTTTCTTATCAAGGTATTAAAAGAGACA--AAAATTTTCCATCTAGTCTTGTGTTCATTTGTTTGTTCAGAAGGTTTTATGCTTGTGGTGGACTCTGGATAGTCTTTGTTATTTTATTAATCTGTCTCTAAAGGGGCTGTACTTTTAGAACGTTGGGACTCTTTTTAGAGCTGGTTCTCTGTGGGGAGGATGAGTTCCCTAGTCCCGGCC----------------TGGGAGGGCTATCTTTGGGCATACAAAGGCCATACCTTTCGCTGCTCCTTGCATGCGCCCACCCGTAGAGTTGTCATCTCACTTGTACGCGGCTTTATGTGTCGCAAGCCTCACTTCCTCGTTCTGTTGGGT------TTGTTCTTGCAGGTATTAGCTGTCAGGCTTATGAGTGATTAAAAATATGCTTTCACTTTCCATTGGCTATGTCTTTGTTCATGGAGATGAGCGCCTGTCAATCTAGCAGCAGTTAAAGATACTTTCTTACTCATGCGGTTCCTTCTAGGGTTATTCAAGAAA--CTTTTG--AATTAATCCATGGAGGCTTTCCGCCTACTTTCCTGGACGTGGACGCGCTAAGCTCGATCTCTTTTTCGAATCCA------------GCTTAGCCTTTCTCAATCTTGGGATCGAAATGATTCCGGGATATCTGGCTATATAGTTTCCTTGATTTCTCCTG----------GGATAGGAGAAA----AGTCCCCTTTATGAAGAGACAATCTCGTTTATTTATCTATGGATTTTTGTTTTTTCAT----CATGATACGA--AGGTGTACAGAGATTTATATCTTTTCTTGGATTCTGGAGTTTAACTAGAGTCGTTGCCTCATTTTTTTCTGGATTTGGGGCTGTGTCTCTGCTATCTTATCTTTTC--TG--AAGACAGGAAGCTTTCAAGGTTGA--------------TTAGGGCTCAATGTGTTATCCCCTGCTTATCT----------TGATCTGAAAGATCCTACCTATGGGGTCAGAGAATTCACCTCGCTGAG------------GTGCTCATAG--TTGATAAGAACATAATTATCATCTTTTTTTAGACTTTTATATTTTTTCATGAGAATATAAAATTAAAA

Polyplax_spinulosa ATACATTTGACCTCTC----TTAATCCTGTCCTT----AATGGGAGAGGT--------------TTTTCTGTATTTAGGGGGTTTGGTGTAGACGGTTAATTCTGTGAATTTTTTTAACTTGCAGGTTAAGTATTTCTAATGAA------CACATCGCATTTATGGCTTTTATTATTTACATAAGTGTGGCTTTCCTTTCTTTCGTACGCCATTTCTGAGTTACATTGCTCCATTGTTGGGGGCATTAAT--TTTCAAGAAGGAGGGCTTGCCATTCTCCCAAGGCCCATGGTTGCCCTTTTGTATGTGAATGTAGATCTATCGCCTTAGTTAGGTCGCTATGCAAATACGCGGCAATATTTAGTTATGAACCTATGTAG--AATCCTTCGTAATGTTACCTTTATGCGGTTCTCTTTGATTGGGTGCTTATCAGCTAGTCTATAACTTTTCCTTATGGAATGCCCATATTGTTTTTTAGTTATACAATTATTTCTTTATATGT--TTGTACTTGAAATTTTTTTGTACCGAGGGAAAAATATCCCAATAAAACTGAGAAATTTATTTTCACAACAAAGAATGGGTTTTATTCTTTGGTTTGGCGGTTGTGGTTGGATAGGTATATCGATGACTTCCACCGGTTTGATGAAGGGCAGTTTAATGTTGTACTCCAGCTTGTATATTTTTATGTATCCATATATGGGGTTGCAATGCTGTCCCTATCTAGGCCCGAATGCTTCCCGATAAAAATAGTTCACTCTCCCCTCTTGTCTCTTTTTAGTCCTATCAGGGGGTGGACGGTGACGTTACCCCCTGCAGGGTTGGCACCAGATTCGTGACTGCATTTAGCTCATTGCGGGTAGTCATATGGGCATAATTATTGACGTATAATTTGAACA----ACATGAAGTTCCCTTTTGTGAGGTCTATACGCGTCTTTCTCTTCTTCCGTTTGCGGGCATACATCTCTTTGACGAAGTAAAGTCTTTTGACCTCGGGGGGGACCATCTTACACACTTTTGTTTTGGCACCGAGTTAATTTATCTCCGGTTGGCTATTCCAATATGTGAGAAGGGAAAAGAATTTGGAGCTGGATATTAGCATTCTCATGGGTATGGTTGTGTTGGCCACAATTTACGTGGCTGAGTGAAGCGGCTATTACAGGCACATATATGCATCCACGGATAAGTTTAGTGTTGCACCTTTGGGGTCTCTTCAAGGTTCGGCTTGAGATGGTTATTTCTTTACGTGGGGTTACGGTTGTCTTCAATCTCATGAGTGCCTCAGAACTATAGTGTGCCATTCATAGTCTTCATGGGCGTTTGCATCTGGAGCTAACATGTTCCTTGTAGGGTTGTCTAACAAATGATGCATCATTTGGTACTTCTAGGTAATTACTTTTCCCACATTTTGGTTTCGGATCCCGCGTAATGATACCGATCTAAGGCTGAACTACTCTCATGGAGTCCTTCATGTAGGTGGATTTATGGTCTTCTGAGGTTATGCAACGTTCTTTTTCCTTTA--CAAGAGGTGAAAATTTGGACCCCCAGTTCAAGAAGAAGGCCCGCTATCATGTAGCTCAGATCAATCCCTTATGGCAATGAAGGTCAGATGCTATGTGTCTGCGGATATTCGTGTATTAGTAGATTGACTTTTAGAAGATG------AAGTTTTTTTGAAGGATGTTGAGTATTGATATTTCCTCATGTCTCTACTTGCTTCCTCCTCATGCTTATTCTGAGAGTAGCTCCAATCACATAAGCGTGGCACATGTATGAGTAGATTGTTCCCACAGCTGAACTTTTTTGATCTACTTTCC--AAGATGGAACGGGCGCCCCGCTTTGATGGATCTCATCTCTCCGTGGGAGAACCGTTGTGTAGAGGGGAGTATCATCTGGCCTCCAGATGGGTAAGTGAGCATCCGGCGTTAACAGTATCTTACCTTAAAGGGATTCTTGGCATGTCGAATTGGGGCAACAAGTTATCCATAAGTGAGCATCCCGGAGATGATTCATCTAAAAACAATTCACCTTCAATTTGAAAAGCCTGCCGTATATAGTTAAATTTAGGTGCTCCTCTTCAGTCTTTT--TGGGGTTCTTCTTTATGTCCGTCTGGCTTTTTTCGTTCTTTGTGCGGAGTGTGCGAAGCTTTCAGGAACAACAAGAGTGTAGGGTTCGGCGGGTTTATTTATTTTCGAGTATTTTTTTTCATTTTTGCTTTTTTATTCCTTCCCGAGTAGGTGGATGATACCCCGTGGGTGGTCTTAAATTTAGGTCCTTTTAAACATATTTCTTCAGGGGTTCCTACTGAGCACATCTTCT----GAAAAATTTTAATCAACTGGCTTTATTCATTCTTGGTCTGTTCTTTCTCAAAAAGATACTGATGCCTTGAATTCGAAGGTTTGGTCTTTTTTATGGACGGTTCAGGCTCAGTTTATGGACATTTCTCTATAGTTATCGTCATATGGCATTTCCCTGCATGTTGGTTGAGCAGGCTGTATGCATTGTGAGTGTTGCTTTCTTTGTACGTTATGTGTACATGAATAGGTCCTCTGGTTATTTTTATCACTTTAGGGTTTTTTGCTTCATAGAGGTCTCATTTTCTTAAAGGTATTCTAATCAAGGTGAGGGGTGGCATCGTTGTCAGCAAGGGCTCTTTTTTGTCTATTATTCAATGGCGGGATTATAGGAGTAAAAACT--CTGTTGGTACGGGTATATATTTATTTATGGACGCTTCTGGTAGTTTCCTGGGCAATTCTTTGGGGCACGTATACAACTCTTCGCTTCCTAATGGGAAGCTGTTATGTTTGGGGGTTAGGTGGAGCCACTTACCGATTTTCATCATTCTATCCTTATTTCTGTGTGCCTTCCAATGCTTCTCAGAAAGGAGTCAACCTTGGCTAGCCCATCCAAAGTGCTTCACCTATTGTGTAAGAGTGTGGCTGTGTGTGGTTCTTTGGGCATATTTCTAGCCGATTCTATGACCGAAATCATGAGCAACCATGTACCCCCCAATCACCGATGTATTTTTTGCTAACATCTCGTCGTCCAGAATTGGGGGTATGCATATATAGATTTATTTTTATTTCCTT------TCGAGCTCTCGGCGTTAGCTATCGAGGTAGACTGATCAATAAAATTTTCTCTACTGCTGGAGATCCGTGAGCCCTTGAATACAGAATAGTACTGTTTATTCTACTTCTTTTGTTGCGATTATTTTTGTTCTTCTCTATGTGCTTTAACTTTTGACGAAGTATGGCTTCCAAGCGCTGGCCTCAAGTCTTTAAGGGTGGCACCTTAGGAGTATAACTTTTCAAATTCCTTCCG--AGAAGAGAGATTTGTAACCTGCCCTGATATATATGTTCGTAGGTTTGGGCTTCCTTTATTTTCCTC------TACAACTCGGGTATATATTTTTTTAGGTAGGCTTTGCTACTTTCGGTGTTTCAATCTCTATCACTTGGGCTGCGAGGTTCCATCTTTCTTGAATCCTTGCCTTGTTATTCCTTTCTATTCAAAGCTAGGTCG--GATGGG--CACTGATTACGTCTCTGTGCCC--TGAGGGTTATCTTTTTTCTTATGCGAGCGGCGAGCCTTGACTCCGAAGGAAGGATTGTAGGGTTAAGTGATAGGGGTTCTTTACCTATTTCTAGGAACCTATATCTGTATAGAGATTTTCATATTTCTCA--CATGGAGGTGCGTTGTTTTCTTTTATTT--------ACCTATCGCCTCGTCCCGATCGTTGACAGCATACGCGCTGCTTCATACCCACGCATAGGCGTTTTTTTGTCTATGCTTTGACTTCTATTCCTCTAG--------GCGTGCCTCTTTCGAGAAAGGGAA--ATATTCTTGACATTGATGGGTTACCCTTCACCTCCGATGTTTTCATCATTTTCTATGGGTTTTTTTATTTGATTGAGTGTTCACTTCCGCATTTAT----CAGTTCATACCATGGTATTT--TGGTTTTATTATGTATCTGTGGCTGTTTGATTTATGGGACTTCTTGATATCTTCGTATGCGGTTGATTATTCTCTTATTTATGATTTTACCAAATTTTTCGGTGGTGTCTGTGCGTATTTGTACATGTGCACGAGGGTATGGTTAGATCTAGGCTCATATCGCTGAAGTCAGCTACGGGAGAGTTCTTTGTTGACAACAGGCGTTTTTTTATGTCTGTGTTCACAGGTACAATATCAGTATAAT--AAAGAAGATTCTAATTCTTCCTCTAGTTTTATCTTCATATTTTTTGTTAGACAATTATTGAGTTGTGGTGGAGGTTGGCTACTCTTTTTTATCTTATAAGAATGAATCAGACGGGGCTGTACTTTTGTAACGCTGGGATTTTCTCTTCTCATTTTTCTAGGTAGAGGG--ATTTTGATGGGGAGAAAAGA--------------AGTTTTGGATTTTTATCTGGGTTTGCAAAGGCCATTCCTATCTCTGCTCCCTGCATGCGCCCACCCGTAGAGTTGTCATCTCACTTGTACGCGGGTTTATATGTCGAGTATT--CATTAGGAGCACCTTTCT----ATAAATATTCTTTTACATTTTATCGGCTAGGCATGTGACAGATTAAAAATATGCTATCACCTTCCATTAGATATGTTTTTATTCATGGAGCTGAGCGCTTTGCAATTTATCAGCCTTTAAAGATTTGCTCATATTCATATGGATCCTTTTAGGGTTACTCAAGAAT--ATATAG--ATTCTTAGAATTGATCTTTGAAGTTTGGATATAGAGATTTTTTCTCGCTATCACCGATGTCTTTATAGCAAGGCCA----------CCAGGTTCTTAG----GGTTTCAACCAGACAAATTGTCTCCTTGGTTCTTAATTAACTCTAGGGACTTATTTCC------------ATCTTTCCGACTTGCCCCGCCCGAGTGAAGGGGTAAGTTTGTGTTTTCCTCTACGGATTTCTATTTGTACTT--TTCTGCTTGGGC--TCCC--CAGGGTGTAATTATGCATACTTTTTCGGTTTTATTTGCTAGCGATCGTTGCCGGTCATTTTTTTGGATGTGGGGTTGTGTCTATAGTTACTTATATTTCCAAGAAGTAAAGAAGTTAATT--AACTTTAA--------------CATCTGCTACATGCCCCTGG--ATTTTTTTCTTCTGGAACGGGAAGACTCTGGCAGGATCAAGCTCTCTCCTAGCCTCTC----GTTGAG------------ATAGGAAGCT--------ATCTTTTCCCTTTTTTGTTTATTTATTTTTTTATATCTCTGCGTGATCGTACAATT--ATGG

Haemapinus_apri ATTCGTTTGACCTGTCACTTCTAGTTAATTCCTT----AATGTTCTGTGT--------------TTGTGTTTAGTTAGGGCGTATGATCTTCTCGGTTCATGGTATGTTGGTAAAAGGCTATCAGGTTCGGATCTAAAAATAAA------CATTATCTATCTCAACTTTTTTTTATTTTCAGAATTATGGTTTCCCTTATTTACCTTCTCCATTGTTAAATTAGTTTGTTCCTTTGTTGGGGATTTTATC--TGTCAATGTGAAAAACTTGCCATTACCCGTGGAGCCGTACTTGCCCTTTTGTTTGTGAACGTAGTTGTATCGCCATAGTTAGGTCGTTATGCAAATACGCGGCAATGTATACCTGCGACAGGGCATTCGTGCTCTAGTGGAGTTTAGTTTTTGTATGTCTTTCTTTGATTGGGTGCCTATCAGCTAGTTTATAGCTATTCTTTATGGAATTCCCATTGTGTTCTCTGAGTTGTTTTGTAGTTATAGTGTGAG--TGGTTATGGATTTTGTGAAGTTGCTCCCAAGTTTATAAGTCCGTTTAAGAGGGATAGTTTCTTCACAACAAAGAATGGGTCTTATTATTTGGGTTGGCGGTTTTGGACAGATAGTTTTATCGGTGATTGGAGGTAGAGTTATTCAGGGCAGTTAAAGTTTGTACGCCAGCTTTTATATTTTTATATATCCGTATATGGGGTTGGAATGTTGTCCACATTTGGGCCCGAATGCTTCCCGATAAAAATAGTTTGTTTTCCCCTCCTTTTTTTATTCAGTTATGTGGGGGGGTGGACGGTGACGTTACCCCCTAGGGTTGTGGCACCAGAGTCGTGATTACATTTAGTTCATTGCGGTTAGTCATATGGGCATAATTATTGACATGTAAATTGGTTGGGAAAATTGACTTTCCTTTTTGTGTCGTTTATACGCGTCTCTTTCTTCTTCCGTTTGCGGGGATACATTTTTATGACGAAATAATGTCTTTTGACCTTGGGGGGGACCGTCTTACACACTTTTGTTTTGGCACCGAGTTAATCTATTTCCGGTTGGTTATTCCAATATATGAGAAGGGAAAAGAGTTTGGACTTGGATATTAGCATGTGCATGGTTTTGGTTATGTTGGCCACAATTTACGTGGATGAGTGAAGCGGCTATTACAGGCACATGTATGCATCCACGGGTAAGTTTAGTGTTGCACCTTTGGGGAACTGTATTCGTACTTTTTGTGTTGGTTATTTTTTTACGTGGGGTTACGGTTGTTTGCAATCTCGTGAGTGTTTCAGAACTATAGTGTGCCATTCATAGTTTTCATGGGCGTTTGCATATGGGCTTAACATGTTCCATATAGGGGTAGTTAACAAATTATAAGTCATTTGATACTTATGGGTAAACACTTTTCCCACATTTTGGTTAGGGATCCCGCGTAGTGATACCGAGTTTCTTGTGAAATTTTCTCATGGAGCTGTAGGCGTGGGTCTTTTTGTTAACATTAGAAGCTGTAGAACGGTATTTTAAGTTTC--GGAATCTTGAGCATTTGGTGCCCCAAGCCAACCAGAAGGTCCTTGTTTAAATGGTTCAGAAGTCTCCCATATGGTAATACGGGTCAGATGATATATGTGTTTGTGTGTTCATGTATTAGTTTGGGGATATCTACAAGGTG------GACGTTCTGTAGGCGAACTTGATTATTGGCGGCTCCGCATTCTTGGTTTTGCATCCTCCTCATGCTTATTATGAGAGCTATCCCTTTTAGTTAAGTGTGGCACATGTATGTCTAGATATCGATATCGATTGA------TTGATCTAATTTAGCAGATCTT----------TTCGCTTTGAGTGAAAGCGTGCATCCATGATGGAGTCGGTTTGTACTCGGGAGTATCATCTGACGTCCTCATGGGTAAAGGAGCATCCGGCGCTAACACTGTTTATGGTCAATTGGTCTATAGGCATGTCGAATTGGGGCAACAAGTTATCCATAAGTGAGTCTTCAAGATTTTATAATGTTTTAAGGGGTTCACCTTCACTGTAGCCAGCCTGCCCTTTTTAGATTCACTTTCCTATGTGGTTTAACTGATTC--AGATGGAGGTTTATATTTGGGTTTAGGTCTTTAGTTTTTGTGTTCGGAGTATCGGAAGACTACAGGTGCAACATCGGTATAAGGTTCGTTGGATGTATTTATATTCGAGTATTTTTTTTCATTTTTGGGTTTTTTTTCTTAACCGAGTGTTTGGAGTCTACCCCGTGGATCACCTTAATAATGGGTCCTTTTAAACATATTTCTTCAGGGGTACGTACTGTGCACAGGATAT----AGGGAAAACACAAGGTGTGGTTATATACGTATTTGGGTTTTTGTATTTCATTGAGATATAGAAGTCTAACATGCGAAGGTTGGGTCTTTTTAATTCACGGTTCAGGATCAGTATTTGGACGTATTTATGTAGTTGTCGTTATATAACATTAGAGACCAAATTGGTTGAATTCGCTGTATGCATTGTGAGTGTTGTTTTTTTTATTCATTATGTGTACTTGAATAGGTCTTTTTTGTTGTTGTATCAATGTAGGGATTTCTTCATCATAGAGCTCATCTAAGCTTTCAGGTGTTCATGTAAGAGTAATGGGTGCTATCGACGTCAGCAAGGGCTCTTTTTTATGCATTAATCAATGGCGGGCTTATAGGAGTACGATGT--GGGTTGTTGTGGGTGTTTTTTTTTCTATGCACGCTTTTGGTAGTTTCCTGGGCAATTCTATGGGGCACGTATACAATTCTTCGCATCCTATTGGGAGTATACGGTGTTTGGGGGTTTCGTGGAACCACTTGTCGTTTTTCTTCATTGTTTCCTTGTATTGTTTTGTTTTTCATTATTTCTCATGTTGGAGTCAACCTTGGTTTCAAAATCGAATATTATTCACCTATAAGGTAAGAGTCTGGTTATGTGCTTTTATGTTGGTGTGTCTCTTTCCGATTTTATGACCGAAATTATGAGCAACCATAAACCCCCCAATCACCGATGTATTCTTTGCTATCATCTCGTCATCCAAAATTGGGGGTGTTCTTTTGCAGGTATATTTGCTTCTCCCTTA----GCAAGGTTAGTTCGTTATGGTTAAAAATTTTATGTTCAGTATGTTTCTATTTACGTTTGGTCATCCGTGATACCTAACGTATAGCAGTGTGCTTATTATTGTAATTATTTCTGTCAATTTTTACATATTCATTTATGCGTGCTTTTTCCTTTGACGAAATTTAGATTCCAAACGGAGGCCAAAAATGTCTAAGGTTTCCACCATGGGAGCATAACTTTTCAATCACAGCTCC--AATTGGTTTATCGTTAACCTGGCCCTGCCTCTAGATAAACTTTTTGATACACCC----TTCTTCAATTATCATTAACATCGGATGTATCTCTATTTAGGTACGCCTCCACATTAAGGGTGTTTCAATCACTTAGACATGGGCATCGTCGTGCCATCCTTCTTGAATACTTAGTTAGCTTTATAGTTCTATATCATCTTTGTTGAAACTCCAATTCAAGTGAGTG--CTTTTGTGATCCTGATTCCTGTACTTATTGTTTTGCGAAGGGCGAGCCTTGACTCCGAGGGAAGGATTGTAGGGTAACATGATTGGGGTTCATAACTTATTTCTGGGAAACTGCGTATTTATACATATTTTCACACTACTGG--GGTTTCTTTGAAGCAGATTTGTTTATAT--------GTATATCGAGTCTACCCGATCGTAGACACTATCATTAATGGTGGATTTCCCACTATTCTCGTTGTTGTCTGTTTATATATTTTTTTTT----------------GTTTTCGTTTATTCTCATTCGAACAGTTGAACAAGATCTTGATGGGTTTTACGGAGGAATCATTCCTTTGGTCATTTTGTGTGGATCTTTGTGTTTGAATGATTGTGTTCCTCCCTATATGC----AATTAGGATTGTTGTTTTTG--TGTTGTTAAGATATTTTTATGGATCTTTGAGTATTGGGTCATGATGGGATATATGCCTTTAGGTGATTCTTCGTAGCATTTAGCGTCTTTCTGTAACCAGTCTTAATTAAAGTGTTGTTTTTTCATCTGTTTGAGGACTTGGTTACATCTGTTCACAGCTAAATGATCACATATTGGAATGATGGTATGTGTGAGGTTAGTCTTTTTTTTACGTTTCTGTAGTTATGTTTAATAAGGTCATATTTCCATTGAGGTTAAAATTATGTACCTTTTTTTGTATTCATTGATCTTGTTAGCCAGATTTTGGTATGTGGTGGAGGTTGGCTACTCTTGGTTATATTTTACAAATGAGAGTTAGAGGGTTTTACTTTTATAACGATGGGAATTTATATTCGTATGTATCTTCAG--------TGAAATTTAAAGATTCATAG----------TCGTGTCAATTCGTTTTTCTTTGGGCATACAAAGGCCATTCCTTTCTCTGCTCCCTGCATGCGCCCACCCGTAGAGTTGTCATCTCACTTGTACGCGGATTATTCTATCGTTGAAG--TTTTCCTTGAGTTTCAGC----CTAAATGTTCATATACATGTTAGCGGGTTCGCCTTGGAGTGATTAAAAGTGTGCCTTCACCTACCATTGGATATACTTTAGTTCATGGAGGTATGCGCACACCATTGTTTCAGCTTTTAAAGGCTTATGTCTTCTCATGCGGCTCCTTTTACGGTTTATCAAGAGT----------ATGTATTTGCGAGATCTTCTACCTGTTCTTTTTTGGGTATCTACTCGGTATCGTCGTTATGTTTATTTAATC--------------CCAATTATTCGAAAAAGATTATCAGTAGGAGGTTGTGGGTAGGTAAGGTTTTATAGGTCTGGGGAGGTTTCTTG----GTGTATCCGTACATGTAT----AATAAATTAGTGGGATTTAAAACTTTGTATGTTCCTGCGGGGCTATGCGCTTTGTCTT--TATTTGATGCTC--AGTGAGATGTGTTTTCTTTCGTCAATGTACTAAGGTATTTGCATAGGTACCATATGATGTATTTAGATATGGTTTTGGGGTTATGTTTCTTCTTATCTATATCTCC--AACCGATTGGGTTTAAAGTGAA--------------------TAGTGGCTTCATCTATTTTTATATTCACTGAC--------------------------------------TACTCCGTGTGAAAAAGACC------------GATTTTCCAT--TCCTCATTTGGCTAGGTTTGTTTTTTTTCTTTGTTTCTATATGTGGATATAATAGGTAAATCATTACT
