# Supplementary material for: Fragmented mitochondrial genomes in two suborders of parasitic lice of eutherian mammals (Anoplura and Rhynchophthirina, Insecta)
Source: Sci Rep. 2015 Nov 30;5:17389. doi: 10.1038/srep17389 (PMC4663631; doi:10.1038/srep17389)
Supplement: Supplementary Dataset 8 [file srep17389-s9.doc]

17 9530

Alloeorhynchus_bakeri TTTTCTACCTTTGACCCTAGAACAGGCCTATTC---------TTTTCATTA------AACTGAACTAGAACGTTT---------------------ATTATTATTTTACTATTTCCCAATCTATACTGACTTTTCCCTACCCGGAATAGAATTTTATTCCTTAAAATAAGAAATAATCTTCATACTGAATTTAAAACCTTGTTAGGTGAAGGAGGA------AGGGGTTTATCTATTATATTTGTAACCTTATTTATATTTATTTTATTTAATAATTTATTTGGTTTAATTCCTTATATTTTCACTAGATCTAGACATTTAACTTTTTCCCTAGCCTTATCACTACCAATCTGATTATCATTTATAATATTTGGG---TGAATTAATATAACTCAAAACATATTAGCCCACCTAATCCCACCGGGGACACCTAGAGCTTTAATACCATTTATAGTATTAATTGAAACTATTAGAAATATTATTCGTCCAGGATCATTAGCTGTTCGATTAACAGCTAATATAATTGCAGGGCATCTTTTAATAAGCCTACTAGGTAATAATTTAATTGAAAATTCCAATTTAACAAGAGCCTTACTAATTATAATTCAAATAATATTAATATTATTTGAATCAGCAGTTTCCATAATTCAAGCATATGTATTTTCTGTCTTAAGAACACTTTATTCTAGAATAGCACCTCTATGATGAAGAATTTTATTTATTATATTTATTATTTCTCTTATAGCAACATACTCC---AACTTGTATTTCCTATGAATACCAAGAAATACAAGTAGAATTGTGAATAAAAAT---------------ATAGAGGAGAAAGTAAATTTATATTCCACAAATCATAAAGATATCGGAACTTTATACTTTATCTTCGGACTATGAGCCGGAATAGTAGGAACCTCATTAAGATGATTAATCCGAATTGAATTAAGTCAACCCGGTTCATTCATTGGAGATGATCAAATTTATAATGTAATTGTTACAGCACACGCCTTCGTTATAATCTTCTTTATAGTTATACCTGTCATAATTGGAGGATTTGGAAATTGACTTGTTCCTTTAATAATTGGTGCTCCAGATATAGCCTTCCCCCGAATAAATAATATAAGATTTTGACTATTACCCCCATCATTATCCTTACTATTAACAAGTAGAATTGTAGAAAGGGGGGCAGGAACAGGATGAACCGTTTATCCCCCACTTTCTACTAACATCGCCCATAGAGGAGCATCAGTGGATTTAACTATCTTCTCTCTACACTTAGCAGGGGTATCATCCATTCTAGGAGCTGTAAATTTTATTTCAACAATTATTAATATACGACCTTCAGGTATATCACCTGAACGCATTCCATTATTCGTCTGATCTGTAGGAATTACTGCCCTATTACTATTATTATCATTACCTGTATTAGCAGGTGCTATTACCATACTATTAACAGACCGAAACTTCAATACATCATTTTTTGATCCTTCAGGAGGAGGAGACCCTATTTTATACCAACATTTATTTTGATTTTTTGGTCACCCAGAAGTATATATTTTAATTTTACCAGGATTTGGATTAATTTCCCATATTATTAGTAAAGAGAGAGGGAAAAACGAAACCTTCGGATCATTAGGAATAATCTATGCCATAATTGCAATTGGATTATTAGGATTTATTGTTTGAGCCCATCATATATTTACAGTGGGAATAGATGTTGATACACGTGCATATTTTACATCAGCCACAATAATTATTGCCGTACCCACAGGTATTAAAATTTTTAGATGACTGGCCACTCTACATGGATGTTCAATAAATTTTTCTCCTTCTATATTATGAGCACTAGGATTTGTATTTTTATTTACAATAGGTGGATTAACTGGAGTAATTCTAGCTAATTCATCTATTGACATTATTCTACATGATACTTATTATGTTGTTGCCCACTTCCATTATGTACTATCTATAGGAGCCGTATTCGCTATTATAGGAAGATTTATCCAATGATACCCCTTATTCACGGGGATAACTATAAATCCCCAATGACTAAAAATACAATTTATAATTATATTTACAGGAGTAAATATAACCTTCTTCCCCCAACATTTTTTAGGGTTAAATGGTATACCTCGACGATATTCAGACTACCCAGACAGATTTATGACTTGAAACATTATTTCATCAATTGGATCTACAATCTCACTAATCGGAGTAATAATTTTCATTTTAATTATTTGAGAAAGAATAATTGCCAAACGGCAAGTATTATTCCCAATAAATATAAATTCTAGAATTGAATGACTACAAAGAACCCCTCCTAGAGAACACTCATATAACGAATTACCCATAATTTCTACAATCTCAACCCAAGATGCTAACTCCCCTCTAATAGAACAACTTATTTTCTTTCACGATCATACTATATTAATTCTCACAATAATTACTATTATAGTAATATATTTAATATCAACACTATTTATTAACAAATATATT---------AATCGGTTCCTACTAGAAGGACAGACCATTGAATTAATTTGAACTATTCTTCCTGCCATTACCTTAATTTTCATTGCATTACCATCACTACGAATTTTATATATCATAGATGAAATTAATAACCCATTAATCACCATCAAAGCAATTGGTCATCAATGATTTTGAAGATATGAATATTCAGACTTTAATAATATTGAA---------TTCGATTCATATATAAAGCCATCTAATGAACTAACAATAAATGAA---TTCCGATTACTAGATGTAGATAACCGAATCATTATACCTATCAATACGCCCGTACGTATTCTAGTTACTGCCACAGATGTTATCCATTCATGAACCATTCCTTCGTTGGGAATTAAGATTGATGCAACCCCCGGACGATTAAATCAAGGATCATTTAATATTAAACGACCAACAATTATATATGGTCAATGCTCTGAAATCTGTGGAGCAAATCACAGATTTATACCTATTGTTATAGAATCAGTTAATATCCCTAGATTTATTAAATGACTAAATATAAATAAAAATCACCCCTTTCACCTAGTTAATTATAGACCCTGACCTTTGACAGGATCAATTGGAGCATTAACTCTAACAAGAGGTATAGTTTCATGATTTCAT---TTAAATAATATATCCCTTTATATTTTAGGAATAATTATTATTCTTATAACTATAATTCAATGATGACGAGATATTGTACGTGAAAGTACATACCAAGGTCTTCACACTAATAAAGTAATTATTGGATTAAAATGAGGTATAATTCTATTTATTATTTCAGAAGTTTTCTTTTTTATTTCTTTCTTCTGAGGGTTTTTCCACAGAAGTCTAGCTCCAACATCAGAAATTGGTAGTTCATGACCACCCTCTGGAATTATAACCTTTAACCCCATACAAATTCCCCTATTAAATACTATAATTCTATTATGTTCAGGATTAACAGCTACATGAGCCCATCATAGATTAATA------GAAGGTAACCGAACACAAACATTACAAGGGCTTACATTCACTGTAATTTTAGGTATTTATTTCTCTATTTTACAAGGATATGAGTATATAGAATCACCCTTTGCTATTAGTGACTCCGTCTATGGTTCTACATTTTTTATAGCAACTGGATTCCACGGTTTACATGTAATTATCGGGACAATCTTTCTATCAGTATGTTTATGACGACATTTTATATATCATTTATCAAAACAACATCATATAGGGTATGAAGCAGCAGCTTGATACTGACACTTCGTTGATGTAGTTTGATTATTCCTTTACATTTCTATTTACTGATGATTATGATGAAACTGAGGATCCTTACTCAGACTCTGTCTATTAATTCAACTATTAACTGGAATTTTCCTAGCTATACATTACACAGCTAACATTGAATTGGCATTTAATAGAGTAATTCACATCTGTCGAGATGTAAATCAAGGCTGAATACTCCGCAATTTACACGCTAATGGCGCCTCATTATTCTTTATTTGTTTATACTTACATATCGGACGAGGTATATATTATGGTTCCTATAAATTAATC---ACTACATGGTATGTAGGAGTAGTAATACTATTTGTAATTATAGGAACCGCCTTCCTAGGATATGTATTACCTTGGGGACAAATGTCCCTTTGGGGAGCCACAGTAATTACCAATCTTCTATCAGCTGTTCCTTATTTAGGTAATGATTTAGTTAAATGATTATGAGGTGGTTTTTCAATTGATAATGCCACTTTAACACGATTCTTTACATTACACTTTCTATTGCCCTTCCTAATTTCAGCCCTAGTATTAATCCACCTACTATTCCTACATCAAACAGGATCTAATAATCCTTTAGGACTAAATAGTAATTATGACAAAATCCCATTTCACCCTTACTTTTCAATTAAGGACTCATTAAGAACAACAATTGTATTATTCCTATTTATTATATTAAGAATATTAGAACCTCGACTACTAGGAGACCCTGAAAACTTTATTCCTGCTAATCCATTAGTAACCCCAATTCACATTCAACCAGAATGATATTTTTTATTTGCATACGCAATTCTTCGATCTATCCCTAATAAATTGGGGGGAGTAATTGCTATAGTAGCATCAATCTTAATTATTATAATTTTACCATTAACTAACAAGCCTAAGATACAATCAATAACATTCTACCCTATTAATAAAAGATTATTTTGAATACTATCAGTAAATTTAATTCTATTAACATGAATTGGGGCTCGACCAGCAGAAGAACCATTTATTTTTACAGGACAAATATTAACTATAACTTATTTTTTATATTTCATTGTAAACATTTCTTTTTTATTATTAATTTTAATGGTTTTATTGGCTGTGGCTTTTGTAACTTTATTAGAGCGGAGGGTTTTAGGTTATATTCAGTTGCGGAAGGGTCCTAATAAGGTTGGTTTTATAGGGCTTTTACAACCATTTTCTGATGGTTTGAAATTATTTTTTAAGGAACAGACTTGACCG---GAAGTTTCAAATTTTGTGATTTATTTTGTTTCTCCAATCTTTATGCTTTTTCTTTCTATATTGATGTGGGTTGTGTTTCCTTTTTTTGTTAATGTGGTTAGT------TTTAATTTGGGAGTTGTATTTTTTTTATGTTGTTCTAGATTGGGAGTTTATGGTATTATATTATCTGGTTGGTCATCAAATTCTAATTATGCTATATTGGGAAGTATGCGTTCTGTAGCACAGACAATTTCTTATGAAGTAAGATTATCTTTGATATTAATTAGTTTGCTTATTTTAGTTCAAGGTTTAAGTCTTATTTATTTTTGGATTTTTCAGCAAAATGTTTGGTTTATTTTTTTATCTATC---CCATTATTTTTGTGCTGATTTAGTTCATGTTTAGCAGAAACTAATCGTTCACCTTTTGATTTTGCAGAGGGTGAGAGAGAGCTAGTTTCTGGATTTAATGTAGAATACAGTAGAGGTGGATTTGCATTTATTTTTTTATCTGAATATATAAATATTATTTTTATGAGTATACTGAGAGTTATTTTTTTTATAGGTTGCGATTTAGGTAGATTAATATTTTATTTTAAGGTGGTTTTTTTGGTATTTATATTTTACTGAGTACGTGGTACTTTACCTCGATTTCGTTATGATAAATTAATATATCTTACATGGAGGGGATTTTTACCTGTTTCTTTAAATTATATTTTATTTATAATCTCACTATTAATCCCTGTATTATTAATT------------------------GTAATGTGCACTTTAATTGCAAAAAAATCCAATATAGACCGAGAAAAACTATCCCCCTATGAATGTGGATTTGACCCATACAAATCCGCCCGAATACCATTTTCTATTCAATTTTTCATAATTGCTATTTTATTTTTAATCTTTGATGTAGAAATTGTGATTATTCTACCCGCTTCCATTACCTTAAAATATGGTATGCTAAGAAACTGAATTATTACT---TCTTCATTCTTCATTATTATCCTTTTACTAGGATTACATCATGAATGATATAACGGAATTCTCGAATGAACATTACTTTTAACTCTTTTGAGATTAGAGGTTTTAGTGATTTCATTATTTATTTTTTTATATATATATGTATTATTATATGGGGAAGGGTTTTATTTTATTATGGTTTTTTTAACCTTTTCTGTGTGCGAAGGTTGTTTAGGTCTTTCTATTTTAGTATCTTTAATTCGTTGTCATGGTAATGACTATTCTTGTACTTTAGTTATAACAATTCTTTTTGATTTTAAGTCTTTAATATTTATGTCTAGAGTTATAATAATTTCTTCAATGGTTATTTTTTATAGTTCTATTTACATAGAGGGGGATAAAAATCGAGTACGATTTTTATATTTAGTTTTAATATTTATTATATCAATAATGATAATAATTATAAGACCCAATTTAGTGAGTATTTTGTTGGGTTGAGACGGTTTAGGGTTAGTTTCTTATGGGTTGGTAATTTATTTTCAGAATTATAAATCTTATGCAGCTGGAATATTAACAATTTTAATAAATCGTGTAGGGGATGCTGCTATTTTAATTTGTGTGGGTTGGATAATTAATTTTGGAAGT---TGACATTATATATATTACTCATTCATCTGG---------GATGAATGAGTAATATATATCTGTTTATTATTAATTCTTGCTGCTTTCACTAAGAGAGCCCAAATTCCGTTTTCTTCTTGGCTTCCAGCTGCTATAGCAGCCCCAACACCTGTTTCAGCTCTAGTCCATTCTTCTACTTTAGTTACAGCCGGGGTTTATTTATTAATTCGATTTAGAGGT---GCTTTTGTTAATATAGATTGTTCTTTATTT---GTTTTGTTT---GGTATAATAACTATATTTATATCAGGTTTAGGGGCTAGTTTTGAGTTTGATCTTAAGAGGATTATTGCTTTATCAACTTTAAGACAGTTAGGCTTAATAATATCAGTCTTATTTATGGGTTATATTGATTTATCGTTTTTTCATTTATTAACTCATGCTTTTTTTAAAGCTCTTTTGATTTCTAATATGTCTTTATGTGGTCTTCCCTTTTTATCTGGGTTTTATTCTAAGGATTTT---ATACTAGAA---ATAATAGTTTTTGATTATTATAATTTAACGGTTTTTTTAATTTTTTATATTTCTGTAGGTTTAACCTCATGTTACACAATTCGTGTTTTATATTATTGTATTTTAGGCAATAAT---------------AATATATATGTGTGTCAA---------TTATATAAGGAAAATTATGTTATGATTTGTTCTATAATAATATTGGTTGTTATATCTATTATAGGGGGTTCAATTTTATCTTGG------------------TTAATTTTTAGATTT------CCAGAATTATTAGTTATGACTTTAACTTTAAAGTTAATAGCTCTTTTTTTTATTATATTTGGTATAATATTAATCTTAACTAGAATA---TTATTACCATTACTAAAG---CATCCATTAAGAATAGGACTAATCATTATCTTGCAAACCCTGATCACTGCTATAATTACAGGACAAATAATTAATATATTCTGATTTTCATATATTTTAATATTAACTATAACAAGAGGTATATTAGTTCTATTTATTTATATAGCAAGAATAGCATCA---AAT---GAAAAATTCAAAAGTATAAAGATATCTGTACTA------------------------TCACTTATCTTAATATTACTACCCTTTATAATTATAATTAATTATAAA---------------------------GAATTCTTTATAATTAATTTATTTAATAATAAATATATAACATTATACTTAAATTATGAA------------------CAATCCATAATTGTAACAAGTTTATTCAATAAACAAGCCTTAATAATTACCATTATACTAGTAATATACTTATTTTATTCATTAATCAGTGTATCCTGAATCGTAAATGTATTTGAAGGAGCGGTTATACAATA-----AGGTATAA-TGAATATTCTTGGTTAATATTAT-------------TTTGTTT-------TTGTTAATATATAATATACAC---ATTGTTAGGTGAAATGTATATGAAAGCTGAT-----------TTAAAGACTAGGATTAGATACCCTATTATT-TTAGCTGTAAAATAA----TAATCATAGTACTAAGAGTTAT----GATCTTG--------AAATTAAAATAATT-TGGCGGTAATTT---AATCTTTCCAGAGGAACCTGCCCTG-TAA-TTGATAATCCACGTTAGATTTAAC---------------------------TTTATTT-----TGACTTGTATACCTCTGT-----CATTGAATGTT-----CTGATAAGTTATTTTCT------------------------TATATTTTGGT------------------------AAAAAAATATGTCAGGTCAAGGTGCAG-TAATAATAA-AGTA--GAGGTGGGTTACTTTTAA-----ATTATT------------AGTGGATGAATATTTGAAATAATAG----AATTAAATAGGATTTGGTAGTAA--GTTTAATTAATTAATAGAACTGATTAAGGCT-CTAAATTATGCACATATCGCCCGTCGCTTTATGAAATAAGTCGTAACAAA-GTAGATCTACCGGAAGGTGGTACCTTTTGTATCAGGGTTTATTAAAT-TAT-ATTAATTATT--TAAT------TTTCCCGAAAT--TA-ATGGGGATATATTAGAATAATTGTTTATGTAGTAATA--TAAAATTAAATTCTAATATAGAGA-TGATATGTCATTCAACATTAA-TAT-ATCTGGTAACTCGGCAAATTTAA---CCTTCACCTGTTTAACAAAAACATGTCCTT-TAGTGAAT-TGATTTAAGGTCAGGCCTGCCCAATGA----TTAATTT--AATGGCCGCAGTATTT-TAACTGTGCGAAGGTAGCATAATCAATTGTTCTTTAATTGGGAACTGGAATGAATGGTTTGATGAGGGGTTAACTTTCTTTATTATATAGAAT----GAATTTAATTTTTAGGTTAAAAAGCTTAAATTAAGTTAAGGGACGAGAAGACCCTATAGAACTTTTTTGGTTGGGGTGACGAATAAATT-TTACTAACTTT----ATTTATTATTTTTCATAGATAAGTGTTTTTATGATCCGTAAATTACGATTATA-A-GATTAAGTTACCTTAGGGATAACAGCG--TAATTTCTCTAGAGA-GTTCACATTAA-TAGAGGAGTTTGCGACCTCGATGTTGGATTAAA-ATAAGTTTTGGGCGTAGTAGCTCA---TTAACTAAGTCTGTTCGACTTTTAAAATTTTACGTGATCTGAGTTCAGACCGGCGTGAGCCAGGTTGGTTTCTATCTTTAA-ATAGAATAATACTTT----TTAGTACGAAAGGAC

Bothriometopus_macrocnemis CTTTCAGTGTTTGATCCAGGCTCAAGAAATTTGTCTTTT---ATCCCTTTA------AAATGACTAATCTCTTTA---------------------ATTGGGTTTATATTAATT---TGACCAATATGGACAGTTAAATCTGGGCATTTTACTTTCATAAACTTTTTATCTATAGGATGAAAAAGTCAACTTGAAGAACAATTTAAGTTTTACAAA---------TTCCCTTTAATTTTTAGTTTGTCTATTTTTATCACAATTTTTTGTATTAATGAAATGGGAATAGTTCCTTATTTATTCACTCCGACAAGACATTTTAGTTTAAATCTTTGTCTTGCCCTTCCTTTATGACTAACTGGAATTATTTGTATA---TTAAAAAAAAATTGAAAAGCTTTATTTTCTCATTTGGTTCCTGAAGGGAGACCAATAGGGTTGGCTCCACTATTAGTGATTATCGAGCTAGTAAGTTTATTAATTCGTCCTATTTCCTTGAGAATTCGGTTAATATCAAATATTATGGCAGGACATATGATTTTAAGATTAGCATCTAGTGCGGTCTCTTCTATATCAATTAGCTCATCATTTTTAATAGAAATGGTGGTATTTGGTCTAATAAGTTTTGAACTATGTGTCGGAATTGTTCAAGCTTACATTTTTTCTTGCTTATTAGTAATGTATTGATCATTTTTACCAATTTCTTTTACATTGTTAATATTTTTTATATCAATTATTATCATAATTATTGTTGTT---CAAAACTTTTTTACTCCAGTCTTCAGTGAAAGAGATAGAGTGGGAAGGGCAGCC------TATATGATAGACAGACAGTTTGTTTATTTTTTTTCAACTAATCACAAAGATATTGGAATTTTATATATAATTTTTGGGGTGTGATCAGGATTGATTGGTTTTGGGTTAAGAATAATCATCCGAATTCAATTAGGTTCACCTTGGGGGCTACTGTTTGATGGTCATATTTTTAATGTTGTTGTTACAATTCATGCTTTTCTAATAATTTTCTTCATAGTTATACCTATAATAATTGGTGGATTTGCAAATTGGATAGTACCTATTATGTTAGGTGCTCCTGATATGGCTTTTCCTCGAATAAATAATATGAGATTTTGACTTCTTCCTCCTTCATTGATTTTACTTTTAATGGCTACATGCATGGAATCAGGTGTAGGATCAGGATGGACGTTATATCCTCCTCTATCTTCTATCTTAGGAAGACCGGGTTTAGAGGTGGGATATTTAATTTTCTCCCTTCATTTAGCAGGTGTGAGATCAATTATAGGAGCAATTAATTTTATTTCTACTATCCTTAATATGTGGTCTTATTCTTTAGATTTTACCAAAATACCTCTATTCTGTTGGGCTGTGCTTATTACAGCCATTCTTTTACTTCTATCTCTTCCAGTTTTAGCTGGAGCTATTACTATGCTTCTGTTAGATCGAAGCTTAAATACTTCCTTTTTTTCACCAGAATTAGGAGGTGATCCAATTTTATATCAACATTTGTTTTGGTTTTTTGGACACCCTGAAGTATATATTTTAATTATTCCCGGATTTGGTTTAATATCCCACATTATCAATGAGTGTAGGGGAAAACCATCTGCTTTTGGGAGATTAGGAATAATTTACGCTATACTTACAATCGGATTATTAGGATTTTTAGTTTGAGCTCATCATATATTTACAGTAGGTATGGATATTGATAGACGAGCATATTTTACAAGGGTAACTATAGTGATTGCTGTTCCAACAGGAATTAAGGTTTTTAGATGGTTGGGAACAATTTTTGGGAGAAAAATTAATTGATCTCTCTCTTCTTTATGAAGAGTAGGGTTCATTTTTTTATTCACCTTGGGTGGACTCACAGGTGTAGTATTAGCTAATTCTTCAATTGATACTTTCATACATGACACATACTATGTAGTTGCTCACTTCCGCTACGTCTTGTCTATAGGGGCAGTATTTGCTATGTTTGCTAGATTATTTCATTGATTTCCTTTATTTACAGGTTTAACTCTTAATCAAAAGCTTATAAAAATTCACTTCTTTGTAACTTTTATTGGAGTAAATTTAACTTTCTTTCCTCAACACTTCTTAGGACTGATGGGTATACCTCGACGTTACGCGGACTACCCAGATTTATATACTCCATGAAATTCAATTTCTTCAATTGGTAGATGTATTTCTGTGGTCGGATTATCAATATTGATTTATGCTATTTATGAAAGATTAATTTCTCAGCGAAAAGCTATTATTCATTCTATAAATATAAGCTCTGTTGAGTGATTATGAGGGTGTCCTCCTTCAGCTCATTCATTTGAGTCAATTTCTTTTATTTCTAAGATATTATTTATAGATCCAATTTCTTTTTCTGGGGAAATTGTTCAATCAGTTCATGACCATGTAATGATTATTATTACTCTAATTGTTATATCAATTAGATATGTATTTTTCATAATATTTTGTCGTTCGAGGCAGATTATGGGAGAACGATTATTTCAATCCAGAGAAATTTTGGAAACAGTTTGAACTGTTTTTCCAGTGTTAATTTTAGTTTTTGCAGCTATTCCCTCTCTACATAGTTTATATATTTTAGAAGAAGAAAAAAATCCAATTATCTCTGTGAAGATTTTAGGAAATCAGTGATATTGAACTTATGAGTTTAATACTGGCTATCATATTAAT---------TATAATTCTTACATAATTCCTTCATCAGATTTAGGAGATAGAGAT---CTACGGAATTTAGAAGTCGATAATAACTTGGTTCTACCAGTTGGGGTAGAAACTCGAGCAATTATCACATCGAGAGATGTGATTCACTCATGAGCAATCCCACCATTGGGTGTGAAAATAGATGCAGTACCTGGCCGAATTAATCAAACAGTCTTTTCAATTTCAATATCTGGTCTATTTTATGGGCAATGTTCTGAAATTTGTGGATCTCTTCATTCATTCATGCCAATTTGCGTAGAAGCTATTCCTCTAAAAAATTGGTTAAAATGATTAGAATTAACTCAATTTCACCCATTTCATATAGTCCAATTGAGACCTTGACCATTGATTTGTTCTTTTAGACTATTTAGAATAATCATTTTAATATATGATTTTTTTAAT---TCTTCAAGATTAAAAAGTTTAATTTTTGCTGTACTTATTCAATTATTAGTTTTGTTTGAATGGTGACGAGATGTGTGTCGAGAAAGAACTTTCCAAGGTTGACACTCTTCCAATGTCGTTCGAGGATTAAAGATTGGATTTATTATATTTATTTGTTCAGAAGTCTTATTTTTTTTTTCTTTCTTTTTTGGATATTTCTTTCTTTCTCTTAATCCGGATGTGGTCTTTGGGGGAATTTGACCCCCAAAGGGCTTAATAGTAGTTGATTTTTTATCCGCTCCCACTCTTAACTCAATTCTTCTTCTATCTAGGGGAGTAAGAATTACTTGAGCACATCATAGAATTCTT------GAATCTAATTTATCTGAGGCAAAAATGGGATTAGTTTACACAGTTTTTTTGGGAATTATATTCTCTATAATTCAATTAATTGAATATTATGAATGTTCCTTCACTATTGCTGATAGACCTTTTGGGTCTATGTTTTTCTTAGCAACTGGATTTCATGGTATCCATGTTTTAGTAGGAACAATTTTTATTATTATTTCTTTTGTTCGCCTTTTAAATAATCAATTTTCAAAGAACCATCACGTCGGCTTTGAAATGAGTTGTTGATATTGACATTTTGTAGACGTAGTATGATTGTTTTTATTTGTTCGAGTATATTGAATATATATGTGGAATTTTGGATCTCTTTTAGGAATTAATCTTCTTCTTCAAATTGTGAGAGGGATCTTTTTAGCAATACATTATGAAGATACTATTACTTCTGCATTTGAAAGAGTGGTTAGAATAATAAATGATATAAATAGAGGTTGATTAATTCGCTTTATTCATGCGAATGGGGCTTCTTTATTTTTTGTTCTTCTGTACTTTCACATCGGTCGAGGGTTGTACTACGGGAGATATAATTTTACA---GGAACTTGGATTGTGGGAGTGATAATTATATTCATTCTCATAGGAACAGCATTTGTAGGGTATGTCCTCCCATGAGGACAAATGTCATTTTGAGGGGCCACAGTTATTACTAACCTTGTTTCGGCTGTCCCATTTATTGGGACAGATATGGTTATTTGATTGTGAGGTGGATTTTCCGTTGACAATCCCACCCTAGTTCGTTTCTTTTCAATCCACTTTGTCTTACCGTTTGTTATTTTGGCTATAGTTATTCTTCATCTTTTATTTTTACATTCAACTGGAAGATCCAACCCACTAGGATTAGCAAATGACAGGGATAAAGTTTACTTTCATCCCTTATTTTCAATTAAAGATATCCTCGGATTAATTATTGTAACATTTTTCTTTCTTTCCACGGTCTTTTTAAAACCAGAAAGATTGATGGATCCAGACAATTTTACTCCTGCCAACCCTATATCTACTCCCCAACACATTCAACCTGAATGATATTTCCTATTCGCTTATACCATTCTTCGATCGATTTCATCAAAGTTTGGGGGAGTAATAGCTTTGGTGTTTTCAATTCTAATTTTAATATTCTTACCTCTTCTTAATATCAAAAGATCTCATAGAATCTTATAT------------AAATTTTTTTTTTGAATTCAAGTTTCAAACTTTATTCTTTTAACCTGATTAGGTTCCATACCTGTAGAACAACCATATGTGCAACTAGGACAAATAGTTTCAATTAGCTATTTTTCTGTATTTCTAATTTGAATTCAACTTATTTTTTTAGCTGTCGGTGTTCTTTTATCTGTTGCTTTTTTTTCACTTTTCGAACGGAAAGTGTTGGCTATCATTCAAAATCGAGTCGGTCCGGACAAAGTTGGGATCGTAGGAATTCTTCAACCTTTTAGTGATGCAATGAAATTGATTTCAAAAACTGAATCCCCATCTCCAAGAAAAAAGGTTGGAGTATTGTATTTTTTTTCTCCAGCTATTATATTTGTAATTTCTTTTATTGTTTGAATTACTTTCCCTTCGAATTGAGAAATTTATTCA------TTTGATAAGAGGATATTATTTGTTATTGCCTGTATAGGCTCAAGAGTTTACGGTCTTGTAATAACTGGTTGATTTTCTAGGTCTAAGTATTCCTTAATTGGAAGGGTTCGAGCTATTGGAATGTCCATTTCCTATGAAATTATCCTAGTATTGGGCCTAGTGTTAATAATATTTCTTCTTGGAACTATAAGGGTAAAATATATTATTTCTTTTCAAGAACAAATGTGGTTGTTCTTTCCTTTGTGA---TTTGTATTTATTATTCTTTTAATCTCTTTTTTAGCCGAAAGTGGTCGAGCTCCTTTTGATCTCTCCGAAGGAGAGAGAGAGTTAGTGTCAGGATACTCTGTTGAGTATGGAGGTATTTCATACACTTTAATTTTTCTATCAGAAAATAGATCTATTATTTTTAGATCAGTTATCTTATCAAGAATATTTTTTGCG------ATAGGAAGAATTTTAACTCTTCCTGGCTTAATTTTTCTT---------GTCGTTTGAATTCGTGGGACGGTGCCACGAATGCGATTTGATCAATTAATAATAATGTGCTGAGTTAAAATTCTTCCTATTATGTTATTTATTTTTGGAATAATTTTTGTTGTTTCTCTTGTATCTCTTCTGGTT------------------------TTTATCGCAAAATTGTTCGATTCTCACGAAGAAGAA---TCTAGATCTGGAGAAGAGTTTGAATGTGGAATAGAATCAATACACCCTACTCACACTCCCATAAACATACAATTTTTTATAATTGGAATTTTATTTCTTATTTTTGATATCGAAGTGGTAGTAATACTACCTTTAATCATTTTA------TCTTGAAATGAAAGAGCAATTTTGATTATT---ATATCTATGATTGCGGCTATTTTAATTATTGGAATATGAATAGAAATTTTCATGGGTTCGTTATATTGAAAGATAATAATATTTCTTATTACTCTCGAATCTGTGATGATACTACTATTTTTTTATATAAGATGTTTTCTCAACGCT---------CCAATTATTTCATTAGTAACCTTTTTAGTTTTAATAGTTCTGGAGGGGGTGGTGGGGTTGAGAGTGTTAGTAAGAAGTGTGAAAATATCTGTTTCTCCATTTGAACTATCATCAGATATAACTATCATATTTGATCAGTTAAGAGTGAGATTCATATTCATGGTTCTCTCAGTTAGAACTTGCGTTCTAATTTATGCTGTGTGATATATAGAAGGAGAAAAAAATTTTAATAAATTTATTGTAACTTTATTCATATTCATTATTTCAATAATGTTCTTATGTATGAGAACAGATATTTATTGAGTTATAGTAGGTTGGGATGGACTAGGAATCACTTCATTTTTTTTAATTATTTTCTTTCAAAATTGAAAGAGGGTAAGAAGGGGGATAGTAACTCTCCTATCTAATCGAATTGGAGACGTGTTTATTGTTACATCAATTTGTCTGGATGTATTTTATTTCGAA---AGAAAATATTCATTG------------------------------------------------ATTTTAATTGCTCTTGGAGCAATTACAAAAAGTGCACAATATCCCTATTCAGCGTGACTTCCGGAGGCTATAGCAGCTCCCACTCCAGTTTCTGCATTAGTCCATTCTTCTACGTTAGTAACTGCAGGAATCTACCTCCTTCTTCGATTTAATGAC---ATATTTAATAATGAATTAGTAAGACTGTTC---ATTCTTTCAGTTGCGAGAATGTCAGCTTTTTTGTCAATCTCTAGAAGATGGGGTGAGTTGGATTTAAAAAAAATTATTGCTCTATCTACTCTCTCTCACTTGAGAATGATAATTTTATTCATCTCTTGTAAAGATTATCTCTGTGCAATTATCCACATAATTAGACATGCATTTTTTAAATCTTCTTTATTCTCTTGTGCTTCAATGGTGGGTGGACCGTTTTTAGCAGGATTTTATTCTAAGGAAATT---ATAATTATA---TCACTATTTTCATTTTCTCAAAAAACTTGAATTTTATGAATAACAATGTTTTTGGTTTTTGGGTCATGTCTTTATTCTGCCCGCATTATTTTCCGCCTTCTATCAAACTTA------------------CCATTCTATTCCTACTCT------------TCGGATCCAGAAGAGTTTAATTCAACCCCTCTACTCATCGTAACTATTCTTAGAATTTCTGCTGGAAGGATAATCATCTGA---------------CTGACTATTTCTTCTTGA------CCAAGATTATCATTTCAGCCAAAAGAATCTGGGGTGAGAAAGTTATTT---CTTTTTTTATCCATAATATTTGTTGGATTAATGGGT---ATTTTCTGTTTAAATATC---AGTCCAATGATGGGAATGATTTATTTATTAGTGAGTACAACTATTTTTACGACAATTCTTTTAATTGTCAAAGGATCT---TTTGTAGGATTTCTATTTTTTTTGTCTACAATTTCCGGACTATTTATTCTTTTTTCTATTTTTATAATAAGAATGAAA---------ATTAAATTT---TTTTCTAAAACTCTTGTGTTAAAA---------------------AGAAAAATCTTCTTTATAATTATCCTATCATCCTTAACAATAATGAGG------------------------ATATTTTCATCTCCGCCAAATGAATGAGGGACATATTTATGATTGGAGGAACAAATTCTTTCA------------------AAATCAGGGTTAATA------------------------ATTTGAATTTATGTTTTTTTAATTTTTTTATTACTTTTATCTCTTCCAATTGTCGATCAAATCCTCAAAGACATTTCTTCTGCGGTAATACATTT-----AGGAAAAA---AATTT-----------------------------CAAAAGAAGAGAATTTCAAAGAAAGAAAAATTAATAAAAATTAATGGTAGAATTTTATTAAAAATTTAT---CAACCCACTGAAAAACTAGGATTAGATACCCTACTATT-------AGAGGACCAACCAACTTCAACAGAGTAGTTGATA-----AAGCTTG--------AAAAGGAGGCAGAA-TGGCGGCA----ATAAACCCGATCAGAGATGCATGTTTGAACAG--CGAAAACACACGGCAAATCCATC---------------------------TCAACCTATG--TTCTTTGTCCACTGCCGT---CTGAAGGAAGATT-----TTGACAAATCGTTCTTTTCCTGGAGTAAATATTCATGATTATTTACTTATAAAATCCGTTGAAAAAGATTTTATAGAGAGGGGTAAGTCAGGTAAAGGTGCAGGATATGGAAG-AGAA--AAAATGTGCTTCATTACT-----ATTCTTA-----------CTTACTTTAAT---TTGAAATATTA----AAAGAAGAAGAATTTGATAGTAAG-ATAAAAAAATAAAATTTATCTGAATAGGCT--CTTTATTGTGTACAAATCGCCCGTCACTCCAAAGGATAAGTCGTAACAAA-GTTAGTCTACCGGAAGGTGGTACCTTTTGTATCAGGGGTGGAAGAAAAAATTATTGATTAATATAA----------TCCCGAAAG--AG-AGTGATCGATTTGAATTG-ATGGTTAAA-GTGAAATGTTTTATCAAAAAAGTTCAGATAGAAA-TGAAATGTT-TTCAAACTCTCATGT-ATCTGGTAACTCGGCAAAAAAA----AGCCTGCCTGTTTATTAAAAACATCTCTTCAAGG---AA-GAATTTAAAGTAGGCCCTGCCCAATGC-----ATTATGTCAATGGCTGCAGAATTT-TAACTGTACAAAGGTAGCATAATAATTTGTTCCTTAATTGGGAACTGGAATGAAAGGGTTGACAAGGCTTAATCTGTCTCATTTTGGAGAAAA----AAAATTGAACTTGGAGTAAAAATGCTCCAATTAAGAAGGGGGACGAGAAGACCCTGTAGACCTTTTTTTCCTGGGGAAGGACTTG-----TTTACAAATAACGGCTAAAAAAAAACT-----AAT----GGGTTAATGATCCATAATAATTATGATAAGGAGAAAAAGGTACCTCAGGGATAACAGCA--TAATACTTTGAAAATAGACCAAATATA-ATTAAAGGCTTATGACCTCGATGTTGAATTAAG-ACTTATGGATGGAGGAAAAATTATC--CAATATG-GTCTGTTCGACCATTAAAGTCTTACATGATTTGAGTTTAGACCGATGTAAATCAGGTCAGATTCTATCTCCTT-TTTA--CGATTTCTTT---TTAGTACGAAAGGAA

Campanulotes_bidentatus TATGGTTTGTGG---ATTGTTTTCTTCGTTTGATCCTTCTTATATAGAATTATATGGGTATGAATCATTACCGTTAAAATGGTTGATGAGATTTGTGCTGGTTTTTTTTTTTGTAAGTGACGAGTTTGGGTTTCTGTTTCTGGAGTTGATTTATTTGATAAAGAAATAGTGAAGTTTTTTTTTGATCTTTTAAAACAATCTATAGGAGGGTTTATG---------AAGATTGGACTGGTCTTATATAGTGTTTTTTTTTTT---TTCTTTTCTAATTTGCTAGGATTAGTTCCTTATGTATTTACTCTTTCTTCCCATTTGTGTGTAAATTTGAGTGTTTCTTTAGTTTTGTGGCTAGGAGGGGTGGTTTATTCG---ATAAAGAAATCTATGGATTCGTTTCTTTCTCATATAGTTCCTTTAGGTTCTCCTGTATTTCTTCTTCCTTTATTGGTTTTAATTGAAACAATTAGGACATTGATTCGTCCGTTAACTTTGGCTATTCGGTTGATGGCTAATGTGATAGCGGGCCATTTAATTATAAGGTTGGTTGGAGGTTTTAGATCAAGACTGGGTTTAGTATCTATT---TTTCCCATGTTTATTGAGTTGGGATTTTTGTTTTTTGAAATGTGTGTGGCTGTTGTTCAGGCTTATGTTTTTAGCAGGTTAATAGTGATATATTTAGCTATAATACCGATTTATTGGTTTTTTGTTTGTTTGATAGTAAATTTATTTATTTATTTTTTTCTTAGC---TTTCATTATTTTAATTTTCTTGTTTTTTCTCATTCTGAAAAAGTTTGTTTT---------AAAAGTGTTAAAGTGAGGTTTATTAGTTTTTTTTCTTCTAATCATAAGGATATTGGTATGATGTATTTAATTTTTGGAATGTGGAGAGGATTATTAGGTTATGGGATAAGGGTAGTAATTCGTACGGAGTTAGGGGAAAGAGGTTCGTTGATTAGAGATTCTCATATTTTTAATGTGTTTGTTACGGCTCATGCTTTTTTAATAATTTTTTTTATAGTTATACCGATTATAATTGGGGGTTTTGCAAATTGATTAGTTCCTGTGATAGTAGGGGCTGTTGATATGATTTTTCCTCGGATGAATAATATAAGGTTTTGGCTTCTTCCTCCGTCATTAGTTTTATTATTAATAAGGAGGGTTATTGATAATGGGGTAGGAACTGGGTGGACGGTTTATCCTCCACTTTCAAGATTTGTTGGTCAACCAGGAAGGGCAGTGGATTATGCTATTTTCTCTCTTCATTTAGCTGGGGTTAGGTCAATTATAGGGGCAATTAATTTTATCTGCACTATTTTAAATATGTGAAGATTTCCGAAGAGGTGGGATTTGGTTCCGTTGTTTTGTTGATCGGTTTTAATTACTGCATTTCTTTTACTTCTTTCTCTTCCAGTATTGGCAGGAGCAATTACTATGCTTTTGTTTGATCGGAATATTAATACTTCATTTTTTGATCCGTCGGGAGGAGGGGATCCTGTCTTGTATCAACATTTGTTTTGATTTTTTGGTCATCCGGAGGTATATATTTTAATTCTTCCTGGATTTGGTTTAATTTCTCATATGTTAAGGGATAATAGTGGAAAAATAGAGGTTTTTGGATCATTAGGAATGATTTATGCAATAGTAGCAATTGGAGTGTTAGGATTTATTGTTTGGGCTCATCATATGTTTACTGTTGGTTTGGATGTAGATAGGCGGGCATACTTTACTTCGGCTACTATAGTAATTGCTGTTCCTACTGGAGTAAAAGTGTTTAGATGAATGGCTACTTTATTTGGAAGACGAGTAAAATGAAGTCCTTCGGAATTATGAGGAATTGGATTTATTTTCTTGTTCACTGTTGGGGGTTTAACTGGAGTAGTTCTTGCTAATTCTTCTTTAGATATTATTCTTCATGATGCTTATTATGTGGTTGCTCATTTTCATTATGTTCTTTCTATAGGAGCTGTATTTGCTGTATTTGGAGGATTTATTCATTGGTTTCCAGTAATTTTTGGGGTAAAAATAGAGTCTGTGTATTTAAAAGTTCAGTTTTTTTGTACGTTTGTAGGGGTAAACCTAACTTTCTTCCCTCAACATTTTCTTGGGTTAATGGGAATACCTCGGCGTTATTCTGATTATCCAGATATGTTTTATTCATGGAATTTTATTTCTTCAATAGGATCGCAAATTACTTTGGTTGGAGTTTCATTGTTTTTCTTTTGTTTAATTGAGGGGTTTTTTAGAAAACGAAGAGTGTTATTTTCGTAT------TCTAGGTCTTTAGAGTGAATGATTGGGTATCCTCCAAATTCTCATTCTTTTGAAATAGGAGTTCAAATTGTAGAATTTTTTTTGCAAGATAGGTGAGGTCCTTTAATAAGTCATATTTCTGGTTTTCATGATCATGTAATAGTTGTCGTTTTAATAATTTTAACTGTGGTTGTATATATTAATATGGTAGTTTTTTTTTTTCCTTGTTAT---------AGTCGGTTTATAAAAAGAAGGGAGGGTTTAGAAACTTTGTGAACTATTCTTCCTTGTATAGTATTAGCTTCTTTAGCTGCTCCTTCTTTGATAACTTTGTATTTGTCTGATGAGTTAAGTAATCCTGTTGTGACTTTGAAGGTTATTGGACATCAATGGTACTGGTCTTATGAATATGAGGATTTGTCGTCTTCTTCT---------TTTGATTCGTATATAATTCCTACTTGTGATTTGTTAAAAGGAGAT---TTTCGTCTTCTTGAAGTAGATAAGAGGGTAAAAGTTCCTTTGAATAGGGAGAGTCGGGTTTTTGTGACATCTTCTGATGTGATTCATTCTTGGACTGTTCCGTGTTTAGGGGTTAAGGTTGATGCTATTCCGGGACGGTTGAATCAGTTGAGTTTATATCCTTCTCGAGTGGGTTTAGCTTATGGTCAATGTTCGGAAATTTGTGGTTCAATGCATTCCTTTATACCGATTTGTTTAGAGGTGGTTCCTCAAGAGGAGTTTTTTCGATGATTATGGAAGTCTGGATTTCATCCGTTTCATGTGGTAGATTTAAGGCCCTGACCATTGGTAATGTCATTGTCTGTTTTCTCGTTAGAATTAAATTTGTATCATTTTTTGAACTTATCTGGAAGGGTGGTATGGATAATTGAGAGATTTTTTTCTTCAATTTTAGTAAGAGCTTTGTGGTGACGGGACGTAATTCGAGAAAGAACTTTTCAAGGACATCACTCTGAGGAAGTTCAAAAGGGATTGGTTCTTGGAGTGCTTTTATTTATCTGTTCAGAAGTAATGTTTTTTTTTTCTTTTTTTTTTGGGTTTCTTTTTTCTGCTTTATGTCCAGATATTGAGATTGGAGAGAGATGACCTCCTTTGGGGATTGAACCACTAAATTTTATGATGGTTCCATTAATGAACACTTTAATTCTTTTATCTAGGGGAGTGTCAATTACTTGATCCCATCATTCTATTATA------GAAGGGGATTGAAAGAATTCTCTTTTTGGGATGGTTATTACAGTATTTCTTGGATTTGTGTTTTCTTTTCTTCAATATGAAGAATATTTTTCTTGTTCATTTACGATAGCAGATAGAGTATATGGATCTTTTTTTTTTCTAATAACGGGATTTCATGGGATTCATGTGATTGTAGGAGTTTTGTTTATTATAGTAAGGTTATTTCGAACTTTAGTTGGGCATTTTTCAAAAAGTCATCACTTTGGATTTGAAGCTGCTGCTTGGTATTGGCATTTTGTTGATGTAGTTTGGTTGTTCTTGTTTGTAACTGTATATTGATGATATTTTTGGAATTTTGGATCTCTTCTTGGGGTTTGTTTAATAATCCAATTAGTTTCTGGGATTTTTCTTTCTTTTCATTATTCTCCTACTATTGAGGAGGCGTTTTCTAGAGTAGTGATGATTGTTGATGATGTTCCGTTTGGATGAATATTTCGAAGAATTCATGCTAATGGGGCTTCGTTTTTTTTTTTTTGGGTTTATTTACACATTGGTCGGGGTTTATATTTAAGGAAATATAAGTTAAAC---CCTGTTTGAATGAAAGGGGTTTTAATCTTTTTTTTTTTAATAGGGACGGCTTTTATGGGATATGTTCCCCCATGAGGTCAAATTTTTTTGGGGGGGGCAACAGTTATTACTAGTCTTTTATCTGCTATTCCTTATATGGGGGGGTTTTTAGTTAAATGGGTTTGGGGGGGATTTTTTGTTAAAGGACCGACTCTTCATCGTTTTTTTTCTCTTCATTATCTTCTTCCTTTGATTTTATCAGTCTTTGTTTTTTTTCAAGTTTTTTTTTTTCAAAGGAAAAGGGGGGTTAATCCCATGGGGGGGAGTTTAAATTCAAAAAAGGGATTTTTTGTTCCTTATTTTTTTTTTGTTGATTTAGGAGGAATTTTTTTTTTTTTTTTTTTTTTTTTTATGTTTGTTTTTGTTTTTCATGATGTATTAATAGATCCTGATAATTTTATTCCTGCAAATCCAATATCTACTCCTCCTCATATTCAACCAGAATGATATTTTTTATTTGCCTATACTATTCTTCGCTCTGTTCCTTCTAAGTTAGGAGGGGTAGTGGCTTTGGTTTTTTCTATTTTATTTCTGGTTTTTCTTCCATTTATTTCTTTATCTAAATCTTTGAAGATTCGAACGGGGTGAGAGTATAAATTTTTGGTGGTTGTTTTGTTTGTAGTATTTTTTTTGTTAACATGAATTGGTTCTATACCTGTAGAATATCCTTATGAGTTAGTTGGTAAGGTATTATCTGTTTTTTATTTTTTTGTTTTAATATTGATTATTGAGGTAGTAGAAGTTTTATTAATGGTTTTATTAAGAGTAGCTTTTTTTTCTTTGTATGAGCGTAAGTTGATAGGGTTAGTGCAGGGTCGAAAGGGTCCAAATAAAGTGGGGGTGGGGGGGGTTCTTCAACCATTTGCTGATGCTATAAAATTAATTAGGAAAAATGAATATGCTCCT---AGGAAGGTAATTAAATTTATTTATGCTGTTTCTCCTATAATTTCATTTTTTATTTCGTTGGTTTTTTGAATTATATATCCTGTGATTTGGAATTTTTTTTCC------TTAAATTTAAGGATTGTTTTTCTTTTAGTTTTGTTTAGAGTTTCGGTATATGGGTTTATTCTTTCTGGGTGATTTAGTTCTTCTAAATATGCTAATATTGGGTGTGCTCGGGCTTTAGCTCAGTCTATTTCTTATGAGGTTGGATTAACTTTGAGGATTATCTTTTTTTGTTTGTTTTTTTCTTGTATTTCTTTGTCAGAGATTTTAGAGGTTCAAGAGTTTTTTTTTTTTTTCCCTTTTTCCTTT------TTATTTGTTTTTATATGGAGGATTTTTTTTGCGGAAACAAACCGTCCCCCTTTTGATTTAGCGGAGGGAGAGAGTGAATTAAAAAAGGGGTTTTGTGTAAAGAAGGGGGAAATAAGGAATCCTTTAATTTTTTTAGGGGAAAATTTATCTGTTTTGTTTGTAAGGATTTTGTTAAAAGTTTTTTTTTTTAGAGGGTCGTTTTTTTTTTTATTATTATGGGGAGGCTTTTTGTTTTTGTTCGTTCTACTCTACCTCGATTTCGGTATGATAAATTGACGTCTATATTTTGGATTGAAGTATTGCCTTTACAATTGTTCTTTTCAGTTCTTTTTCTTATTTTGGTTTGCGAGTGTGGGTATTGTAATTATCTTTTTTTTAAGT------------------------TTTATTAGAAGAATATTTTGTGTAAATGAAAAAACA---GGAATAAATTTGGAAATGTATGAATGTGGAATTGAACCGATTCAAGAAGATAAAGCTCCGTTTTGTATACATTTTTTTTTAGTTGGGGTGTTGTTTCTTTTATTTGATGTTGAATTGATTGTTTGTATTCCTATAGTATGAATG------AGTGTGTATGAAAAGGTTTGAGGATTATTA---TGATTTGTGTTTTTTTTTATTATTTTTGTTGGGTTAGTTTTAGAAATAGTAATGGGGACGTTTGATTGAAAATTTGTAATAATTTTAATTTCTTTTGAAATAATAACATTAACAACTTTTTTGATAATGTTGTCTAAAGTGTGAATT---TTATATAATATTCATTTTTTCACTTCTTTATTAGTTTTTTCTGTAATGGAAGGGGTTTTGGGGGTGTCTATTTTGGTGATATTATTTTCTAATTCTAAGATTTTTTGTGAGAGAAATTTTGAAGTATTGTTAGTTGTTGATTCATTTTCTATAATTTTTTTGTTTACTGTAGGAATGGTTAGGAGATTTGTTTTACTTTATTCTAATTATTATATGATGGGAAGATTGTTTAAAAAGAAGTTTATTTTAGTAATGATGATTTTTATTCTTTCTATATTTGTTTTAAGGTTAAGTGGAGATTTATTTTGGGTAATGATTGGATGAGATGGATTGGGATTTTCTTCTATGTGTTTGATTTTTTTTTTTCAAAATTGGAAAAGGTTTAATAGTTCAATGGTAACTTTTATTTCTAATCGAATTGGGGATTTTTTGATTATCTCTTTTTTTTGTTTTTCTATTTTATTTAATGGG---AGATTATTTTTTGAGAATTATGTTTCCTCT---------------------TCTTTTTTAGGATTGTTTTTGTGTTTTGGTGCTTTGAGAAAGAGTGCTCAAGTTCCATTTTCGGCTTGACTTCCTTTGGCAATAGCTGCTCCAACTCCTGTTTCTTCTTTGGTTCATTCTTCTACTTTAGTTACGGCAGGGGTTTTTTTATTAATTCGTTTTAAGAGT------TTTCTTTGTGAGAAATTATTTAGAGTA---ATTTTTTTGGTTAGCTTTATTACTATTTTTTTGGCTGGGATAAGATCAGTGGGGGAATATGATTTAAAAAAGGTAATTGCTTTGTCAACTCTTTCTCATATTGGATTAATAATAATGTTTGTGGGGATGGAGAGATTTATTTCTGCAAAGATTCATTTAGTAATTCATGCCTTTTTTAAGTCTCTTTTGCTTTCAACTTTTAGGATGATAGGAGCACCATTTTTTTCTGGATTTTTTTCGAAAGAAATT---CTTTTAGGA---TGAGTATATGAAAGGACGGTTAGAATGGTAAGAGTATTATTATTTTTTTTTTCCGTTTCTTTAACTTGTTCATATTCGATTCGTATGTTATTTTATTTTTTTTTTCCCTGTTTT------------------TCTTTTTCTTTTATAATTCCAAGGAGTGAGTATCCCTCTAAGATGTTTTTTTTTATAAGAGTTGTTTCTTCTTTCTTAGCAGGAGTGATGGGGTTTGTTCTAATTGGG------------------AAGGAGGAAAGAGAG------ATTTCTTTTACCGTATTTTCTTCTTTTCGGAAAGTGGGGTTATTAGTAAGGATTGCTATTGGATTTTCTTTTTTATTTATTAGTTTG---TTTTTTTGGTGGTTTTCTTTGTCGAGGAGATTGTCTTTATTGATGGTGTTAGTTTTTTCTGTGATTAGAACGTTGAGTTTTTCTTTTTTTTTAGAGTTTAAGGTCCCGTTGTTTATAGTGGTTTTAACTCTTAGAGGGGGTTTGTTTGTGTTGGTTTCTTTTGTAATCATGTTTTTTCCT---GATGATTATAAGTTTGGGGTAACTTATGCGAAAAAAATCTTT---------------------TATTCTTGTTTATCAATAGTAATATTAGGGATGTCGATGTCTTTTTTT---------------------------TGAAGGGGAGATTTGTTAATAGGGGAGGAATTGGTTGTTGGTGGGTTTAAGGTTTGGGGA---------------------------------------------------AGATTTATTATGGTTTATTTTTTTTTTATTTTATTACTTTTTTTGTTTTTATTTGTAGTTAATAGGGTGGTAAATCTTAGAACTGGGGCGGTTAGACATCCGTCGGATATCAAGAAAA---------------------------------TAAATTCGGTTCACATTCATTTACTAAATTTATTTT--TATATTCGGTAAAATGATAGTGGGAATTAGTTTAAGCATGGGGTAGAGACCAGGATTAGATACCCTGTTATTCTTTAGAATAGGAATA----------------TAGTGAGCAGATTAAGTGTGG--------AAAAGTAAAAAACA-TGGCGGTTTTCT----ATCATATTAGAGAAACATGTGCT--GAAAACGAATTTGCACGAAGAATCATTCTTTTTCTTGAAACCCTATGTTGGG---TAAATTT-----CATTTTGCATACCGCTGT---CTGAAAGACTTTT-----CTATAGAAAATGTCTCA------------------------TATCACTAGGAAATTTT------------TAATTTGTTGAAATAAGGCAAGTAAAGGTGTAGATTATGAAGA-AGAA--AGCATGTGTCTCAGTTTG-----ATTAGT--------------GGAAAGAATAAGTGAAACTTTTC-----TTGAAAAAGAATTTAACAGTAAA-GTGAGTTTATTAAACTTGTTTGAATAGGAA--ATGGAAAGTGTACAAATTGCCCGTCACTCCAGTGGAGAAGTCGTAACATA-GTTGGACTACTGGAAAGTGGTACCTTTTGTATCAGGGTGGGAGGATAAAA--GTTCATTATTATGAAG------CTTCCCGAAAA--GA-AAGGATTTATTTCTGTTTTATTTTTTCA-GTTGCA-ATTGGATTTTAAAATCAGAAATGGAAA-TGAGAAGTT-TTTCGTCTTTCGTAT-ATCTGGTAACTCGGCAAAAGAATTGGATCCTGCCTGTTTAATAAAAACATCTCCTCAATTT--GA-TTATTTGAGGTATTGCCTGCTCAATGC-----GA-AAGTAAATAGCCGCAGTATTT-TGACTGTGCAAAGGTAGCATAATAAATTGGCCTTTAATTGGGGTCTAGGATGAATGGCTTGACAAGGATTTAACTGTCTTAGGGAGAAATGTTTT--GAAATTTAATTGTTTGTAAAAATGCAGACATGTGAGAGAGGGACGAGAAGACCCTATAGATCTTATTTATTTGGGGAAAATCCTGAAATGTATTAAAACTTCGGTTAATAAAAAAATTTAATAAT----TAATTTTTGATCCATTTGTAGAGTGAAAAAATGAAAAAGTTACCTTAGGGATAACAGGA--CAATGATTGTCTTAGAGGCCTTATTTA-AGACAACGTTTGTTACCTCGATGTTGAATTAAG-TTATCTAATTAAAGGAGAAATTTTT--ATAGTTG-GTCTGTTCGACCATTAAAAACTTACATGATTTGAGTTTAGACCGACGTAAGTCAGGTCAGATTCTATCTTCTC-TTTAGTTTATTTCTTT---TT-GTACGAAAGGAT

Haematomyzus_elephantis ATGTCTAGATTTGACCCGTGCTCGAGGGTAACTGGATTA---ATAAATTTATCAGAGAAGTGAATATTTGCTTCA---------------------TCAGGTATCATTCTATCAGGAAGAGTATTCTGACTCTGCCCAAGAGGGGTTCATTTTTTTATTGGCAAACTTGCGAAAGCTTTAACAAGTCTAACGCAACAAGATGTCTTTCATTTTAAA---------AGAGTGGCATTAGCCTGCTGTGCAATTTTTATTAATTTATGTGCAATTAACATAGTGGGTCTTTTACCATATTCTTTTCCACCTTCTAGTCACTTGACATTTAATTTGGCCTTAAGTCTGCCTTTATGGGCAGGGGGTTTGATGTACTCA---ATGAGAACTTCATTGAAGAGGTTTTCTTCTCATTTTCTCCCTGAAGGAAGGCCATTACCCCTCTCCCCCTTTCTAGTAATCGTCGAGATTATCTCTAGACTTATTCGGCCATTCTCTTTAAGAGTTCGGCTTATATCTAACATTATAGCAGGCCATATAATTTTGACTCTTATAGGCCAAGCTGCTGCTTCTTCTACCTTTGTTGTAATTCCA---GCTACGTTGACTCAAGCTGCCTTTATTGGATTTGAACTTGGAGTTAGGGTGGTTCAAGCTTTCGTTTTCATAAACCTGCTTTATCTATATTGACTTATGTTCCCTAGTTGGTGATTATTATTAATGATATTTTCTCTTATTGTTACTTTTGCAGTTATTTCA---ATTGTATTTTTTATGGTGAGGGAGGTTCCAGTTCTTGAGAACTCACGTAACTCA------TCAGCTCAACTTAAAGTGAGATGTCTGTTGTTTTCAACTAATCACAAGGATATTGGAATTTTGTATTTGCTTTTTGGAGCATGGTCTGGAGTTGTAGGTTTCACCTTAAGAATGTTTATTCGTATGGAATTAGGAGAGGCTGGTAAATTAATTTCTGATAGGCACATTTATAATGTCATTGTGACTTCTCATGCATTTCTAATGATTTTTTTTATAATTATGCCAATTATAATTGGAGGTTTCGCCAATTGACTAGTTCCAGTTATGCTGGGAGCCCCTGATATAGCTTTTCCTCGGCTCAATAATATAAGATTTTGGCTTCTACCGCCATCTTTATTCATACTTCTGTTGAGGGGGTTTGTTGGAGATGGAGTAGGCTCGGGGTGGACGGTGTATCCTCCTTTATCTAGAAGGGTAGGTCACCCAAGAGTGAGGGTTGATATTTCAATCTTCTCTTTACACCTTGCAGGAGTTAGATCTATTTTAGGGGCTATTAATTTCATTACAACTATTGTTAATATGTGAAGATTCCCTGCAAAATTTGAATTAATACCTTTGTTTTGCTGGTCAGTTTTAATTACTGCGGTTCTTTTACTTCTCTCTCTTCCGGTCCTTGCAGGGGCGATTACTATGCTGTTACTGGACCGTAACGCTAATTGCTCATTTTTTGACCCTTCAGGAGGGGGGGATCCAATTTTGTATCAACATTTATTCTGGTTTTTTGGTCATCCAGAAGTTTATATTTTAATTCTTCCAGGATTTGGACTAATTTCTCATATTATTGCTGAAGAAAGTGGGAAGAAAGAGGTTTTTGGTAGGCTGGGCATAATTTATGCTATAATTTCAATTGGAGTTTTAGGGTTTGTAGTTTGAGCTCATCACATATTTACTGTAGGTATGGACATTGACAGTCGTGCTTACTTTACAAGTGCTACAATGGTTATTGCTATTCCTACTGGAGTAAAAGTGTTTAGATGACTATCAACCTTATACGGCTCTACTCCATTCTACTCACCTGCCAAGCTTTGAGCAATTGGATTTGTATTTTTATTTACCGTCGGAGGGTTGACAGGAGTGGTTTTAGCCAATTCGAGAGTTGATGTTGTTTTACACGACACTTACTATGTAGTTGCACATTTCCACTACGTCCTCTCCATGGGAGCAGTGTTTGCTGTATTCGCAGCGTTTATTCACTGATTTCCTACAGTGACGGGAATTTCTCTTTCAAGTTCACTACTTAAAGCCCATTTTCTAACAACATTTATTGGTGTAAATGTTACTTTCTTCCCTCAGCACTTTTTGGGGCTGATAGGAATGCCTCGGCGATATAGGGATTATCCAGATATTTTCTTTGCCTGAAATGTAATCTCTTCAGCAGGAAGTGTGATTACCTTGAGAAGAGTAGTCTTATTCTTTTTTTTATTGTGAGAAGGGTTTTCATCGTGTCGAAAACTTACATTTAATAGGGCCTGCCCATCGTCTTTGGAGTGATTTATAGGAGGACCACCTTCTAGTCACTTGTACGAACAGGTGCCTGTGTTAGTTTATATATCATTATCTGACAGGCTTTCGCCTAGAATAAAATTTATTAGAAAAACTCATGATTTCATTTTAATTGTTGTAATTATAATTATTTCTTTAGTGCTTTATTTATCTTATTTTTTACTATTTGGATCTGGCTAC---------AGTCGGAGGGTTGTTGGGAGAGAAGGCCTAGAAGTTTTTTGGGCTTTGGTACCAATATGCCTTTTAGCAAGCTTAGCAGTTCCATCTCTACATTGTTTATACTTCAGAGAAGAGAATTTTAATCCTTTAATATCAATTAAAGCTGTGGGGCACCAATGATATTGATCTTATGAATACAGAGATTTTGATAGAGTTTCC---------TTCGATTCTTACATAATA------AGGGATTTAAATGTCTGAGAT---GTTCGCCTTTTAAGAGTTGATCAGAGTGTGATTCTCCCGGTTCAAGAAAGAATTCGGGCAATTGTTTCATCTTCTGATGTAATTCACTCATGAGCTCTTCCCGCTTTAGGCGTAAAAGTAGATGCAGTTCCTGGACGTTTAAACCAATCCCTTGTTCGCAGAGAGAAGGTAGGAGATGTATTTGGGCAATGTTCTGAAATCTGTGGTAGATTGCACAGTTTTATGCCTATCTGTCTAAGATTTGTTCCTAAGCCTCAGTTCTTAAATTGAGTTAAAAAATTAGGATTCCACAGATTCCACATTGTTGATCAAAGGCCTTGACCTATTATTTTAAGGGTTGGGGTCATAACTTCTATTTCGAATACATTTATTTTATACTTGAGCGATTGTAACTTCATAGGGGCTGTAACTAGGTGGATCGCTACTTGCACTTGCGCAGCTTTGTGGTGACGAGATGTAATTCGTGAATCGTTTTTTCAAGGGTTTCACAGTCACTCAGTTATGGCAGGTCTAAGAATAGGATTCATTTTGTTTATTGCTTCTGAAGTTATATTCTTCATATCTTTTTTTTGAAGGTTCTTCTATGTTTCCTTAAATCCTGATATCGAATGTGGCAGGGTGTGACCTCCCGCAGGGGTTCAAAGGTTAAGGGCATTTAATGTACCTCTTTTAAATAGAATTCTATTGATCAGAAGAGGAGTGTCCATTACCTGAGCTCACCACGCTCTCGTT------ATGAGGAATATAAAAGAAACAGCAGTTGGATTAGGGATTACATTGATATTAGGACTTACATTTTCTGTTGTCCAATCTTTTGAATACTTACACACTAGATTTTCTATAGCAGACAGAGTATACGGATCTGTATTCTTTCTAACTACTGGGTTTCATGGAATTCATGTTTTAGTTGGTAGAATGTTTATTACTGTTAGATTGTGTCGAACTATTTTAGGTCAATTTTCGTGTAATCATCATGTTGGGTTTGAATTTTCAGCTTGATACTGACATTTTGTTGATGTTGTTTGGCTGTTCTTATTCATTTCAATATATTGATGATATATATGAAATTTTGGATCCCTTTTAGGACTTTGTCTCAGAGTGCAGCTTGTAACTGGTTTATTCTTAGCAATACATTATAACCCTTCCGTAATAAATGCTTTTGAGAGGGTTGTAAAAATTATAAATGATGTTAATTGAGGTTGAGAAGTTCGACTTCTCCATGCTAATGGAGCTTCACTATTTTTTGTTTGTTTATTTACCCACATTGGTCGGGGTTTATATTATAAAAGGTACTCAACTAACCCTCTTACTTGAAGGGTTGGGGTCATTATTTTACTGATAGTTATAGCTACAGCCTTTTTAGGGTATGTTCTCCCTTGAGGGCAGATATCTTATTGAGGGGCTACTGTGATTACTAATTTACTGTCAGCCATCCCTTACTTTGGGGAAGATCTTGTTTTATGACTATGGGGTGGTTTTAGGGTTGGCAGTCCAACCTTATCTCGGTTTTTTACTATGCATTTTATTCTTCCGTTTATTGTCTTAAGCTTGGCTATTACGCACATTGCGGCCCTTCACATCAAGGGTAGAAGAAATCCATTAGGGTTGTCGAGGGATTATGATAAAGTTCCATTTCACCCTTATTATTTGGTTAAAGATATAGTCGGATTTGTGTTAGTGAGATTCTTTCTTTTAATAGTGGTATTTACATTGCCGCACTTATTCATAGATCCTGATAATTTTTCGGCAGCCGACCCTCTGAAAACCCCTCCTCACATTCAACCAGAGTGGTATTTTCTTTTTGCATACGCCATTTTACGTTCTATCCCTAGAAAATTGGGGGGCGTAGTGGGTTTACTAGCAAGAATTTTAATTTTACTTGTTCTTCCGGTAATAGAA---AGAAAATTCTCTAGGAGACGGTTTGAGCCAATCTCAAAGGTTATATTCTGGTTCTTAGTAAGAACTTTTGTAACTCTTACGTGAGTTGGGATAATGCCTGTTGAACACCCCTTTGATTTAATTGGAAAGGTATTATCTGTTTTATACTTTTTTGTGTTTATTATTCAAATTCAAATCGTTATATTTGTTGTTTCTGTTTTAGTGAGTGTAGCCTTTTTTTCCCTTTATGAGCGAAAAGTTTTAAGGTTGATTCATATTCGCAAAGGGCCTAATAAAGTTGGAGTTCTAGGCCTATTTCAACCATTTAGTGATGCTATGAAGTTAGTTAGAAAATCCATCCACCCTCCAGTCAAAGTCGAAAGGAGTTTATTATTTAAAATGTCTCCTATTATTTTAATCACAATTGTTGTGATAGTATGAAGAGTAATACCG------ATATATGGT------TACTCTTCTGTTTGAAGGGGGTTATTCTTACTATTACTTTTCAGTTTAACGTCTTATGGCCCTATTTTTGGAGGCTGAATCTCCAATTCATGTTTTTCAGTAATTGGTAGAGTGCGAAGGGTGATTATAATAGCTTCCTACGAGATCACGTTGTCTTTTTCAATATTAAGACTTTTTTTAATAGGGAAATCATTTTCCTTGGAAATCAGTTTCAACATGATAGACCTGCCACTCACTATTTTCTCAGTAGCCCCTTGACTAAGTGTTAGGTTAATTATCTCTCTTTTAGCAGAGAGGGGTCGGAGACCTTTTGACCTTTCAGAAGGGGAAAGTGAACTAGTAGCAGGTTACACAGTGGAGTATGGGGGTATTGATTACACCTTAATCTTCTTGGGAGAAAATATGTCAACCCTATTAATATGTGTGATTGCAAGAATAGTTATATTTAAC---TCCCTGAACATTGTGAGGGTTTTTAGCACAATTTCCCTTGTT------------ATTTTTATTCGAGGAGTGGTACCGCGAGTTCGGTATGACCATATAATTTTATTATGTTGAGTGATTATTTTACCTATTTTAATCAGAAGGGTGAGGTTAATGGTTATTATATTTACCTGTTTAATCATAATA------------------------GTTGTGAGAGGTTTATTCTCAGGGTCCGAATTAAAG---CAAAGGTCAAATGAGCCTTTTGAATGTGGAATGGATGTTTTTATTAGTTCACGGACTCCATATGCTCTCTATTCATATTTAGTCTTAATTTTATTTGTTATTTTTGATATTGAATTAATTGTTTCAATCCCTCTTGTGTTTACA---AACTTATTAACGTGTGATATTTGAAGAATTGTG---TGAACCATCTATTCACTTTTTATGCTTATAGGCCTGACAGTCGAATTTTGACTAGGAAGGTTGACATCTTGTCCTGTAAGGTGTTTAGTTGGGTTAGAAATAATGTCAGTAATTAGATTTTTACTAATAAATTCATCTGGAATTTTCTTTATAAGGCAAGCAACATCCTTAATTTTAATTATTACATTTGTTGTCTGTGAGGGGGTTTTAGGTTTAAGTTTGATCTCACCTTTTATAAAAAGCACCTCTTCATTCATAGCAATGGATGTTTCTGTTTTAATTAAAGTTGATTATCTAAGGTTAGGGTTTTTATTTATAGTAAGGGTTGTATCCTTCACGGTTCTTATTTATTCAAATTTATATATGGCAGGTTCTCCGGATTTTAATAAATTCATTTTAATCTTAGTGAGGTTTATTGCCTCAATAATAACTCTTGCTTTCAGCGGTTCCTTGTTTTGAGCTTTTATTGGATGAGATGGCCTCGGGCTAAGTTCATTTTGTTTAGTTATATTTTATCAGAATTGAAAAAGCTTTAATAGAGCTCTAACCACCTTTATCATAAACCGGGTGGGTGACGCCTTCCTTCTATTAAGGTTGTGGAGGATTTTATCAATAGGCTCT---AATATCTCCATGTTTTCTGCGAGAGCCCCT------------TTTGAATACGTGGTTGCGGGGGTTAGAGGTTTAATTTGTGCTTGCTCTAAAAGGGCTCAAGTGCCATTTTCTGCTTGACTTCCTTTAGCAATAGCCGCCCCTACGCCAGTTTCTTCACTAGTTCACTCATCCACTTTAGTTACTGCAGGCATTTATATGATTATTCGATTTAAATCA---GATTTTTTATATTCAAATTATCAGATTTTCGTGCTGTCTTTACTTTCAGGAATAACAATTCTCCTTGCAGGAGTCAGTTCTCTCGTGGAGTTTGATTTGAAAAAGGTTATTGCTCTTTCCACTTTACTTCACATTGGAGTAATGATTAACTCTGTTGCCCTTCAAATATTTGACCTTGCCTTGTTTCATATAATGGCTCATGCTATATTTAAGTCTTTGTTATTTATGATTTCAAGAATAGTCGGATTCCCTTTTATGAGGGGGTACTATTCTAAAGATTTAATTATCCTATCTTCCTTTAGAGTAGGAAGCTTTATAAAGTTGTGAAGAATAATAACTTTAAGGTCAGCCATCTTATTCTCTTCGGCTTACTCCTTTCGGCTACTTCTATTTTTGTCACTTAATCGTAGAAAGTCAAGCCCATTGCCCATGTTTACTCTTAAA------AAAGAAGTTTCATCTATTGAAATAGTTAAATTTAGACTCTATTTTGGCATTATTCTAAGAGTCTCCTTAGGGAGAGTATTGGAACTACACCAAGATTTAACAAAATGGGCA---------------ATCCTTCCGTCTCTCGTGGAAGTCGCTCTAAAACTGTTTGTCTGGTTAGCCCTTTTTTTAGGTATTTTATTTTGATTTACCCAAATT---TTATTTTGATTGAGGTCA---AGAATCGTGACTGAATTAATTTCTCTGATTAGTTTTACAATTATTGTATCATTTTGAAGGTTTATAACATGAAGATCGGAGTTTGCCCCTCTTATATTTACATATGCAACTTTGGGTGGGCTTACAATTTTAACGACTTTTATAATTATATGAAGGCCTCGTTGA---AGTAAGGTAGGCCGACCTTTGCACTCTCTAAGTAAA---------------------GTAGTCGCATGATTCGTAGTAGTTGTACTTTTTCTATGTCTAACAGAT---AGAATTATGCTAGATTTAAATACAGGAGTTACCCTTCCTTGACTTTGAGTTAATACTAGCTGAATGGGAGTTCAAGATTGAAGCGCTGGAGTTTCGAATCTCCATTGAAGATGAATTGAATCTCATTTTGTATGACCTAATGTGTGTATCATTGGATTTATGCTCATGATTCTACTTTTTAGACTCTTAATTGTTGACTCTTCTACTTACGGAAAAGGTGGTGCGGTCATACATTT-----AGGTCAAG-TAGACTTTTGGTAGAGAAGG----------------TAACACGAT-TTTGTGTTCAATTTACCGTTAAAGT---GATAAAAGGTAAAATTATTAAACAAGTTGGT---------------AAACTAGGATTAGATACCCTACTATTCTTGTGAATT-ATTAA--TTTTCTCTAGAAATTAAATGATAT----AATCATT--------AAACCTATGCAGGAGTGGCGGTTCTT----GGCCCAATCAGAGGCTTATGTTT---TAAATCGAAATTACACGAA-CATCTTACCAC---AAA------------------TTATATT-----GTCCTTGCACACCGCCGT--CGGAAGGGAAAGAG-----TTAAA----------TC------------------------TGAACCTTTATCAGCACTCATATGTGGAATACGTGGCTGAACTAAGACAGGTCAAGGTGCAGGTTATATTTATGGGGAAAATGTAAGTCATTATTTG-----AGTTATA-------------TAAGTTTATGAGATGAAATTTCCCATAATTGTTATAGAATTTGACAGTAAA-TGGAGTTTAAAATACTGCATTGAACAGGGT--CTCAAGAGTGTACAAATCGCCCGTCGCTCCCTTGGATAAGTCGTAACAAA-GTTGCACTACCGGAAGGTGGTACCTTTTGTATCAGGGTTTGAGGAATAA---AATAGTTCATTTACAA------CTTCCCGAAAG---GGGGAGATCTTTGATGGGAGTCAAGGTTGTTGTTACATTACCATCTG-CAATCTCATTAAAGTGG-TGAAATGCCTTTCGCTCCTTCTGAT-ATCTGGTAACTCGGCAATTCAGA---GTTCAGCCTGTTTAATAAAAACATGTCCTTCTGGGCATTTACATAGAAGGTCGGGCCTGCCCGCTGCACCCTTGAT-GTGAAGGGCCGCAG-AACTATAACTGTGCTAAGGTAGCATAATAATTTGCCCTTTAATTGAGGGCTAGAATGAATGGTTTGACTAGATCTCACCTGTCTCAGTTAAATGAGATTT--AAACTTAAACTTTAAGTGAAAATGCTTAAATGTTAGGAAAGGACGAGAAGACCCTGTAGATCTTGTTTGTCTGGGGCGGATGT--------CAATAACATGATT----GAATATTAAAACATGATTGTTGAACTATTAGACCCTCCACAGCGAGGGCTTATGATAAAGTTACCTCAGGGATAACAGCGCATGATATTTTCTTTTAAGATCATATTGAATAGAAAAGATTGCGACCTCGATGTTGAATTAAGCTTC-CTTTCTGAAGTAAAAGGTAGA--AAAGTTA-GTCTGTTCGACTATTAAAAGCTTACATGATTTGAGTTTAGACCGACGTGAGTCAGGTCAGATTCTATCCTTCC-TATTGAGAATTCATCTTGTCTAGTACGAAAGGAT

Psococerastis_albimaculata TTTTCTGTTTTCGATCCATCTACAACTATCTTTAAT------TTATCCATC------AATTGATTAAGAACATTA---------------------CTAATCTTTATTATATTACCCCTCATCTTTTGGTTAGTACCGACTCGATTGAATTTAGTCTGAATAAAAATTATTTTCACTTTACATAACGAATTCAAAACCTTAATTGGTGAAAATAAATTAAATACAGGAAACACCATTATATTTATTTCCCTATTTTCATTAATTCTATTTAATAACGTCTTAGGACTTCTACCTTATATTTTTACTAGAACTAGTCATATATCAATAACATTAGCTCTTTCTTTACCCTTATGAATCGCGTTCATACTTTTTGGT---TGAATTAATTTTAGACAACATATATTCAGCCATCTAATCCCACAAGGAACCCCTGCAATTCTTATACCTTTTATAGTATGTATTGAAATAATCAGAAATATCATCCGTCCAGGAACTCTTGCCATTCGACTGTCAGCAAATATAATTGCAGGTCACCTATTAATAACTCTTCTAGGTAACACCGGTCCTAGTTTATCCCTTATTATATTAAATATCCTTATCTTCACCCAAATTCTTTTATTAACCTTAGAAACAGCTGTAGCTTTCATCCAATCATACGTATTTGCAATTTTATCCACTCTCTATTCAACAATAAATCCTATATGATGATTCTCCTTATTTATTATATTTGTAACAATTCTACTGTCTTCTAATTCA---TTAAATTATTTT------TATTCAAAAAATGCTCTAGATATTAATTCTTTTAAA------AAAAGAAATATTAAAAATAACATTAATTTATTTTCTACAAATCATAAAGATATTGGAACATTATATTTCATTTTTGGTATTTGAGCTGGTATAGTAGGGTCAAGTCTAAGTATCTTAATCCGGTTAGAATTAAGACAACCTGGATTATTTTTAGAAGATGACCAAACCTATAATGTTATTGTGACTGCTCATGCATTTATTATAATTTTCTTTATAATTATACCTATTATAATTGGTGGATTTGGAAATTGATTAATCCCACTAATACTAGGAGCTCCTGACATAGCATTCCCCCGAATAAATAATATAAGATTTTGATTATTACCCCCCTCTTTAACTCTCCTAATTTCAAGAAGCTTAGTAAATACCGGGGCAGGTACAGGTTGAACTGTTTACCCTCCGTTAGCAAGAACATTAGGACACCCTGGAGCATCTGTCGATCTAGCCATTTTTTCTCTTCATCTTGCTGGAGTAAGATCAATTCTGGGAGCAGTAAATTTTATCACTACAATTATTAATATACGATCACAAGGTTTATCATTCGAACGAATACCTCTATTGGTTTGGTCTGTTTTAATTACAGCTGTTTTATTACTTTTATCATTACCAGTATTAGCTGGTGCTATCACCATACTCCTCACAGACCGAAACCTAAACACCTCCTTCTTTGACCCCGCAGGTGGAGGAGACCCTATTCTTTACCAGCACTTATTCTGATTTTTTGGCCATCCGGAAGTATATATTTTAATTCTCCCCGGATTTGGCCTGATTTCTCATGTAATTAGACAAGAAAGAGGAAAAAAAGAAACATTTGGGGTTCTTGGAATAATTTATGCTATAATGGCTATTGGACTTTTAGGATTTGTTGTATGAGCACATCATATGTTTACTGTTGGTATAAACGTTGATACACGAGCCTATTTTACTTCTGCTACTATAATTATTGCTATTCCTACAGGAATTAAAATTTTCAGTTGACTAACAACCCTTTACGGGGCAAATATTACTTTTACTCCGTCAATCCTTTGGAGATTAGGATTTGTGTTCTTATTCACAATCGGAGGATTAACTGGGGTAATCCTGGCCAATTCATCTATTGATATTGTTTTACATGACACCTATTACGTTGTAGCTCATTTCCACTATGTTCTTTCTATAGGAGCTGTATTTGCTATTATAGCCGGTTTTATCCAATGGTACCCGTTATTTACAGGAATAGTATTAAACGAAAAACTTTTAAAAATTCAATTTTTCATTATATTTATTGGTGTAAATATAACCTTTTTCCCACAACATTTCCTAGGCTTAAGTGGAATACCCCGACGGTATAGAGACTACCCTGATGTATATACCTCATGAAATGTAGTCTCTTCAATTGGAAGAATAATTTCTTTAATTGGTATTATCTTTTTAATTTATATCATTTGAGAAAGATTTATA---ATACGCTTACCCTCATTTTATAATTATTCTAACTCATCACTAGAATGAACACAAAAGTTACCTCCTTCGGAGCATTCATACTCTGAATTACCTATAATTTTCAATTTAAATCTTCAAGAAAGTTCTTCTCCTTTAATAGAACAATTAAACTTTTTTCATGATCACTCCATCTTAATCTTACTAATTATTACATGCTCTATTTCATATTTAATAGTTGCAATAATTTCAAATAAAATCACA---------AACCGATTTTTAATAGAAAATCAATTAGTGGAAATGATCTGGACAGTTATCCCTGGGGTAATTTTAATTTTTATTGCCCTACCTTCTCTACGAATCCTTTACTTATTAGATGAAGTGACCGCTCCATCCTTAACCCTAAAAACTATTGGCCATCAATGATACTGATCATACGAATATTCAGACTTCATAAATGTGGAA---------TTTGATTCTTATATAGTCCCTCCTTCTGAAAATAATCAATCAGAC---TTCCGCCTATTAGAAGTAAATAATCGAATTGTATTACCTTATAATACACAAACCCGAATTTTAGTTACAGCCGCAGACGTTTTACATTCATGGGCAATACCTTCACTTGGAGTTAAAGTAGACGCTAACCCTGGCCGTATTAACCAAACAAGCTTTTTAATTAACTACCCCGGCTTATTCTACGGCCAGTGCTCAGAAATTTGTGGATCTGTACACTCATTCATGCCAATTGTACTAGAAAGAACAACTAAAAATAGTTTTATTAACTGATTATTATTAACACAAAATCACCCTTATCATTTAGTCAATGTAAGCCCATGACCTCTTACAGGAGCTATTTCTGCTTTATTTATAACTTTAGGAATAGTTGAATGATTTAAT---ACTCCCCAAAACTACCTAATAAAAATAGGACTTATCATAATAATTCTAACTATAATCCAATGATGACGAGATGTAGTGCGAGAAAGAACCTTCCAAGGAAATCATTCATTCAAGGTATCCCGAGGTATACGCTGAGGAATAATTCTTTTTATTACATCAGAAATTTTCTTCTTCGTATCTTTCTTTTGAGCATTCTTTCATTCAAGATTAGCTCCTAATATTGAACTGGGAATTATATGACCCCCAAAAAGAATTACCCCTTTTAATCCACTACAAATTCCCCTTTTAAACACTATTATTTTACTTTCCTCCGGAATTACTATCACTTGAGCCCATCACGCTCTATTA------AAAAATAATTATTCACAAACAATTCAAGCCATATTAATTACAGTAGTATTAGGAATTTACTTTACTATCCTTCAAGGGTATGAGTATGTTGAAGCTTCCTTTTCAATTGCAGACTCTATCTACGGATCATCTTTTTTCATAGCAACAGGATTTCATGGAATCCATGTAATTATTGGAACAATTTTCATTTTAATAATGCTAATTCGTCAATATAAACCTCACTTTTCTAATATACATCACTTCGGCTTCGAAGCCGCTGCTTGATATTGACATTTTGTAGATGTAGTCTGATTATTTCTATATGTAACAATTTACTGATGAACAATATGAAATTTTGGTTCACTTTTAGGTCTCTGCCTTGGAATCCAATTAATTACAGGTATTTTTCTAGCTATACATTACACAGCCGACATTAATATAGCATTTTCAAGCGTTGTACATATCTGTCGTGATGTTAATAATGGGTGACTTCTCCGAACCATCCATGCTAATGGAGCCTCGTTTTTCTTTATTTGTCTTTACACCCATATCGGTCGTGGTATTTATTATGGTTCTTATAATTTATTA---TTTACATGAATAATTGGAGTGATTATTTTATTTCTAGTTATAGGTACAGCATTCATAGGGTATGTTTTACCTTGAGGGCAGATATCATTTTGAGGAGCAACCGTAATCACAAACCTAGTTTCAGCCATTCCCTATCTAGGAACATCAATTGTACAATGAATTTGAGGGGGATTTGCTGTTGACAACGCTACATTAACACGATTTTTTACTTTCCATTTTATTTTACCTTTTATTGTTTTAGCTATAGTAATCATCCATTTATTATTTTTACACCAAACAGGCTCTTCAAACCCTTTAGGCCTAAAAATAAATATTGATAAAATTTCTTTCCATCCTTATTTTTCATTCAAGGATATTTTAGGCTTCTTAATTATACTCCTAATCTTAACATTGTTGACATTAATAGACCCTTATATATTAAGAGATCCAGACAACTTCATTCCCGCAAATCCTTTAGTAACTCCTGTTCACATCCAGCCCGAGTGATATTTTTTGTTCGCTTACGCAATTTTACGTTCAATTCCTAACAAGCTTGGAGGTGTAATTTTTCTAATTCTATCAATCGCAATTTTATTTATTTTACCTTTTTCTCAAAAAAATTTAATTAAAGGATACCAGTTCTTCACTATTAACAAACTCTTATTCTGAGTCTACGTAAACTTAATTATTCTTCTAACATGAATTGGAGCCCGCCCTGTTGAAGATCCTTATATTTTTATTGGGCAATCTCTAACATTAATTTATTTTCTTTACTTTTTAGTTAACGTGTCTTTTTTATTGATAATAATTTTAGTTTTAGTAGGGGTTGCGTTTTTGACATTATTAGAACGAAAAGTTTTAGGATATATTCAAATTCGAAAAGGTCCTAATAAGGTTGGGGTGATTGGTATTTTACAGGCTTTTAGAGATGCAATTAAATTATTTACTAAGGAAATAACGTATCCT---AATAAGTCTAATTATTTAATATATTACTTTTGTCCTTTAATTTCTTTTCTGTTGATTTTTATTATTTGAAGTGTCACCCCCTTTTTATATGTTATAATAAAT------TTTAATTTTGGATTTTTATTTTTTATGATGTGTTTGAGAATAGGAGTATATGGAATTATAGTGGCTGGGTGGTCTTCTAATTCTAGCTATTCTTTATTAGGAGGACTACGGGCAGTGGCCCAAACTATTTCTTATGAGGTAAGCTTGGGGTTTTTAATAATAAATATGCTTATTTTATGTGGGAGTTATAGTTTTATAGATTTTTTTTTTGGTCAGTATTATTGTTGATTTGTATTTACGAGATTT---CCTTTATTTTTTTTGTTGTTTACTTCTATACTAGCAGAAACCAACCGCACTCCTTTTGATTTTGCTGAAGGAGAGTCTGAGTTGGTCTCTGGTTTTAATATCGAGTACAGAAGTGGTGGGTTTGCCTTAATTTTTTTGGCAGAGTATTCGAGAATTTTATTAATAAGACTGGTTTTAGTATTGATGTATTTAGGGGGTGACACAACTGGGATACTATTTTTTTTTTTAGTAAGATTTATTTCTTTTTCTTTTATTTGGGCTCGAGGTACTTTACCTCGATATCGATATGATAAATTAATGAATGTATGTTGAAAGAGTTTTTTGCCTGTATCTTTATTATATTTAGTCTTTATTTTAGTCGCCATTATTGCAAACCTAATAATA------------------------ATTTTATGTATAATTTTATCAAAAAAATCATTATATGAACGAGAAAAATTTTCTCCCTTTGAATGTGGATTTGACCAAAAATCATCGTCTCGAATACCATTTTCTTTACGATTTTTTTTAATTACTATCATTTTTTTAATCTTTGACGTAGAAATCGCACTCATTTTACCCGCAATTAACCAAATTAATTTATCAACAGCAAATCAATGATTACTAATA---AATACTTTATTCTTAATTATTTTATTAATTGGTCTTTTTCATGAATGAAACCAAGGAGCACTGGAATGATCTTTACTGGTTATATTAGTAAGTTTAGAATTTATAAGACTAATTATTTTTTTTATATTAATGTTTAATTTGTGGATGTTTAGT---GAGAAATATATTTTAATATATTATTTAACTTTCTGTGTTTGTGAAGGGGCATTTGGGCTATCTTTATTAGTGTGTTTAGTTCGGTCTGTAGGTAATGACTATGGGGTGGGGGTAAGAATATGTGTTTATATAGATTGGATATCTAGATCTTTCATAAGATTCGTTTTATTGATTTCTTTTGTTGTTGTTTGTTACAGAATTAGTTATATAGGATCTGATAAATATTCTAGAATATTTATTATATTAGTTTTTTTTTTTGTATTGTCAATAATGTTGTTGATCATTAGACCTAATTTAATTAGAATTTTATTGGGATGAGATGGGTTGGGATTAATTTCTTATTGTTTAGTTATTTATTATCAAAACATTAAATCTTATAATGCTGGGATGGTGACAGCGATAACTAACCGTATTGGAGATGTAATACTTTTAATGGGAATTGCTTGAATAATTAATTTTGGTAGT---TGAAATTATTTATTTTATTTTAATTATTCT---------GATAATATATTTTATATTATTGGAGTATTTATAGTATTGGCTGCAATAACTAAGAGTGCACAAATTCCTTTTTCTTCTTGACTACCAGCTGCAATAGCAGCTCCTACGCCTGTTTCTGCGTTAGTTCATTCATCAACTTTGGTTACGGCAGGTGTTTATTTATTAATTCGATTTGATTAT---ATTTTTAAATATAGTTTTATTTATAACTTT---ATAATTGTAGTTTCTGTTCTGACCATATTTATATCAGGGCTAGGGGCTATTTTTGAATATGATTTAAAGAAAATTATTGCTTTATCAACATTAAGTCAACTTGGACTAATAATTAGAACTTTATGTTTAGGGATAACTGAATTTTGTTTTTTTCATTTGTTGACTCATGCTTTATTTAAATCTCTTTTGATATCTAATTTATCTTTATGTGGAATACCATTTTTAGCTGGGTTTTATTCTAAGGATTTG---ATTTTAGAA---AATATTTTAATGTTTAATATGAATAGACTTATTTATTTTTTATATTTTTTATCTACAATGTTAACAGTTGTGTATACATTTCGATTAATTTATTTTAGTATAGTTAATTCATTT---------------AAATTAATACCTTATCAT------TGTTTTAATGATGAAGATTATTTAATACTTTTTAGTATAATTGTAATAGTTTTTATAGTTATTATTGGAGGTTCTATAATAATGTGA------------------CTAATGTTTATAGAT------TTTAGAGGAATTATTTTAGGGTTGTATTCTAAACTATTGACTTTGGTAGTTTGTCTAACAGGGTCCCTGACTATATTTTTAAACATA---ATTTTCTTTATTTTAATG---ACCCCACTTTCCTTAGGACTAACATTAATTCTCCAAACATTATTACTGAGACTCCTATTAGGTACAATAACATCATCCTTTTGATTCCTTTACTTGCTAGTTTTAATCTTTATTGGGGGGATATTAGTATTATTTATCTATGTGACCTCTATTTTTCCA---AAT---GAAAAATTCTCATTTACACAAAACAACATTTTTATT---------------------CTACTTATCTCAGTTTCTTTACTAAGAACAATTTTATATATTCTAAAT------------------ATAAATTTTATTATAACCCCAAACTTAAACTATTTAGAAAATATCTTATCTATAAAGTCAAATACAATT------------------ATAATCAGAACTATT---AAAATTTTTAACACCCAAGCTAATATAATCTTAATTTTTTTAGTTAATTATTTGTTCTATTGTATAATAATTGTTATTAAAATAACAGCCTTTTTTAAAGGAGCGGTTACACATTC-----ATTTCTAT-TCAATATTATTAAAATTT------------------TAATTTT---TTGATTTTGTTTATTTAATTTAAAT---AATTTTAAGTGAAATATTTAAGAAAATTAAT----------CAGATAAACTAGGATTAGATACCCTATTATAAT------TAATGTAAATTCAATTTTGAGTAGTA------------AATGTTTAGGACAGGAAACTTAAAGAATT-TGGCGGTGTAT----ATTCTAATTAGAGGAATATGTTTTT-TAA-TTGATAATCCACGTT-TATCTTACTTTAA----------------------TTAATTT------ATTTTGTATATCTCCGT--CTTTAAAAAATTTT-----TTAAAATATTTTTTATT------------------------TT---------------------------TTATAATTTAAAATAAGTCAGGTCAAGGTGCAGATTATATTAA-AGTT--AAAATGTATTACAATAAA-----ATTATT------------TTTGGTTTAAGATTGAAATATGTTT-----ATTAAATTGGATTTGTAAGTAAAATTTTTTGTATAATAATTATTTGAATAAAGTT-AATATATATGTACATATTGCCCGTCATTCTTAGAGACAAGTCGTAACAAA-GTAGTTTTACTGGAAAGTGGTACCTTTTGTATCAGGGTTTAA---ATATA--AAAATTTATATAAGAT------TATCTCGAATG--GT-TTTGATTTAATTATATATAATTATTAAT-GTAGAAAAATTAATATTTAATATATAATTAGAAA-TGAAAAGTTAGTCGTTTAACCATAA-TTCTAGTAACTCGACAAAAGTAA---TTT-CGCCTGTTTATCAAAAACATGTCTTTTTGGTAA----AATATAAAGTCCAATCTGCTCACTGA-----AGAT-TTAAAGAGCCGCAGTATAC-TGACTGTGCAAAGGTAGCATAATCAATAGTTTTTTAATTGAGAACTTGTATGAAAGATTAGACGAGAATTAAATTTTCTTTATTCTACTAAATT---TAATTTTAATTTTAAGTGAAAATGCTTAATTTATTTTAAAAGACGAGAAGACCCTATAGATTTTTTTTAATTGGGGCGATTTTATAAAA-TAAAAAACTTAAATTTTTTATTACCATAAATTATTGAA---TTTTCTGATCTAAAAATTTTAAAGAAA-A-GATTAAATTACCTTAGGGATAACAGCG--TAATTTTATTTAAGA-GTTCATATCTA-TAATAAAGTTTGCGACCTCGATGTTGAATTAAG-ATAATATATTGGAGTAGAAATTAAT--ATAATTA-GTCTGTTCGACTATTAAATTCTTACATGATTTGAGTTCAGACCGGCGTGAGCCAGGTCAGTTTCTATCTTTAA-TTTTTTTTGTTTAGA----TAAGTACGAAAGGAC

Longivalvus_hyalospilus TTCTCTGTTTTTGATCCATCAACAACTATTTTTAAT------TCATCCATT------AATTGATTAAGTACATTT---------------------TTAATTTTTATTATACTACCCTTAATCTTTTGACTCGTTCCTACTCGATTCAATTTAATTTGAATAAAAATTATTTTTATTCTCCATAAAGAATTCAAAACTTTAATTGGACAAAATAAATTAAATCTAGGAAATACTATCATATTTATCTCCCTATTTTCACTCATTTTATTCAATAACGTCCTAGGATTATTACCATATATCTTTACAAGAACAAGCCATATATCTATAACCTTAGCTTTATCATTACCTTTATGAATTGCATTTATACTATTTGGA---TGAATTAACTTTAGTCAACATATATTTAGACACTTAATTCCACAAGGAACTCCTGCTATTTTAATACCTTTTATAGTTTGTATTGAAATAATTAGAAATATTATTCGTCCAGGCACTCTAGCTATCCGTCTTTCTGCTAATATAATCGCAGGTCATCTTCTCCTAACTTTATTAGGTAACACCGGACCTTCTTTATCTATATTCATATTAAACATTCTTGTTTTTACTCAAATTCTTCTTCTCACTTTAGAAACAGCTGTAGCCTTCATCCAATCTTATGTATTTGCAATCTTAACTACTTTATATTCAACAATAAATCCAATATGATGATTCTCTCTATTCCTTATCTTTGTAACCACCCTTACAATCTCTAACTCA---TTAAACTATTTT---------TATTCAAATAATTCTTTATCTTCCTCTTCTTTA------ACCAATAAAAATACAATTAATGTAAATTTATTTTCTACAAATCATAAAGATATTGGAACCCTTTATTTTATTTTTGGAATTTGAGCTGGTATAGTAGGCTCAAGCTTAAGTATATTAATTCGTTTAGAATTAAGTCAACCCGGATTATTTTTAGAAGATGACCAAACCTATAATGTAATCGTAACCGCTCACGCATTTATTATAATTTTCTTTATAATTATACCTATTATAATTGGAGGATTTGGAAACTGACTGGTACCTTTAATACTAGGAGCCCCTGATATAGCCTTCCCTCGAATAAATAATATAAGATTCTGATTATTACCACCATCCTTAACCCTATTAATTTCAAGTAGTTTAGTTAATACAGGTGCAGGAACAGGTTGAACAGTTTACCCTCCATTAGCTAGAACATTAGGACATCCAGGAGCCTCAGTAGACCTAGCAATTTTTTCTCTTCATCTAGCAGGTGTTAGATCAATCTTAGGAGCAGTAAATTTTATTACAACAATCATTAATATACGCTCACAAGGATTAACATTTGAACGAATACCTTTATTTGTTTGATCTGTCTTAATTACAGCCATTCTTCTTCTCTTATCACTTCCTGTTTTAGCAGGAGCAATCACTATACTTTTAACAGACCGAAACTTAAATACATCTTTTTTTGACCCAGCAGGAGGAGGTGATCCAATCCTTTACCAACACTTATTTTGATTTTTTGGTCATCCTGAAGTTTATATTTTAATTTTACCTGGATTTGGTTTAATTTCCCATGTAATTAGTCAAGAAAGAGGAAAAAAAGAAACATTTGGAGTTTTAGGAATAATTTATGCCATACTAGCTATTGGACTTTTAGGATTTGTAGTTTGAGCTCATCATATATTTACTGTTGGCATAAATGTTGATACACGAGCCTATTTTACTTCAGCTACTATAATTATTGCAATTCCCACAGGAATTAAAATTTTTAGTTGATTAACAACCCTTTATGGAGCTAATATTGCATTTACACCTACTATTTTATGAAGACTAGGATTTGTATTTTTATTTACAATTGGTGGTTTAACTGGAGTAATCCTTGCTAACTCTTCAATTGATATTGTTCTTCATGACACCTATTATGTAGTTGCTCATTTTCATTATGTATTATCAATAGGAGCCGTATTCGCTATTATAGCAGGTTTTATCCAATGATACCCTTTATTTACAGGTATAGTTTTAAACGAAAAACTTTTAAAAATCCAATTTTTCGTTATATTCATTGGTGTTAATATAACATTCTTCCCTCAACATTTCCTTGGATTAAGAGGTATACCCCGACGATATAGAGATTATCCAGATGTATATACATCATGAAATGTAATTTCTTCTCTTGGAAGAATAATTTCTTTAATTGGTATTATTTTCCTTATTTACATTATATGAGAAAGATTTATA---ATACGTACACCCTCATTTTATAATTATTCTAATTCATCTCTAGAATGAACCCAAAAATTACCTCCTTCTGAACATTCCTACGCAGAATTACCTATAATCTTCAATTTAAATCTTCAAGAAAGTTCTTCTCCTTTAATAGAACAATTAAACTTTTTTCATGATCACTCTATCTTAATTCTATTAATCATTACATGTTCTATTTCCTATTTAATAATTGCAATAATCTCAAATAAAATTACT---------AATCGATTTCTTATAGAAAATCAACTAGTAGAAATAATTTGAACCGTAATTCCTGGAGTAATTCTAATTTTTATTGCTCTCCCTTCACTTCGAATTCTTTATTTACTTGATGAAGTAACCTCACCATCTTTAACATTAAAAACTATTGGACATCAATGATATTGATCTTATGAATATTCAGATTTCCTAAATGTAGAA---------TTTGACTCCTATATAATCCCCCCCTCAGAAAATAATTCCACAGAT---TTACGATTGTTAGAAGTAAATAACCGAATTGTCATCCCTTATAATACTCAAACCCGAATTTTAGTAACAGCTGCAGATGTACTACATTCTTGAGCTATACCTTCTTTAGGAGTAAAAGTAGATGCTAACCCAGGACGAATTAATCAAACCAGATTCTTAATTAATTACCCTGGATTATTTTATGGTCAATGTTCTGAAATTTGTGGTTCAGTACATTCCTTTATACCAATTGTTGTAGAAAGAACAAATAAAAATAGATTTATTAACTGACTATTATTAACACAAAACCATCCTTATCATTTAGTTAATGTAAGACCTTGACCTCTTACAGGTGCTATTTCCGCCTTATTTATAACATTAGGAATAATCGAATGATTTCAT---TCTCCAGAAAATTATTTAATAAAAATAGGATTTACTATAATATTATTAACAATAATTCAATGATGACGTGATGTTGTTCGAGAAAGAACTTTCCAAGGAAATCATTCTTTTAAAGTATCCCAAGGAATACGTTGAGGAATAATTCTATTCATTACATCAGAAATCTTTTTCTTTATCTCTTTTTTTTGGGCATTCTTCCATTCTAGTTTAGCCCCTAATATTGAATTAGGTATTATATGACCACCTAAAAGAATCACCCCCTTTAATCCACTACAAATTCCACTTTTAAATACTATCATCTTACTTTCATCAGGAGTTACTATTACATGAGCCCACCATGCTCTATTA------AAAAATAATTATTCCCAAACAATCCAAGCTATAGTAATTACAGTAATATTAGGTATTTATTTTACTGTTCTTCAAGGATATGAATATATAGAAGCTTCGTTTTCCATCGCAGACTCCATTTATGGAGCCTCATTCTTTATAGCTACAGGATTTCATGGCCTCCATGTAATCATTGGAACAATCTTTATTCTAATAATATTAATTCGGCAATATAAGCCCCACTTTTCCAACAACCATCATTTTGGCTTTGAAGCAGCTGCTTGATATTGACACTTTGTAGATGTAGTTTGATTATTTTTATATGTAACAATCTATTGATGAACAATATGAAATTTTGGTTCTCTTTTAGGATTATGTTTAGGTATTCAATTAATTACAGGTATTTTTCTAGCTATACATTACACTGCAGATATTAATATAGCATTTTCTAGTATTGTTCATATTTGTCGTGATGTTAATAATGGATGATTACTTCGAACTCTCCACGCCAATGGAGCCTCATTCTTTTTTATTTGTTTATATATTCATATTGGACGAGGTATATATTATGGATCTTTTAATTTATTA---TTTACTTGAACAATTGGAGTTATTATTTTATTTCTAGTAATAGGTACAGCATTTATAGGATATGTACTTCCCTGGGGCCAAATATCTTTTTGAGGAGCTACAGTTATTACAAACTTAGTTTCTGCTATTCCTTATTTAGGTACATCAATTGTACAATGAATTTGAGGAGGATTTGCCGTTGATAACGCCACTCTAACTCGATTTTTTACTTTCCATTTTATTTTACCTTTCATCGTTTTAGCTATAGTAATTATTCATTTATTATTTTTACACCAAACAGGATCATCTAATCCTTTAGGATTAAAAATAAACATTGATAAAATTTCCTTTCATCCTTACTTTTCTTTTAAAGATATTTTAGGATTTTTAATTATATTATTAATATTAATTTTATTAACACTAATAAATCCTTATATATTAAGAGACCCTGATAATTTTATTCCAGCAAATCCTTTAGTAACTCCAGTTCATATTCAACCAGAATGATATTTTTTATTTGCATATGCAATTTTACGATCAATTCCTAATAAATTAGGAGGAGTTATTTTTTTAGTCTTATCAATCGCAATTCTATTAATTTTACCATTTTCACAAAAAAATCTAATCAAGGGATATCAATTTTTTACTCTTAATAAATTCCTATTCTGAACTTATGTAAATTTAGTTATCCTTCTAACATGAATTGGAGCACGCCCTGTCGAAGACCCTTATATTTTTATTGGACAATCATTAACGTTAATCTATTTCTGCTATTTCCTAATTAACGTATCTTTTTTGTTAATGATAATTTTGGTTTTAGTCGGAGTAGCATTTTTAACATTATTGGAACGAAAAGTTTTAGGATATATTCAGATTCGGAAAGGACCAAATAAAATTGGTTTTATAGGAATTTTACAAGCTTTTAGAGACGCTATTAAATTATTTACTAAGGAAATAACGTATCCT---AATAAATCTAATTATTTAATATATTATTTTTGTCCTTTAATTTCTTTTTTGTTAATTTTTATTATTTGAAGAGTTACTCCATTTTTATATTACATAATAAAT------TTTAATTTTGGTTTTTTATTTTTTATAACTTGTTTAAGAATGGGAGTGTATGGGATTATAGTTGCAGGGTGATCTTCTAATTCTAGATATTCATTATTAGGGGGACTTCGTGCTGTAGCTCAAACGATTTCTTATGAGGTAAAATTAGCTTTTCTTATAATAAATGTTTTAATTTTATCTGGAAGATATAGATTTATAGATTTTTTTTTTGGTCAATATTATTGTTGATTTGTATTTACTAGATTT---CCTTTATTTTTATTATTATTTACTTCTATATTAGCTGAAACTAATCGTACTCCTTTTGATTTTGCTGAAGGGGAATCAGAATTAGTTTCTGGATTTAATATTGAATATAGAAGAGGAGGATTTGCGTTGATTTTTTTAGCTGAATATGCTAGAATTTTATTAATAAGATTAGTTTTAGTATTAATATATTTTGGGGGTAATACAAGAAGAATTTTATTTTTCTTTTTAGTAAGAGTTATTGGTTTCTCTTTTATTTGAGCTCGAGGTACTCTGCCACGTTATCGTTATGATAAATTAATAAATGTATGTTGAAAAGGTTTTTTACCTGTATCTTTATTATATTTGGTGTTTTATCTTAACTTCTTTAATTGCAAACCTAATAAT------------------------AGTCTTATGTATAATTTTATCAAAAAAATCACTCTATGACGAGAAAAATTTTCTCCTTTCGAATGTGGATTCGACCAAAAATCATCCTCCCGACTACCTTTTTCTCTACGATTTTTCCTTATTACTATCATTTTCTTAATTTTTGATGTAGAAATTGCTTTAATTTTACCAGCAATCAATAATATTTATTTATCCAACGCAAGTCAATGACTAGTCTTA---AACACTACATTTTTATTAATTTTATTAATTGGGTTATTTCATGAATGAAATCAAGGAATCTTAGAATGATCTTTATTAATTATATTATTAAGTTTAGAATTTGTAAGATTAATTATTTTTTTTATATTAATAATTAATTTATGAATATTTTCT---GAAAAATATGTTTTAATATATTATTTAACTTTTTGTGTTTGTGAAGGTGCTTTTGGATTATCCTTATTGGTAATATTTAGTTCGTTCAGTAGGTATGATTATGGTGTAAGAATTAATATATGTATTTATATAGATTGGATATCGAGATCTTTTATGAGATTTGTTTTATTGATTTCTTTTGTTGTAGTTTGTTACAGAATTAGTTATATAGGATCTGATAAATATTCAAGTATATTTATTATATTGGTTTTTTTTTTTGTTTTGTCGATGATATTATTGATTATTAGACCAAATTTAATTAGAATTTTGTTAGGATGAGATGGGTTAGGTTTGATTTCTTATTGTTTAGTTATTTATTACCAAAATATTAAGTCTTATAATGCTGGTATAGTTACTGCGATGACAAATCGTATTGGGGATGTAATAATTTTAATGGGTATTGCCTGAATAATTAATTTTGGTAGA---TGAAATTATTTATTTTATTTTAGCTGTGAT---------GATAAAGTGTTTTTTATTATTGGTGTATTTATAATGTTTGCTGCAATAACTAAAAGTGCTCAAATTCCTTTTTCTTCATGACTTCCTGCTGCAATAGCGGCTCCTACTCCTGTTTCTGCATTAGTTCATTCTTCTACTTTGGTTACTGCTGGTGTTTATTTATTGATTCGGTTTGATTAT---ATTATGAGGTATAGATTTATTTATAACTTT---TTGCTTTTGGTTTCTGTTTTAACTATATTTATGTCTGGATTGGGGGCTATTTTTGAGTATGATTTAAAGAAGATTATTGCTTTATCAACATTAAGACAGCTTGGATTAATAATTAGAACCTTATGTTTAGGTATAACAAGTTTTTGTTTTTTTCACTTATTAACCCATGCTTTATTTAAGTCTTTATTGATATCTAATTTATCTCTTTGTGGAATGCCTTTTTTAGCTGGTTTTTATTCAAAAGATTTA---ATTTTGGAA---AATATTTTAATGTTTAATATAAATGGGTTGATTTATTTTTTATATTTTTTTTCTACTATGTTAACGGTAGTTTATACATTTCGTTTAATTTATTTTAGAATAATTAATTCATTT---------------AAATTGATGAGCTATCAT------TGTTTTAATGATCAAGATTATTTAATATTATTCAGAATAATGGTAATAGTTTTTATAGTTATTATTGGGGGATCTATAATAATATGA------------------TTAATATTTATAGAT------TTTAGAGGTATTATTTTAGATTTATTTTCAAAGTTATTAACTTTAATGGTTTGTTTAGTGGGGTTTTTAAGAATAATTATAAACATA---ATCTTTTTCATTTTATTA---AATCCTCTCTCCTTAGGATTAGTTTTAATTTTACAAACTTTAGTTCTAAGTATTACTATAGGAACAATAACA---TCTTTTTGGTTCTTATATTTGTTAATCTTAGTTTTTATTGGAGGAATATTAGTTCTATTTATTTATGTAACCTCAATCTTTCCA---AAT---GAAAAATTTTCATTTAATCAAAATATATTTATTATT---------------------TTTTTAATTTCAGCTTTATTTATAATTTTTATATTATCATTTATAAAT------------------ATAAACTTCATAATAAATCTTAATCTAAATAATTTAGAAATCATTTTAAACATAAAATCAAATATAATT------------------ATAATTAGAACTATA---AAAATTTTTAGAACCCAAGGTAATTTAATTTTAATATTCCTAGTTAATTATTTATTTTATTGTATAGTAATTGTTATCAAAATAACAAACTTCTTTAAAGGAGCGGTTATACAAAT-----CATTTTGAGTAAATTTTTATTGGTT--------------------TATATTTATTTTTATTTTTATTATAAATTTTGGAT---GTTATTTGGTGGAATTATATATTAAATCTAT----------ATTATAGACTAGGATTAGATACCCTATTATTATTAGTTGTAAATTAG----TAACCATATTATTAATAGTTAT----ATTCTTT--------AAATTAAAAGAACT-TGGCGGTAATTT---AATCTTTCCAGAGGAACCTGTCCTT-TAA-TTGATATTCCACGAAGTATATTACT--------------------------TTAATTTT----AAGCTTGTATACCTCTGT-----TGATGAATGTT-----TTGTAAGAATATTTTCT------------------------------------------------ATGTTTAATTTTATATTTAATGTTAGGTCAAGGTGCAG-CAATATTAA-AGTA--GTGATGGGTTACATT--------ATTAATATA---------TTTGGATTAATAAATTGATATTTAT----TATGAAATAGGATTTGGTAGTAA--ATTCTTTTATCTATTAGGGTTGATTTTTGCT-CTAGATTATGTACATATCGCCCGTCACTCTACGGGATAAGTCGTAACAAAGGTAGTTTTACTGGAAAGTGGTACCTTTTGTATCAGGGTTTAA---ATAAG--AAAGATTATATAAATT------TATCTCGAATG--AT-TTTGATTTAATTTTTTATAATTATTAAT-GTAGAAAAATTAATATTTAATAAATAATTAGAAA-TGAAAAGTTAGGCGTTTAATCATAA-TTCTAGTAACTCGACAAAAATAA---TTT-CGCCTGTTTATCAAAAACATGTCTTTTTGAAAT----AATTTAAAGTCCAATCTGCTCACTGA-----AGAT-TTAAAGAGCCGCAGTATAT-TGACTGTGCAA-GGTAGCATAATCAGTAGTTTTTTAATTGAGAACTTGTATGAAAGATTAGACGAGAATTATATTTTCTTTATTTTAATAAATT---TAATTTTAATTTTAAGTGAAAATGCTTAAATTTTTTTAAAAGACGAGAAGACCCTATAGATTTTTTTTAATTGGGGCGATTTTATAAAA-TAAAAAACTTAAATTTTT-ATTACCATAGATAATTGAA---TTTTTTGATCTAAAAATTTTAAAGAAA-A-GATTAAATTACCTTAGGGATAACAGCG--TAATTTTATTTAAGA-GTTCATATCTA-TAATAAAGTTTGCGACCTCGATGTTGAATTAAG-ATAATATATTGGAGTAGAAATTAAT--ATAATTA-GTCTGTTCGACTATTAAATTCTTACATGATTTGAGTTCAGACCGGCGTGAGCCAGGTCAGTTTCTATCTTTAA-TTTATTT-ATTTAAG----TAAGTACGAAAGGAC

Pediculus_capitis ATATCTTCTTTTGACCCTTCTACTTCTATTATTCTAGGA---TTAAAAGTA------AAATGATTTATTATTCTT---------------------TTTCCTTTCTTTTTTATAACTGGAAGTTATTATTTAATTCACTCAGGCTACCGTTTTTACGTGAGTTTTGTGTTAAATAATTTATTAATAAAACACTACATATCT---------------------------------ATTATTAGGTTATCTGTTTTTATAATAATTTTAACCCTTAATACAATTTCACTTATGCCATTGGTGTTGCCTTGCACTTCGCATTTAAGAGTTAATTTAGGACTTTGTTTACCTTTATGGATAAGAGGGGTTGTTTACTCT---TTAAAAAGCTCTATGCGAGGGTTTCTAGCTCATCTTCTTCCTTACGGGAGTCCAATTATACTAAGTCCGTTCTTAGTGGTAATCGAGTTGTTAAGAGTCTCGATTCGTCCTGTATCTCTAAGAGTTCGACTTCTAGCGAATATCACAGGAGGACATTTAATTATAAATCTTTTAGAAGAAGGCTTATCTTCAGCTGTACTTCTTGTACTTCCTTTCTCAATAGCAGCGTATGTTCTTCTCTTAGCTGCTGAGCTATTTGTCTCATTTATTCAGTCTTACGTTTTAAGTAAACTGGTTTCAATTTACTGAGAACTTTGTCCGTCGATGTGAACGTTGTATTATATAATTGTAATATTTGTATTATATTTTATATTAACT---ATGATTTATTTTATAAAAATTGATAATTCAAATTATTTAGAGAATTTTAAATTA------AAAAATAAAACAATCTCCAATCTTTCTATATTTTCTACAAATCATAAAGATATTGGATTTTTATACTTATGCTCTGGAGTTTGGTTTGGACTTTTAGGCTTAAGGTTAAGGTTAATAATCCGGTTAGAACTTTCTAGAACAGGCTTGCTTTTGTCTGATAGACACCTATATAACGTATTTGTTACTTCTCACGCTTTTGTAATGATTTTTTTTATAGTTATGCCTGTAATAATAGGCGGTTTTGCAAATTGATTAGTTCCTTCAATATTAGGGTCTCCAGATATAGCATTTCCTCGTATAAATAATATGAGTTATTGACTTCTCACACCCTCTGGGATTTTGCTTATTAGTAGCTCATTTGTTCAAGGTGGTGTGGGTACTGGCTGGACTGTTTATCCCCCTCTTAGGTCTCTAGAAGGCCAACCTTCTGTTTCAGTTGATTTAGCTATTTTAAGTCTTCATTTAGCAGGAGTGAGTTCGATTTTAGGATCAGTAAATTTTATTAGAACTATTTTTAACATATGGCCTCAATATTTTGGCTTAGTTCGACTGCCTTTATTTTGCTGGAGAGTGTTGGTAACAGCCTTTTTATTATTACTGTCACTTCCAGTTTTAGCTGGAGCTATTACAATGCTCTTAATAGACCGTAATTTCAATTGCTCATTTTTTGATCCTTTAGGGGGTGGTGATCCTGTTTTATACCAACATTTATTTTGATTTTTTGGACATCCTGAAGTTTATATTCTTATTCTTCCTGGATTTGGTCTTATCTCTCATATGGTGGTAGATTGTTGTGGAAAGAAAGAAGTTTTTGGGTCATTAGGAATGATTTACGCAATATCCGCTATTGGGGCTTTAGGTTTTGTAGTTTGAGCACATCACATGTTTACAGTTGGATTAGATGTGGATAGACGGGCTTATTTTACTAGCGCTACTATAACAATTGCAATTCCAACGGGAGTGAAAGTCTTTAGGTGATTAGGCACTTTGTTTGGCCCAAAATTAAAAAGGAGAATTAGCTTGTTGTGATCTTTAGGATTTATTTTCCTTTTTACAATTGGAGGTTTAACAGGCATTGTTCTTTCTAACTCATCCGTAGATGTTTCACTACATGACACTTATTATGTAGTTGCTCACTTTCATTACGTTTTATCTATGGGTGCTGTATTTGCTATTTTTGGCGCTTGAAACCACTGATTCTCACTAGGGACTGGACTTAAACTTCGTAAGTCTTTTATAAATGTTCACTTTTGGTTAAGATTTGTGGGAGTGAATTTGACTTTCTTTCCTCAGCACTTTCTCGGGTTAGCTGGGATGCCTCGACGTTACTCAGACTATCCTGACGTTTACCTGAGGTGAAACAAAATTTCTTCAATAGGAAGGCTAATTACTACTTTGGGTGTTGTAATCTTTCTTTTAGCTCTTATAGAAAGGTTTTCTAATCCTCAAAAAATTGTATTTAGAGATGCTAGATTGCAAGACCTTCCGCGTCTAATGGGTATACCAGCAAGAATGCACTCTCATTTTACTCTTACATTTACTAGAGTATGTGGATTCCAAGATAGAAATTCTCCTTTAATAGTTTTTGTGTGTGATACTTATGACCTTGTGTCTATTGTTTGTGTGGGGGTGATCTCCTTAGTAATGTACGTGGCTGTTAGTTTCTTTTTTATAAAATCTTGA---------AATTACTATTTTATAGGTCTTGAAAGATTGGAGATTGTTTGAGTTATCTTACCCTCACTCTCTTTAGCAGGGTTAATTTTACCGTCACTTCATTGTTTATACTTAATAGACGAGGTTCTTTCTCCCGCTATGAGATTAAAAGTGGTCGGACATCAGTGATTTTGGTCTTATGAGTACGGGGATTGAGAAAATATTGAA---------TTTGATTCATATATGATAAAATTAGAGGAGCTTGACTCGTCATGTCCTTTTCGACTTTTGGAGGCTGATTTAAGCGTGTTTATCCCTTATTTGACTGAAGTGCGTGCTATTGTAACATCTGCCGATGTTATTCATTCTTGAGCAATTCCTATAATAGGAGTAAAAGTAGACGCTATTCCTGGGCGTTTAAACCATGCACTTATTTACTCATTTAAAATTGGCACATCTTATGGTCAGTGTTCTGAGATTTGTGGTGCTTATCACAGGTTTATGCCGATTAAAGTCACTACTCTTCCAAAAGAAGACTTTATAAAATGAGTTAAAGATTTAAAATTTCACCCATTTCATCTTGTTGATGTAAGACCTTGACCTATTTTTTTAAGATTTTCTCTTTTATTTTCAGCGTCCATAACATTGTGTTGAATTAACGGGCTTTATTCTTTTTATATTTTAATAATTAGAATTGTTGTTTCATCTTTAATTGTTTCTTTTTGGTGACGAGACGTTACTCGAGAGGCTACTTTTCAGGGTAAACATACAATAGAAGTAATTGCTGGATTGCGTTTAGGAATGCTTATGTTTATTGCTTCAGAGGTAATGTTTTTCTTTTCGTTTTTTTATGCTCTGTTTTTTCTTTCTTTAAGGCCTGACGTGTCATTGGGACTTCTTTACCCTCCTGTGGGTGTTAGCCCTGTAGGCGTTTTAGGAGTTCCTCTTTTAAATTCTATTTTATTACTCTCTAGCGGTGTGTCTATTACTTGAGCTCATTATGAGCTTTTA------AGGAAAAATATTTCTTCTAGGCTTATCGGCTTATTAATCACTTTAATTTTAGGTCTAGTGTTTCTAATATTTCAGGCTGTTGAATATAAAACAAGCTCTTTTACTATGGCTGATAGAAGTTTTGGCTCAGTGTTTTTTCTAATAACCGGCTTCCACGGAGCACATGTTTGTGTGGGAGTTGTGTTTATTACAATTAGAACCATTCGGCTTTACTTAAATCACTATAATAATAATCATCACTTAGGGCTTGAGCTAGCTGCATGATACTGACACTTTGTCGACGTAGTGTGGTTATTTTTATACCTAACTCTTTACTGATGAATACTCTTGAAATTTTGGGTCTTATTAGGCTTGTTTCTTTCAATTCAGATTTTAAGAGGTCTTTTTCTGGCTTCTCATTATGAGGCTTCTACTAAT---TCGTTTTGAAGTGTTATTTTAATTGATTTTGATGTAAATAGAGGGTGGTTGATTCGTAGTTTTCATGCTAACGGCGCTTCTTTTTTCTTCATTCTTGTCTACGTTCATATTTGGCGTGGTTTATGATTTGGTTGTTTTACACAAAAA---TATGTTTGATTTTCAGGAATTTCTATTCTTCTTCTTATAATAGCAGCAGCTTTTATGGGGTATGTTCTTCCTTGAGGTCAAATATCTTTTTGAGGAGCGACTGTAATTACTAATCTTTTAAGTGCTATTCCTATTGTTGGAAGAGATTTGGTTATTTGAGTGTGAGGAGGGTTTTCAGTTAGACATCCTACTTTAGAGCGGCTGTTTACTCTTCACTTTCTTTTACCGTTTGTCTTATTGGGGTTTGTTATAGCTCACATTATTCTCCTCCACCAACACGGTTCTAGAAATCCTTTAGGATTGGATTTGGATAGTGATAAAGTTTATTTTTATCCTTACTTTTATCTAAAAGATATTTTAGGAGGTTTTGTGTGTTTATTTTTATTTGTTTTGATTTGCATTTATTCGCCGGACTTCTTCATAGACCCGGATAATTTTGTTGAATCAAACCCGATAATTACACCTCCACATATTCAACCAGAGTGGTACTTTCTATTTGCATATGCAATTTTACGGAGTGTACCTAACAAGTTAGGAGGAGTTGTAGCTTTGCTTCTAAGAATTTTATCTCTGTCTTTAATTAGCATA---------GGAAGCTCTGTCTCAAGTCGCTTTAGAATAAGGCGAATGATTTTAACTTATTCTTTTACGAGAGTTTTTGTTATGCTCTCATGACTTGGCTCTCTTCCTGCTGAGTATCCTTTTACTCTGCTAAGTCAAGTTGTAAGAGTAATTTATTTCATTCAAGTAATTCTATTTATGCAGTCAGTATTTATTGTTTTATCACTTTTAATTTGTGTTGCTTATTTCTCCTTGTTTGAACGTAAACTTCTTAGGTTAGAGCAAATCCGGCTTGGACCAAATAAAGTAGGACCAATTGGTATTCTTCAACCTTTAAGAGATGCTCCCAAATTATTATCCAAGACTATTTGTCCGCAG---AGAGAG------TCATGAGAGTTATTCATTATGCCGTTTATTACATTTATGTTAAGCGTTTCCTGATGGTACCCGCTCTATTTTCCAAAAACTTTATGA------GAATCAAATAACTCACTTTTAATCCTTATTTTTATCTCAAGAGTAAGAGTTTATGCATTAATTTTTACAGGGTCACTGCCAAAATCAAAATATTCGGCTCTAGGAAGACTGCGTGCAATTACTCTTTCTATTTCTTTTGAATTAGTTTTTTCCACTGCTATGCTAAGTATGGCTGTAGTTTTTAATTCTTTTAGCATTAAATTTATAGCAACCAATCAGAGC---GTGCCTAATATTATCTCAATAATT---GTAGTTGGAATTTTAGTATGAACCTCACTAATTGCTGAATGCGGACGAACTCCTTTTGATTTACCAGAAAGAGAGTCTGAATTAGTAAGAGGTTTTAACGTAGAGTACGGAGGTAGGCGTTATGTTTTACTTTATTTAAGAGAAAGATTGTTACTTACCATTTCATCTATTATTATAAGCATTTTATATACCTGC---GGGTATAATCCATTAGTGGTTTTAACATGAATTAGAATTTCT------------ATTGTTATACGAGCTAGAGCTCCTCGCATTCGGTACGATAAATGCATAATATTCGGGTGAGAATTTTCAATTCCTTTAATCTTAATATTTATGAGATTTACTATTTTGATTATTTTGGCTATTATTTTAGCT------------------------TTAGTTACTTTGGTTTTTGTATCTCAAAGTCCTAAATTAACTCTAGATAGGACGCCCTATGAGTGCGGTGTAATGCCTTTTAGAATAAGAACTCTGTCTACACACATTCATTTTTATGTTGTAAGTGTGGTATTTCTTATTTTTGATGTAGAATTAGTAGCTACTTTGCCTGTTGTTACTTCA------AGGCTATTAGAGAAAGATTGATTGTCAATT---TGACTTTTAATTCCACTTATTCTCACCTTAGGGTTGCTTTTAGAACTTCATTATGGAAGTTTAGATTGAAAAATTATATCTTCTCTTATTTCTCTTGAATTAAGTTGGTGCTGGGTTTACGTAATTATACACATTGCACTTTCTGAA---AGGTTAGACACTCTTCTAAGATCAGAGGTTTTAAGTGTTATTGTTTGCGAGAGAGTTGTAGGACTTTCTTTACTTATTAGGCTAACTTATGGGTGAGGAAGGACTGGTTTCAGGGGGGTAGAAATTATTATTGTACTGGATAATTTAAGCCTGACTTTTTTGCTTATAGTATTAACAATTAGGTCGCTTGTTATAGCTTATAGAAACTATTATATAGCAGGTCATAATCTTGGCGGTGATTTTTACGTTTCCATGGTCTTATTTATTGTAAGAATATTACTTCTATCTCTTAGAGGCTCAATGTTCTGATCTTTTATTGGGTGGGACGGTTTAGGAATAATAAGTTTAGTTTTAATTTTATTTAATAAAAGATGGAGTTCTCAAAAATCGGGAGTGATTACTTTCCTAATGAACCGATTAGGTGATTCTTTTATAATTATTTGCTCGTCATACTTAAGAGTGTGAGGGATA---TGTGAA---------------------------------GTTTATTGGTTTTGAATTTTAACTTCTTTGTATTTAATTGGAGGGGCTTCAAAAAGAGCTCAATTTCCATTTTCTAGCTGATTGCCGGAAGCTATGGCAGCCCCTACTCCCGTGAGTAGGTTAGTTCATTCTTCAACATTAGTAACTGCTGGTATTTATGTTTTAGCTCGTTATGGAAGA---ATAATCGATAGTTTTTACATT------------CTTACCTACTTATCTTCTATTTCTATTATTATTTCGGGTGTTTCAGCTCTCTGAAGGAGAGATTTAAAGAAAGTTGTAGCTTATTCAACTCTTTCTCACATTAGCTTAATGCTTTTTTACTTATCTGAAGGAAGGGTGGAAGGAGCTTTAATCCACATGTTGACGCACTCTGTGTTTAAAAGGCTGTTATTTTCTTGCCTTTCAATGGCCGGACTGCCGTTTCTTTCGGGAGGTTATTCAAAAGAGGTG---TTA---------CTTATTTTAAGTTTAAATAGATCAATTATGAAGCTTATTATATTTCTTACAGCTGTAATTTTCACTAGAGGTTATTCGTTTCGCATTATTTACTTACTTTCTAGAAAT---------------------ATTAATATAACACAAAAT------------ATTGCAGTAAGGAGGTTGTTTAGCAGCCCCCTTAAACTGAGGCAAATGCTTAACGTTCTA---------ATTTCAGCTTGAATTTCCTCAAGTCCTGGCTACTTATCTAAACTC------AGAAGGCAG---AGAGTTAATTTAGAGGGTAAAATTATAATACTCTTTATAATTCTATTTGGTATGGTTGCTATGTTTTTTACGTTT---CTTATTTTAGTAGGCTCT---GATCTTTTGCTGAAGCTTTTTTCTCTAGCTATAACTGTTTTTACAGTCGGACTGCTTATTCTTCTAAACTCTTACTCTTCATGGTCTTGGTTGCTTTTATGGTTAGGGATTTTGGGTGGTTTAATTGTCTCACTTTCTATGGCTTTTATTGTAACGCCA---AAA---ATTAATTCCTCAAAGGACTGGTCTAGAAGATTAAGA---------------------TTTATAAGAGCGTTTTGAATTTTAACTTCTGTTATTCTATCTGCTATA------------------------ATATGAAAAGTAGAATTTAAAGAATGATCTGACTTGTACTCTACAGAG---AGTGTAAGAGAA------------------ACTTATAATTCACTA---CTTATAGATCTTAAGGTTTATACAGCTGTGGTTATTCTTTTAATT------TACATTTTAATGCTTCCTGTTATAGAAGTTTTAACGTCACCTTATAGACGTGCGGTCATACATTA-----AAGTCAAA-TTAATTGGTTAAAGCTAATAAATAATTACTATTAAGTACAACAACTCTACTTATTATTGTAAGAGTAAAAT---CTTTATAAATAAGATCTTACGAGGCGCTAGC-----------ATAAAGACTAGGATTAGATACCCTATTATGGGCGTGAGTATGAAAGTTTATTAAGTTAATAGCCTTAATTAAAGCA---------------AAACCTATTTACTA-TGGCGGCTGTT----AGTCTCACCAGAGTCGTATGTCCT--TAA-ACGAAACTGCGCGAT-TATCTTACCTTTTAACTTTTGCTTATATAAAATAATTACATTATAGCAATCTGGCACGTCGCTGTACTAAAAGTGAAGATT-----TTAAATAACTCATCTTC------------------------TAATTACTTATTAGCA-------------TTTATTAATAATGTA---CAAGTCAAGACGCCGTTT----TAAGAGGCTTAAGATG---TACGATTTA-----ATTGATTTG---------ATAGAATTTATAAGAATGAAAACTTTCTTTATTAAAAT-AATTTGGAAGTAAA-ATAGGGTTATTATTCTTATTTGAAATGAGCTACTAATAAGTGTACATATCGCCCGTCATTCCAATGGACAAGTCGTAACAAA-GTTGATTTACTGGAAAGTGGTACCTTTTGCATCAGGGGTTAAGAAATAA--GATTATTAAATTAATAA------CCTCCCGAAAG-AAGC-AGGATCTTAAACTTTATTGTTGGTTGTTGTTTCATTAACATTAA-TAATAATATTTAAGAAGCTAAAAGTTATAACGACTCTTCTGATCACCTGGTAACTAGGCAAAC-ATTT--AGCCGGACTGTTTAATAAAAACATTTCCTCTTGCATAAA---AGGG-AGGTAAAGTCTGCTCGGTG-----TAATTGATTAACAGCTGCAGTAACT-TGACTGTACAAAGGTAGCGTAATCACTTGTCTTTTAATTGAAGACTAGAATGAACGGCTTAACCAAGCTAATTCTGTCTCTTTTTAACCTTAAG---AAATTTAAGATTTAAGTTAAAACGCTTAAATTATTTAGAGGGACGAGAAGACCCTGAAGATCTTTTTTATTTGGGATGAATGT--------AATAAACATTATG----GTTTACTAAGACTTA---AACGTCTTTATTCTGACCCGTTTAAACGAGAATCTGTTTAAGTTACCTCAGGGATAACAGCG--CAATGTCTTTTATTA-GACCTTATAAAA-ATAGAAGTTTGCGACCTCGATGTTGAATTGAGTTAAACTTTAAGTAGAAGAGTACTTA--AGAGTAG-GACTGTTCGTCTTTTAATAGCTCACATGATTTGAGTTTAGACCGACGTGAGTCAGGTCAGATTCTATCTTCTAATATTAAGCA---ATCTT-TTTTGTACGAAAGGAC

Heterodoxus_macropus CTATCAATTTTTGATCCTTGTTCAAGAATTTTAAATATT---TTTAATTTT------AACTGAATAGTTATAAGA---------------------TTATGTTTATTAATTCCTTTAAATTCATTTTGAAAAGTTCCAAGAATTTTATTAACATTTTTTGATTATATAAAAAATCTAATGAAATCTATATTTACTAAT---------------------------AAAGTAAATTTTCAAATTTCAAGGATTTTTTTTTTAATTTTAGTGTTTAATATTTTAGGAATTTTTTGTTTTACATTTTCTGTAACTAGACATTTAGTTATTAATTTATCTTTAGGATTTTCTATTTGAGTAGGAACTTTATTATATAGTTCAATTTATAAA---TTAAGTGATTTTTTAGCCCATTTGACACCAATAGGGTGTCCTATGGTTTTAGTTCCTTTTATAGTAGTAATTGAATTTATTAGAATAATAATTCGACCTATTACTTTATCTTTACGACTAATAGCTAATATATTAGCTGGACATATAATTTTATCATTAATTAGAACAGGAGTAAGGTTAATACTTTCTTTATTTATTCCTTCAGGAATATTGTTGTTACTAGGATTTTATTTATTTGAAATTGGAGTAGCAATTATTCAAGCTTACGTTTTTTCAATTCTTCTTTCTTTATATTGAGAAATATTTCCATATCTTTGAATATGAATTTTCATATTACTTTTATCTGTTTTTATTTTGTTTTTTATG---AAATTATTTTTTTCAAATTTAATTTCTAAACGAAATAATAGATATTGTAATAAA------CATTATAAATTTGATAAAAAATTAGAATTTTATTCTTCTAATCACAAAAATATTGGTATTTTATATATAATTCTAGGAAGATGATCTGGACTATTAGGATTTAGATTGAGAATAATAATTCGATTAGAATTATCTGACTCAGAAATATATTTATTTAACCCACATATTTACAATGTTGTTGTTACATCACATGCATTTTTAATAATTTTTTTCTTTATTATACCATTTATAATTGGAGGTTTTGCTAATTGATTAGTACCTATTATAAATGGTAGGCCTGATATATCGTTCCCTCGAATAAACAATATAAGGTTTTGATTATTACCTCCTTCTTTAATCTTTATATTATGTAGAATAATACTAGATGGAGGTTCTGGGACAGGTTGAACTGTTTACCCTCCATTGTCTTCTTTAACTGGACATGCAGGTATGTCTGTAGATATATTAATTTTTTCTCTTCATCTTGCTGGGATTAGATCAATTATAGGGGCTATCAATTTTATTACTACTATTTTTAACATA---GTTTTCTTTAAAAATTTATCAATAATAAGATTATTTAATTGATCAGTATTAATTACTGCTTTTTTATTACTTTTATCTTTACCAGTATTAGCAGGTGCTATTACAATATTATTATTTGATCGAAATTTTAATTCAAGATTTTTTGATCCTATTGGAGGAGGAGACCCTATTTTATATCAACACTTATTTTGATTTTTTGGACACCCTGAAGTTTATATTTTAATTTTACCAGGATTCGGTTTAATTTCTCATATTATTGTTCAAGAAAGAGGAAAATGTGAAACTTTCGGAGTTTTAGGAATAATTTATGCTATATTATCTATTGGAATTCTTGGATTTATTGTATGAGCTCATCATATATTTACTATTGGTATGGATGTTGATACTCGGGCATATTTTACTTCAGCGACTATAATTATTGCAATTCCTACTGGAATTAAAATTTTCAGATGGTTATCTACTTTTTTTGGTAGAAAAATAAAATTTAATTCTTCAGAATTATGAAGAATGGGTTTCGTTTTCTTATTTACTGTAGGTGGTTTAACAGGGGTAGTTTTAGCAAACTCTTCTATTGACATTGTTCTTCATGACACCTATTATGTTGTTGCCCATTTTCACTATGTGTTATCAATAGGAGCAGTATTTGCGGTATTTTCAGCTTTTACACATTGATTTCCATTATTTTTTGGTGTAAAAATAAGAAATGCTTTAATAATTCTTCATTTTTGAATTACTTTTTTAGGAGTTAATTTAACTTTTTTCCCTCAACATTTTTTAGGACTTAGAGGTATGCCTCGACGTTATATTTGTTATCCCGATTTTTATTATTCTTGAAATTTTTATTCAAGAATTGGATCAATAATTACTTCGGTAAGATTATTAATATTTGTTTTCATAATTTTTTATAGATTTTTTGAAAATAAAAAATTATTATTCTACTCTTTTAGAATAAATTCTATTGAGTGAATGCTAGGAACTCCACCTTCTGCTCATTCTTTAAATGAAAGACCTGTCTTAATTGAATTTAATCTTTCTGATGGATGTTCATTAATTATAGAAAATATAGTTGCATTTCATGATTTCACTTTAATAATTCTTTTATTTATTACAACAGTAGTACTAATAATATTAATTTCTATTATAATTACTAATTTAGTA---------AATCGATTTTTAATTTATAATGAAGTATTAGAATTTATTTGAACAGTGATTCCTAGGTTTATTTTATTGATTATTGCATTACCTTCTTTAAAAATTCTTTATTTAGTAGATGAATTACTTAATCCTGAAGTTACAGTCAAAGTCATTGGAAATCAATGATATTGATCATATCAATATTCAGATTTATTTAATATTGAA---------TTTGATTCTTACATAAAGAAATGAGAAGGGCTA------TCTGAT---TTTAAATATTTGGATGTAGATAATCGAACTGTACTTCCTGTAGACACTAATATTCGAATAATTATTACATCTTCTGATGTAATTCACTCATGAACAATCCCTAGATTAGGGGTTAAATTAGATGCCAATCCTGGACGATTAAATCAATTAAATATTTTAGGTAATCGATTAGGATTATTTTTTGGTCAATGTTCAGAAATTTGTGGAATTTTACATTCATTTATACCAATTTGTGTAGAAATAGTAAAACCAGAGTGATTCTTAAAATGATTATATAAAAATGGTTTTTTTTTATTTCATATTGTAGATGAGAGGCCTTGACCTTTATTTCTTTCATTTAGAGTTTTTTTAAATATATTAAGAGCTTTAGTTTATTTAAAG---TTTCATATTTTAATTTATATGTTACTAAGTAATATATTAAGAATTTTAATTTTTTACATGTGAATACGAGATATAATTTCTGAAAGAACTATGCAAGGAATACATACTTTAAAAGTTCAAAATGGAATTAAAATAGGAATAGTATTATTTATTACATCTGAGGTTATATTTTTTTTTTCTTTCTTCTGAAGATTGGGATATTATATAGTAAGTCATGAATATATTCTT---AGAAATTGACCTCTTTTAGGTATTATAAGATTAAATCCATCTACAGTGCCATTATTGGGTACAATAATTTTATTAAGATCTGGAGTATCTGTAACATGATGCCATAATGAGCTTATATTAAGAGAAGGAAATCTCAGAAGAATAAAAAATTCATTATTAATTACAGTAATTCTTGGAATAGTATTTGCAGCTCTTCAAATATGAGAATATTTTATATCTACTTTTACTATAAGAGATGGTGTATATGGGTCGTTGTTCTATATAATAACTGGATTTCATGGATTTCATGTTATTGTTGGAACAATTTTTTTATTTATTATTTTTTTACGATTAAAAAATTATCATTTTTCTAGACACCATCATTTAGGATTTCAAGCAGCAGCTTGATATTGACATTTTGTTGATGTTGTTTGAATTTTTTTATATATTATGTTATATTGAGGTTATATATGAAATTTTGGAAGTTTATTAGGGCTATGTCTCTTTATTCAAATTGGATCAGGTTTATTTTTATCACTTCATTATAATTCAAATGTTGAGTTAGCTTTTAGAAGTGTTATTTATATAATAAATGATGTTAATCATGGATGAATTTTACGTGTAATTCATGCAAATGGGGTAACAATAATATTTATTTTTATGTATATTCATATTGCTCGTGGACTTTACTATAAATCTTATAAACTAACT---TTAGTTTGATTAGTTGGGATCTTAATCCTACTATTAACAATAGGAACTGCATTTTTAGGATATGTTCTTCCTTGGGGGCAAATATCATTTTGAGGTGCTATAGTAATTACTAATTTAATTAGAACTATTCCTTATTTAGGAGTAACATTAGTTGAATGGGTGTGGGGAGGATTCTCTGTTAGAGAGCCAACTTTAACTCGATTTTTTTCATTTCATTTTATTTTACCTTTTGTAATTCTAGGGGCATCTGCTTTACATATTATTTTTTTACATAAGTATTTAAGATCGAACCCCCTTGGTTTA---CCTAAGACTGATATAATTTCATTTCACCCATTTTTTACTGTTAAAGATATCTTGGGTGTAGTATTATTTTTATTTAGTTTATTATTTTTATCTTTAACAGAGCCTTATAAGTTTATAGACCCAGATAATTTCATTTTAGCAAACTCTATAGTTACTCCAGTTCACATTCAACCAGAATGATACTTTTTATTTGCTTATTCTATTTTACGGGCTGTTCCTAATAAATTAGGAGGGGTTATTGGTTTATTAATGTCTATTCTAGTATTAGCATTATTTTTATTTTCTAATAAAAGAAAATCTCAAGAAAGGGTATATTAC---------AAATCATTCTGTTGAGTTCAATTCACTATTTTTATATTATTAACATGAACTGGAAGATTGCCAGTAGAATCTCCTTTTTTAGAAATTGGACAATGTTTATCAGTAATATATTTTTTAAATATATTTTTATTATTACAACATTTTATCCTTGTTATTATAATACTTCTTACTGTTGCATTTTTTACTTTATTAGAACGAAAAATTTTAGGCTATATTCATTTCCGAAAAGGTCCAAATAAAGTTTTATTAAAAGGAGTTTTGCAACCTATTGTTGATGCTATAAAATTAATTACAAAGGATGATTCTCCAATC---ATTTATAGAAATATTTTTTTGTATTATATTTCTCCTATATTTAGATTTATTATAAGAATAATTATTTGAATAATTCTTCCTTTACAATTTATTATTTTTAAT------TGAGTTAATAGATTTTTAATTTTATTTATATTACTAGGAATAGGAGTATATAGAATATTTTTGTCAGGTTGATCTTCAAATTCTAAGTATGCTTATTTAGGAAGACTTCGAGCTGTAAGGCAATCAATTTCTTATGAAATTTTAATAAGGATATTATTTATTGCTTTAATAATAACAACAAAAGGTATAAGAATTTATTATATTTTAAAATTTGACCCT---------TTGGTATTTTTTTTATTT---CCTTTTTTTATTGCATATTTATTTATTGGCTTAGCAGAATTAAATCGATCACCTTTTGACTTATCTGAAGGAGAGAGAGAATTAGTAGCAGGCTATACAGTAGAATATGGAGGAATTATATACACAATAATTTTTTTAAGAGAAAATATTATAATTATATTTTTTTGTTACATAGGTTCATTATTTTTTTTT------TATATTAATAGGACTATGAGAATTATTTTTTCAATAATAATAATTTATTTAGTTTGTTTAATTCGAGGAATTCTTCCACGAATTCGATATGACCATTTAATAATATTTTGTTGAAAAATTATGTTACCTTTAATAGTAATTTTTGTCAATCTTTTACTTAGAAGACTAATTATTTTAGTTTTATAC------------------------TATCTTAGAGTATTATTTATAGATAATAAGAATATTGTTGAAGATGGAAAAAAAGAGTTTGAATGTGGTTTTCGAGCAGAAAATTTATCTCGGTTACCTTTTTCAATGCAATTTTTTAGAATCGCTTTAGTTTTTCTTATTTTTGATGTTGAATTAATTATTATTTTACCTTATATTTTTAATTTTAATCACATATATATA---------------------TTTAGAATTATAATAATTTTGTTATATTTAGGAACCCTTTTAGAATGAATAGAAGGAAGATTAGATTGATATATAATAATTACTTTATTAAGATTTGAATTAATTGGTTTGGTAAATTTTATGATGATTAATTTTTTATTTCCC------------AATATAAAATTGATTTTAATTATAGTAACATTTTTAATGTTAGAAAGAGTATTAATATTAATTCTCTATACATGTTTGATTCGTGAATTTGGAATAGAAAGATTTATTGAATGTAACTTTAATTTTATTTTTGATAAATTTTCTATAATCTTTTTATTTATAGTTTTAATTATTTCAAAAAATGTGTTAAAATACTCTTATTTTTATTTTGTTGGGACTGTATGAACTTTACGATTTATTGGAATTTTAATTTTTTTTATTGTATCAATATTATGATTAATTATATCTTATGATATATTTACTTTTATTGTAGGATGAGATATGTTAGGTGTATCTTCTTTTTTATTAATTTTATACTATAATTCTTATAAATCGAAAAAAAGAAGATTAATTACTTATATTAGAAATCGGTTTGGTGATGGATTTTTTATATTAGCTATAGTATTAGCTAGACCTTTATTTAGC---GAATTTTTTTTATTTAAAGCTCAT---------------------------TATTTTCTTCCAATTTTAGTATTTTGTACTAGAATTACAAAAAGAGCTCAATTTCCATTTTCTAGATGACTACCTGAAGCTATAGCAGCCCCAACTCCTGTATCAACTTTAGTACATTCATCTACTCTAGTTACAGCTGGATTTTATTTTTTATTTCGATTTCAAGAACTTTGAATTAATAATATTTATGCTTTAAATTTA---TTGTTATTTATTTCATTGTTTACGATAACTTTAGCCAGAAGAGCTGCTTTAATAGAATATGATTTGAAAAAAGTTATTGCTCTTTCAACTTTAAGACAGATTAGTTTTATATTTTTTAGTTTAAGTTTGAAGTTAACAACTTTAGCTTTTTTTCATATAGTTATACATGCATTTTTTAAAGCAGCAACTTTTTGTCAAATGTCATTGTCAGGATTTTTATTTCTTTCAGGGTTTTATTCAAAAGATTTA---ATTTATAAATCTTTTTTAGCCTATAATAATTTAAATTATTATTTTATGATTGTTTTTCTTATTTCAATTGTTTTAACTATATTATATTGTTTACGAATATGTTTAATAATAATAAATTTT---------------------TTAAAAATAACTTTTTAT---------TTAAATTTTAAAGATATGAATATTTTATTACCAGTATTAGAATTAATATTACTTTCAATTGTTTCAGGTTCTGTTTTAATATGA---------------------------TATTTA------TGTGAATCAGTAATAATTCCATCAAAATTAGAATTTATGAATTATTTATTATTTATAATTATATTAATTTCTATTTTATCTAGAATTTCATTTTTCTATCTGAGAAATCTTAGAAAGATTATAAGATTAATTTTAGTTCTAATTAATATTATTTCAGTCTCAGGATTAATTATGATTTATTCATTATCTTCTTTTGAAGGAATAATTTTGGTAATTGTTTTTTTAACAGGACTATTTATTTTAATATCTTATTTAGTAAGTGTAACTCCT---GAA---AATCCAAAATTTGTAAATTTCTTTTATAAAGAAAAAGCTCAATCCAAAAATTTTTATTTCATGAAATGAATTCTTTTATTATCAATTTTGTTATTTATTTTATTA------------------GCCTTATATTGAGAGTTTAATAGACAATCTTTATGATTTGAAGTTAAATTTAGA---AAAGAAAATTTT------------------AATTTTTCTTTTTTA---TCTCCATTTAAACACATTTCTATTTATATAGTTAATATCTTAATTTTTTTACTTTTATTTATTGTATGAATATATATACGATTTACTTATACAAAAAAGGGAGCGGTTAAACATTTTCTGTGAGTTAATTAAAAT-------------------------------------------AATGAAGATTACAACAAATAATTTAAAATTATTGGTAAAATGATTTAAGAAAACTAT----------AATCTAAACTAGGATTAGATACCCTATTATT----------------------------GTAGAATTAATCA-----AATCTTCAAC-----AAAATTAAATAACA-TGACAGTATAAATATTTACAGATTAGAGAAATATGTGATA-TAA-ATGATAATCCCCAAC-AATCTTAC---------------------------TTAGTTTAT---AAATTGGTATACCGCCGTCTTAATATTAAATTTAAATATTTAAAGAATTAAGGGAT------------------------TTAGAATTA--------------------TAATTTTTTTTTATAAGTCAGGTTA-GGTACTGTTGACAACTAAGGAATTCATGTAT-------TGCA-----ATTATTATAAAAAATTATTTTGGTATAATTTTTTTTAAAATAA-----ATGAAATTGAATTTAATAGTAAA-TTTTATTTAATATATAAAATTGAATATAGTA-ATATTTTATGTACAAATCGCCCGTCAATCTTTTAGATAAGTCGAAACACA-GTAAATTTACTGGAAAGTGGTACTTTTTGCATCACGGTTTAT--AGAATAG-TAATATAAATTATATT------ACTCCCGAATT--AATAATGATCTATTTTTTTAAAAATCTAAAT-GTTTCAAAA-TTTTTTAAAATAAAAAAATTGAAG-CTATAAGTAATACGAATATTAAGAT-ATCTGGTAACTAGGCAAATATAA---AATCTGAATGTTTATCAAAAACATTTTCTTTTTATTA----TATAAAAGATAAGTTCTGTTCACTGAC---TATATGTTAAAGAACCGCAGGA-----AACTGTGCTAAGGTAGCAAAATAAATTGTATATTAATTGTATTCCAGAATGAATGAATTAACAAGATTTTTACTTTCTTAAAAATTACTATTT----AATTTTGATTTTAAGTTAAAATTCTTAAATTAAGATGTAAGACGAGAAGACCCTGTAGATCTTTTTTGGTTGGGGAAACAATTAAAGC-GAAAAATCTTTGATTTTCTAA-ATCTTAAATTTAAGGA----ATTATAATCTTTACTTATTAAGATTA-A-TATAAAGTTACCTCAGGGATAACAGCG--TTATTAAATTTTTGA-GATCTTATTAG-AAATTTAGATTGCGACCTCGATGTTGAATTAAG-ATTACATTTATATGCAGAAAGATAA--ATAGTTA-GTCTGTTCGACTATTTAAATCTTACATGATTTGAGTTAAGACCGACGTAAGTCAGGTCAGTTTCTATCTACAT-CATTTTTTTCTTTTT-----TAGTACGAAAGGAC

Ibidoecus_bisignatus TTTTCCATTTTTGATCCGTGTGTAAGATATACAAGC------TTTCAATTT------AAATGATTGATTTCATTA---------------------AGTTGAATAATAGTTATAACAGTTAAATTTATGAATTTAGATTAT---GTAAAATTATATTATTTGGTGGTTTCTAATTTTTTTATTTCATCATCAAAAACTTTATTAAAAAGAAGATAT------AAAGTTGTATCTGTTAAGATCGTCTCCGTTTTTACAATAATTTTAATATGTAATCAGTTAAGTATAGTCCCATTTGTTTTTGGTCCTACTAGACATTTATCATTTAATTCGGCTGTTGCTTTATCAAGATGGTTAGCAGGGATTATTACGATA---CTTTTAATTTCATTTAAAGATTCTGTTTCACATTTTGTTCCTTTAGGAAGACCAATATTTTTAACTCCTTTTTTATTTATTGTAGAGGTGATCAGTTGTTTAATTCGACCTGTAGCGTTAAGAGTTCGGTTAATATCAAATATAATGGCTGGACATATTATTATTGTATTATTAAGAAACCTTATTTGTAGGTTAAACTCTTAT---------TATTTGATCCCAATTGAATCATTCATTTTTTTATTTGAGTTATGTATTTCTATTGTTCAAGCTTATGTTTTTTCAAGTCTCTTAGCTTTATACTATAAATTATTTCCATGTACGTGATTATTAATTTTTTTTTTAGCTCTATGTGTAATTGTTTTTTGTCTTTCTTTGATAAATTTCTTTGTTCTAATTGATGAAAGTACTTTTAAAAAAACTCCAAAGAAA------GATAAGCAAAAACGAGAGGCATTAACCTTTTATTCGACTAATCATAAAGACATTGGGATACTTTATTTGATTTTTGGAATCTGATCAGGATTATTAGGTTATAGTATAAGACTGATTATTCGAATAGAACTAAGTCAGATAACTAATTATATTAATGATGGTCACATCTACAACGTAATCGTTACATCTCATGCTTTTTTAATAATTTTTTTTATAATTATACCAATTATAATTGGAGGTTTTGCTAATTGGTTAGTTCCTTTAATAATTGGCTCTCCAGATATAGCATTTCCTCGAATAAATAATATTAGATTTTGATTACTTATTCCTTCTTTATTATTTTTATTAATAAGAATTTTTATAGGAGAAGGAACTGGAACAGGATGAACCGTATACCCCCCCTTATCAAGT---------CAAGCATCAATTTCTGTAGACATTTCAATTTTTTCTTTACACTTAGCAGGTTTAAGTTCAATTTTGGGAGCTATTAATTTTATTTGTACTATTATAAATATATGATTATCA------TCTATATTTTTACTACCCTTATTTTGTTGATCAATTTTAATTACTGCTTTTTTATTATTACTTTCCTTACCGGTTCTTGCAGGAGCAATTACTATGTTACTTCTAGATCGGAATATTAATTGTTCTTTCTTTGATCCTATGGGAGGAGGAGATCCAATTTTATACCAACATTTATTTTGATTTTTTGGACATCCTGAAGTATATATTTTAATTCTTCCTGGTTTCGGTCTAATTTCTCATATTATTTGCGAAGAAAGAGGAAAAAAAGAGGTGTTTGGTTCTTTAGGAATAATTTATGCCATGTTATCTATTGGTATTTTAGGATTTGTTGTATGGGCACATCATATGTTTACTGTAGGTATAGATGTGGACAGACGAGCTTACTTCACCGGAGCAACTATAATTATTGCAGTTCCTACTGGAATTAAAGTATTTAGATGAATATCCACCTTATTTGCAAGGAATATTAATTGGTCAGTATCTTCATTATGAAGGTTAGGATTTGTCTTTCTATTTACAATCGGAGGTCTTACGGGGGTAATACTAGCTAACTCATCAATTGATATTGCTCTTCACGATACTTACTATGTTGTAGCTCATTTTCATTATGTTTTATCCATGGGTGCTATAGTAGCATTTATGGCTAGGTTATTCCATTGGTTCCCATTAATTTTTGGAGTTTATCTTAATTCAAAATTTTTAAAAATTCATTTTTTTGTAACTTTTATTAGGGTAAACATAATTTTTTTTCCTCAACATTTCTTAGGATTAGCAGGAATACCTCGTCGGTATATAGATTACCCAGATATATTTAGTTCATGAAATGTAATTTCTTCTTTAGGTTCTACTTTATCTATTATCAGTTTATTTATAATGATGTTCTTAATTTTTGAAAGGCTAATTTCAAAACGACTGGTTGTGTTTAGATGTAAAATTCCTATTTCTATTGAATGAGTAAATGGATTTCCTCCTAGAAATCATTGTAACGAAATAGTACCACAATTAACTACTCTTTGTTTTCAAGATAGAAATTCTCCTTTAATAATACATATTAACCATCTTCATGACCATATTATGGTTGTTATTATTATGATTATTTCTATTGTTATATATGTATTATTGACAATTGTTATAAACCCTTGCTCA---------AATCGATTCTTTTTCGGTAGAGAAGTGTTGGAATTAATTTGAACTTTGGCTCCTAGAATTGTATTAGCAATTTTAGCTATTCCATCACTTCATATTTTATACTTAATAGATGAATTAAAA---CCTATGATTAGTATTAAGTCTATTGGTCACCAGTGGTATTGGTCATATGAGTATGGAGATTTATGTAGAATTGAG---------TTTGACTCATATATGATTATAGAACAAGATTTAGAATTAGGTATA---ATGCGATTGTTAGAAGTAGATAATCGGACAGTAATTCCTGTAGGAATAGAAATTCGGATACTAATTACATCTACTGATGTAATTCACTCATGAACTATTCCTACACTGGGGGTAAAAATAGATGGAGTTCCTGGTCGTTTGAACCAAATTTATTTATCAAGTAATATTTGTGGTTTAATGTATGGTCAGTGTTCAGAAATTTGTGGAAGTTTTCATTCATTTATACCTATTTGTTTAGAAGTTTTATCTGAGTCTCGATTTATATCTTGGTTGAATAATTATGGGTTTCATCCTTTTCATATTGTTTCTATTAGTCCATGACCTATTTTATGTTCTTTTTCTATTATATCTTTTGTGATTAATTCTTTGTATTATATAAATAAATTTTTGACTTTAGATTTACTTTTAGAGTCATTATTGTCTTTAATTTTAGTTATTTTTTGTTGATGGCGTGATGTCATTCGAGAAAGAACGTTTCAGGGTTTTCACATGAAAAAAGTGTGTTTTGGTTTATATATAGGAGTTTCAATATTTATTATTTCAGAAGTAATATTTTTCTTTTCTTTTTTTTTTGGTTATTTTTTTTCTAGTTTAGTCCCAGACGTAGAAATTGGATGTTCATGACCACCAGTGGGAGTTCAATCTTTAAGGTTCATAGATGTTCCATTATTAAATACAATAATTCTTCTATCTAGAGGAATTTCTATTACTTGGTCCCACCATTCTTTATTG------GAAAATAATTTTACTAACTGCTTATTAGGGATAATTTTCACTGTTATCTTAGGTTTGATTTTTACTTTTTTTCAATTTATAGAGTATTTAGAGTGTTCTTTTTCTATAGCTGATAGGGTTTATGGGTCACTTTTTTATATTTCTACAGGCTTTCATGGAATTCATGTAATTGTAGGCACATTATTTATTATTGTTTCTTTTATTCGAATAATGAAATATCATTTTTCCATTCACCATCATTTGGGGTTTGAATTTTCTATTTGATATTGACATTTTGTAGACGTAGTGTGATTGTTCTTATTTTTAAGAGTATATTACTTATTTATGTGAAATTTTGGTTCATTATTGGGGATATGTTTGATAGTTCAAATTTTTTCTGGTTTGTTTCTTTCTATACACTATAATACTTCTATTGATGATGCTTTTAATAGAGTTTTATCTACATGTAATGATGTTAACTTAGGGTGATTGATTCGTTATATTCATGCTAATGGAGCCTCAATATTTTTTATACTTGTATACTGTCATATTGGTCGAGGTTTATATTTTGGGAGTTTCAATATAACC---TTGACTTGATTTTCAGGAGTGATTATTCTTTTATTATTAATGGGTACCTCATTTTTAGGTTACGTTTTACCTTGGGGACAGATATCTTTTTGAGGAGCAACTGTTATTACTAACTTAGTGAGAACTATTCCTTATGTAGGAGATCAGTTAGTTTATTGGTTATGAGGAGGGTTTTCTGTTAGTGAACCTACACTAAATCGATTTTTTTCTATTCATTTTATTTTGCCGTTTGTTTTAATGATAGTGGTCTTAGTTCATATTTTCTCCCTTCATAAAAGAGGAAGAAGGAATCCTTTGGGAATTTCTCCTAATTGTTTAAAAATTTCTTTTCATCCTTATTTTTGAAATAAAGACGTTTTGGGATTTGTTGTTGTATTAATTATTTTTACCGTTACATTAATTTTTCTCCCTGATGTATTCATAGATCCTGATAATTTTTCTGTAGCAAATCCTATATCAACTCCTGCCCATATTCAACCGGAGTGATATTTTTTATTTGCTTACGCAATTCTTCGGTCTATTCCTACTAAATTAGGAGGGGTAGTAGCATTAGTATTTTCTATTGTAATTTTATTTATTATTCCCTTTATTAGAAGAGGAAAAAATAAAAGACTTAATTTTTAT------CATAAAATAATTGTTTTAATACAAGTTTCTAATTTTCTTTTATTAACGTGATTAGGAGCCATACCAGTAGAATTTCCATTTTTAATAATAAGAAAAATTTTTTCTTCCATGTATTTTATTTTTATAATTTTATTACTACAATTCTTGTTAATTATTGTTGGATTATTGTTATCTGTTGCTTTTTTTTCTTTGTTTGAACGAAAAATATTAAGAGTAATTCAATTTCGAAAAGGTCCAAATAAAGTAGGGTTAATAGGTTTTTTTCAACCTTTTTCTGATGCAATTAAATTATTATTTAAAAGTAATGAAATACCA------AATTTAAGGAATATATTTTACTATTTTGCACCAATAGTATTTTTTATTTTGTCAATTTTAATTTGAATTAGTATTCCCAGAAAGTGGAATTTATTCAAT------TTCTCTTCTAGTTTCATTTTCGTTATATTTCTCTACGGAATTCCCATTTATAGAATAATTTTTATAAGATGAATTTCTAATTCTAAATATTCCAAAATTGGCTCAATTCGTTCTGTAGCACAATCTATTTCTTATGAGATTATTTTGTCTTCATGTTTATTATTCTTGATAATAATAGTATATTCTTCTTCTATAAATTTATTATATTTTTATCAATCATACGTGTGACTTTTGTATCCTTGTTTT---CCTATTTTTTTTATTATATTTATTTCTATTTTAGCTGAAAGTAATCGTTCTCCATTTGATCTAACAGAAGGAGAAAGAGAGTTAGTATCGGGGATTTTTGTTGAATTGGGAGGAGTTTGATATATTTTAATTTTTTTAGGAGAAAACTTGTATTTGTTATTTTCTTCATTTTTAATATCTTTTAGAATATTAGGAAATTCATTAATTGTTTTAAAGTTTGTAATTATTACTATATTAATA------------GTTTGAATCCGAGGAACTGTTCCTCGGATTCGCTATGATAAAATAATAGATTTGTGTTGAATTAGAATAATACCAATCTGTATATCTTTTATTTCTATTGTAATCACAGTATTATTAAATATTGTTCTATGT------------------------TTTATTTCTATTATATTTTTAAAAAATGACATAAAA---CAAGACAGAAATGAGAGTTTTGAATGTGGAATGGAAACATTTTTTAATTTTAATTCATTTTATTGCTTGCATTTTTTTTTAATTGGAGTTTTATTTTTAGTTTTTGATATAGAAATTATTATTTGTATTCCAATAATTTTTTTA------AATTTGGAAATGATTAAAATATTATTATAT---TGGAGATTAATAATAATTGTATTAATTGTTGGTTATTATTTAGAGTTAGCTATTGGAACTTTGAACTGGAAAATAATCACAATTTTGATTTCATTAGAAATATTA---GTTTGAACGATTTTTTTGTTTATGATTTATATAGAAAAATCTATTATTTCTATTTCTTTGTTTCTATTGTTTTCATGTATGTTGATTTGTGAAGGAGTAGTTGGTTTAACTATTTTATCAAAGTTATATAAAAACTATAGCTCTTTTAATCAAGTATCATTCTCATTATCAATAATTGTAGATAAATACTCTTTGATTTTTATAATTATAGTTACAATAATCAGAACCATTGTAATAATTTATTCTATCTATTATATAATAGAAGAAAAAATGAAAAAAAAGTTCTTTTTATCGATATTTTTTTTTATTCTGTCGATAATAATCCTTTCATTTTCTGCTAATATTTTTTGATTGATAGTAGGGTGGGATGGTTTAGGTCTCTCTTCATTTATTCTTATTATATATTTTCAAAATTGAAATAGATTCAATAGATCTATAACTACATTTATATGTAATCGATTTGGTGACTTGTTTATACTTATTAGTATTGCTTTAATAGTTAACTTTTTATCT------AATTTTTGTTTATCTTTTTTGAATTCA---------AACTTGTTTAGTTTTTTGTTTTGTTTTTTTATTATTGTTTGTGCTATAACTAAAAGTGCTCAAGTTCCCTTTTCTGTTTGACTCCCTTTAGCCATAGCAGCTCCTACACCTGTATCATCATTGGTTCATTCTTCTACATTGATTACAGCTGGAGTTTTTTTATGCATTCGATTTAGAAGT---ATTCTTATGGAAGTTCACTTTCTTTTTATT---TTATCATATATTTCATCTTTTACTTTTATCATATCTGGCCTGTCAGCAATATATGAATACGATTTAAAAAAAATTATTGCTCTATCAACTCTTTCTCATATAGCATTGATTTTTTTTTTTTTAAGTATAAATAGATTTGAGTCTTCTATGATTCATTTGATTACTCATGCTATCTTTAAATCTTCTTTGATTCCTATTTTTAGAATAATAGGAATTTTATTTTTATCAGGATTTTATTCTAAAGAGTTA---ATGGGAATA---ACTATGGTTTGATATAATACAAAAAACTGTTATATTGGATTATATTTTATTGCTGTAGTTTTAACTTGTATATATTCTACACGTTTGTTATATATTTTAATTAATAACAAAATTAAT------------AGTAACATATTATATAAA---------------GAAGAGAATCAATCAATTTATTATATTTTATATGTTAGAAGAAGAATTTCGATTTTTTTAGGAGGATTTCTATTAAAA---------TTAATTACATCTTTTTGGAAATTT------CAAAAAGATGTAATCTTAATAAAATTGTTATTTTGATTGATTCTCTTCTTTATATTTATTGGAATGTTAATTTTGTCAGTTTTGATC---TTATTTTTTATCTCATCT---TCTTCTGTTATGATATTTTTTTTAATAATCATTAGTTTTTTAATATTTGGAGTTTTGATGTGATCAGTTTCTTTTAATTTTTTTTTATCTTTAATATTTTTTTTGATGACATTAGGAGGATTGTTAATTTTATTTTCGTTTATTCCAATGTTG------------------GAATTTAATTTCAGAATGAAAAATTCA---------------------------TTTATGAAATTAGAATTATTGTTTTATTTATTTATTATAATTTTTGTA------------------------TGGATAAAAATTAATTCTTTGGATTTAGTCAATTTTTATTTAAATTTT---GATGATATTATA------------------AAGTATTATATAAAA---------------ATAGACTTCACTGGTTTGTCGTTGTTTTTAATATTAATTATATTTATAATATTATTTATTTTGGACTCGATCGTCAGAACCTCTGAGGGTGCGGTCATACTTT------AAAGAAATTTAAGTTTTCTTTCTATT-------------------TATTTTTATATAAATTTATTGGTTAAAAATTAATA---GTTAATCGGTAAAATAAAATAAAAAACTTAT---------ATATGTAAACTAGGATTAGATACCCTATTATACTTATGAATAAAA--------------------AATAGAAAGAGTAAATTTTATTTAATTGAAAATTATTTGCA--TGGCGGCT--TTAAAAACCGAATTAGAGAAATATGTTTG--TAA-TAGAATCTACGCTAA-AACCTTTC---------------------------TATATAT-----TTTCTTACACACCGCCGT--CGGAAGTATATATT------TAAATTTATAAACTTT------------------------TATACA-----------------------TTATTTTATATAATAAGTCAGGTCAACGTGTAGGTAATGATGT-AGTATTGATGTATTTCTATATATA-----TTTATTA-------------TGAATTAAAATTTGTAATAATTT-----ATGAAACAGAATTTAATTGTAA--AAAAAATAAAAATATTTTCTTGAAATTGGCT-CTTTAAAGTGTACAAATTGCCCGTCAATCTAAAGGATAAGTCGAAACAAA-GTTGATTTACTGGAAAGTGGTACCTTTTGTATCAGGGCTTAAGGAATAAAT--TTGATTA-TATCTT-------TTTCCCGAAAA--AA-AAGGATCTTGTAAAATTA-AAGGTTAAA-GTTAAA-ATTTTATCTTAAAATTTTTACAAGATG-TGATAAATCATTCGGCTTTTTTTAT-ATCTGGTAACTAGACAAAAAATT---AACCCACCTGTTTATTAAAAACATGTCCTTTAGAAA-AA-AAATTAAAGGTCTGACCTGCCCCCTGCTT--AAACAAGTTAATGGCTGCGGTATTTGTGACTGTACAAAGGTAGCATAATAAATCGTCCTTTAATTAAGGTCTAGAATGAATGGTTTGATGAGGTTAAATTTGTATCAATTGTTATAAATT----AATTTTTTCTGTTAGTAAAAAAACTGACATTTTTCGAAGGGACGAGAAGACCCTTTAGATCTTATTTTCTTGGGATGAGACTAATA----AAATAAACTATAGTAAAAAAAAAGATAAATAAATCAA-AAAAGAAAGAAATAATTTTATATTTATAAAA-GAAAAAGTTACCTAAGGGATAACAGCA--TAATA-TTAATAAGAAGATCTTACTCT-TATTAATGTTTATGACCTCGATGTTGAATTAAA-ATTTCTTCAAATAGAAAAAGATTTG--ATAGTTA-GCCTGTTCGGCTATTAAAATTTTACATGATTTGAGTTTAGACCGACGTGAGTCAGGTCAGATTCTATCTTTCG-ATTTG-GTATTCTTCT---TTTGTACGAAAGGAT

Lepidopsocid_sp TTTTCATCTTTTGATCCTTTAACTAATATCTTCAAT------TTACCTTTA------AATTGACTAAGATCTTTA---------------------TTATTTATTATATTTATCCCTTCATTGTATTGAATTTTACCTTCACGAATACAATTAATTTGAAATTTAATTATTAAAACATTACATAATGAATTTAAAACACTTTTAAAAAATTCATCA---AATTTTGAAAGAACAATAATTTTTATTTCAATTTTTAGATTTGTTTTAATTAATAATTTTTTAGGCTTATTTCCATATATTTTTACTAGAACAAGTCAATTAGTTTTAACTTTAACTCTTTCACTACCTTTGTGATTAAGATTTATAATTTACGGT---TGATTAAATAAAACCAATCACATATTTGCTCATTTAGTTCCAAATGGAACACCAGGTATTCTTATACCATTTATAGTATGCATTGAAACTATTAGAAATATTATTCGTCCGGGAACATTAGCAGTACGATTGACAGCAAACATAATTGCTGGACATTTAATCTTAACTTTATTAGGAAATACAGGATCAAATATAACAATTTTTTTAGTAACCTTCTTAATTATTATTCAAATTGCATTATTAATATTAGAAATAGCTGTAGCATTTATTCAATCTTATGTAATTGCAATTTTAATTACTCTTTATTCGAGAATAATACCTATACCTTGATTATCATTATTTTTTCTTTTTCTATTAACCTTATTAATAACTAATTGT---ATTAATTATTTT------------TATTTTCAACCTAATATTTTTTTTTCAAAA------AAATATTTAAATATTAAAAAAAACATTATATTTTCTACAAATCATAAAGATATTGGTACTTTATATTTTCTATTAGGAATTTGAGCAGGAATAGTTGGAACTAGTATAAGAATTTTAATTCGATTTGAATTAGGTCAACCAGGTTTATTTTTAGAAGATGATCAAATTTATAATGTTATTGTAACAGCTCATGCATTTATTATAATTTTTTTTATAATTATACCTATTATAATTGGTGGATTTGGTAATTGATTAATTCCTTTAATATTAAGAGCACCTGATATAGCATTTCCTCGAATAAATAATATAAGTTTTTGATTATTACCCCCTTCATTAACTTTATTATTAATAAGTAGAATAACAAATGTTGGTGCTGGAACCGGATGAACAGTTTACCCTCCTTTATCAGCAGCAGTCGCTCATGCAGGTGCATCAGTTGATTTAGCAATTTTTTCTTTACATTTAGCTGGAATTAGATCAATTTTGGGAGCAGTAAATTTTATTTCAACAATTATTAATATACGATCTAACGGATTAACTTTAGAACGATTACCACTATTTGTATGATCCGTTTTCTTAACAGCAATTTTATTATTATTATCTTTACCTGTATTAGCAGGGGCAATTACTATATTATTAACAGATCGAAATTTAAATACCTCATTTTTTGATCCTGCAGGTGGAGGAGATCCTATTCTTTATCAACATTTATTTTGATTTTTTGGTCATCCAGAAGTTTACATTTTAATTTTACCAGGATTTGGTATTATTTCTCACGTTATTAGACAAGAAAGAGGTAAAAAAGAAACATTTGGAGTTTTAGGTATAATTTATGCTATAATAGCAATTGGATTATTAGGTTTTGTAATATGAGCACATCACATATTTACAGTAGGTATAGATGTAGATACTCGAGCTTATTTTACATCTGCAACAATAATTATTGCTATTCCAACAGGTATTAAAATTTTTAGTTGATTAGCAACTTTACATGGGTCAAAAATATTTTTTTCTCCATCATCTTTATGATCTTTAGGATTCGTATTTTTATTTACCATTGGTGGTTTAACAGGTGTTATTTTAGCTAATTCTTCTATCGATATTGCTCTACACGATACATATTATGTAGTAGCACATTCCCATTATGTACTATCTATAGGAGCTGTATTTGCTATTATAGCTGGGTTTATTCAATGATTTCCTCTTCTTACTGGATTAACTTTAAATAATAATTGATTAAAAATTCAATTTATAATTATATTTATTGGAGTAAATATAACTTTTTTTCCTCAACATTTTTTAGGTCTTATAGGAATACCACGACGATATAGAGATTATCCAGATATTTATACTTCATGAAATATAATTTCATCTTTGGGATCTACAATTTCCTTAATTGGTATTATATTTTTCATTTTTATTATATGAGAAAGTTTTATTTCTAATCGAAAGCCTATTTTTTCTATACATATATCTTCTTCAATTGAATGATTACAAAAATATCCTCCTTCTGAACATTCTTATAATGAATTACCAATTATTTATAATATTAATTTACAAGAAAGTGCTTCACCATTAATAGAACAACTAATTTTCTTTCATGATCATTCATTATTAATTATTACTATAATTACTGTAATAGTCTCCTATATTATAGCATCTTTATTTTTTAATTCATTTACT---------AATCGATTTTTATTAGAAAATCAAACAATTGAAATAATTTGAACTATTATTCCAGGAATCGTATTAATTTTCATTGCTTTACCTTCTTTACGACTTCTTTATTTATTAGACGAAACTAAAATACCATCTATTACTTTAAAAACAATTGGTCATCAATGATACTGAAGATATGAATATTCAGATTTCAATAATATTGAA---------TTTGATTCATTTATAATTCCTTCAAATGAAAATTTTAATTCAGAT---TTTCGACTTTTAGAAGTAAATAATCGAACAATTTTACCATTCAAAACACAAATTCGTATTTTAGTTACAGCAGCTGATGTTTTACATTCATGAGCTATACCATCTTTAGGTGTAAAAATTGATGCTAATCCTGGTCGATTAAATCAAACATCTTTAAATATTAATCGTCCGGGATTATTTTATGGGCAATGTTCTGAAATTTGTGGAGCTGTTCACTCATTTATACCTATTGTTATTGAAAGAGTTCATAAAAATAGATTTATTAATTGACTTAATTTACATTCTAATCACCCTTATCATTTAGTAGATGTAAGTCCATGACCATTAACAGGTGCAATTGGAACTATAATTTTAACATCAGGTGTAGTTAAATGATTCCAT---ATATTTAATATATTTTTATTCTTTATTGGAATAATAATTATTTTATTAACTATATTTCAATGATGACGAGATGTAGTACGAGAAAGTACCTTTCAAGGTAAGCATTCAATTTCAGTATCTAATGGAATACGATGAGGAATAATTTTATTTATTACATCCGAAGTATTTTTTTTTATCTCATTTTTTTGAGCATTTTTTCATAGAAGATTATCACCATCTATCGAAATTGGAATAATTTGACCTCCTAAGGGAATCCAACCTTTTAATCCATTTCAAATTCCTTTTCTTAATACTGTAATTTTAATTTCTTCAGGAATTACCATTACATGAGCTCACCACTCACTATTA------AAAAATAATAATAGTCAGACCATTCAAAGATTAGTAATTACCATTATTTTAGGATTATATTTTACTATTCTTCAAGGAATTGAATATTGAGAAGCACCATTTTCAATTGCTGATGCTATCTATGGATCTTCATTCTTTATAGCAACAGGTTTTCATGGAATTCATGTAATAATTGGAACTACATTTATTTTAATAATACTTATTCGACAAATAAATAATCATTTTTCTAATTATCATCATTTTGGATTTGAAGCAGCAGCATGATATTGACATTTTGTTGATATTGTATGATTATTTCTTTATATTTCAATTTACTGATGAACTTGATGAAATTTTGGATCTTTATTAGGATTATGTTTGATTATTCAAATTTCTTCAGGTCTATTTTTAGCAATACATTATTCAGCCCATATTGACTTAGCCTTTTCAAGAATAATTCACATTTGCCGGGATGTAAATAATGGTTGAATTTTACGAACAATTCATGCAAACGGAGCTTCTTTTTTTTTCATTTGCTTATATTTACATGTAGGACGAGGAATTTATTATAGTTCTTATAATTTACAT---ATAACCTGATTTATTGGAATTTTAATTTTATTATTAACTATAGCAACTGCCTTTGTAGGATATGTTTTACCTTGAGGACAAATATCTTTTTGAGGAGCTACGGTTATTACTAACTTATTATCCGCCATTCCGTACTTAGGACAAATATTAGTTCAATGAATTTGAGGTGGATTTGCAGTTGATAATGCCACATTAATTCGATTTTTTACATTCCATTTTATTTTACCATTCATTATTTTAGCAATATCAATTATTCACTTATTATTTTTACATCAAACAGGTTCTAATAATCCATTAGGAATTAAAATAAATATTGATAAAATTCCATTTCATCCTTTTTTTTCTATTAAAGATTTATTTGGATATATAATTATACTTCTAATTTTAATTTCATTAAATTTTAGTATACCTTACATTTTAGGAGACCCAGATAACTTTACTCCAGCAAATCCACTATCCACCCCTGTTCACATTCAACCAGAATGATATTTTTTATTTGCTTATGCTATTTTACGATCAATCCCCAATAAATTAGGAGGTGTTTTAGCTTTATTATTTTCAATTTTAATTTTATATATTTTACCCTTATTTAAA---AATAAATTTCGATCTACACAATTTTATCCAATTAATAAAATTTTATTCTGATCATTCACAACAATTTTTATTTTATTAACGTGGGCAGGAGCTAAACCAGTCGAAGACCCATTTATTTTTACAAGTCAAATTTTGACAGTATTATACTTTTCATTTTTTATTATTAATATTAATTTTATCTTATTATTAATTGGAATTCTTGTAGGAGTAGCATTTTTAACTTTATTGGAACGAAAAGTATTAGGATATATTCAAATTCGTAAAGGCCCTAATAAATTAGGTTTTATTGGATTATTACAACCTTTTAGTGATGCAATTAAGTTGTTCACTAAAGAACAAGTATTTCCC---AATATAAGAAATTATTATCCTTATTATTTTTCTCCTGTATTTTTATTTTTTTTGTCATTAATTTCTTGAATGATTATACCTTATTTTTGGGTTTTGAAAAAT------TTAAGATTAGGTTTATTATTTTTTATGGTATGTTTAAGTTTGGGGGTTTATGGTATCATAATTAGAGGGTGAAGATCTTTTAGATTATATGCTTTGTTAGGAAGCCTTCGTTCTGTAGCTCAAACAATTTCTTATGAAGTAAGATTGTCTTTTATTTTAATTAGTATATTAATATTAATTGGTAATTATAGATTTATAAGTTTCTATTTTTATCAAAAATATTTATATTTATATATATTTGGTTTT---CCTTTATGTTTAATATGAATGGTATCTATGTTAGCTGAAGTTAATCGAACACCTTTTGACTTTGCTGAAGCGGAGTCTGAATTAGTTTCAGGATTTAATATTGAATATGGAATAGGTGGATTTGCATTAATTTTTTTAGCTGAATATTCTAGAATTTTTTTAATAAGAATAGTATTTACATGTTTTTTTTTAGGTGCTGATATACTAAGATTATTTTTTTTTTTTAAGCTATTATTTGTTTCTTTTTTTTTTATTTGAGTACGAGGTAGATTACCACGATTTCGTTATGATAAGTTGATAAATTTATGTTGAAAAAGATTTTTATCTTTATCTCTTTTTATTTTAAGTGTTACATTTACTATTTTTCTTTCAATATTATTAAAT------------------------TTTATAGCTACATTTCTTTCTTATAAAACAATTGAAGATCAAGAAAAAATATCCCCATTTGAATGTGGATTTGATCCTATAAAATCTTCGCGTATACCATTTTCTCTTCGTTTTTTTTTAATTACCATTATTTTTCTAATTTTTGATGTAGAAATCACTTTTATTTTACCTATAATTTTAAATTTTAATAAATCAAATATATTGTTATGAATTTTTATT---ATCTTTGTATTCATTTTAATTTTAATTTTAGGTTTATTACATGAATGAAATCAAGGAGCTTTAGAATGATCAATTTTGATAATATTAATTAGTTTAGAATTTATAAGATTAATTTTATTTCTTTTAATTTATTTTAGTTTATTTAATTTAAATTTAGAAGGATATTTTTTAATAATTTTTTTGACTTTTTGTGTTTGTGAAGGTGTAATTGGGTTATCCTTACTAGTAGGAATAATTCGTTCTCATGGAAATGATTTATCTGTAAATATCCAAATTGTAATTTTATTTGACTGAATATCTATATTTTTTTTAAGTTTAGTAACTTTTATTTCTTGTTTAATTTCAAATTATAGAAATTCCTATATATTAGGAGATAATAATTCAAAATTGTTTATATTTTTAATTATTATGTTTGTATTTTCAATAATATTAATAATTGTAAGACCTAATATAATTAGAATTTTATTAGGTTGAGATGGTTTGGGATTAGTATCTTATATTTTAGTAATTTATTATCAAAATGTAAAATCTTATAATGCTGGTATATTAACTGTATTATCAAATCGAATTGGTGATATTATAATTTTAATTTGTATTGGTTGACTATTTAGATTTGGTAGT---TGAAATTATTATTTTTATATTAATATAATTACTACCGATGATTATTTAATTAGATTAATTGGTTGATTTATTATTATTGCAGGTATAACTAAAAGAGCTCAAATTCCTTTTTCTTCTTGATTACCGGCTGCTATAGCAGCCCCTACTCCTGTATCTGCATTGGTTCATTCTTCTACATTAGTTACAGCGGGGGTTTATTTATTAATTCGATTTTTTCCT---TTATTTAATATTAGAAAATTTAGAATGATT---TTATTATTCATTTCTGGTTTAACTATATTTATATCTGGTTTAGGAGCTAATTTTGAATTTGATTTAAAAAAAATTATTGCTTTATCAACTTTAAGTCAATTAGGATTAATAATAGGAAGTTTATCTATAGGATTAACTAATTTTTGTTTTTTTCATTTATTAAGACATGCTTTATTTAAAGCATTATTAGTTTCTAACTTATCTTTATGTGGAATTCCATTTTTATCTGGATTTTATTCTAAAGATTTA---ATTTTAGAA---TTAATTTCTATAAGAGAAATTAATTTAATTTCTTATTTTTTTTTTTTTGCATCTACAGGATTTACAATTAGTTATTCTTTTCGATTATTTAGTTTTTTAATATTAGGAAACTTT---------------AATTTATTTAGATTCCAT------TGTATTGAAGATAAAGATTATATTATAATAAAATCTATATTTGGTTTATTTTTGGGATCTTTATTTGGAGGAAGAATATTAATATGA------------------TTGATTTTTCCATTT------AGAAAGATAATTTTGTTACCTTTATTAATAAAATTTATAGTTTTGTTTTTAATTTTAATTGGTACTTTATTATCCACCTTTTCTATT---TTATTTTTATTTATAAAA---CATCCATTATCAGCAGGTTTAATTTTAATTATATCAACCATTTTAGTTGCCCTTATAACAGCCTATATACTTCAAACTTTTTGATTTTCGTATATTTTAACCTTAATTCTAATTGGAGGAATATTAATTTTATTTATTTATATAATTAGTTTATCTCCT---AAC---CAAAAATTTATAATTTCATCTATTCTTTTTTTAATTCCCTTA---------------TTTTTAATTATTCCAATAATAATAAATATAATTGATCCAATAATTTTA------------------------------------------ATAGAATTATCACAAAAAATTAATGATCAA---ATTAACTGAAAT------------------TTACCCCAATCAATT---AAATTATTTAATACAAATTCAAGAATTTTAACTATTATTAGAATTAATTATTTATTCTTAATTATAATTATTGTTACAAAAATTACATCTAGATTAAAAGGAGCGGTTATACATA------AGATTAAAGTAAATATTATTAATTTA-------------------TAAACTAAAAAATTTTTATTAAATTTATTTTTTAAT--TTTATTAGATAAAATGTTTTGAAAAATTGTA-----------TAATAAACTAGGATTAGATACCCTATTATT-----GTAATGGAAAAATTTTTAATTGAGTAGTAAAAAAAAAAATTAATTTTT--------AAACTTAAAGAATT-TGGCGGCATTTT---ATTCTGTTTAGAGGAATATGTTTTTATAATTTGATAATCCACAAA-AATCTTAC---------------------------TTAATTTAA---AGTTTTGTATATCTCCGT--CATAAGAATGTTTT-----ATAAGAAGTTTTTCTAT--------------------------------------------------ATATAATTTAATATAAAATGTCAGGTCAAGGTGCAGATTATGATTA-AGAA--AAAATGTATTACAATAAA-----ATTATT------------TTTGGTTTTAGTTTTGAAAAAATTT----AATTAAATTGGATTTAAAAGTAAATATTTAAATATTATTTTTTTTTGAAAAAGTT--TTAAAGTGTGTACATATCGCCCGTCACTCTTAGAGATAAGTCGTAACAAA-GTAGATTTACTGGAAAGTAGTACCTTTTGTATCAGGGTTTAATGAATATT--TTTTTTTAAAATATAT------TTTCCCGAATA--TA-AATGATTTATTTA---ATTATTTTTTAC-ATGTAACAAATGTTTAAAAATAATTTAATTGGAA-TGAAAAGTTAATCGTTTTTATAGAT-TTCTGGTAATTCGGCAAAATAAA---TTTTCGCCTGTTTATCAAAAACATGTTTTTTTGAAATTA-TAATTTAAAATTTAATCTGCTCACTGAT----AAATATTAAAGAGCCGCAGTATTT-TGACTGTGCAAAGGTAGCATAATAAATAGTTTTTTAATTGATAACTGGAATGAAAGATTGGATGAAAAATTGACTGTCTTCAATTTTATTTTTA---GAATTTAATTTTTTAGTTAAAAAGCTAAAATTTTTTTATAAGACGAGAAGACCCTATAGATTTTTTTTTATTGGGGTGATATTAAAATA-TAAATAACTTTTAAAATTATGAATTATTAATATGTAAA---TTTT-TGATCCTTTT-TTTTGATTAAT-A-GATTAAATTACCTTAGGGATAACAGCG--TAATTTAACTGGAAA-GTTCTTATTGA-AAGTTAAGTTTGCGACCTCGATGTTGAATTAAA-ATAATTAATGGGAGTAGAAGATCAT--TTAATTA-GTCTGTTCGACTATTAAAATTTTACATGATTTGAGTTCAGACCGGCGTGAGCCAGGTCAGTTTCTATCTTTAA-ATAAATATGATTAAT----TTAGTACGAAAGGAT

Liposcelis_bostrychophila TTTTCAATTTTTGACCCATCTAGTAAGCTCGTGTCT---------------------AATTGATTTATTTGTCTA---------------------ATCTTATTAATTTTTAAA------------------GTGAAAACACCATCAAGGCTTGTAGATGTCTCTTTAAGTAATTTTCTT---TCTGTGTTCATTAAAGAAATGAAAATTATTAAA------ATTCACTTATTTTTTTTCCTTTTGCCTATCTTTATTTTGATTTTGGCTTTCAACATCTCTGGGATCTTTCCTTTTACATTCACCCTAACTAGGCATATAAGAATAACTTTTTCTTTAGCCTTTCCTATATGGTTAAGACTGATAATTATAGGA---TGATTGAAA---TTCAATAGTATATTCGCCCACCTTGTCCCTTTAGGGTGTCCCACAGTTTTAATACCGTTTATGGTATTAATCGAAACAATTAGTCTAATTATTCGTCCGCTAACTTTAGCTGTTCGTCTCGCAGCCAATATAATTGCTGGGCATATGATTTTATCGTTAATCAGGATAAGGGCCTTCAACTCCTCTATTGTATTCGTATCC---TCACTATTAGCTGAGTCTATAATCTTGCTTTTAGAATTGGCAGTCGCAATAATTCAGCCCTATGTCTTCTTCATCCTATTAACCCTTTATAGCCAGATAGCCCCGTTATGATGACTAGGAAGACTAATCTTTATATTTTTTGGTTTAACCATCTTATTCCAA---ATTTTATACTAT------------ACAACTGAGATCTCAACCCTTGAGAAATTC------AATGATAAGATCACTTTTTTCAATTTTCTTTTATCGACAAATCATAAGGATATCGGATCGCTATATTTTATATTTGGGGTTTGATCGGGTCTATTAGGCCTAAGACTTAGGCTTTTGATACGAGTAGAACTTTCCTTCAATTCTAGGAGGCTGAGCTCTTCT---GTGTTTAATAGACTAATCACCTCACATGCTTTTCTAATAATTTTTTTTTTTATTATACCTATATTAATTGGGGGCTTTTCTAATTGGATAATCCCACTTTTGATTAGATCACCTGATATAGCCTTCCCACGGTTGAATAATTTAAGATTTTGATTTCTCCCCCCTTCTTTACTTTTAATTTCATTCAGAATAATCGTTGGACCAGGCGCAGGAACCGGGTGGACAGCCTACCCTCCTCTGTCAGCTATCGAAGCTCATTCAGGTTTCAGTGTAGATCTAGTAATTTTTTCCCTACATTTGGCTGGAATTAGTTCCATCCTGGGAGCCATCAATTTTATCACTACTTCAATTAATTTATGAATCGAGCCCCGGCAATTCGAGTTACTGCCTTTATTTAGATGGTCTGTGTTAATCACAGCATTTCTTTTGCTTCTTTCTCTTCCCGTTTTGGCTGGAGCAATTACTATACTTTTATTTGATCGTAATTTAAGAACTTCATTCTTTGATCCATCTGGAGGGGGGGACCCAATTCTCTTTCAACATTTATTTTGATTTTTTGGACACCCTGAAGTCTATATTTTAATTTTACCAGGATTTGGGCTAATCTCTCACATCATCTCTCAAGAGAGA---ATAAAAGATGTATTTGGCAGATTAGGCATGATCTATGCCATGCTCTCAATCGGAGCTCTAGGTTTCATCGTATGAGCCCACCACATATTCACTGTGGGCATGGATGTGGATAGACGAGCATATTTTACCTCTGCAACAATAATTATCGCGATCCCTACCGGAGTTAAAGTTTTCTCTTGGTTAACTACTGTTTATGGGAGAACAGTTACTCCTTCTTCTTCGACTTTATGAAGTCTAGGATTTATTTACTTATTCACCATCGGGGGTTTAACTGGGATTATCCTATCGAATTCAAGAATTGATGTTATTCTACATGATAGATACTATGTTGTTGCCCACTTCCACTATGTTCTTTCCATGGGGGCTGTATTTTCAATCTTTAGAGGATTGAATTTCTGATTGCCTCTTTTTCTGGGGGGGTCAGTTAATGAATTAAAAAACAAAGTTCACTTCTTCTTAACTTTTATCGGAGTTAATCTCACATTTTTCCCCCAACACTTTCTAGGTCTATCTGGTCTCCCACGACGATACTCGGACTACCCAGACCACTACACATATCTAAATCTGATTTCTTCGATTGGTTCATGAATCAGAATAATTAGCATTATCTGGTTAATTACTCTTATTTTTGATGGTGTAATAAAAAAAAATTCTGTTATTTTTATATTAACCCCTTCTTCAAGAATCGAATGAATCGAGGGGCAGCCACCTAAATTTCACACATGTTTATACTCGCCTCAAATTTTTAATTTCATATTATTAGAAAGGAGGGGGCCTGTAATAGAACAAATGAGGGAGTTTCATGATCATGCTATAATGATTCTTTTCCTCATCGTTTCTTTTTTAACTATTGTTTTTTGAGTTACTTTAACTAATAAAGGATTA---------AACCTTAACATTTTGACAAGAGAAGTTCTTGAGATATTTTGGTCATCTCTGCCTGTTTTCATCTTATTAATTCTTGCTATTCCCTCTATTCAAGTTCTGTTTATAATAGAAGAAGTTATCTCCCCCCTGATAACAATTAAAATCATAGGCAATCAATGATTTTGAACGTACGAATATAGTGACTTAACTAATGTAAAA---------TTTGATTCGGTAATTAGTAAGACTAACATT------------------TTCCGTCTTCTTGATGTTAATAAAGCTCTAATTCTACCAATTACCACCCATGTCCGTCTTCTCTTATCGTCAAATGACGTGATCCACTCTTGAACTCTGCCCTCATACGGGTTAAAAATTGACGCCAATCCAGGCCGCCTAAATATAGGCTCTCTTTACAGCTATCGTTCGGGCTACTTTTATGGTCAGTGTTCAGAAATCTGTGGGGTTGACCACTCATTTATACCTATTAAAGTTGGTTTCACTTCTCTGGACTGGTTTAAGAATTTTATAAAGAGGGTAAAACTGAGAGACTTTCATCTAGTAGATATCAGGCCGTGGCCTTTAATAATAAGCTTGACTACAGCTAACACCATTCTGTCTCTATACATCAAAATGAATTTTTTGTCATGTCTATTATTGATGACCATTTCTATAATT---------TTTGTATTTTTCCTATGATCACGAGATATCATACGAGAGAGAACCTTTCAGGGAATACACCCTTTAAAAGTTCAGCTTTCACTTAAATACGGAATAATCCTGTTCATTACTTCTGAAGTTATATTTTTCTTGTCCTTTTTCTGAACATTTTTACATTCGGCCCTTAGCCCCACAAATGAAATTGGTAACTCTTGACCAAGATGGGGTGTTGAGCCTATTAATCCCTTTGGAATCCCTTTGCTGAACACTCTAGTCTTAGTTTCATCAGGGGTTTCTATTACATATTCTCACCATAGAATATTA------AATCAAAATTTTAATTTTACCATTCTCTGAGTAGTAATTACAGTTCTTTTGGGAGGGTATTTTACGATTCTACAGCTCATGGAATATATGACATCATCATTTTCTATCATGGACTCAGTTTATGGCTCAATTTTTTTTATCTCTACAGGCTTTCATGGCATCCATGTTCTAGTTGGCACTCTGATGATTCTATATTCCCTCATTCGTCTATTCAGCTTTCAATTCAGCTCTGCTCATCATTTAATGTTTGAATTCTCTTGTTGATACTGACACTTTGTTGATTTAATCTGACTTTTTCTTTTTCTATCAATTTATTGATGGTACTTCTGAAACTTGGGGTCGCTTCTCGGGCTATGTCTTTCTATTCAAATCTTAACAGGAGTGTTTTTAACAATATTCTTTAAAGCAGATCTCAGGCAATCTTTTACCAGAGTAGTAAGAATTATAAATAATATTAATAACGGGTGAATCATCCGGTTTATTCATTCCACTGGGGCATCCATATTTTTTATTATCTGCTACGCCCATGTCGGAAAAGCACTATTTTTCTCTTCATTTTACTTTTGA---AAGGTTTGAGTTTCTGGACTAGTGTTAATTCTTTTACTCATAATAGAGGCTTTTCTAGGCTATGTCTTACCATGAGGACAGATATCATTTTGAGGAGCTACAGTAATCACAAATTTAATTTCTGTTATTCCTTACTTTGGCCCCCTTGCTGTTCAGTGACTGTGGGGGGGCTTTAATGTTGGTGATCCCACACTAACACGATTCCTATCATTTCATTTTATCATTCCATTTATCATGATTGCTATAAGAGGAGTACATTTAATTCTTCTTCATGAGACTGGGTCATCAAACCCTCTAGGCATGCCTTTGAACATAGATAAAGTTAGATTCAGAAAATTTTTTATTATTAAAGACCTAGTTACCCTAGCTCTGGTTTTGTTAGGGCTAATTTTACTTAGAACAATATCTCCTTTTATATTTATAGACCCTGAAAATTTTCTTAAAGCTAACCCAATGGTGACACCCATCCATATCCAGCCTGAGTGATACTTTCTGTTCGCCTATGCAATCTTACGATCTGTTCCTAATAAACTTGGGGGGGTGTTAATGTTAGCTTTATCCATCATTATTATTTTAATTCTCCCTCTCTTATCTAAAAACAAGATAAAAGGACTTAAGTTTAGATTTTTG---AAATGACTTCTTTACTTCCACTTTGGATCATTCTCTATTTTAACATGACTGGGCATGCAACCTGTTGAAGATCCCTTCATTTACTTAGGTAAAGTTTACTCAGTTCTTTATTTCATTTTTTACTTCCTCTTTATCAATTTAATCTTACATATCGTCTTGATACTTTTAAGAGTAGCTCTGTTTACACTATTTGAACGTAAAATTCTAGGGCTCATTCAACTTCGTAAAGGGCCTTGTAAAGTAGGGCCTTTAGGCCTTTTACAACCTTTCTCAGATGCTTTAAAACTTTTTTCAAAATTTTCCTCAGCTCCA---ATCAAAGGAAACTTAATACTTTATTACTTCACTCCTCTGTACTTTCTTATTCTTTCATTAGTCTTTTTTTTGAATAAACCATTCTTATCAACTTCTTTT---------TTTACTTTATCAATTTTAGTTTTACTCTTTCTATATACCACCAGGGTTTACACTACTTTAGTAACCGGATGATCTTCTAACTCAAAATATTCCCTTATTGGGTCTATGCGTAGAATTGCCCAGTCCCTCTCATATGAAATTACCCTCGGTCTTCTTTTCTTCAGCTTTGCCTTCATCATGTCTTCAACTTTAATATTTAAAATTATAAATTTTAACTCATCAATACTTCAACTATTTTATTGTCCT------CTTTCTATGATTTTATTTCTGAACTACTTGATTGAAAGTAACCGAACTCCTTTTGATTTATCTGAGTGTGAATCAGAGCTAGTCTCTGGGTTCAACGTTGAATTTGGAGGGGCTGAATTCTCTTTAATTTTTTTAGGGGAAAATTTAATATTAGTTTTCAACTCTTTAGTATTATCCTTCTTTATTGGCAGTATCTCAGTATATCTAATTTTTTGACTTGTGATTATTTTCATCAAA------------GTCTCTATCCGAGGTGCATACCCACGCTACCGATTAGATAGAATGATGGAATTATGCTGATTGATCTACCTCCCCCTAACCATTGTTCTTCTAAGTGTTTTAACTTTAATCCTCATAGTGGTTTTCTTAATT------------------------TTTTTTGTTCATATAGTTAACAGAGTAGGTAAAGGTGAGAGATACACAGATCATCCATTTGAGTGTGGAATTAGAAGAAATTTTTCTTCACGTATTCTCTTTTCCCTCCCCTTTTTTTTAATCACTTTACTTTTTTTAATATTTGATGTTGAGATTATCCTGCTTTTTGTTTTAATTTTTTCT------GATCTGTCAGTGATATTCTTTCTTTTATATATCGTGATTTTATTC------CTTCTTATTGCGAGACTCCTGATAGAGTGATATTATGGATCTCTAGTATGGATGCTTCTTATAATAATCCTGAACATAGAAATAATCATGGTCTTGATATTTTTATTTATGTTTAATTATAAGATTAAA---------------ATTGTGATGATTATATTTATAGTAATGATAGTATGTGAAGCCATTATCGGGCTAATCTATTGTGCTTGCTGGTCATTAATCTTCAACAATCTTAAATCAATTTCATGAAGATGATTGTTTTTTTTTGATGCCTATTCAGCTTCCTTCTCTTCTGTGGTTCTTTTAATCTCAGCATCAATCGTGTTTTATTCCATATCTTATATACAACAAGAAAAGGAAAAAATCAAGTTTTTTTTAACCCTATATATGTTCATTCTATCAATGCTAATCTTAATTTTCTCTTTTAATATTTCTTCCCTTCTAGTCGGTTGAGATGGGTTGGGTGTAACTTCCTTTCTATTGATTTATTATTACCACTCATTAAAAAGAACAAACTCCTCTCTCATTACATTAACATTAAATCGGGTTGGAGATTTAATAATTATCTTTTCAATTACTATAGGACTCACAGTTTATACT---TGAAATTTTTTCTTTAGGGTAGAGACAATT---------------------AAAGTTTTTAATTTTCTTTTAATAACTGCAGCATTAAGAAAAAGAGCTCAGCTCCCCTTTTCTTCCTGGCTACCTCTAGCTATAGCTGCCCCCACCCCTGTTTCCTCTCTTGTTCACTCTTCAACTCTAGTCACTGCTGGTGTGTATCTCCTCTATCGA---GCTCCT---TTATCTATAATTAGATGCCTCAGAAATTAT---ATTTTTTTCATTACGAGCCTCACGCTAATTATGAGAAGGGTTTTAGCATTACAAAGATTCGACTTAAAGGAAATTGTCGCCTTTTCAACAATAAGGCATATCAGCTTAATGATAATAGGAATCTCAAATGGTCTTTATAAATTTTCATTTTTTCACCTTTGTACCCATGCTTTATTTAAGGCACTTTTAATCTCAAGATGCTCAATAATGGGGCTTCCATTTATGGCGGGGTTTTACTCTAAAGACGGG---CTAATTGAT---GAAAGCTCATTT------------TTATCAGTTTCTATGCTTTTTGTCATTCCTGTCCTTTTATCTTCCCTATACACCATACGCTTATTATACTATATTTGTTTATCTAAAACT---------------TCTGGGATAAGATTAAAA------------------ACTAATGACATGATAAGGTTTTCAATATTTTCTTTAGCATGATTTTCTATTTTTAGTGGAGCAGCAATTCAATGA------------------AGACTATTTCCTCTC------CTTTTTATTTCAATAATGCCT---CAGTTAAAAACCTTAATTTTAATTATATTAATTATAAGAATAGTGATATTATATATTACGGTT---ATATTTACGTTAGAGAGT---TCAATATTTAGGGCCTTAGTTTATATGTTTGTAATAATTTTTTTAGGCAGAGGGATTATAGCCACTGTTTCAGGAAGAGTTTGAATGAGAATGATTTTTTTAATTTTTATAATTGGGGGGTTGATGGTATCATTTTTCTATATAGTAAGGTTGACCCAC---AAT---ATAGTATTTAGAATTTCCCCCCTAGTATTA---------------------------TTTATCAGGGTATGTCTAGTCCCTTTTAGGCTTGCAAGTTTAGGTAAT---------------------------------------TTTCATAATTGTTTAGACTATTTTTTATTTTCT------------------------------------------------------------TCTCCAAGCTATCTGGTGGTTGTTTTGTTTATATTATTCTTGCTTTTCTTAATTCTGTTTATAATTGATTTTAAACTTAAGGCTATAAAAGGAGCGGTAAAACAT-------AGCCCCATTTTATTCTTTCT-------------------------TAGATTT---TGTTTATAGGATGGTTAACTTAGAG---TTTATTGAGTCTAATGACATAGGAAGTAGGT----------GAAACAGACTAGGATTAGAAACCCTATTATTTCT---------ATTACTCATATTCTAAAGACTAACAAT-------GATCTG------------------------TGGCGGCTCTCT----TTCAAATTAGAGATCCATGGACC--TAA-TGGACAGCCCACCCA-AATCTTCATATG-----------------------TTTAGCTCTA--CATTTTTTATATCGCCGA--CTGAAAAAAAAATC--------ATAAATTTTTATTT------------------------TGTCAGTTTAA-------------------------TACTGATAAGTCAGGTAAAGATGAAGATTATAGCAT-TGAAAGTTTGTGGATCA----AAA-----ATTTTCA-------------GGAATTAGGTAGTTGAGACAC------TATAAAAATGAATTTAAAAGTAAA-GCTTCTTAATTAAAGTGACTTGAAAATTGTT-AAAGAGAGTGTACATACCGCCCGTCACTTCTAGGAATAAGTCGTAACATG-GTTAATCTACTGGAAGGTGGTACCTTTGGTATCAGGGTTTAT--AGATTTC-TGAAACTATAA-TGTT------ATTCCCGAATA--GA-TACGATTTAATTA---GATGAATTTAATTGTAGCAAAA--ATTTTTTAAATTTTAATTAGAAG-TGATAAGCTTATCGGGTGTC--TAG-ATCTGGTAACTCGACAAAATTAT---CAA-AACCAGTTTACCAAAAACATGTCCCTCTTTAA-----TTTCTTGGGTCGGCCCTGCTCACTGA-------ATTTTAAAGAGCCGCTTTA---------GTGTTAAAGTAGCATAATCAATTGTCCTCTTATTAAGGTCTAGAATGAACGGGAGAATTATTGATAAATTATATACACTGAAATTTAAA----AAATTAAATTTTAATTTATAATGCTTAAATTAATTAAGGGGACGAGAAGACCCTA-GGATCTTTTTTAGCTGGGGAAATTAGAAAAAT-AAACT--TTTCTAGATTTAAACATTATAGATGTTTG------TTTAAGATTAAAGTAAATTA-------A-GATAAAATTACCCTAGGGATAACAGCG--CCATAACAAGTCAGATGATCAGAATTT-TACTTGTGATTGCGACCTCGATGTTGAATTAAA-ATTAG----AGGCCTGGAAGTTCAGGCCTGATTCAGTCTGTTCGACTGGTTAA-TTTTACATGATTTGAGTTCTGACCGACGTAAGTCAGGTTAGTTTCTATCTCTTA-T---TTTCACCCAGG------AGTACGAAAGGAG

Pediculus_humanus ATATCTTCTTTTGACCCTTCTACTTCTATTATTCTAGGA---TTAAAAGTA------AAATGATTTATTATTCTT---------------------TTTCCTTTCTTTTTTATAACTGGAAGTTATTATTTAATTCACTCAGGCTACCGTTTTTACGTGAGTTTTGTGTTAAATAATTTATTAATAAAACACTACATATCT---------------------------------ATTATTAGGTTATCTGTTTTTATAATAATTTTAACCCTTAATACAATTTCACTTATGCCATTGGTGTTGCCTTGCACTTCGCATTTAAGAGTTAATTTAGGACTTTGTTTACCTTTATGGATAAGAGGGGTTGTTTACTCT---TTAAAAAGCTCTATGCGAGGGTTTCTAGCTCATCTTCTTCCTTACGGGAGTCCAACCATACTAAGTCCGTTCTTAGTGGTAATCGAGTTGTTAAGAGTCTCGATTCGTCCTGTATCTCTAAGAGTTCGACTTCTAGCGAATATTACAGGAGGACATTTAATTATAAATCTTTTAGAAGAAGGCTTATCTTCAGCTGTACTTCTTGTACTTCCTTTCTCAATAGCAGCGTATGTTCTTCTCTTAGCTGCTGAGCTATTTGTCTCATTTATTCAGTCTTACGTTTTAAGTAAACTGGTTTCAATTTACTGAGAACTTTGTCCGTCGATGTGAACGTTGTATTATATAATTGTAATATTTGTATTATATTTTATATTAACT---ATGATTTATTTTATAAAAATTGATAATTCAAATTATTTAGAGAATTTTAAATTA------AAAAATAAAACAATCTCCAATCTTTCTATATTTTCTACAAATCATAAAGATATTGGATTTTTATACTTATGCTCTGGAGTTTGGTTTGGACTTTTAGGCTTAAGGTTAAGGTTAATAATCCGGTTAGAACTTTCTAGAACAGGCTTGCTTTTGTCTGATAGACACCTATATAACGTATTTGTTACTTCTCACGCTTTTGTAATGATTTTTTTTATAGTTATGCCTGTAATAATAGGCGGTTTTGCAAATTGATTAGTTCCTTCAATATTAGGGTCTCCAGATATAGCATTTCCTCGTATAAATAATATGAGTTATTGACTTCTCACACCCTCTGGGATTTTGCTTATTAGTAGCTCATTTGTTCAAGGTGGTGTGGGTACTGGCTGGACTGTTTATCCCCCTCTTAGGTCTCTAGAAGGCCAACCTTCTGTTTCAGTTGATTTAGCTATTTTAAGTCTTCATTTAGCAGGAGTGAGTTCGATTTTAGGATCAGTAAATTTTATTAGAACTATTTTTAACATATGGCCTCAATATTTTGGCTTAGTTCGACTGCCTTTATTTTGCTGGAGAGTGTTGGTAACAGCCTTTTTATTATTACTGTCACTTCCAGTTTTAGCTGGAGCTATTACAATGCTCTTAATAGACCGTAATTTCAATTGCTCATTTTTTGATCCTTTAGGGGGTGGTGATCCTGTTTTATACCAACATTTATTTTGATTTTTTGGACATCCTGAAGTTTATATTCTTATTCTTCCTGGATTTGGTCTTATCTCTCATATGGTGGTAGATTGTTGTGGAAAGAAAGAAGTTTTTGGGTCATTAGGAATGATTTACGCAATATCCGCTATTGGGGCTTTAGGTTTTGTAGTTTGAGCACATCACATGTTTACAGTTGGATTAGATGTGGATAGACGGGCTTATTTTACTAGCGCTACTATAACAATTGCAATTCCAACGGGAGTGAAAGTCTTTAGGTGATTAGGCACTTTGTTTGGCCCAAAATTAAAAAGGAGAATTAGCTTGTTATGATCTTTAGGATTTATTTTCCTTTTTACAATTGGAGGTTTAACAGGCATTGTTCTTTCTAACTCATCCGTAGATGTTTCACTACATGACACTTATTATGTAGTTGCTCACTTTCATTACGTTTTATCTATGGGTGCTGTATTTGCTATTTTTGGCGCTTGAAACCACTGATTCTCACTAGGGACTGGACTTAAACTTCGTAAGTCTTTTATAAATGTTCACTTTTGGTTAAGATTTGTGGGAGTGAATTTGACTTTCTTTCCTCAGCACTTTCTCGGGTTAGCTGGGATGCCTCGACGTTACTCAGACTATCCTGACGTTTACCTGAGGTGAAACAAAATTTCTTCAATAGGAAGGCTAATTACTACTTTGGGTGTTGTAATCTTTCTTTTAGCTCTTATAGAAAGGTTTTCTAATCCTCAAAAAATTGTATTTAGAGATGCTAGATTGCAAGACCTTCCGCGTCTAATGGGTATACCAGCAAGAATGCACTCTCATTTTACTCTTACATTTACTAGAGTATGTGGATTCCAAGATAGAAATTCTCCTTTAATAGTTTTTGTGTGTGATACTTATGACCTTGTGTCTATTGTTTGTGTGGGGGTGATCTCCTTAGTAATGTACGTGGCTGTTAGTTTCTTTTTTATAAAATCTTGA---------AATTACTATTTTATAGGTCTTGAAAGATTGGAGATTGTTTGAGTTATCTTACCCTCACTCTCTTTAGCAGGGTTAATTTTACCGTCACTTCATTGTTTATACTTAATAGACGAGGTTCTTTCTCCCGCTATGAGATTAAAAGTGGTCGGACATCAGTGATTTTGGTCTTATGAGTACGGGGATTGAGAAAATATTGAA---------TTTGATTCATATATGATAAAATTAGAGGAGCTTGACTCGTCATGTCCTTTTCGACTTTTGGAGGCTGATTTAAGCGTGTTTATCCCTTATTTGACTGAAGTGCGTGCTATTGTAACATCTGCCGATGTTATTCATTCTTGAGCAATTCCTATAATAGGAGTAAAAGTAGACGCTATTCCTGGGCGTTTAAACCATGCGCTTATTTACTCATTTAAAATTGGCACATCTTATGGTCAGTGTTCTGAGATTTGTGGTGCTTATCACAGGTTTATGCCGATTAAAGTCACTACTCTTCCAAAAGAAGACTTTATAAAATGAGTTAAAGATTTAAAATTTCACCCATTTCATCTTGTTGATGTAAGACCTTGACCTATTTTTTTAAGATTTTCTCTTTTATTTTCAGCGTCCATAACATTGTGTTGAATTAACGGGCTTTATTCTTTTTATATTTTAATAATTAGAATTGTTGTTTCATCTTTAATTGTTTCTTTTTGGTGACGAGACGTTACTCGAGAGGCTACTTTTCAGGGTAAACATACAATAGAAGTAATTGCTGGATTGCGTTTAGGAATGCTTATGTTTATTGCTTCAGAGGTAATGTTTTTCTTTTCGTTTTTTTATGCTCTGTTTTTTCTTTCTTTAAGGCCTGACGTGTCATTGGGACTTCTTTACCCTCCTGTGGGTGTTAGCCCTGTAGGCGTTTTAGGAGTTCCTCTTTTAAATTCTATTTTATTACTCTCTAGCGGTGTGTCTATTACTTGAGCTCATTATGAGCTTTTA------AGGAAAAATATTTCTTCTAGGCTTATCGGCTTATTAATCACTTTAATTTTAGGTCTAGTGTTTCTAACATTTCAGGCTGTTGAATATAAAACAAGCTCTTTTACTATGGCTGATAGAAGTTTTGGCTCAGTGTTTTTTCTAATAACCGGCTTCCACGGAGCACATGTTTGTGTGGGAGTTGTGTTTATTACAATTAGAACCATTCGGCTTTACTTAAATCACTATAATAATAATCATCACTTAGGGCTTGAGCTAGCTGCATGATACTGACACTTTGTCGACGTAGTGTGGTTATTTTTATACCTAACTCTTTACTGATGAATACTCTTGAAATTTTGGGTCTTATTAGGCTTGTTTCTTTCAATTCAGATTTTAAGAGGTCTTTTTCTGGCTTCTCATTATGAGGCTTCTACTAAT---TCGTTTTGAAGTGTTATTTTAATTGATTTTGATGTAAATAGAGGGTGGTTGATTCGTAGTTTTCATGCTAACGGCGCTTCTTTTTTCTTCATTCTTGTCTACGTTCATATTTGGCGTGGTTTATGATTTGGTTGTTTTACACAAAAA---TATGTTTGATTTTCAGGAATTTCTATTCTTCTTCTTATAATAGCAGCAGCTTTTATGGGGTATGTTCTTCCTTGAGGTCAAATATCTTTTTGAGGAGCGACTGTAATTACTAATCTTTTAAGTGCTATTCCTATTGTTGGAAGAGATTTGGTTATTTGAGTGTGAGGAGGGTTTTCAGTTAGACATCCTACTTTAGAGCGGCTGTTTACTCTTCACTTTCTTTTACCGTTTGTCTTATTGGGGTTTGTTATAGCTCACATTATTCTCCTCCACCAACACGGTTCTAGAAATCCTTTAGGATTGGATTTGGATAGTGATAAAGTTTATTTTTATCCTTACTTTTATCTAAAAGATATTTTAGGAGGTTTTGTGTGTTTATTTTTATTTGTTTTGATTTGCATTTATTCGCCGGACTTCTTCATGGACCCGGATAATTTTGTTGAATCAAACCCGATAATTACACCTCCACACATCCAGCCAGAGTGGTACTTTCTATTTGCATATGCAATTTTACGGAGTGTACCTAACAAGTTAGGAGGAGTTGTAGCTTTGCTTCTAAGAATTTTATCTCTGTCTTTTATTAGCATA---------GGAAGCTCTGTCTCAAGTCGCTTTAGAATAAGGCGAATGATTTTAACTTATTCTTTTACGAGAGTTTTTGTTATGCTCTCATGACTTGGCTCTCTTCCTGCTGAGTATCCTTTTACTCTGCTAAGTCAAGTTGTAAGAGTAATTTATTTCATTCAAGTAATTCTATTTATGCAGTCAGTATTTATTGTTTTATCACTTTTAATTTGTGTTGCTTATTTCTCCTTGTTTGAACGTAAACTTCTTAGATTAGAGCAAATCCGGCTTGGACCAAATAAAGTAGGACCAATTGGTATTCTTCAACCTTTAAGAGATGCTCCCAAATTATTATCCAAGACTATTTGTCCGCAG---AGAGAG------TCATGAGAGTTATTCATTATGCCGTTTATTACATTTATGTTAAGCGTTTCCTGATGGTACCCGCTCTATTTTCCAAAAACTTTATGA------GAATCAAATAACTCACTTTTAATCCTTATTTTTATCTCAAGAGTAAGAGTTTATGCATTAATTTTTACAGGGTCACTGCCAAAATCAAAATATTCGGCTCTAGGAAGACTGCGTGCAATTACTCTTTCTATTTCTTTTGAATTAGTTTTTTCCACTGCTATGCTAAGTATGGCTGTAGTTTTTAATTCTTTTAGCATTAAATTTATAGCAACCAATCAGAGC---GTGCCTAATATTATCTCAATAATT---GTAGTTGGAATTTTAGTATGAACCTCACTAATTGCTGAATGCGGACGAACTCCTTTTGATTTACCAGAAAGAGAGTCTGAATTAGTAAGAGGTTTTAACGTAGAGTACGGAGGTAGGCGTTATGTTTTACTTTATTTAAGAGAAAGATTGTTACTTACCATTTCATCTATTATTATAAGCATTTTATTTACCTGC---GGGTATAATCCATTAGTGGTTTTAACATGAATTAGAATTTCT------------ATTGTTATACGAGCTAGAAACCCTCGCATTCGGTACGATAAATGCATAATATTCGGGTGAGAATTTTCAATTCCTTTAATCTTAATATTTATGAGATTTACTATTTTGATTATTTTGGCTATTATTTTAGCT------------------------TTAGTTACTTTGGTTTTTGTATCTCAAAGTCCTAAATTAACTCTAGATAGGACGCCCTATGAGTGCGGTGTAATGCCTTTTAGAATAAGAACTCTGTCTACACACATTCATTTTTATGTTGTAAGTGTGGTATTTCTTATTTTTGATGTAGAATTAGTAGCTACTTTGCCTGTTGTTACTTCA------AGGCTATTAGAGAAAGATTGATTGTCAATT---TGACTTTTAATTCCACTTATTCTCACCTTAGGGTTGCTTTTAGAACTTCATTATGGAAGTTTAGATTGAAAAATTATATCTTCTCTTATTTCTCTTGAATTAAGTTGATGCTGGGTTTACGTAATTATACACATTGCACTTTCTGAA---AGGTTAGACGCTCTTTTAAGATCAGAGGTTTTAAGTGTTATTGTTTGCGAGAGAGTTGTAGGACTTTCTTTACTTATTAGGCTAACTTATGGGTGAGGAAGGACTGGTTTCAGGGGGGTGGAAATTATTATTGTACTGGATAATTTAAGCCTGACTTTTTTGCTTATAGTATTAACAATTAGGTCGCTTGTTATAGCTTATAGAAACAATTATATAGCAGGTCATAATCTTGGCGGTGATTTTTACGTTTCCATGGTCTTATTTATTGTAAGAATATTACTTCTATCTCTTAGAGGCTCAATGTTCTGATCTTTTATTGGGTGGGACGGTTTAGGAATAATAAGTTTAGTTTTAATTTTATTTAATAAAAGATGGAGTTCTCAAAAATCGGGAGTGATTACTTTCCTAATGAACCGATTAGGTGATTCTTTTATAATTATTTGCTCGTCATATTTAAGAGTGTGAGGGATA---TGCGAA---------------------------------GTTTATTGGTTTTGAATTTTAACTTCTTTGTATTTAATTGGAGGGGCTTCAAAAAGAGCTCAATTTCCATTTTCTAGCTGATTGCCGGAAGCTATGGCAGCCCCTACTCCCGTGAGTAGGTTAGTTCATTCTTCAACATTAGTAACTGCTGGTATTTATGTTTTAGCTCGTTATGGAAGA---ATAATCGATAGTTTTTACATT------------CTTACCTACTTATCTTCTATTTCTATTATTATTTCGGGTGTTTCAGCTCTCTGAAGGAGAGATTTAAAGAAAGTTGTAGCTTATTCAACTCTTTCTCACATTAGCTTAATGCTTTTTTACTTATCTGAAGGAAGGGTGGAAGGAGCTTTAATCCACATGTTGACGCACTCTGTGTTTAAAAGGCTGTTATTTTCTTGCCTTTCAATGGCCGGACTGCCGTTTCTTTCGGGAGGTTATTCAAAAGAGGTG---TTA---------CTTATTTTAAGTTTAAATAGATCAATTATGAAGCTTATTATATTTCTTACAGCTGTAATTTTCACTAGAGGTTATTCGTTTCGCATTATTTACTTACTTTCTAGAAAT---------------------ATTAATATAACACAAAAT------------ATTGCAGTAAGGAGGTTGTTTAGCAGCCCCCTTAAACTGAGGCAAATGCTTAACGTTCTA---------ATTTCAGCTTGAATTTCCTCAAGTCCTGGCTACTTATCTAAACTC------AGAAGGCAG---AGAGTTAATTTAGAGGGTAAAATTATAATACTCTTTATAATTCTATTTGGTGTGGTTGCTATGTTTTTTACGTTT---CTTATTTTAGTAGGCTCT---GATCTTTTGCTGAAGCTTTTTTCTCTAGCTATAACTGTTTTTACAGTCGGACTGCTTATTCTTCTTAACTCTTACTCTTCATGGTCTTGGTTGCTTTTATGGTTAGGGATTTTGGGTGGTTTAATTGTCTCACTTTCTATGGCTTTTATTGTAACGCCA---AAA---ATTAATTCCTCAAAGGACTGGTCTAGAAGATTAAGA---------------------TTTATAAGAGCATTTTGAATTTTAACTTCTGTTATACTATCTGCTATA------------------------ATATGAAAAGTAGAATTTAAAGAATGATCTGACTTGTACTCTACAGAG---AGTGTAAGAGAA------------------ACTTATAATTCACTA---CTTATAGATCTTAAGGTTTATACAGCTGTGGTTATTCTTTTAATT------TACATTTTAATGCTTCCTGTCATAGAAGTTTTAACGTCACCTTATAGACGTGCGGTCATACATTA-----AAGTCAAA-TTAATTGGTTAAAGCTAATAAATAATTACTATTAAGTACAACAACTCTACTTATTATTGTAAGAGTAAAAT---CTTTATAAATAAGATCTTACGAGGCGCTAGC-----------ATAAAGACTAGGATTAGATACCCTATTATGGGCGTGAGTATGAAAGTTTATTAAGTTAATAGCCTTAATTAAAGCA---------------AAACCTATTTACTA-TGGCGGCTGTT----AGTCTCACCAGAGTCGTATGTCCT--TAA-ACGAAACTGCGCGAT-CATCTTACCTTTTAACTTTTGCTAATATAAAATAATTACATTATAGCAATCTGGCACGTCGCTGTACTAAAAGTGAAGATT-----TTAAATAACTCATCTTC------------------------TAATTACTTATTAGCA-------------TTTATTAATAACGTA---CAAGTCAAGACGCCGTTT----TAAGAGGCTTAAGATG---TACGATTTA-----ATTGATTTG---------ATAGAATTTATAAGAATGAAAACTTTCTTTATTAAAAT-AATTTGGAAGTAAA-ATAGGGTTATTATTCTTATTTGAAATGAGCTACTAATAAGTGTACATATCGCCCGTCATTCCAATGGACAAGTCGTAACAAA-GTTGATTTACTGGAAAGTGGTACCTTTTGCATCAGGGGTTAAGAAATAA--GATTATTAAATTAATAA------CCTCCCGAAAG-AAGC-AGGATCTTAAACTTTATTGTTGGTTGTTGTTTCATTAACATTAA-TAATAATATTTAAGAAGCTAAAAGTTATAACGACTCTTCTGATCAC-TGGTAACTAGGCAAAC-ATTT--AGCCGGACTGTTTAATAAAAACATTTCCTCTTGCATAAA---AGGG-AGGTAAAGTCTGCTCGGTG-----TAATTGATTAACAGCTGCAGTAACT-TGACTGTACAAAGGTAGCGTAATCACTTGTCTTTTAATTGAAGACTAGAATGAACGGCTTAACCAAGCTAATTCTGTCTCTTTTTAACCTTAAG---AAATTTAAGATTTAAGTTAAAACGCTTAAATTATTTAGAGGGACGAGAAGACCCTGAAGATTTTTTTTATTTGGGATGAATGT--------AATAAACATTATG----GTTTACTAAGACTTA---AACGTCTTTATTCTGACCCGTTTAAACGAGAATCTGTTTAAGTTACCTCAGGGATAACAGCG--CAATGTCTTTTATTA-GACCTTATAAAA-ACAGAAGTTTGCGACCTCGATGTTGAATTGAGTTAAACTTTAAGTAGAAGAGTACTTA--AGAGTAG-GACTGTTCGTCTTTTAATAGCTCACATGATTTGAGTTTAGACCGACGTGAGTCAGGTCAGATTCTATCTTCTAATATTAAGCA---ATCTT-TTTTGTACGAAAGGAC

Haematopinus_suis ATATCAGTGTTTGACCCCTGTTCTACTTTGTTGAGGTTAAATTTACCTTTG------AAATGGTTACTTGTGGTT---------------------TTAGTCGTATTAAGACTAAGAGGGCGTTATTGAATTCTATCTTCAGGGTTGCAGTGTGTAATAGTGTGGGTTAAAAATGGACTAATTCATGGATTACGTGAGTCATATAAAAACTACAAA---------CAGTTTATACTCATTCTTCACACACTGTTTTTTTTTATTTTTTCTAGTAATTTTATAGGACTATCACCTTTCATGTTTACTCTCTCGTCCCACTTGGTTTATAATTTAAGATTATGTTTCCCATTGTGGTTAGGAGGAATTTTATATTCG---TGATCAAAATGTTGAAAGAAAACATTGGCACATTTAACCCCTGTAGGTAGTCCAGTAGCTTTAGCTCCCTTTTTAGTGTTGGTAGAAACTGTAAGGTTGATTATTCGACCAATTAGTTTAAGTGTTCGATTGATGGCTAACATGACAGCAGGTCACATAGTAATTACTCTTGCTGAACAAGGGGCCATGTCTGTAGCTTCTTATGTTGGGAGGTTTTATGTGTTGTTAGTAATAGTATTATTATTGTTTGAGTTAGGGGTGGCTCTAATTCAGGCTTATGTGTTCATAAGACTAATGTCCTTGTATTGGGAAATGTCTCCTATGTGATGGTTACTGCTTGAAGTTTGATTTTTTGTGAGTTTTATAAGGTGTTGTAGA---TGTTTATATTGGGAATTGTTTGTAGAAAGATTAGCGTCTCCAAAAGTGTTTTTG------AAAGTCTTAAAAGAAGGTGATTACGTGTTACTTTCAACTAACCATAAGGATATTGGTGTTCTGTATTTGATTTTAGGTGTTTGAGCAGGTTTGTTAGGGACAAGAATAAGGTTGTTGATTCGCGTTGAGTTGGGTAGAGTGAATAGATTAATTTCTAGTGGACATGTTTATAATGTATTTGTTACAGCTCATGCGTTTTTGATAATTTTCTTCATAATTATGCCAGTAATAATTGGAGGTTTCGGAAATTGGTTAGTACCAACTATGTTGGGGGCTCCTGATATGGCATTTCCTCGAATAAATAATATAAGATTTTGGCTATTACCACCATCACTTTTTTTATTAATCTCAAGATTGATTGTTGGAGGAGGTGTTGGTACTGGCTGAACGGTATACCCCCCACTAAGGGGGTTGGTCGGACAGCCCAGAAGGTCTGTAGATTTAACTATTTTTAGACTACATTTAGCGGGTTTAAGTTCCATTATGGGTGCTATTAATTTTATTTGTACTATTGTAAACATGTGGGTTTGTGGAAAAAAGTTAGAACTATTACCTCTGTTTTGTTGGTCAGTTTTAATTACTGCTGTCCTACTGTTACTTTCTTTGCCGGTTTTAGCGGGTGGGATTACTATATTGTTGATAGATCGTAATATAAATTGTTCGTTTTTTGACCCTTTGGGTGGGGGTGATCCTGTGTTGTACCAACACCTATTCTGGTTTTTTGGCCATCCTGAGGTTTATATTCTCATTTTACCTGGTTTTGGATTGATTTCACATATAATCATGGAGGAGAGAGGGAAAAAAGAGGTTTTTGGAACGTTGGGAATAATTTATGCTATGGTCGCTATTGGATTATTGGGGTTCATTGTATGAGCACACCGTATGTTTACAGTAGGGATGGATGTAGACAGTCGGGCATATTTTACCAGAGCAACTATGGTTATTGCCATTCCCACAGGAGTAAAAGTGTTTAGCTGGTTGGCAACTCTCTTTGGAGGTAAGCTTGTTATGTCAGTAACACTATTGTGATGTTTAGGGTTTATCTTTTTATTTACTGTTGGTGGTTTAACAGGTTTAGTTTTAGCTAATTCTTCAGTTGATGTAGTATTACACGATACCTACTATGTAGTAGCACACTTTCATTACGTGTTATCTATGGGGGCTGTATTTGCAATAATTGGAGCATTTAATCATTGATTTCCCATCATTAGCGGTGTAAGGTTAAATCAGAAGTTGATGAAAGTTCACTTTTGGATAACGTTTATTGGTGTAAACATGACGTTTTTCCCACAACACTTCTTAGGTTTGAGAGGCATGCCACGTCGATATGTGGATTATCCTGATGTTTTTCTATGTTGAAACATATTATCGTCAATTGGTAGACTAGTTAGAGCTGTGGGAGTGTTGTTATTAGTATATGCAATTTATGAAAGGCTAGTGAGAAAACGGGTTGTTGTGTACAGTCTATCT---GGGAATTCTTTAGATGCTATATTTGGCTGTCCACCTAATGCTCACACACATGAGAGGGTGCCTTTGGTCTTTAACATAGGATTCCAGGATAGTTCCTCTCCAATGATGGGTTATATTACTGGAGTGCATGATTGGATTATAATTGTGGTATTAGTTGTTGTTTCAATTGTTATATATGTGTTAGGAGGAATAATACTAACAAAGGGGTGG---------GACCGTTTTCTTGTAAGTGCAGAGACTTTGGAGTTTATTTGAGCGGGACTACCAGCTATTTCTTTGGGGTTGTTGGCTATCCCTTCCCTTCATTGTCTCTACCTAATAGAGGAAGCTTATTCTCCTTTTTTAAGGTTTAAAGTTGTAGGCCATCAATGATACTGGTCATATGAATATTCTGACTATTCAAATTTGGAG---------TTTGATTCATACATGCTAAGTCAAGATTCCTTG---------------TTTCGCCTCTTGGAAGTAGATAATGCAGTTGCTATTCCAATGGATTGTGAAGTACGCGTTTTAGTTACATCTGGAGATGTAATTCACTCTTGGACAGTACCTTCAATGGGTGTAAAGAGGGACGCTATTCCAGGTCGGTTAAACCAGCTAGTACTAATTGGATCAAAATTGGGATCTTATTACGGCCAGTGTTCAGAAATGTGTGGGGCTAATCATAGTTTTATGCCGATTAAGGTTGATGTTCTTACGAAAGATTTATTTATGAATTGATTATTAAAGGGAGGATTCCATCCATTTCATCTAGTGAGACCTAGACCATGACCTCTATTGTTGAGAGTTTCCACTTTTTCCCTAATAGTTGGATTTTATGTATGAATGTCG---AGTATAGGGAGGGTGTTGATAGTATTAGGTGTGTTTAGAGTTATGTTGAGGTTGTTTTGTTGGTTGCGAGATGTGATTCGTGAAAGGACCTACCAAGGGTGTCATACAATACGGGTCATGAAAGGTTTACGTTTGGGTATTGTTATATTTATTATCTCAGAGGTAATGTTCTTCTTTTCCATTTTCTTTGGAGTGTTTTTTTTATCTTTAAACCCTGACGTAGTTTTAGGAAGATCCTACCCACCCGTAGGTATTCAACCTTTAAATTATATAGGAGTACCATTTTTGAATACTATGATTTTACTGTCGAGAGGTGTAACTGTAACTTGATGTCATCATGGAATTATG------AGAGGTAATAAACACCATAGAGTGTTGGGTTTGACTATTACAGTGGTGTTGGGAGTGTTGTTTGTTATGTTTCAGTTTGAGGAATACTACGAAAGTTCATATACTATTGCCGATAGTGTATGTGGTTCATTGTTTTACATGTCGACTGGTTTTCATGGAATTCATGTTATGTTAGGTACTGTAATATTAATTGTAAGTTTAGTCCGATTAACAATAAACCATTTTAGAAGAATTCATAATTTAGGTTTTGAGATATCGGCGTGATATTGACATTTTGTGAGTGTTGTT---------TGTTATGTTTCAGTT------TGATACTTGTGAAACTACGGTTCCTTGTTATTGATTTGTTTAGTAATACAAATTGTTAGTGGTATTTTTCTATCTATGCACTATGAGGCATCAATACTAAATGCATTCTCCAGAGTAGTATCAATGGTAAATGATGTAAACTGAGGTTGACTTATTCGAATGGTTCACGCTAATGGGGCATCATTTTTCTTTATTGCTATTTATATTCATATCGGACGAGGTTTATACTATGGGAGATACCGAATAGTA---GGAGTCTGGTTAGTAGGGGTGGTATTGTTGTTTTTACTTATGGCAACAGCATTCTTAGGTTATGTTTTACCTTGAGGTCAAATGTCATATTGAGGAGCAACTGTAATTACAAATTTGCTATCTGCAATTCCTTATTTTGGTGAGGCTATAACAGGATGGTTGTGGGGAGGTTTTTCTGTTGGTAATCCAACTTTGGTACGGTTCTTTTCATTTCATTTTGTGTTGCCTTTTGTTATTTGTTTGTTTGTGTTATTTCACTTGGTTTTCCTTCACTGGTTTGGAAGTTCAAACCCTTTAGGGTTATCCAACAAATCAGATATGATTTATTTTCACCCATATTTTAGTGTAAAAGATGTTCTGGGATTAGTTGTGGCTTTGTTTATTGTTTGTGTTGTAGTTCTTCTTTTTCCTGATTTGTTTATAGACCCTGATAATTTTATTGAAGCAAATCCGATAAACACACCCCCTCATATCCAACCTGAATGATATTTTCTGTTTGCATATTCTATCCTTCGTTCGATCCCTAACAAATTAGGTGGAGTTGTGTCTTTGTTGGCAAGGGTGATAATTTTAGCATTACTTCCACTTTAT------GGAAAAGGGTTTAGATTTCGCTTTATAGGACTAAAGAAGATTTTATATTGATTTCACGTGATGGTATTTTTTATATTGACAGTGTTAGGTTCAATGCCTGTAGAATATCCATATACTGCTATTAGCCAAGTGATGACTTTAATTTATTTTGTGAATTTTATATTCCTAGTACAAATGTTATTTACTATTATATCAATTTTTATTGCTGTAGCTTTTTTCTCTCTTTTAGAACGGAAGATATTGAGGATCTCTCAAAACCGAGAAGGTCCAAATAAGGTTGTTCTGAAGGGGTTTTCTCAACCAATTGGTGATGCAGTTAAACTTTTATCGAAATCTACTAGTTTGCCA---AACTTAGGCTTCTATTCGGTATACACTTTAGGTCCTCTAGCTCTGCTTAGAATTAACACCTTCTTATGGATTACAACTCCA------TTTCTTTCCAAGTTTATCCACTTAAACCATTCCGGCATAGTTATACTTCTAATCCTAAGTGTTACAGCCCTACCTACCATTTATAGAGGTTGATTCTCTAATTCAACATTTAGAACTATGGGGGCAATTCGCTCTGTTGCTCAATCTCTCTCATTCGAGATTACATTTAGATTCAGACTATTTATTAGATTCCTTATAATTCAATCATTATGTTTGGAAAACCTTCCTAAGTTTCAAAGATGAAGCTGA---CTGTTTTGATGTATTCCGTGAATTTCTCTTGTAACGTTGATTTGTTTTTTAGCAGAAAGAGGACGTAGACCTTTTGACCTTCCAGAAGGTGAGAGGGAGTTGGTAAGTGGTTACACTATTGAATTTGGTGGATTGCATTATACATTAATTTTTCTTGGAGAGAATCTTGCTGTAATATTTATAACCATAATTTTCTCCACCACCTATCTTGGT---GGGTTTTCTTTGTGAAAAGCAAGTACACTAGTATTAATTATT------------GTTATAATTCGGAGGTCTTACCCCCGTATTCGATATGACCAACTTATACAGTTAAATTGAGTCGGGATCTTACCTCAACTTATCTCTTCAGTTTGATTGGTGCTTGTGTTATCTATTATGTTATTCTTATTA------------------------GTACTGTCTGTGTTAATTTCTTCTATATCAGAGACAAGTTTTGATACAAATGAGTCTTTTGAGTGTGGATTTTTTACAGGAAGGGATATTCACTTACCGTTTTGTGTTCATTTCTTTATTGTAGGTATCCTTTTTGTAGTATTTGACATGGAATTGGTAATTTCACTACCACTAATTGTGGCA------AATCTGAGAGAACCAGTATGGTTATTATGA---TGGTTAGTATACAGCATTATTTTGTTTATTGGAATTCTATTAGAAGTAACATGTGGTTCAATTGATTGGGGTATAATAATTGCTCTATTAAGAGTTGAGTTTCTATCTGTCAGCCAGTTTTATGCTGTCCTTTTTCTTGTTAATCCAAGATCGTTAAATTTTAATAGATGTTTGGTGTTACTGTCAATTCTAGTGTTGGAGGGAAGCTTAGGTTTGACCATTCTTGTTTCGACAAGTCTAAAAATTGATTCTACAATAATTTGAGATGTTGGTTGTGTAATGGTTGTAGATGGTTTAAGGTCGTTATTTTTGTTTACAGTTTTTCTTGTAAGATTTATAGTTTTCAATTATAGAGTGCATTATTTTTCTTATTTAGAAGGGTTTAATAAGTTTATTATTACGCTGTTTTTGTTTGTCATATCAATATGTATTCTTTGTTTAAGACCAAGAATGTTTTGAGTGATAGTTGGTTGGGATGGTTTAGGATTGACTTCATTTGGCTTAATTATTTTCTATCAAAATTGAAGGAGGTTTAGGAGGGGTTTGTTTACATTTTTAATAAATCGTATAGGCGACATGTTTATGATTTCGGTCATTGTGATACTGTCAAGA------------TGCAACATGTTGAAAAGAGTGTCAATAAGG---------------TCTGCTGTTCAAATTTCTGCACTCTTACTGTTAGGTGCAATAACAAAAAGAGCTCAATTGCCCTTTTCTTCATGACTTCCACTAGCAATGGCTGCCCCTACTCCGGTGAGGAGGTTAGTTCACTCGTCAACTTTAGTAACTGCTGGAATCTATTTGCTAATTCGCTTTGAAAGT---TTATTCCCATCGGAGGTGTTACAAGCA------CTTAAGGTAGTGTCAATCATGACGATTGTTTATGCGGGAGTATCAGCACTTTGTGAAGTGGATTTAAAAAAGGTAGTAGCTCTTTCTACACTAACCCATTTAGGAATTATAACACTATATGTATCTATCGGGAGTGTGACTGCTGCTACAACTCATTTAGTTTTCCATGCATTCTTTAAGAGAGCATTATTCATACTATCTTCAATAGCAGGTCTGCCTTTTTTGACTGGATTTTACTCGAAAGAAGTA---------------ATGGTGATGTTAGCAGAAGATTCTTTGCTTACACTGGTTTCATTTTTGTTAGGGGTAATGTTAACTTCTGGTTATTCAGTTCGATTAATAGTACTAATTTTTAAATCA---------------------CCCAATTTTATTTCTGATAAAAAGGATTTTACCCAAGTAAGAGAAGGTTTGGTTAGAGCAAGGGTGCACGGGTTTATGATTAGAGTACTTGGCGGTAGAATGTTACTGTGG------GTTGTAATACCTATTACGATGGTTATG------GGTTACAATTTAAGATGTAGGGTTTTAAATAAGCTATTGATTATTGTGTCGCTTGCAGGAGGACTTATTGCAGCATTATGTTCCTTA---TACTTGTGGATAGCTTCA---AGGTGTAGAATGGTAGTTCTGTCTTTATCTGTGCAAATTGTTATATACAGGGTAATGTTGGCAATGAGGGTTACTCACTATTGAATTGTAATTTTAAGGATTATAGGCTTGTTGGGAGGTTTGATTGTCTTTCTGTCATTTATCCTTATAATACTTCCT---AATCCAGATTTAGGTGTTTTTAATAAATGAAAT------------------------------TATGTAGGATTATCAATACTTATATTATTAATACTGACTAGTATTTAC------------------------------------------------------------CTACCAGTAGTAGATAAGAATGAGCCT------------------GAACTGTTGCCAATT---TCACTACATTTATATGCTTACGGCCTATGTTTCTTTTTATTGCTTTTTGTGTTACTAATTATAGTAGGTATTATAAATTATGGTTATAAATCTATCTATCTAGCGGTCATACACTA-----AGCTCAAGTTAACTTTTATACTGAT--------------------TATACTAGTAACATGAAATAGTAGCTAAATTTAA----GTAGGAAGGTGAAATAAAGTAAGAAATTGTA----------CCTATAAACTAGGATTAGATACCCTACTATA-TACAACGTA----AGTATCATATCAAGGTAGTAATGGTTAC----GGTCCAT-------GAAAACTAATAACATATGGCGGTTGAC----AATCTTTCCAGAGACCCATGTTTG--TAA-TCGATAATGCACGTC-AATCTTACCCTAT----------------------TTAA--------GATTTTGTACACCGCTGT-------CGGAAGGTT-----GTGGATAATTT------------------------------CATAATCTATT------------------ACTATCAAAGTAATAAGTCAAGTCAAGGTGCAGACTACGGTGG-GGGTATAATGTGGGTCA-------------------------------TTGGTCTAATATGAACAGACATGT----TATTGAAAAGAATTTGGTAGTAA--ACTTAAAAATTATATTGAGTTGAAATAGCA--ATTGTCAATGTACAAATCGCCCGTCACTCCGTTGGATAAGTCGTAACAAA-GTTGATCTATTGGAAAATGGTACCTTTTGTATCAGGGACTTAGGAATGAGTAACAGTTTATA--CGAA------TATCCCGAAAA--GAAAAGAATCCTCTAT---ATTAAAGATTATTGTTTAATTAATATCTTCAAATTAT-AAAGAGTGG-TGAAATGTCATACGATTTTCTAGTT-ATCTGGTAACTCGGCAAATAATT---AGTCTGACTGTTTACTAAAAACATTGCCTTTAGAAG--T-AAATTGAAGGTAAGACCTGCTCACTGCGTT-GATAACGTAAATAGCTGCAGTATTC-TGACTGTGCTAAGGTAGCATAATAACTTGTTTCTTAATTGGAATCTAGAATGAACGGTTCTACAAGACTAAGTCTGTCTCTGTGAAGTAAAATT---GAATTTTAATTTTAAGTGAAAATGCTTAAATACGTTAGAAGGACGAGAAGACCCTATAGATTTTTTTTACCTGGGAAGGGGTCTACACA-CATTAAGCTATGTTTAATAAAACTAAAATAATAGGAAATTTAATGATGATCCTCAAGTTGTGAAAAAA---AGATAAATTACCTTAGGGATAACAGCA--CAATTGTCTTTCCTA-GATCTAATTGGATGATACAGATTGTGACCTCGATGTTGAATTAAG-TTATCATTTAGATGAAGAAGGTTAA--ATGGTTA-GTCTGTTCGACTATTAAAAACTTACATGATTTGAGTTTAGACCGACGTGAGTCAGGTCAGATTCTATCCTCTG-A-AATTGAGTTTACCCT-TTTAGTACGAAAGGAT

Pthirus_pubis ATGTCTATATTTGATCCTAGAATGATCTTAGTAACTGGG---TTACCTGTA------AAGTGGCTGGGTGCAACC---------------------TTAGTCTGTCTGTATGCGGGAGGCGAGTTTTGGCTTGTTAACTCTGGGTACCGGAGGGCTGTTACTAAAGTGTGCTTATTCCTTCATGAATTGCAT---------------------------------TTTATTCCGTTTTTGGGTGTTGTAAGATTTTATTGTGTATTGAGCATAAATGTTATCTCGTTAATTCCTTTTACTTTACCTTTTACCTCTCATATTTCTGTTAATTTGGGGATATGTTTGACTCTGTGGTTAAGTGGCTTACTTTACTCT---TTTGGAAGTTCGCTCAGGAAGTCTTTGGCTCATTATTTACCTTTGGGGAGTCCAATGGTTCTGGGTCCGTTCCTAGTTCTAATTGAGGTAGCCAGGGTTCTTATTCGGCCAATTAGGCTAAGAGTGCGGCTGATGGCTAACATTTTAGGTGGCCATATTATTATGAGTCTAATCGAGGAAAGGTCCTTTGGT---AGCTTGATTAGAGGGATTTTCACTTTACTAACATATTCTCTAATAGTTTGTGTAGAGATGTTTGTAGCTACTGTACAGGCTTATGTACTCAGAAAGCTCTTAAGAATCTACTGAGAGCTATCCCCCTTACTTTGGTTAAATATTTTTATTTTTATGGTGTTGGTGTTTATGGTTATGTTGTCT---GGATCTTATTTTTCTATGTCCACTGTTGAAGTTAAGTTTCGGTATGAAGCTCGG------GTTATTTTAACGTTGGAAAAGTGTATACTATTTTCTACTAATCACAAGGATATTGGACTTTTGTATCTGCTTTCTGGAATTTGATTTGGTTTAGTTGGCTTGTCAATGAGTCTAATTGTGCGTGTAGAACTTTCATCAACCAGGTCATGATTGGTTAATAGACATACTTATAACGTGTTTGTTACATCTCACGCTTTTGTAATGATTTTTTTTATGGTTATGCCTGTTATGATAGGAGGTTTTGCTAACTGGCTAGTTCCTTTATTTTTAGGTGCTCCTGACATGGCTTTCCCCCGGATGAACAATATAAGCTATTGGCTGATTATACCCTCTGGGGTTCTGTTAATTGCGAGTTCAATAATCCAAGGTGGAACAGGTACTGGCTGGACTATTTATCCTCCGCTAAGTCCTTTAGAAGGCCAACCTTCTTTATCAGTGGATTTTACCATCTTTAGCCTCCATTTAGCTGGAGTAAGTTCTATTTTAGGCTCAGTAAACTTTATTAGGACAATTTTAAATATATGACCTTCTAGGTTAAAGATTTACCGGTTACCTTTGTTCTGCTGGTCTGTCTTAATCACGGCCTTTTTGCTTCTACTCTCTTTACCTGTACTTGCGGGTGCTATCACTATGCTACTATTAGATCGGAATTTTAATTGTTCCTTTTTTGACCCTCTAGGTGGAGGTGATCCTGTTCTTTACCAGCATCTTTTTTGGTTTTTTGGTCATCCTGAGGTTTATATCTTGATCTTACCTGGGTTTGGATTGATCTCTCATATAGTTGTTGACTTGAGAGGTAAGAAAGAAGTCTTTGGTTCATTAGGAATAATCTACGCTATAGTATCGATTGGTGTTCTAGGGTTTGTTGTTTGAGCCCACCACATGTTCACTGTTGGTCTAGATGTAGATAGACGTGCTTACTTCACTAGAGCTACTATGACCATTGCTATTCCAACGGGAGTAAAAGTCTTTAGCTGATTAGGTACTCTATTTGGCCCTAAACTTCACTTAAGAGTGAGGTTGCAGTGGTCTCTGGGATTCATCTTTTTATTTACTGTCGGGGGCCTGACAGGGATTATTCTATCTAACTCGTCAGTTGACGTTCTTCTTCACGATACCTACTATGTTGTAGCTCATTTCCACTATGTATTGTCTATGGGTGCAGTGTTTGCTATCTTTGGTGCGTGGAACCATTGGTTTTCTTCTTTAAGAGGCTGCTACCTTAACCCTAAAGTTATATCCACTCATTTTTGAGTAAGGTTTATCGGGGTAAATTTAACCTTTTTCCCTCAGCATTTCCTCGGTCTCAGGGGGATACCTCGACGTTACTCAGATTACCCGGATGCTTTCTACGGGTGGAATAAAATCTCCTCACTAGGAAGTATACTAACTTTTGTAGGAGTGCTACTATTTCTCTATGCTTCGTTTGACAGAGTTGCTAGAGCAAATAAAGTACTGTTTGAGAAAGTTAGAGTACAGGATCTTGTGGGTCTTGTTGGTAAGCCGTCAGGGTTCCATACATTTACCACTAGATTACATCTGTCAGATTTTACTTTTCAGGATGGTAGATCTCCAGTTATAGCGTTTGTTTCTAATACTTACGACCTTGTTTGTGTTGTTTGTGTCGGTGTTATCGCTTTAGTGGCTTATGTCTCTCTATTCTTTCTTTTGAACAAGTCGTGA---------AATTATTACTTTGTAGGCCTTGAGAGGTTAGAAACTATCTGGGTAATTTTACCGTCTTTAGCGCTAGCCTCTTTGGTCTTACCTTCTTTGCACTGTTTATATTTGATGGATGAAATCTACACTCCTTCAGTTACCCTTAAGGTTATCGGCCACCAGTGGTATTGGTCCTATGAGTACGGAGACTGAGATAGAATTGAG---------TTTAATTCTTACATGTTAAGTGAAGGTGACTTAAATAACGTCGAGTCATTTCGTCTTTTAGAGTCTGATTGCAGGGTGTATATTCCTTCTAGAACAGAAATTCGTGCTATCATTACTTCTTCGGATGTCATTCATTCTTGGGCTATTCCTAGACTAAGGGTTAAGATAGATGCTGTCCCTGGACGGCTAAACCATTCTGTCATTTACTCTCATAAACTTGGGAGTCTGTATGGTCAATGCTCTGAGATGTGTGGGGCTTACCACAGGTTCATACCAATTTCTGTCAAGACTGTTCCTAAGGCGGTATTTATCGCATGAGTGAAGGAAAGTAAGTTTCACCCATTTCACCTGGTTGATTTTAGCCCTTGGCCTTTATATTTGAGTCTATCCTCTTTATGTTTTGCAGTGGCTGTTCTGTGTATTTTAGGTTCATTAAAAGGGTTT------TTAGCTTTTTCTGTAATTGTGTTTGTGCTTGTGCTTTCTTTGTGGTGACGTGATGTAATTCGTGAATCAACTTTTCAAGGTAAACATACTATCGAGGTTAAGCGGGGTATCCGTTTAGGAATAATTTTGTTTATTATGTCTGAGGTAATGTTCTTCTTCTCGTTTTTCTATGGTCTTTTCTTTCTAGCTCTTAACCCAGATGTAACTTTAGGTGGTCGGTTTCCTCCGGTGGGTCTAGCGTCAATAGGGCTACTTGGTGTGCCTCTGTTAAACTCGTTTTTACTCTTATCAAGTGGGGTCAGGGTAACTTGATCTCACTACGAGATTCTC------CGGGGTGATGTACCAAAGTCTTTACTGGCGTTGGCTATGACTCTAGCTCTAGGTACTGTGTTTTTAGCCTTTCAGTATCTGGAGTACAAACTTAGGAGGTTTAGGATCTCTGATAGTGCTTATGGGTCTATCTTTTACCTTATAACTGGCTTTCATGGGTTTCACGTTATGGTGGGAGTGGTATTCATTTTAGTAATAACAGTTCGTTTAGCTAAGGGTCATTTGAACTCAAGACATCATACAGGGTTTGAGCTATCAGCCTGATACTGACATTTCGTGGATGTAGTATGGTTACTCTTATTTGTGTCACTTTACTGATGGTATTCTTGAAATTTTGGTTCTCTTTTGGGTTGTTTTTTAGTTGTTCAAGTGTTTACTGGATTTTTGTTGTCTACTCATTATGAGGCTTCTTTGTCT---TCATTTGAAAGGGTACTCTTGATCGAGTTTGATGCTTTAGAAGGGTGATTAGTTCGTAGCTTACATGCTAACGGAGCTTCTTGATTTTTTATTCTGGCTTATTTCCATATTTGACGCAGTCTCTGGTTTGGTTGTTTCTCTCAAAAG---CTCGTTTGAGTGTCAGGTATCCTAATTTTATTGCTTATAATGGCTATCTCTTTTTTGGGTTATGTCCTTCCTTGGGGGCAGATGTCGTTTTGAGGGGCTACTGTTATCACAAATCTTTTCAGGGCTCTACCTTTTGTAGGCTCAGAGTTGGTTACCTGGATTTGAGGTGGATTCTCAGTGGGGTCGCCCACGCTAGAGCGATTTTTTAGTTCCCATTTTATGCTAAGAATGGTTTTGCTGTGCTTTGTTATTTTCCACATTACTTTTCTTCATGAGAATGGCTCTAGAAACCCTTTAGGGTTGGATCTACAATCAGACAAGGTGTATTTTTATCCTTACTTTATGCTTAAGGATTTGCTTGGAGGCTTAGTTGCGATGACTATCTACTTCTCTCTAGGGCTTTATTCGCCTGACCTATTTATGGATCCTGATAATTTTATAGAGGCTAACCCTTTAGTGACTCCTCCGCATATTCAGCCAGAGTGATATTTCCTCTTTGCTTATGCCATTTTACGGGCTGTGCCTAGAAAGCTTGGCGGGGTAGTTGCTTTAGTAATGAGCATTGTATCTTTGGTAGTTTTACTGCTA---------GGTAAAAGTTATTCAGCACGGTTTAGGGTAGCACGGAAAGTACTAGTTTACTCTCTGGTAGTTAGAGTGCTTATTCTCTCTTGACTAGGAGCTATACCTGCTGAAGTGCCATTTGTAAAGGTTAGACAAGTTGCAAGGGTAATTTACTTTACTCTAATCTTACTAATTTTGCAAGGGTTTATAACAGTTGTAATATTGATGGTTTGTGTAGCTTATTTCTCCTTATTCGAGCGAAAGATACTAGGTCTAACTCAGCTCCGGCTAGGACCAAATAAAGTAGGCCCTACAGGTGTTCTTCAACCATTAAGTGATGCTCCTAAGCTTCTAACTAAGTCTATTCAAGCTCAT---ATGGAC------CCTTTTATTTACGCATTTGTTACTTTTCTCTCTTTTTTACTTTCTTTAACTTATTGATTTAGTATTCCGATTAAGTTTACGCCATGGAGGTGTGAAAGAGATAACATGGGTATCTGGATTATTATCGGCTTGAGCCTGGTTGTTTATGGTCCTGTTTCTTGTGGTTGATTCTCGCACTCTAAGTTTTCAGTCTTAGGTAGAACCCGTGCTATCGCTCAGTGTATTTCGTTTGAACTTGTCCTTTCAGTTATCTTACTTTCGGTATTTAGGTTTTCATCCTGTACTTCCGTTCTAGGAATTTGTATAGCACAGCTTTATGCTCCTAACTTACTGACAGCCCCG---CTACTAGCTTTCGGGGTCTGAATTTCTCTTCTTGCTGAAAGAGGTCGTAGCCCATTTGATTTACCTGAAGGTGAAAGGGAATTGGTAAGAGGGTTTAATGTGGAGTACGGAGGTGTCCGGTACATTTTAATTTATCTGAGAGAAAGTGTGACTCTTATGGTATCTTGTATGATTATGAACAGTTTATTTTTAGGA---GTAACCTACTTGCTACCTATACTAATTTGAGTGGTTATGAGT------------ATCCTTCTACGATCTAGATTACCTCGGATGCGGTTTGACCATTGTATAGCGCTAGGGTGAGTAACTTTATTATCTTCTGCTATCTCTTATGCTTTATTAGTTGTGGTTGTTGCCCTAGTGGCTGTGTTTGCG------------------------TTTTTATCAGTTACTCTCTCTTCACAGGAGGTAGAA---GGATACGACCATTCCCCTTTTGAGTGCGGTGTTATGCCTTATTCTACTTCGTACTCACCATTTTATGTCCAATTTTACTGCATAAGAGTTATTTTTTTGGTATTTGATGTAGAAATTGTTATCTTATTGCCTATAGTTGAGGCT------TCTTTACCTGAAGTTAGAGGAGTTGTATTT---TGGTTGCTTACGTTCCTACTTTTATTTATGGGATTGTTAATTGAAATCGGCTACGGGTCTTTAGAGTGGAAGTTTATGTCTTCTTTAATTTCTATTGAAAGATGTTGATGTTGGTCGTACTTTGCTCTTATGTTCACTGTTGGTCTA---GATGTAGATAGGCTAATTATTTCAGAGATTTTATCTGTTATGGCTTGTGAAAGAGTAATTGGGCTAACTCTCAGCTCTAACCTCTCAAGCAGTAGTTGTTACTCTTTTCTTCTAGATTTAGATATAGTATTTATTTTTGATCGGGTATCTGTCATGTTTTTAATGATGGTGTTAGTCGTTAGGGCAGTGGTGATAGTTTTTGGTAGATTCTATATGACTTCTTCTTTACATGGTGTTGCTTTTACATTTAGGATATTGATTTTCATCGCCAGGATGGTTATTTTATCTGTCACAGGATCTTTGTTTTGGTTATTTTTAGGGTGAGACGGTTTGGGCTTATCTAGGTTCATTTTAATTATTTTTAACAAGAATTGATCTTCCTCTAAATCAGGGTTAATTACTTTTTTAATAAACCGGTTAGGGGATGTGTTGATAATCGTAGCCTCTTCGTGGTTACTTATTCAAGGCTCT---------------TTCGGTTCAGGGCTTTCT---------------------GCTCTTGGAAGAGTTGTATTCACTATCGGTTGTTTGTCAAAGAGAGCTCAGTTTCCTCTTTTAAGATGGTTACCCGAGGCTATAGCAGCTCCTACCCCTGTAAGAAGGCTGGTTCACTCTTCAACTCTGGTTACTGCAGGCATTTATACACTAGTTCGGTTTGGTAGC---GAAGTCAGAAATTGGGGTTCT------------ATCGTTGCATTTTCTTTTTTTTCGTTAGTTGTATCAGGGCTTTCTGCTCTTGTTTCAACGGACTTAAAGAAGGTAGTAGCATATTCTACCCTCTCTCACATTAGCCTTATAATTCACTATCTCTCAGAGGGCTGTATTGACGCTTGTATACTTCATATAGTTATGCACGCAATCTTTAAGAGTCTTTTGGTTTCTTTAATTTCGATAGCAGGGCTACCTTATTTAAGAGGTGGATTCTCTAAAGAGGTT---GTT---------TTAATACATAGCCTTAAGTTCGGGTTGTTTAGAGCAAGGTTCTTTCTGGTTTCTGTATTCTTAACTAGGTCTTATTCTGTGCGAATTATGTGGATCTTATTTAGCCCC---------------------TCTAACTTTGGGTTAAAG---------TTAGGCTTAAACACAGCCCTATTCTCATTCTCTTTGAGTCTAGGTGTGTACCTCAATATGATGGTGGTATTCTGAGCAGTCTGA---------ACTCCTTTTTGAATGGGGTTTATA------GCCCATGAGTCGAGGGTCTCTGTACTAAGAAAGACTCTAATGATTAGAGTGGCGTCACTAGGATTATTGAGATTAGTGTGTTTAGTT---TACATGAGGTTGACAAAA---AGGTTAGTTATTATGTTATTGGATATAGCGTTTATGGGGGTGATTATTAAAGTTCTTCTCTCGTTGTATGTTACTTCTAACTGGCCTTGACTTTTACTCCTGCTGGGTGTCGTCGGAGGTTTAATCGTAGTGATTTCTGTTATTCTGGTTGTTATGCCT------------GGGCCTAGCGCCTTACACTGGAGGGTTGATAAA---------------------GGGTCTACAGTGACTTTTGTGCTTGTGCTAATTTTGGCTGTGGCTATT------------------------AGCTAC---------TTAGACGAGTTAGATAGCTTTTTTAGCCTGACT---TATAGGACATGC------------------CGTTTAAATATACTG---TCAAACTCTTCATTCTTGTACCCTGAGGGGGTGTTAGTACTGCTAATTGGGCTACTTTTAATACTTCCTGTCATAGAGGTCTTGCTTTCGTTTTATGGGCGTGCGGTCATACATTA----GTCATCAAGCTAAATATTTATTC-----------------------TAGACAGGTGTGATTAGTGGTTACGTGACTTTAGTTGCTTTTAATCGTGAAATTCACTTATAAGCTTAA--AAACACGGCTAAAAGACTAGGATTAGATACCCTATTATGAGTGTTTATGAAATGTGTCTAAGTCAGTA-----------------GCCTTTCCTTAGGCAAAATCTATTTGCTA-TGGCGGCTATT----AACCCTATCGGAGTCGTATGGACG--TAA-ACGAAGCTGCACGAG-CATTTTACCTTAACTTTACCGGTAGACAAGGTTGGTCTGGTA-----AGTCTTACACACCGCTGT--CAACTAAGGAACTT-----TTACAAGACTTTAGTCT------------------------AAGGTAAGTGA------------------TGGACTGGCCTGTGTGGACAAGTCAAGGTGTAGA--ATGTCAA-GGTGCTTATGTACGATTTAATTGA-----CTGAGTAAGTTGACATTAATTGTACTATGCAATCGAA-----------GCTAATAAAAATTCGACAGTAAGGTGACTTAGAGCATATTCATCTGAACTAGGTA-ACAAATAGTGTACAAATCGCCCGTCATTCCTACGGACAAGTCGTAACAAA-GTTGGTTTACCGGAAGGTGGTACCTTTAGCATCATGGGCTTAGGATTAATTAATTCATGCTTTAGCGGAGTATGTTTCCCGAAAGGAAGCGAGGATCTTGG--TTTGGTTCCTTCTCTTGTGACATTACGGCTAAGTAATCAAATCACGGAA--TGAAATGTGAGTCGGTTCTTCTGATTATCTGGTAATTAGGCAACCTAAAG--GGTTCGAATGTCTAGTAAAAACATTTCCTAAAGAAAATA---ATTTTAGGTATGACCTGCTCAGTGC--TGTAGATAGTAAATAGCTGCAGTAACCGTAACTGTAAAAATGTAGCATAATCAATTGTCTCTTAATTGGAGTCTAGGATGAACGGTCTAACGTAACCCTGTCTGTCTCTCTTTAATTACATG---AAGTT-AAATTCTAAGTTAAAATGCTTAGATGTAAAGATTGGACGAAAAGACCCTAAAGATTTTCGTTACCTGGGATGAG-GT--------AGTCAGTTCTAAGTAACGACTTTCAATATACAGCCAACACGGCTATGATGATCCTATTGAGATTAAATGAGTTTAA-TTACCTTAGGGCTAACAGCG--TGATAACTATTCTCA-GAACTTATGGGA-ATGGTGGTTTACGACCTCGATGTTGAATTGGGT-ACACTTTTCATAGAAGAATATGGA--AGAGTGA-GACTGTTCGTTTCTTAATTACCCACATGATTTGAGTTTAGACCGACGTGAGTCAGGTCAGATTCTATCCATCT-TCTTAAG-A---GACTT-TTCAGTACGAAAGGAC

Polyplax_asiatica ATGTCAATTTTTGACCCAAGGCTTGGGGTTGAGGGG------CTTTCGGTA------AAATGGATTTGAGGGTTA---------------------GCCCCTTTCTTGCTTTTGAGCGGAGGCTTTTGGGCCTTGTTGTCCGGCGTGACAGGAGTTCTTTCTATGTTTGGAAAGCAAGTATCAAATTCAGTTCTACCTTCGCTGAAGGAAGGTAAG---------CCTTTAGCTTTGAGTCTAATGGGGGTTTACATGGTGCTCCTAACAATGAATTTATTAAGATTGAGCCCATTCTCTTTTTGCCCCCCTTCCCATTTAAGCTTTGGGCTTAGGGTGTGCTTTCCCCTTTGGGTTGGGGGTTTAATTTCTTCT---TACAAGAAAAGTAAGGATAAATTTCTATCTCACTTTCTTCCACTAGGAACGCCTCAAGGTTTAAGGTGATTTTTAGTGCTAATCGAGTTAACTAGCCAAGCCGTTCGGCCATTGAGGCTGAGGGTTCGGCTAATGGCCAACCTAACGGCTGGACATATAATCATGAGTCTTGCCGAGAAAGCCTCTTTACTATTTCCATTATTATTTAAACTCCAGTTCTTGATAATTCTATCACTTTTACTTGTGTTTGAGTTTGGAGTGGCTTTAATTCAGGCTTATGTGTTTATAAGCCTGCTCTCTCTTTACTGAGATATAGCTCCAACTTATTGAGTTATTTTACTTTTATTAATTCAAACTATCTTCCTAATAGGGAAAATG---GGTTTATATTTTTTATTAGTAAGCAGGCTTGCTGAGGGGCAAGGAGTTAAGTCTCATAAGTCTTTGGTTAATCAGGATTTTTTGCTCTTCTACTCTACAAATCATAAAGATATTGGAGTATTATATTTAGTATTTAGGGCGTGAGCAGGTTTGCTGGGCTATGGGCTTAGCTTCTTGATTCGCTTAGAACTTTGTCATCCTGGGAATTTAATGGAAGACGGGCATGTCTTCAATAGTTTAGTGACTGCTCATGCTTTTGTGATAATTTTCTTTATAGTAATACCGGCTATGGTAGGGGGGTTTGGCAACTGGCTAATTCCCTTGATAATTGGGTTTCCGGATATGGCCTTTCCCCGAATAAATAATATGAGTTTTTGGCTTCTACCCCCTTCTCTTTTTCTTTTGCTTAGAAGAGCTTTAATCCAGGGAGGAGTGGGGACTGGGTGAACGGTTTACCCTCCTCTTAGAAGGAGGGTTGGTCAGCCCGGAGCTTCAGTGGATTTGGCTATTTTTAGCCTTCACCTCGCGGGGGTGAGGTCAATCCTTGGAGCAATTAATTTTATTTCAACAATTATAGTTGGGTGGAAATTTTCC---CAAATTGATAAGCTTCCATTATTTTGTTGGGCAATATTGATTACAGCGTTTCTCTTGTTACTTTCGCTCCCAGTTTTAGCCGGGGCTATTACTATACTTTTATTTGATCGAAACTTAAACTGTTCATTCTTTGACCCTTCCGGGGGAGGGGATCCAGTTTTGTACCAACATTTGTTTTGATTTTTTGGTCACCCTGAGGTTTATATTTTAATTTTACCTGGGTTTGGACTTATTTCTCACATAATTTCCGATGAAATGGGGAAGAAGGAGGTTTTTGGGAGTTTAGGGATAATTTATGCCATGAGGGCAATCGGGGCTATAGGATTTGTTGTTTGGGCCCATCACATGTTTACTATTGGGATAGATGTAGATAGTCGGGCTTATTTTACAAGGGCCACTATAATTATTGCAATTCCAACGGGAATTAAAGTATTTAGGTGGCTAGCCACCATTATTGGCGGGAAAGCCCCCCGGAGCGTTTCAATGCTTTGGGTAGCCGGGTTTATCTTTCTTTTTACAGTTGGGGGCCTAACAGGTTTGGTTTTAGCTAACTCTTCAATTGATGTAGCCCTCCACGACACTTATTATGTTGTTGCTCACTTTCATTATGTTCTTTCCATGGGGGCAGTCTTTGCCATCTTTGGCAGATTTAACCACTGATTTCCCCTTCTCAGAGGGGTTGTCTTGCCCCAGAAATGAATAAAGGCTCACTTTTGAGCTACCTTTCTTGGAGTAAATTTGACTTTCTTCCCTCAACATTTCTTAGGACTAAGGGGGATGCCCCGTCGATATAGGGATTATCCGGATTCTTTTAGGTGCTGAAACACTCTTTCCTCAGCTGGAAGGTCAATTACCTCAGTCAGGATATTAGTGTTCATTGCCTGCCTTTGAGAGGGCCACCTCTCCTGCCGTGTCTCCCTTTGACACGGAGCC---TGGGCTAGGCTGGATTGATTTAGGGGGTTCCCTCCCTCGGAGCACTCACACGAAAGACCCCCTCAGATTTTCCTAGTTAGCCTTCAAGACTCTAATTCTCCTTTGATGGGACATATTGAGAGGGTACATGATTGACTAATAGTTGTACTTGCTGGTATCATTTCCGTAGTCATTTATGTAAGAATTTGGACCTTTTTGAGAAAGGAATGA---------AACGTTTTCTTTTTTGACAGGGAGTGGTTGGAAGTCATTTGGATTATGTTCCCGTCTATTGTTCTTCTGACTTTAGCCTTTCCATCCCTTCAATGTCTTTATTTGCTAGAGGAGGTAAGACTTCCCAAGTCAACTATTAAGGCAGTAGGGCACCAGTGGTACTGGAGGTATGAATTGGTCTCTCCTACGAGCCTAGAGACCTTCTTGTTTGACTCGTACCTTTTGCCT---AAGGAGTGGGAGACTGGGGCTGCACCTCGGCTCTTGGACTGTGACTCCTCAATTCTTCTTCCAGTAGGAGAAGAGACTCGCTTAGTAGTAAGAAGAGGAGATGTAATTCACTCTTGGGCACTTCCAAGAATAGGAGTAAAGGTGGATGCAATCCCGGGTCGCTTAAACCAAGTTATTCTTTACCCATTAAAAAGGGGAATTTCATTTGGCCAGTGCTCCGAGATTTGCGGGGCAAATCACAGATTTATGCCAATTAAAGTTGAAGCCATTCCTCGAGAGGAATGGATTTCCATTCTCAAATTGAGGAAGTTTCATCCTTTTCATATTGTCAGAGTAAGGCCTTGACCTTTACTCTCTAGGGCTTCGGGGTTTACGCTGGCTGTTGGCTTAGTTGAGGCATTCTCG---GGAATAGGTTGATTCTTGGTAGGCTGGGGAAGCCTAAGTAGAGGGGCTTTAGCTGGCCTATGGTGGCGGGATGCCATTCGGGAGTCCTTCTTGCTCGGCGAGCACACGATGGAAGTGACTCGAGGCTTACGAGTCGGAGTAATTCTCTTCATTCTATCGGAAGTAATGTTCTTTTTTTCCATTTTTTTTGCATTCTTCTTTTTATCTTTGAATCCCGATGTTAGACTGGGGGGCCAGTGGCCTCCGGAGGGGCTCTCTCCAGTGCCGTACATGGGAGTTCCATTAATAAACACTGTCCTTCTATTATCTAGAGGGATTTCTTTAACATGGTCCCACCATGCCCTAATT------GGGGGCATGGCCTTTCGCTCTGCTTTTCCCCTGCTGATTAGGGTTCTTTTAGGAGGCGGGTTTTTACTTCTTCAGGCGGAAGAATATAGGGAGTGCTCCTTTTCTATCTCTGATAGAAGATTTGGCTCTTTATTCTTTGTAAGAACGGGGTTTCATGGGGTTCATGTGATAATTGGGACAGTTTTTCTGTCTGTCAATCTTGTTCGAGTTATTTCTGCCCATTTCTCCCCCCACCATCACCTGGGGTTTGAGGCGGGGGCCTGGTACTGACATTTTGTAGATGTTGTTTGACTATTCTTGTTTATTACCATTTACTGATGGTTTATGTGGAATTATGGGTCTCTGCTGGGCTTGTGCTTGGGGTTACAACTTGTAACTGGGTTATTTCTCGCTATACAGTTTTCAGCTGCCCAAGGTCTCTCTTTTGAGAGAGTTTTAAGAATTATAAATGATGTAAAAGGGGGATGGGCAATCCGGCTTCTTCATGCCAATGGGGCCTCCTTGTTTTTTATTCTTATCTACCTCCACCTAGGCCGGGGCCTATATTATGGAAGGTATCGGCTGATA---GGGGTTTGGATGGTGGGGGTTATAATTGTCTTTACCTTAATGGGTACTGCATTTTTAGGGTATGTTCTGCCCTGAGGACAGATGTCCTTTTGGGGCGCCACTGTAATTACAAATTTAGTGTCAGCTATTCCCTACTTAGGAGAATCTCTCGTAACCTGGATTTGAGGGGGGTTTAGGGTTGGAAACCCTACTTTAACCCGGATATTTTCACTCCATTTTATCCTTCCATTCATAATTGTGTTTCTGGCTCTCTCTCACCTTTTCCTTCTTCATGAGAGCGGAAGCTGGAACCCCCTGGGGTTAAGGGAGGATTCCGACAAAGTGGCCTTTCACCCTTATTTCATCTCTAAAGATTTCTTAGGGGTGTCCGTTTTAGGGAGGTTAACTTTGGCTTCCCTATTCCTCTTTCCTGATTTGTTTATAGATCCAGATAATTTTACCCCTGCTAACCCCTTAGCCACTCCAGCCCATATTAAGCCCGAGTGGTACTTTCTTTTTGCTTACTCAATCCTTCGTTCAATTCCTAGGAAGCTTGGAGGGGTGGCCGCCTTAGTTTTAAGGATTTTAGTCTTAGCCTTTCTTCCATTA---------ACGAAATCCTCTAGCGGCCGGTTTTCCTTCCTTCACTCCTTTACCTTTTGATTTCAGTTAAATAATTTCTTAATCTTAACGTGGCTAGGAGGTGCAGCCGTAGAGCTGCCCTTTACTCTTGTAAGTAAATTTATTACTTTCTCCTACTTTTCAGTATTTATTAGGTGACTTCCCTTGCTAATTTCATTACTAGCGGTTTTAATCATAGTTGCATTTTTTACTCTTTTTGAGCGAAAATTCCTTGGAATCCTTCAGACCCGCAGGGGGCCAAGGAAGGTTGGGTTTTGAGGCCTCTTGCAACCTTTTAGGGACATGTTAAAGCTTGTCACTAAGACTACGCCAAGCCCG---GGAAAAGGCTTACCTGCCGGATACCTTTTGGGACCTTTTGGAATGCTGATTCTCAGGGGATTAGTCTTCGCCTTTTTACCATTTAAGTTTTCTAAAGAA---------AATGAATTTAGAGCAGTAACAGTTTTGATGGTCCTAAGAGTCTCAAGATTTCCTCTTATCATTAGAGGATGATTCTCTCGTAGGAAGTACTCAGTGCTCGGGAGACTCCGAAGAGTCTCTCAGCATATCTCATTCGAAATCCCACTCTCCACGTCAATTATTTCCATTGTTATTTTAAACCAAACAAGAAGGATTCAT---GAAATTTTAATCCACAGGGGGGTCTGAGTCTCCTTTTTTTGCCCC---ACTCTTCCAGTTCTGATAACTTTAAGTCTTAGGGCCGAAGCCTCCCGATTACCATTTGATCTACCAGAGAGAGAGAGTGAATTAGTCAGTGGGTACTCCATTGAGTACGGCGGAGCAATGTTCACAATTATCTTTTTAGCTGAAGCTTGCTCCCTCTTACTACTTAGGGGCCTATTTTCTTCCCTCCTTTCCGGA---AGGATTAGACCACCCCTTTCCCTTTCACTGCTCTTCTTAGCA------------GTCTGAGCCCGGGGGGTCCTCCCTCGAGTTCGCTACGACCTAATAATGGAAGTTTGTTGAGTAGAGCTCGTCCCTATAGCCCTGTCTACTCTATGGATAGCGAGTTTGCCCGGATTAGTGATTTTTCTTCTTTCTTTGGTAGCTTTGGCTATTGTCTTAGTAGCTTGCATGTTCTCTGAAAAAAATATTTACTCTAAGCAAATGTTAGAGGCTTTTGAGTGCGGGTTTAGCCCAATTGGTCACCCTCACGGGCCCCTAAGGGTTCAGTTTCTACTTGTAGGAATCTTATTTCTAATCTTTGATATTGAAGTGATTATCATTCTTCCAGTTTTATTTTTA------AAGGTTAATTTTCTTCAGTGAGTTTTGTTC---TGGACATTTTACTTTATAATCATGCTTTGAGGCTTACTAGTTGAAGTAGAATTTGGAACAATCAGCTGAGGAGCAATTACTGCTTTGGTTAGCGCTGAAATTATTATGAATGTAAGGTATTGTTTAGGAACAATAAGCTTTGAAGGGACGGGAGTCCAGGGGTTTATTTGCTTAACTATGCTGGTTTTCTCAGTGGCAGACAGGGTTATAGGGCTTACTACCCTAAGGACTTCCTTTATAAGAAGAGCTCACTTAGCACCTTCAGAGCCCTATTTAGGTTTAATGCTGGATTGGATTAGATTTCTATTCCTCTTAATGGTTTTAGGGGTTAGTAGAAGGGTTTTTCTATATTCTAAGGGGTATTTTAAGAAGGATGAACAT---AACAAGTTTTTCCCAATTCTTAGCTCATTTGTGGTTTCGATATTGGTTTTAGTTTCGAGGAAGGGGTTCTTTATAGCCTTGGTAGGCTGGGACCTGCTGGGCATTAGGTCCTTGTGCTTAATCTTTTACTTTAAGTCATGGTCTTCTTACAATGGAGGACTGGTAACGTTTTTAAGGAATCGATTTGGGGATCTACTCTTGTTTAGGAGCCTAGGTTTACTCCTTGTTGGAGGGAGGGAGTGAAGGTTTCCCCTCAGTTCACCAGGACCA------------------------TGGGGCAGGGGTCTCATGCTCTTAGGGGCAATAACTAAGAGGGCCCAATATCCATTTTCAGCGTGACTTCCTTTGGCCATAGCGGCACCTACCCCAGTTAGAAGTTTAGTTCATTCCTCCACCTTGGTTACTGCTGGTCTTTTCATTGTTGTTCGACATAGTCCTTCAACCTTTCCTTCAGTATCGTGGTTAGGGGTT---------TTAGTTTCTTTTGCCAGAGTTATCTACGCTTGCTCTAGAGCTTTAATCGAGGTGGATTTGAAGAAAATCATTGCATTTTCCACTTTATCTCATTTAGGGCTAATGGTTCTCTTTGTATCAATGGGGAGAATTGAGGCGGCCCTTGTCCACATGCTTAGCCATGCTAGGTTTAAAAGGATGACTTTTTCTTTAACCTCAATAGCCGGTTTACCTTTCCTAAGGGGATTTATCTCAAAGGAAAAT---CTGTTTTGC---AATTTAAATTCTCAATGGGAAGGCCTTTTGCCAGCCCTGACCTTATCCCTAGGGACTGTGGGGACAGCGGCCTACAGCCTCCGGATCCTTCTCTTCTTACGAAACTCTCAA------------------GCCTTTAGCCCGTTTTCTTCGAATTCCTTTGGGGAATCAGAGAAATGAATGTCTCGAGGCATAATTCTTGGACTCATTATTAGATTATCTCTTTGTATTTTCCTTCCTTGG---------------GGAATCAGTGAGGAAAAG------AGCTCTCCCCTCTTAATAGAGAGGAGGACTAAGTCCTCGGTTTTCATTTTTATGCTTATCGGGATTTTATTTGTTTTTTTCTCAATT------CACTGAATGACTGAA---AGAGTTGTCACTAGTAGAATTTTTATAATTCTTTTTTCCTTAGGCATCTCTTGAGAGGTTTTCAAGCTCAGAAGATCAGTCTGACCGTCTATCTTATTCTTTCTAGGTATTTTGGGAGGACTGGTTGTCCTACTGGCTTATTCGTTTATGCTGTTCTCT---TGA---AAAGAGCAGGGGAAAGCTTTCTCAAAGGGCTTTGAG---------------------TTTAGGGGGCTACAAATGGTAGTATATTCGCCTCTAGCTTTAATCCTT---------------TGGATCCTTGAAAAGGATTCTCTTACTCTTATCGGAGGGTCTAGCAGTAAATTCCAGCCTTCTGCTTGAAGG------------------GTAGCTTCAATAAGC---TTTGACTATAGGAATCACTATATCTATTCAATTCTCTTTTTATTTAGAACTTTATTTATCATTTTATTTTCGATTGATGAAATTATCAAAAATTTCAAAAACGCGGTCAAACACGAAGGCAAAATCAC--TGAA--------------------------------TGTCTGGGGAGACTTAATTTTGGCAGAGGATAAA---------CAGGTAGAATTGTGTCAGAAAT----------AATAAGTTTAAACCAGGATTAGATACCCTGTTATTTTATTACGTA----------ATCCCATACCCCAAGTAATTAA----AGAGTTTCCTCTCCTAACTCAAACTAGTCATGGCGGCCCGTT----ATCCAGTCAGAGGTGCATGTCCC--TAA-TCGAAGCCGCCCGAA-TATCTTACCTTC-----------------------CCTGTTA-----TGTTTTGTACACCGCTGT-----GTAAGACTGCT-----AAGAAAATTTACGGTCT------------------------TC---------------------------TCCTATGGGGGAAAAGCTCAAGCCATGGTGCAGAGAATGGGAA-GGGTCGGATGTGCGCCA----GTG-----ATTGATTTCACCCAGGAGCTTGAAATAGAGCGT--------------TCTGTAAAAGAATTTGATAGTAAG-AGTAAATCATTAACTTACTCTGAATGTGGGACATAATGGGTGTACAAATTGCCCGTCATTCCGTCGGACAAGTCGTAACAAA-GTTGCCCTACTGGAAAGTGGTACCTTTTGTATCAGGGTTAGAGGATAAAAG-ATAATTAGATTATTAA------ACTCCCGAAGT--GA-AAGGATTTTGTAGGGCTGGGCCTCTACT-GTTGAATTAGTGG-GGAAAAAGCCCTACACGATG-CGAAAAGTAATTCGTCTTTCATGGT-ATCTGGTAATTCGGCAATTAAGG---GTTCAGACTGTTTAGTAAAAACATTTCCTGCCCCAA----AAATGGCAGGTAAGGCCTGCTCCCTGTC---TCTTTGATAAAGAGCCGCAGTAGCT-TGACTGTGCTAAGGTAGCATAATAATTTGCCTCTTAATTGGAGGCTGGAATGAAGGGTCTAACGTGAGCCCAACTGTCTCGATTAGGAAATAGTT--GAAGTTTAGTTCTGGGTGAAAACTCCCAGATAGGATAGAGGGACGAGAAGACCCTGTAGAGCTTTTTTACCTGGGAGGGGGAGGCTGATTAAGACCAGTCTATAACTTTAAGACTAATCGTCAAAATT---ATCCATGATCCCGTATTTTCGATCAGT--TGAAAAAGTTACCTCAGGGATAACAGCA--TAATATCTCTCCTTTAGTTCTTATGGAGTGAGGGAGGTTATGACCTCGATGTTGAATTAAG-TTCTCTTCGCTATGTAAAAGGAGTG-AATTGTTA-GTCTGTTCGACTATTTAAAACTTACATGATTTGAGTTTAGACCGACGTGAGTCAGGTCAGATTCTATCTTCTA-TCAAATTATCCTTTT----TTAGTACGAAAGGAC

Polyplax_spinulosa ATGACTATTTTTGACCCTTCGTCA------TTTAATTCCCTTGTACCTTTG------AAATGGGGCAGAAGAGTT---------------------TTTTTTCTAGTTATCTTGAGAGGCGGTTTCTGAGTCGTCAGGACTGGTTTTAAATTGCTAGTAGAGATGTTCTTATTTAACCTATGTCAAGGATTTAAAGTAATGTTTCTAAATTGGAAA---------CAACACTCTGCCATGTTAATTGGTCTGTTTTACTTAATTTTAACAATAAATGTAGTAGGGCTGTTACCTTTCTCATTTTCTGTAACAGCTCATTTGTCTTGGAGATTGACAATTTGCCTCCCCATATGATTGGGAGGGGCTATCTACATG---TTCTCAAAGGACAGAGAAGGAGCCTTGGCTCATTTTCTCCCTCATAGGGCGCCCATAGGCTTAGCGCCATTTTTAGTGATCGTAGAAATGGTGAGAATGCTTATTCGCCCGTTGAGGTTAAGGGTTCGGCTGATGGCTAATATCACAGCTGGACACATAATTTTGAGGTTGATTGAGACTCTTATCGTTAGG---AATTCTCTTTCTGTTAAGTGATTTACTCTCTTAATAGCAGGATTTCTCCTTTTTGAGTTAGGAGTGGCTTTGATCCAAGCTTATGTGCTGATAAACCTTTTGTCTCTATATTGAGAGATAGCTCCAATGATATGGTTATTTTTTAGCTTAATAACTAATTTAATTTTTCTTTTAATAATTGTT---TTAGTAACTTTCGATAAGTTTTTCTTCTGTTACCCAGAAGGTGAGAAGAAATATTCTCCTAAGTATAAAACTTGTAGTAAAATTTTCATATTTTCCACTAATCACAAGGACATTGGCGTTTTGTATTTACTATTTGGGTTTTGGGCAGGTTTAGTAGGATTTGGTATGAGAGTAATTATTCGAATTGAACTTTCTCAACCAGGTTTATGGATAGAAAGAGGACATGTTTTTAATTGCTTGGTCACTTCCCATGCATTTGTAATAATCTTTTTTATAGTTATGCCAATCATAATTGGAGGGTTTGCCAATTGGCTTGTCCCTCTGATACTTAGTGCTCCCGATATGGCTTTCCCTCGAATAAATAACATGAGGTTCCACCTTCTTCCACCGTCTTTTGTTCTTCTCTTATTGAGCTCTCTTATTCAAGGGGGAGTGGGCACGGGATGGACTGTGTACCCGCCTCTAGCTAGAGGGTTGGGGCAGCCAAGAATTTCGGTAGATCTGGCTATTTTTAGGCTCCATTTGGCAGGGGTCAGGTCTATCATAGGAGCAATCAATTTTATTTGCACGGTTATCAATTTTTGAAAGCAA------ACGATAGAAAGATTGCCTCTTTTCTGTTGAAGGGTACTAATCACTGCCGTTCTATTACTTCTTTCTTTACCCGTATTGGCAGGAGCTATTACTATGCTACTTTTTGATCGAAATGTTAACAGGTCCTTTTTTGACCCTTCCGGGGGAGGGGACCCTATTCTCTATCAGCATCTGTTCTGATTTTTTGGGCACCCTGAGGTATATATTTTAATTCTCCCAGGATTTGGTCTTATTTCCCACATAATTGTTGATGAGAGAGGTAAAAAGGAAATTTTTGGAAGTCTAGGGATAATCTATGCTATATCTTCTATTGGAGTGATGGGATTTGTGGTGTGGGCGCACCACATATTCACTGTTGGGCTAGATGTAGATAGGCGAGCTTATTTTACTAGGGCAACGATGATTATTGCTATTCCAACAGGAATTAAAGTCTTCAGGTGATTAGCCACTCTTTTTGGAGGATCTTCATTTCAAAGAGTCTCTGGTCTGTGGAGAATGGGATTTATTTTCCTTTTCACTGTTGGAGGGTTGACAGGGTTAGTCCTCTCTAACTCCTCGATTGATGTAGCCCTTCATGATACTTATTATGTAGTGGCTCATTTCCACTATGTCCTATCTATGGGGGCAGTATTTGCAATTCTTGGCAGGCTTAATCATTGATTCCCATTGGTCAGAGGGTTGGTACTAAACCAGAAATGAATGGCTATTCACTTCTGAGTGACATTCCTGAGGGTCAATTTAACCTTTTTCCCTCAACACTTCTTGGGTTTATCCGGAATGCCCCGCCGGTACATTGACTACCCTGATTCGTACAGAGCTTGAAATCTAACCTCGTCAATAGGAAGCTCTCTATCCATCGTGAGAGTGGGAATTTTGATTGGGTCTTTACTCGAAGGTTTCATGGCAAAGCGTTTACTTTTATTCCCCTTGTAT---CAAAGAAGGGTAGAAAATATTTTAGGAACCCCTCCTAGGTTTCATAGAAACGAGAGGGCACCAGCCTATTCTATTGTTAGCCTTCAAGACTCTAATTCTCCTTTGATGGGACATATTGAGAGGGTACATGATTGACTAATAGTTGTACTTGCTGGTATCATTTCCGTAGTCATTTATGTAAGAATTTGGACCTTTTTGAGAAAGGAATGA---------AACGTTTTCTTTTTTGACAGGGAGTGGTTGGAAGTCATTTGGATTATGTTCCCGTCTATTGTTCTTCTGACTTTAGCCTTTCCATCCCTTCAATGTCTTTATTTGCTAGAGGAGGTAAGACTTCCCAAGTCAACTATTAAGGCAGTAGGGCACCAGTGGTACTGGAGGTATGAATTGGTCTCTCCTACGAGCCTAGAGACCTTCTTGTTTGACTCGTACCTTTTGCCT---AAGGAGTGGGAGACTGGGGCTGCACCTCGGCTCTTGGACTGTGACTCCTCAATTCTTCTTCCAGTAGGAGAAGAGACTCGCTTAGTAGTAAGAAGAGGAGATGTAATTCACTCTTGGGCACTTCCAAGAATAGGAGTAAAGGTGGATGCAATCCCGGGTCGCTTAAACCAAGTTATTCTTTACCCATTAAAAAGGGGAATTTCATTTGGCCAGTGCTCCGAGATTTGCGGGGCAAATCACAGATTTATGCCAATTAAAGTTGAAGCCATTCCTCGAGAGGAATGGATTTCCATTCTCAAAAAAACAAAATTTCATCCCTTCCATATTTTGGATAATAGGCCATGGCCTGTGATTATAAGATTAAATATTTTAAGGGTTGCTTCCCTTCTGTCCAGATCTTTCTTT---TGAGGAGTTTCCTTACTTTTAATTGTTCCGGTCCTCGGGCTATTGTTCTCGGTATCCTTGTGGTGACGAGATGTGGTAGCCGAAAGGCTGTTTCAAGGGAATCACACAAAGGAGGTAGTTAGGGGCTTGCGAGCAGGTGTCTTAATATTCATTTTGTCCGAGGTTATATTCTTTTTTTCTATCTTTTTCGCCTTTTTTTTTATCTCTCTTTCCCCTGATGTGAGAGTAGGGATGGAATATCCTCCCGTAGGAGTGGGCTCTTTGAACATTTTTAGTGTTCCTTTGTTAAACACAATTATTTTGCTATCAAGAGGAGTTTCGCTAACTTGGAGGCACCATTCTTTACTC------GAAAAAAACTTGTTTAATTCAAACCTAGGTCTGTTAATTTCAATTTCTTTGGGGTCATGATTCCTATTTCTGCAGAATAAGGAGTATCTAGACTGTCCATTTGATATTTCTGACAGAGTGTTTGGTTCTTTATTCTTCATAGGGACTGGATTCCACGGTCTTCATGTTTTAATTGGGACAATTTTTCTGCTTATCAGATTAATTCGTTCGATAATAGGACATTTCTCTCCCTGTCATTGCTTGGGGTTCGAGGCTAGGGCTTGATATTGGCATTTTGTTGATGTCGTTTGGCTCTTCCTGTTCGTCACAGTGTACTGGTGATATCAATGAAATTATGGCTCTCTTCTGGGGTTTATCTTTTTAATTCAGCTTTTTAGAGGATTTTTTTTAGCTTTGCAATATGAGGGCTCCTCGATGTTATCTTTTAAAAGAGTAATCTCTTATATACAAAGAGTTGAGGGGGGCTGGGCTATTCGATTTGTTCACGCAAATGGAGCGTCTTTCTTTTTTGTGCTCATTTATTTGCATATTGGCCGAGGCATTTACTACGGAAGGTACAAAAATCTT---CTTGTTTGGGTCACAGGGGTAATAATGATTTTTATTTTGATGGGGACAGCGTTCCTAGGCTATGTGTTGCCTTGAGGGCAGATATCCTTTTGGGGAGCTACGGTCATTACTAATCTACTTTCTGCCTTTCCATACATTGGTGAGAGCCTGGTTTATTGATTGTGGGGCGGGTTCAGAGTGGGCAGGCCTACCTTGACTCGAATGTTTTCTATCCACTTTCTTATGCCATTTATCTTACTAGTTGTTGCCCTTTCTCACATTGCTTTTCTCCACGAAAAGGGAAGATCAAACCCCTTAGGGCTAAGTCCTCATTCCCATAAAGTAGCATTTCATCCTTATTTTGTGGTAAAGGATGTTGTTGGGCTAGTTGTGGTAGGGTTGCTGTTTGGGGCAATAATTTTGCTTAGTCCTGATTTGCTGATGGACCCAGATAACTCTATTGAGGCAAACCCAATGGTCACTCCTCCGCATATTCAACCCGAATGGTACTTTTTATTCGCTTACACCATTCTTCGGTCAGTGCCCAGGAAATTGGGAGGGGTGATGGCTATAATCATAAGAATCTTGATTTTGTTGATTTTGCCCTTT---------TCTGATGCGTCATCTGGCCGATTCAGGCTTATCCGCAGGGTAAGAACTTGAATTCAAATCAATAACTTTTTCCTTCTTACGTGGCTTGGAAGAATGCCAGTGGAAGCTCCTTTTGAGATAACAAGAAAGTATGTTACTTGTTTTTACTTTCTAACATTCCTCTTTTGATTTGCAGAGTTGATTTTATTAGTCTCGTTTCTTCTTATAGTTGCCTTTTATACTTTATTTGAACGAAAGGTCATGGGCCTTTCCCAGAGACGGCTTGGGCCTTCCAAAGTCCTTTTAAAAGGAGTGGGACAGCCATTTAGGGATGTGATAAAACTTTTATCAAAAATATCTCTTTCTCGG---AGAAATGAAGAAGAATTATGATACACCCTGGCTCCTTGCATGATAATAATTGTGTCAGTGAGGGTATTGGGGGCTTTGCCCTTTTATTTTTTCCCTTCA---------TACCAAACCTCTGGGGTCATTATCATATTTTTATTAAGAGTGAGGGCCTTTTGGCTCACATTATCTGGTTGATTTTCTAATTCGTCCTACTCAACTTTAGGGGCATGCCGAAGAGTCTCTCAATCTTTATCATTTGAAATTCCTTTAGCACTTTGTTTTATTTCCCTGTTCCTCATCTCTAAAAGGCTTAGTGTTCGG---GATTGAGGT---CAACTCGAGTTGACCGTTCTACTGGTTGCGCCC---TGAAGAGGATTAATTCTTTTGTTTTCATTCATTGCAGAAGCAGGACGGAGACCATTTGATCTTCCCGAGAGAGAAAGAGAATTGGTCAGAGGCTTCAATGTTGAGTACGGAGGCTTACTTTTTACTCTTATTTTTCTAAGTGAAACTCTCATAATACTAGTCATCAGTAGAATATTTTCGATTATTTTCCTCCAC---CAGTGTGAAGGGTGACGATTCGTTTTATCTTTATTTATTTTG------------ACTCTTATCCGTCCGTCGGTCCCTCGTATTCGATTCGACCAGGCCATGACGGCAGCGTGGCTTTCCATGACCCCGACTGCAATTAGAGCAGTCTTCTTTTTTGTACTCATTGCATTTTGTACCTTACTAATTTCACTGCTAAGG------------GCAGTAGCCCTTCTTTTCCGAAGAAAGAGGGGTAAA---ATTATCTCCTTAGAGCATTTTGAATGCGGGTTTACTCCGTTTCATCCTTCCCGAATAGTATTCTCTATTCATTTCTTTCTCATTGGTGTTTTATTTTTGATCTTTGATTTAGAGGTCGTTTCAACTTTACCAGCTATCTTTATC------CATGTCTCAATTACTCAGTGGGTGATATTC---TGGGTATTTTACTTTATAGTCATGCTCGTTGGACTTGTCTTGGAGTTCTACTGAGGGACATTTCTTTGAATAATTCTATCAGTAATTGCAGGATTAGATTTTATCTCTTCTTTAATTTTCATTGAATTATTTACTCATAACTTTTTATCGGGCTGTGTGGTCCTGGTAGCTGTCATATTTGTCACAATTGTAGCAACAGAAGGGGTGATGGGATTAAGAATCCTCAGTGCGTCAATTATTCGACTAGAAAGGTCCAGGCTGACAGGCGAGGAGGTTTCTTTTTGTTTTGATCAGACGAGTGCTGTTTTCTTATTTATAGTACTAGTTGTATCCACCAGAGTAACTAAGTACTCAAGATATTATATT---AATAGAAAAGATTTACTAAAGTTTCTGTCTCTACTAAGATTTTTTATTCTGTCAATGATTTTGTTGTGTTTGAGAACTAATTTTATCTGAAGATTAGTGGGGTGAGACGGGTTAGGGCTCACTTCTTTATTTTTGATTCTTTATTACAGAAATTGAAATTCAAGAACAGGAGGACTTGTGACTTTTTTGGTTAATCGCCTGGGAGATTTATTTCTTCTATCTTCTATCTTCTTACTGAGAGTGAGAAGGGGC---ATGTTGTGGATAGGGGGAAGAAACAAAGAG---------------------AGTTTGTTTGGCATCTTGTTTATTCTAGGAGTTTTGGCCAAAAGCGCCCAGTTTCCTTATTCATCTTGGCTGCCGCTGGCAATAGCTGCCCCAACCCCTGTCAGGAGGTTAGTTCATTCATCAACTTTAGTAACTGCCGGGGTCTTCATAATTGTCCGGAGCTATTTT---CATTTTAGGGAAGCAACCCTTTTCCTG------ATAAAGATTATGTCATTTTTAACAATCTTTTATTCAGGACTGAGGGCTATTGTTGAACAGGATTTGAAAAAAATCATTGCCTACTCGACACTCTCTCATTTGAGGATCATAGTTTTTTTAATTTCGATGGGCAGCCTAGAAGCTGCTTTATGCCATATGTTTATTCACGCTCTTTTTAAGAGCATGTTAGCTTCTATTATTTCCATGATAGGAATTCCTTTCTTGAGAGGATTCACTTCGAAGGAGATG---ATGATAAGT---ATGTCATTTAGGAATTTTGATTCTTTTTGAAATGTATTGGGGATGATTAGGAGAATCTTCTTCTCCTCCGCATATTCAACCCGAATGGTACTTTTTATGAGCCAGAGGGCTCAT---------------CCTAGAGTTTCTTTGAGG------GGCTTATCCAAGCCGAGAACTAAAATGTGCTCATCTCTTTGAGTGTCATTTAAATTAAATCTACTCAGAGGGACTTTTATTTTACCT------------------ATCCTGTTCCCAGACCTGTGTCCGCCCGCCCCAGAGGTAGAAAGGGGAGTTAAGGTGTTAGTCGTCTTATCTCTTCTCACGGGAATATTTCTTATTTTTGTAACTTTC---TTCCTAGCTTTAGGGGCT---TCTCCT---CAGGGAGTGGTAAACTTGATTGCCATGACTTTTTTCTCTGGCTTGTTCATTTTAGCATATGCAGAGTCGGTGTGGCCAGGGTCTATGTTCTTGTTAGGGATCGTAGGGGGCTTAGTTGTCCTGATAAGATTCACTTTCATAATGTTCCCCAAGGAAAGATACAAAGAGAGTTTTAAGTTT---AATCTCTTGAAA---------------------CATTCGTGGCTAACAATAGCCCCTCTGGGG---ATGTTTTTATTCCTGTCGTGAGAGACAGGAGAGAGGACATCGTGAGCTAGAGATTCAAAGGCCTCTTCTTCTCTTAGTCCTTCTTCG------GTTTGGAGG------------------ATGAGAGATAGTCTC------------ATTCTCTTCTCTCCGTTCTTATTAGTATTTATTTTTATGTTTTTATTAATTATTCTTCTTGCGGTTGAGTCAGTGACTAAGTTA---ATTGGCGCGGTCAGACATCT------GGGCAAA-TCAATTTTTCTTGGAAAAACA---------------TAAATTG------------GACAGTGAAACTTACT---GCTTAAAGGTGAAATGTTGGTAGAAATTGAA-------------ATAAACAGGGATTAGACACCCCTTTATAGGTTTGTAAG-AATAATTTCCAAAGCTAATGTTACAAA--------GATGTGG-----TTAAAATTTATTTATCA-TGGCGGTA----GAGAGTCTATTCAGAGGCATATGTCAG--TAA-ACGAAACTGCCCGAA-CACTTTACTTTT-----------------------TTAAATT--------CTTGTACATCGCTGT--TTAGAAAG-----------GTAGAACAAGTCTGTTC------------------------TGCCCAACTAG------------------ATATAGATTTTAGTAAATCAAGTCAAGGTGCAGAC-ATAAAGA-AGAAAAGATGTATGTCATTGCTCC-----A-----------------AGAGAAGGGAATATTGAAAACTTTCCC--TTTTAA---GAATTTGATAGTAAG-GGCCACTCACTATTTAGCCCTGAATGAGGA--ATTCTCTGTGTACAAATCGCCCGTCATCCCGATGGACAAGTCGTAACAAA-GTTGACTTACTGGAAGGTGGTACCTTTTGTATCAGGGTTGGAGGA-GAAAG-GCAATTT-ATTAATTG------ATTCCCGAAAA--TG-AAAGATTTT-TAAAACTGCAAAGTTAAC-GTTCAAATGTTAA-GGAAAATGGTTTTAAGGGAG-CGAAAAGTTAGACGATTTCATTGAT-ATCTGGTAATTCGGCAAA-GAGG---ACCTGGAATGTTTAATAAAAACATTTCTTTTATAGA----AAATTATAAGTAAGCCCTGCTCACTGCT---TAG--AGTAAATAGCCGCGGTATTT-TGACCGTGCTAAGGTAGCATAATAATTTGCCTTTTAATTGAAGGCTAGAATGAAAGGGTAAACCGAGGTTCAACTGTCTCTTCTTAGAGAGAA----GAATTTCAACTTTGAGTGAAAATGCTCAAATGTGTTAGAGGGACGAGAAGACCCT-TAGATCTTTGTTACCTGGGAGGGGGCTA-TACTTCAAAC---TTTAGGTTACTAAAAGAAAT---TTAATTC---AAATATGATGATCCTTGTTGGATAAA----AGAATAGATACCTAAGGGATAACAGCG--CTATTTTCTTTTTTTAGACCACTTAAAATAAAGAAGCTTGCGACCTCGATGTTGAATTAAG-TTAAATTGAAT-TAGAAAAAGAATT-CTTATTTA-GTCTGTTCGACTATTAAAAACTTACATGATTTGAGTTTAGACCGACGAGAGTCAGGTCAGATTCTATCTTCTA-TGATATTTTACCTTT----T-AGTACGAAAGGAC

Haemapinus_apri ATATCTGTGTTTGACCCCTGTTCTACTTTGCTGAGGTTAAATTTACCTTTG------AAATGGTTACTCGTGGTT---------------------TTAGTTGTATTAAGATTGAGAGGGCGTTATTGAATTCTGTCTTCTGGGTTACAGTGTGTAATAGTGTGGGTTAAAAATGGACTAATTCATGGATTACGTGAGTCCTATAAGAACTACAAA---------CAGTTCATACTTATTCTTCACACGTTGTTTTTTTTTATTTTTTCTAGTAATTTTATAGGATTATCGCCTTTTATGTTTACTCTTTCATCCCACTTGGTTTATAATTTAAGATTATGTTTCCCATTATGGTTGGGAGGAATTTTATATTCG---TGATCAAAATGTTGAAAGAAAACATTAGCGCATTTAACCCCTGTAGGTAGTCCAGTAACTTTAGCTCCCTTTTTAGTGTTGGTAGAGACTGTGAGGTTGGTTATTCGGCCAATTAGTTTAAGTGTTCGATTAATGGCTAACATAACAGCAGGTCACATAGTAATTACTCTTGCTGAACAAGGAGCCATGTCTGTAGCTTCTTATGTTGGGAGTTTTTATGTATTGTTAGTAATGGTACTATTACTGTTTGAGTTAGGGGTGGCTCTAATTCAGGCTTATGTGTTCATAAGACTAATGTCCTTGTATTGGGAAATGTCTCCTATGTGATGGTTACTGCTTGAAGTTTGATTTTTTGTGAGTTTTATAAGGTGTTGTAGA---TGTGTATATTGGGAATTGTTTGTAGAGAGGTTAGCGTCTCCAAAAGTATTTATAAAAGTCCCTGTGTTTAAAGAAGGCGATTATGTGTTACTTTCAACTAACCATAAGGATATCGGTGTTCTGTATTTAATTTTAGGTGTTTGAGCAGGTTTATTGGGGACAAGGATAAGGTTGTTGATTCGTGTTGAGTTGGGTAGAGTGAGTAGATTAATTTCTAGTGGACATGTTTATAATGTATTTGTAACAGCTCATGCATTTTTGATGATTTTCTTTATAATTATACCAGTAATAATTGGAGGTTTTGGAAATTGGTTAGTGCCAACGATGTTGGGGGCTCCTGATATAGCGTTTCCTCGAATAAATAATATAAGATTTTGATTATTACCGCCATCACTTTTTTTATTAATCTCAAGATTAATTGTTGGAGGGGGTGTTGGTACTGGGTGAACGGTATACCCCCCGCTTAGGGGGTTGGTCGGACAACCAAGAAGGTCTGTAGATTTAACTATTTTTAGATTACATTTAGCGGGTTTAAGTTCTATCATGGGTGCTATTAATTTTATTTGTACTATTGTAAACATATGGGTTTGTGGAAAAAAATTAGAACTATTACCTTTGTTCTGTTGGTCAGTTTTAATTACTGCTGTCCTACTGTTACTTTCTTTGCCGGTTTTAGCGGGTGGGATTACTATATTGTTGATAGATCGTAATATAAATTGTTCGTTTTTTGACCCTTTGGGTGGGGGTGATCCTGTGCTATACCAGCATCTATTCTGGTTTTTTGGTCATCCTGAAGTTTATATTCTTATTTTACCTGGGTTTGGGTTGATTTCGCATATAATTATGGAGGAGAGAGGAAAAAAAGAGGTTTTTGGAACATTGGGAATAATTTATGCTATGGTTGCTATTGGATTGTTAGGGTTCATTGTATGAGCACACCATATATTTACAGTAGGAATGGATGTAGACAGTCGGGCATATTTTACCAGAGCAACTATGGTTATTGCCATTCCCACAGGAGTAAAAGTGTTTAGTTGGTTGGCAACTCTCTTTGGAGGTAAGCTTGTTATATCAGTAACATTGTTGTGGTGTTTAGGTTTTATCTTTTTATTTACTGTTGGTGGTTTGACAGGTTTAGTTTTAGCCAATTCTTCAGTTGATGTAGTATTGCACGATACTTATTATGTAGTAGCACACTTTCACTATGTGTTGTCTATGGGGGCTGTATTTGCAATAATTGGAGCATTTAATCATTGATTTCCCATCATTAGCGGTGTGAGGTTAAATCAGAAGTTGATGAAAGTTCATTTTTGAATAACGTTTATTGGTGTAAACACGACGTTTTTCCCACAACACTTCTTAGGTTTGAGAGGTATACCACGCCGATATGTGGACTATCCTGATGTTTTTCTATGTTGAAACATGTTATCATCAATTGGCAGACTAGTTAGAGCTGTAGGAGTACTATTATTAGTATATACAATCTATGAAAGACTAGTGAGAAAACGGGTTATTTTGTATAGTTTATCC---GGGAATTCTTTAGATGCTATATTTGGTTGTCCACCTAATGCCCACACACATGAGAGCGTGCCTTTAGTCTTTAAAATAGGATTTCAAGATAGTTCCTCTCCAATGATGGGTTACATTACTGGAGTACATGATTGAATTATAATTGTGGTATTAGTTGTTGTTTCAATTGTTATGTATGTGTTAGGAGGGATAATACTAACAAAGGGGTGG---------GACCGTTTTCTTGTAAGTGCAGAGACTTTGGAGTTTATTTGAGCAGGACTGCCAGCTATTTCTTTAGGGTTGTTGGCCATTCCTTCCCTTCATTGTCTCTATTTAATAGAAGAAGCTTATTCTCCTTTCTTAAGGTTTAAGGTTGTAGGTCATCAATGATATTGATCATATGAATATTCTGATTATTCAGATTTGGAG---------TTTGATTCATACATGTTAAGTCAGGATTCCTTG---------------TTTCGCCTTTTGGAAGTAGATAATGCAGTTGCTATTCCAATGGATTGCGAAGTACGCGTTTTGGTTACATCCGGAGACGTAATTCACTCTTGAACAGTACCTTCGATGGGTGTAAAGAGGGACGCTATTCCAGGTCGGCTAAACCAACTAGTATTAATTGGATCAAAATTGGGATCTTATTATGGCCAGTGTTCAGAAATATGTGGGGCTAATCATAGTTTTATACCAATCAAGGTTGATGTTCTCTCGAAAGATTTATTTATGAATTGATTATTAAAAGGAGGATTTCATCCATTTCATCTAGTTAGACCTAGACCATGACCTCTATTGTTGAGAATTTCCACTTTTTCTCTAATAGTTGGATTTTATACATGAATGTCA---AGTATGGGTAGGGTGTTGATAATGTTAGGCGTGTTTAGAGTACTGTTGAGATTGTTTTGTTGGTTGCGAGATGTGATTCGTGAAAGGACCTACCAGGGGTGTCACACAATACGGGTCATGAAAGGTTTACGTTTGGGTATTGTTATATTTATTATTTCAGAGGTAATATTCTTCTTTTCTATTTTTTTTGGAGTGTTTTTTTTATCTTTAAACCCTGACGTAGTTTTGGGAAGATCTTATCCGCCTGTAGGTATTCAGCCTTTGAATTATATGGGAGTACCGTTTTTGAATACTATGATCTTGCTATCTAGAGGTGTAACTGTAACTTGATGTCATCATGGAATTATG------AGGGGTAATAAACATCATAGAGTGGTGGGGTTGATTATTACAGTGATGTTGGGAGTGTTGTTTGTTATGTTTCAGTTTGAGGAGTACTACGAAAGCTCATATACTATTGCCGATAGTGTATGTGGTTCATTGTTTTACATGTCGACTGGTTTCCATGGAATTCATGTTATGTTAGGTACTGTAATATTAATTGTAAGTTTGGTCCGATTAATAATAAACCATTTTAGAAGAACTCACAATTTAGGTTTTGAGATATCGGCGTGGTATTGACATTTTGTAGACGTTGTATGGTTATTTTTATTTATTTCTATCTACTGATGGTACCTTTGAAACTATGGTTCTTTGTTATTGGTTTGTTTAGTGATGCAGATTGTTAGTGGTATTTTCCTATCTATGCACTATGAGGCGTCAATACTAAATGCATTCTCTAGAGTAGTGTCAATGGTGAATGATGTAAACTGAGGTTGACTTATTCGAACAGTTCACGCTAATGGGGCATCATTTTTCTTTATTGCCATTTATATCCATATTGGACGGGGTCTATACTACGGAAGGTATCGAATAGTG---GGAGTCTGATTAGTAGGGGTGGTATTGTTATTTTTGCTTATGGCAACAGCATTTTTAGGTTATGTTTTACCTTGAGGTCAAATGTCATATTGAGGAGCAACTGTGATTACAAATTTACTATCTGCAATTCCTTACTTTGGTGAAGTAATAACAGGATGGTTGTGGGGAGGTTTTTCTGTAGGAAATCCGACTTTAGTACGGTTCTTTTCGTTTCATTTTGTGTTGCCTTTTGTTATTTGTTTATTTGTATTATTTCACTTGATTTTTCTTCACTGGTTTGGAAGTTCAAACCCCTTAGGATTATCTAACAAATCAGATATGATTTATTTTCACCCGTATTATAGTGTAAAAGATGTTCTAGGATTAATTGTGGCTTTGTTTATTGTTTGCGTTGTAGTTCTTCTTTTTCCTGATTTGTTTATAGACCCTGACAATTTTATTGAAGCAAATCCGATAAATACACCCCCTCATATTCAACCTGAATGATATTTTCTGTTTGCATATTCTATCCTTCGTTCAATCCCTAATAAATTAGGTGGAGTTGTATCTTTGTTAGCAAGGGTGATAATTTTAGCGTTACTTCCACTTTAT------GCAAAAGGGTTTAGATTTCGCTTTATAGGATTAAAAAAGATCTTGTATTGATTTCACGTGATAGTATTTCTTATGTTGACAGTGTTAGGTTCAATGCCTGTAGAATACCCCTATACTGTTATTAGCCAAGTGGTGGCTTTAATTTATTTTGTAAATTTTATATTCCTAGTGCAAATGTTATTTACTATTATATCAATTTTTATTGCTGTAGCTTTTTTCTCTCTTTTAGAGCGAAAGATATTGAGGATTTCCCAAAACCGAGAAGGTCCAAATAAGATTGTTCTAAAAGGGTTTTCCCAACCAATTGGTGATGCAATTAAACTCTTATCAAAATCTACTAGTCTGCCA---AACTTAGGCTTCTATTCGGTATACACCCTAGGCCCCCTAGCTCTACTTAGAATTAACACCTTCTTATGGATTACAACTCCA------TTTCTTTCCAAGTTTATCCACTTTAATCATTCCGGTATAGTTATACTACTAATCTTAAGTGTTACAGCTCTACCTACTATTTATAGAGGTTGATTCTCTAATTCAACATTTAGAACTATAGGAGCAATTCGCTCTGTTGCCCAATCTCTCTCATTCGAGATTACGTTTAGATTCAGACTATTCATTAGATTCCTTATAATTCAATCATTATGTTTGGAAAACCTTCCCAAATTTCAAAGATGGAGCTGA---CTGTTTTGATGTATTCCGTGAATTTCTCTTGTAACATTGATTTGTTTTTTAGCAGAAAGAGGACGTAGACCTTTTGACCTTCCAGAAGGTGAAAGGGAATTGGTAAGTGGTTATACTATTGAATTTGGTGGATTACATTACACATTAATTTTCCTTGGAGAGAATCTTGCTGTAATATTTATGACCATAATTTTCTCCACCACCTATCTTGGT---GGGTTTTCCTTGTGGAAAGCAAGTATGTTAGTATTAATTATT------------GTAATAATTCGAAGGTCTTACCCTCGTATTCGATACGACCAACTTATACAGTTAAATTGAGTCGGAATTTTACCTCAACTTATCTCTTCAGTTTGATTAGTGCTTGTGTTAATTATTATGTTATTCTTATTA------------------------GTATTGTCTGTGTTGATTTCTTCTATATCAGAAACAAGTTTTGATACAAATGAGTCTTTTGAGTGTGGATTTTTTACAGGAAGGGATATTCACTTACCATTTTGTGTTCATTTCTTTGTTGTAGGTATCCTTTTTGTGGTATTTGACATGGAATTGGTAGTTTCACTTCCACTAATTATAGCG------AATTTAAGAGAATTAGTATGATTGTTATGA---TGATTAGTATACAGCATTATTTTGTTTATTGGAATTCTATTGGAAGTAATATGTGGTTCAATTGATTGGGGTATAATAATTGCTCTGTTAAGAGTTGAGTTTCTATCTGTCAGTCAGTTTTATGCTGTCCTTTTTCTTGTTAACCCAAGATCATTAAATTTTAATAGATGTTTAGTGTTATTGTCAATTCTAGTGTTAGAAGGAACCTTAGGTTTGACCATTCTTGTTTCGACGAGTCTAAAAATTGATTCTACAATAATTTGGGATATTGATTGTGTAATGGTTGTAGATGGTTTAAGGTCGTTATTTTTGTTTACAGTTTTTCTTGTTAGATTTATAGTTTTCAACTATAGAGTGCACTATTTTTCTCATTTGGAAGGGTTTAATAAGTTTATTGTTACACTGTTCTTATTTGTCATATCAATGTGTATTCTTTGTTTAAGACCTAGAATGTTTTGAGTGATAGTTGGTTGAGATGGTTTAGGACTGACTTCATTTGGCTTGATTATTTTCTATCAAAATTGAAGGAGGTTTAGGAGGGGTTTATTTACATTTTTAATAAATCGAATAGGCGACATGTTTATAATTTCGGTTATTGTGATACTATCGAGA------------TGCAACATGTTGAAAAGAATATCAATAAGG---------------TCTGTTGTTCAAATTTCCGTATTCTTACTGTTAGGAGCGATAACAAAAAGAGCCCAATTGCCCTTTTCTTCATGACTTCCGCTAGCAATGGCTGCCCCCACTCCGGTTAGGAGGTTAGTTCACTCGTCAACCTTAGTAACTGCTGGAATTTATTTGCTAATTCGTTTTGAAAGT---TTATTCCCGTTAGAGGTGTTACAAGCA------CTTAAGATAGTATCAATTATAACAATTGTTTATGCAGGAGTGTCAGCACTTTGTGAGGTGGATTTGAAAAAGGTGGTAGCTCTTTCTACACTGACCCATTTAGGAATTATAACTTTATATGTGTCTATTGGGAGTGTGATTGCTGCTACAACCCATTTAGTTTTTCACGCATTCTTTAAAAGAGCATTGATTGTACTATCTTCAATAGCAGGTCTGCCTTTTTTAACTGGATTTTATTCAAAAGAAGTA---------------ATGGTGATGTTGGCAGAAGATTCTTTACTTACGCTGGTTTCGTTTTTGTTGGGGGTAATACTAACTTCTGGTTATTCAGTTCGGTTAATAGTATTAATTTTTAAATCA---------------------CCCAATTTTATTTCTGATAAAAAGGATTTTATCCAGGTAAGAGAAGGTTTGGTTGGAGTAAGGGTGAACGGGTTTTTAATTAGAGTGCTTGGTGGAAGTGTGTTACTATGG------GTGGTAATGCCTGTTACGATGGTTATG------AATTACAATTTAAGATGTGGGATCTTGAATAAGCTATTGGTTATTGTGTCACTTGCAGGAGGACTAATTGCAGCATTGTGTTCCTTA---TATTTGTGGATAGCATCA---AGATGTAGAATGGTAGTTTTGTCTTTATCTGTACAAATTGTCACATACAGGGTAATATTGGCAATGAGGGTTACTCATTATTGAATTGTAATTTTAAGGATTATGGGTTTGTTGGGAGGTTTGATTGTCTTTCTATCGTTTATCCTTATAATACTTCCT---AATCCGGATTTAGGTGTTTTTAATAGATGAAAT------------------------------TATGTAGGACTATCAATACTTATATTATTAATAATATCGACTTGTACC---------------------------------------------------------TACCTACCAGTAGTAGATAAGAATGAGCCT------------------GAATTGTTACCAATT---TCACTACATTTATGTGCTTATGGTTTATGTTTCTTTTTATTGCTTTTTGTGTTACTAATTATAGTAGGTATTATAAATTATGGTTATAAGTCTATCTATCTAGCGGTCATACACTA-----AGCTCAAGTTAACTTTCATACTGAT--------------------TGTACTAGTAACATGTAGTAGTAACTAAATTTAA----GTAGAAAGGTGAAATAAAGTAAGAAATTGTA---------ATCAATAAACTAGGATTAGATACCCTACTATA-TACAATGTA----AATATTATATCAAGGAAGTAATGGTTAT----GGTCCAT-------GAAAACTAATAACATATGGCGGTTGAC----AATCTTTCCAGAGACCCATGTTTG--TAA-TCGATAATGCACGTC-AATCTTACCCTAT----------------------TTAAG-------AATTTTGTACACCGCTGT-------CGGAAGGTT-----GTGGATAATTT------------------------------CATAATCTAAT------------------ACTATCAAAGTGATAAGTCAAGTCAAGGTGCAGACTATGGTAG-GGGTATAGTGTGGGTCA-------------------------------TTGGTCTATTGAGCAGACATAT------TATTGAAATGAATTTGGTAGTAA--ACTTAAAAATTATATTGAGTTGAAATAGCA--ATTGTTAATGTACAAATCGCCCGTCACTCCGTTGGATAAGTCGTAACAAA-GTTGATCTATTGGAAAATGGTACCTTTTGTATCAGGGACTTAGGAATAAGAAACAGTTTATA--CATA------TATCCCGAAAA--GAAAAGAATCCTTTAT---ATTAGAGGTTATTGTTTAATTAATATCTTTAAATTAT-AAAGAGTGG-TGAAATGTCATACGATTTTCTAGTT-ATCTGGTAACTCGGCAAACGTTT---AGTCTGACTGTTTACTAAAAACATTGCCTTTAGAAG--T-AAATTGAAGGTAAGACCTGCTCACTGCGTT-GATAACGTAAATAGCTGCAGTATTC-TGACTGTGCTAAGGTAGCATAATAACTTGTTTCTTAATTGGAATCTAGAATGAATGGTTTTACAAGACTAAGTCTGTCTCTGTGAAATGAAATT---GAATTTTAATTTTAAGTGAAAATGCTTAAATGCGTTAGAAGGACGAGAAGACCCTATAGATTTTTTTTACCTGGGAAGGGGTCTATACT-CAT-AAGCTATGTTTAATAAAACTAAAATAGTAGGAAATTAAATGATGATCCTCAAGTTGTGATAAAA---AGATAAATTACCTTAGGGATAACAGCA--CAATTGTCTTTCCTA-GATCCTATTGGACAATACAGATTGTGACCTCGATGTTGAATTAAG-TTATCATTTAGATGAAGAAGGTTAA--ATGGTTA-GTCTGTTCGACTATTAAAAACTTACATGATTTGAGTTTAGACCGACGTGAGTCAGGTCAGATTCTATCCTCTG-A-AATTAAGTTTACTCT-TTTAGTACGAAAGGAT
