# Supplementary material for: Fragmented mitochondrial genomes in two suborders of parasitic lice of eutherian mammals (Anoplura and Rhynchophthirina, Insecta)
Source: Sci Rep. 2015 Nov 30;5:17389. doi: 10.1038/srep17389 (PMC4663631; doi:10.1038/srep17389)
Supplement: Supplementary Dataset 9 [file srep17389-s10.doc]

17 6850

Alloeorhynchus_bakeri TTTCACTTGACCAGACGGCTTT------TTTCTT----AATGACAGACTT--------------ATATATTTCTTTCCAACTTATGCTTTCCACCGAAAGATTTTTCTAAATAGAAAACTCAACGATTAAACTTTTGGGAGGGG----AGGGTTTCATATTTGTACTTTTATTTATTTTTAAAATTTTGGTTATCCTAATTTACAGTCAGCATTACTTTCCTGCTTTCCTCCATTGTTTCTTATATTTGG--TGATAAATACCAAAATTTGCCACTATCCCCGGACCCAGGCTTATCCTTATGTTTATGAACATAGAAATATCGCCGGTCTTGCGTCGTTACGCAAATATGCGGCACTTTATAGCTCTGGAAAATTATGAAATCAATTACAGGCTTCTATATATCAATATTTATTTTTGATCGCGTTCATATCAGCTAGTTTTCGTTTAGACCTTATCAGATGCCCCTTGTGAGATTTTTATATTTATATTCCTATGCACTATC--AATTTATTCTTGATCCAGAAACAGAGATGTAAAAAA----------ATGAGAAAGTAATTTATCACAACAAAGAATGGACTTTATTATTTGGCTTGGCGGATGTGGACTCTTAGTGTTATCGATGATTAGCACCGGTCTTATGGGAGACAATTAAAGTATGTACGCCAGCTTGTATATTTTTATGTATCCGTATATGGGGTTGGAATGCTGTCCTTATATGGGCCCGAATGCTTCCCGATAAAAATAGTTTGCTTTCCCCTCTTTCTTCTTTACAGAGATGTGAAGGGGCGGACGGTGACGTTACCCCCTTCACAAATGCCAAGGGGCTCGTGATTACATTTTCCTCATTGCGGGTTCTCATCTGGGCGTAATTATTCACATATAAATCGCCTCGGATTCCCGACGATCCTTTTGTTGTCGTGGATACGCCTTTCTTTTTTCTTCCGTTTGCGGGCATACATCTTTACGACGAATTAAACTCTTTTGACCTCGGGGGGGACCATTTTACACATTTTTGTTTTGGCACCGAGTTAATTTATTTCCGGTTGGTTATTCCAATATAGAAGAAGGGAAAAGAACTTGGTCTTGGATATTAGCATATGCATGGTTTTGGTTATGTTGGCCACAATTTACGTGGATGAGTGAACCGGCTATTACTCGCACATATATGCGTCCACGGATAAATTTAGTGCTGCACCTCAGGTGTCATAATTTCCCTCATTTTGGCCTGGTTGTTTTTTTACATGGGGTTACGGGTATCTGCAATCTCATGAATATCTCAGAACTATAGTGTGCCATTCATAGTCTTCATGGGCGTTTGCATATGGAGTTATCATGTACCTTTTACGGATACATAACCCATGCTAAATCATTATATATTTACGGGTAAATACTTTTCCCACATTTTGGTTAAGGATCCCGCGTATCGATACCGAAGTTATACTGAAATATTCTCATGGTCACATTCCTATGGGTATATTTATTTATATTGGAAGATATGCAACGCAGTTTTTCCATAAATAATCAGATGATGCTCAAGACCCCCAGGACATCTAAAGATTCCATATTCACATTCACCAGAGCAATCCCCTATGACACTATTTTTCAGACAACATTTATCTACATATACATATGTATTATTATTCACCTTTATAAAATAAT------AACGTTCTCTGAGGCAACATGATTATTGACATCTCCGCATACTTATTTATGCTTCCTCCTCGATTTTAATATGAGAATAAAACCTTATACATAAGCATGGCACATGTTTGAGTAGATATCGATTAAAAATGA------TTGATCTAATAACCTCAAGACTACATAAGA--TTCGTTCTGAGTGAAACGATATATCCATAAACCCGTCGATCTGTACGCACGAGTATCATCTGACATCCTCTTGGATAAATGAGCACCCGGCGTTAACAGGTCTTAAATAACGCCACATATTAGGCATGTCGAATTGGGGCAACAAGTTATCCATGTATGATCGTAAATCCAGTTATAATGCTAAATAAAAAACACCTTCACTGTAATAAGCCTGCCTTACGGTCATGGGCTTACCTACAGGGATGTTCTGTTCA--TTAAAAATTCCTTAATTTGGATATATATCTATACATATCATGTGCGGAATGTCGGAAGACTACAGGCTCAACAAAAGTATATGGTTAATGGGATATCTTTATATTCGAGTTTTTTTATTCTTTTTGGGTTTTCAAGAGCTGCCCACTCGAATGGAGTCTGCCCCTCGGATATACTTAACCATCAATCCCTTTAAACATATCTTTTGTCGGTTACGCACTGGCCACAAGTTAT----GAGGAACGACCAACTTCAGGCTACTTACGTATTTGGATTATTTCATTTCAGGTAGATAATGATCCCTTGCATAGGATCGTTAGGTCACTTTTATGCACGGTTCAGGTTCAGTATATGGACATTTCTTCGTTGTTTGCGCATTATTACATTTCAACACACAATGGTAGAGCGCGCTGTATGCATTGTGAGTGTTGTTTTCTTAATTCATTATGTGTTTGTGAATGGGTCTTCTAGCTTGCTTTATCACTTTACGGATTTCTGCATCATAACGCAAATGATTGCTTAAAGGTATCAATTGCGGAGTAACAGGTGATCTCGAATTCAGCAAGGGCTCTTTTTTATTGTTTATTCAATGGCGGGATTATAGGTCTAAATTAT--ACACTGTAGTGGGTGTATCTTTGTATATGGACGCTTCTGGTAGTTTCCTGGGCAATTCCTTGGGGCACGTATACAACTCTTCGCGTCCTATTGGAAGATTGTAATGTTTGGGGGTTTCATGAAAGCACTTACCGTTTTACTTCATTCTTTCCTTCTATTCGCCTGTTTATCACTCTTTCTCACAACGGTCAAAACCTTGGCTAAAGAATAGAAAATCCTTCACCTATTTCATAAGATCTTAGACACATGTTTTTCTTTATATTTAGATTTGACCCGCTCTGGGACCGAAATTATCCGCAACCTTGTACCCATCAATCACCGATGTATTTTTTGCTAGCATCTCGTCATCCAAAATTGGGGGTATGCATGTGCTCATTTATATATATTTCCTTACAAAACCAAATCATCATACTTTACCATAAAAAGTTTTTGATCTTCGTAATTATCTTTACTGATGGGCCGCCGCGAGACCTTATTTACGGCAATTTACATACTATTTTTATTATGTAAATTCTTTTTTTTATTTATGTTTTTGCGTGCTTGTACTTTTGACGAGGTTTGGTAATCATTCGAAGGCCAAAAGTGGTTATGGCTTTCACCTTTCGAGGTTAATTTTTTAAGACAACTGCC--GAGTTCAATTGTATTATTGTTCCCATTTATCTTTCTTCATTTATTGGTGTTTCCTTTTGTAAGTGTAG----TTAATTGGGTGTTTTTTTTGTGTCAGTTGGGTTAGGATATTTTCGGTGTCTCAATCAATAGCATTTGGAGATCGTCGTGCCAACATTCTAGAGTAGTTTCTTATTTATAGTTCTATTTGTCAGGTTAGCTATTATTTGATTTCACAAAGTTGTTATTTTTTCAT--CCTTTTTTTGTGTTAGTCTGTTGCGAACAACGTCCCTTGATTGCGAGGGAAGGACTGTTCGGTTAAGTGATAAGAGGGGGTTGCTTATTTTTTCGATAATAAATATTTATAGATCTAGGTATTTTTATGGTGGATTGGAGTTATTTTATTAAGTGTTTTTGTTTATTTTATGGTCGGGACTTCCCGTTCGTAGAAATTATTACTACTGAGGGTTTTCCGTTCTTAATAATTTTTATATTCCTTTATCCGTTTTTAT----------------GTATTGACTTATGCAAAATCAAATGACGGAAACTTCCCTAGATGGGTTGACCTAAATCGCCGATCCTTTCATCATTTTATATGCATTTTTTTATTTGAGTGAATGTATATCTCCGCTCATACTTAATAGGATCTAGAATGATATAC--TCTCTTTTATATATCTTTCTGGTTCACAGATGTAAAGGATCTGATGACTTCTTTACCTTTAGTTGAGTTTGTATTCTTTTATTTTTTAATTAGTTTTTTAGGGAGGTTTATTATATGTTTTTACTTTCGTTGGAGGTGTTGGCTTCATTTGTTCTTATCGTGCAGGAAGATATCTGACTTGTATACATCTTTGATTAATCTTATTTATTCAGGTATATATTCTCATGTATTTTAAGTCATTAATGAGGGAAAAACGGTCGTTTTTATTGTTTATTTATATTCATATATATATATAGCCAATTGTAGATTTTTGGTGGAGGTTGGTTGTTCTAGGTTGTATTATTCAAATAAATCTAGCGCGGATTTACATTTATAACGGTGGGAGCGCATTTATTGGTGGTGATATAATTGGAG--TGCATAATTATATCTTATTG------GAGATGGTATTAATTGTTTTTTATCTGCGCTTACAAAGGCCAATCCTTTCTCTGCTCCGCGCATGCGCCCACCCGTTCGCCTGTCATCTCACTTGTACGCGGGTTATTTTATCGTTAGGG--GCTTGTAAATGATGTCTTTT--GTTTTT--GGATATACATTTATTCGGTTGGGCAGTTGATTGACTAAAGATATGCTTTCACTTAGCATTGGTTATATTCGTTTTTATGGTAATGATTTCTTTTCATTTTACCAGCTTTTAAGCCTTTATTCAAATTCTTTGGGCTCCTTTTTCGGTTTATCAAGATT--ATCTGA--ATATGTTTGATATAAATTACGTTTTTATTTTAATTCGTGGTTACTCTGTAACATCGGTTTTATATGATTTGGAAAA----------AAATTAGTTGCA------TTTAAAGAAATAGTATATTGTCATATATTTGTGTATTCATATGGGGTCATTTTCTG------------TTATTTAGTT----CCGATTTTGTATACTTACTTAATTATGCCTTTTTATATTTGGATATTTATTTACAGAT--TTTTCCTTCTAA--CACCTTAGATGGCTATATATTTCAACCTATACGCATATACGGCAATATAAATTTTGTTTCTAATTTATTTACATACAGGGATTTGTCTTTATTAATGCAGATGCTC--AA--GAAATTAAAGATAAATTCGTCT----------------TCCTATTTATTTCTCCTTATATATATAATAAA------------------GATTTTATATAATTTTAAAAAATAATACTTTATTAATAGA------------CATCATATGTACAGTTTTAAAACAGCTTATATACATATCTGTATTATTTTTATCTTATAGGTTCTGATGTAAGTTTGAGGGCGGTTATACAATA-----AGGTATAA-TGAATATTCTTGGTTAATATTAT-------------TTTGTTT-------TTGTTAATATATAATATACAC---ATTGTTAGGTGAAATGTATATGAAAGCTGAT-----------TTAAAGACTAGGATTAGATACCCTATTATT-TTAGCTGTAAAATAA----TAATCATAGTACTAAGAGTTAT----GATCTTG--------AAATTAAAATAATT-TGGCGGTAATTT---AATCTTTCCAGAGGAACCTGCCCTG-TAA-TTGATAATCCACGTTAGATTTAAC---------------------------TTTATTT-----TGACTTGTATACCTCTGT-----CATTGAATGTT-----CTGATAAGTTATTTTCT------------------------TATATTTTGGT------------------------AAAAAAATATGTCAGGTCAAGGTGCAG-TAATAATAA-AGTA--GAGGTGGGTTACTTTTAA-----ATTATT------------AGTGGATGAATATTTGAAATAATAG----AATTAAATAGGATTTGGTAGTAA--GTTTAATTAATTAATAGAACTGATTAAGGCT-CTAAATTATGCACATATCGCCCGTCGCTTTATGAAATAAGTCGTAACAAA-GTAGATCTACCGGAAGGTGGTACCTTTTGTATCAGGGTTTATTAAAT-TAT-ATTAATTATT--TAAT------TTTCCCGAAAT--TA-ATGGGGATATATTAGAATAATTGTTTATGTAGTAATA--TAAAATTAAATTCTAATATAGAGA-TGATATGTCATTCAACATTAA-TAT-ATCTGGTAACTCGGCAAATTTAA---CCTTCACCTGTTTAACAAAAACATGTCCTT-TAGTGAAT-TGATTTAAGGTCAGGCCTGCCCAATGA----TTAATTT--AATGGCCGCAGTATTT-TAACTGTGCGAAGGTAGCATAATCAATTGTTCTTTAATTGGGAACTGGAATGAATGGTTTGATGAGGGGTTAACTTTCTTTATTATATAGAAT----GAATTTAATTTTTAGGTTAAAAAGCTTAAATTAAGTTAAGGGACGAGAAGACCCTATAGAACTTTTTTGGTTGGGGTGACGAATAAATT-TTACTAACTTT----ATTTATTATTTTTCATAGATAAGTGTTTTTATGATCCGTAAATTACGATTATA-A-GATTAAGTTACCTTAGGGATAACAGCG--TAATTTCTCTAGAGA-GTTCACATTAA-TAGAGGAGTTTGCGACCTCGATGTTGGATTAAA-ATAAGTTTTGGGCGTAGTAGCTCA---TTAACTAAGTCTGTTCGACTTTTAAAATTTTACGTGATCTGAGTTCAGACCGGCGTGAGCCAGGTTGGTTTCTATCTTTAA-ATAGAATAATACTTT----TTAGTACGAAAGGAC

Bothriometopus_macrocnemis CTTCGTTTGACCGGTCAGAATTTCTT--ATCCTT----AATGCTATTCTT--------------ATGGTTATTTAT--TGCCATTGACGTAATCGGCATTACTTATAATTTTTCATGGTGAAAGCACTGAGACATTAATTTAAA------TTCCTTATTTAGTTTCATTTATACATTTTGATAAGAATGGATGTCCTATTTTACCCACAGCATTAGTTAACTTGCTGCCTCCTTTGCTACGGATATTGAT--TTAAAAAATGAAGCTTTTTCCATTGTCCGAGGAGCCATGGTTGCCCCTTTGTATATGACTGTAGTTTTATCGCCATTCTTAGATCGTTATTCAAATATGCGGCAATATTTAGTTGCTCAGGCGTTCTCATTCATAGTCTCTTTTATGAATGTGTTTGGCTATAGTTGACTTGGTGGATGTCAGCTAATTTTCTGTTTTGTATTATGTCTTTTCCATTCTTACTTTTATTTTTATTCATATATATATATGTGT--CAAATTTTACCCGTTTAGGAAGGAAGGTGGAGGCGC----TAATATGAAGCATTGTTATTTTTCACAACAAAGAATGGATTTTAATATTTGGGTTGTCGGTTATGGTTGGTTAGATATATCGATCATTGGTCCCTGGGCTCTTTGAGGCAATTTAAGTGTGTACATCAGCTTCTATATTTTTATGTATCCATATATGGGGTTGCAATGATGTCCATATTTGGGCCCGAATGCTTCCCGATAAAAATAGTTTGCTCTCCCCTCTTATTTCTTTATGCACTGATGATCGGGTGGTCGGTGACTTTACCCCCTTCTCATTTGGAGCCGGTTGAGTGGTATTATTTTCCTCATTGCGGGTAGTCATATGGGCATAATTATTCACATCTAAATTGTCTATCTTGATTACAAATCCCTTTTGTGGCGTCTATACGCATCTTTCTCTTCCTCCGTTTGCGGGCATACATCTCTTTGACGAGTTAAACTCTTTTTCCCGATTGGGGGACCATTTTACACATTTTTGTTTTGGCACCGAGTTAATTTATATCCGGTTGGTTATTCCAATATAAGATGAGGGAACCTCGCTTGGAGTTGGATATTAGCATCTACATGGTTTTGGTTTTGTTGGCCACAATTTACGTGGATGAATGAAGCGGCTATTACAGGTACATGTATGCGTCCACGGATAAGTTTAGTGTTGGACATTTGGAGAAATAATGTCCTTCTCTTTGAGGTGGTTATTTTTTTACTTGGGGCTACGGGTGTTTGCAATCTCATGAACTTATCAGAACTATAGTGTGCCATTCGTAGTTTTCATGGGCGTTTGCATTTGCAGTTTTCATGTTCCTTTTACGGTTACCTAACAAACTATAAATCATTTTGTACTTATGGGTAATTACTTTTCCCACATTTTGGCTATGGATCCCGCGTAGCGATACCGATTTAACCCTGAATCATTCTCATGGAGTGATTCGTGTGGTTTCATTTATTAGCATTAGAAGTTATTCCACGAAGCATATCATCATAAATAGTCGTGATGTTTGGGTGCCCCTCGCCATCTTGATCATTCTTATTCAAATTTTTATGACCATTCTTTCGGGAATGTCATCGTCAGACAGTATATATATACCTATGTATTCATAGTAGTTTTTATATTTTGCGTCAGCAATATGGGACGTTTTCATCAGGAATTTGAACGTTGACGTTTCCGTTTATTTGTTTGCGCATCCTCCTCAAGTTTAATTTGAGAGAAAAACCATATTCGTAAATTTGGAACATGTATGACTAGATTAAACGGTACAATAA------TAAATCTAATATCCTCTCGATTGGGAAGGA--CTCGAATTGAGTGAAAAATTGTCTCCGTGGGTGAACCGGCATATACTCAGGAGTATCATCTGGCATCCCCTTGGGTAAATGAGCGTCCGGCGATAACAACGTTTTCATTCATTCGGCTTTTAGGCATGTCGAATTGGGTCCTCATCTTATCCATTGGTGAGCATCCCTAAAATGTTAATGTTGATTACCATTCACCTTCAATGTCATTAGCCTGCCTTATTGTCTTAGCTTTAGATATATTTATTAGATTTTAA--TCTCAGTTAAAGTTATTTGCGTCTATCATTTTGTTTTTGATGTGCGGAGTTGCGGAAGACTTCAGGTGCATCTCAAGTGTCGGGTTAAATGGTTATATTTATTGTCGAGTTTTTTTTTTCTTTTTTGGTATTTTCTTCCTAACCGAGTGTTTGGGGATTGCCCCAAGGTTATGTGTGATTTTTCGCCCACCTAATCATCTCTCTTCAGGGGTAGATACTGGCCACAAGATCT----GATCAATTTCGAGCAAATGGTTGTTAACGTTTTTGGATATTTTCATATCATTATGATATAGATGTCTTACATGCGAAGCCTTGGTCATTTTTTTGCACGGTTCAGGATCAGTTTGTGGACATTTATATATTCTTGTCGCTTTAAAACATTTCAAAACACAGTGGTTGAATAGTGTGTATGCATTGTGAGTGTTGTTTTTTTTGTCGGTTATGATTAATTGAATTGGTCCTTTGGATAACTCTCTCAATGTAGGGATTTTTGCATCATAGAGAACATACTCGCTTGAAGGTGTAGATATAAGAATAAAGGGTGTTATCGTTATCAGCAAGGGCTCTTTTTTGTCTCTTATTCAATGGCGGGTTTATAGGAGTAAATTAC--GGACTGATGTGGGTATATATTTATCTATGGACGCTTGTGGTAGTCTCCTGGGCAATTCTTTGGGGCACGTATACAACTGTTCGCGTCCTTATGGACGAATGTATTGTTTGGGGGTTTCGTGAAACCACCTGTCGTTTTTCATCATTGTTTCCTTGTATTTGCATGTATCTCACTTTTTTTCATCACGGAGTCAACCCTGGTTGCAAGAAGGAAAGTTATTCACCTTTTTCATAAGAATCTGGTTATATGTACTTTTTTCTTCACGTTTTTAACCGAAGTTATGACCGAAATTACCCGCAACCATTCACCCCACAATCACCGATGTATTCTTTGCTAACATCTCGTCATTCTCAATTGGGGGTATGCTTGTTTTCATCTATTTATTTTTCCCTCTAAATAAAGTCCAAGATTTTA--------AATTTTTTTGATCAGTTCAATTATCTTTACTGTTGGTCATCCGTGACACCTAGTCACTGGCAATGTTCATAGTATTTCGTTTCTATTGATCACTATTTTTGCGTGGGTCTTTTCGTGCTTTTTCCTTTGACGAAGTTTGCATATCAAACGGTGGCCGAAAGTGGATGTGGATCTCACCTTAGGAGCATAATTATTCAAACGATCCCTCCCAGAAAAGTGGGTTTTATTTTTCCCGCATATTTGTATTCTTATGTTGATACTTCCTCAATGGAATTATC----TTGAAAAGATTTTTGTATGCTGATGGTCAGGTTAGGCTGTATACGGTGTTTCAGTCAATATCTTATGGAGGTCGGCATGGATTCATTCTAGAATATCTGTTTGGCTGTTTATATTTCTCTGGACATAGGTAATAATATTCTTCAGACAATTGTTTTTTCCTTTG--TTGTTTATATCTTTATTCTTTTGCGAAGGGCGGCCCTTGACTTCGAGGGAAGGATTGTTCGGTATCGTGATAGGGGATTCTAACTTATTTCTTCGAAAAGTCATATTTAGTCGTATTTTCAGATTTTTGC----ATGGAGATTTACCTCCGGTTATTTCT------GTGTTGATCGGGACGTCCCGATCGTTGACATTATATATTGTGGTAAATCTCCATATTTTTATTTGGATATTTGTGTTCCTGTTCCTCTGT----------------TTATGCAATTTTGATCCAGAGAGA--TCAGTCGGGAGATTGATGGGATGATCATCACCACCAACCCATAAATCATTTTATATGGATTTTTCTATTTGAATGAGTGTGTATCTCCTTATATTT----TCTGAAGAAGGCATTTATAT--ATTCATATGCGCATTTATATGGATTGATGAATTTATGGTCTTTATGAAATATATTTCTATACCTGATCGTATATCTCTTTTTTAATAGTGTTCTAAGC------CCATATTCTTGTACTTTTGTTTATGTCTGAGGGTGTGGTTAGGTTTGTAGAGGTAAATTCGTTCCCTTGACTTCTCGAATACATATTTGACATTAGGTAGTTATTTATGTCTTCGTAGACTGGTCTATTAGCGTTGTAATGAGGGAAAAATTAAAATTATGTACTTTTATTTATATTCATATTTTTTGATAGACGAATTATGGTATGTGGTGGAGGCTGGATACTCTTTTTTATATTTTTCAAATGAAAGGTAGAGGGATGTACCTCTTCAACGATGGGAGTTTATGTACTCATTGCTGAGTTTTATTGA--AGAATATCTT--------------------------------ATTTATGCCTGGGCATACAAAGGCCATACCTATCGCTGCTCCGAGCATGCGCCCACCCGTTCGCTTGTCATCTCACTTGTACGCGGATTACTCTCTCGTTAAGA--ATTTAAAAGATTGTAGCTTT--ATCTTCGTGCAGATTCGCTTTTTCATTCAGAGTGGGGATTGATTAAAAATATGCCTTCACCTTCCATTAGATATATTTTTATTCTGAAGATACTTGGCATATCAATATAGCAGCTTTTAATCTCTTTTTCTGGCTCATGTGGGGCCTTTTGCGGTTTATCAAGAAT--ATATAT--TCCTTTTCTTTCCAAAACTGATTTTGATACATTTTTGTTTGGTCTGCTTATCGCCGATATTTCGCTCTTCAATT------------CCTTTATCTATC--------TCGACCGAGATTAATCACCCCTCTATGTACATCTAGATTCGCGGAGATATATTG----------CTACATTCTCTG----CCAGTTTCTTCACCAAGATCGGGTAGAATTTT--CTTTTTTCATATTTGTGGTTATGG--ATTTTGTTAAAT--AGCCATATGGATATTATTTTGTAGACACATTTACACATCTTTATGTAAGGTC--TTGTGGTTCTTTTTTTTCACATTCGGCTTTATCTTTTCATTTATATAGATAA------ATAATT--TTTCAAACCTGTTTAA--------------AGAAATTTTTATATATCTTCTCTTACATATAG----------------ATTTTCTCCCCCAAGATGGGACTATTTGTTGAGACAATCTTC------------AATCGGTTAT----------------ATTGATTAGTTTTTATTTTTTTCTTTTCCTCCATGTGACAATCTAAGAATTCTCGCGGTAATACATTT-----AGGAAAAA---AATTT-----------------------------CAAAAGAAGAGAATTTCAAAGAAAGAAAAATTAATAAAAATTAATGGTAGAATTTTATTAAAAATTTAT---CAACCCACTGAAAAACTAGGATTAGATACCCTACTATT-------AGAGGACCAACCAACTTCAACAGAGTAGTTGATA-----AAGCTTG--------AAAAGGAGGCAGAA-TGGCGGCA----ATAAACCCGATCAGAGATGCATGTTTGAACAG--CGAAAACACACGGCAAATCCATC---------------------------TCAACCTATG--TTCTTTGTCCACTGCCGT---CTGAAGGAAGATT-----TTGACAAATCGTTCTTTTCCTGGAGTAAATATTCATGATTATTTACTTATAAAATCCGTTGAAAAAGATTTTATAGAGAGGGGTAAGTCAGGTAAAGGTGCAGGATATGGAAG-AGAA--AAAATGTGCTTCATTACT-----ATTCTTA-----------CTTACTTTAAT---TTGAAATATTA----AAAGAAGAAGAATTTGATAGTAAG-ATAAAAAAATAAAATTTATCTGAATAGGCT--CTTTATTGTGTACAAATCGCCCGTCACTCCAAAGGATAAGTCGTAACAAA-GTTAGTCTACCGGAAGGTGGTACCTTTTGTATCAGGGGTGGAAGAAAAAATTATTGATTAATATAA----------TCCCGAAAG--AG-AGTGATCGATTTGAATTG-ATGGTTAAA-GTGAAATGTTTTATCAAAAAAGTTCAGATAGAAA-TGAAATGTT-TTCAAACTCTCATGT-ATCTGGTAACTCGGCAAAAAAA----AGCCTGCCTGTTTATTAAAAACATCTCTTCAAGG---AA-GAATTTAAAGTAGGCCCTGCCCAATGC-----ATTATGTCAATGGCTGCAGAATTT-TAACTGTACAAAGGTAGCATAATAATTTGTTCCTTAATTGGGAACTGGAATGAAAGGGTTGACAAGGCTTAATCTGTCTCATTTTGGAGAAAA----AAAATTGAACTTGGAGTAAAAATGCTCCAATTAAGAAGGGGGACGAGAAGACCCTGTAGACCTTTTTTTCCTGGGGAAGGACTTG-----TTTACAAATAACGGCTAAAAAAAAACT-----AAT----GGGTTAATGATCCATAATAATTATGATAAGGAGAAAAAGGTACCTCAGGGATAACAGCA--TAATACTTTGAAAATAGACCAAATATA-ATTAAAGGCTTATGACCTCGATGTTGAATTAAG-ACTTATGGATGGAGGAAAAATTATC--CAATATG-GTCTGTTCGACCATTAAAGTCTTACATGATTTGAGTTTAGACCGATGTAAATCAGGTCAGATTCTATCTCCTT-TTTA--CGATTTCTTT---TTAGTACGAAAGGAA

Campanulotes_bidentatus TAGGTTTG--ATGTTTTTGTTGTCTTTTTAAGATATTGGTTGATATACGTAAATGTGAGAATTGGCGGTTTTTTTGAATGCGGTTGGTTCGTTCGGGTGATTTTGAAAGAATGTAATTTTTTGACTTTAACATCATGGGGTTAT------AAATGGCTGTTTTAAGGTTTTTTT--TTTTTCAATTCTGGTTGTCCTAGTTTACCTTCTCCATTTGGTAATTAGGTTCTTGTTTTGCTGGGGGTGTTATC--ATAAAATCATGATCTTCTTCCAATGTCCTTGGTCCCGTTTCTCTCCTTTTGTTTATGAACATAGACTTATCGCCTTACTTGCATCGTTATGCAAGTATGCGGCATTATATAGTTGTGGGGTTAGTCAGCTGGTTGTTCAT--TTCCATTTATGATTGGTTTTTTTTGAATTGGTGCGTGTCAGCTAGTTTAGAGTTATGTATTATTGCATATCCATTATGTTTTGTTGTTATGTAATTTTATTATTTTCTAG--TTCATATTAATTCTGTTTTCCATCGAAAGTTGTT------AAAGGTAAGTAGTTATAGTTTTTCTCAACAAAGAATGGATATTATTATTTGGATTGAGGGTTTTGGTAGGATAGGTGTATCGACGATTGGGAAGGGTCTTATAGGATCCAATTTAAGTTTGTACGCCAGCTTTTATATTTTTATGTATCCATATATGGGGTTGCAATGTTGTCCGTATGTGGGCGTGAATATTTCCCGATAAAAATAGTTTGCTCTCCCCTCTTGTTTTTTTATAGAGGTATGAAAGGGTGGACGGTGACGTTACCCCCTTCAGTTGTGGCACCGGAGGCGTGATAGCATTTTCCTCATTGCGGGTAGTCATATGGGCATAATTATTGACATTTAAATTGAGTTCCAAAGTGGATTGTCCTTTTTGTGTCGTTTATACGCTTCTTTCTCTTCCTCCGTTTGCGGGCATACATCTTTTTGACGAAATAAACTCTTTTGACCTCGGGGGGGACCGTTTTACACATTTTTGTTTTGGCACCGAGTTAATTTATCTCCGGTTGGTTATTCCAATTTAGGAAAAGGGAAATGAGTTTGGTCTTGGATATTAGCATGTGCATGGGTTTGGTTATGTTGGCCACAATTTACGTGGTTGAGTGAAGCGGCTATTACTCGCACATGTATGCGTCCACGGGTAAGTTTAGTGATGCACTTTTGGAGCGGTAATGAGCCTCGATTTGGGATGGTTATTTTTTTACGTGGGGTTACGGGTGTCTGCAATCTCTTGAATATCTCAGAGCTATAGTGTGCCATTCATAGTCTTCATGGGCGTTTGCGTTTGGGGTTATCATGTTCCGTATTTGGGTAAATGATCGTTATTAAGTCATTTTTGACTTGTGGGTAACTACTTTTCCCACATTCTGGTTATGGATCCCGCGTATCGATACCGAATTTTATCTGAATTATTCTCATGGTCCAATACTTGTGGGTTCTTTTTTTTTGTTATGAGGTTTTAGAACGAGGTTTTTTCTA----TCAGTCTTGATGATATGGTACCCCAATCCATCTTGAATGGGTCAATGTGATTTTTTCAGAAGTGGGCCTTATAGCAATTCGGTTCAGACAGTATGTGTGTTTATATTTACGTGTGTTAATAAATGTGTTTTTTTCCTGTA------AGCGTTATAAAGAGGAGGTTGAACTTTGACATCTCCTGATGTTTGCTCTTGCGCCCTCTTATACTTTATTTCGAGATTAGAACCGTGTACTTAAGTATGGCACATGTATGTCTAGATAGAGATTTCTCTCTC------TTGATCTAATATCCACTGGATTTTAAGGGA--TTCGCTCTGAGTGAAAAGGTAAGTCCTTAAAGGAAGCGGTTTGTACTCTCGAGTATCATCTGACGTCCTGTTGGGTAAGTGAGCATCCGGCGTTAACATTAGTTTACCTCCGGTGGTTGCTAGGCATGTCGAATTGGGTCATCATCTTATCCATTGTTGAGTGTCCCAGAGATTTTCGTGTTTGAATCGGTTCACCTTCAGTGTGATTAGCCTGCCTTGTATTCTTTCGTTTTCTTGATTAATTTACATTTTAATTTCGGAGGTGTTGATATGAAGTTTTTCTCATTTGTAGGCTTTGTGCGGAGTATCGGAAGACTTCAGGCACATCGAGAGTCAAAGGTTGTCTGGGTCTTTTTATTGTCGAGTATTTTTTTTCTTTTTTGGTTCTTTTCGCTTTGCCGAATGAATGGGAAGTGCCCCTTGGATGACCCTAATTATATGTCCTTATAAACTTATCTTTTCAGGGGTTCATACTGTCCACATCATAT----GAGGGATGAAAATCCTTTGGATGTATACGTTTCTGGTTGTTTTCTTCTCATAGAGATATTTCTGTCTTACATGCGAAGGTTAGGTCTTTTTTCTATACGGTTCAGGATCAGTATGTGGGTTTTTATATGTAGTTTTCGACTTGTGGCATTTCAAAGCACATTGGTTGAGCGCGCTGTATGCATTGTGAGTGTTGTTTTTTTTGTACGTTATGTGTATTTGAATTGGTCCTCTGGGTTGTTATATCATTGTTCGGATTTCTTCTTCATATCCCACATGAGAGCTTTCAGGTGTATATGTGAGAGTCCTTGGTGATTTCGAGATCAGCAAGGGCTCTTTTTTTTTGGTTATTCAATGGCGGGTTTATTAGAATAAATTAA--CCGTTGATAAGGGTTTATTTTTTTTTATGGACGCTTATGGTAGTCCCCTGGGCAATTTTTGGGGGCACGTATACAGCTTTTCGCATCCTAATGGGGTTTTGTAATGGTTGGGGGTTTTGTAAGGCCACCTCACGTTTTTCCTCATACTCTCCTTATTTTCGTTTGTTTTTCAGTTTTTTTCAAGAAAGGGGTAACCATGGGGAGTTAATCAAAAGGTTTTGTCCTATTTTTTGTGATTGGGGATTTTTTTTTTTTTTTTTATTTGTTTGTTTCAGAGTTTATGACCGAAATTATCCGCAACCATTCACCCCCCAATCACCGATGTATTTTTTGCTAACATCTCGTCGTCCTCAATTGGGGGTGTGCTTGTTTTCATTTTTCTGTTTCTCCTTATTCTTTCAATCTTAAATCGACGGTGGATAAATTTTGTGTGTTTTTGTGTTTTTTTTTACTGATGGTCATCCGTGATACCTAGATTGTGGAAGTTTTCGTTTTATTTTGTTTATTTATATGAGTGTGAGTTTTTATGTTTTTAGGTGCTTTTTCTTTAGACGAATTATGGTTGTCAGGCGAAGGCCAAAAGTGGGTGGGGGTCTCACCTTGCGAGCATAATTATAGAAAAGATAGCCC--AGAAGTATAATTATTAGCGTTCCCATATTCTTTTATTCTTGTTTTGATATTACCGTATTGAATTTTTC----TTAATTAGATGTTTCTTTGTTTTTAGGTTCGTTAGGTTATCTTCGGTGTTAGTCTCAATAGCAAATGGTGGCCGGCTTGCCATCATTCTAGAGTGGTTACTTAGATATTTTTTGTTTTTTTCTGATTCTTTCGAATTTGAGTCAGATTTTTTTTTTCCTTTCTT----TTTTGTTTATTGAGATTTTTGCGAACAACGCCCCTTGATTGCGAGGGAAGGATTAAAAGGTTTGGTAAAAGGGAATAGAACCTTATTTTTGGGAAATTTCGTTTTTGTAGATTTTTAAGTTTTTTTAGGGTCTTTTTTTTTTTTTGGGGGTTTTTTTTTTGTCTCTTACTGATTGGATATAATGCGCTTATTGGTTAATATGCTTAAATGTCTTCATTTTTTTTTTTGTTGCAGGTGGATGTATATTTTTTTAG----------------TTATAGAGATTTTGGTAAGAAAAC--GGATAATTGAATTAGATGGGATGACCATCAGAGAAAGCCCTTTGATCATTTTTTGTGGGTTTTTCTTTTTGAGTGATTATGTTGATCCATGTTGAT----AGGTTAGAAAGTTGGGTTTT--TGTTGTTTTTTTATATTTGTGGTTGTTTGAATGTATGGACTTGATGAATTGTATATTTATTCTTGAATATACTTACACTTTTATATTTTCAAGTTGAT--TTTAAAATCATTTTACTCTTTTGTTTTCGTATGAGGGTTTGGGTTCATTTGTATTTTTTCAATCAAATTTTGGAAGAATTGAGTTTTTGTGTGATCTTTCATATTTTTTTACGTGGATGTAGAGTTGTTTCTTATCAATATAATATGGAGTTTTAAAAAATTATTTGTATATATTTATCTTCATTTGTTTAGTTAGGGGATTTTTGGTATATGGTGGAGGTTGGTTTCTCATTGTTATTTTTTTCAAATGAAAGTTAAAGTCATGTACTTATTCAACGATGGGATTTTATATTCTTTTTGTTTCATTTTTAAGG--AGTTTTTTGAAATAGTTCTC--------------TCTTTTGGTTTTTTTGTTGGGCTTAGAAAGGCCAGTCCTTTCGCTGCTCCTTGCATGCGCCCACCCGTTCTCTTGTCATCTCACTTGTACGCGGGTTTTTTTATCGTTAAAG----TTCTTGGAAATTTTAGGT--ATTTTTGTAGTTATACATTTTTGCGGATAGTCGTGGGATAGATTAAAAGTATGCTTTCACCTTCCAATGGTTATATATTTGTGGATGAAGTTATTCGCAAATCATTGTATCAGCTTTTAATCCTTTCTTCACTTAGATATGGGCCCTTTTTCGGTTTTTCAAGAAT--CTTTGG--TGGTTAGAAGACGTAGATGTAGGTTTTTTTTTTTTCGTTCTTACTGTCTATCATCGATTTTTTATTTTTTCCTGTT------------TCTTTCTTATATCCAGAGGATACCTCAAATTTTTTTATAGGTGTTCTCTTTTGCGGGTATGGTTGTCTATGG------------AAGAGAAGGA----ATTCTTACGTTTTCTCTTCGAAGTGGTTTTGTAGATGCATGGTTTCTTTTTTATAGTT--TTTTTGTGTTTCTTTCAGAGTTTCTTTTATGTTTGTTTTCGTATAGACTTAGTTTCTTTTTTGATTAAGTCCTTTTATGTGTTTACCTAGGGGGTTTTGTTTGTTCTTGTATATTTTTCC--GAGATAAATTGGGTACTAGCAAAAATTT--------------TATCTGTTTCATGTATTTGGATTCATTCTTTT------------------TGAGGGGATTTTATGGGAGATTGTGTGGGGTTAAGTTGGG----------------------------------AGTTATATGTTATTTTTTATTTTTCTTTTTTTTTTTGTGTAAAGGTGTAACTAGACGGGCGGTTAGACATCCGTCGGATATCAAGAAAA---------------------------------TAAATTCGGTTCACATTCATTTACTAAATTTATTTT--TATATTCGGTAAAATGATAGTGGGAATTAGTTTAAGCATGGGGTAGAGACCAGGATTAGATACCCTGTTATTCTTTAGAATAGGAATA----------------TAGTGAGCAGATTAAGTGTGG--------AAAAGTAAAAAACA-TGGCGGTTTTCT----ATCATATTAGAGAAACATGTGCT--GAAAACGAATTTGCACGAAGAATCATTCTTTTTCTTGAAACCCTATGTTGGG---TAAATTT-----CATTTTGCATACCGCTGT---CTGAAAGACTTTT-----CTATAGAAAATGTCTCA------------------------TATCACTAGGAAATTTT------------TAATTTGTTGAAATAAGGCAAGTAAAGGTGTAGATTATGAAGA-AGAA--AGCATGTGTCTCAGTTTG-----ATTAGT--------------GGAAAGAATAAGTGAAACTTTTC-----TTGAAAAAGAATTTAACAGTAAA-GTGAGTTTATTAAACTTGTTTGAATAGGAA--ATGGAAAGTGTACAAATTGCCCGTCACTCCAGTGGAGAAGTCGTAACATA-GTTGGACTACTGGAAAGTGGTACCTTTTGTATCAGGGTGGGAGGATAAAA--GTTCATTATTATGAAG------CTTCCCGAAAA--GA-AAGGATTTATTTCTGTTTTATTTTTTCA-GTTGCA-ATTGGATTTTAAAATCAGAAATGGAAA-TGAGAAGTT-TTTCGTCTTTCGTAT-ATCTGGTAACTCGGCAAAAGAATTGGATCCTGCCTGTTTAATAAAAACATCTCCTCAATTT--GA-TTATTTGAGGTATTGCCTGCTCAATGC-----GA-AAGTAAATAGCCGCAGTATTT-TGACTGTGCAAAGGTAGCATAATAAATTGGCCTTTAATTGGGGTCTAGGATGAATGGCTTGACAAGGATTTAACTGTCTTAGGGAGAAATGTTTT--GAAATTTAATTGTTTGTAAAAATGCAGACATGTGAGAGAGGGACGAGAAGACCCTATAGATCTTATTTATTTGGGGAAAATCCTGAAATGTATTAAAACTTCGGTTAATAAAAAAATTTAATAAT----TAATTTTTGATCCATTTGTAGAGTGAAAAAATGAAAAAGTTACCTTAGGGATAACAGGA--CAATGATTGTCTTAGAGGCCTTATTTA-AGACAACGTTTGTTACCTCGATGTTGAATTAAG-TTATCTAATTAAAGGAGAAATTTTT--ATAGTTG-GTCTGTTCGACCATTAAAAACTTACATGATTTGAGTTTAGACCGACGTAAGTCAGGTCAGATTCTATCTTCTC-TTTAGTTTATTTCTTT---TT-GTACGAAAGGAT

Haematomyzus_elephantis ATTCAGTTGACCTGTCAGGTACGGTT--ATAATTTCGAAATGATTTGCTC--------------TCGGATATCTTCGGAGGTTTTGCTTGCCAGGGGTCATTTTATGGAACTGCAAGCTTACAGCTACCACAGAGTTTCATTAA------AGGTGCTTGCTGTGGCATTTATAATTTGGCATAAATGTGGCTTTCCTATCTTCCCCTCAGCATTACTTAATTGCTTAGCTCCTTTGGCGGGGTTATTATC--ATAGACTCTTAAAGTTTCTCCATTCTCCGAGGAGCCTTCCCTTCCCTTCTGTATGTGAATATTCAGCTATCGCCTTTCTTAGGTCGCTATTCAAATATGCGGCAATATTTACCTATGGCAGCGCGCTCTCACTTGTGTATCC--GCACTTACCAGCGCTTATGGTTGACTGGGTAGGTGTCAGCTTGTTTATAACTCTTACTTATGCTATTTCCAGTGTGTTTTTTATATTTTCCTATGTACTTGCGTATTC--ATGTTTTTATGTAGGAGTCCGTCTGAAATCCGAATC----TCGCCACTAAGTAGTGCTTTTTTCACAACAAAGAATGGATTTTATTCTTTGGGCTGTCGGGTGTGGTTACTTAGATTTATCGATGATTGGGAGCGGAATTATTCGAAGCAATTAAAGTATGTACTCCAGCTTCTATATTTTTATATATCCATATATGGGGTTGCAATGCTGTCCGTATCTGGGCCCGAATGCTTCCCGCTAAAAATAGTTTGCTCTCCCCTCTTTTATCTCTTTAGGGTTGTGGGAGGGTGGTCGGTGACGTTACCCCTTTCAGAGGTGGCACCAGGTAGGTGAATTCATTTTCTTCACTGCGGGTAGTCATTTGGGCATAATTATACACATGTAAATTGAGTTCCGCAATTGATTATCCTTTTTGTGTCGTTTATACGCGTCTTTCTCTTCCTCCGTCTGCGGGCATACATCTTTCTGACGAAGCAATGTCTTTTGACCTCGGGGGGGACCATTTTACACATTTTTGTTTTGGCACCGAGTTAATTTATCTCCGGTTGGCTATTCCAATATGCGAGAAGGGAAAAGAGTTTGGAGCTGGATATTAGCATATTCATGGGTTTGGTTGTGTTGGCCACAATTTACGTGGATGAATGAAGCGGCTATTACAGGCACATGTATGCATCCACGGGTAAGTTTAGTGCTTCACTTTAGGTCACCCTTTATCCCGCAACTTGGCATGGTTGTTTTTTTACGTGGGGTTACGGGTGTTTGCAATCAGGTGAGTGTTTCAGAACTATAGTGTGCCATTCATAGTCTTCATGGGCGTTTGCGTTTGCGCTTATCATGTTCCACGTACGGATTCCTTCAGTCCTCTAAGCCATTCTACACTTATGGGTAAGTACTTTTCCCACATTTTGGCTATGGATCCCGCGTAAGGATACCGAATTTTTGCTGAAGTATTCTCGCGGAGGTATACTTAGAGGTGTTTTTTTTTTTTTTGGAGGTTTCTCTGCGAACTACTTAAAGGCTGCCTCTCTTGATGTTATGGGGCCCCTCAGCATTTAGACAGTCCGTTTGTTAATTCTTTCGAAGCTTCCCAGATAATTATAGAAACCAGATTATTTATGTGTATATATATTCTTGTCTTATTTCTATTTTCTTTGGTCGGTA------AGCGAGGTGTGGAGGAGGCTGAGTTTTGGCTTGTCCATTGCTTTGCAGTTGCGTCCTCCTCATGTTTATTAGGAGAAATTAACCTTATTCATAAGCGTGGCACATGTATGTCTAGATAAGGATTGAAGGTTC------TTGATCTAATAT----AGGATTAAGTTGGA--GTCGCTTTAGGTGACAAGGTATCTCCGTCAGAAGATCGGCATGTTCTCTCGAGTATCATCTGGCCTCCGCTTGGGTAAGTGAGCGTCCGGCGTTAACATCCTGTCGAGGAAAGTGGGAGTTTGGCATGTCGAATTGGGAGTTCAAGTTATCCATTGCTAGTTGTCCAACCCATTTTAATGGTAAAATTGGTTCAAGTTCAATGTGACAAGCCTGCCATATTTAGGTGGGTATACTCATTCAAACTTATTTTATTAGGATGAATTATGGGCGTACAGTGATGCACTGACTGGCGCTTTGTGCGGAGTATCGGATCTTTTCAGGTTCAAGCATCGTATGCGGCTAGATGGTTATTTTTATGCTCGAGTATTTTTATTCTTTTTGAGTTTTTAGTTCTTAACCGAATGATGGGAGGTTGCCCCGCGGGTCAAGTTAGGCTTAAGTCCCTTTAAAGATCTTTATAGAGGGGTTCATACTGGCCACAGCCTGT----ATAGAAATAAGAACGCGTGGTTGGATACTTATTTGGCTACTTTCGTGTCATCTTGATATTCAACAGTTTCATGCGAAGGTTAGGTCGTTTTTCTACACGGTTCAGGATCAGTTTGTGGAGATTTATACGTAGTTTGCGACATTTGGCATTTCTGAACACAGTGGTTGATTTCGCTGTATGCATTGTGAGTGTTGCTTTTTTTATTCATTATGTGTAATTGAATTGGTCCTTTGGCTTGCTAGGTCACTGTACGGTTTTTTGCATCATAAACCTCGTATAAGCTTGAAGGTGTAAATATAAGAGTAATGGGTGGAGTCGCTCTCAGCAAGGGCTCCTTTTTGTTGTTTTACCAATGGCGGGTTTATAAAAGTATCACAACCCTACTGAGGTGGGTATATTTCTATGTATGCACGCTTTTGGTAGTCTCCTGGGCAATTCTATGGGGCACGTATACAATTCTTCGCATCCTATTGGGAGACTGTTTTGCTTGGGGGTTAGGTGGAGCCACTTTCCGTTTTACATCATTATCTCCTTATGTTTAGTTGCATACCAATGCGCCTCAATAAGGAGAGAACCTTGGTTTCAGGATAGAAAGTCCTTCACCTATATTGTAAGAATGTGGTTGTTTGTAGTTTTCTTTATGTGTTTACTTCCCATTTTATGACCGAAATTTCGCGCGACCCTAAACCCCCCAATCACCGATGTATTCTTTGCTAGCATTTCGTCATCCAGAATTGGGGGTGTGGTTCTGCAGATTTATTTCTGTCTCCGTATGA--AGAATTTCAGAGCGTTGACCATTCAAGTATTTTGTTTTGTAGACTTGTACCTACTGGTGGATATCCGTGACACCTTGATTATGGAAGTTTTCGTTTTATTTTGTTTATATCAATCAATGTATTTGTGTTCGTTTGTAGGTGCTTTTTCCTTAGACGAAGTTTAGTTATCAATCGAAGGCCAAAAGTGGGTCTGGCTTTCACCTTAGGAGCATAATTGTAGAATCATCACCCCGTAAGTGAAGAGTTTTTTAAATTCCCATATTTATACATGTGTATGTTGAGGTATCC----ATTAGG----TATCTCGTTGAGGGTTTTTTCTTTCTTTAGTTACTCTAGGCCATTTGGGGTGATTCAATCTGTTTCGTATGGAGGTCGAGGTATATATGCTCTAGAATACTTTCTTTCATTTAGCTTTTTATGGAATCTTTCTTGAATAGTTAAATATGACTCCCTACATTTTCGTGCCCTGCTAGGTAGTTATATTCCTTTGCGAAGGGCGAGCCTTGACTTCGAGGGAAGGACTGTGCGGTAACGTGATAGGGGATGATAACTTATTTTTGGGAAAATTCACCTTTATTGGTATGCAGATGTATTTAA--TCCTAAATGTAGGTTTAGACATTCCTGT--------ATTTATCGGGGTGTCCCGGTCGTAGACAATATTTTTTGTGGTATATTTCCATTTATAGAGGTAGTTATGTATATTTACTGTTATATAT----------------GTGTAGGGTTTTTCGGTCGATTAA--CAAGTCAAGACCTTGATGGGATGAGTTTATAGTCCGACCCTAGCCTTATCTATTGTTTATTTTTGTATTTGAATGATTATGTTCATCCCTGTTTAC--AATTTTACTGGAATTGAGATGT--TGACATTATCCTTTATCTATGGCTACGTGATTTGCTGGAGTTACTCTGCCGTAGTGTTGTGGTTGAATATTCGTATAGTTTTCTATAATCTCGGATTTTTATAGCAGCACTCTTATTTATATACTTGTGTTGGAGGGTTTGGTTAGTTATTCCCTTATAAAGACTCTCTTATGCATGAGTTCGTTTATAAGTGATACTAGTTGGTTTTTTATGTAGGTGTTCTTACGTCTATTATCAATTTAATGCGGTCCCGATTAAAATTATTTATTTGTAGTTATGCTCATATACCTGCTTAGGGTCTTTTTGGCTTATGGTGGAGGCTGGCTAGTCTTTGTTGTATTTTACAAATGAAAGTTAAAGGCCTACACTTATATAACGGTGGGAGCTTCTCTTTAGTTTGAGATTTTCATGGTC--AAATTCATTTTCGCAGGCCC--------TTGATAGTGTGCGGGTAGGGTTATTGGCTGTCAAAGGCCAGTCCTTTCGCTGCTCCTTGCATGCGCCCACCCGTTCTCCTGTCATCTCACTTGTACGCGGATTAATATATCGTTAATC--GATTTTTATCAATACAATTTGTCTTCTTCTTCGGATACATCTCTGCGGGTAGTCCTGTGATTGATTAAAAGTATGCCTTCACTTCTCAATGGGTATATAATCGTGCCTCAATTTGACTGCTTTTCAATATGCCAGCATTTAATCTTTTTTATATTCAGATGTGGTTCCTTATAGGGTATATCAAGATTATATCTTCTCTTAGGTGGAGTTATAATTTGAGATATACTTAGTCGCATTTTTTCTCGCTATCTTCGCTCTCTTTTTTCCTAACGAGAATCAGCCTTCCATTTACCTAA----AAGAGTTCTCATGAATGTAATTAGCTTATTGGATATCTAGGTTCTTGGAGGTTTGACTCACAGATTACAATGGC----------ATCTCCTCCTGTGAGTGCCTAACTTTGTTGTTGCCTTTTTGGATTTTTTGTTACCAAT--TTTTTGTTAGTC--AGATGTACGATTATTCCTATAGTTACATATGTTCTTTGAGTTATACTGAGTCGATTGCCCCTATTTACTAGCACTTGGGGCTACATTTACACTTATATATTGAGCCCGTG--AGAAGTGGCGCCTTCATCCTAGAA--------------GTGTGCTGTTGTGTGTGTCTTTCTTGCTACGA--AGATATCTGATTAAACGGGTACCTCCTGCTTGGTAAACAGTGATGGGTCAGATGAGGCGGGTTCAACTCATGAGTGATGATCCATTGTTGCCAAGTTGATATGGTTATCTATATCTCTTTAGCTTTATGTGATCTCACTAGGAAGGGGGCGGTCATACATTT-----AGGTCAAG-TAGACTTTTGGTAGAGAAGG----------------TAACACGAT-TTTGTGTTCAATTTACCGTTAAAGT---GATAAAAGGTAAAATTATTAAACAAGTTGGT---------------AAACTAGGATTAGATACCCTACTATTCTTGTGAATT-ATTAA--TTTTCTCTAGAAATTAAATGATAT----AATCATT--------AAACCTATGCAGGAGTGGCGGTTCTT----GGCCCAATCAGAGGCTTATGTTT---TAAATCGAAATTACACGAA-CATCTTACCAC---AAA------------------TTATATT-----GTCCTTGCACACCGCCGT--CGGAAGGGAAAGAG-----TTAAA----------TC------------------------TGAACCTTTATCAGCACTCATATGTGGAATACGTGGCTGAACTAAGACAGGTCAAGGTGCAGGTTATATTTATGGGGAAAATGTAAGTCATTATTTG-----AGTTATA-------------TAAGTTTATGAGATGAAATTTCCCATAATTGTTATAGAATTTGACAGTAAA-TGGAGTTTAAAATACTGCATTGAACAGGGT--CTCAAGAGTGTACAAATCGCCCGTCGCTCCCTTGGATAAGTCGTAACAAA-GTTGCACTACCGGAAGGTGGTACCTTTTGTATCAGGGTTTGAGGAATAA---AATAGTTCATTTACAA------CTTCCCGAAAG---GGGGAGATCTTTGATGGGAGTCAAGGTTGTTGTTACATTACCATCTG-CAATCTCATTAAAGTGG-TGAAATGCCTTTCGCTCCTTCTGAT-ATCTGGTAACTCGGCAATTCAGA---GTTCAGCCTGTTTAATAAAAACATGTCCTTCTGGGCATTTACATAGAAGGTCGGGCCTGCCCGCTGCACCCTTGAT-GTGAAGGGCCGCAG-AACTATAACTGTGCTAAGGTAGCATAATAATTTGCCCTTTAATTGAGGGCTAGAATGAATGGTTTGACTAGATCTCACCTGTCTCAGTTAAATGAGATTT--AAACTTAAACTTTAAGTGAAAATGCTTAAATGTTAGGAAAGGACGAGAAGACCCTGTAGATCTTGTTTGTCTGGGGCGGATGT--------CAATAACATGATT----GAATATTAAAACATGATTGTTGAACTATTAGACCCTCCACAGCGAGGGCTTATGATAAAGTTACCTCAGGGATAACAGCGCATGATATTTTCTTTTAAGATCATATTGAATAGAAAAGATTGCGACCTCGATGTTGAATTAAGCTTC-CTTTCTGAAGTAAAAGGTAGA--AAAGTTA-GTCTGTTCGACTATTAAAAGCTTACATGATTTGAGTTTAGACCGACGTGAGTCAGGTCAGATTCTATCCTTCC-TATTGAGAATTCATCTTGTCTAGTACGAAAGGAT

Psococerastis_albimaculata TTTCGTTTGACCTCACACATTTAA----TTTCAT----AATGTTAGACTT--------------CTATTTATATTTCCCTATTTTGTTGTCCACCGTTAATTGTTGATAAATATTTACTTCAAAGATTAAACTTATGGGAAAAATTAAACGGAAACATATTTATTCCTTTTCTTATCTTTAAAAGTTTGGCTCTCCTAATTTACAGACAGCAATTCATACTTGCCTTCTTCCTTTGATGCTTATCTTTGG--TGATAATTAGCACAATTTAGCACTATCCCAGGACCCGCATCTATCCTTATGTTGATGAATATAGAAATATCGCCGGACCTGCATCGCTTCGCAAATATGCGGCACTTTATACCTCTGGAAACGGCCAGTTTCCTATATTTAAATCTATTTACCAATCTTTTTACTTGAACGCGTGCTTATCATCTAGTTTGCATTTTCACCTTATCACATAACCATTGTGTTTCTTTTATATTTGTACATCTCTTCTCAATC--TTAATATT----TATCAAAAGCCTGAATAATCTTAA----AAAGAAATAAAAAAATAATTTTTCACAACAAAGAATGGACTTTATTATTTGGATTGGCGGATGTGGTCAGCTAGATTTATCGTTGATTAGCACCGGTTTTTTGAGAGACAACTAAAGTATGTACGCCAGCTTATATATTTTTATATATCCATATATGGGGTTGGAATGTTATCCCTATCTGGGCCCGAATGCTTCCCGATAAAAATAGTTTGTTTTCCCCTCTTACCTCTATTCAGAGTTGTAAACGGGCGGACGGTGACGTTACCCCTTGCAGACTTGGCACCGGGCTCGTGACTGCATTTTCCTCACTGCGGGTAGTCATCTGGGCGTAATTATACACATATAAATCGTCCAGGTTTCTTGACGATCCCTTTGTTGTCGTTTATACGCGTTTTTCTTTTCTTCCGTTTGCGGGCATACATCTCTACGACGAACTAAACTCTTTTGACCGCGGGGGGGACCATCTTACACATTTTTGTTTTGGCACCGAGTTAATTTATCTCCGGTTGGCTATTCCAGTATAGCAGAAGGGAAAAGAACTTGGGTCTGGATATTAGCATATGCATGGCTTTGGTTGTGTTGGCCACAATTTACGTGGATAAGTGAACCGGCTATTACTCGCACATATATGCATCCACGGATAAATTTAGTGCTACACCTTAGGGCAAATACTTACCCTCATCTTGAGTTGGTTGTTTTTTTACATGGGGTTACGGGTATCTGCAATCTCATGAATGTTTCAGAACTATAGTGTGCCATTCATAGTCTTCATGGGCGTTTGCATATGCGGTTATCATGTACCTTTTACGGATGTTTAAGAAACTTTAAATCATTTTATATTTATGGGTAAATACTTTTCCCACATTCTGGTTAGGGATCCCGCGTAAGGATACCGAGTTAACTCTGAAGTGTTCTCATGGAGATATTCTTATGGATATTTTTATTAATATTGGAAGTTAT--ATCGTTCCTCTTTAAATATCAATCTCCTGATGACCAAATTCCCCTCGACATCTATCGATTCCATATTTAATTAACTCAGAAGTCTCCCTTATGACATTAATTTTCAGACATCATTTATTTCTATATACTGTCATTCTATTATGTGCATATTCAAAAATAC------AACGTTTTATGAAACATTGTGAATATTGACGTATCCGGGTATTTATTTATGCCTCCTCCTCGATCTTATTTTGAGAGTACGCCCTCTTACCTAAACATGGCACATGTATGTCTAGATATCGATTATAAGTGA------TTGATCTAATGTCCCCTCGAAAAACATCGA--TTCGCTTTGAGTAAAACGATGTTTCCTAAAACCAACCGATTTGTACGCGCGAGTTTCATCTGGCATCCTCCTGGGTAAGTGAGCAACCGGCGATAACAACAGTTTTATAATACCGGTTTTTAGGCATGTCGAATTGGGTCGTCATCTTATCCATGTCTGAAGACACAAAAAGTTATAATGTTTTTTACCAAACACCTACATTGTAAGTAGCCTGCCCTACGGGCATTCGCTTTTATACTTGGATGTGATGTTAA--ACCCCAAATACTATAAATGGCTATATATATCTACATATCATGTGCGGAGTGTCGGAAGACTTCAGGAACATCTTAAGTTCCGGGATCGTGGGATATCTTTATACTCGAATTTTTTTGTTCTTTTTGGCTTTTCATCAGTTGCCCAAATGACTGGATATTGCCCCAAAGATACCCTTAACCCTCAATCCCTTTAAACATATTTCTTCTCGGATACATACTGGCCACAGCCTTT----AAAAAATATCCAACATCAGCATTTATACGTGTTTGGATTATTACATCTCAGGTAGATAGTGAGCTCTTTCATGCGATCATTAGGTCTCTTTTATGCACGGTTCAGGATCAGTATATGGACATTTATTTATATCTATCGCATAAACCCATTTCAAATCACATTGGTTGAGCGCGCTGTATGCATTGTGAGTGTTGTTTTCTTAGTACATTATGTGACATTGAATTGGTCCTTTGGCTTGCTGGATCATTATACGGATTTCTGCATCATAACGCGAATAAATGCTTTCAGGTGTCAATTGCGGAGTAAAAGGTGCTCTCGACATCAGCAAGGGCTCTTTTTTATTGCTTAACCAATGGCGGGATTATAGGTCTAAATTTT--TTACTGATATGGGTATATTTTTCTGTATGGACGCTTATGGTAGTTTCCTGGGCAATTCTTTGGGGCACGTATACAACTGTTCGCATCCTACTGGACTCATGTCATGATTGGGGGTTGCGTGAAAGCACTTACCGTTTTACTTCATTATTTCCTTATGTTTGCATGTATATCATTTTTTTTCACAACGGTCTCAACCTTGGCTAAATAAATGAAAATTCTTCACCTATTTCTTAAGAATTTGGTTTTATATCTCTATTTACTTTTACTTATGACCTAATTTAGGACCGAAATTATCCGCAACCTTGTACCCGTCAATCACCGATGTATTTTTTGCTAGCATTTCGTCATCCAAAACTGGGGGTATTTCTATCTTCATGCATTTTTATTTCCTTTCCAAAAATTATAAGGTACATTTTACATAAAACTTTTTTGGTTAGTAATTATATCTCTACTGATGGGCCGCCGTGAGACCTAATTTATGGCATCCTACTTATTATTCTTATTTTGTAAGTTCTTTTTTATATATTTGTTTGTGGGTGCTTTTACTTTTGACGAAGTTTGGTAATCAATCGAAGGCCAAAAGTGGGTATGGATTTCAGCTTAGGAGCATAATTTTACAAGAATACTACC--AAAATCAATATTATTATATTTGCCTTATTCTTCTTTATTTATATTGAGGTACCCTTTTTAGTATATAA----TTAATTGGTTTTTTTTATATTGTTAGATGGGTTAGGATATGTGCGGTGTCTCAATCAGTATCTTTTGGGGCTCGGCGTGCCAACATTCTAGAGTAGTTGGTTTTATATAAATCTATTTTGGGAGTAAGTTATGATTTTTTGGCATATATGTGTTGTTTACAGTT--CCTTTTTTTTTTTTACTCATCTGCGAACAACGACCCTTGATTGCGAGGGATCGATTGTTCGGTTAAATGATAAGAGGGGGTTGCTTATTTTTGCGATATCAGATTTTTATAGCTGTTTGTTTATTATTGGGGGAACACGGATCTTTTTTTTTGTAGTTATTCTTTCTTATTGGCCGGGACTTCCCGTACGTAGAAATTATAAGTTGTGAAAGTTTTCCGTTCTTTTTATTGTTTATTTGTGCATATGCAACTATAT----------------ATTTTGATATTTTCAAAATCTTTAGACGGAAATTTCCCTTGATGGGTTGACAAATCTCTCCGATCCTTTCTTCGTTTTTTATACATATTTTTATTTGAGTGAATGCCTATTTCCGCATAACAATAATTTCACGCAACATGTTCTAT--AAACTTTTTTATATTTTTATGGCTTTCAGATGAACAGGGCCTGATGTCTTCTGTATTTGTAGTTGATTATAGCTATATTTTTATTTATTTAATTTGATTTAG--GAAATAATTTATTATATTACTTTGGTTGGAGGGCTTGGCTTCTTTTGTTGTTGTCGTCGTGGAAGATAGGGTGGGTAGATTGGTTAATGATGATTCAGTCTTATAGTTGTTTTTATTCTTGTGTGTTGTAAGATAGTAATGGTCGAAATATCAGATTTATATTTGTTTTTTTGTTTTCATATTTTTATATAGCCAATTATAGATTTTTGGTGGAGGTTGGTTATTCTATGTTGTATTATACAAAATAATCTAAAGCGGATGTACGCATACAACGATGGGAGTATCTTTATGGATGCTGATATAATTGGAG--TGAATATTTTTATTAATATC------GAAAATTTTAATATGGGTTTATGTTTGCGCATACAAAGGCCAATCCTTTCTCTGCTCCGCGCATGCGCCCACCCGTTCGCTTGTCATCTCACTTGTACGCGGGTTATTTTATCGTTGATA--ATTTAATAAGTTATTAAATT--ATATGTGTTCGTCTACATTTATTCGGCTGGGCATTTGATAGATTAAAAATATGCTTTCACTTAGCACTGGCTATATAGACTTTGTTGGATACGATTTGTTTTCATTTTACCAGCTTTTAATCCTTTATTCAATTTCTTTGGGATCCTTTTGCGGTTTATCAAGATT--ATTTGA--AAATTTATTTAAATAAAGCTATTATTTTTATTTTTCACATTTACGTGTTAACTTCGTTATTATTAGATGTAATCTT----------AATTATCCTACA----TGTTAAGAGAGATATTATCTTTAGATATGTATGTTTATGTATATGGGGTCATATATTG------------CTATTTATGA----TTAGGGATATTTGGTTTATCAACTTTACTTGTGTTGCTACGGTCCTACATTTTTAAAT--ATTTTTATTTAT--ACCCCTTCTTGGCTACTTATCTCAACTTTTCTAGCTCTTTGGACATACTCTCTTTGTTCTTATTCTGTTTATTTATGGGGATTTGTTTTTATTAGTACTCATTTCC--AA--GAAATTTCTTACCAAAAAATTTAT--------------CTCTATTCGTTCTTCTAGACATTTTAATCTAA------------ATAATTATATACCCAATTAATATTGAAAATTTTCATAATCAAACAT------------ATATAGACAT--AAATTTAAACCAGCAAATATTTATTTTTGTAATATTTTTATGATATATGTATAAATACGCTTTTAAGGGCGGTTACACATTC-----ATTTCTAT-TCAATATTATTAAAATTT------------------TAATTTT---TTGATTTTGTTTATTTAATTTAAAT---AATTTTAAGTGAAATATTTAAGAAAATTAAT----------CAGATAAACTAGGATTAGATACCCTATTATAAT------TAATGTAAATTCAATTTTGAGTAGTA------------AATGTTTAGGACAGGAAACTTAAAGAATT-TGGCGGTGTAT----ATTCTAATTAGAGGAATATGTTTTT-TAA-TTGATAATCCACGTT-TATCTTACTTTAA----------------------TTAATTT------ATTTTGTATATCTCCGT--CTTTAAAAAATTTT-----TTAAAATATTTTTTATT------------------------TT---------------------------TTATAATTTAAAATAAGTCAGGTCAAGGTGCAGATTATATTAA-AGTT--AAAATGTATTACAATAAA-----ATTATT------------TTTGGTTTAAGATTGAAATATGTTT-----ATTAAATTGGATTTGTAAGTAAAATTTTTTGTATAATAATTATTTGAATAAAGTT-AATATATATGTACATATTGCCCGTCATTCTTAGAGACAAGTCGTAACAAA-GTAGTTTTACTGGAAAGTGGTACCTTTTGTATCAGGGTTTAA---ATATA--AAAATTTATATAAGAT------TATCTCGAATG--GT-TTTGATTTAATTATATATAATTATTAAT-GTAGAAAAATTAATATTTAATATATAATTAGAAA-TGAAAAGTTAGTCGTTTAACCATAA-TTCTAGTAACTCGACAAAAGTAA---TTT-CGCCTGTTTATCAAAAACATGTCTTTTTGGTAA----AATATAAAGTCCAATCTGCTCACTGA-----AGAT-TTAAAGAGCCGCAGTATAC-TGACTGTGCAAAGGTAGCATAATCAATAGTTTTTTAATTGAGAACTTGTATGAAAGATTAGACGAGAATTAAATTTTCTTTATTCTACTAAATT---TAATTTTAATTTTAAGTGAAAATGCTTAATTTATTTTAAAAGACGAGAAGACCCTATAGATTTTTTTTAATTGGGGCGATTTTATAAAA-TAAAAAACTTAAATTTTTTATTACCATAAATTATTGAA---TTTTCTGATCTAAAAATTTTAAAGAAA-A-GATTAAATTACCTTAGGGATAACAGCG--TAATTTTATTTAAGA-GTTCATATCTA-TAATAAAGTTTGCGACCTCGATGTTGAATTAAG-ATAATATATTGGAGTAGAAATTAAT--ATAATTA-GTCTGTTCGACTATTAAATTCTTACATGATTTGAGTTCAGACCGGCGTGAGCCAGGTCAGTTTCTATCTTTAA-TTTTTTTTGTTTAGA----TAAGTACGAAAGGAC

Longivalvus_hyalospilus TTTCGTTTGACCTCACACATTTAA----TCTCAT----AATGTTAGACTT--------------TTATTTATATCTCCTTATTTTGCTGTCCACCGTTAATTATTGATAAATATTTATCTCAAAGATTAAACTTATGGCAAAAATTAACTGGAAACATATTTATTCCTTTTCCTATTTTTAAAAGTCTGGTTTTCCTAATTTACAGACAGCAATTCATACTTGCTTTCTTCCTTTGATGCTTATCTTTGG--TGATAATTAGCACAATTTAGCATTATCCCAGGACCCGCATTTATCCTTATGTTGATGAATATAGAAATATCGCCGGACCTGCATCGCTTCGCAAATATGCGGCACTCTCTACTTTTGGAAACGGCCTCTTTCATTTATTTAAATCTGTTTACCAATCTCTCTACTTGAACGCGTGCTTATCATCTAGTTTGCATTTACACTTTATCACATAACCATTGTGTTTCCTTTCTATTTGTACACCTACATTCAATC--TTAATATT------TATCAAAATCTTTCTCTCTCTT----ACAAAAAAACATAAGTAATTTTTCACAACAAAGAATGGACCTTATTATTTGGATTGGCGGATGTGGTCAGTTAGATTTATCGTTGATTAGCACCGGTTTTTTGAGAGACAACTAAAGTATGTACGCCAGCTTATATATTTTTATATATCCATATATGGGGTTGGAATGCTGTCCTTATCTGGGCCCGAATGCTTCCCGATAAAAATAGTTTGTTTTCCCCTCTTACCTTTATTCAGAGTTGTAAACGGGCGGACGGTGACGTTACCCCTTGCAGACTTGGCACCGGGCTCGTGACTGCATTTTCCTCACTGCGGGTAGTCATTTGGGCGTAATTATACACATATAAATCGTCCAGGTTACTTGACGATCCTTTTGTTGTCGTTTATACGCATCTCTCTTTTCCTCCGTTTGCGGGCATACATCTTTACGACGAATTAAACTCTTTTGACCGCGGGGGGGACCATCTTACACATTTTTGTTTTGGCACCGAGTTAATTTATTTCCGGTTGGTTATTCCAGTATAGCAGAAGGGAAAAGAACTTGGGTTTGGATATTAGCATCTGCATGGCTTTGGTTGTGTTGGCCACAATTTACGTGGATAAGTGAACCGGCTATTACTCGCACATATATGCATCCACGGATAAATTTAGTGTTACACCTTAGGGCAAATGCTTACCCACATTTTGAGCTGGTTGTTTTTTTACATGGGGTTACGGGTATCTGCAATCTCATGAATGTCTCAGAACTATAGTGTGCCATTCATAGTTTTCATGGGCGTTTGCATATGCGGTTATCATGTACCTTTTACGGATGTTTAAGAAACTTTAAATCATTTTGTATTTATGGGTAAATACTTTTCCCACATTCTGGTTAGGGATCCCGCGTAAGGATACCGAGTTAACTCTGAAGTATTCTCCTGGAGATATTCTTATGGATATTTCTATTAATATTGGAAGTTAT--ATCGACCCTCTTTAAATATCAATCTCCTGATGACCAAATTCCCCTCGACATCTAGCGATTCCATATTTAATTAACTCAGAAGTCTCCCTTATGACATTAATTTTCAGACATCATTTATCTTTATATACTGTCATTCTATTATATGCATATTCAAAAATAC------AACGTTCTATGAAACACTGTGAATATTGACGTATCCGGGTATCTATTTATGCCTCCTCCTCGATCTTATTCTGAGAGTACTCCCTCTTACTTAAACATGGCACATGTATGTCTAGATATCGATTCTAAGTGA------TTGATCTAATATCCCCTCGAAAAATCACGA--TTCGTTTTGAGTAAAACGATGTATCCTAAAACCAACCGATTTGTACGCGCGAGTCTCATCTGGCATCCTCTTGGGTAAGTGAGCAACCGGCGATAACAACAGTTTTATAATACCGGTTTTTAGGCATGTCGAATTGGGTCGTCATCTTATCCATGTGTGAAGACAAAAAAAGTTATAATGCTTTTTACCAAACACCTACATTGTAAGTAGCCTGCCCTACGGGCATTCGCTTTTATACTTGGATATGATGTTCA--TCCCGAAATATTATAAATGGTTACATATTTTTACATATCATGTGCGGAGTGTCGGAAGACTTCAGGAACATCTTAAGTTCCAGGATCGTGGGATATCTTTATACTCGAATTTTTTTATTCTTTTTGGCTTTTCATCAGTTGCCCAAATGATTGGATATTGCCCCAAAGATACCCTTAACCCTCAATCCCTTTAAACATATTTCTTCTCGGGTACATACTGGCCACAGCCTTT----AAAAAATATCCAACATCAGCATGTATACGTATTTGGATTATTACGTCTCAGGTAGATAATGAGCTCTTTCATGCGATCATTAGGGCTCTTTTATGCACGGTTCAGGCTCAGTATATGGACATTTATCTATATTTATCGCATAAACCCATTTCAAAACACATTGGTTGAGCGCGCTGTATGCATTGTGAGTGTTGTTTTTTTAGTACATTATGTGACATTGAATTGGTCCTTTGGTTTGTTGGATCATTATACGGATTTCTGCATCATAACGCGAATAAATGCTTTCAGATGTCAATTGCGGAGTAAAAGGTGTTCTCGACCTCAGCAAGGGCTCTTTTTTATTGTTTAATCAATGGCGGGATTATAGGTCTTAATTTT--TTACTGACATGGGTATATTTTTCTGTATGGACGCTTATGGTAGTCTCCTGGGCAATTCTTTGGGGCACGTATACAATTGTTCGCATCCTATTGGACTCATGTCATGATTGGGGGTTGCGTGAAAGCACCTACCGTTTTACTTCATTATTTCCTTATGTTTGCATGTATATCATTTTTTTTCACAACGGTCTCAACCTTGGTTAAATAAATGAAAATTCTTCACCTATTTCTTAAGAATTTGGTTTTATATTTTTATTTATTTTTACCTATAACCTAATTTAGGACCGAAATTATCCGCAACCTTGTACCCGTCAATCACCGATGTATTTTTTGCTAGCATTTCGTCATCCAAAATTGGGGGTATTTTTGTTTTCATGCATCTTTATTTCCTTTCCAAAAACTATAAGGTACATTTTACCTAAAATTCTTTTGACTAGTAATTGTATCTCTACTGATGGGCCGCCGTGAGACCTAATTTATGGCATCTTACTTATTATTTGTATTCTATAAGTTCTTTTTTATATATTTGTTTGTGGGTGCTTTTACTTTTGACGAAGTTTGGTAATCAATCGAAGGCCAAAAATGGTTATGGATTTCAGCTTAGGAGCATAATTTTACAAGAATACTACC--AAAATCAATATTATTATATTTGCCTTATTCTTTTTTATTTATATTGAGGTACCCTTTTTATAATATAA----TTAATTGGTTTTTTTTATACTGTTAGATGGGTTAGGATATGTGCGGTGTCTCAATCAGTATCTTTTGGGGCTCGGCGTGCCAACATTCTAGAGTAATTGCTTCTATATAAGTTTATTTTCGGAGTAAGTTATGATTTTTTGGCATATATGTGTTGTTTACAGTT--CCTTTTTTTTTTTTACTCATTTGCGAACAACGACCCTTGATTGCGAGGGATCGATTGTTCGGTTAAATGATAAGAGGGGGTTGCTTATTTTTGCGATAGCAGATTTTTATAGTTGTTTGTTTATTATTGGGGAAACAGAGATTTTTTTTTTTGTAGGTATGGTTTCTTATTGGCCGGGACCTCCCGTACGTAGAAATTATAAGTTGTGAAGGTTTTCCGTTCTTTTTATTGTTTTACTAATTTTAATGAACCAAAA----------------AGCTATTAAATTATAAAAATACCTTGCGGAAATTTCCCTTGATGGGTTGACAAATCTCTCCGCTCCTTTCCTCGTTTTCTATACATATTTTTATTTGAGTGAATGCTTATTTCCGCATAAAAATTATTTCAAGCAGCATGCTGTTT--AAACACTTTTTTATTTTTATGGTTTTCAGATGAACAGGATTTGATGTCTTTTATATTTTTAGTTGATTGTAGTTATATTTTTATTTATATAATTTGATTTTC--GAAATAGTTTATTATATTACTTTGGTTGGAGGGCTTGGTTTCTTTTGTATTTAGTCTTAGAGTAGATAGGGTAGATAAATTGATTAATGATGATTCAGTCTTATAGTTGTTTTTATTCTTGTGTGTTGTAAGATAGTAATGGTCGAAATATCAGATTTATATTTGTTTTTTTGTTTTCATATTTTTATATAGCCAATTATAGATTTTTGGTGGAGGTTGGTTATTCTATGTTGTATTATACAAAATAATCTAAAGCGGATGTACGCATACAACGATGGGAGTATATTTATGGATGCTGATATAATTGGAG--TGAATATTTTTATTAGTGGA------GAAAGTTTTTATATGGGTTTATATTTGCGCATACAAAGGCCAATCCTTTCTCTGCTCCGCGCATGCGCCCACCCGTTCGCTTGTCATCTCACTTGTACGCGGGTTATTTTATCGTTGATA--ATATAGTAAGTTATTAAATT--TTCTTTGTTCGTTTACATTTATTCGGTTGGGCATTTGATAGATTAAAAATATGCTTTCACTTAGCACTGGTTATATAGACTTTGTTGGATACAGTTTGTTTTCATTTTACCAGCTTTTAATCTTTTATTCAATTTCCTTGGGATCCTTTTGCGGTTTATCAAGATT--ATTTGA--AAATTTATTTAAATAAGGTTATTATTTTTATTTTTCACATTTACGTGTTAACTTCGTTATTATTAGATATAATCTT----------AATTATAGTACA----TGTTAAGACAGATATTATTTTTAGATATGTATGTTTATGTATATGGGGTCATATATTG------------TTATTTATGA----TTAGGGATATTTGATTTTTCAATTTTACTTATGTTGTTGTGGTTTTAGATATATAAAT--ATTTTTATTTTT--AACCCTTCTTGGTTGTTTATTTCAACTTGTCTAGATACATGGACATAC--TCTTTGTTTTTATTTTATTTGTTTATGGGGATTTGTCTTTATTAGTACTCATTTCC--AA--GAAATTTCTTAACAAAATTTATAT--------------TTTTATTCGCTTTTATATTTATTTTCTTATAA------------ATAATTATATAACTAACTAAAATTGAATATTTAAATAATCAAATAT------------ATATAGACAT--AAATTTAGACCAGGAATTATTTATTTCTGTAATATTTTTATGATGTATGTATAAATACAATTTTAAGGGCGGTTATACAAAT-----CATTTTGAGTAAATTTTTATTGGTT--------------------TATATTTATTTTTATTTTTATTATAAATTTTGGAT---GTTATTTGGTGGAATTATATATTAAATCTAT----------ATTATAGACTAGGATTAGATACCCTATTATTATTAGTTGTAAATTAG----TAACCATATTATTAATAGTTAT----ATTCTTT--------AAATTAAAAGAACT-TGGCGGTAATTT---AATCTTTCCAGAGGAACCTGTCCTT-TAA-TTGATATTCCACGAAGTATATTACT--------------------------TTAATTTT----AAGCTTGTATACCTCTGT-----TGATGAATGTT-----TTGTAAGAATATTTTCT------------------------------------------------ATGTTTAATTTTATATTTAATGTTAGGTCAAGGTGCAG-CAATATTAA-AGTA--GTGATGGGTTACATT--------ATTAATATA---------TTTGGATTAATAAATTGATATTTAT----TATGAAATAGGATTTGGTAGTAA--ATTCTTTTATCTATTAGGGTTGATTTTTGCT-CTAGATTATGTACATATCGCCCGTCACTCTACGGGATAAGTCGTAACAAAGGTAGTTTTACTGGAAAGTGGTACCTTTTGTATCAGGGTTTAA---ATAAG--AAAGATTATATAAATT------TATCTCGAATG--AT-TTTGATTTAATTTTTTATAATTATTAAT-GTAGAAAAATTAATATTTAATAAATAATTAGAAA-TGAAAAGTTAGGCGTTTAATCATAA-TTCTAGTAACTCGACAAAAATAA---TTT-CGCCTGTTTATCAAAAACATGTCTTTTTGAAAT----AATTTAAAGTCCAATCTGCTCACTGA-----AGAT-TTAAAGAGCCGCAGTATAT-TGACTGTGCAA-GGTAGCATAATCAGTAGTTTTTTAATTGAGAACTTGTATGAAAGATTAGACGAGAATTATATTTTCTTTATTTTAATAAATT---TAATTTTAATTTTAAGTGAAAATGCTTAAATTTTTTTAAAAGACGAGAAGACCCTATAGATTTTTTTTAATTGGGGCGATTTTATAAAA-TAAAAAACTTAAATTTTT-ATTACCATAGATAATTGAA---TTTTTTGATCTAAAAATTTTAAAGAAA-A-GATTAAATTACCTTAGGGATAACAGCG--TAATTTTATTTAAGA-GTTCATATCTA-TAATAAAGTTTGCGACCTCGATGTTGAATTAAG-ATAATATATTGGAGTAGAAATTAAT--ATAATTA-GTCTGTTCGACTATTAAATTCTTACATGATTTGAGTTCAGACCGGCGTGAGCCAGGTCAGTTTCTATCTTTAA-TTTATTT-ATTTAAG----TAAGTACGAAAGGAC

Pediculus_capitis ATTCTCTTGACCTCACTCATATCTGG--TTAAGT----AATGTTATATCT--------------TTCCTTTTTTATACGGAGTATATTATCATCGGTACGTTTAGTAGTTGTTTAAAATTTTATAACATAATTC----------------------ATATAGTTTCGTTTATATATTTACCTAAACATTCCTATCCTTGTTTCCTGACTCCATTAGGTAATTGGCTTGTTCCTTTGATAGGGGTGTTATC--TTAAAGTCATCGGGTTCTGCCACTCTCCTAGGAGCCATATCTAGCCTTTTGTGTATGATTTTAGGTTCATCGCCGTTCCTAGGTCGCTCTGCAAATACGGGGCATTATATAACTTTGAGAGGTTTCTCGCGTCTCTGTCTCCTTTCATGCGCTAGTCTCTTTGCGCGACTTTGTTCTTATCATCTAGTTTAGAACTGTTCATTATGGACTTGCCTCATTGACTTTATAATATGTATTTGTTTTATTATTTAC--ATATTATTATAAATGAAATCAATATTGAAATTAATT----AAAAAAACATTCAACTTCATTTTCACAACAAAGAATGGTTTTTATTTGTCGGGTTGTTGGCTTTGGTTAGTTAGTTATATCGTTGACTTCAGACGGTTCTTTTCGAAGCACTTAAAGTTTGTACTCCAGCTTGTATATTTTTATGTATCCGTATATGGGGTTGCAATGTTGTCCTCATTTGGTCCCGAATGCTTCCCGATAAAAATAGTATGCTCTACCCTCGGATTTCTATAGAGTCTTGTCAGGGGGTGGACGGTGACGTTACCCCCTAGTCCTGAGGCACCTCGTTCGTGATTGCATTTAGCTCATTGCGGGTAGTCATTTGGTCGTAATTATAGACATTTAAATTGCCCATATTGGTTGTCGCTCCTTTTTGTGAGGTTTGTACGCTTTTTTTTCTTCCTCCGTTTGCGGGCATACATCTTTATGACGAATTAATGTCTTTTGACCTTGGGGGGGACCGTTTTACACATTTTTGTTTTGGCACCGAGTTAATCTATCTCCGGTTGGCTATTCCAATGTGTGATGTGGGAAAAGAGTTTGGTCTTGGATATTAGCATTCGCATGGGCTTGGTTGTGTTGGCCACAATTTACGTGGTTGAGTGAAGCGGCTATTACAGGCACATACATGCATCCACGGGTAAGTTTAGTGTTGGACTTTTGGCCAATTAAAGAGATAGTTTTTGTCTTGGTTATTTCTTTACATGGGGTTACGGATGTCTTCAATCTCGTGAGTTCCTCAGAACTATAGTGTGCCATTCATAGTTTTCATGGGCGTTTGCATTTGGGCTGAACATGTTTCCTGGACGGCTAACTCGAATCTTATAAGTCATTTGTTAGTTGTGGGTAATTACTTTTCCCACATTCTGGTTGCGGATCCCGCGTATCGATACCGAGTTACTAGTGAAAAATTCTCATGGAGCTATACACTTGGGTGTATTTCTTTGCCTATGAAGTTTCAACCCAAAATGTTTAGGAGCAGTTCAGACTCCCGCTATGGATCCGCAGATCATCCATTACCTACTTACAGGTTGGGTTCAGAAGAATCCCTTATGTTTGTTGGAACTAGACTGTTCATGTTGGTGGGTATTCTTGTATTAGTGCGTAGTTTTTTATAATCTG------AATATATTATGGCTGAAGTTGAATGTTGGTATTTCCTCCTTCTTGCGGTTATTTCCTCCTCATGTTTATTATGAGAGTCTTCCCGCATAGTTAAGTGTGGCACATGTTTGTCTAGATAGGGATGGAAAATGA------TTGATCTAATATAATTGAGACTGATCTCTGCCTTCGCTTTGAGCGATTAGGTTTATCCTATTACGAGTCGGCATGTACTCGCGAGTATCATCTGGCATCCATATGGGTAAGTGAGCATCCGGCGTTAACAGCCTATTATCTTAAATGGACTCTAGGCATGTCGAATTGGGGCTACAAGTTATCCATAAGTACACCTCCAAGAGATTATAATGGTAAGATTAATTCACCTTCACTGTGAGTAGCCTGCCATTTTTAGTTTCCTTTTTTCGCTCATACTTTGTGATAAGGCTTATCTTTAATTTATATAGATGTGTTCTCTTATGTTCTTTGTGCGGAGTACCGGAGCACTTCAGGAACAACATGAGTATGCGGTTCGTTGGATCTATTTATGCTCGAGTATTTTTTTTCTTTTTAGCCTTTTTCTTCTTAGCCGAGTTCTTGGCTCTTACCCCGTGGGTAGCCGTGGGTTTGGGTCCCTTTAATCATTTTTCTTCAGGGGTTCATACTGGCCATAGACTTT----AGAAAAATTCTCAGCTATGGTTTTATACTTATTTGGCTGTTTCTATTTCAGCGTGATAAAACAGTCTTACATGCGAAGAGTTGGTCGTTTTTCTATACGGTTCAGGGCCAGTTGGTGGGTGTTTATACATAGACATCGCTTATTAACATAAAAAAACACATTGGCTGACTGCGCTGTATGCATTGTGAGTGTTGTTTTTTTACTACCTTATGTGATCTTTAATTTGGTTTTTGGTTTTCTTCATCAATTTAGGGCTTTCTGCTCCATAGAGCTCACAA--TCTTTGAGGTATTTATGATTGAGTAAAGGGTGTTATCGAGTTCAGCAAGGGCTCTTTTTTATCTGTTAGTCAATTGCGGGTTTGTTGGTGTTACCAAA--TAGTTGTTTCGGATTCATCTCTCTATATGCGCGCTTATGGTAGTCTCCTGGGCAATTCTTTGGGGCACGTATACAACTTTAGGCATCCATGTGGAGGATTGTATTGGTTGGGGGTTTCGTAGCACCACTTGACGCTTTACCTCATTCTTTCCTTGTTTTTGGTTGTATGCCAATATCTCTCACACAGGTCAGAACCTTGGTTGATTGAAGGAAAGTTATTTACCTATTTACTAAGAATTTGGGGTTGTTGTTTTTTTTGTTTATTGATTATCCCGATTTTATGACCGAAATTGTGATCAACCATATACCCCCCAATCACCGATGTATTCTTTGCTAGCATTTCGAGGTCCAAAATTGGGGGTGTGCTTCTCTAGATTTTCCTTCTTATAGAT------GGAGTCGTTCAGCGTTAGATAGCGATATTTACTATCTTACAGGTTTGTATCTTCTGCTGGTCCTCCGCGATACCTTACCTCTAGCAGTGTAGGTATTATTATCAGTATCTTTATCATCGTTTATGTTTTCCTTTATTGGTGCTATTTCTTTTGACGAACTCTAGTTGACAATCGCTGGCCAAAAGTGGCCATGGATCTCACCTTAGGAGCCCAATTTTTCAAACATTGCCCA--AGGA----TCTGGATTTTATATCCTTATACTTATTTAGGTTCTGTGTACCCTTATTCCAAACTTTG----GATCAAAATCCTTTATCTATTTATTCAGGTAGGTTAGCTTATTTACGGTCCTCCAATCAATATCGCCTGGAGCTCGGCATACCTTCATTCTTGATTGTTTTCACGCATCTAGATGCGTGTTTAATCTTAGATAATTATGCACAACAAG--GTCCAAATATTCATAT--GTGTGGATTTGTTGACTCCTATGCGATGGGCGACCCTTGATTCCGAAGGATCGATTGTAGGGTTAAGTGATAGGGGAGCGTAGTTTCTTATTAGGAAGTTTTCTACATTCTCATATATAGATTTTAACTG--GGTAAACCTTGTGTTTACTGATAGATTC--------ATGTATCGGCAGGCCCCGATCGTAGAAATGATATTTGGTGGATTTCATCCTTATTTATTTATAGTTACATTTATATTTGCATATTTGC----------------TTGTACTTGTTTGTTCCAAGCCAATTACCTGAAGACCCTAGATGGGGTATCCTTAGATAGACCTTCACCAATCATTTAGTGTAGGTGTTTCTATTTGAGTGATTGTGCACTTCCGTGTACTC----AGCTTTGAAAGATGTTTCAT--TGCTTTATCCCTATCTACTTGGTTCTTTGACTCATAGGAGTTGATGAAATATTCTCCTATTCCTGATTAGTGTGTGGTTAGTATATCAATGCCTTCGA--AGTTGAACCTCTAGTCGAGTTTAGGTATGTTGGAAGGTGTGGCTTCTTCTATAGCTACTAGGTGGGAGACGGTTAGGGGTGAATATATGTCTGAAATTAGCTACTTTTCTATGTTTACATAGTCCTGTATGCTAAGAATATAATGCGGCAAACTGGGGGATTTAGTTCATGTTTTTATGTAGATTTCTCTTCCTAGGGTCATTTTGTCTTATGGTGGAGGTTGGATATAGTTGTTTATTTTTAAAAAGTGAGTCCAAATCGGGTATACTTCTATAACGTTGGGATCTTATATATTGTCTCTATTAGGTTGGGAT--TGGA----------------------GTTATGTTTGATTTACTCTTTATTATGGGGGCTCAAAGGCCATTCCTTTCAGTGTTCCGAGCATGCGCCCACCCGTAGAGTTGTCATCTCACTTGTACGCGGATTAGTTTGCCGTAGGAG--ATATGAAGTTTAAT--------CTACTATTTCTCATTCATATATTCGGGTTCGCCTTGAGAGGATTAAAAGTGTGCTATCACCTTCCAATAGTTATCTTTTATTTCGAGGAGGTGAGGGCTTATCAATTTACCATCGTTTAAAGCTTTTTTCTGCTTCATGCGGCTCCTTCTTCGGGGTATCAAGAGT--TT------CTATTTAGTTAAAGTCATATAACTATATTTCTACGCGTATTTACAGGGTATCTTCGATATTATTCTTCAGAA--------------ATAAATACCAAA--------ATGCGTAGAGTTTTAGAGCCCTAACTAGCAATCTAAGTCT------ATTCGCTGATTCTCAGCCGGTATTTCAACT----AGAGCA--AGGTAATTGAGGAAATATATCTTTATATCTTTGGATGTGCATTTTTACTT--CTATTTGTGGTC--GACTTTCTAACTTTTCCTGCATACGTTTACGTGGCTCTATCTCTAATCTATCTCTGTCTGTTCTTTTGTTGGATTTGGGGTTATGTTCCTTCATGCTTATGTACCC--AA--ATAATCTCAAGATGTCAGAGTTAG--------------TTATAGGCTTTGATTTACTCGTATCTTCGCAT----------------ATTGAAGTGATTAAGATGTCGATTTATCACGA--AGGTAGGA------------ACTAAATCCT--CTATGACTAAGTTAACGCGTGTATCTTTAT----TAATTTATCTCCGTATGAGTTTACTCCCTAAGCGGCGGTCATACATTA-----AAGTCAAA-TTAATTGGTTAAAGCTAATAAATAATTACTATTAAGTACAACAACTCTACTTATTATTGTAAGAGTAAAAT---CTTTATAAATAAGATCTTACGAGGCGCTAGC-----------ATAAAGACTAGGATTAGATACCCTATTATGGGCGTGAGTATGAAAGTTTATTAAGTTAATAGCCTTAATTAAAGCA---------------AAACCTATTTACTA-TGGCGGCTGTT----AGTCTCACCAGAGTCGTATGTCCT--TAA-ACGAAACTGCGCGAT-TATCTTACCTTTTAACTTTTGCTTATATAAAATAATTACATTATAGCAATCTGGCACGTCGCTGTACTAAAAGTGAAGATT-----TTAAATAACTCATCTTC------------------------TAATTACTTATTAGCA-------------TTTATTAATAATGTA---CAAGTCAAGACGCCGTTT----TAAGAGGCTTAAGATG---TACGATTTA-----ATTGATTTG---------ATAGAATTTATAAGAATGAAAACTTTCTTTATTAAAAT-AATTTGGAAGTAAA-ATAGGGTTATTATTCTTATTTGAAATGAGCTACTAATAAGTGTACATATCGCCCGTCATTCCAATGGACAAGTCGTAACAAA-GTTGATTTACTGGAAAGTGGTACCTTTTGCATCAGGGGTTAAGAAATAA--GATTATTAAATTAATAA------CCTCCCGAAAG-AAGC-AGGATCTTAAACTTTATTGTTGGTTGTTGTTTCATTAACATTAA-TAATAATATTTAAGAAGCTAAAAGTTATAACGACTCTTCTGATCACCTGGTAACTAGGCAAAC-ATTT--AGCCGGACTGTTTAATAAAAACATTTCCTCTTGCATAAA---AGGG-AGGTAAAGTCTGCTCGGTG-----TAATTGATTAACAGCTGCAGTAACT-TGACTGTACAAAGGTAGCGTAATCACTTGTCTTTTAATTGAAGACTAGAATGAACGGCTTAACCAAGCTAATTCTGTCTCTTTTTAACCTTAAG---AAATTTAAGATTTAAGTTAAAACGCTTAAATTATTTAGAGGGACGAGAAGACCCTGAAGATCTTTTTTATTTGGGATGAATGT--------AATAAACATTATG----GTTTACTAAGACTTA---AACGTCTTTATTCTGACCCGTTTAAACGAGAATCTGTTTAAGTTACCTCAGGGATAACAGCG--CAATGTCTTTTATTA-GACCTTATAAAA-ATAGAAGTTTGCGACCTCGATGTTGAATTGAGTTAAACTTTAAGTAGAAGAGTACTTA--AGAGTAG-GACTGTTCGTCTTTTAATAGCTCACATGATTTGAGTTTAGACCGACGTGAGTCAGGTCAGATTCTATCTTCTAATATTAAGCA---ATCTT-TTTTGTACGAAAGGAC

Heterodoxus_macropus CTTCATTTGACCTGTCAGATTTAAAT--TTAATT----AATGATGTATAG--------------TTTGTTTTATCCTTAATCTTTGAAGTCCAGATTTTTACTTTTGATAATAAAACTATAATCATTTACAA------------------AAGTAATTCAATTCAGATTTTTTTATTTGTTTAAATTTGGATTTTGTTACTTTCGTACAGCATTGTATAATTTCTTGGTTTCATTGGTGGACTTTTTAAGTCATTAAA--TTAGGATTTTGCCATTACCCATGGTGCCATGTTTGTCCTTATGTGTATGATTATAGATATATCGCCATACTTTCTTCGCTATGCAAATTTGCGGCAATATTTTCTTATAGACGGGTAGTTATCTTCTTTTATCCTCGGATTTTTTTCTGGTTTATTTTGAATGGGTGCATATCAGCTAGTTTTCATCTCTTCTTTATGGAATTTCCTACTTGATTGATTTATTTCTTTTCGTTTATTTTTTTAT--AATTTTTTTCAATTATTCAACGAAAAAGTATGAAAA----CATAAATTGAAAAATTGATTTATCTCAACAAAAAATGGATTTTAATATCTGGAGTGTCGGCTTTGGTTAGTTAGATATATCGTTGATTTCGATCGAATTATTTTAACCCAATTAAAGTGTGTACTCCAGCTTTTATATTTTTTTATATCCTTATATGGGGTTGCAATGTTGTCCATATAAGGAGCCGAATTCTTCCCGATAAAAATAGTTTGTTTTCCCCTCTTATTTATTTTGAGATATCTGAGGGGTCGGACGGTGACGTTACCCCTTTCTCTTACGGCAGCGGATTCGTGAATTTATTTTCCTCACTGCGGATAGTCATATGGGCATAATTATACACATTTAAAT--GTTTTTAAAATTTCATATAGTTTTAATGTCGTTTATACGCTTTTTTCTTTTCTTCCGTTTGCGGGCATACATTTTTTTGACGAATTAATCAGTTTTGACCATGGGGGGGACCATTTTACACATTTTTGTTTTGGCACCGAGTTAATTTATTTCCGGTTGGTTATTCCAATATGTCAGAAGGGAATGGAACTTGGGTTTGGATATTAGCATTTTCATGGATCTGGTTATGTTGGCCACAATTTACATGGATGAGTGAACCGGCTATTACTCGCACATATATGCATCCACGGATAAATTTAGTGTTTCACTTTTGGAGAAATAATTAATCTCGATTTGAGATGGTTGTTTTTTTACGTGGGGTTACGGGTGTTTGCAATCTCATGAATGTCTCAGAACTATAGTGTGCCATTCATAGTTTTCATGGGCGTTTGCGTTTTCGCTTACCATGTTCCTTTTTTGGGTAAATAGAAGCTTATATCTCATTTGATACTTTTGGGTAATTACTTTTCCCACATTTTGGCTAGGGATCCCGCGTAATTGTACCGATTTATATCTGAATTTATCAGATGGTCATATACTCGTAGTTTTATTTGTTTATATTTTAAGTTTTGAAAAAAATTTTTTTATCTTAGATAATCATGATGATCTGGACCCCCTCGCCATCTTAAGAAGCCGTTTATGATTAACTTCGAGGTGTCTTATATGAAAATGTGCTTCAGATTACTTATATCTTTTTATACACGTGTCTATATTTATTCATATATACAATTGT------AACGTTTTATTAAAGAGTTTGATTATTGACGTATCCAGTTATTTTTATATGCTTCCTCTTAAATCTTATTGTGAGATTCTAACCGAGTACGTAAGTATGGAACATGTATGTCTACATATCGATTTTAAATGA------TTGATCTAATAAAATGGAGGCT----TCGA--TTAATATTGAGTGAAACGACGTCTCCGTGAACAAATCGATATATACTCTCGAGTATCATCTGACATCCAGTTGGGTAATTGAGCAACCGGCGTTAACATTAAATTTGGAACGTTGGTTTTTTGGCATGTCGAATTGGGATTTCATCTTATCCATTGGTGAATGTAACCGATGTTTTAATGTTTAAAAAGGTTTTTTTTCAATGTGAGAAGCCTGCCTTTTCTTCTTAGGTTTTTAAATTTAGGCTTGTTATTAA--TTCAATTTATTAATTTCTAGAAATTTAGATTTATTTTAATTGATCGGAATATTCGAAGACATCAGGATCAACTTAAGTCAAAGGATAAATGGATGTTTTTATACTCGAGTATTTTTTTTCTTTTTGAGTTGGTATAATGTAGCAGATAATCT--AGAATGCCCTTTGGATATAGTTAACCTCACGTCCTTTTGGACATATTTTTAGTCGGGTTCGTACTGTGCAAAGACTATTTAGGAGGAACTAGAGATAAAATCTTTTATACGTATCTGGATGTTTGCGCCTCAATTGGATATTATTCACTTACATAGGAGGGTTAGGTCTTTTTAATATACGGTTCAGGTTCAGTATGTGGACATTTTTTTATATTTTTCGTTAAAATACATTTCAGCACACATTGGTTCAGCGCGCTGTATGCATTGTGAGTGTTGATTTTTTAATATTTTATGGGTAATTGAATTGGAGTTTTGGCTTGCTTTATCAATGGTCGGTTTTTTTCCTCATAAATCAAGTGATTGCTTAGAGGTATTAATATAAGAGTAACAGGTGATTTCGGTATCAGCAAGGGTACATATTTATTTATTAATCAATGCCGGGCTTATAAATCTAAACTAC--TTGTTGTTGTGGATTTATCTCTTTACATGGACGCTTTTGGTAGTCTCCTGGGCAATTCTTTGGGGCATGTATACAATTATAGACATCCTATTGGGTACTTGTGATGGTTGGGGGTTTCGTAGGACCACTTACCGTTTTTCTTCATTATTTCCTTGTATCTGGGCTCGCTTCAATATTTTTCAAATATTAGTCAACCCTGGTT--CCAAACGAATATTCTTCACCTTTTACGTAAGAATTTGGGTGTTTTTTTTTAGTTTTTTTTTCTTACGACCTAAATTATGACCGAAATTATTTGCAATCATGTACCCGTCAATCACCGATGTATTTTTTGCTATCATTTCGGCGTCCAAAATTGGGGGTATGGTTTTATTCATCTGTTTGCTTTTTTTTTCAAAAAGAATCCAGAAGGTTATA------AATCTTTGTGGTCATTACATTTATTTTTACTGACGGAGTTCCGTGATCCCTTTTGAATGGCATGTTTCGTATTATTTTAAATTTTTTTTTCACATTATCTGTATATATCTCTACGTGCTTTTACTTTTGACGAAATTTGGTAATCATTCGAAGGCCAAAAGTTTTTAAGGGTTTCACCATGTGAGCATAATTATACAAGAGATCCCAT--ATTAAGAAATTTTTTATAATTCCCATTTAGTTATATAGATATATTGATATCTCCTTCATTATATTTAA----TGGTAAAGTTTTATTTTTATTTCTGGATGGGTTAAGATTTTTTCGGTGTCTCAATCAATAGCTATTGGAGCTCGGCGTAGCATCATTCTAGAATTTATAGATTTTTATGCTTATATACACAAGGATAGATTATAATTTAATTGACC------TTGTTTTTTTTT--CCTTTTATGCTATTTTATGGTTGCGATTAACGTCCCTTGATTTCGAGGGAAGGATTGTGCGGTAACGTGATAGGGGATATTAACATATTTTTAGGAAAATATATATTTTTTGTAATGGTCTTTTTTTT----TAATAAAGACATAGATATTTTCATATATATTATTGTTGTTATCGGGATCTCCCGATCGTAGACATTATATTTTGTGAAATATTTCCTTATGTATTTGTAACTTTCTAGAGCTATATTTGTTTTA----------------TACTAGGTTTTTATGAAAAAAAATGTGAGAGGAAAAGATTGATGGGTTCGGCGAAATTTCCGTTCCTTTCATCATTTTAGATGCTTGTTTCTATTTGAGTGATTATATATTTCCTAATTTAATTAACAATTAAT--------------TTAGATATATATTTTTTATTGGACCTTTGATGATGAGGAGTTGATGTAATATATACTTTTAGTTGATTATGGTTGTAATTATATATAATTTTTTCC--------AAATAATTATTTATATGTACTTTTATTTGAAGGTTTATTTATCTTAACTGTTATCGGATTGGATGAAGTTATGATGAATTAATTATTTGAAATTTCATATTTTTTTATGTTTATATTCAAAAGTTTAATATCTATTTATTGTGGACGTTGACTTCGTTATGGATTTATTTTTATGTTCATTTTGTTATATTCTAGAATTTACTTATGTGGTGGAATTTGGGTTCTCTTTTTTATTTTATAAATCTAAATCAAAAAGAGTTATACTAATAGAACGTTGGGAGGTTTTATTTGCATGTTTGCAGCCTTTTAG--GATTTTTTTTAAGCCA------------------TATTCTCCATTTGTTTTGACAGATACAAAGGCCATTCCTTTCAGTGCTCCGAGCATGCGCCCACCCGTTCACTTGTCATCTCACCTGTACGCGGTTTATTTTTTCGTTCAGACTTGATAAAAATTAGCTTAATT--TTTTTTATTCTTTTACATACTTGCAGAGGCGCTTATGATAGATTAAAAGTATGCCTTCACTTAGCAATAGTTATTTTTAGTTAGTTAATTACACTTGCTTTTCAATGTATCAGCTTTTAAGCGCACTTTGCAATTCTTTCGGTTTTTTCTTCGGTTTATCAAGATT--ATTAAATCTTTTGCTAAAAATTAATATATTATATGTTTCTATTCATGTTTACATTTTATGTTCGATTGTTATATATAATT--------------TTAAATACTTTA------TTAATTAAGAATAAATTTTTCCGTTTGATTATTTCTTCATGTTCGGTCGTTTATTG------------------TATT----TGGATCGTATATCCTCAATTGATTATAATATTTTTTATATATTTATTCATTTTCAGATTCTTTTTACTAGAACTAGAAATATAGTTATTTGTCTATAAATATTCGTTCGGTTATATATTATCTTTCTCTTGAGGATATTTGTATGTTTTTACGGCTTTATTTATTCTATTGTAGGTACCC--GA--AACCAATTGTAATTTTTAAAGAAAGCCATCAAAATTTATTATAATGATCTTTTTTCATTTTTTTATTTTT------------GCTTTATGGATTAAAGCATCTTTGTTGAGTAATTAG--AAGAAATT------------AATTTCTTTT--TCCCTTAACAATTCATTAATGTAAATTTATTTTTCTTTTTATGTTGATTAATCGTTACTAACAAAAGGGCGGTTAAACATTTTCTGTGAGTTAATTAAAAT-------------------------------------------AATGAAGATTACAACAAATAATTTAAAATTATTGGTAAAATGATTTAAGAAAACTAT----------AATCTAAACTAGGATTAGATACCCTATTATT----------------------------GTAGAATTAATCA-----AATCTTCAAC-----AAAATTAAATAACA-TGACAGTATAAATATTTACAGATTAGAGAAATATGTGATA-TAA-ATGATAATCCCCAAC-AATCTTAC---------------------------TTAGTTTAT---AAATTGGTATACCGCCGTCTTAATATTAAATTTAAATATTTAAAGAATTAAGGGAT------------------------TTAGAATTA--------------------TAATTTTTTTTTATAAGTCAGGTTA-GGTACTGTTGACAACTAAGGAATTCATGTAT-------TGCA-----ATTATTATAAAAAATTATTTTGGTATAATTTTTTTTAAAATAA-----ATGAAATTGAATTTAATAGTAAA-TTTTATTTAATATATAAAATTGAATATAGTA-ATATTTTATGTACAAATCGCCCGTCAATCTTTTAGATAAGTCGAAACACA-GTAAATTTACTGGAAAGTGGTACTTTTTGCATCACGGTTTAT--AGAATAG-TAATATAAATTATATT------ACTCCCGAATT--AATAATGATCTATTTTTTTAAAAATCTAAAT-GTTTCAAAA-TTTTTTAAAATAAAAAAATTGAAG-CTATAAGTAATACGAATATTAAGAT-ATCTGGTAACTAGGCAAATATAA---AATCTGAATGTTTATCAAAAACATTTTCTTTTTATTA----TATAAAAGATAAGTTCTGTTCACTGAC---TATATGTTAAAGAACCGCAGGA-----AACTGTGCTAAGGTAGCAAAATAAATTGTATATTAATTGTATTCCAGAATGAATGAATTAACAAGATTTTTACTTTCTTAAAAATTACTATTT----AATTTTGATTTTAAGTTAAAATTCTTAAATTAAGATGTAAGACGAGAAGACCCTGTAGATCTTTTTTGGTTGGGGAAACAATTAAAGC-GAAAAATCTTTGATTTTCTAA-ATCTTAAATTTAAGGA----ATTATAATCTTTACTTATTAAGATTA-A-TATAAAGTTACCTCAGGGATAACAGCG--TTATTAAATTTTTGA-GATCTTATTAG-AAATTTAGATTGCGACCTCGATGTTGAATTAAG-ATTACATTTATATGCAGAAAGATAA--ATAGTTA-GTCTGTTCGACTATTTAAATCTTACATGATTTGAGTTAAGACCGACGTAAGTCAGGTCAGTTTCTATCTACAT-CATTTTTTTCTTTTT-----TAGTACGAAAGGAC

Ibidoecus_bisignatus TTTCATTTGACCTGGTAGTAACAG----TTCATT----AATGTTATTCTT--------------AGTGATATGTATACGTAATTATAATTGATA--GTAATTTATATTGTGTTCAATTTTATTCTCTCAAACTTTTAAAGAGTA----AAGTGTTCGTAAATGTTCGTTTACATATTTATTGAACATTAGATGTCCTTGTTTGGCCACAGCATTTCTTAATCGCGTGCTTTCAGTGTTGCGGATATACAT--CTTTATTCTTAAGATCGTTCCATTGTCCTTGGAGCCATTTTTACCCTTTTTTATGTGAGTATAGTGTTATCGCCGTGCTTAGGTCGTTATTCAAATATGCGGCAATATATGTTTTTAGAACTATTGAGTTAATCTA------TATTATCCATGATCTTATTTTTTTGATTTGATTCATGTCAGCTAGTTTTCAGCTTTGCTTTATAAATTTTCCTGACTGTTTTATTTTTTTGCCTTGGTATGTTTTGCTTCTTATAATTTTGTCTATGAGAAGACTTAAAAACCCAAAA----GAAACAAACGGAGCTTACTTTATCACAACAAAGAATGGATCTTATTATTTGGATTGTCGGTTTTGGTAAGATAGCTATATCGATGACTAGCAATACAATAATAAGAGGCAATTAAAGTATGTACTCCAGCTTTTATATTTTTATATATCCATATATGGGGTTGCAATGTTGTCCTTATATGGTCCCGAATGCTTCCCGATAAAAATAGTTTGTTCTATCCTCTTTTTTTTTTATAGATTTATGGGAGGACGGACGGTGACGTTACCCCTTTCAG------CAGCTCATTCGTGAATTCATTTTCTTCATTGCGGTTAGTCATTTGGGCATAATTATTGACATATAAATTGTTTC----TCATTTTTCTCCTTTTTGTGTCATTTATACGCTTTTTTTTCTTCTTCCGTCTGCGGGCATACATTTCTCTGACGAAATAATGTCTTTTGACCATGGGGGGGACCATTTTACACATTTTTGTTTTGGCACCGAGTTAATTTATCTCCGGTTGGCTATTCCAATATTGGAGAAGGGAAAAGAGTTTGGTCTTGGATATTAGCATTTTCATGGATTTGGTTGTGTTGGCCACAATTTACGTGGATGAGTGAAGCGGCTATTACGGGCACATATATGCGTCCACGGATAAGTTTAGTGATTCACTTTTGCAGAAATAATGTCGTTCTCTTTGAGTTGGTTGTTTCTTTACATGGGGCTACGGGTATCTGCAATCTCATGAATGCCTCAGAACTATAGTGTGCCATTCATAGTTTTCATGGGCATGTGCTTATGCAGTTTTCATGTTCCTTATTTGGGTTACTAATCAATTTTAAATCATTTTGTACTTATAGGTAAATATTTTTCCCACATTTTGGTTGCGGATCCCGCGTAATGATACCGAATTTAGTCTGAAGTATTCTCTTGGTCACTTTCATATAGTTTTATATATTTTTATTTGAAGCTATTCAACGCTGTGTTTAGTGAAATCCATTCATGATGGTAAGGTTCCCCAGAACATGAAGAATGTCCCATTACACCTTGTTCAGAAGAATCCCTTATATCAATAACACTCAGACAATATGTGTATATATATATTCATGTATTAGTTTTTACATGTATAACCTGTC------AACGTTTTTTGGAGGAGTTTGATTATTGACTTGCCCAGATGTTTGCATTTGCATCCTCCTCAATTTTATTATGAGATTAA--CCATATAGATAATCATGGCACATGTATGTCTAGATAGGGATTTGAGATGA------TTGATCTAATATATGACAGATTGATTGGAT--ATCGTTTTGAGTGAAACGACGTATCCGTGGATGAATCGATCTATACTCACGAGTATCATCTGACATCCACCTGGGTAAATGAGGGTCCGGCGTTAACAATTATTTCAGAAATTGGGTTATTAGGCATGTCGAATTGGGAGTTCATCTTATCCATTGTTGAGTTTTCGATCCGTTATTCTGTTAAAATAGGTTCACCTTCAATGTTCATAGCCTGCCATTTTGTCTTTCATATTCTTGTATAATCTTTATAATAAAATTTTACTTGATTCTTTGATCTTTTTCTTATTTGTATTTTGTGTGCGGAGTATCGGAAGACTTCAGGTTCAATAAAAGTTGTTGGTTTAATGGGTTCATTTATATTCGAGTATTTTTTTTCTTTTTTGGTATTTTTCAGTTGTCCGAGTGAATGGTGTCTGCCCCGTGGGTCATCTTAGTTATGAGTCCTTTTAAACATATCTCTTCAGGGATTCATACTGTCCACATCTTTT----GAAAAATTACAATGTTTTGGATATTTACGTATTTGGTTATTTACTTTTCATTATGATATTGATGTCTTTCATGCGAAGGTTAGGTCCTTTTAATTCACGGTTCAGGATCAGTATGTGGACTTTTATATGTTCTTATCGATATAATACATTTCATCACACATTGGTTGATTTCATTGTATGCATTGTGAGTGTTGTTTTTTTTTTAGGTTATATTTTATTGAATTGGTCTTTTGGATTGTTATGTCAATTTTCGGTTTTCTTCATCATAAAACTCATGAGAGCTTAAAGGTTTTCACTGAAGAGTAATTGGTGTTATCGTAATCAGCAAGGGCTCATTTTTATCTGTTATGCAATGGCGGGTTTATTGGAGTTAAATAC--TTACTGTTTCGGGTATATCTTTTTTTATGGACTCTTTTGGTAGTTTCCTGGGCAATTCTTTGGGGCACGTATACAATTGTAGACATCCTAGTGGGACATTGTTATGTTTGGGGGTTTCGTAGGACCACCTAACGTTTTTCATCATTATTTCCTTGTTTATATGTGTTTGTCAATTTTCCTCAAAAGGGAGAGAACCTTGGATTCCCAATGTTAAATTCTTCACCTATTTGAAAAGAGTTTGGTTGTGTGTTTATATTTACGTACTTATTTCTCCGAGTTTATGACCGAAATTTCGTGCAACCATTCACCCGCCAATCACCGATGTATTTTTTGCTAGCATCTCGTCATCCACAATTGGGGGTGTGCTTGTTTTCATGTATTTTTATATCCTTATAGAGGGAAAAAAAGCTAATTTA----CAAAATATGTTTATCAGTTCAATTCTTTTTACTGTTGGGCATCCGTGATTCCTTTTATATAGAAATTTTCTCATTATTATTTATATTTTTCTCATTTTTTATATGTGGTTTTTTTCGTGCTTTTTCTTTTGACGAAATTTAGGTATCATTCGAAGGCCAAAAGTGGTTATGGTTTTCACCTTTCGAGCATAATTTTTTAAAGAAGAATCC----AATTAGAAATTTTATATTGCCCATGTTTTTATTTTCATTTATTGATAGATCCAGAATGAATTTTAA----TTTCTCAGTTATTTGTATTTCTTAGGATCCATTAAGATATTTATAGTGATTCAATCAATATCAAATGGTCATCGTCGTGCCATCATTCTAGAATATTTTCTCTGTTTTTTTTATATATGTTATCTCTCATAATTTTTATTTACATCTAGTTGCTTTTACCTGTT--CCATTTTTATATTTATTCATTTGCGAAGAACGTCCCTTGACTACGAGGGAAGGATTGTTCGGATTTGTGATTGGGGGTTGTAATTTATTTTTGGGAAATTTATTTTTTTCTCTTTTATTCTTAGATTTGGAATCTTATGTTTAATTGTATATACATTTAT--------GTTGATCGGGACGTCCCGATCGTAGAAAATATGATTTGTGATAGATATCCATTGATTCTTATTCATGTATACGTTTTTAAATGTCTTG----------------TTATTCATATTTTTAAAAGAATAA--CAGAAGAAGAAGTTGATGGGATGAACTTTTAATTAATCTTTATGTTCATTTTTTATGGGTTTTTTTGTTTGAATGAATATATTGATCCATATTTTT----AATTGAATATAAATTTTTTA--TGAGTTATATATGTTTATGTGGTATATTGATTGCATGGACTTAATGAAATATACATTTATTCTTGAATTT--GTTGACATTTTTTTATATTAATGAAATCATATTCATTCTTTTCTTTTTTCTGATTTATTGGAGGGTGTGGTTACATTTTCAATTTAAAAATAAGTCTTAACAGTTCTTTCTTTCATATGTGAAATATCTTATTTATATATGTACATATAGACATGTATATTATCATTATAATATGAGAAAATAAAAAATTTTTTTCATTTTTTTATCTTCATATATCTTCTTTCGCAAATTTTGTTATGTGGTGGAGGTTGGCTTCTCTTATCTATATTATTCAAATGAAAGTTAAAGTCATACACTTATTGAACGTTGGGATTTTATCTATAGATGCTTATGTAATTTTTC----AATTTGTTTCTTTTAATC------AATTTTAGTTTTTTTGTTTTATATGTTGGCATACAAAGGCCAGTCCTTTCGTTGCTCCTTGCATGCGCCCACCCGTTCTCTTGTCATCTCACTTATACGCGGGTTTTTTGATCGTTAGAG--ATCTATGAGTCATTCTTTAT--TTTCTAATTCTCTTACTTATATTCGGCTTCGCATTAGATAGATTAAAAATATGCCTTCACCTTCCAATGCTTATTTTTTTTTAGATAAAGTTGATCTCATATCATTATACCAGCATTTAATCTCTTATCCATTTAGATATGGATTTTTTTTCGGTTTATCAAGATT--ATGGAT--ACATGTTGTAAAACAAAATGTAATGGTTTATTATGCGTGTTTACTGATTATCACCGTTTTTAATTTATAAAAAAATAA--------AGAAATTTTAAA----------GAGAAACATCATTATAATTTTAGTAGAGAGATTCATTTTTGGGGTTCTTTAA------TTATACTCTTTGAATT----CAAAGAGTATTTATAATTTTTTTGTTATCTTTTTATTTATGGATTTATTTTCGTTTAT--TTTTTTATTCTC--TCTCGTATATTTTTTTATATATAGTTTTATTTGGGTTTATTGTCGTTCTTAATTTTTTTCTTATTTTTTTATACTTGGGGTTTTATTTTTTCTTATCCATTT------------GATTAATTAGATAAAATC------------------TTATAATTGATTTTTTTATTTTATATATTTGT----------------TGATAAATAATCTTGATTGTAATTTATTAATT--GAGAATAT------------AATATAATAA----------ATGATTACGGTTTCTTTTTTATTTATATTTATATTTTTATTTGATCATGTAGACTCGAGGGCGGTCATACTTT------AAAGAAATTTAAGTTTTCTTTCTATT-------------------TATTTTTATATAAATTTATTGGTTAAAAATTAATA---GTTAATCGGTAAAATAAAATAAAAAACTTAT---------ATATGTAAACTAGGATTAGATACCCTATTATACTTATGAATAAAA--------------------AATAGAAAGAGTAAATTTTATTTAATTGAAAATTATTTGCA--TGGCGGCT--TTAAAAACCGAATTAGAGAAATATGTTTG--TAA-TAGAATCTACGCTAA-AACCTTTC---------------------------TATATAT-----TTTCTTACACACCGCCGT--CGGAAGTATATATT------TAAATTTATAAACTTT------------------------TATACA-----------------------TTATTTTATATAATAAGTCAGGTCAACGTGTAGGTAATGATGT-AGTATTGATGTATTTCTATATATA-----TTTATTA-------------TGAATTAAAATTTGTAATAATTT-----ATGAAACAGAATTTAATTGTAA--AAAAAATAAAAATATTTTCTTGAAATTGGCT-CTTTAAAGTGTACAAATTGCCCGTCAATCTAAAGGATAAGTCGAAACAAA-GTTGATTTACTGGAAAGTGGTACCTTTTGTATCAGGGCTTAAGGAATAAAT--TTGATTA-TATCTT-------TTTCCCGAAAA--AA-AAGGATCTTGTAAAATTA-AAGGTTAAA-GTTAAA-ATTTTATCTTAAAATTTTTACAAGATG-TGATAAATCATTCGGCTTTTTTTAT-ATCTGGTAACTAGACAAAAAATT---AACCCACCTGTTTATTAAAAACATGTCCTTTAGAAA-AA-AAATTAAAGGTCTGACCTGCCCCCTGCTT--AAACAAGTTAATGGCTGCGGTATTTGTGACTGTACAAAGGTAGCATAATAAATCGTCCTTTAATTAAGGTCTAGAATGAATGGTTTGATGAGGTTAAATTTGTATCAATTGTTATAAATT----AATTTTTTCTGTTAGTAAAAAAACTGACATTTTTCGAAGGGACGAGAAGACCCTTTAGATCTTATTTTCTTGGGATGAGACTAATA----AAATAAACTATAGTAAAAAAAAAGATAAATAAATCAA-AAAAGAAAGAAATAATTTTATATTTATAAAA-GAAAAAGTTACCTAAGGGATAACAGCA--TAATA-TTAATAAGAAGATCTTACTCT-TATTAATGTTTATGACCTCGATGTTGAATTAAA-ATTTCTTCAAATAGAAAAAGATTTG--ATAGTTA-GCCTGTTCGGCTATTAAAATTTTACATGATTTGAGTTTAGACCGACGTGAGTCAGGTCAGATTCTATCTTTCG-ATTTG-GTATTCTTCT---TTTGTACGAAAGGAT

Lepidopsocid_sp TTTCTCTTGACCTTACAAATTTAA----TTCCTT----AATGCTAGTCTT--------------TTTTATATTTATCCTCTTTATGATTTCCTCCGATCATTATTGAATTATATAAACTTCAAAGATTAAACCTTTAAAATCTC--AATTGAAGACATATTTATTCATTTAGTTGTTTATAAAATTTTGGTTTTCCTAATTTACAGACAGCATTGTTTACTTACCTTCCTCCTTTGTTAGTTATATTAGG--TGTTAAAAACAACAATTTGCCATTGTCCAAGGACCCGGATCTATCCTTATGTTGATGAACATAGAAATATCGCCGGACTTGCGTCGTTACGCAAATATGCGGCATTATTTACTTTTGGAAACGGTCAAATACATTTTTGTACTTTTATATATCAATGCTTTTATTTGAATGCGTGCTTATCATCTAGTATGCATTTATACCTTATCAGATATCCATCCTGTTTCTTTTTTCTTTCTTTACTTTTATACAATG--ATAATATT--------TATTCACCAAATTTTTTCAA----AATATTAAATAAAAAAATATTTTCACAACAAAGAATGGACTTTATTCTTTGGATTGGCGGATGTGGACAGATAGATTTATCGTTGATTGGCACCGGTTTTTTGAGAGACAATTAAAGTATGTACGCCAGCTTATATATTTTTATATATCCATATATGGGGTTGGAATGTTATCCTTATTTAGGCCCGAATGCTTCCCGATAAAAATAGTTTGTTTTCCCCTCTTACTTTTTTATAGAGATACAAGTGGGCGGACGGTGACGTTACCCCTTTCGCGCGTGCCAGCGGGCTCGTGATTGCATTTTCTTCATTGCGGATAGTCATTTGGGCGTAATTATTCACATATAAATCGTCAAGGTTACTTGACGTTCCCTTTGTTGTCGTTTTTACGCATTTTTTTTTTCTTCCGTTTGCGGGCATACATTTTTACGACGAATTAAACTCTTTTGACCGCGGGGGGGACCATCTTACACATTTTTGTTTTGGCACCGAGTTAATTTATTTCCGGTTGGATATTCCAGTATAGCAGAAGGGAAAAGAACTTGGGTTTGGATATTAGCATATGCATGGTTTTGGTTGTATTGGCCACAATTTACGTGGATGAGTGAACCGGCTATTACTCGCACATATATGCATCCACGGATAAATTTAGTGTTGCACTTCAGGTCAAATTTTTTCCCTCTCTTTGTCTTGGTTGTTTTTTTACATGGGGTTACGGGTATTTGCAATCTCATGAATGCCTCAGAACTATAGTGTGCCATCCATAGTCTTCATGGGCGTTTGCATATGCGGTTATCATGTTCCCTCTACGGTTACTTAAAAAATGTTAAATCATTATATATTTATGGGTAAATACTTTTCCCACATTTTGGCTATGGATCCCGCGTAAGGATACCGAATTAACTCTGAAATATTCTCTTGGTCACATTCTTATGGATATTTTTATTTATATTGGAAGTTATTCAACGAACCATTTTCATCAATTCTCTCATGATGTTCAAATACCCCTCGACATCTAAAGATTCCATATTAAAATAATTCAGAAGGCTCCCTTATGACACTATTTTTCAGACATCTTTTATATACATATACGTATGTTCTAATATGCTCTTTTTTAATCTTAC------AACGTTTTTTGAAACAACATGAATATTGACATATCCGGATGTTTATTTATGCTTCCTCTTCGCTCTTATTTTGAGAACAAATCCTCATACTTAAACATGGCACATGTATGAGTAGATATCGATTAAAAATGA------TTGATCTTATATCCTCAAGAAATTAATCGA--TTCGCTTTGAGTAAAACGACATTTCCTTAAACCAATCGATTTGTACGCGCGAGTTTCATCTGGCATCCTCTTGGGTAAATGAGCAACCGGCGTTAACAACTCTTAAATAACGCCGGTTTTTAGGCATGTCGAATTGGGGCGTCATCTTATCCATGTATGAAGGTCAAAAAAGTTATAATGCTAATTCATCAACACCTACATTGTGAGTAGCCTGCCTTACGGGCATGGACATATTTACTCGGGTGTAATGTTCA--ATTTAAATTTTTTTTTATGGATATATATTTTTACATTTCATGTGCGGAGTGTCGGAAGACTTCAGGAACATCATTCGTTCAAGGATCGTGGGATATTTTTATACTCGAGTTTTTTTATTCTTTTTGGCTTTTCAAGAGTTTCCCTCATGAATGGATATTGCCCCAAGGATCACCTTAACCTTCAATCCTTCTAAACGTATTTATTCTCGGATACATACTGGCCACATCCTTT----AAAAAAAAAGCAACATCAAGTTGTATACATATTTGGTTTATTACATCTCAGGATGATATGGAGCCCTTTCATGCGAGCATTAGGTCTCTTTTATGCACGGTTCAGGATCAGTATATGGACACTTATTTATATCTATCGCAATAAAACATTTCAATACACATTGGTTGAGCGCGCTGTATGCATTGTGAATGTTGTTTTCTTAATTCATTATGTGACTGTGAATTGGTCTTTTGGTTTGTTATATCAATTCTCGGCTTTTTGCATCATATCGCCAATGATTGCTTTCAGATATCAATTGCGGAGTAAAAGGTGATTTCGACATCAGCAAGGGCTCTTTTTTATTGTTTATTCAGTGGCGGGATTATAAGTCTAAATTCA--ATACTGTTATGGATTTATTTTTTTACATGCACGCTTGTGGTAGTTTCCTGGGCAATTCTTTGGGGCACGTATACAATTTTTCGCATCCTATTGGCAATTTGTCATGATTGGGGGTTGCGTGAAAGCACTTATCGTTTTACTTCATTATTTCCTTATATTTGCATTCATATCATTTTTTTTCACAACGGTCAAAACCTTGGATAAATAAATGAAAATCCTTCACCTTTTTCATAAGATTTTGGTAATATATCTCTATTTATTCTTAATTAGATCCTAATTTGGGACCGAAATTACCCGCAACCCTTCACCCGTCAATCACCGATGTATTTTTTGCTAGCATTTCGTCATCCAAAATTGGGGGTTTGCTTTTTTTCATTTATTTTAATTTCCTTTTAA--AAAATTCGTCACCATTTACCATAAAAATTTTTTGTCTTACACATTTATTTTTACTGGCGGGCAACCGTGAGACCTTATTTACAGCAATTTACGTTTTATTTCTTTTATATAAATAATTATTTTTTTATGGATCTGTGGGTGCTTTTACTTTTGACGAAGTTTGGTAATCAATCGAAGGCCAAAATTGGTTATGGTTTTCACCTTAGGAGCATAATTTTACAAGACAGTTTCC--AAATAGAATATACCTATATTTCCCGTTTTTTTTTTTTCTTATTCTGATATATCCTATTTGGTTTAAAA----TTAGTTGGTTTTTTTTATGTTGTTAGTTGGGTTAGGATATATAGGGTGAGTCTTAGTTTAGCTTTTGGAGCTCGTCGTGCCAACATTCTAGAGTAGTTTCTTATTTATAGATTTATTTATGGAATAAGTTATAGTTTATTTACAAATATTTATTTAATTTGGTT--CCTTTGTTATTGATGTTCATTTGCGAGTAACGACCCTTGATTGCGAGCGATCGATTGTTCGGTTAAATGATAGGATGGGGTTGCTTATTTTTGCGATATCAGATTTTTATAGATGTTTACTGTTTTTTGGGCGAATCTAGTTTTTTTTTTAACTTTTTGTTCTTTTTTATTGGTCGGGAGTTCCCGTTCGTAGAAATTATAATTTGTGAAAGTTTTTCTTTCCTTTATTTAGGTACTTACATTTCTTCATTTTTAA----------------TTATGCACTTCTTCTAAAACATGAGACAGAAAATTCCCTTGATGGGTTGACCATAATCTCCGATCCTTTCCTCGTTTTTTATACATATTTCTATTTGAGTGAATACTTATTTCCATATTTAATTAAAATCAAATTTTTTGATTTAT--ATTTGTTTATTTATTTATTTGGTTTTCAGATGAACAGGGCTTGATGTCATTTATATTTATAGTTGATTATAGTTATTTTTCTTTATTATTAGTTTTAATTAATTGAGGTATTTTATATTTTTACTTTGGTTGGAGGGTATGGTTTCTTCTGTGGATATCGTCCAGGAAGATTTCGTAAATCAATGTATTTTTGATGATTCATTTTTTTAGTTGTACTTATTCTGTTATTCAATAAGAATCTAATTTGGGAAAAATCAATTTTATTTTTATATATTTGTTTTCATATTTATATGTAGCCAAATATAGATTTTTGGTGGAGGTTGGTTGTTCTAATTTGTATTATACAAAGTAATCTAAAGCGGATTTACGTTTTCAACGATGGGAATATATTTATTGATGGTGCTTTAGTTGGAG--TGAATATATTTAATAAATATACACGAGATATTATAGTTATGGTGTTATATATGCGGATACAAAGGCCAATCCTTTCTCTGTTCCGCGCATGCGCCCACCCGTTCGCTTGTCATCTCACTTGTACGCGGGTTATTTTATCGTTTTCC--TTTTAAATAGAATTAGATAT--TTTTTTATTCGGTTACATTTATTCGGTTGGGCAATTGATTGATTAAAAATATGCTTTCACTTAGCATTGGTTATATGGAGTTTCATGGTTACAATTTGTTTTCATTTTAGCAGCTTTTAAGCTTTTGTTCAATTTCTTTGGGATCCTTTTTCGGTTTATCAAGATT--ATTTGA--TTATTCATAGGAATAATTATTCTATTTTTTTTGCTCACGGTTACATAGTATCTTCGTTTTAGTTTTATTTGGAATT----------AATTTTAGTTCA----TGATGAGAAAGATAATATATAATCATTTGGTTTTTTGGTCTTTTGGGGAGATTTATTG------------TTATTTCCTT----AGAAATATTTTTCCTTTTATAATTATGTTTTTTTATTTATGGACTTTTTCACTTTCAT--TTTTTTTTATAA--CACCTTTCGCGGTTATTTATATTCACATTTGTGCCTATACGCTAATCTCAACTTTGTTTCTAATTTACTTATCTATGGGGATTTATTTTTATTAATATAGTTTCCC--AA--CAAATTATATTCTCATCTTTTTATCCTT----------TTTTATATCCATATATAAATATGACCATATTT----------------------------ATGATTTCCAAAATAAGACA--ATAATGAA------------TTCCCATCAT--AATTTTAAACAATCAGATTTACATATAGATAATATTTTTTATATATATGTACAAATACTCAGTTAAGGGCGGTTATACATA------AGATTAAAGTAAATATTATTAATTTA-------------------TAAACTAAAAAATTTTTATTAAATTTATTTTTTAAT--TTTATTAGATAAAATGTTTTGAAAAATTGTA-----------TAATAAACTAGGATTAGATACCCTATTATT-----GTAATGGAAAAATTTTTAATTGAGTAGTAAAAAAAAAAATTAATTTTT--------AAACTTAAAGAATT-TGGCGGCATTTT---ATTCTGTTTAGAGGAATATGTTTTTATAATTTGATAATCCACAAA-AATCTTAC---------------------------TTAATTTAA---AGTTTTGTATATCTCCGT--CATAAGAATGTTTT-----ATAAGAAGTTTTTCTAT--------------------------------------------------ATATAATTTAATATAAAATGTCAGGTCAAGGTGCAGATTATGATTA-AGAA--AAAATGTATTACAATAAA-----ATTATT------------TTTGGTTTTAGTTTTGAAAAAATTT----AATTAAATTGGATTTAAAAGTAAATATTTAAATATTATTTTTTTTTGAAAAAGTT--TTAAAGTGTGTACATATCGCCCGTCACTCTTAGAGATAAGTCGTAACAAA-GTAGATTTACTGGAAAGTAGTACCTTTTGTATCAGGGTTTAATGAATATT--TTTTTTTAAAATATAT------TTTCCCGAATA--TA-AATGATTTATTTA---ATTATTTTTTAC-ATGTAACAAATGTTTAAAAATAATTTAATTGGAA-TGAAAAGTTAATCGTTTTTATAGAT-TTCTGGTAATTCGGCAAAATAAA---TTTTCGCCTGTTTATCAAAAACATGTTTTTTTGAAATTA-TAATTTAAAATTTAATCTGCTCACTGAT----AAATATTAAAGAGCCGCAGTATTT-TGACTGTGCAAAGGTAGCATAATAAATAGTTTTTTAATTGATAACTGGAATGAAAGATTGGATGAAAAATTGACTGTCTTCAATTTTATTTTTA---GAATTTAATTTTTTAGTTAAAAAGCTAAAATTTTTTTATAAGACGAGAAGACCCTATAGATTTTTTTTTATTGGGGTGATATTAAAATA-TAAATAACTTTTAAAATTATGAATTATTAATATGTAAA---TTTT-TGATCCTTTT-TTTTGATTAAT-A-GATTAAATTACCTTAGGGATAACAGCG--TAATTTAACTGGAAA-GTTCTTATTGA-AAGTTAAGTTTGCGACCTCGATGTTGAATTAAA-ATAATTAATGGGAGTAGAAGATCAT--TTAATTA-GTCTGTTCGACTATTAAAATTTTACATGATTTGAGTTCAGACCGGCGTGAGCCAGGTCAGTTTCTATCTTTAA-ATAAATATGATTAAT----TTAGTACGAAAGGAT

Liposcelis_bostrychophila TTTCATTTGACCTCAGAACTGTTC--------------AATGTTATTGCT--------------ATTTTTATTTAA------------GTAAACCCTCAGCTGTGAGTTCTTAGAATTCT--TCGTTTATAAGAATAAATATAA----ATCATTTTTTTTCTTTCCATTTATTTATTTGCTTAAATTCGGATTTCCTTACTTACCTACAGCAATAGATACTTTCTTGCTTCCATTGTTAGCTATATATGG--TGTTAA--TTAAAGATTTGCCACTGTCCTTGGTGCCACGTTTATCCTTATGTTTATGAACATAGCTATATCGCCCTACTTGCGTCGCTGCGCAAATATGCGGCAATATTTTCTTATAGATAGGCTTAATCTCATGTTTGTTC--TCCTTTGCGATCATATTTCTTTGATTGCGTGCATATCACCTAGTTTTTATCTTTACCTTAAGCAATGCCCTTTGTGCTGGAGCTATTTATTTTTGGTTACATTTTTCA--ATTTTATA--------ACACGAATTCACCTGAAATT----AAGAAAATACTTTTAATTCTTTTCACAACAAAGAATGGTCCTTATTATTTGGGTTGTCGGCTTTGGCTAGCTAGCTTTATCGGTGACTTCTTAATCAGAGCTAGTCTC--GTTTAAAGCTATACTCCAGCTTCTATATTTTTTTATATCCATTTATGGGGTTTCAATGATATCCCTTTATAGTCCCGAATGCTTCCCGTTAAAATTAGTTTGTTCTCCCCTCTTCTTTATTCTTAGATATGTGGCCGGGCGGACGGTGACGCTACCCCCTTCGCATGAGCCATCGGTTAGGTGACTGTATTTTCCTCATTGCGGATAGTCATCTGGGCATAATTATACACTCATAATTTGATGACCCGCATTGATTCTCCTTTTAGTGTCGTTTATACGCTTCTTTCTCTTCCTCCGTTTGCGGGCATACATCTTTTTGACGAATTAGACTCTTTTGACCTCGGGGGGGACCATCTTTCACATTTTTGTTTTGGCACCGAGTTAATTTATTTCCGGTTGGCTATTCCAATATTCCAGAAG--ATAAGAGTTTGGAGTTGGATATTAGCATCTTCATGGGCCTGGTTATGTTGGCCACAATTTACGTGGATGAGTGAAGCGGCTATTACTCGCACATATATGCATCCACGGGTAAGTTTTCTGTTACACGTTAGGAGACGTACCCTCTCTCACTTTGAGCTGGTTATTATTTTACATGGGGTTACGGATATCTTCAATCAGATGAGTATCTCAGAAGTATAGTGTGCCATTCATAGTCTTCATGGGCGTTTTCATTTAGGGTTAATTTGTTCCCTTTCTGGGGTCGTAAGATTAAAAAAGTCATTTTTTACTTATGGGTAACTACTTTTCCCACATTCTGGCTTCGGCTCCCGCGTATCGATACCGACATAACTACTAACTATTCTCATGGTCTGATAGATATAGATATTGTTATACCTATTTGAGGGTATAAAAAATCGTATTTATTTACCCTCTCAGATGATGATGAGGCACCCCAATTCAACTGTTTATCCCCAATTTAATTATTTTTGAAGAGGGCCGTATGACAATAGGATTCAGACAGCATATATCTTTCTATGTTCTTTTACATGTTTTGGTACTTACAAAAGGTT------AACTAAATTTACAGGAGTCTGAATTTTGTCTCCTCCGTTTATTTTTATCTGCATCCTCATCAGTCTTTATATGAGAGTATTCCCCTATACATAAATATGGAACATGTTTGACTAGATAAGGATTACAAGTAA------TTGATCGTATAGAAACAAAT------------TTCGCTCTGAGTAAAAGCCTATCTCCATACACCAGTCGCTCTTTTCTCAAGAGTATCATCTGACCTCCTCTAGGTTAAATGAGCAACCGGCGCTAAATGGTCCTTAAGTACGTCGGTATTTAGGCATGTCGAATTGGGGTGACATCTTATCCATAAGTGGTTACTCCTGATGTTAAAATTATAAAGGTAACTAGGATTCACTGTGAATAGCCTGCCTTATATAGTTACACGCAAACATCTTCCTTAATAAATAATTTTTCTGCTTTTTATACATTCATAT------TTGTTTTTCTTGTCCGGAATATCGGAAGACTTCAGGATCACCTTAAGTCACTTCCTAATAGGATATCTTTATACTCGAGTATTTTTTTTCTTTTTGACTTTTCATCGCCTAGCCACAAGAATGGAATCTGCCAGTGGGGTGACCATAACCTTGGATCCTTCTAAACCTGTTTGTTCTCGGGTTCATACTATCCACAAGATTT----AACAAATTAATTACATCTTGGTGTATACGTCTTTGGGGTATTACATCTCACTATGATAATACTCTCTTTCATATGATCGTTAGGTCATTTTTATTCACGGTTCAGGATCAGTCTGTGGACCTATATCTTATCCTATCGCTTTAGTTCATTAGTCGCCACATTATTTGATTTCTGTGTATGCATTGTGATTATTGCTTTCTTTCTTCATTATGTGTATTTGAATTGGTCCTCTGGCTTGCTTCATCAATTTACGGGTTTTTACATTTTTAAGCGACTAGCATCTTACAGGTGTAGATATAAAAATAAAAGGTGATATCGTTATCATCACGGGCTCATTTTTATATTGTAGCCAGTGGAAGCCTTTTTTCTCTTTATTTG--AAGTTGGTTCGGCTGTTTATCTTTCTATATGAGCTTCTGGTAGTTTCCTGGGCAATTCTTTGGGGCACGTATACAATTATTCGTATCCTATTGGCCCTGCGTCATGCTTGGGGGTTAAGTGGGACCACCTACCGTTCTTCTTCATTATATCCTTATATATGCATAGGGGTCATTATCTCTCAGAACGGTCTCAACCCTGGATCCTTAAATGAAAGTAGTTAGAATTTTATATAAGACTGTACCTGCCTGTTTTTGGCTATTTCTAGACATTCCCTTATTTATGACCGAAATTCTAAGCAACCATGTACCCATCAATCACCGATGTATTCTTTGCTAGCATTTCGTCGTCCAAAACTGGGGGTTTATTTGCTTTCATATATATTTATCTCCCTTTTCAAAAAAATAAGGCTAATTAGTTTT--AATGCTCTTATTCATTGGTCTTTCATTTACTGCTGGATCACCGTGAGACCTTATTATTGGAAGTTATCGTCTTATTATTTTATTCTTTATAATTATTTCAATGTTTATCTTTAGGTGCCTTTACCTTTGACGAAATCTGGCTATCACTCGAAGGCCTGAAGTGGCCTTGGCTTTCACCTTTCGAGCTTAACTTTTCAATTTCTCGCCC--ATAAGGAATTATCTTATATTACCCCTTATTCTATCTTCTTGTTTTTTTAAAACCTTTTTCACTCTT------TTACTTTCATTTGTTTCTTTCTTAACACAGGTTAACACTTGTACGGTGTCTCAATCAATATCCTATGGTCATCGAGATGCCATCCTTCTAGAATACCTGGCTCTTTTTAGTTGCTTATATTCTCACTTATTTAAATATAATTAATCTCATCTCACTTTTATGCC----CTTCATATTTTTCTAATATTATGAAGAACGACCCTTGATTTCGATGGATCGACTGTTCGGTTAAGTGATTGGGGGCGATTTCTTATTTTTGGGAAATTATTTGTTTAATCTTGTTTTCTTTTATGGAGATTCGTTACTATTTTGCTGTATATTTATAA--------GTTCATCGGGGCTACCCGTACGTTGAAGATATGATTTGTGTTATTACTCCCTACATGTCTCTAGGTTTACTTATCTATGTGTTTTTAT----------------TTTTGTCAATGTAAAGGTGGAAGGGAAGTAACGACACCTTGATGGGATAGAGAATTTCTCCGATCTTTTCCTCCTTTTTTATACTTCTTTTTATTTGAGTGAATATCTCTTTGTTTATTTTC----GACTTCGTATTTTTCTTTTAATGTATTTTT----CTCTATGCAGCTCTATGATGTATAGGTCCTGTTGATCTCTATATATCTAAATGAATATATGTTTATTTTTTTATTTAATAAAATAA----------ATGTATATATTTATGTATATGTTGGAGCATATGGCTATTATGGCTGTGTCTTATTTAAAACTAATCATTCTGAGTGTTTTTTTTGAGCTATCGCTCTTTCTCGTGTCTTTATTCGCTCATGTTTTATCATTCTAATCACAGAAAGAAAATAATTTTTTACCTTAATTTATCTTCATCTATTTATTTTCTTAAATTCTCCTCTGTGGTGGAGGTTGGGTACTCTTCTTTATTATATACATCTTAAAGACAATCTCCTATACTTACTTAACGGTGGGATTATATATTTTCATACATGGCTACGTTAAC--TGAATTTTTTAGGTGAACAT--------------AAGTTTAATTCTTTATACGCGCTTAGAAAGGCCACTCCTTTCTCTGCTCCCTGCATGCGCCCACCCGTTCTCCTGTCATCTCACCTGTACGCGGGTTACTCTTACG--GCCC--TTTCATATAGTGCTAGAATA--ATTTTTATACAGCTACCTATATAGAGGTTTGCTTCAAGTTGATTAAGAATGTGCTTTCACATAGCAATAGTTATATATGGATTCAAGGCTTAAATTTCTTTTCACTTGACCAGCTTTTAAGCCTTTATTCAGTGTCATATGGCTCCTTATGCGGTTTATCAAGAGG--CTATGA--GAAGTCTT--------TTTCGTTCATCTTTGTATCCGTCTTTTCTCCTTAACATCGTTTTTATAATTGTTTCAAAC----------TCGGATAGTTAA------------ACAAGAATATAGTTTCATTTTCTTGCTGTTTCATTTAGGGGCGCATCATG------------AGCTTTCCCT----CTTTATTCATATCC--CATTAAACTTATTTATATTTATATAGATGTATTTTAATACGT--ATTTACTTGAAG--TCATTTAGGCTTGTTAATTTGTATATTTTTGGAGGGATATGCACGTTCGGAGGTTGATAGATATTTTTATTTATATGGGGTTATGTTCTTTTTAATGTAGTTACCA--AA--ATGTTTAGATTCCCCTGTTT------------------TTATAGGTTGCTGTCCTTAGCTGCAGTTGGAA--------------------------TTCAAATGTTGATATTTTTTTC----------------------------------------TCCCAGTACTGTGTGTTTTTATTTTTTTCTTTTTATCTTTATATGATTAACTAAGCATAAGGGCGGTAAAACAT-------AGCCCCATTTTATTCTTTCT-------------------------TAGATTT---TGTTTATAGGATGGTTAACTTAGAG---TTTATTGAGTCTAATGACATAGGAAGTAGGT----------GAAACAGACTAGGATTAGAAACCCTATTATTTCT---------ATTACTCATATTCTAAAGACTAACAAT-------GATCTG------------------------TGGCGGCTCTCT----TTCAAATTAGAGATCCATGGACC--TAA-TGGACAGCCCACCCA-AATCTTCATATG-----------------------TTTAGCTCTA--CATTTTTTATATCGCCGA--CTGAAAAAAAAATC--------ATAAATTTTTATTT------------------------TGTCAGTTTAA-------------------------TACTGATAAGTCAGGTAAAGATGAAGATTATAGCAT-TGAAAGTTTGTGGATCA----AAA-----ATTTTCA-------------GGAATTAGGTAGTTGAGACAC------TATAAAAATGAATTTAAAAGTAAA-GCTTCTTAATTAAAGTGACTTGAAAATTGTT-AAAGAGAGTGTACATACCGCCCGTCACTTCTAGGAATAAGTCGTAACATG-GTTAATCTACTGGAAGGTGGTACCTTTGGTATCAGGGTTTAT--AGATTTC-TGAAACTATAA-TGTT------ATTCCCGAATA--GA-TACGATTTAATTA---GATGAATTTAATTGTAGCAAAA--ATTTTTTAAATTTTAATTAGAAG-TGATAAGCTTATCGGGTGTC--TAG-ATCTGGTAACTCGACAAAATTAT---CAA-AACCAGTTTACCAAAAACATGTCCCTCTTTAA-----TTTCTTGGGTCGGCCCTGCTCACTGA-------ATTTTAAAGAGCCGCTTTA---------GTGTTAAAGTAGCATAATCAATTGTCCTCTTATTAAGGTCTAGAATGAACGGGAGAATTATTGATAAATTATATACACTGAAATTTAAA----AAATTAAATTTTAATTTATAATGCTTAAATTAATTAAGGGGACGAGAAGACCCTA-GGATCTTTTTTAGCTGGGGAAATTAGAAAAAT-AAACT--TTTCTAGATTTAAACATTATAGATGTTTG------TTTAAGATTAAAGTAAATTA-------A-GATAAAATTACCCTAGGGATAACAGCG--CCATAACAAGTCAGATGATCAGAATTT-TACTTGTGATTGCGACCTCGATGTTGAATTAAA-ATTAG----AGGCCTGGAAGTTCAGGCCTGATTCAGTCTGTTCGACTGGTTAA-TTTTACATGATTTGAGTTCTGACCGACGTAAGTCAGGTTAGTTTCTATCTCTTA-T---TTTCACCCAGG------AGTACGAAAGGAG

Pediculus_humanus ATTCTCTTGACCTCACTCATATCTGG--TTAAGT----AATGTTATATCT--------------TTCCTTTTTTATACGGAGTATATTATCATCGGTACGTTTAGTAGTTGTTTAAAATTTTATAACATAATTC----------------------ATATAGTTTCGTTTATATATTTACCTAAACATTCCTATCCTTGTTTCCTGACTCCATTAGGTAATTGGCTTGTTCCTTTGATAGGGGTGTTATC--TTAAAGTCATCGGGTTCTGCCACTCTCCTAGGAGCCACATCTAGCCTTTTGTGTATGATTTTAGGTTCATCGCCGTTCCTAGGTCGCTCTGCAAATACGGGGCATTATATAACTTTGAGAGGTTTCTCGCGTCTCTGTCTCCTTTCATGCGCTAGTCTCTTTGCGCGACTTTGTTCTTATCATCTAGTTTAGAACTGTTCATTATGGACTTGCCTCATTGACTTTATAATATGTATTTGTTTTATTATTTAC--ATATTATTATAAATGAAATCAATATTGAAATTAATT----AAAAAAACATTCAACTTCATTTTCACAACAAAGAATGGTTTTTATTTGTCGGGTTGTTGGCTTTGGTTAGTTAGTTATATCGTTGACTTCAGACGGTTCTTTTCGAAGCACTTAAAGTTTGTACTCCAGCTTGTATATTTTTATGTATCCGTATATGGGGTTGCAATGTTGTCCTCATTTGGTCCCGAATGCTTCCCGATAAAAATAGTATGCTCTACCCTCGGATTTCTATAGAGTCTTGTCAGGGGGTGGACGGTGACGTTACCCCCTAGTCCTGAGGCACCTCGTTCGTGATTGCATTTAGCTCATTGCGGGTAGTCATTTGGTCGTAATTATAGACATTTAAATTGCCCATATTGGTTGTCGCTCCTTTTTGTGAGGTTTGTACGCTTTTTTTTCTTCCTCCGTTTGCGGGCATACATCTTTATGACGAATTAATGTCTTTTGACCTTGGGGGGGACCGTTTTACACATTTTTGTTTTGGCACCGAGTTAATCTATCTCCGGTTGGCTATTCCAATGTGTGATGTGGGAAAAGAGTTTGGTCTTGGATATTAGCATTCGCATGGGCTTGGTTGTGTTGGCCACAATTTACGTGGTTGAGTGAAGCGGCTATTACAGGCACATACATGCATCCACGGGTAAGTTTAGTGTTGGACTTTTGGCCAATTAAAGAGATAGTTTTTGTCTTGGTTATTTCTTTACATGGGGTTACGGATGTCTTCAATCTCGTGAGTTCCTCAGAACTATAGTGTGCCATTCATAGTTTTCATGGGCGTTTGCATTTGGGCTGAACATGTTTCCTGGACGGCTAACTCGAATCTTATAAGTCATTTGTTAGTTGTGGGTAATTACTTTTCCCACATTCTGGTTGCGGATCCCGCGTATCGATACCGAGTTACTAGTGAAAAATTCTCATGGAGCTATACACTTGGGTGTATTTCTTTGCCTATGAAGTTTCAACCCAAAATGTTTAGGAGCAGTTCAGACTCCCGCTATGGATCCGCAGATCATCCATTACCTACTTACAGGTTGGGTTCAGAAGAATCCCTTATGTTTGTTGGAACTAGACTGTTCATGTTGGTGGGTATTCTTGTATTAGTGCGTAGTTTTTTATAATCTG------AATATATTATGGCTGAAGTTGAATGTTGGTATTTCCTCCTTCTTGCGGTTATTTCCTCCTCATGTTTATTATGAGAGTCTTCCCGCATAGTTAAGTGTGGCACATGTTTGTCTAGATAGGGATGGAAAATGA------TTGATCTAATATAATTGAGACTGATCTCTGCCTTCGCTTTGAGCGATTAGGTTTATCCTATTACGAGTCGGCATGTACTCGCGAGTATCATCTGGCATCCATATGGGTAAGTGAGCATCCGGCGTTAACAGCCTATTATCTTAAATGGACTCTAGGCATGTCGAATTGGGGCTACAAGTTATCCATAAGTACACCTCCAAGAGATTATAATGGTAAGATTAATTCACCTTCACTGTGAGTAGCCTGCCATTTTTAGTTTCCTTTTTTCGCTCATACTTTGTGATAAGGCTTATCTTTAATTTATATAGATGTGTTCTCTTATGTTCTTTGTGCGGAGTACCGGAGCACTTCAGGAACAACATGAGTATGCGGTTCGTTGGATCTATTTATGCTCGAGTATTTTTTTTCTTTTTAGCCTTTTTCTTCTTAGCCGAGTTCTTGGCTCTTACCCCGTGGGTAGCCGTGGGTTTGGGTCCCTTTAATCATTTTTCTTCAGGGGTTCATACTGGCCATAGACTTT----AGAAAAATTCTCAGCTATGGTTTTATACTTATTTGGCTGTTTCTACTTCAGCGTGATAAAACAGTCTTACATGCGAAGAGTTGGTCGTTTTTCTATACGGTTCAGGGCCAGTTGGTGGGTGTTTATACATAGACATCGCTTATTAACATAAAAAAACACATTGGCTGACTGCGCTGTATGCATTGTGAGTGTTGTTTTTTTACTACCTTATGTGATCTTTAATTTGGTTTTTGGTTTTCTTCATCAATTTAGGGCTTTCTGCTCCATAGAGCTCACAA--TCTTTGAGGTATTTATGATTGAGTAAAGGGTGTTATCGAGTTCAGCAAGGGCTCTTTTTTATCTGTTAGTCAATTGCGGGTTTGTTGGTGTTACCAAA--TAGTTGTTTCGGATTCATCTCTCTATATGCGCGCTTATGGTAGTCTCCTGGGCAATTCTTTGGGGCACGTATACAACTTTAGGCATCCATGTGGAGGATTGTATTGGTTGGGGGTTTCGTAGCACCACTTGACGCTTTACCTCATTCTTTCCTTGTTTTTGGTTGTATGCCAATATCTCTCACACAGGTCAGAACCTTGGTTGATTGAAGGAAAGTTATTTACCTATTTACTAAGAATTTGGGGTTGTTGTTTTTTTTGTTTATTGATTATCCCGATTTTATGACCGAAATTGTGATCAACCATATACCCCCCAATCACCGATGTATTCTTTGCTAGCATTTCGAGGTCCAAAATTGGGGGTGTGCTTCTCTAGATTTTCCTTCTTATAGAT------GGAGTCGTTCAGCGTTAGATAGCGATATTTACTATCTTACAGGTTTGTATCTTCTGCTGGTCCTCCGCGATACCTTACCTCTAGCAGTGTAGGTATTATTATCAGTATCTTTATCATCGTTTATGTTTTCCTTTATTGGTGCTATTTCTTTTGACGAACTCTAGTTGACAATCGCTGGCCAAAAGTGGCCATGGATCTCACCTTAGGAGCCCAATTTTTCAAACATTGCCCA--AGGA----TCTGGATTTTATATCCTTATACTTATTTAGGTTCTGTGTACCCTTATTCCAAACTTTG----GATCAAAATCCTTTATCTATTTATTCAGGTAGGTTAGCTTATTTACGGTCCTCCAATCAATATCGCCTGGAGCTCGGCATACCTTCATTCTTGATTGTTTTCACGCATCTAGATGCGTGTTTAATCTTAGATAATTATGCACAACAAG--GTCCAAATATTCATAT--GTGTGGATTTGTTGACTCCTATGCGATGGGCGACCCTTGATTCCGAAGGATCGATTGTAGGGTTAAGTGATAGGGGAGCGTAGTTTCTTATTAGGAAGTTTTCTACATTCTCATATATAGATTTTTACTG--GGTAAACCTTGTGTTTACTGATAGATTC--------ATGTATCGGCAGAACCCGATCGTAGAAATGATATTTGGTGGATTTCATCCTTATTTATTTATAGTTACATTTATATTTGCATATTTGC----------------TTGTACTTGTTTGTTCCAAGCCAATTACCTGAAGACCCTAGATGGGGTATCCTTAGATAGACCTTCACCAATCATTTAGTGTAGGTGTTTCTATTTGAGTGATTGTGCACTTCCGTGTACTC----AGCTTTGAAAGATGTTTCAT--TGCTTTATCCCTATCTACTTGGTTCTTTGACTCATAGGAGTTGATGAAATATTCTCCTATTCCTGATTAGTGTGTGGTTAGTATATCAATGCCTTCGA--AGTTGAGCCTTTAGTCGAGTTTAGGTATGTTGGAAGGTGTGGCTTCTTCTATAGCTACTAGGTGGGAGACGGTTAGGGGTGAATATATGTCTGAAATTAGCTACTTTTCTATGTTTACATAGTCCTGTATGCTAAGAAAATAATGCGGCAAACTGGGGGATTTAGTTCATGTTTTTATGTAGATTTCTCTTCCTAGGGTCATTTTGTCTTATGGTGGAGGTTGGATATAGTTGTTTATTTTTAAAAAGTGAGTCCAAATCGGGTATACTTCTATAACGTTGGGATCTTATATATTGTCTCTATTAGGTTGGGAT--TGGA----------------------GTTATGTTTGATTTACTCTTTATTATGGGGGCTCAAAGGCCATTCCTTTCAGTGTTCCGAGCATGCGCCCACCCGTAGAGTTGTCATCTCACTTGTACGCGGATTAGTTTGCCGTAGGAG--ATATGAAGTTTAAT--------CTACTATTTCTCATTCATATATTCGGGTTCGCCTTGAGAGGATTAAAAGTGTGCTATCACCTTCCAATAGTTATCTTTTATTTCGAGGAGGTGAGGGCTTATCAATTTACCATCGTTTAAAGCTTTTTTCTGCTTCATGCGGCTCCTTCTTCGGGGTATCAAGAGT--TT------CTATTTAGTTAAAGTCATATAACTATATTTCTACGCGTATTTACAGGGTATCTTCGATATTATTCTTCAGAA--------------ATAAATACCAAA--------ATGCGTAGAGTTTTAGAGCCCTAACTAGCAATCTAAGTCT------ATTCGCTGATTCTCAGCCGGTATTTCAACT----AGAGCA--AGGTAATTGAGGAAATATATCTTTATATCTTTGGGTGTGCATTTTTACTT--CTATTTGTGGTC--GACTTTCTAACTTTTCCTGCATACGTTTACGTGGCTCTATCTCTAATCTATCTCTGTCTGTTCTTTTGTTGGATTTGGGGTTATGTTCCTTCATGCTTATGTACCC--AA--ATAATCTCAAGATGTCAGAGTTAG--------------TTATAGGCTTTGATTTACTCGTATCTTCGCAT----------------ATTGAAGTGATTAAGATGTCGATTTATCACGA--AGGTAGGA------------ACTAAATCCT--CTATGACTAAGTTAACGCGTGTATCTTTAT----TAATTTATCTCCGTATGAGTTTACTCCCTAAGCGGCGGTCATACATTA-----AAGTCAAA-TTAATTGGTTAAAGCTAATAAATAATTACTATTAAGTACAACAACTCTACTTATTATTGTAAGAGTAAAAT---CTTTATAAATAAGATCTTACGAGGCGCTAGC-----------ATAAAGACTAGGATTAGATACCCTATTATGGGCGTGAGTATGAAAGTTTATTAAGTTAATAGCCTTAATTAAAGCA---------------AAACCTATTTACTA-TGGCGGCTGTT----AGTCTCACCAGAGTCGTATGTCCT--TAA-ACGAAACTGCGCGAT-CATCTTACCTTTTAACTTTTGCTAATATAAAATAATTACATTATAGCAATCTGGCACGTCGCTGTACTAAAAGTGAAGATT-----TTAAATAACTCATCTTC------------------------TAATTACTTATTAGCA-------------TTTATTAATAACGTA---CAAGTCAAGACGCCGTTT----TAAGAGGCTTAAGATG---TACGATTTA-----ATTGATTTG---------ATAGAATTTATAAGAATGAAAACTTTCTTTATTAAAAT-AATTTGGAAGTAAA-ATAGGGTTATTATTCTTATTTGAAATGAGCTACTAATAAGTGTACATATCGCCCGTCATTCCAATGGACAAGTCGTAACAAA-GTTGATTTACTGGAAAGTGGTACCTTTTGCATCAGGGGTTAAGAAATAA--GATTATTAAATTAATAA------CCTCCCGAAAG-AAGC-AGGATCTTAAACTTTATTGTTGGTTGTTGTTTCATTAACATTAA-TAATAATATTTAAGAAGCTAAAAGTTATAACGACTCTTCTGATCAC-TGGTAACTAGGCAAAC-ATTT--AGCCGGACTGTTTAATAAAAACATTTCCTCTTGCATAAA---AGGG-AGGTAAAGTCTGCTCGGTG-----TAATTGATTAACAGCTGCAGTAACT-TGACTGTACAAAGGTAGCGTAATCACTTGTCTTTTAATTGAAGACTAGAATGAACGGCTTAACCAAGCTAATTCTGTCTCTTTTTAACCTTAAG---AAATTTAAGATTTAAGTTAAAACGCTTAAATTATTTAGAGGGACGAGAAGACCCTGAAGATTTTTTTTATTTGGGATGAATGT--------AATAAACATTATG----GTTTACTAAGACTTA---AACGTCTTTATTCTGACCCGTTTAAACGAGAATCTGTTTAAGTTACCTCAGGGATAACAGCG--CAATGTCTTTTATTA-GACCTTATAAAA-ACAGAAGTTTGCGACCTCGATGTTGAATTGAGTTAAACTTTAAGTAGAAGAGTACTTA--AGAGTAG-GACTGTTCGTCTTTTAATAGCTCACATGATTTGAGTTTAGACCGACGTGAGTCAGGTCAGATTCTATCTTCTAATATTAAGCA---ATCTT-TTTTGTACGAAAGGAC

Haematopinus_suis ATTCGTTTGACCTGTCACTTTTAGTTAATTCCTT----AATGTTCTGTGT--------------TTGTGTTTAGCTAGGGCGTATGATCTTCTCGGTTCATGGTATGTTGGTAAAAGGCTATCAGGTTCGGATCTAAAAATAAA------CATTATCTATCTCAACCTTTTTTTATTTTCAGAATTATGGCTTCCCTTATTTACCTTCTCCATTGTTAAATTAGTTTGTTCCTTTGTTGGGGATTTTATC--TGTCAATGTGAAAAACTTGCCATTACCCGTGGAGCCGTGCTTGCCCTTTTGTTTGTGAACGTAGTTATATCGCCATAGTTAGGTCGTTATGCAAATACGCGGCAATGTATACCTGCGACAGGGCATTCGTGCTCTAGTGGAGTTTAGTTTTTGTATGTTTTTTTTTGATTGGGTGCCTATCAGCTAGTTTATAGCTATTCTTTATGGAATTCCCATTGTGTTCTCTGAGTTGTTTTGTAGTTATAGTGTGAG--TGTTTATGGATTTTGTGAAGTTGCTCCCAAGTTTTT----AAGTTTAAGAGGGATAGTTTCTTCACAACAAAGAATGGGTCTTATTATTTGGGTTGGCGGTTTTGGACAGATAGTTTTATCGGTGATTGGAGGTAAAGTTATTCAGGGCAGTTAAAGTTTGTACGCCAGCTTTTATATTTTTATATATCCGTATATGGGGTTGGAATGTTGTCCACATTTGGGCCCGAATGCTTCCCGATAAAAATAGTTTGCTTTCCCCTCCTTTTTTTATTCAGTTATGTGGGGGGGTGGACGGTGACGTTACCCCCTAGGGTTGTGGCACCAGAGTCGTGATTACATTTAGCTCATTGCGGTTAGTCATATGGGCATAATTATTGACATGTAAATTGGTTGGGAAAATTGACTTTCCCTTTTGTGTCGTTTATACGCGTCTCTTTCTTCTTCCGTTTGCGGGGATACATTTTTATGACGAAATAATGTCTTTTGACCTTGGGGGGGACCGTTTTACACACTTTTGTTTTGGCACCGAGTTAATCTATTTCCGGTTGGTTATTCCAATATATGAGAAGGGAAAAGAGTTTGGACTTGGATATTAGCATGTGCATGGTTTTGGTTATGTTGGCCACGATTTACGTGGATGAGTGAAGCGGCTATTACAGGCACATGTATGCATCCACGGGTAAGTTTAGTGTTGCACCTTTGGGGAACTGTATTCGTACCTTTTGTGTTGGTTATTTTTTTACGTGGGGTTACGGTTGTTTGCAATCTCGTGAGTGTTTCAGAACTATAGTGTGCCATTCATAGTTTTCATGGGCGTTTGCATATGGGCTTAACATGTTCCATATAGGGGTAGTTAACAAATTATAAGTCATTTGATACTTATGGGTAAATACTTTTCCCACATTTTGGTTAGGGATCCCGCGTAGTGATACCGAGTTTCTTGTGAAATTTTCTCATGGAGCTGTAGGCGTGGGTTTTTTTGTTAGCATTAGAAGCTGTAGAACGGTGTGTTAAGCTTC--GGAATCTTGAGCATTTGGTGCCCCAAGCCAACCAGAAGGTCCTTGTTTAAATGGTTCAGAAGTCTCCCATATGGTAATACGGGTCAGATGATATATGTGTTTGTGTGTTCATGTATTAGTTTGGGGATATCTACAAGGTG------GACGTTCTGTAGGCGAACTTGATTATTGGCGGCTCCGCATTCTTGGTTTTGCATCCTCCTCATGCTTACTATGAGAGCTATCCCTTTTAGTTAAGTGTGGCACATGTATGTCTAGATATCGATATCAATTGA------TTGATCTAATCTAGCAGATCTT----------TTCGCTTTGAGTGAAAGCGTGCATCCATGATGGAGTCGGTTTGTACTCGGGAGTATCATCTGACGTCCTCATGGGTAAAGGAGCATCCGGCGTTAACACTGTCTATGGTCAATTGGTCTATAGGCATGTCGAATTGGGGCAACAAGTTATCCATAAGTGAGTCTACAAGATTTTATAATGTTTTAAGGGGTTCACCTTCACTGTAGCCAGCCTGCCCTTTTTAGGTTCACTTTCCTATGTGGTTTAGTTGATTC--AGATGGAGGTTTATGTTTGGGTTTAGGTATTTAGTTTTTGTGTTCGGAGTATCGGAAGACTACAGGTGCAACATCGGTATAAGGTTCGTTGGATGTATTTATATTCGAGTATTTTTTTTCATTTTTGGGTTTTTTTTCTTAACCGAGTGTTTGGAGTCTACCCCGTGGATCACCTTAATAATGGGTCCTTTTAAACATATTTCTTCAGGGGTACGTACTGTGCACAGGATAT----AGGGAAAACACAAGGTTTGGTTACATACGTGTTTGGGTTTTTGTATTTCATTGAGATATAGAAGTCTAACATGCGAAGGTTGGGTCTTTTTAATTCACGGTTCAGGATCAGTATTTGGACGTATTTATGTAGTTGTCGTTACATAACATTAGAGATCAAATTGGTTGAATTCGCTGTATGCATTGTAGGTGT------TGTAGTTCGT----TGTATTTGAATAGGTCTTTTTTATTGTTGTATCAATGTAGGGATTTCTTCATCATAGAGCTCATCTAAGCTTTCAGGTGTTCATGTAAGAGTAATGGGTGCTATCGATGTCAGCAAGGGCTCTTTTTTATGCATTAATCAATGGCGGGTTTATAGGAGTACGATGT--GGGTTGTTGTGGGTGTTTTTTTTTCTATGCACGCTTTTGGTAGTTTCCTGGGCAATTCTATGGGGCACGTATACAATTCTTCGCATCCTATTGGGAGCATACGGTGTTTGGGGGTTTCGTGGAACCACTTGTCGTTTTTCTTCATTGTTTCCTTGTATTGTTTTGTTTTTCATTGTTTCTCATGTTGGAGTCAACCTTGGTTTCAAAATCGAATATTATTCACCTATTAGGTAAGAGTCTGGTTGTGTGCTTTTATGTTGGTGTGTCTCTTTCCGATTTTATGACCGAAATTATGAGCAACCATAAACCCCCCAATCACCGATGTATTCTTTGCTATCATCTCGTCATCCAAAATTGGGGGTGTTCTTTTGCAGGTATATTTGCTTCTCCCTTA----GGAAGGTTAGTTCGTTATGGCTAAAAATTTTATGTTCAGTATGTTTTTATTTACGTTTGGTCATCCGTGATACCTAACGCATAGCAGTATACTTATTATTGTAATTATTTCTGTCAATTTTTACATATTCATTTATGCGTGCTTTTTCCTTTGACGAAATTTAGATTCCAAACGGAGGCCAAAAGTGTCTAAGGTTTCCACCATGGGAGCGTAACTTTTCAATCACAGTTCC--AATTGGTTTATCGTTAACTTGGCCCTGCCTCTAGATAAACTTTTTGATACACCC----TTCTTCAATTATCATTAACATCGGATGTATCTCTATCTAGGTACGCCTCCACATTAAGGGTGTTTCAATCACTTAGACATGGGCATCGTCGTGCCATCCTTCTTGAATACTTAGTTAGCTTTATAGTTCTATATCATCTTTGTTGAAACTCCAATTCAAGTGAGTG--CTTTTGTGATCCTGATTCCTGTACTTATTGTTTTGCGAAGGGCGAGCCTTGACTCCGAGGGAAGGATTGTAGGGTAACATGATTGGGGTTCATAACTTATTTCTGGGAAACTGCGTATTTATACATATTTTCACACTACTGG--GGTTTCTTTGAAGCAGACCTGTTTATAT--------GTATATCGAGTCTACCCGATCGTAGACACTATCATTAATGGTGGATTTCCCACTATTCTCGTTGTTGTCTGTTTTCATATTTTTTTTT----------------GTCTTCGTTTATTCTCATTCGAACAGTTGAACAAGATCTTGATGGGTTTTACGGAGGAATCATTCCTTTGGTCATTTTATGTGGATCTTTGTGTTTGAATGATTGTATTCCTCCCTATGTGC----AACTAGGACCGTTGTTTTTG--TGTTGTTAAGATATTTTTATGGATCTTTGAGTACTGGGTCATGATGGGATATATGCCTTTAGGTGATTCTTCGTAGCATTTAGCGTCTTTCTGTAACCAGTCTTAATTAAAGTGTTGTTTCTTCATCTGTTTGAGGAGTTGGTTACATCTGTTCACAGCTAAATGATCACATATTGGAGTGGTGGTATGTGTGAGGTTAGTCTTTTTTTTACGTTTCTGTAGTTATGTTTAATAAGGTCATATTTCTATTGAGGTTAAAATTATATACCTTTTTTTGTATTCATTGATCTTGTTAGCCAGATTTTGGTATGTGGTGGAGGTTGGTTACTCTTGGTTATATTTTACAAATGAGAGTTAGAGGGTTTTACTTTTATAACGATGGGAATTTATATTCGTATGTATCTTCAG--------TGAAATTTAAAGGTTCATAG----------TCGCGTCAATTCGCCTTTCTTTGGGCATACAAAGGCCATTCCTTTCTCTGCTCCCTGCATGCGCCCACCCGTAGAGTTGTCATCTCACTTGTACGCGGATTATTCTATCGTTGAAG--TTTTCCTCGAGTTTCAGC----CTAAGTGTTCATATACATGTTAGCGGGTTCGCCTTGGAGTGATTAAAAGTGTGCCTTCACCTACCATTGGATATACCTTAGTTCATGGAGGTACGCGCACACCATTGTTTCAGCTTTTAAAGGCTTTTATCTTCTCATGCGGCTCCTTTTACGGTTTATCAAGAGT----------ATGTATTTGCGAGATCTTCTACCTGTTCTTTTTTGGGTATTTACTCGGTATCGTCGTTATGTCTATTTAATC--------------CCAATTATTCGAAAAAGATTACCAGTAGGAGGTTGTAGGCAGGTCAGGTTATATAGGTCTGGGGAGATTTCTTG----GTGTATCCATACATGTAT----GGTAAATTAGTGAGGTTTAAAACTTTATATGTTCCTGCGGGGCTATGCGCTTTGTCTT--TATTTGATGCTC--AGTGAGATGTGTCTTCTTTCGTCAATGTATTAAGGTATTTGCATAGGTACCATATGATGTATTTAGATATGGTTTTGGGGTTATGTTTCTTCTTATCTATATCTCC--AACCGATTGGGTTTAAAATGAA--------------------TAGTGGTTTCATCTATTTTTATCTACAGATTA----------------------------------------CTCCGTGTGAAAAAGACC------------GACTTTCCAT--TCCTCATTTAGCTAGGCTTGTTTTTTTTCTTTGTTTCTATATGTGGATATAATAGGTAAATCATTACTGCGGTCATACACTA-----AGCTCAAGTTAACTTTTATACTGAT--------------------TATACTAGTAACATGAAATAGTAGCTAAATTTAA----GTAGGAAGGTGAAATAAAGTAAGAAATTGTA----------CCTATAAACTAGGATTAGATACCCTACTATA-TACAACGTA----AGTATCATATCAAGGTAGTAATGGTTAC----GGTCCAT-------GAAAACTAATAACATATGGCGGTTGAC----AATCTTTCCAGAGACCCATGTTTG--TAA-TCGATAATGCACGTC-AATCTTACCCTAT----------------------TTAA--------GATTTTGTACACCGCTGT-------CGGAAGGTT-----GTGGATAATTT------------------------------CATAATCTATT------------------ACTATCAAAGTAATAAGTCAAGTCAAGGTGCAGACTACGGTGG-GGGTATAATGTGGGTCA-------------------------------TTGGTCTAATATGAACAGACATGT----TATTGAAAAGAATTTGGTAGTAA--ACTTAAAAATTATATTGAGTTGAAATAGCA--ATTGTCAATGTACAAATCGCCCGTCACTCCGTTGGATAAGTCGTAACAAA-GTTGATCTATTGGAAAATGGTACCTTTTGTATCAGGGACTTAGGAATGAGTAACAGTTTATA--CGAA------TATCCCGAAAA--GAAAAGAATCCTCTAT---ATTAAAGATTATTGTTTAATTAATATCTTCAAATTAT-AAAGAGTGG-TGAAATGTCATACGATTTTCTAGTT-ATCTGGTAACTCGGCAAATAATT---AGTCTGACTGTTTACTAAAAACATTGCCTTTAGAAG--T-AAATTGAAGGTAAGACCTGCTCACTGCGTT-GATAACGTAAATAGCTGCAGTATTC-TGACTGTGCTAAGGTAGCATAATAACTTGTTTCTTAATTGGAATCTAGAATGAACGGTTCTACAAGACTAAGTCTGTCTCTGTGAAGTAAAATT---GAATTTTAATTTTAAGTGAAAATGCTTAAATACGTTAGAAGGACGAGAAGACCCTATAGATTTTTTTTACCTGGGAAGGGGTCTACACA-CATTAAGCTATGTTTAATAAAACTAAAATAATAGGAAATTTAATGATGATCCTCAAGTTGTGAAAAAA---AGATAAATTACCTTAGGGATAACAGCA--CAATTGTCTTTCCTA-GATCTAATTGGATGATACAGATTGTGACCTCGATGTTGAATTAAG-TTATCATTTAGATGAAGAAGGTTAA--ATGGTTA-GTCTGTTCGACTATTAAAAACTTACATGATTTGAGTTTAGACCGACGTGAGTCAGGTCAGATTCTATCCTCTG-A-AATTGAGTTTACCCT-TTTAGTACGAAAGGAT

Pthirus_pubis ATTCATTTGACCAGATATTTGTACGG--TTCCGT----AATGCTGGGCAC--------------TTGTTGCTTAGCGGGGGATTTGCTGTAATCGGTACGAGGCGTACAAGTTGTTTTCTCAGATTCA----------------------TTATCCTTTTGGGTGTAGTTTATGGTTTAGATAAGTATTCTTATCCTTACTTCCTTACTCCAATTCGTAATTGGATTGTTACCTTGTTAGGGTTCTTATC--TTGGAGTCCTAGAATCTTGCCATATTCCTTGGAGCCATGTCTGGCCTTCTGTCTATGAGTGCAGGTCTATCGCCATAGCTAGGTCGCTATGCAAATTTGGGGCAATATATAGCTATGAGAAGTCTTGG--AGTTATAGGGATTTACTTCTACTATCCTATGTTGGTGAATTTGTGCACGTCAGCTAGTCTAGAACTTTAGATTATGGACTTCCCTTCTTGTTAAATTTATTTATGTTTGTTTATGTATTTTC--GGTCTATTTCATTCACGTGAGTAATTCGTAGAGCCG----GTATTTACTTGAAATGATCTTTTCACAACAAAGAATGGCTTTTACTCTTCGGATTGTTGGTTGTGGTTTCATAGCTATGTCGGTGACTTCTCACAGTCTGTTGTAAAGCAACTAAAGTTTGTACTCCAGCTTGTATATTTTTATGTATCCGTATATGGGGTTGCAATGCTGTCCTTTTTTGGGCCCGAATGCTTCCCGATAAAAATAGTATGCTATATCCTCGGGTCTTTATGCAGTCATATCAGGGGACGGACGGTGACATTACCCCCTAGCCTTGAGGCACCTCTTTCGTGATTACATTTAGCTCATTGCGGGTAGTCATTTGGTCGTAATTATAGACATTTAAATTGCCTCAGTTAAATTACGTTCCTTTTTGTGTCGTTTATACGCTTTTCTCTCTTCTTCCGTCTGCGGGCATACATCTCTTTGACGAATTAATGTCTTTTGACCCTGGGGGGGACCGTCTTACACACTTTTGTTTTGGCACCGAGTTAATTTATTTCCGGTTGGTTATTCCAATGTGTGATTAGGGAAAAGAGTTTGGTCTTGGATATTAGCATGTTCATGGGTCTGGTTGTGTTGGCCACAATTTACGTGGCTGAGTGAAGCGGCTATTACAGGCACATACATGCATCCACGGGTAAGTTTAGTGTTGGACCTTTGGCCAACTCATTAGGTAGTTCATGTCCTGGTTATTTTTTTACGTGGGGCTACGGATATCTTCAATCTCGTGAGTCTCTCAGAACTATAGTGTGCCATTCATAGTTTTCATGGGCGTTTGCATTTGGGCTGAACATGTTTCTCTTAGGGTGTACTAACCAAGTATTCACCATTTGGTAGTTATGGGTAATTACTTTTCCCACATTCTGGCTAGGGATCCCGCGTATCGATACCGAGCTTTAGGTGAAAAATTCTCCTGGAGATCTACTTGTGGGTCTCTTTCTTAGCTCTTGAAGGTGCAGGCAAAAGTCTTTGAAAGTAGGTCAGACTGTGGCTGTGGAACCTCGGTTCAACTTACACAGTTCACTTCGATTACTTCAGAGGAGTCCCGTATGCTTGTTCAAACTAGACTGTTGGTGTTGGTGGGTATGCTTGTGCTAGTTCCTTTTTCTTTAAAATCTG------AATATATTGTGGCTGAAGTTGAACATTGGTATTTCCTCTTGCCTGCTCTTGTTTCCTCTTCATGTTTATTATGAGAATTAACCCTCGTACCTAAGTATGGCACATGTATGTCTAGATAGGGATGGAAGATGA------TTAATCTAATTTAGGAGGGATTAAAAGTGATCTTCGCTTTGATCGATGAGGTTAATCCTCAGACGAATCGGCATATACTCTCGAGTATCATCTGGCATCCAGCTAGGTAAATGAGCGTCCGGCGCTAACATCGTATTATCCAAACTGGAGCTTAGGCATGTCGAATTGGGGCTACAAGTTATCCATTCGTAAACGTCCAAGCGTTTATGCTGGTAAGAAGAATTCACCTTCACTGTGATTAGCCTGCCTTTATTAGCTTCTCTTTGTTGCGTGCGTCTTGATTTGGTCTTAAGGTT----TTGCTTTCGTATGTTTGTCTGTCTTCTTTGTGCGGAGTATCGGATCACTTCAGGAACAACATGAGTAACGGGATCGTTGGATATTTTTATATTCGAGTATTTTTTTTCTTTTTAGGCTTTTTCTGCCTAACCGAGTACTTGGGGCGTTCCCCGTGGCTGCTCATGGCTCTGGGTCCCTTTAATCTTTTCTTTTCAGGGGTAGGTACTGTCCATAGAATCT----CGGGGAGTCCAATCTTCTGCTTGCATACCTGCCTGGACGTTTTTGCTTCATACTGATAAACTAGAGTTAGATTCGAAGGCTAGGTCATTTTACTATACGGTTCAGGTTCAGTATGTGGGTGTTTATTTGTATACGTCGTTGCAAGGCATTAATCAGCACAACGGTTGACTTCGCTGTATGCATTGTGAGTGTTGTTCTTTTTGTTCCTTATGTGTATCTGAATTGGTCCTTTGGTGTTTTGTGTCAGTTTACGGTTTTTTTCACCATAGAGCTCTTTC--TCTTGAAGGTCTTTATGATTGAGCTTGAGGTGTTGTCGAGTTCAGCAAGGGCTCTGTTTTATCTGCTATTCAATTGCGAGCTTGTTGGTGTTTCCAAA--CTGTTGGTTCGGATCTATTTTTCTATATGCATTCTTTTGGTAGTCTCCTGGGCAATTCTTTGGGGCACGTATACAACTTTAGGCCTCCTTGTGGTCGATTGTACTGATTGGGGGTTTCGTGGTCCCACCTGACGTTTTAGTCCATTATCTAGATGTTTCTTGTTGTATTTCAATACTTCTCAGAAAGGTCAGAACCTTGGTTGACTCATCGAAAGTTATTTACCTATTATCTAAGATTCTGGGGTTGTGCATACATTATTTCCTGGCTTATCCCGACTTTATGACCGAAATTATGAGCAACCTTGTACCCCCCAATCACCGATGTATTCTTTGCTAGCATTTCGGCGTCCAGAACTGGGGGTGTGCTTGTATAGATGTTCTTGTGTTTCTCT------GGAAAGTATCGCCGTTAGGTGCCGAAGTCTGTTATCCTGTGTAGGTCTATCTTCTGCTGGGCATCCGCGAGTCCTTGTAAGTAGCAGTGCAGGTATTATTACCTATTTCTATTTCAGGTTATACGTGTATTTATGTTGGTGCTATTTCTTTTGACGAAATCTGGCTACCACTCGCTGGCCAAAAGTGGCCACGGGTCTCACCTTAGGAGCCCAACTCTACAATCATCAGCCA--ATGA----CCTTATTAGCTTGTACTTCTTCTTTTCTTCTTACTATGTTAGATCCATAATTACCCTGAGTGGAAGGAAAATGGATTGATATATGGTTAGCTGTGTTAGGCCGTTCTGGGTGTTTCCATCAATTTCGTTTGGAGACCGGCATGCCATGATTCTTGACTGTCTTCGTATTTCTTCGTTTAGTTTCTCTGACTCGTCTGGATTGATGCCACTTAGCCCAATTCTACGCCC--CTCTGCTTGGGTTGATTCCTCTGCGAAGGGCGAGCCTTGATTCCGAGGGAAGGATTGTAGGGTTAAGTGATAGGGGGTCGTAATTTATTACTAGGAAGGTACCTATGTTCTGATATATAAAGTTTTTTGG--GTACTATTCTCCATCTATTGGTGTATAG--------ATCTCTCGTCAGTTCCCGATCGTTGACATGATGCCTGGTGGTACTTTTTCTCGCATTCTAGCTTTTGTGTGTGTGCCTGTGCGTTTGC----------------TTTTTCGTACCTTCTCCAGAGTGA--GGTAGACATCCCTTGATGGGGTATCCTATCACTCTATCCCTTTAGTCATTTATGATAGGTATTTTTGTTTGAGTGAATGTATTTTTCCATGTGAGC----TCTTCCGAGTAGGGGTGTTT--TGTTCTACTTCTCTTTTTATGGTTTTATGAATGGTAGGTCTTGATGAATTATTCTCTTATTCATGAAGTGTGTGTGTCTATTGCCTATTTACGTGGCT--GAGTGAAGCTATATTCGAATTTTCGTATGCTGGAAGGTATGGCTACCTAGTCAACTTCAGAGAGTGTATCTTCTCTGATTGAATGTTTATTTGACGGTTCGTATTTTTATATGTTTGTGTAGGCGTGTATGTTTGGAGTTTAATACTCTCTTCAGGGTGCTTACTTAGATTTATTTATGCAGATGTATTTTCGTACGGTCTTTTTGTTTTTTGGTGGAGGTTGGTTTCAGTTATTTATATTTAAAAAATGTCTCTCAATCGGTTATACTTTTATAACGTTGGGAGTTTATATGTGCTCTCTGTTCTATCAGGTC----------TTGGTCGGCTTC--------------GCCTGGAGGTGTTTACATGGTGTTTCAAAGGCCATTCCCTTTAGTGTTCCGAGCATGCGCCCACCCGTAGAGCTGTCATCTCACCTGTACGCGGATTAACCTGTCGTTGGAG--GAGTAGAATGGGTC--------ATGTGCTTTCTTTTTCTTGTGTTCGGCTTCGCCTGTTCACGATTAAAAGTGTGCTATCACCTTCCAATAGCTATATCATACTTCGAGGTGATGAGCTGATCTCAATGTATCAGCATTTAAAGCTTTGTTCTTATTCATGCGGCTCCTATTAGGGGGTTTCAAGAGT--GT------TTATCAAGCTAATTGGTTTTAGGCAGTTTTCTGTTCGTTTTTACAGTCTATCGTCGATATTGATTTTTAGCC--------------TCAATTGGTTAA------TTGGTTAAACGCCTTTTCTTTCTTAGCTGGGTTACTAAATATGTGTTTTGGCGTTG------ACCCTTTGATGGTTAT----GCCAGATCAGGTTCGTCTAGAAACCTATATAGGTGCTCCTGGTTTTAGTTGTTGTTGT--TAATAGTTACAA--AGTTGTATATTTTTGAATGCTTATGGGTATATAAGTCTCTTCTTTAGTACTCAATGCCTGCTTTCTCTCTGGGTGTGGGGTTATGTGTATTCGTATCTGTGTATCC--------GGCCAGGCTTCATGAGGTGAAA--------------GGTCACGTACTTGTCTGTCTATTTGCGTGCAT----------------AGTA------TTGAGATTGAAGTTTTAGCTAC--TAAGACTG------------CGTTAAATCT--TCAATCTCTTTTTACCGAGGGTTTGTCTCTATGGCTCTTTATCTCCGTATGAGTTTCTTCTTTAGGCGGCGGTCATACATTA----GTCATCAAGCTAAATATTTATTC-----------------------TAGACAGGTGTGATTAGTGGTTACGTGACTTTAGTTGCTTTTAATCGTGAAATTCACTTATAAGCTTAA--AAACACGGCTAAAAGACTAGGATTAGATACCCTATTATGAGTGTTTATGAAATGTGTCTAAGTCAGTA-----------------GCCTTTCCTTAGGCAAAATCTATTTGCTA-TGGCGGCTATT----AACCCTATCGGAGTCGTATGGACG--TAA-ACGAAGCTGCACGAG-CATTTTACCTTAACTTTACCGGTAGACAAGGTTGGTCTGGTA-----AGTCTTACACACCGCTGT--CAACTAAGGAACTT-----TTACAAGACTTTAGTCT------------------------AAGGTAAGTGA------------------TGGACTGGCCTGTGTGGACAAGTCAAGGTGTAGA--ATGTCAA-GGTGCTTATGTACGATTTAATTGA-----CTGAGTAAGTTGACATTAATTGTACTATGCAATCGAA-----------GCTAATAAAAATTCGACAGTAAGGTGACTTAGAGCATATTCATCTGAACTAGGTA-ACAAATAGTGTACAAATCGCCCGTCATTCCTACGGACAAGTCGTAACAAA-GTTGGTTTACCGGAAGGTGGTACCTTTAGCATCATGGGCTTAGGATTAATTAATTCATGCTTTAGCGGAGTATGTTTCCCGAAAGGAAGCGAGGATCTTGG--TTTGGTTCCTTCTCTTGTGACATTACGGCTAAGTAATCAAATCACGGAA--TGAAATGTGAGTCGGTTCTTCTGATTATCTGGTAATTAGGCAACCTAAAG--GGTTCGAATGTCTAGTAAAAACATTTCCTAAAGAAAATA---ATTTTAGGTATGACCTGCTCAGTGC--TGTAGATAGTAAATAGCTGCAGTAACCGTAACTGTAAAAATGTAGCATAATCAATTGTCTCTTAATTGGAGTCTAGGATGAACGGTCTAACGTAACCCTGTCTGTCTCTCTTTAATTACATG---AAGTT-AAATTCTAAGTTAAAATGCTTAGATGTAAAGATTGGACGAAAAGACCCTAAAGATTTTCGTTACCTGGGATGAG-GT--------AGTCAGTTCTAAGTAACGACTTTCAATATACAGCCAACACGGCTATGATGATCCTATTGAGATTAAATGAGTTTAA-TTACCTTAGGGCTAACAGCG--TGATAACTATTCTCA-GAACTTATGGGA-ATGGTGGTTTACGACCTCGATGTTGAATTGGGT-ACACTTTTCATAGAAGAATATGGA--AGAGTGA-GACTGTTCGTTTCTTAATTACCCACATGATTTGAGTTTAGACCGACGTGAGTCAGGTCAGATTCTATCCATCT-TCTTAAG-A---GACTT-TTCAGTACGAAAGGAC

Polyplax_asiatica ATTCATTTGACCAGCTGGGTGAGG----CTTCGT----AATGATTGGGTT--------------GCCCTTTTCTTTAGGGGGTTTGGCTTTTTCGGGTACGGGTCTTCATTTGGAACAGTTCAATCGTCTCCTCCTAAGAGGAA------CCTTGCTTAGCTATGGGTTAATGTCTCTACATAATTTTAGTTAGCCTTTCTTTGCCCCTCCATTAGTTGGCTAGGTTGTTCCCTTGGTGGGGTTATTCTC--TAAAAAAGAAGAAATTCTTCCATTCTCCCTGGACCCCAGGTTAGTGTTTTGTCTATGATTACAGCAGCGTCGCCTTAGCTAGGTCGCTATGCAACTACGCGGCAATATATAGCTGCGAAAGCTCTTCTTTCCTTTTTTAACTCATTTTATATCTTCCTTTCTGTTTGATTGGGTGCTTATCAGCTAGTTTATAGCTCTTCCTTATGGAATGCCCACTATGGTATTTCTTTTTATCAACATTTCTATGGAAAT--GGTTTATTTTTTGTAGAGCTGCGAGGCAGGGTAATCCAAATCTTGTAACAGATTTTCTTTTATCACAACAAAGAATGGGTTTTATTGTTTAGGCTGGCGGTTCTGGTAGGCTAGTTTTATCGTTGACTTGCACCGGAATTATGAGAGGCAGTTTAAAGTTGTACGCCAGCTTGTATATTTTTATGTATCCGCATGTGGGGTTGGAATGCTATCCTTATATGGTTCCGAATGCTTCCCGATAAAAATAGTTTGCTCTCCCCTCCTTTCTTTCTAGAGGCTTATCAGGGGGTGGACGGTGACGTTACCCCCTAGAGAGGTGGCACCGGGCTCGTGATTGCATTTAGCTCACTGCGGGTAGTCATCTGGGCATAATTATTCACATATGTGGTGAATTTC--CAATGAAACTCCTTTTTGTGGCATTTATACGCTTCTTTTTCTTCCTCCGTTTGCGGGCATACATCTTTTTGACGAATTAATGTCTTTTGACCTCGGGGGGGACCGTTTTACACATTTTTGTTTTGGCACCGAGTTAATTTATTTCCGGTTGGCTATTCCAATATTCGAGAATGGAAAAGAGTTTGGAGTTGGATATTAGCATAGGCATGGGCATGGTTGTGTTGGCCACAATTTACATGGATGAGTGAAGCGGCTATTACAGGCACATATATGCATCCACGGATAAGTTTAGTGCTGCACATATGGGGAAGCCCCGAGGTTCATCTTGGTGCGGTTATTTCTTTACGTGGGGCTACGGTTGTTTGCAATCTCATGAGTGCCTCAGAACTATAGTGTGCCATTCATAGTCTTCATGGGCGTTTGCATTTGGAGTTAACATGTTCCCTCTAGGGGTGTTTCCCAAATGATAAGCCATTTGGCACTTCTGGGTAATTACTTTTCCCACATTTTGGCTAGGGATCCCGCGTAAGGATACCGATCTTAGTGTGAAACCTTCTCGCGGAGTCATACTCGTAGATTTGTTTATGCTGCTTGGAGGCACTTCTGCGGTTCCTTGCAGGGC--TGGCAGCTGATGTTAGGGTTCCCCTCGACATCCAGAAGCCCCCAATTTCTGTAGCTCAGATCAATCCCTTATGGCAATGAAGGTCAGATGCTATGTGTCTGCGGATATTCGTGTATTAGTAGATTGACTTTTAGAAGATG------AAGTTTTTTTGAAGGATGTTGAGTATTGATATTTCCTCATGTCTCTACTTGCTTCCTCCTCATGCTTATTCTGAGAGTAGCTCCAATCACATAAGCGTGGCACATGTATGAGTAGATTGTTCCCACAGCTGAACTTTTTTGATCTACTTTCC--AAGATGGAACGGGCGCCCCGCTTTGATGGATCTCATCTCTCCGTGGGAGAACCGTTGTGTAGAGGGGAGTATCATCTGGCCTCCAGATGGGTAAGTGAGCATCCGGCGTTAACAGTATCTTACCTTAAAGGGATTCTTGGCATGTCGAATTGGGGCAACAAGTTATCCATAAGTGAGCATCCCGGAGATGATTCATCTAATTAGAATTCACCTTCAATGTAGGTAGCCTGCCTTCTTCAGGCTCGGTTACCTGCGTGGTTGTGAGCTTTC--GGATGGTGTTTTGTGGTGGGAGCTAGAGGGGCTTGCGGCTTGTGCGGAGCATCGGATCTTTTCTGGGACAACATGAGTACCGGGTTCGGTGGGTATCTTTATCTTCGAGTATTTTTTTTCATTTTTGCTTTTTTTTTCTTAACCGAGTAGCTGGGGCATGCCCCGAGGCTTCCCGTCCTAATGGGTCCTTATAAACGTCTCTTTTCAGGGATTCTTACTGTCCACAGCCTAT----GGGGATGCTTCGTCGCTTCCCTCTATAGGTCTTTGGGGGGTTTTCTCTCAGCGAGATAAGGATGTCTTTCATTCGAAGAGTTGGTCTTTTTTGTAGACGGTTCAGGGTCAGTATATGGACGTTTCTTCGTAACTGTCGGTATTCGCCATTTCCCCACACACTGGTTGAGCGGGCTGTATGCATTGTGAGTGTTGCTTTTTTTATACATTATGTGTTATTGAATAGGTCCTCTGGTTTGTTGGTTCACTGTACGGTTTTCTGCATCATTTCGCGCCAGGCTTCTTGAAGGTTTAGATATAAGAGTAAGGGGTGGCATCGCTCTCAGCAAGGGCTCTTTTTTATCTATTACTCACTGGCGGGCTTATAGGAGTACGCTAT--GGGTTGATGTGGGTATATGTTTACTTATGGACGCTTTTGGTAGTCTCCTGGGCAATTCTTTGGGGCACGTATACAATTGTTCGCATCCTATTGGGATCCTGTACTGATTGGGGGTTAGGTGGAACCACTTACCGATTTTCCTCATTATCTCCTTATATGTTTCTGCCTTCCACTTTCTCTCAGAAGGGAGTGAACCCTGGTTAGGAGATCGAAAGTGCTTCACCTATTATTCAAGATTTTGGGTTCGTTTGGAGTTACTTGCTCCTTTCTTTCCGATTTTATGACCGAAATTACCCGCAACCTTGCACCCGCCAATAACCGATGTATTCTTTGCTATCATCTCGTCATCCAGAACTGGGGGTGCGCTTGTTTAGATTTGTTTGCTTCTCCTT------ACAATCTCAGGGCGTTTCTTCTCATCTTACTTTGTTCATTAAAATTTTATTTACTGCTGGGGGCGCGTGACTCCTTACCTGTAGAATTATACTTTCTATTTCGTTTATAGTGCTCCTTCTATTCTTCTGCGTTTATATGTGCTTTTACCTTTGACGAATTCTGGATCTCAACCGAGGGCCAGAAGTGGTTTGGGCTTTCACCTTAGGAATTTAACTGTACAAACACCCAGCC--GGAAGGTTCCGCGGTACTTTGGCCTTGGATCTATCTAGGGTTGTTTGCTTTTCCTTAATTTCAAGA------AAGATTAGGCGTACGTTTATGTCTAGGTTCAGTTCCCTATATAGGGTGTTTCCGAGAATATCGTCTGGAGCTCGAGGTTCCACAATTCTTGAATCCCTTCACTCATATTCATGTATTTAACAACAGAGATCA--GAATTTATCAAGGGGTTGGTTCTTTTTGCC--ACCTCCGTCTATACTTAGCTAGGCGAGCTCCGTTCCTTGACTCCGAAGGAAGGATTGTAGGGTATCATGATAGGGGGCATTTACATATTTTTGCGAGCTGTCCTTTCTCTAGGGCTTTTCTCCTCTTCGG--AGATAGCCCCCTTCCTTCCTCTTTTTGC--------GTTGGCCGGGGTCTCCCGGTCGTAGACTATATGAGTTGTGGTGACTGTCCATGCCTTCACCTTGATGCAGTTCCGGTTGTATTTCTCTTCTTGTGCTTGCATGTTTGTGCTGATTTTCGAAAAAATTATCAACAATTTGAGCTTGATGGGTTAGCCATGGCACCCAGGCCCTAGGTCATTCTCTGTGGATTTTTCTATTTGAATGAGTATATATCTCCGTTTTTTT----AAGTAATTCTCATGGTTTTT--TGACTTTATTATATATCTTGGGTTCTGTGAGTGATTGGACATAGTGGGGCATACGCTTGTAGGCGAATATATAAGTAGTATGTTGGACATAGTTGAGGACGGGTCAGGTTATTGTTACATCTGTTTTCGTGCGAAGGTATGGCTACACCTAGACTCTTATAGAGGCCATTGCCCTCGACCTATTGGTTATCTGATGATAGTTCTTTCTTTATGTTTGGGTAGAGAGGTTTCTTATCAAGGTATTAAAAGAGACA--AAAATTTTCCATCTAGTCTTGTGTTCATTTGTTTGTTCAGAAGGTTTTATGCTTGTGGTGGACTCTGGATAGTCTTTGTTATTTTATTAATCTGTCTCTAAAGGGGCTGTACTTTTAGAACGTTGGGACTCTTTTTAGAGCTGGTTCTCTGTGGGGAGGATGAGTTCCCTAGTCCCGGCC----------------TGGGAGGGCTATCTTTGGGCATACAAAGGCCATACCTTTCGCTGCTCCTTGCATGCGCCCACCCGTAGAGTTGTCATCTCACTTGTACGCGGCTTTATGTGTCGCAAGCCTCACTTCCTCGTTCTGTTGGGT------TTGTTCTTGCAGGTATTAGCTGTCAGGCTTATGAGTGATTAAAAATATGCTTTCACTTTCCATTGGCTATGTCTTTGTTCATGGAGATGAGCGCCTGTCAATCTAGCAGCAGTTAAAGATACTTTCTTACTCATGCGGTTCCTTCTAGGGTTATTCAAGAAA--CTTTTG--AATTAATCCATGGAGGCTTTCCGCCTACTTTCCTGGACGTGGACGCGCTAAGCTCGATCTCTTTTTCGAATCCA------------GCTTAGCCTTTCTCAATCTTGGGATCGAAATGATTCCGGGATATCTGGCTATATAGTTTCCTTGATTTCTCCTG----------GGATAGGAGAAA----AGTCCCCTTTATGAAGAGACAATCTCGTTTATTTATCTATGGATTTTTGTTTTTTCAT----CATGATACGA--AGGTGTACAGAGATTTATATCTTTTCTTGGATTCTGGAGTTTAACTAGAGTCGTTGCCTCATTTTTTTCTGGATTTGGGGCTGTGTCTCTGCTATCTTATCTTTTC--TG--AAGACAGGAAGCTTTCAAGGTTGA--------------TTAGGGCTCAATGTGTTATCCCCTGCTTATCT----------TGATCTGAAAGATCCTACCTATGGGGTCAGAGAATTCACCTCGCTGAG------------GTGCTCATAG--TTGATAAGAACATAATTATCATCTTTTTTTAGACTTTTATATTTTTTCATGAGAATATAAAATTAAAAGCGGTCAAACACGAAGGCAAAATCAC--TGAA--------------------------------TGTCTGGGGAGACTTAATTTTGGCAGAGGATAAA---------CAGGTAGAATTGTGTCAGAAAT----------AATAAGTTTAAACCAGGATTAGATACCCTGTTATTTTATTACGTA----------ATCCCATACCCCAAGTAATTAA----AGAGTTTCCTCTCCTAACTCAAACTAGTCATGGCGGCCCGTT----ATCCAGTCAGAGGTGCATGTCCC--TAA-TCGAAGCCGCCCGAA-TATCTTACCTTC-----------------------CCTGTTA-----TGTTTTGTACACCGCTGT-----GTAAGACTGCT-----AAGAAAATTTACGGTCT------------------------TC---------------------------TCCTATGGGGGAAAAGCTCAAGCCATGGTGCAGAGAATGGGAA-GGGTCGGATGTGCGCCA----GTG-----ATTGATTTCACCCAGGAGCTTGAAATAGAGCGT--------------TCTGTAAAAGAATTTGATAGTAAG-AGTAAATCATTAACTTACTCTGAATGTGGGACATAATGGGTGTACAAATTGCCCGTCATTCCGTCGGACAAGTCGTAACAAA-GTTGCCCTACTGGAAAGTGGTACCTTTTGTATCAGGGTTAGAGGATAAAAG-ATAATTAGATTATTAA------ACTCCCGAAGT--GA-AAGGATTTTGTAGGGCTGGGCCTCTACT-GTTGAATTAGTGG-GGAAAAAGCCCTACACGATG-CGAAAAGTAATTCGTCTTTCATGGT-ATCTGGTAATTCGGCAATTAAGG---GTTCAGACTGTTTAGTAAAAACATTTCCTGCCCCAA----AAATGGCAGGTAAGGCCTGCTCCCTGTC---TCTTTGATAAAGAGCCGCAGTAGCT-TGACTGTGCTAAGGTAGCATAATAATTTGCCTCTTAATTGGAGGCTGGAATGAAGGGTCTAACGTGAGCCCAACTGTCTCGATTAGGAAATAGTT--GAAGTTTAGTTCTGGGTGAAAACTCCCAGATAGGATAGAGGGACGAGAAGACCCTGTAGAGCTTTTTTACCTGGGAGGGGGAGGCTGATTAAGACCAGTCTATAACTTTAAGACTAATCGTCAAAATT---ATCCATGATCCCGTATTTTCGATCAGT--TGAAAAAGTTACCTCAGGGATAACAGCA--TAATATCTCTCCTTTAGTTCTTATGGAGTGAGGGAGGTTATGACCTCGATGTTGAATTAAG-TTCTCTTCGCTATGTAAAAGGAGTG-AATTGTTA-GTCTGTTCGACTATTTAAAACTTACATGATTTGAGTTTAGACCGACGTGAGTCAGGTCAGATTCTATCTTCTA-TCAAATTATCCTTTT----TTAGTACGAAAGGAC

Polyplax_spinulosa ATACATTTGACCTCTC----TTAATCCTGTCCTT----AATGGGAGAGGT--------------TTTTCTGTATTTAGGGGGTTTGGTGTAGACGGTTAATTCTGTGAATTTTTTTAACTTGCAGGTTAAGTATTTCTAATGAA------CACATCGCATTTATGGCTTTTATTATTTACATAAGTGTGGCTTTCCTTTCTTTCGTACGCCATTTCTGAGTTACATTGCTCCATTGTTGGGGGCATTAAT--TTTCAAGAAGGAGGGCTTGCCATTCTCCCAAGGCCCATGGTTGCCCTTTTGTATGTGAATGTAGATCTATCGCCTTAGTTAGGTCGCTATGCAAATACGCGGCAATATTTAGTTATGAACCTATGTAG--AATCCTTCGTAATGTTACCTTTATGCGGTTCTCTTTGATTGGGTGCTTATCAGCTAGTCTATAACTTTTCCTTATGGAATGCCCATATTGTTTTTTAGTTATACAATTATTTCTTTATATGT--TTGTACTTGAAATTTTTTTGTACCGAGGGAAAAATATCCCAATAAAACTGAGAAATTTATTTTCACAACAAAGAATGGGTTTTATTCTTTGGTTTGGCGGTTGTGGTTGGATAGGTATATCGATGACTTCCACCGGTTTGATGAAGGGCAGTTTAATGTTGTACTCCAGCTTGTATATTTTTATGTATCCATATATGGGGTTGCAATGCTGTCCCTATCTAGGCCCGAATGCTTCCCGATAAAAATAGTTCACTCTCCCCTCTTGTCTCTTTTTAGTCCTATCAGGGGGTGGACGGTGACGTTACCCCCTGCAGGGTTGGCACCAGATTCGTGACTGCATTTAGCTCATTGCGGGTAGTCATATGGGCATAATTATTGACGTATAATTTGAACA----ACATGAAGTTCCCTTTTGTGAGGTCTATACGCGTCTTTCTCTTCTTCCGTTTGCGGGCATACATCTCTTTGACGAAGTAAAGTCTTTTGACCTCGGGGGGGACCATCTTACACACTTTTGTTTTGGCACCGAGTTAATTTATCTCCGGTTGGCTATTCCAATATGTGAGAAGGGAAAAGAATTTGGAGCTGGATATTAGCATTCTCATGGGTATGGTTGTGTTGGCCACAATTTACGTGGCTGAGTGAAGCGGCTATTACAGGCACATATATGCATCCACGGATAAGTTTAGTGTTGCACCTTTGGGGTCTCTTCAAGGTTCGGCTTGAGATGGTTATTTCTTTACGTGGGGTTACGGTTGTCTTCAATCTCATGAGTGCCTCAGAACTATAGTGTGCCATTCATAGTCTTCATGGGCGTTTGCATCTGGAGCTAACATGTTCCTTGTAGGGTTGTCTAACAAATGATGCATCATTTGGTACTTCTAGGTAATTACTTTTCCCACATTTTGGTTTCGGATCCCGCGTAATGATACCGATCTAAGGCTGAACTACTCTCATGGAGTCCTTCATGTAGGTGGATTTATGGTCTTCTGAGGTTATGCAACGTTCTTTTTCCTTTA--CAAGAGGTGAAAATTTGGACCCCCAGTTCAAGAAGAAGGCCCGCTATCATGTAGCTCAGATCAATCCCTTATGGCAATGAAGGTCAGATGCTATGTGTCTGCGGATATTCGTGTATTAGTAGATTGACTTTTAGAAGATG------AAGTTTTTTTGAAGGATGTTGAGTATTGATATTTCCTCATGTCTCTACTTGCTTCCTCCTCATGCTTATTCTGAGAGTAGCTCCAATCACATAAGCGTGGCACATGTATGAGTAGATTGTTCCCACAGCTGAACTTTTTTGATCTACTTTCC--AAGATGGAACGGGCGCCCCGCTTTGATGGATCTCATCTCTCCGTGGGAGAACCGTTGTGTAGAGGGGAGTATCATCTGGCCTCCAGATGGGTAAGTGAGCATCCGGCGTTAACAGTATCTTACCTTAAAGGGATTCTTGGCATGTCGAATTGGGGCAACAAGTTATCCATAAGTGAGCATCCCGGAGATGATTCATCTAAAAACAATTCACCTTCAATTTGAAAAGCCTGCCGTATATAGTTAAATTTAGGTGCTCCTCTTCAGTCTTTT--TGGGGTTCTTCTTTATGTCCGTCTGGCTTTTTTCGTTCTTTGTGCGGAGTGTGCGAAGCTTTCAGGAACAACAAGAGTGTAGGGTTCGGCGGGTTTATTTATTTTCGAGTATTTTTTTTCATTTTTGCTTTTTTATTCCTTCCCGAGTAGGTGGATGATACCCCGTGGGTGGTCTTAAATTTAGGTCCTTTTAAACATATTTCTTCAGGGGTTCCTACTGAGCACATCTTCT----GAAAAATTTTAATCAACTGGCTTTATTCATTCTTGGTCTGTTCTTTCTCAAAAAGATACTGATGCCTTGAATTCGAAGGTTTGGTCTTTTTTATGGACGGTTCAGGCTCAGTTTATGGACATTTCTCTATAGTTATCGTCATATGGCATTTCCCTGCATGTTGGTTGAGCAGGCTGTATGCATTGTGAGTGTTGCTTTCTTTGTACGTTATGTGTACATGAATAGGTCCTCTGGTTATTTTTATCACTTTAGGGTTTTTTGCTTCATAGAGGTCTCATTTTCTTAAAGGTATTCTAATCAAGGTGAGGGGTGGCATCGTTGTCAGCAAGGGCTCTTTTTTGTCTATTATTCAATGGCGGGATTATAGGAGTAAAAACT--CTGTTGGTACGGGTATATATTTATTTATGGACGCTTCTGGTAGTTTCCTGGGCAATTCTTTGGGGCACGTATACAACTCTTCGCTTCCTAATGGGAAGCTGTTATGTTTGGGGGTTAGGTGGAGCCACTTACCGATTTTCATCATTCTATCCTTATTTCTGTGTGCCTTCCAATGCTTCTCAGAAAGGAGTCAACCTTGGCTAGCCCATCCAAAGTGCTTCACCTATTGTGTAAGAGTGTGGCTGTGTGTGGTTCTTTGGGCATATTTCTAGCCGATTCTATGACCGAAATCATGAGCAACCATGTACCCCCCAATCACCGATGTATTTTTTGCTAACATCTCGTCGTCCAGAATTGGGGGTATGCATATATAGATTTATTTTTATTTCCTT------TCGAGCTCTCGGCGTTAGCTATCGAGGTAGACTGATCAATAAAATTTTCTCTACTGCTGGAGATCCGTGAGCCCTTGAATACAGAATAGTACTGTTTATTCTACTTCTTTTGTTGCGATTATTTTTGTTCTTCTCTATGTGCTTTAACTTTTGACGAAGTATGGCTTCCAAGCGCTGGCCTCAAGTCTTTAAGGGTGGCACCTTAGGAGTATAACTTTTCAAATTCCTTCCG--AGAAGAGAGATTTGTAACCTGCCCTGATATATATGTTCGTAGGTTTGGGCTTCCTTTATTTTCCTC------TACAACTCGGGTATATATTTTTTTAGGTAGGCTTTGCTACTTTCGGTGTTTCAATCTCTATCACTTGGGCTGCGAGGTTCCATCTTTCTTGAATCCTTGCCTTGTTATTCCTTTCTATTCAAAGCTAGGTCG--GATGGG--CACTGATTACGTCTCTGTGCCC--TGAGGGTTATCTTTTTTCTTATGCGAGCGGCGAGCCTTGACTCCGAAGGAAGGATTGTAGGGTTAAGTGATAGGGGTTCTTTACCTATTTCTAGGAACCTATATCTGTATAGAGATTTTCATATTTCTCA--CATGGAGGTGCGTTGTTTTCTTTTATTT--------ACCTATCGCCTCGTCCCGATCGTTGACAGCATACGCGCTGCTTCATACCCACGCATAGGCGTTTTTTTGTCTATGCTTTGACTTCTATTCCTCTAG--------GCGTGCCTCTTTCGAGAAAGGGAA--ATATTCTTGACATTGATGGGTTACCCTTCACCTCCGATGTTTTCATCATTTTCTATGGGTTTTTTTATTTGATTGAGTGTTCACTTCCGCATTTAT----CAGTTCATACCATGGTATTT--TGGTTTTATTATGTATCTGTGGCTGTTTGATTTATGGGACTTCTTGATATCTTCGTATGCGGTTGATTATTCTCTTATTTATGATTTTACCAAATTTTTCGGTGGTGTCTGTGCGTATTTGTACATGTGCACGAGGGTATGGTTAGATCTAGGCTCATATCGCTGAAGTCAGCTACGGGAGAGTTCTTTGTTGACAACAGGCGTTTTTTTATGTCTGTGTTCACAGGTACAATATCAGTATAAT--AAAGAAGATTCTAATTCTTCCTCTAGTTTTATCTTCATATTTTTTGTTAGACAATTATTGAGTTGTGGTGGAGGTTGGCTACTCTTTTTTATCTTATAAGAATGAATCAGACGGGGCTGTACTTTTGTAACGCTGGGATTTTCTCTTCTCATTTTTCTAGGTAGAGGG--ATTTTGATGGGGAGAAAAGA--------------AGTTTTGGATTTTTATCTGGGTTTGCAAAGGCCATTCCTATCTCTGCTCCCTGCATGCGCCCACCCGTAGAGTTGTCATCTCACTTGTACGCGGGTTTATATGTCGAGTATT--CATTAGGAGCACCTTTCT----ATAAATATTCTTTTACATTTTATCGGCTAGGCATGTGACAGATTAAAAATATGCTATCACCTTCCATTAGATATGTTTTTATTCATGGAGCTGAGCGCTTTGCAATTTATCAGCCTTTAAAGATTTGCTCATATTCATATGGATCCTTTTAGGGTTACTCAAGAAT--ATATAG--ATTCTTAGAATTGATCTTTGAAGTTTGGATATAGAGATTTTTTCTCGCTATCACCGATGTCTTTATAGCAAGGCCA----------CCAGGTTCTTAG----GGTTTCAACCAGACAAATTGTCTCCTTGGTTCTTAATTAACTCTAGGGACTTATTTCC------------ATCTTTCCGACTTGCCCCGCCCGAGTGAAGGGGTAAGTTTGTGTTTTCCTCTACGGATTTCTATTTGTACTT--TTCTGCTTGGGC--TCCC--CAGGGTGTAATTATGCATACTTTTTCGGTTTTATTTGCTAGCGATCGTTGCCGGTCATTTTTTTGGATGTGGGGTTGTGTCTATAGTTACTTATATTTCCAAGAAGTAAAGAAGTTAATT--AACTTTAA--------------CATCTGCTACATGCCCCTGG--ATTTTTTTCTTCTGGAACGGGAAGACTCTGGCAGGATCAAGCTCTCTCCTAGCCTCTC----GTTGAG------------ATAGGAAGCT--------ATCTTTTCCCTTTTTTGTTTATTTATTTTTTTATATCTCTGCGTGATCGTACAATT--ATGGGCGGTCAGACATCT------GGGCAAA-TCAATTTTTCTTGGAAAAACA---------------TAAATTG------------GACAGTGAAACTTACT---GCTTAAAGGTGAAATGTTGGTAGAAATTGAA-------------ATAAACAGGGATTAGACACCCCTTTATAGGTTTGTAAG-AATAATTTCCAAAGCTAATGTTACAAA--------GATGTGG-----TTAAAATTTATTTATCA-TGGCGGTA----GAGAGTCTATTCAGAGGCATATGTCAG--TAA-ACGAAACTGCCCGAA-CACTTTACTTTT-----------------------TTAAATT--------CTTGTACATCGCTGT--TTAGAAAG-----------GTAGAACAAGTCTGTTC------------------------TGCCCAACTAG------------------ATATAGATTTTAGTAAATCAAGTCAAGGTGCAGAC-ATAAAGA-AGAAAAGATGTATGTCATTGCTCC-----A-----------------AGAGAAGGGAATATTGAAAACTTTCCC--TTTTAA---GAATTTGATAGTAAG-GGCCACTCACTATTTAGCCCTGAATGAGGA--ATTCTCTGTGTACAAATCGCCCGTCATCCCGATGGACAAGTCGTAACAAA-GTTGACTTACTGGAAGGTGGTACCTTTTGTATCAGGGTTGGAGGA-GAAAG-GCAATTT-ATTAATTG------ATTCCCGAAAA--TG-AAAGATTTT-TAAAACTGCAAAGTTAAC-GTTCAAATGTTAA-GGAAAATGGTTTTAAGGGAG-CGAAAAGTTAGACGATTTCATTGAT-ATCTGGTAATTCGGCAAA-GAGG---ACCTGGAATGTTTAATAAAAACATTTCTTTTATAGA----AAATTATAAGTAAGCCCTGCTCACTGCT---TAG--AGTAAATAGCCGCGGTATTT-TGACCGTGCTAAGGTAGCATAATAATTTGCCTTTTAATTGAAGGCTAGAATGAAAGGGTAAACCGAGGTTCAACTGTCTCTTCTTAGAGAGAA----GAATTTCAACTTTGAGTGAAAATGCTCAAATGTGTTAGAGGGACGAGAAGACCCT-TAGATCTTTGTTACCTGGGAGGGGGCTA-TACTTCAAAC---TTTAGGTTACTAAAAGAAAT---TTAATTC---AAATATGATGATCCTTGTTGGATAAA----AGAATAGATACCTAAGGGATAACAGCG--CTATTTTCTTTTTTTAGACCACTTAAAATAAAGAAGCTTGCGACCTCGATGTTGAATTAAG-TTAAATTGAAT-TAGAAAAAGAATT-CTTATTTA-GTCTGTTCGACTATTAAAAACTTACATGATTTGAGTTTAGACCGACGAGAGTCAGGTCAGATTCTATCTTCTA-TGATATTTTACCTTT----T-AGTACGAAAGGAC

Haemapinus_apri ATTCGTTTGACCTGTCACTTCTAGTTAATTCCTT----AATGTTCTGTGT--------------TTGTGTTTAGTTAGGGCGTATGATCTTCTCGGTTCATGGTATGTTGGTAAAAGGCTATCAGGTTCGGATCTAAAAATAAA------CATTATCTATCTCAACTTTTTTTTATTTTCAGAATTATGGTTTCCCTTATTTACCTTCTCCATTGTTAAATTAGTTTGTTCCTTTGTTGGGGATTTTATC--TGTCAATGTGAAAAACTTGCCATTACCCGTGGAGCCGTACTTGCCCTTTTGTTTGTGAACGTAGTTGTATCGCCATAGTTAGGTCGTTATGCAAATACGCGGCAATGTATACCTGCGACAGGGCATTCGTGCTCTAGTGGAGTTTAGTTTTTGTATGTCTTTCTTTGATTGGGTGCCTATCAGCTAGTTTATAGCTATTCTTTATGGAATTCCCATTGTGTTCTCTGAGTTGTTTTGTAGTTATAGTGTGAG--TGGTTATGGATTTTGTGAAGTTGCTCCCAAGTTTATAAGTCCGTTTAAGAGGGATAGTTTCTTCACAACAAAGAATGGGTCTTATTATTTGGGTTGGCGGTTTTGGACAGATAGTTTTATCGGTGATTGGAGGTAGAGTTATTCAGGGCAGTTAAAGTTTGTACGCCAGCTTTTATATTTTTATATATCCGTATATGGGGTTGGAATGTTGTCCACATTTGGGCCCGAATGCTTCCCGATAAAAATAGTTTGTTTTCCCCTCCTTTTTTTATTCAGTTATGTGGGGGGGTGGACGGTGACGTTACCCCCTAGGGTTGTGGCACCAGAGTCGTGATTACATTTAGTTCATTGCGGTTAGTCATATGGGCATAATTATTGACATGTAAATTGGTTGGGAAAATTGACTTTCCTTTTTGTGTCGTTTATACGCGTCTCTTTCTTCTTCCGTTTGCGGGGATACATTTTTATGACGAAATAATGTCTTTTGACCTTGGGGGGGACCGTCTTACACACTTTTGTTTTGGCACCGAGTTAATCTATTTCCGGTTGGTTATTCCAATATATGAGAAGGGAAAAGAGTTTGGACTTGGATATTAGCATGTGCATGGTTTTGGTTATGTTGGCCACAATTTACGTGGATGAGTGAAGCGGCTATTACAGGCACATGTATGCATCCACGGGTAAGTTTAGTGTTGCACCTTTGGGGAACTGTATTCGTACTTTTTGTGTTGGTTATTTTTTTACGTGGGGTTACGGTTGTTTGCAATCTCGTGAGTGTTTCAGAACTATAGTGTGCCATTCATAGTTTTCATGGGCGTTTGCATATGGGCTTAACATGTTCCATATAGGGGTAGTTAACAAATTATAAGTCATTTGATACTTATGGGTAAACACTTTTCCCACATTTTGGTTAGGGATCCCGCGTAGTGATACCGAGTTTCTTGTGAAATTTTCTCATGGAGCTGTAGGCGTGGGTCTTTTTGTTAACATTAGAAGCTGTAGAACGGTATTTTAAGTTTC--GGAATCTTGAGCATTTGGTGCCCCAAGCCAACCAGAAGGTCCTTGTTTAAATGGTTCAGAAGTCTCCCATATGGTAATACGGGTCAGATGATATATGTGTTTGTGTGTTCATGTATTAGTTTGGGGATATCTACAAGGTG------GACGTTCTGTAGGCGAACTTGATTATTGGCGGCTCCGCATTCTTGGTTTTGCATCCTCCTCATGCTTATTATGAGAGCTATCCCTTTTAGTTAAGTGTGGCACATGTATGTCTAGATATCGATATCGATTGA------TTGATCTAATTTAGCAGATCTT----------TTCGCTTTGAGTGAAAGCGTGCATCCATGATGGAGTCGGTTTGTACTCGGGAGTATCATCTGACGTCCTCATGGGTAAAGGAGCATCCGGCGCTAACACTGTTTATGGTCAATTGGTCTATAGGCATGTCGAATTGGGGCAACAAGTTATCCATAAGTGAGTCTTCAAGATTTTATAATGTTTTAAGGGGTTCACCTTCACTGTAGCCAGCCTGCCCTTTTTAGATTCACTTTCCTATGTGGTTTAACTGATTC--AGATGGAGGTTTATATTTGGGTTTAGGTCTTTAGTTTTTGTGTTCGGAGTATCGGAAGACTACAGGTGCAACATCGGTATAAGGTTCGTTGGATGTATTTATATTCGAGTATTTTTTTTCATTTTTGGGTTTTTTTTCTTAACCGAGTGTTTGGAGTCTACCCCGTGGATCACCTTAATAATGGGTCCTTTTAAACATATTTCTTCAGGGGTACGTACTGTGCACAGGATAT----AGGGAAAACACAAGGTGTGGTTATATACGTATTTGGGTTTTTGTATTTCATTGAGATATAGAAGTCTAACATGCGAAGGTTGGGTCTTTTTAATTCACGGTTCAGGATCAGTATTTGGACGTATTTATGTAGTTGTCGTTATATAACATTAGAGACCAAATTGGTTGAATTCGCTGTATGCATTGTGAGTGTTGTTTTTTTTATTCATTATGTGTACTTGAATAGGTCTTTTTTGTTGTTGTATCAATGTAGGGATTTCTTCATCATAGAGCTCATCTAAGCTTTCAGGTGTTCATGTAAGAGTAATGGGTGCTATCGACGTCAGCAAGGGCTCTTTTTTATGCATTAATCAATGGCGGGCTTATAGGAGTACGATGT--GGGTTGTTGTGGGTGTTTTTTTTTCTATGCACGCTTTTGGTAGTTTCCTGGGCAATTCTATGGGGCACGTATACAATTCTTCGCATCCTATTGGGAGTATACGGTGTTTGGGGGTTTCGTGGAACCACTTGTCGTTTTTCTTCATTGTTTCCTTGTATTGTTTTGTTTTTCATTATTTCTCATGTTGGAGTCAACCTTGGTTTCAAAATCGAATATTATTCACCTATAAGGTAAGAGTCTGGTTATGTGCTTTTATGTTGGTGTGTCTCTTTCCGATTTTATGACCGAAATTATGAGCAACCATAAACCCCCCAATCACCGATGTATTCTTTGCTATCATCTCGTCATCCAAAATTGGGGGTGTTCTTTTGCAGGTATATTTGCTTCTCCCTTA----GCAAGGTTAGTTCGTTATGGTTAAAAATTTTATGTTCAGTATGTTTCTATTTACGTTTGGTCATCCGTGATACCTAACGTATAGCAGTGTGCTTATTATTGTAATTATTTCTGTCAATTTTTACATATTCATTTATGCGTGCTTTTTCCTTTGACGAAATTTAGATTCCAAACGGAGGCCAAAAATGTCTAAGGTTTCCACCATGGGAGCATAACTTTTCAATCACAGCTCC--AATTGGTTTATCGTTAACCTGGCCCTGCCTCTAGATAAACTTTTTGATACACCC----TTCTTCAATTATCATTAACATCGGATGTATCTCTATTTAGGTACGCCTCCACATTAAGGGTGTTTCAATCACTTAGACATGGGCATCGTCGTGCCATCCTTCTTGAATACTTAGTTAGCTTTATAGTTCTATATCATCTTTGTTGAAACTCCAATTCAAGTGAGTG--CTTTTGTGATCCTGATTCCTGTACTTATTGTTTTGCGAAGGGCGAGCCTTGACTCCGAGGGAAGGATTGTAGGGTAACATGATTGGGGTTCATAACTTATTTCTGGGAAACTGCGTATTTATACATATTTTCACACTACTGG--GGTTTCTTTGAAGCAGATTTGTTTATAT--------GTATATCGAGTCTACCCGATCGTAGACACTATCATTAATGGTGGATTTCCCACTATTCTCGTTGTTGTCTGTTTATATATTTTTTTTT----------------GTTTTCGTTTATTCTCATTCGAACAGTTGAACAAGATCTTGATGGGTTTTACGGAGGAATCATTCCTTTGGTCATTTTGTGTGGATCTTTGTGTTTGAATGATTGTGTTCCTCCCTATATGC----AATTAGGATTGTTGTTTTTG--TGTTGTTAAGATATTTTTATGGATCTTTGAGTATTGGGTCATGATGGGATATATGCCTTTAGGTGATTCTTCGTAGCATTTAGCGTCTTTCTGTAACCAGTCTTAATTAAAGTGTTGTTTTTTCATCTGTTTGAGGACTTGGTTACATCTGTTCACAGCTAAATGATCACATATTGGAATGATGGTATGTGTGAGGTTAGTCTTTTTTTTACGTTTCTGTAGTTATGTTTAATAAGGTCATATTTCCATTGAGGTTAAAATTATGTACCTTTTTTTGTATTCATTGATCTTGTTAGCCAGATTTTGGTATGTGGTGGAGGTTGGCTACTCTTGGTTATATTTTACAAATGAGAGTTAGAGGGTTTTACTTTTATAACGATGGGAATTTATATTCGTATGTATCTTCAG--------TGAAATTTAAAGATTCATAG----------TCGTGTCAATTCGTTTTTCTTTGGGCATACAAAGGCCATTCCTTTCTCTGCTCCCTGCATGCGCCCACCCGTAGAGTTGTCATCTCACTTGTACGCGGATTATTCTATCGTTGAAG--TTTTCCTTGAGTTTCAGC----CTAAATGTTCATATACATGTTAGCGGGTTCGCCTTGGAGTGATTAAAAGTGTGCCTTCACCTACCATTGGATATACTTTAGTTCATGGAGGTATGCGCACACCATTGTTTCAGCTTTTAAAGGCTTATGTCTTCTCATGCGGCTCCTTTTACGGTTTATCAAGAGT----------ATGTATTTGCGAGATCTTCTACCTGTTCTTTTTTGGGTATCTACTCGGTATCGTCGTTATGTTTATTTAATC--------------CCAATTATTCGAAAAAGATTATCAGTAGGAGGTTGTGGGTAGGTAAGGTTTTATAGGTCTGGGGAGGTTTCTTG----GTGTATCCGTACATGTAT----AATAAATTAGTGGGATTTAAAACTTTGTATGTTCCTGCGGGGCTATGCGCTTTGTCTT--TATTTGATGCTC--AGTGAGATGTGTTTTCTTTCGTCAATGTACTAAGGTATTTGCATAGGTACCATATGATGTATTTAGATATGGTTTTGGGGTTATGTTTCTTCTTATCTATATCTCC--AACCGATTGGGTTTAAAGTGAA--------------------TAGTGGCTTCATCTATTTTTATATTCACTGAC--------------------------------------TACTCCGTGTGAAAAAGACC------------GATTTTCCAT--TCCTCATTTGGCTAGGTTTGTTTTTTTTCTTTGTTTCTATATGTGGATATAATAGGTAAATCATTACTGCGGTCATACACTA-----AGCTCAAGTTAACTTTCATACTGAT--------------------TGTACTAGTAACATGTAGTAGTAACTAAATTTAA----GTAGAAAGGTGAAATAAAGTAAGAAATTGTA---------ATCAATAAACTAGGATTAGATACCCTACTATA-TACAATGTA----AATATTATATCAAGGAAGTAATGGTTAT----GGTCCAT-------GAAAACTAATAACATATGGCGGTTGAC----AATCTTTCCAGAGACCCATGTTTG--TAA-TCGATAATGCACGTC-AATCTTACCCTAT----------------------TTAAG-------AATTTTGTACACCGCTGT-------CGGAAGGTT-----GTGGATAATTT------------------------------CATAATCTAAT------------------ACTATCAAAGTGATAAGTCAAGTCAAGGTGCAGACTATGGTAG-GGGTATAGTGTGGGTCA-------------------------------TTGGTCTATTGAGCAGACATAT------TATTGAAATGAATTTGGTAGTAA--ACTTAAAAATTATATTGAGTTGAAATAGCA--ATTGTTAATGTACAAATCGCCCGTCACTCCGTTGGATAAGTCGTAACAAA-GTTGATCTATTGGAAAATGGTACCTTTTGTATCAGGGACTTAGGAATAAGAAACAGTTTATA--CATA------TATCCCGAAAA--GAAAAGAATCCTTTAT---ATTAGAGGTTATTGTTTAATTAATATCTTTAAATTAT-AAAGAGTGG-TGAAATGTCATACGATTTTCTAGTT-ATCTGGTAACTCGGCAAACGTTT---AGTCTGACTGTTTACTAAAAACATTGCCTTTAGAAG--T-AAATTGAAGGTAAGACCTGCTCACTGCGTT-GATAACGTAAATAGCTGCAGTATTC-TGACTGTGCTAAGGTAGCATAATAACTTGTTTCTTAATTGGAATCTAGAATGAATGGTTTTACAAGACTAAGTCTGTCTCTGTGAAATGAAATT---GAATTTTAATTTTAAGTGAAAATGCTTAAATGCGTTAGAAGGACGAGAAGACCCTATAGATTTTTTTTACCTGGGAAGGGGTCTATACT-CAT-AAGCTATGTTTAATAAAACTAAAATAGTAGGAAATTAAATGATGATCCTCAAGTTGTGATAAAA---AGATAAATTACCTTAGGGATAACAGCA--CAATTGTCTTTCCTA-GATCCTATTGGACAATACAGATTGTGACCTCGATGTTGAATTAAG-TTATCATTTAGATGAAGAAGGTTAA--ATGGTTA-GTCTGTTCGACTATTAAAAACTTACATGATTTGAGTTTAGACCGACGTGAGTCAGGTCAGATTCTATCCTCTG-A-AATTAAGTTTACTCT-TTTAGTACGAAAGGAT
